# Highly Cα-regio-, enantio- and diastereoselective Mukaiyama-type annulation of siloxyfurans: stereodivergent synthesis of multi-stereogenic tricyclic γ-lactones

**DOI:** 10.1039/d6sc01491g

**Published:** 2026-04-29

**Authors:** Lifei Gan, Zi-Qing Li, Tao Chen, Xuanchen Wan, Junyang Zhang, Jiangtao Ren, Ming Jiang, Penglong Cao, Jinhai Huang, Yu-Hua Deng, Fangzhi Peng, Run Tian, Yingcheng Wang, Zhihan Zhang, Zhihui Shao

**Affiliations:** a Key Laboratory of Medicinal Chemistry for Natural Resource, Ministry of Education, School of Chemical Science and Technology, School of Pharmacy, State Key Laboratory for Conservation and Utilization of Bio-Resources in Yunnan, Yunnan University Kunming 650091 China dengyuhua@ynu.edu.cn ycwang@ynu.edu.cn zhihui_shao@hotmail.com; b College of Chemistry, Central China Normal University Wuhan 430079 China zhihanzhang@ccnu.edu.cn; c Yunnan University Affiliated Hospital, Yunnan University Kunming 650000 China; d Southwest United Graduate School Kunming 650092 China

## Abstract

The first Cα-selective asymmetric reaction of 2-siloxyfurans, a class of versatile nucleophiles, has been developed with both high enantioselectivity and diastereoselectivity. Moreover, by changing the achiral co-catalyst to a newly developed combined co-catalyst, a rare diastereoselective reversal was achieved, selectively yielding the thermodynamically less stable diastereomer. DFT and experimental studies reveal that the observed Cα-selectivity results from dispersion and electrostatic interactions between 2-siloxyfurans and the electrophile/catalyst, while the diastereodivergent synthesis arises from a divergent C–O bond formation *via* dynamic kinetic lactonization-driven epimerization. This work not only provides a method to overcome the challenges of Cα-selective asymmetric reactions of 2-siloxyfurans, but also offers a stereodivergent synthesis of chiral tricyclic γ-lactones. Importantly, the resulting chiral tricyclic γ-lactones are not only the core structures in natural products and bioactive molecules but also serve as an appealing platform for diversity-oriented synthesis (DOS), streamlining the construction of other valuable enantioenriched compounds.

## Abstract 2

We disclose a catalytic asymmetric Mukaiyama/lactonization reaction of 2-siloxyfurans with outstanding Ca-selectivity, enabling achiral Lewis acid-directed diastereodivergent access to important multi-stereogenic γ-lactones.

## Graphical abstract

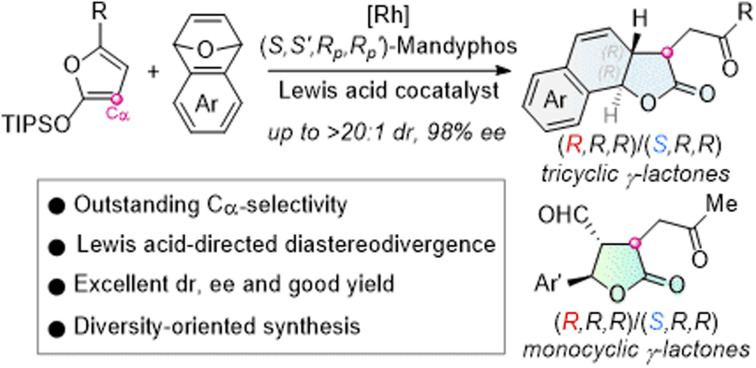

## Introduction

**Fig. 1:**
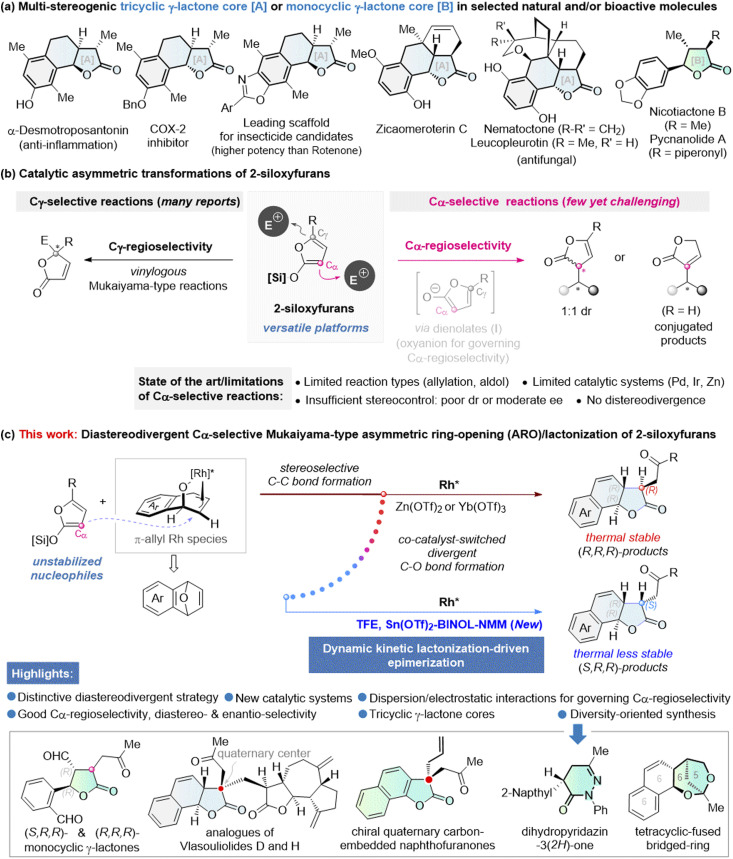
Previous studies and this work. (a) Multi-stereogenic tricyclic γ-lactone core [A] or monocyclic γ-lactone core [B] in selected natural and/or bioactive molecules; (b) catalytic asymmetric transformations of 2-siloxyfurans; (c) this work: diastereodivergent Cα-selective Mukaiyama-type asymmetric ring-opening (ARO)/lactonization of 2-siloxyfurans.

Stereodivergent synthesis of multi-stereogenic chiral compounds is of significant importance, as different absolute or relative configurations often dictate distinct physiological and pharmacological profiles.^1–3^ Multi-stereogenic γ-lactones are widely found in natural products, pharmaceuticals, flavors, and fragrances.^4–8^ Thus, the development of catalytic asymmetric methods for constructing these structures, especially in a stereodivergent manner, is of great interest. Although many elegant methods have been developed for the synthesis of enantioenriched γ-butyrolactones with one or two stereocenters on the γ-butyrolactone ring,^9–18^ there are few catalytic asymmetric protocols for the diastereo- and enantioselective synthesis of γ-butyrolactones with three stereocenters. The tricyclic γ-lactone core [A], shown in Fig. 1a, represents a privileged subclass of γ-lactone architectures.^19–24^ Nevertheless, there is only one catalytic enantioselective approach available—based on asymmetric transfer hydrogenation followed by *syn*-selective lactonization—to deliver a single diastereomer of such tricyclic γ-lactones.^16–18^ To date, there has been no approach involving catalytic asymmetric carbon–carbon (C–C) bond formation^25^ that would provide a more direct and efficient route to these chiral tricyclic γ-lactones. Moreover, despite remarkable progress in the area of stereodivergent synthesis,^26–30^ a diastereodivergent synthesis of these architectures has not been achieved. Thus, it is highly desirable to establish a modular and stereodivergent catalytic strategy to access structural diversified chiral tricyclic γ-lactone scaffolds based on C–C bond formation from simple building blocks.

2-Siloxyfurans, as unstabilized dienolate nucleophiles, are a class of versatile platforms in organic synthesis.^31,32^ In asymmetric catalysis, the Cγ-selective vinylogous Mukaiyama-type reactions of 2-siloxyfurans with various electrophiles have been widely exploited to afford chiral γ,γ-disubstituted butenolides, due to the existence of a thermodynamically more stable conjugated π-system in the resulting products (Fig. 1b).^33–40^ In contrast, catalytic asymmetric Cα-selective Mukaiyama-type reactions have lagged behind and remain a significant challenge.^41–43^ In 2012, Feringa and Hartwig independently reported the asymmetric Cα-selective allylation reactions of 2-siloxyfurans using Pd and Ir catalysis, respectively.^41,42^ Both studies indicate that 2-siloxyfurans are activated by the carboxylate leaving group of the allylic electrophiles to generate dienolate anions I. In Feringa's Pd catalyst system, hydrogen-bonding interaction between the oxygen anion and the chiral ligand was proposed to play a key role in controlling regioselectivity.^41^ Despite this insight, both reactions still failed to control the stereochemistry at the 2-siloxyfuran nucleophiles, resulting in either a 1 : 1 diastereomeric ratio (dr) or producing conjugated γ-butenolides *via* double bond isomerization. Meanwhile, Mlynarski and co-workers described a chiral Zn(ii)-catalyzed asymmetric Mukaiyama aldol reaction to produce conjugated γ-butenolides, in which water-containing solvents played a crucial role in directing Cα-regioselectivity.^43^ However, the enantioselectivity remained modest, with only up to 70% ee. Currently, there is still no general catalytic protocol that can achieve both high enantio- and diastereo-selectivity in the Cα-selective reaction of 2-siloxyfurans, and a diastereodivergent version also remains an elusive goal, due to multiple challenges in controlling regioselectivity and enantioselectivity as well as tuning diastereoselectivity. These challenges have severely limited the potential applications of the Cα-selective reaction of 2-siloxyfurans.

Herein we describe the first successful Cα-regioselective, enantioselective and diastereodivergent reaction of 2-siloxyfurans through the development of new catalytic systems (Fig. 1c). The reaction of unstabilized 2-siloxyfuran nucleophiles with oxabicyclic alkenes^44–46^ as electrophilic partners proceeded with high regio-, enantio- and diastereoselectivity *via* a cascade annulation, providing a diastereodivergent synthesis of chiral tricyclic lactone frameworks with three stereocenters in a single step. Different from the traditional diastereodivergent C–C bond formation strategy,^47–51^ this stereodivergent approach involves common stereoselective C–C bond formation, followed by divergent lactonization processes (C–O bond formation) directed by a co-catalyst. By tuning the co-catalyst, a rare diastereoselective reversal occurs, selectively yielding the thermodynamically less stable diastereomer. In contrast with traditional stoichiometric thermodynamic-driven epimerization,^13,52^ this stereoinversion involves a catalytic dynamic kinetic lactonization-driven epimerization process. Furthermore, the observed Cα-regioselectivity is attributed to the distinctive dispersion and electrostatic interactions between 2-siloxyfurans and the electrophile/catalyst. The resulting chiral tricyclic γ-lactones as new versatile platforms for diversity-oriented synthesis^53,54^ have been demonstrated by the diastereodivergent synthesis of multi-stereogenic monocyclic γ-lactones and diverse asymmetric syntheses of chiral quaternary carbon-embedded tricyclic γ-lactones as well as other important chiral frameworks, such as dihydropyridazin-3(2*H*)-ones and dioxabicyclo[3.2.1]octanes,^55*a*^ thus significantly expanding the chemical space of stereochemical diversity, skeletal diversity, and functional-group diversity.

## Results and discussion

### Reaction development

We initiated our studies by performing the reaction of oxabenzonorbornadiene 1a with 2-siloxyfuran 2a (TIPSOF) at 45 °C in the presence of Rh(COD)_2_OTf and Mandyphos L2 as the chiral catalyst (Table 1). The reaction led to the formation of phenol *via* the decomposition of oxabenzonorbornadiene 1a (entry 1). Interestingly, the reaction did not proceed when ZnF_2_ was used to activate 2-siloxyfuran 2a to generate the corresponding dienolate anion I (entry 2).^41,42^ After extensive efforts,^55*b*^ the desired chiral tricyclic γ-lactone product 3a with an *R*,*R*,*R*-configuration^56*a*–*e*^ was finally obtained in 71% yield with >20 : 1 Cα/Cγ selectivity, >20 : 1 dr and 92% ee (entry 3), when Zn(OTf)_2_ was used as a co-catalyst.^57^ Reducing the loading of Zn(OTf)_2_ to 20 mol% led to a sharp decrease in diastereoselectivity, indicating that Zn(OTf)_2_, acting as a Lewis acid, was involved in the key stereodetermining transition state, either by activating the bridgehead oxygen to facilitate the Rh oxidative insertion^46,57^ or by activating the nucleophile (entry 4). When Zn(OTf)_2_ was combined with ZnF_2_—an additive that promotes the desilylation of 2-siloxyfuran to dienolate anion I—the yield and diastereoselectivity significantly decreased (entry 5). This indicates that dienolate anion I is not an effective intermediate in this reaction. The silyl groups of 2-siloxyfurans affected both reactivity and selectivity. Smaller silyl groups, such as triethylsilyl (TES, 2b) and trimethylsilyl (TMS, 2c), resulted in decomposition of both substrates (entry 6), whereas the bulkier TBDMS group (2d) led to a poor diastereomeric ratio of 2 : 1, along with a small amount of 5a (entry 7).^56*f*^ Both the anion and cation of the metal Lewis acid salts influenced reactivity and selectivity. For example, Zn(OAc)_2_ was unreactive (entry 8). Intriguingly, KOTf as a co-catalyst afforded the major diastereomeric tricyclic γ-lactone 4a with an *S*,*R*,*R*-configuration, albeit with only moderate yield and enantioselectivity (entry 9).

**Table 1:** Selected optimization of the reaction conditions^a^

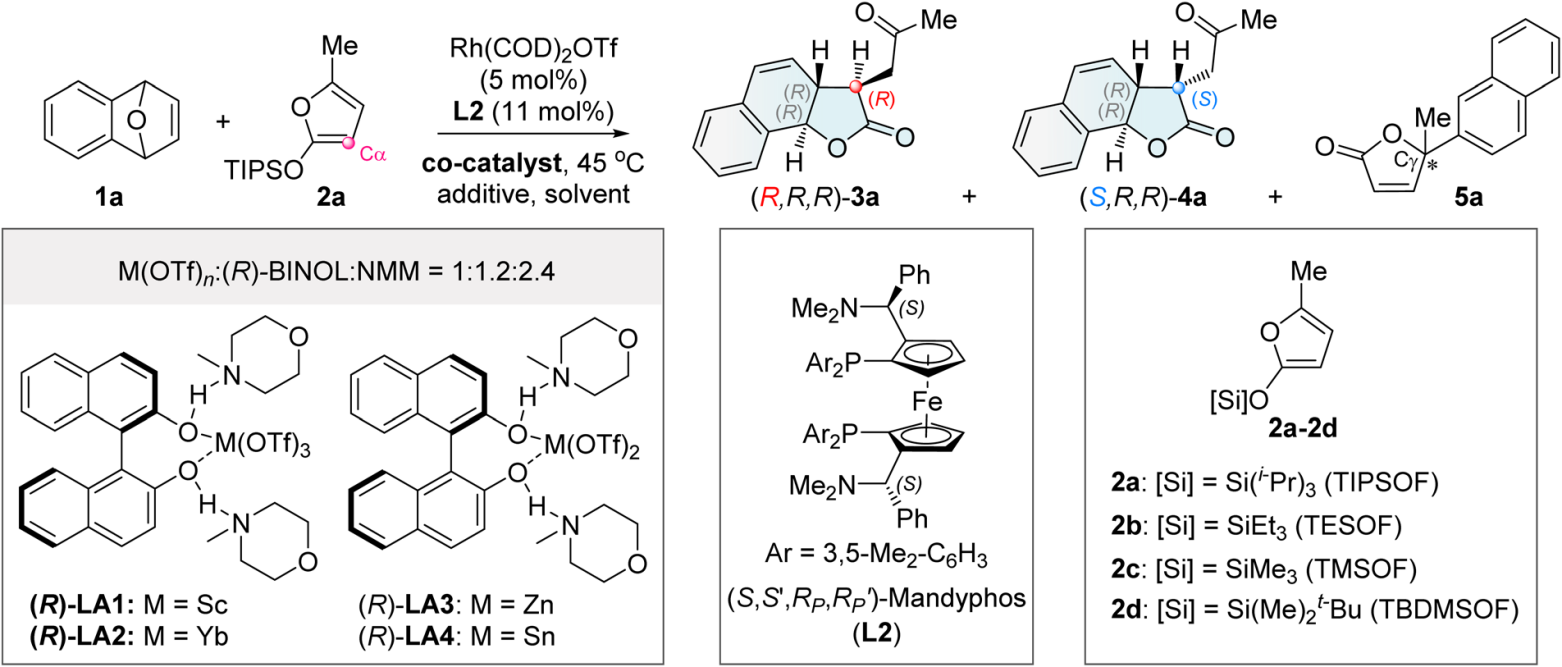						
Entry	Co-catalyst	Solvent, additive	Yield (%)	dr (3a/4a)	ee (%) (3a/4a)	rr (Cα/Cγ)
1	None	DCE/CHCl_3_, w/o	ND	—	—	—
2	ZnF_2_	DCE/CHCl_3_, w/o	NR	—	—	—
**3**	**Zn(OTf)**_**2**_	**DCE/CHCl**_**3**_ **, w/o**	**71**	**>20 : 1**	**92/—**	**>20 : 1**
4^b^	Zn(OTf)_2_	DCE/CHCl_3_, w/o	52	3.7 : 1	92/91	>20 : 1
5	Zn(OTf)_2_	DCE/CHCl_3_, ZnF_2_	39	4.6 : 1	87/98	>20 : 1
6^c^	Zn(OTf)_2_	DCE/CHCl_3_, w/o	ND	—	—	—
7^d^	Zn(OTf)_2_	DCE/CHCl_3_, w/o	65	2 : 1	80/91	11 : 1
8	Zn(OAc)_2_	DCE/CHCl_3_, w/o	ND	—	—	—
9	KOTf	DCE/CHCl_3_, w/o	37	1 : 13	76/59	>20 : 1
10^b^	(*R*)-LA1	DCE/CHCl_3_, w/o	77	1 : 11	79/97	9 : 1
11^b^	(*R*)-LA2	DCE/CHCl_3_, w/o	34	1 : 4.6	92/93	>20 : 1
12^b^	(*R*)-LA3	DCE/CHCl_3_, w/o	50	1 : 2	96/97	>20 : 1
13^b^	(*R*)-LA4	DCE/CHCl_3_, w/o	45	<1 : 20	—/93	>20 : 1
**14^b^**	**(*R*)-LA4**	**CHCl**_**3**_ **, TFE**	**76**	**<1 : 20**	**—/95**	**>20 : 1**
15^b^	(±)-LA4	CHCl_3_, TFE	71	<1 : 20	—/95	>20 : 1
16^b^	w/o (*R*)-BINOL in (*R*)-LA4	CHCl_3_, TFE	73	1 : 9	—/94	>20 : 1
17^b^	w/o NMM in (*R*)-LA4	CHCl_3_, TFE	61	1 : 1.4	97/93	>20 : 1
18^b^	w/o Sn(OTf)_2_ in (*R*)-LA4	CHCl_3_, TFE	NR	—	—	—

a Unless otherwise noted, the reaction was conducted with 1a (0.1 mmol), 2a (0.5 mmol), Rh(COD)_2_OTf (5 mol%), L2 (11 mol%), co-catalyst (50 mol%), and additive (2.0 equiv. ZnF_2_ or 3.5 equiv. TFE), in indicated solvent (2 mL, 1 : 1 mixed solvents) under an argon atmosphere at 45 °C. The yield refers to the combined yield of 3a and 4a. The dr value was determined by ^1^H-NMR analysis for the ratio of 3a and 4a. The ee value of 3a/4a was determined by chiral HPLC analysis. The regioselective ratio (rr) was determined by ^1^H-NMR spectroscopy for the ratio of (3a + 4a) and 5a.

b Co-catalyst (20 mol%) was used.

c Using 2b or 2c (0.5 mmol) in place of 2a.

d Using 2d (0.5 mmol) in place of 2a. NR = no reaction. ND = not detected. w/o = without.

Inspired by the potential capability of Kobayashi's combined chiral Lewis acid catalysts based on rare-earth metals, such as (*R*)-LA1 and (*R*)-LA2, in asymmetric reactions,^58,59^ we evaluated several related systems to improve the inversion of diastereoselectivity toward (*S*,*R*,*R*)-4a. The combined Lewis acids (*R*)-LA1 (Sc(OTf)_3_-(*R*)-BINOL-NMM) and (*R*)-LA2 (Yb(OTf)_3_-(*R*)-BINOL-NMM) as co-catalysts successfully switched the diastereoselectivity toward (*S*,*R*,*R*)-4a as the major diastereomer (entries 10 and 11). Encouraged by this result, other combined chiral Lewis acid co-catalysts were examined (entries 12 and 13 and Table S6 in the SI). Among them, (*R*)-LA4 (Sn(OTf)_2_-(*R*)-BINOL-NMM)—a previously unreported catalyst based on main-group metals—provided (*S*,*R*,*R*)-4a with excellent regio-, diastereo-, and enantio-selectivity, albeit in 45% yield (entry 13). The yield was significantly improved to 76% by adding the proton source CF_3_CH_2_OH (TFE) in CHCl_3_, affording the desired product (*S*,*R*,*R*)-4a with >20 : 1 Cα/Cγ selectivity, >20 : 1 dr, and 95% ee (entry 14: conditions B).^56*a*–*e*^ In contrast to Kobayashi's work where the chirality of BINOL in combined Lewis acids controlled enantioselectivity, the chirality of BINOL in LA4 had no effect on stereoselectivity. Even racemic LA4 (Sn(OTf)_2_-(±)-BINOL-NMM) afforded excellent results (entry 15 and Table S8 in the SI). The absence of BINOL resulted in the major diastereoisomer 4a with diminished diastereoselectivity (entry 16). No NMM led to a low dr, delivering the mixed products 3a and 4a (entry 17). Without Sn(OTf)_2_, the reaction did not work (entry 18). This study not only provides an unprecedented method for diastereodivergent asymmetric synthesis but also expands the chemistry of combined Lewis acid catalysis by demonstrating its unique ability to switch diastereocontrol, opening a new avenue in asymmetric synthesis.^60^

### Mechanistic study

**Scheme 1:**
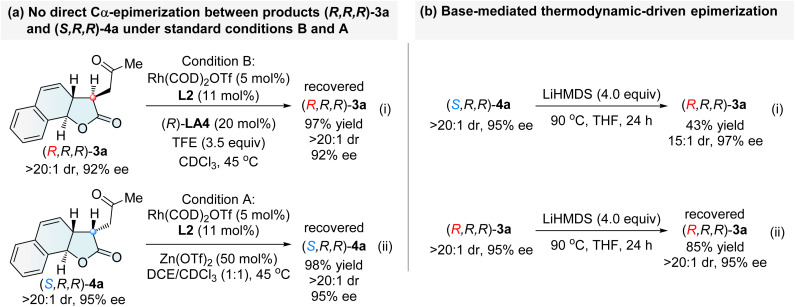
Interconversion experiments of 3a and 4a.

To gain insight into the reaction mechanism underlying the diastereodivergent formation of tricyclic γ-lactones, a series of mechanistic experiments were conducted. First, no direct Cα-epimerization between (*R*,*R*,*R*)-3a and (*S*,*R*,*R*)-4a was observed under standard conditions B and A, respectively (Scheme 1a(i and ii)). Interestingly, upon treatment with lithium hexamethyldisilazide (LiHMDS, 4.0 equiv.) at an elevated temperature (90 °C), (*S*,*R*,*R*)-4a was converted into (*R*,*R*,*R*)-3a in 43% yield with 15 : 1 dr, accompanied by a significant decomposition of 4a (Scheme 1b(i)). In contrast, (*R*,*R*,*R*)-3a could not be converted into (*S*,*R*,*R*)-4a (Scheme 1b(ii)).^55*b*^ These results suggest that the chiral tricyclic γ-lactone (*S*,*R*,*R*)-4a is a thermodynamically less stable isomer, while the thermodynamically stable diastereomer is (*R*,*R*,*R*)-3a.

**Fig. 2:**
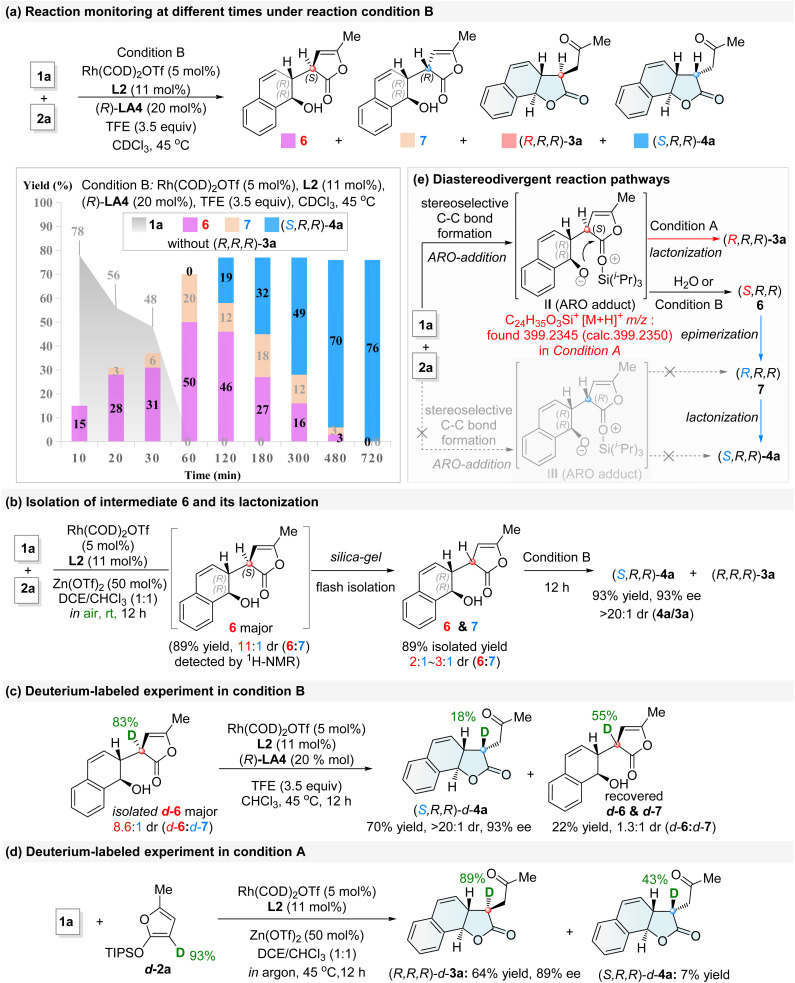
Control experiments. (a) Reaction monitoring at different times under reaction conditions B; (b) isolation of intermediate 6 and its lactonization; (c) deuterium-labeled experiment under conditions B; (d) deuterium-labeled experiment under conditions A; (e) diastereodivergent reaction pathways.

Next, the reaction evolution for the formation of product (*S*,*R*,*R*)-4a was monitored in deuterated solvent under standard conditions B at various time points. As shown in Fig. 2a, intermediate 6 initially appeared—then its diastereomeric counterpart 7—and subsequently the product (*S*,*R*,*R*)-4a. Notably, intermediate 6 as the major diastereoisomer can also be obtained under modified conditions A in air at room temperature with the catalysis of [Rh]-L2/Zn(OTf)_2_ (Fig. 2b: 11 : 1 dr, 89% yield by ^1^H-NMR analysis). However, silica-gel chromatographic separation afforded a mixture of intermediates 6 and 7 with a 2 : 1 to 3 : 1 dr in 89% yield, indicating that intermediate 6 is readily prone to Cα-racemization. Under conditions B, the isolated mixture of 6 and 7 (2 : 1 to 3 : 1 dr) led to the product (*S*,*R*,*R*)-4a in 93% yield with >20 : 1 dr. These results indicate that, under conditions B, intermediate 6 with an (*S*,*R*,*R*)-configuration is unlikely to undergo the direct lactonization to form the product (*R*,*R*,*R*)-3a. A significant loss of deuterium content was observed in both 4a and the recovered intermediate mixture 6/7, indicating that during Cα-epimerization from intermediate 6 to 7 and the subsequent lactonization to product (*S*,*R*,*R*)-4a (Fig. 2c), H/D exchange occurs *via* a deprotonation–reprotonation step.

Under conditions A, a high deuterium retention ratio of 96% was observed in (*R*,*R*,*R*)-*d*-3a, based on *d*-2a (93% D) (Fig. 2d).^55*b*^ High-resolution mass spectrometry (HRMS) further identified the reactive ARO adduct II—not intermediate 6—as the key species leading to (*R*,*R*,*R*)-3a.

Based on the above experimental findings, it was proposed that both dual catalytic systems proceed through a common stereocontrolled transition state for C–C bond formation, followed by divergent lactonization pathways (Fig. 2e). Crucially, the diastereoselective inversion is governed by the synergistic action of both the combined multifunctional co-catalyst LA4 and the proton additive TFE.^55*b*^ This system promotes the protonation of the ARO-adduct II (to (*S*,*R*,*R*)-6)—thereby suppressing the formation of (*R*,*R*,*R*)-3a—while concurrently enabling a dynamic kinetic lactonization-driven epimerization pathway of (*S*,*R*,*R*)-6 to (*R*,*R*,*R*)-7 that selectively yields the thermodynamically less stable isomer (*S*,*R*,*R*)-4a. This proposed mechanism was supported by the density functional theory (DFT) calculations elucidating the observed diastereoselective reversal (Fig. S6–S8 in SI). DFT results confirm that the combined catalyst (*R*)-LA4 acts as a multifunctional catalytic system, integrating the capabilities of both a Lewis acid and a proton-transfer catalyst across the process. Computational data indicate that these two diastereomers 6 and 7 are thermodynamically similar, which is consistent with the experimental observation that the epimerization from 6 to 7 occurs relatively easily while maintaining a consistently low dr. Conversely, the pathway from 6 to (*R*,*R*,*R*)-3a was calculated to be energetically disfavored.

**Fig. 3:**
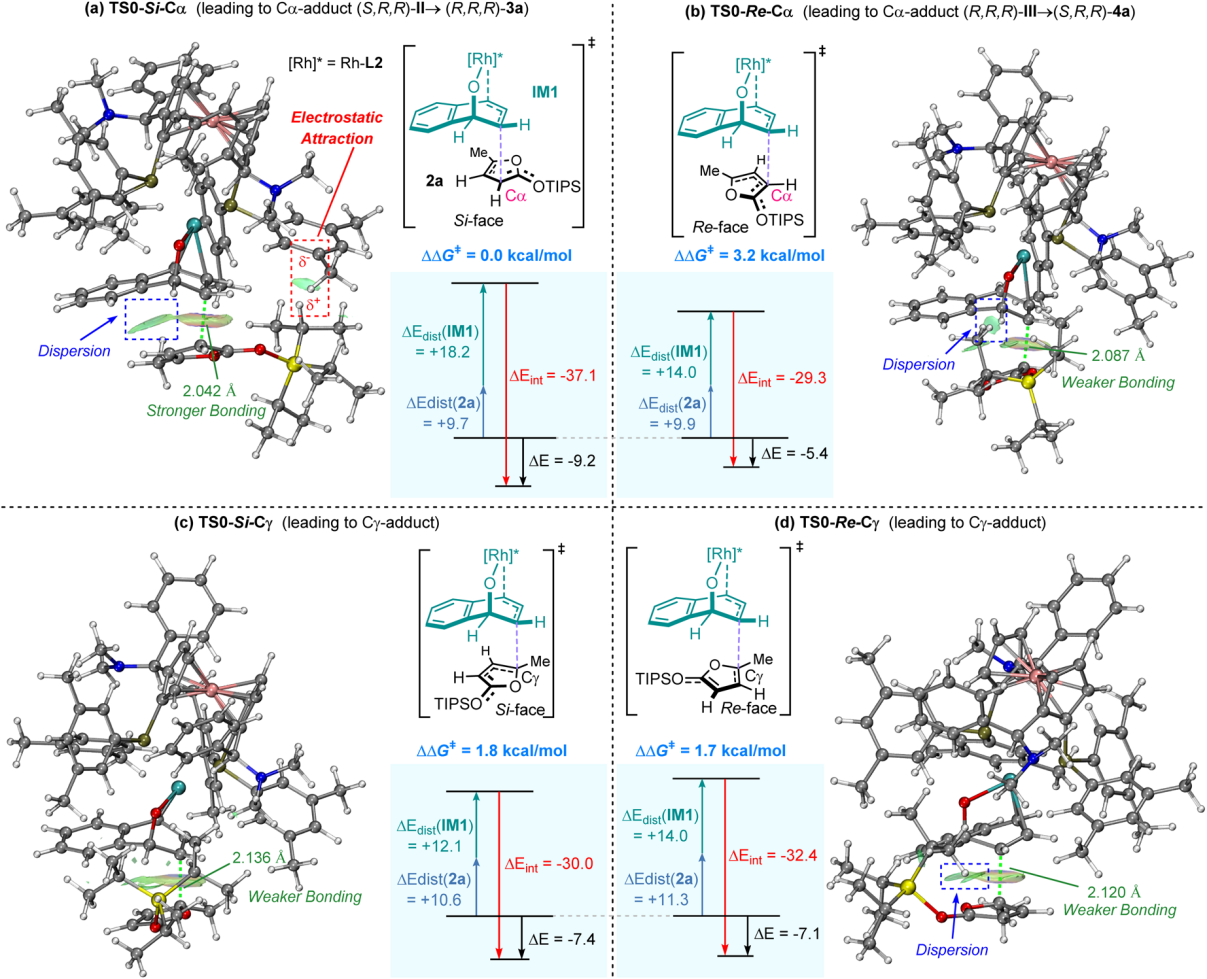
IGMH & distortion/interaction (DI) analysis for regio-determining and stereo-determining transition states in the C–C bond formation step. (a) TS0-*Si*-Cα (leading to Cα-adduct (*S*,*R*,*R*)-II → (*R*,*R*,*R*)-3a); (b) TS0-*Re*-Cα (leading to Cα-adduct (*R*,*R*,*R*)-III → (*S*,*R*,*R*)-4a); (c) TS0-*Si*-Cγ (leading to Cγ-adduct); (d) TS0-*Re*-Cγ (leading to Cγ-adduct).

According to the results above, DFT calculations were performed to elucidate both regioselectivity and stereoselectivity in the C–C bond formation step (Fig. 3, S4 and S5 in the SI). The computational results were consistent with the experimental observations regarding the regioselectivity and stereoselectivity. Distortion/interaction analysis and the independent gradient model based on Hirshfeld partition (IGMH)^61,62^ revealed that non-covalent interactions between π-allylic [Rh]* species (IM1) and TIPSOF 2a dominate the energy trends among different transition states. In the most favored transition state TS0-*Si*-Cα, significant dispersion forces and electrostatic attraction were observed between the furan ring of 2a and the π-allylic [Rh]* species (IM1), contributing to the stabilization of the transition state. In contrast, the three disfavored transition states lacked electrostatic attraction and exhibited diminished dispersion due to steric hindrance from silyl groups, as evidenced by elongated C–C bond lengths in these transition states.

**Fig. 4:**
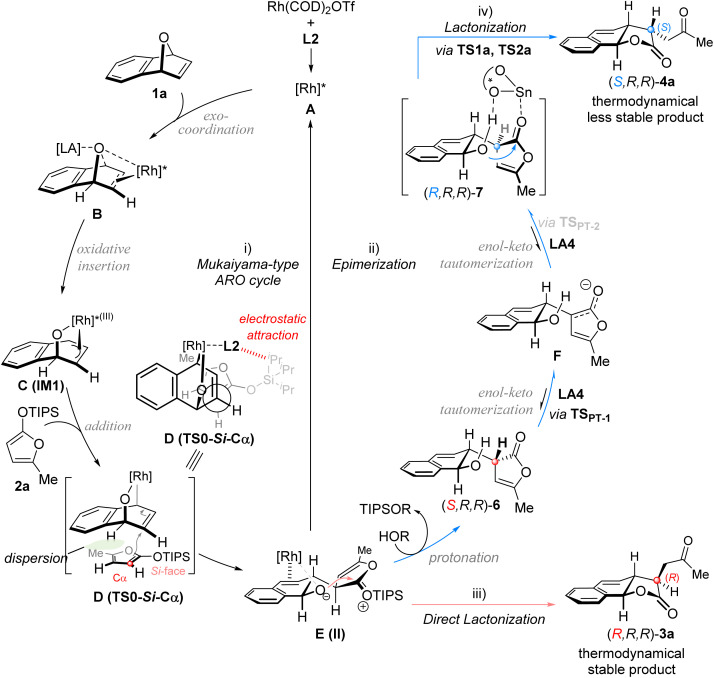
Possible reaction mechanism.

Based on the above results and related studies,^44,45,50,57^ a rational mechanism is proposed in Fig. 4. The chiral rhodium catalyst ([Rh]*) initially coordinates with oxabenzonorbornadiene 1a, followed by desymmetric oxidative insertion to generate the π-allylic [Rh]* species C (or IM1). Subsequently, a Cα-selective nucleophilic attack of 2a on C proceeds *via* the favored transition state D (or TS0-*Si*-Cα), yielding the ARO adduct E. This adduct undergoes direct lactonization to furnish the tricyclic γ-lactone (*R*,*R*,*R*)-3a while regenerating the Rh catalyst. In the presence of a proton source ((*R*)-LA4 or TFE), adduct E is rapidly protonated to form intermediate 6, which undergoes Cα-epimerization toward intermediate 7, followed by lactonization of 7 to deliver the tricyclic γ-lactone (*S*,*R*,*R*)-4a.

### Substrate scope

**Scheme 2:**
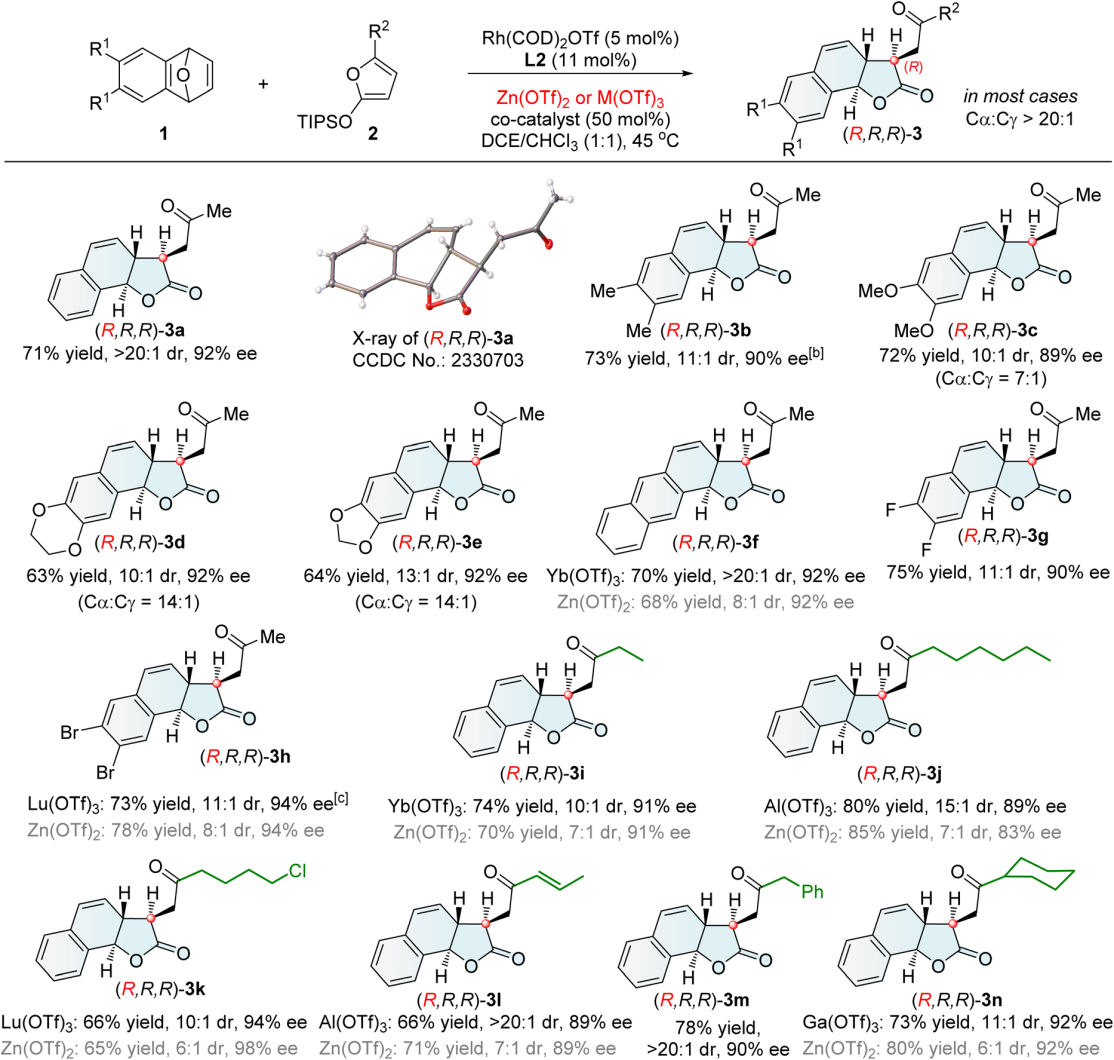
Asymmetric synthesis of tricyclic γ-lactones (*R*,*R*,*R*)-3^[a]^. [a] Unless otherwise noted, the reaction was conducted under the conditions described in entry 3 of Table 1, and the dr value refers to the ratio of 3 and 4. [b] L2 (15 mol%) was used. [c] At 50 °C.

With the optimized chiral catalytic systems and reaction conditions in hand, we investigated the generality of Cα-ARO/lactonization reactions. In the protocol catalyzed by Rh(i)/L2 and Zn(OTf)_2_, the scope of oxabenzonorbornadienes 1 was firstly examined (Scheme 2). A range of oxabenzonorbornadienes 1 bearing electron-rich, electron-neutral and electron-deficient groups on the benzene ring performed well in this reaction, affording tricyclic γ-lactones 3a–h. The naphthalene-containing substrate 1f smoothly participated in the reaction, and Yb(OTf)_3_ as a co-catalyst provided the desired tricyclic γ-lactone 3f in 70% yield with >20 : 1 dr, 92% ee, and >20 : 1 Cα/Cγ selectivity. Replacing Zn(OTf)_2_ with Lu(OTf)_3_ as the co-catalyst improved the diastereoselectivity for 3h from 8 : 1 to 11 : 1 dr while maintaining good enantioselectivity. Subsequently, the scope of 2-siloxyfurans 2 was investigated. Various 2-siloxyfurans bearing different substituents performed well in this process, producing the corresponding tricyclic γ-lactones (3a and 3i–n). Functional groups such as halides and olefins were well tolerated (3k and 3l). Notably, the 2-siloxyfuran with R = allyl led to tricyclic γ-lactone 3l featuring a conjugated enone moiety suitable for further transformation, resulting from olefin isomerization of the Cα-ring opening/lactonization product.

**Scheme 3:**
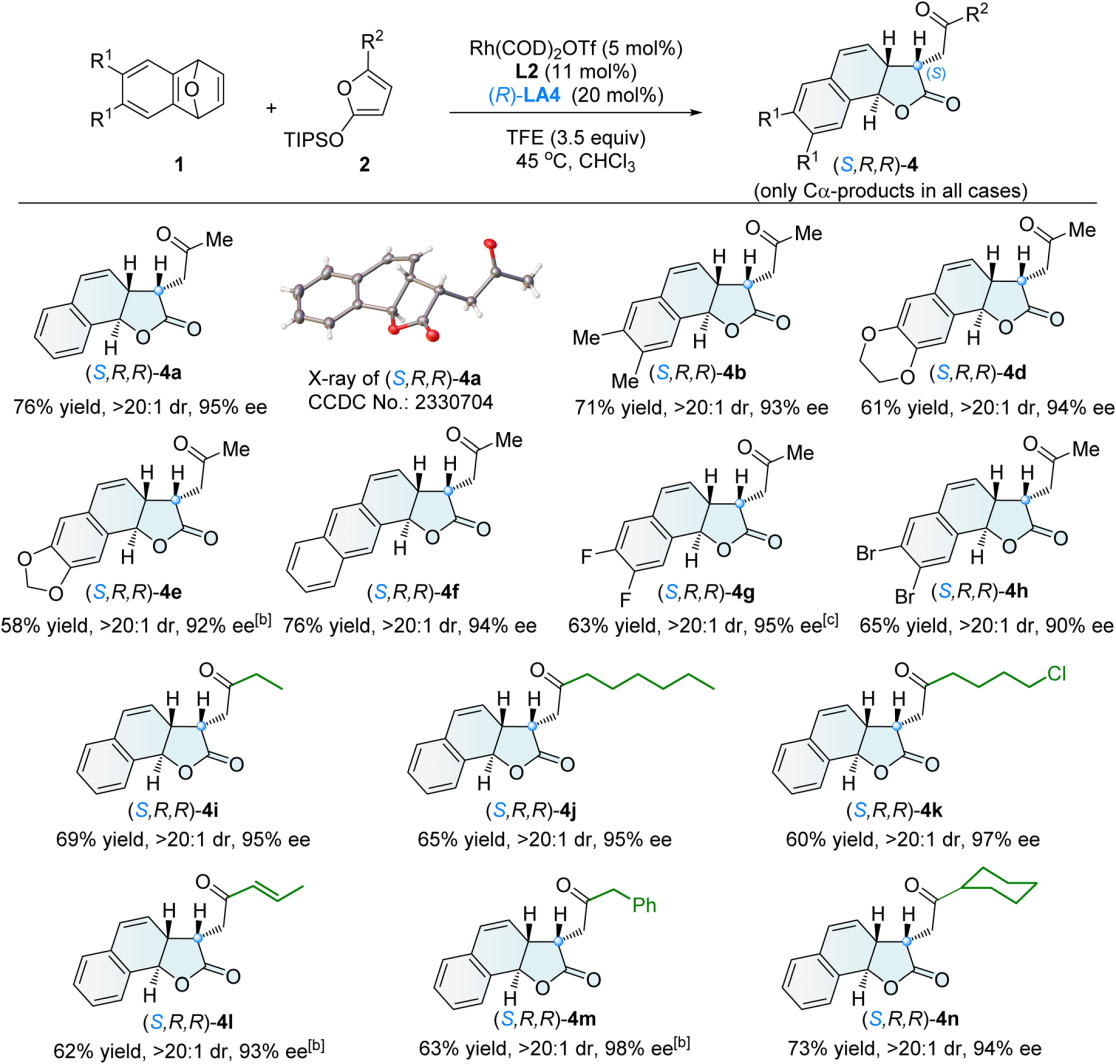
Asymmetric synthesis of tricyclic γ-lactones (*S*,*R*,*R*)-4^[a]^. [a] Unless otherwise noted, the reaction was conducted under the conditions described in entry 14 of Table 1, and the dr value refers to the ratio of 4 and 3. [b] 2 (3.0 equiv.) was used, at 40 °C. [c] At 40 °C.

The diastereodivergent Cα-ARO/lactonization reaction, catalyzed by Rh(i)/L2 in combination with the multifunctional co-catalyst (*R*)-LA4, demonstrated a broad substrate scope. A variety of tricyclic γ-lactones (*S*,*R*,*R*)-4 were obtained in generally good yields, exhibiting excellent diastereoselectivity (>20 : 1 dr), exclusive Cα-selectivity, and high enantioselectivity (90–98% ee) (Scheme 3).

### Stereodivergent synthesis and synthetic applications

**Scheme 4:**
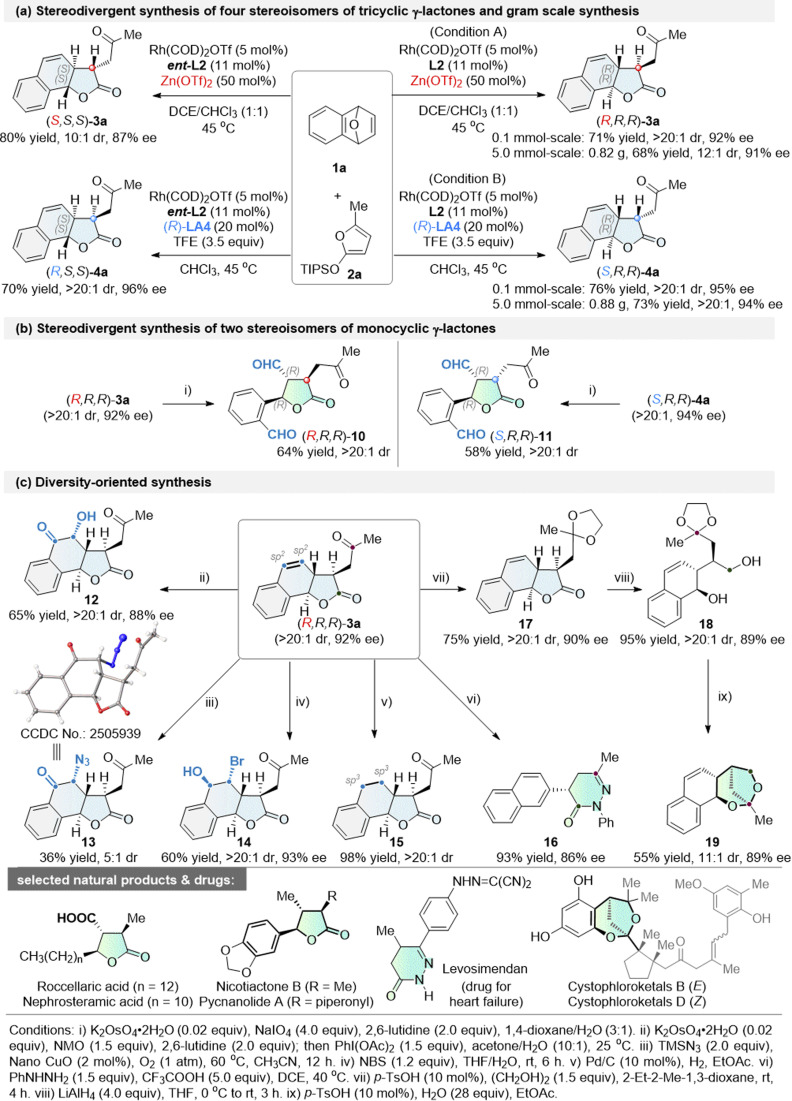
Stereodivergent synthesis, gram scale synthesis and diversity-oriented synthesis.

As indicated in Scheme 4a, changing the chiral ligand L2 to *ent*-L2 in the Cα-ARO/cyclization cascade reaction between 1a and 2a yielded (*S*,*S*,*S*)-3a in 80% yield with a 10 : 1 dr and 87% ee. Similarly, (*R*,*S*,*S*)-4a was obtained in 70% yield, with a >20 : 1 dr and 96% ee.^56*a*–*e*^ Thus, four stereoisomers were synthesized in good yields, exhibiting both excellent enantioselectivity and diastereoselectivity. Moreover, the tricyclic γ-lactones (*R*,*R*,*R*)-3a and (*S*,*R*,*R*)-4a were prepared on a gram scale with comparable reactivity and selectivity (Scheme 4a). Although a slight decrease in diastereoselectivity was observed for (*R*,*R*,*R*)-3a, it still maintained a high dr of 12 : 1, demonstrating the robustness of this diastereodivergent protocol.

**Scheme 5:**
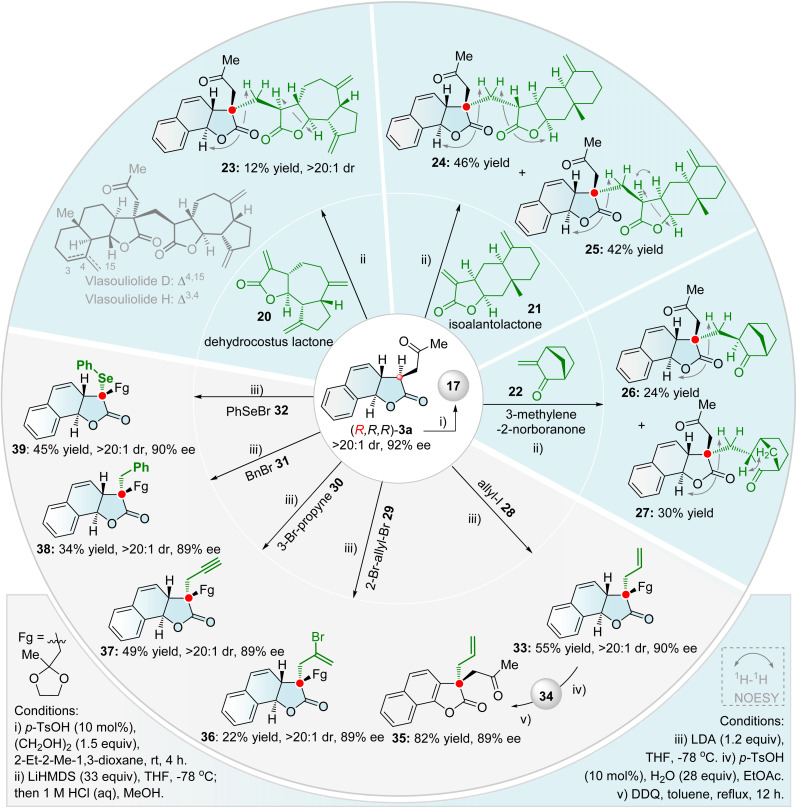
Diverse Cα-quaternizations of tricyclic γ-lactone 3a.

Diversity-oriented synthesis (DOS), a powerful tool for drug discovery, aims to efficiently construct compound libraries with structural complexity, stereochemical, and functional diversity.^53,54^ By leveraging the multiple functional groups embedded in the resulting tricyclic γ-lactone products, we successfully executed a series of intriguing DOS protocols starting from (*R*,*R*,*R*)-3a (Schemes 4b, c and 5).

The alkene moiety in 3a serves as a versatile linchpin for downstream diversification, enabling rapid expansion of three-dimensional chemical space and modular elaboration of skeletal and functional complexity. Oxidative cleavage of the olefin under catalytic K_2_OsO_4_/NaIO_4_ conditions—avoiding the use of volatile, toxic OsO_4_—yields γ-aryl-substituted monocyclic γ-lactone (*R*,*R*,*R*)-10 in 64% yield with >20 : 1 dr (Scheme 4b). The same cleavage protocol applied to (*S*,*R*,*R*)-4a furnishes the complementary diastereomer (*S*,*R*,*R*)-11 in 58% yield with >20 : 1 dr. Thus, these transformations provide a diastereodivergent gateway to valuable monocyclic γ-lactones bearing three contiguous stereocenters.

Interestingly, switching the co-oxidant manifold to NMO/PhI(OAc)_2_ while retaining catalytic K_2_OsO_4_ redirected the reaction toward ketohydroxylation, delivering α-hydroxy ketone 12 (Scheme 4c: 65% yield, >20 : 1 dr, 88% ee). Notably, a heterogeneous copper-based catalyst diversified the alkene functionalization manifold, facilitating oxyazidation to furnish α-azido ketones 13 in 36% yield (5 : 1 dr), instead of the previously reported C=C double-bond cleavage.^63^ The structure and absolute configuration of 13 were confirmed by single-crystal X-ray analysis. Exposure to NBS in THF/H_2_O promoted hydroxyl-bromination, ultimately yielding the densely functionalized product 14 (60% yield, >20 : 1 dr, 93% ee). Pd/C-mediated hydrogenation of (*R*,*R*,*R*)-3a cleanly saturated the double bond to provide 15 in 98% yield, enhancing the sp^3^ character of this scaffold.

Treatment with PhNHNH_2_ led to a cascade amidation/condensation cyclization of 3a, affording the privileged dihydropyridazin-3(2*H*)-one 16 with 86% ee and 93% yield. Due to the poor diastereoselectivity (1 : 1 dr) in the direct LiAlH_4_-mediated reduction of 3a, an alternative cascade reduction protocol was developed. This protocol commenced with the protected ketal 17, followed by the reduction of lactone to give the diol 18. Finally, hydrolysis-initiated cyclization of 18 delivered the complex fused 2,7-dioxabicyclo[3.2.1]octane 19, thereby enabling skeletal diversity synthesis. Ring-distortion reactions represent a hallmark strategy in diversity-oriented synthesis, involving structural reshaping of existing ring systems through predictable chemical operations—such as ring cleavage, rearrangement, aromatization, and fusion—to rapidly access diverse structural and skeletal molecular platforms. With this strategy in mind, we have successfully transformed the tricyclic γ-lactone 3a into privileged chiral scaffolds including monocyclic γ-lactones,^4,5,64^ dihydropyridazin-3(2*H*)-ones,^65^ and fused dioxabicyclo[3.2.1]-octanes^66^—structural motifs that are widely found in natural products and pharmaceutical agents.^55*a*^

The efficient construction of chiral quaternary stereocenters represents a fundamental yet challenging objective in modern organic synthesis, due to their three-dimensional structural rigidity and unique biological activities.^67,68^ By employing two kinds of efficient enantioselective sequential processes, diverse chiral quaternary carbon stereocenter-embedded tricyclic γ-lactones were synthesized (Scheme 5). These transformations circumvent the reliance on natural chiral sources typical of traditional synthetic methods and achieve precise control over complex three-dimensional architectures and functional group diversity starting from simple feedstocks. To avoid competitive regioselectivity issues, the ketal-protected tricyclic γ-lactone 17 was used as the starting substrate. First, the LiHMDS-mediated Michael protocol efficiently introduces the natural scaffolds (20–22) onto tricyclic γ-lactones, delivering variants bearing chiral quaternary stereocenters (23–27). Coupling with dehydrocostus lactone 20, followed by acid-promoted deprotection, rapidly furnished the single diastereoisomer 23—an analogue of the natural bis-sesquiterpene lactones Vlasouliolide D and H^69^—with excellent diastereoselectivity (>20 : 1 dr). As we know, this is the first enantioselective synthesis of the bis-sesquiterpene lactone core. When isoalantolactone 21 was employed as a Michael acceptor in a similar sequential procedure, column chromatographic isolation afforded two stereoisomeric products 24 (46% yield) and 25 (42% yield). Reaction with 3-methylene-2-norbornanone 22 similarly afforded the stereoisomers 26 (24% yield) and 27 (30% yield). ^1^H–^1^H NOESY correlations confirmed the absolute configuration of all these stereoisomeric products, revealing uniform stereochemistry at the newly formed quaternary carbon center. Next, the direct LDA-mediated S_*N*_-type Cα-alkylation of γ-lactone with various alkyl halides 28–32 provided an alternative strategy to access chiral γ-lactones bearing quaternary stereocenters. Several synthetically useful functional groups—including allyl (33, 36), propargyl (37), and benzyl group (38)—were successfully incorporated into the lactone core, demonstrating the method's versatility. Employing PhSeBr under these conditions smoothly afforded the Cα-selenylated product 39 (45% yield, >20 : 1 dr, 90% ee). Additionally, DDQ-mediated oxidative aromatization of the dihydronaphthyl unit in deprotected 34 readily afforded chiral quaternary carbon-embedded naphthofuranones 35—a π-expanded homologue of the privileged benzofuranone scaffold.^55*a*^

## Conclusions

In summary, we have established the first Cα-selective, diastereodivergent asymmetric Mukaiyama-type ring-opening/lactonization of 2-siloxyfurans with oxabicyclic alkenes. A series of chiral tricyclic γ-lactones with three contiguous stereocenters were synthesized in excellent enantioselectivity (up to 98% ee), diastereoselectivity (up to >20 : 1 dr) and regioselectivity (up to >20 : 1 Cα : Cγ). By simply changing the co-catalyst (Zn(OTf)_2_ or Yb(OTf)_3_) to a novel multifunctional co-catalyst (Sn(OTf)_2_-BINOL-NMM), the diastereoselectivity was efficiently switched. Four stereoisomers with (*S*,*S*,*S*), (*R*,*R*,*R*), (*R*,*S*,*S*), and (*S*,*R*,*R*)-configurations were readily obtained. This protocol features several advances: (a) the first highly enantioselective and diastereoselective Cα-selective reaction of 2-siloxyfurans, as well as the first diastereodivergent asymmetric Cα-selective reaction of 2-siloxyfurans; (b) developing an alternative catalytic asymmetric route to privileged chiral tricyclic γ-lactones based on a stereoselective C–C bond formation strategy; (c) successfully accomplishing a transition-metal-catalyzed ARO reaction of oxabenzonorbornadienes with unstabilized enolates, exhibiting high stereocontrol of nucleophiles; (d) discovering a distinctive and rare diastereoselective reversal strategy—a catalytic dynamic kinetic lactonization-driven epimerization—that selectively delivers the thermodynamically less stable diastereomer. Moreover, DFT and experimental studies revealed that the origin of Cα-regioselectivity arises from the dispersion and electrostatic interactions between 2-siloxyfurans and the electrophilic partner/chiral catalyst, opening a new platform for reaction design. Additionally, these tricyclic γ-lactones serve as versatile platforms for diversity-oriented synthesis. This utility was demonstrated by the diastereodivergent synthesis of monocyclic γ-lactones with three contiguous stereocenters and the construction of several complex scaffolds, including chiral quaternary carbon-embedded tricyclic γ-lactones, dihydropyridazin-3(2*H*)-ones, and fused dioxabicyclo[3.2.1]octanes.

## Author contributions

Z. H. S. conceived and directed the project. Y. H. D. and Y. C. W. directed the project. Z. H. Z. and Z. Q. L. performed the DFT calculations. L. F. G. performed most of the chemistry experiments. X. C. W., J. T. R., M. J., P. L. C. and J. H. H. assisted in the separation and purification of some target products. J. Y. Z. assisted in the synthesis of some substrates. T. C., F. Z. P. and R. T. provided guidance for the project. All authors participated in the discussion. Z. H. S. and Y. H. D. prepared this manuscript.

## Conflicts of interest

All authors declare no competing interests.

## Supplementary Material

SC-OLF-D6SC01491G-s001

SC-OLF-D6SC01491G-s002

## Data Availability

CCDC 2330703 ((*R*,*R*,*R*)-3a), 2330704 ((*S*,*R*,*R*)-4a), 2333474 ((*R*,*S*,*S*)-4a), 2341397 (9) and 2505939 (13) contain the supplementary crystallographic data for this paper.^56*a*–*e*^ The authors declare that the data supporting the findings of this study are available within the article and the supplementary information (SI) as well as from the authors upon request. The coordinates of the optimized structure are available from the source data. Supplementary information: experimental procedures, experimental equipment, synthetic applications, characterization data, computational results, X-ray data, HRMS, HPLC and NMR spectra for all new compounds. See DOI: https://doi.org/10.1039/d6sc01491g.
