# Supplementary material for: Highly Cα-regio-, enantio- and diastereoselective Mukaiyama-type annulation of siloxyfurans: stereodivergent synthesis of multi-stereogenic tricyclic γ-lactones
Source: Chem Sci. 2026 Apr 29;17(23):11445–58. doi: 10.1039/d6sc01491g (PMC13147288; doi:10.1039/d6sc01491g)
Supplement: SC-017-D6SC01491G-s001 [file SC-017-D6SC01491G-s001.pdf]

Supplementary Information (SI) for Chemical Science.
This journal is © The Royal Society of Chemistry 2026

Supplementary Information

Highly Cα-Regio-, Enantio- and Diastereoselective Mukaiyama-type Annulation of Siloxyfurans: Stereodivergent Synthesis of Multi-stereogenic Tricyclic γ-Lactones

Lifei Gan,[‡a] Zi-Qing Li,[‡b] Tao Chen,[‡a] Xuanchen Wan,[a,d] Junyang Zhang,[a] Jiangtao Ren,[a] Ming Jiang,[a] Penglong Cao,[a] Jinhai Huang,[a] Yu-Hua Deng,[*a] Fangzhi Peng,[a] Run Tian,[c] Yingcheng Wang,[*a] Zhihan Zhang,[*b] and Zhihui Shao[*a,d]

[a] *Key Laboratory of Medicinal Chemistry for Natural Resource, Ministry of Education, School of Chemical Science and Technology, School of Pharmacy, and State Key Laboratory for Conservation and Utilization of Bio-Resources in Yunnan, Yunnan University; Kunming 650091, China*
[b] *College of Chemistry, Central China Normal University; Wuhan, 430079, China*
[c] *Yunnan University Affiliated Hospital, Yunnan University; Kunming, 650000, China*
[d] *Southwest United Graduate School; Kunming 650092, China*

E-mail: zhihui_shao@hotmail.com; zhihanzhang@ccnu.edu.cn.

Table of contents

1. General information S1
2. Competitive reaction pathways and optimization reaction condition S2
3. General procedures for catalytic asymmetric [3+2]-annulation S11
4. Mechanism studies S27
5. Gram-scale reaction S70
6. Synthetic transformations S72
7. Single-crystal x-ray diffraction date S93
8. References S100
9. NMR spectra S104
10. HPLC spectra S167

1. General information

Unless otherwise noted, all reactions were set up in a 10 mL Telfon-screw capped test tubes under the atmosphere of Argon (Ar). Solvents were purified under Ar using a solvent purification system.

Analytical thin layer chromatography (TLC) was performed using silica gel plates. Visualisation was detected by ultraviolet fluorescence, and/or phosphomolybdic acid, and/or $KMnO_4$.

Flash column chromatography (FC) was performed using *Qingdao* (200-300 mesh) silica gel.

NMR: ^{1}H NMR, ^{13}C NMR, ^{19}F NMR spectra were recorded on Bruker Avance 400 MHz, 500 MHz, 600 MHz Spectrometer. ^{1}H and ^{13}C chemical shifts were referenced internally to residual solvent peaks relative to TMS (δ = 0 ppm) at 299 K. Chemical shifts (δ (ppm)) are reported relative to TMS (δ (^{1}H) 0.0 ppm, δ (^{13}C) 0.0 ppm). The solvents' residual proton resonance and the respective carbon resonance (for $CHCl_3$; δ (^{1}H) 7.26 ppm, δ (^{13}C) 77.0 ppm) were used for calibration.

HPLC spectra were recorded on an *Agilent* HPLC. Column, eluent and retention times for HPLC analysis used for the determination of enantiomeric ratios are given below in the details of the relevant experiments.

Optical rotations were measured on a *JASCO* DIP-370 polarimeter.

High-resolution mass spectra (HRMS) was recorded on a VG Auto Spec-3000 spectrometer.

All reagents were either prepared acc1ording to known literatures or purchased from *Energy-chemical, TCI*, *Bide-pharmatech*, Laajoo, Leyan, Adamas-beta® and Adamas-life®.

2. Competitive reaction pathways and optimization reaction condition

2.1 Competitive reaction pathways

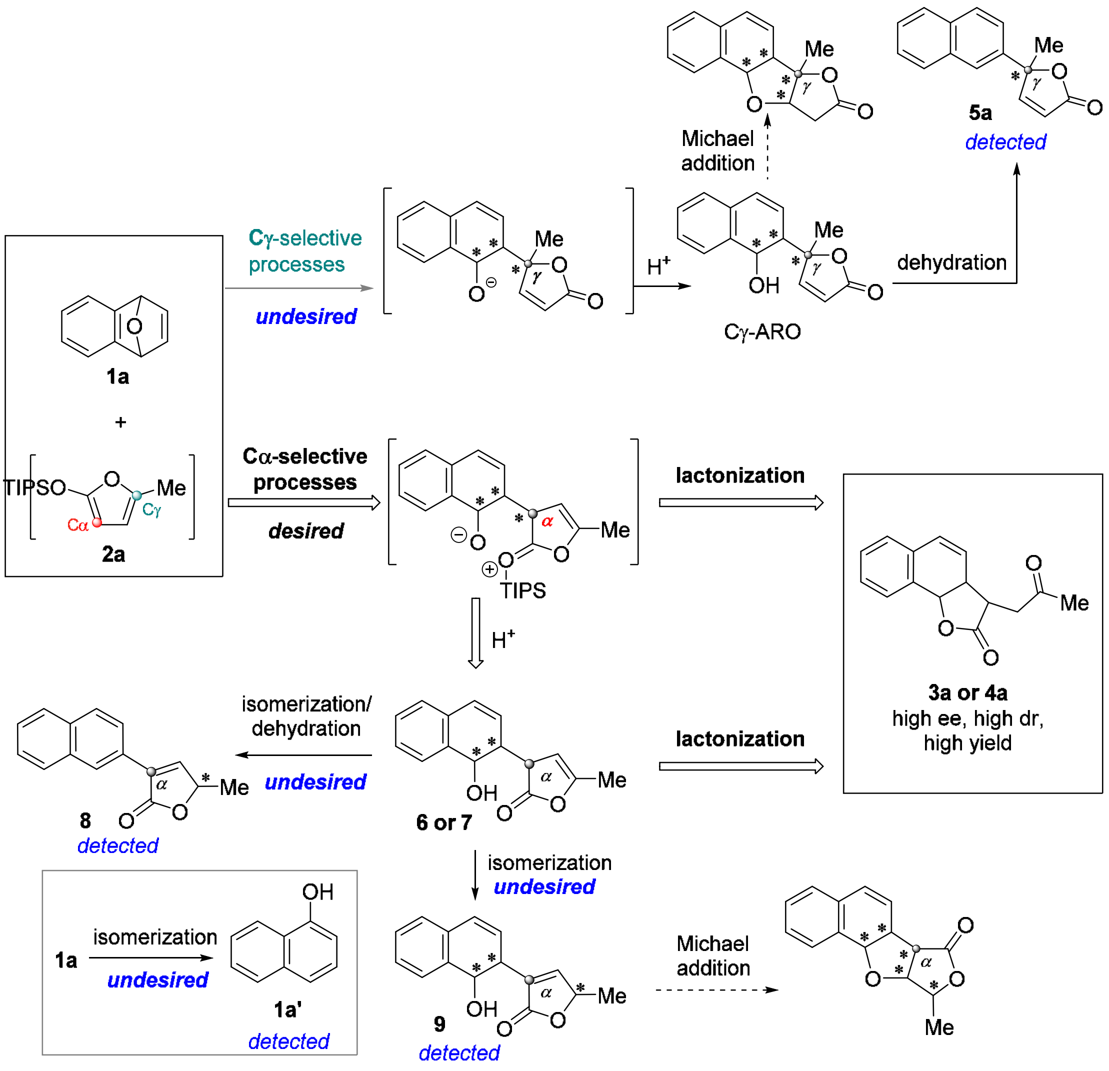

Figure S1. The complex competitive reactions and side products.

2-Silyoxyfurans and oxabenzonobornadienes exhibits rich reactivity, and through monitoring of the reaction system, we have discovered that this reaction involves complex competitive reactions and diverse side products. In addition to obtaining products **3a** and **4a**, we also detected several by-products. For example, the Cγ of 2-silyoxyfurans attacks the oxabenzonobornadienes, resulting in the dehydration product **5a**. The intermediate **6** or **7**, generated by the Cα of 2-silyoxyfurans attacks the oxabenzonobornadienes. It not only could undergo lactonization to afford products **3a** or **4a**, but also could further isomerization/dehydration to yield product **8** and

isomerization to yield product **9**. Raw material **1a** can also be isomerized into **1a'**.

2.2 Optimization of reaction conditions

Table S1. Initial exploration[a]

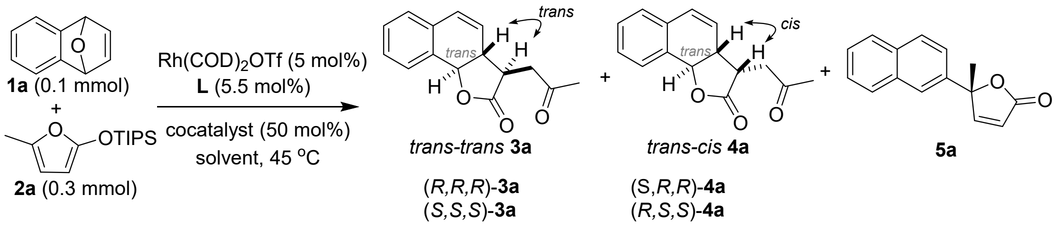

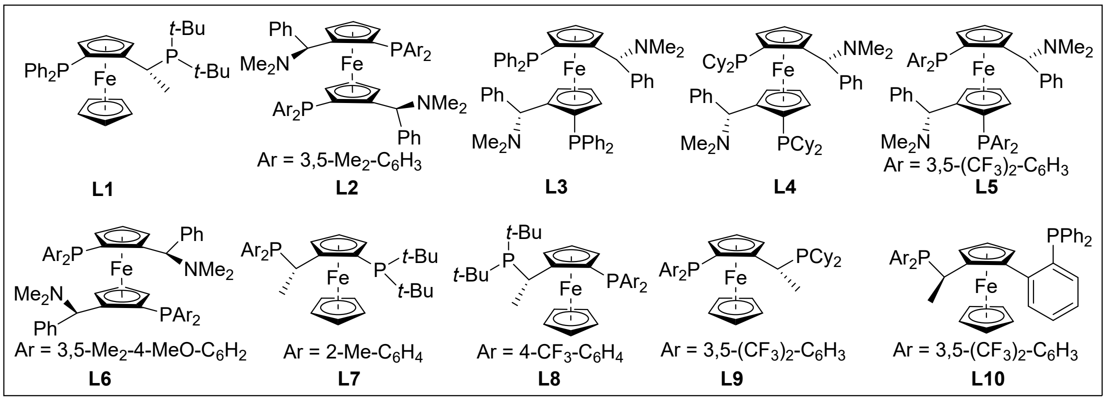

entry	L	co-catalyst	solvent (mL)	time (h)	product of **3a**+**4a** yield (%)[b]	dr[c]	ee (%)[d]	rr[e] (Cα/Cγ)
1	**L1**		THF (1)	3	-	-	-	-
2	**L1**	-	THF (1)	3	23	2:1	-89/-86	2:1
3	**L1**	$Zn(OTf)_2$	THF (1)	12	27	2:1	-89/-86	2:1
4	**L2**	$Zn(OTf)_2$	THF (1)	12	27	5:1	87/86	4:1
5	**L2**	$Zn(OTf)_2$	$PhCH_3$ (1)	12	12	3:1	87/85	1:2
6	**L2**	$Zn(OTf)_2$	PhCl (1)	12	40	1:1	87/82	2:1
7	**L2**	$Zn(OTf)_2$	DCE (1)	12	69	6:1	85/82	3:1
8	**L2**	$Zn(OTf)_2$	$CHCl_3$ (1)	12	47	12:1	92/86	15:1
9	**L2**	$Zn(OTf)_2$	DCM (1)	12	32	3:1	85/83	2:1
10	**L2**	$Zn(OTf)_2$	DMF (1)	12	trace	-	-	-
11	**L2**	$Zn(OTf)_2$	CH_3CN (1)	12	43	4:1	87/87	6:1
12	**L2**	$Zn(OTf)_2$	1.4-dioxane (1)	12	27	2:1	80/82	5:1
13	**L2**	$Zn(OTf)_2$	DCE/$CHCl_3$ (1:1, 1 mL)	12	45	7:1	87/86	12:1
14	**L2**	$Zn(OTf)_2$	DCE/$CHCl_3$ (1:1, 2 mL)	12	56	9:1	87/85	13:1
15	**L1**	$Zn(OTf)_2$		12	50	3:1	-72/-68	12:1
16	**L3**	$Zn(OTf)_2$		12	45	5:1	-71/-51	5:1
17	**L4**	$Zn(OTf)_2$		12	35	1:1	-79/-84	2:1
18	**L5**	$Zn(OTf)_2$		12	56	3:1	-79/-74	7:1
19	**L6**	$Zn(OTf)_2$		12	45	11:1	85/65	>20:1
20	**L7**	$Zn(OTf)_2$		12	trace	-	-	-
21	**L8**	$Zn(OTf)_2$		12	25	2:1	-83/-89	5:1

22	**L9**	$Zn(OTf)_2$	12	31	4:1	-91/-85	2:1
23	**L10**	$Zn(OTf)_2$	12	39	2:1	-84/-73	4:1

[a] According to the Lautens' procedure,[1] the reaction was conducted with **1a** (0.1 mmol), **2a** (0.3 mmol), $Rh(COD)_2OTf$ (5 mol%) and **L** (5.5 mol%) in solvent (1.0 mL) under argon atmosphere. [b] Yield of isolated product refers to the total yield of **3a**+**4a**. [c] The dr value was determined by ^{1}H-NMR spectroscopy and show **3a**/**4a**. [d] The ee value of **3a** or **4a** was determined by HPLC analysis on a chiral stationary phase and show **3a**/**4a**, and their corresponding formula involved were as following: ee((*trans-trans*)-**3a**) = $\frac{[(R,R,R)-\mathbf{3a}]-[(S,S,S)-\mathbf{3a}]}{[(R,R,R)-\mathbf{3a}]+[(S,S,S)-\mathbf{3a}]}$, ee((*trans-cis*)-**4a**) = $\frac{[(S,R,R)-\mathbf{4a}]-[(R,S,S)-\mathbf{4a}]}{[(S,R,R)-\mathbf{4a}]+[(R,S,S)-\mathbf{4a}]}$. [e] rr refers to the regioselective ratio of Cα:Cγ between (**3a**+**4a**) and **5a**,[2] which is determined by ^{1}H NMR spectroscopy. [f] Reaction time is 12 h. ND: not detected.

Table S2. Screening the rhodium salts, ratios of substrate and chiral ligand [a]

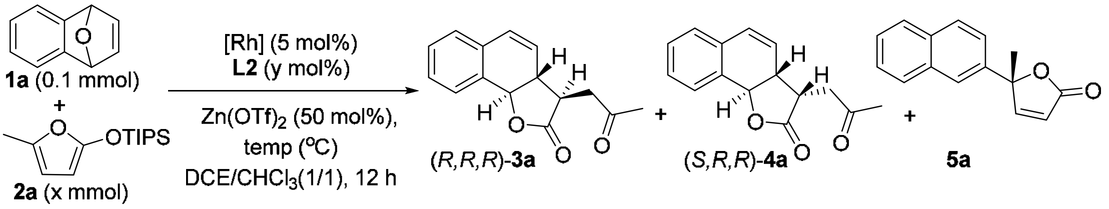

entry	[Rh] salt	**2a** (x mmol)	**L2** (y mol%)	temp. (°C)	Product of **3a**+**4a** yield (%)[b]	dr[c]	ee (%)[d]	rr[e] (Cα/Cγ)
1	$Rh(COD)_2OTf$	3.0	5.5	45	56	9:1	87/85	13:1
2	$Rh(COD)_2BF_4$	3.0	5.5	45	38	9:1	83/75	15:1
3	$Rh(NBD)_2BF_4$	3.0	5.5	45	37	13:1	82/89	>20:1
4	$[Rh(COD)CF_3]_2$	3.0	5.5	45	53	10:1	72/88	14:1
5	$[Rh(COD)OMe]_2$	3.0	5.5	45	13	6:1	79/56	9:1
6	$[Rh(CO)_2Cl]_2$	3.0	5.5	45	16	5:1	58/43	3:1
7	$[Rh(NBD)Cl]_2$	3.0	5.5	45	17	5:1	77/75	4:1
8	$[Rh(COD)OH]_2$	3.0	5.5	45	53	14:1	76/78	9:1
9	$Rh(COD)_2OTf$	1.0	5.5	45	32	6:1	86/85	4:1
10	$Rh(COD)_2OTf$	2.0	5.5	45	46	6:1	87/85	5:1
11	$Rh(COD)_2OTf$	4.0	5.5	45	57	7:1	88/85	10:1
12	$Rh(COD)_2OTf$	5.0	5.5	45	63	12:1	87/86	15:1
13	$Rh(COD)_2OTf$	6.0	5.5	45	57	8:1	87/85	12:1
14	$Rh(COD)_2OTf$	5.0	11	45	71	>20:1	92/-	>20:1
15	$Rh(COD)_2OTf$	5.0	15	45	68	>20:1	92/-	>20:1
16	$Rh(COD)_2OTf$	5.0	11	RT	56	10:1	91/-	>20:1
17	$Rh(COD)_2OTf$	5.0	11	40	68	>20:1	92/-	>20:1
18	$Rh(COD)_2OTf$	5.0	11	50	67	>20:1	91/-	>20:1
19	$Rh(COD)_2OTf$	5.0	11	60	60	>20:1	90/-	>20:1
20	$Rh(COD)_2OTf$	5.0	11	70	50	>20:1	87/-	>20:1

[a] General reaction conditions: **1a** (0.1 mmol), **2a** (x mmol), $Zn(OTf)_2$ (50 mol%), [Rh] salt (5

mol%) and **L2** (y mol%) in the mixture solvent of DCE (1.0 mL) and $CHCl_3$ (1.0 mL) under argon atmosphere for 12 h. [b] Yield of isolated product refers to the total yield of **3a**+**4a**. [c] The dr value was determined by ^{1}H-NMR spectroscopy and show **3a**/**4a**. [d] The ee value of **3a** or **4a** was determined by HPLC analysis on a chiral stationary phase and show **3a**/**4a**. [e] rr refers to the regioselective ratio of Cα:Cγ between (**3a**+**4a**) and **5a**, which is determined by ^{1}H NMR spectroscopy.

Table S3. Screening Lewis acid co-catalysts and additives [a]

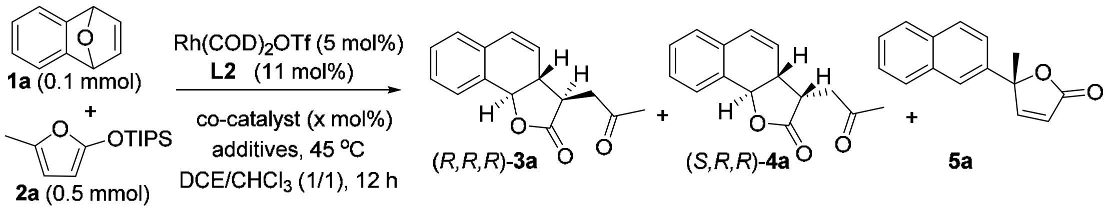

entry	co-catalyst (x mol%)	additives	product of **3a**+**4a** yield (%)[b]	dr[c]	ee (%)[d]	rr[e] (Cα/Cγ)
1	$Zn(OTf)_2$ (0)	none	trace	-	-	-
2	$Zn(OTf)_2$ (10)	none	46	2.3:1	91/89	>20:1
3	$Zn(OTf)_2$ (20)	none	52	3.7:1	92/91	>20:1
4	$Zn(OTf)_2$ (30)	none	60	8:1	92/91	>20:1
5	$Zn(OTf)_2$ (40)	none	65	13:1	92/90	>20:1
6	$Zn(OTf)_2$ (50)	none	71	>20:1	92/-	>20:1
7	$Zn(OTf)_2$ (80)	none	69	10:1	91/90	>20:1
8	$Zn(OTf)_2$ (100)	none	63	9:1	92/90	>20:1
9	$Zn(OTf)_2$ (50)	ZnF_2 (2.0 equiv)	39	4.6:1	87/98	>20:1
10	$Zn(OTf)_2$ (50)	NaF (2.0 equiv)	37	4.3:1	86/98	>20:1
11	$Zn(OTf)_2$ (50)	NH_4F (2.0 equiv)	80	7:1	86/98	>20:1
12	$Zn(OAc)_2$ (50)	none	ND	-	-	-
13	KOTf (50)	none	37	1:13	76/59	>20:1
14	$Bi(OTf)_3$ (50)	none	18	12:1	93/95	4:1
15	$Ca(OTf)_2$ (50)	none	63	1:2.3	82/78	>20:1
16	$Cu(OTf)_2$ (50)	none	21	4.2:1	88/83	3:1
17	$Sn(OTf)_2$ (50)	none	40	1.4:1	93/91	>20:1
18	$Sc(OTf)_3$ (50)	none	27	8:1	95/89	>20:1
19	$Mn(OTf)_2$ (50)	none	80	1.5:1	85/80	>20:1
20	$Co(OTf)_2$ (50)	none	73	7:1	90/95	>20:1
21	$Ni(OTf)_2$ (50)	none	74	1:1.4	90/88	>20:1
22	$Mg(OTf)_2$ (50)	none	77	3.5:1	85/77	>20:1
23	$Ga(OTf)_3$ (50)	none	48	>20:1	94/92	4:1
24	LiOTf (50)	none	50	1:1.5	75/60	>20:1
25	ZnF_2 (50)	none	NR	-	-	-
26	$ZnCl_2$ (50)	none	20	2:1	84/71	>20:1

entry	co-catalyst (x mol%)	additives	product of **3a**+**4a**			rr[e] (Cα/Cγ)
			yield (%)[b]	dr[c]	ee (%)[d]	
27	ZnI_2 (50)	none	32	3:1	39/40	5:1
28	$Al(OTf)_3$ (50)	none	53	>20:1	94/93	9:1

[a] General reaction conditions: **1a** (0.1 mmol), **2a** (0.5 mmol), co-catalyst (50 mol%), $Rh(COD)_2OTf$ (5 mol%) and **L2** (11 mol%) in the mixture solvent of DCE (1.0 mL) and $CHCl_3$ (1.0 mL) under argon atmosphere at 45 °C for 12 h. [b] Yield of isolated product refers to the total yield of **3a**+**4a**. [c] The dr value was determined by ^{1}H-NMR spectroscopy and show **3a**/**4a**. [d] The ee value of **3a** or **4a** was determined by HPLC analysis on a chiral stationary phase and show **3a**/**4a**. [e] rr refers to the regioselective ratio of Cα:Cγ between (**3a**+**4a**) and **5a**, which is determined by ^{1}H NMR spectroscopy.

Table S4. Screening the other unstabilized nucleophiles [a]

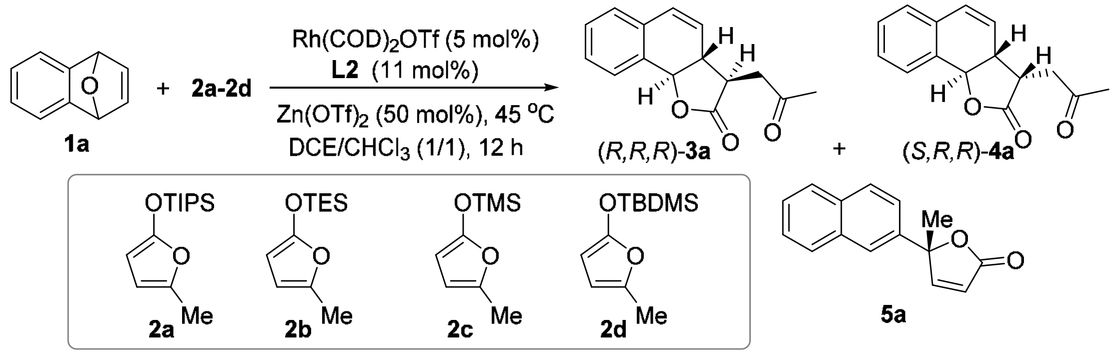

entry	nucleophiles	product of **3a**+**4a**			rr[e] (Cα/Cγ)	yield of Cγ-site product (%)
		yield (%)[b]	dr[c]	ee (%)[d]		
1	**2a**	71	>20:1	92/-	>20:1	-
2	**2b**	trace	-	-	-	-
3	**2c**	trace	-	-	-	-
4	**2d**	65	2:1	80/91	11:1	6 (**5a**)

[a] General reaction conditions: **1a** (0.1 mmol), **2** (0.5 mmol), $Zn(OTf)_2$ (50 mol%), $Rh(COD)_2OTf$ (5 mol%) and **L2** (11 mol%) in DCE (1.0 mL)+$CHCl_3$ (1.0 mL) under argon atmosphere at 45 °C for 12 h. [b] Yield of isolated product refers to the total yield of **3a**+**4a**. [c] The dr value was determined by ^{1}H-NMR spectroscopy and show **3a**/**4a**. [d] The ee value of **3a** or **4a** was determined by HPLC analysis on a chiral stationary phase and show **3a**/**4a**. [e] rr refers to the regioselective ratio of Cα:Cγ between (**3a**+**4a**) and **5a**, which is determined by ^{1}H NMR spectroscopy.

Table S5. Screening the reaction time [a]

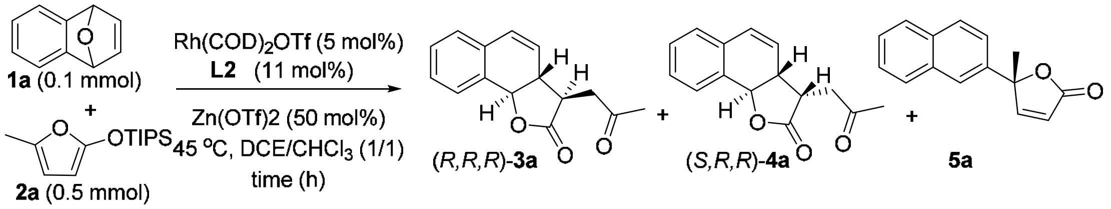

entry	time (h)	product of **3a**+**4a**			rr[e]
		yield (%)[b]	dr[c]	ee (%)[d]	(Cα/Cγ)
1	0.5	38	>20:1	94/-	>20:1
2	1	63	>20:1	92/-	>20:1
3	2	69	>20:1	92/-	>20:1
4	3	70	>20:1	92/-	>20:1
5	4	71	>20:1	92/-	>20:1
6	5	71	>20:1	92/-	>20:1
7	12	71	>20:1	92/-	>20:1

[a] General reaction conditions: **1a** (0.1 mmol), **2a** (0.5 mmol), $Zn(OTf)_2$ (50 mol%), $Rh(COD)_2OTf$ (5 mol%) and **L2** (11 mol%) in DCE (1.0 mL)+$CHCl_3$ (1.0 mL) under argon atmosphere at 45 °C. [b] Yield of isolated product refers to the total yield of **3a+4a**. [c] The dr value was determined by ^{1}H-NMR spectroscopy and show **3a**/**4a**. [d] The ee value of **3a** or **4a** was determined by HPLC analysis on a chiral stationary phase and show **3a**/**4a**. [e] rr refers to the regioselective ratio of Cα:Cγ between (**3a**+**4a**) and **5a**, which is determined by ^{1}H NMR spectroscopy.

Table S6. Screening the combined co-catalysts and additives for (*S,R,R*)-**4a** [a]

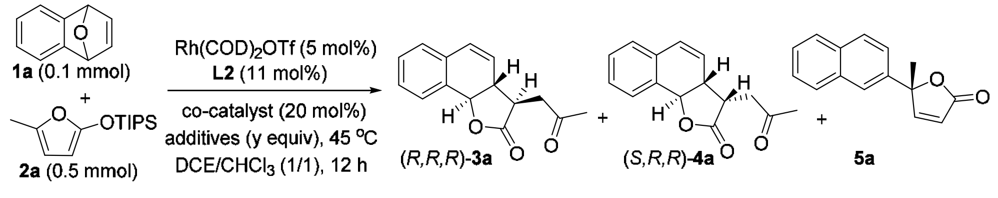

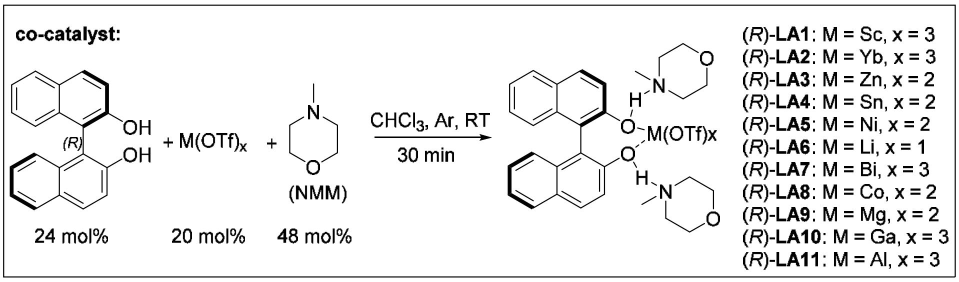

entry	co-catalyst	additives (y equiv)	product of **3a**+**4a**			rr[e] (Cα/Cγ)
			yield (%)[b]	dr[c]	ee (%)[d]	
1	KOTf	none	<10%	-	-	-
2	$Ca(OTf)_2$	none	<10%	-	-	-
3	(*R*)-**LA1** (Sc^{3+})	none	77	1:11	79/97	9:1

entry	co-catalyst	additives (y equiv)	product of **3a**+**4a**			rr[e] (Cα/Cγ)
			yield (%)[b]	dr[c]	ee (%)[d]	
4	(*R*)-**LA2** (Yb^{3+})	none	37	1:4.6	92/93	>20:1
5	(*R*)-**LA3** (Zn^{2+})	none	50	1:2	96/97	>20:1
6	(*R*)-**LA4** (Sn^{2+})	none	45	<1:20	-/93	>20:1
7	(*R*)-**LA5** (Ni^{2+})	none	<10%	-	-	-
8	(*R*)-**LA6** **(**Li^{+}**)**	none	<10%	-	-	-
9	(*R*)-**LA7** **(**Bi^{3+}**)**	none	28	<1:20	-/93	>20:1
10	(*R*)-**LA8** (Ni^{2+})	none	trace	-	-	-
11	(*R*)-**LA9** (Mg^{2+})	none	32	<1:20	-/90	>20:1
12	(*R*)-**LA10** (Ga^{3+})	none	20	<1:20	-/92	>20:1
13	(*R*)-**LA11** (Al^{3+})	none	18	<1:20	-/93	>20:1
14	(*R*)-**LA4** (Sn^{2+})	none	45	<1:20	-/93	>20:1
15	(*R*)-**LA4** (Sn^{2+})	*i*-PrOH (2.0)	61	<1:20	-/91	>20:1
16	(*R*)-**LA4** (Sn^{2+})	H_2O (2.0)	44	<1:20	-/85	>20:1
147	(*R*)-**LA4** (Sn^{2+})	*t*-BuOH (2.0)	67	<1:20	-/89	>20:1
18	(*R*)-**LA4** (Sn^{2+})	HFIP (2.0)	65	<1:20	-/88	>20:1
19	(*R*)-**LA4** (Sn^{2+})	TFE (2.0)	68	<1:20	-/88	>20:1
20	(*R*)-**LA4** (Sn^{2+})	TMSOH (2.0)	51	<1:20	-/87	>20:1
21	(*R*)-**LA4** (Sn^{2+})	TFE (1.0)	37	<1:20	-/89	>20:1
22	(*R*)-**LA4** (Sn^{2+})	TFE (2.0)	68	<1:20	-/88	>20:1
23	(*R*)-**LA4** (Sn^{2+})	TFE (3.5)	73	<1:20	-/89	>20:1
24	(*R*)-**LA4** (Sn^{2+})	TFE (5.0)	62	<1:20	-/85	>20:1
25	(*R*)-**LA4** (Sn^{2+})	TFE (10.0)	67	<1:20	-/79	>20:1
26	(*R*)-**LA4** (Sn^{2+})	TFE (20.0)	66	<1:20	-/69	>20:1
27	(*R*)-**LA1** (Sc^{3+})	TFE (3.5)	83	1:13	80/97	>20:1
28	(*R*)-**LA2** (Yb^{3+})	TFE (3.5)	62	1:7	92/93	>20:1

[a] General reaction conditions: **1a** (0.1 mmol), **2a** (0.5 mmol), $Rh(COD)_2OTf$ (5 mol%), **L2** (11 mol%), cocatalyst (based on 20 mol% Lewis acid salt, in $CHCl_3$) in the solvent of DCE (1.0 mL) under argon atmosphere at 45 °C for 12 h. The solution of cocatalyst was prepared freshly in a separate reaction tube with the indicated Lewis acid (0.02 mmol), (*R*)-BINOL (0.024 mmol) and NMM (0.048 mmol) in $CHCl_3$ (1.0 mL) at RT for 30 min. [b] Yield of isolated product refers to the total yield of **3a**+**4a**. [c] The dr value was determined by ^{1}H-NMR spectroscopy and show **3a**/**4a**. [d] The ee value of **3a** or **4a** was determined by HPLC analysis on a chiral stationary phase and show **3a**/**4a**. [e] rr refers to the regioselective ratio of Cα:Cγ between (**3a**+**4a**) and **5a**, which is determined by ^{1}H NMR spectroscopy. HFIP: $(CF_3)_2CHOH$. TFE: CF_3CH_2OH. TMSOH: $(Me)_3SiOH$.

Table S7. Screening the solvents for (*S,R,R*)-**4a** [a]

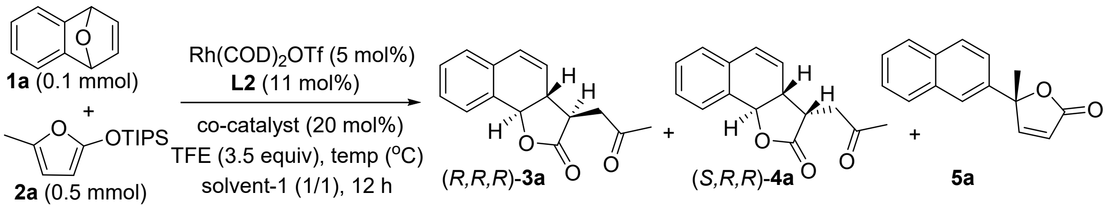

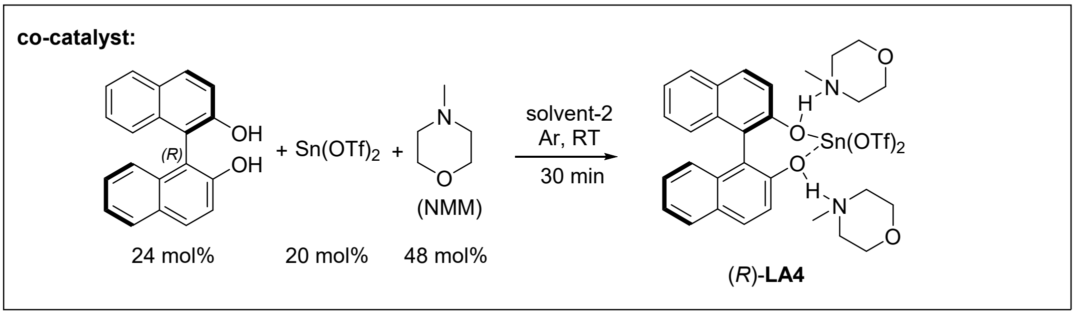

entry	solvent-1 [solvent-2]	temp. (°C)	product of **3a**+**4a** yield (%)[b]	dr[c]	ee (%)[d]	rr[e] (Cα/Cγ)
1	DCE [$CHCl_3$]	45	73	<1:20	-/89	>20:1
2	DCE [DCE]	45	46	<1:20	-/68	>20:1
3	$CHCl_3$ [$CHCl_3$]	45	76	<1:20	-/95	>20:1
4	$CHCl_3$ [$CHCl_3$]	RT	NR	-	-	-
5	$CHCl_3$ [$CHCl_3$]	40	70	<1:20	-/95	>20:1
6	$CHCl_3$ [$CHCl_3$]	50	72	<1:20	-/95	>20:1
7	$CHCl_3$ [$CHCl_3$]	70	54	1:10	95/94	>20:1
8	$CHCl_3$ [$CHCl_3$]	90	31	1:5	95/93	>20:1

[a] General reaction conditions: **1a** (0.1 mmol), **2a** (0.5 mmol), Rh(COD)$_2$OTf (5 mol%), **L2** (11 mol%), (*R*)-**LA4** (based on 20 mol% Sn(OTf)$_2$, in solvent-2) and TFE (3.5 equiv) in the indicated solvent-1 under argon atmosphere at 45 °C for 12 h. The solution of cocatalyst (*R*)-**LA4** was prepared freshly in a separate reaction tube with the Sn(OTf)$_2$ (0.02 mmol), (*R*)-BINOL (0.024 mmol) and NMM (0.048 mmol) in the indicated solvent-2 (1.0 mL) at RT for 30 min. [b] Yield of isolated product refers to the total yield of **3a**+**4a**. [c] The dr value was determined by ^{1}H-NMR spectroscopy and show **3a**/**4a**. [d] The ee value of **3a** or **4a** was determined by HPLC analysis on a chiral stationary phase and show **3a**/**4a**. [e] rr refers to the regioselective ratio of Cα:Cγ between (**3a**+**4a**) and **5a**, which is determined by ^{1}H NMR spectroscopy. NR: no reaction, both starting materials didn't react at RT.

Table S8. The influence of combined catalysts for (*S,R,R*)-**4a** [a]

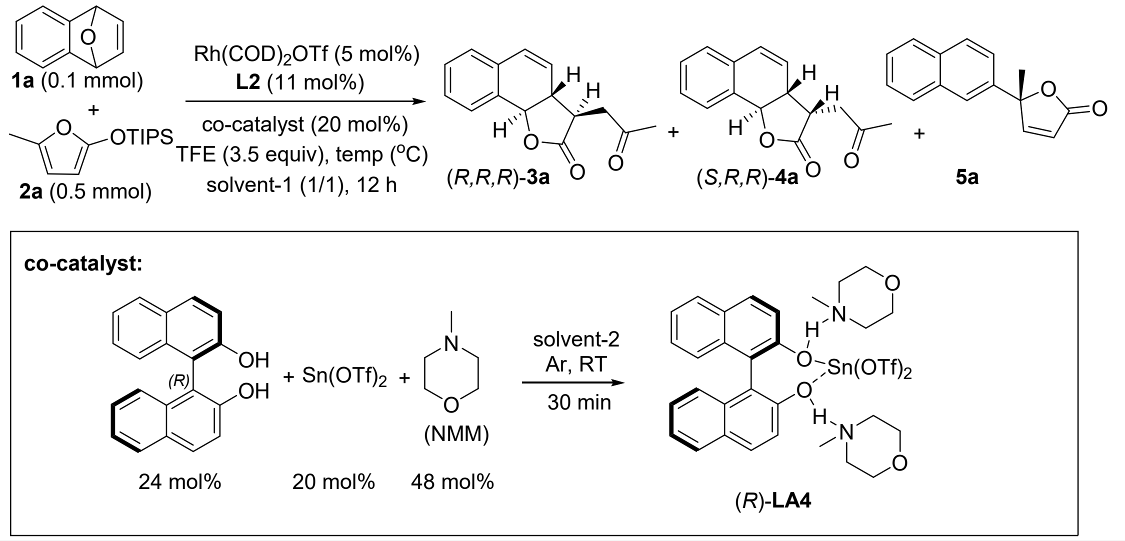

entry	co-catalyst (x:y:z)	additives	product of **3a**+**4a** yield (%)[b]	dr[c]	ee (%)[d]	rr[e] (Cα/Cγ)
1	(*R*)-**LA4**	TFE	76	<1:20	-/95	>20:1
2	(±)-**LA4**	TFE	71	<1:20	-/95	>20:1
3	(*S*)-**LA4**	TFE	69	<1:20	-/95	>20:1
4	(*R*)-BINOL:Sn(OTf)2: NMM = 1.2:0:2.4	TFE	trace	-	-	>20:1
5	(*R*)-BINOL:Sn(OTf)2: NMM = 0:1:2.4	TFE	73	1:9	99/94	>20:1
6	(*R*)-BINOL:Sn(OTf)2: NMM =1.2:1:0	TFE	61	1:1.4	97/93	>20:1
7	(*R*)-BINOL:Sn(OTf)2: NMM = 0:1:2.4	none	73	1:13	99/94	>20:1
8	Sn(OTf)2	TFE	68	1:1.5	93/91	-
9	(*R*)-BINOL	TFE	trace	-	-	-
10	NMM	TFE	trace	-	-	-
11	-	TFE	trace	-	-	-

[a] General reaction conditions: **1a** (0.1 mmol), **2a** (0.5 mmol), Rh(COD)$_2$OTf (5 mol%), **L2** (11 mol%), cocatalyst (based on 20 mol% Sn(OTf)$_2$, in $CHCl_3$) and TFE (3.5 equiv) in the solvent of $CHCl_3$ (1.0 mL) under argon atmosphere for 12 h. The solution of cocatalyst (*R*)-**LA4** was prepared freshly in a separate reaction tube with the Sn(OTf)$_2$ (x mmol), BINOL (y mmol) and NMM (z mmol) in $CHCl_3$ (1.0 mL) at RT for 30 min. [b] Yield of isolated product refers to the total yield of **3a**+**4a**. [c] The dr value was determined by ^{1}H-NMR spectroscopy and show **3a**/**4a**. [d] The ee value of **3a** or **4a** was determined by HPLC analysis on a chiral stationary phase and show **3a**/**4a**. [e] rr refers to the regioselective ratio of Cα:Cγ between (**3a**+**4a**) and **5a**, which is determined by ^{1}H NMR spectroscopy.

3. General procedures for catalytic asymmetric [3+2]-annulation

3.1 General procedure A for the synthesis of (*R,R,R*)-3

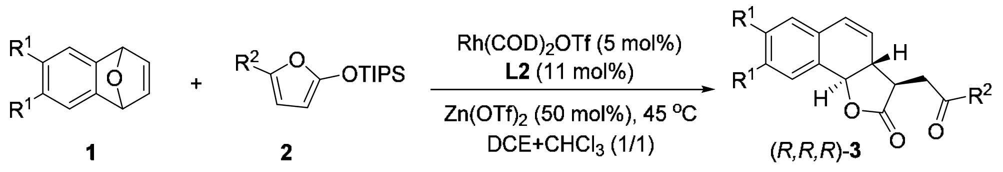

Under an argon atmosphere, Rh(COD)$_2$OTf (2.34 mg, 5.0 mol%) and **L2** (10.3 mg, 11 mol%) were dissolved in the mixture solvents of DCE (1.0 mL) and $CHCl_3$ (1.0 mL) and the resulting solution was stirred for 30 min at RT. Subsequently, Zn(OTf)$_2$ (18.2 mg, 50 mol%), **1** (0.1 mmol) and **2** (0.5 mmol) were added. The reaction mixture was stirred under argon atmosphere at 45 °C for 4-12 h. After reaction completion, the solvent was evaporated under reduced pressure. The residue was purified by flash column chromatography on silica gel (petroleum ether/ ethyl acetate = 10:1) to afford (*R,R,R*)-**3**.

***Note*:** All *racemic* products **3** were synthesized in the presence of the racemic ligands (**L2** and *ent*-**L2** (1:1)). Due to the error in the preparation of racemic ligand, some racemic products mixture have a low ee values but below 5%.

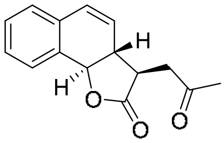

(*R,R,R*)-**3a**

According to the general procedure A: White solid, 17.2 mg, 71% yield, >20:1 dr, 92% ee;

^{1}H NMR (600 MHz, CDCl$_3$) δ 7.47-7.41 (m, 1H), 7.33-7.28 (m, 2H), 7.21-7.16 (m, 1H), 6.57 (dd, *J* = 9.5, 3.0 Hz, 1H), 6.30 (dd, *J* = 9.5, 2.3 Hz, 1H), 5.04 (d, *J* = 14.4 Hz, 1H), 3.28-3.14 (m, 2H), 2.75-2.59 (m, 2H), 2.25 (s, 3H);

^{13}C NMR (101 MHz, CDCl$_3$) δ 205.2, 176.8, 134.4, 132.8, 131.7, 128.2, 127.8, 127.0, 124.1, 123.9, 79.1, 46.7, 42.7, 40.4, 30.1;

HRMS (ESI) m/z: $[M+H]^+$ calculated for $C_{15}H_{15}O_3^+$: 243.1016, found: 243.1016;

HPLC analysis: Daicel CHIRALCEL® OD-H, *n*-hexane/*i*-PrOH = 85/15, flow rate = 1.0 mL/min, λ = 254 nm, retention time: t $_{major}$ = 12.8 min, t $_{minor}$ = 15.6 min;

$[\alpha]_D^{25}$ = +48.3 (c = 0.1, CHCl$_3$).

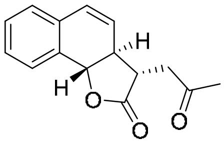

(S,S,S)-**3a**

According to the general procedure A, *ent*-**L2** was used as the ligand. White solid, 19.3 mg, 80% yield, 10:1 dr, 87% ee;

Spectral data were in agreement with those of the enantiomer reported above;

HRMS (ESI) m/z: $[M+H]^+$ calculated for $C_{15}H_{15}O_3^+$: 243.1016, found: 243.1017;

HPLC analysis: Daicel CHIRALCEL® OD-H, *n*-hexane/*i*-PrOH = 85/15, flow rate = 1.0 mL/min, λ = 254 nm, retention time: t_{minor} = 13.0 min, t_{major} = 15.2 min;

$[\alpha]_D^{25}$ = -30.5 (c = 0.1, $CHCl_3$).

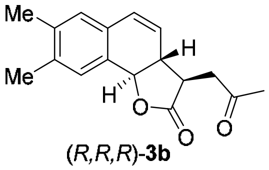

(R,R,R)-**3b**

According to the general procedure A: White solid, 19.7 mg, 73% yield, 11:1 dr, 90% ee;

^{1}H NMR (600 MHz, $CDCl_3$) δ 7.21 (s, 1H), 6.97 (s, 1H), 6.51 (dd, J = 9.5, 3.0 Hz, 1H), 6.21 (dd, J = 9.5, 2.2 Hz, 1H), 4.98 (d, J = 14.3 Hz, 1H), 3.25-3.15 (m, 2H), 2.69-2.57 (m, 2H), 2.28 (s, 3H), 2.25 (d, 6H);

^{13}C NMR (151 MHz, $CDCl_3$) δ 205.3, 177.8, 136.6, 136.0, 131.6, 130.0, 129.5, 128.4, 124.8, 123.0, 80.8, 47.5, 42.8, 40.7, 30.1, 19.7, 19.5;

HRMS (ESI) m/z: $[M+H]^+$ calculated for $C_{17}H_{19}O_3^+$: 271.1329, found: 271.1324;

HPLC analysis: Daicel CHIRALCEL® OD-H, *n*-hexane/*i*-PrOH = 90/10, flow rate = 1.0 mL/min, λ = 254 nm, retention time: t_{major} = 15.0 min, t_{minor} = 17.5 min;

$[\alpha]_D^{25}$ = +6.5 (c = 0.1, $CHCl_3$).

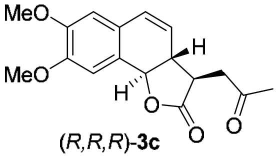

(R,R,R)-**3c**

According to the general procedure A: White solid, 21.7 mg, 72% yield, 7:1 rr, 10:1 dr, 89% ee;

^{1}**H NMR (400 MHz, CDCl$_3$)** δ 7.00 (s, 1H), 6.74 (s, 1H), 6.48 (dd, *J* = 9.5, 3.0 Hz, 1H), 6.19 (dd, *J* = 9.5, 2.2 Hz, 1H), 4.97 (d, *J* = 14.6 Hz, 1H), 3.89 (d, *J* = 15.5 Hz, 6H), 3.27-3.13 (m, 2H), 2.72-2.57 (m, 2H), 2.25 (s, 3H);

^{13}**C NMR (101 MHz, CDCl$_3$)** δ 205.3, 177.8, 148.8, 148.3, 129.2, 127.2, 125.0, 124.0, 110.8, 105.6, 80.8, 56.1, 56.1, 47.5, 42.7, 40.7, 30.1;

HRMS (ESI) m/z: [M+H]$^+$ calculated for $C_{17}H_{19}O_5^+$: 303.1227, found: 303.1226;

HPLC analysis: Daicel CHIRALCEL® OD-H, *n*-hexane/*i*-PrOH = 80/20, flow rate = 1.0 mL/min, λ = 254 nm, retention time: t $_{major}$ = 19.3 min, t $_{minor}$ = 23.0 min;

[α]$_D^{25}$ = +25.8 (c = 0.092, CHCl$_3$).

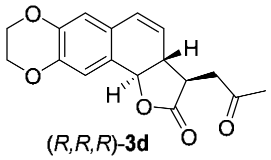

(*R*,*R*,*R*)-**3d**

According to the general procedure A: White solid, 18.9 mg, 63% yield, 14:1 rr, 10:1 dr, 92% ee;

^{1}**H NMR (600 MHz, CDCl$_3$)** δ 6.94 (s, 1H), 6.72 (s, 1H), 6.43 (dd, *J* = 9.5, 2.9 Hz, 1H), 6.16 (dd, *J* = 9.5, 2.3 Hz, 1H), 4.92 (d, *J* = 14.2 Hz, 1H), 4.29-4.22 (m, 4H), 3.25-3.12 (m, 2H), 2.68-2.55 (m, 2H), 2.24 (s, 3H);

^{13}**C NMR (151 MHz, CDCl$_3$)** δ 205.3, 177.7, 143.2, 142.8, 129.0, 127.9, 126.0, 124.1, 116.3, 111.4, 80.5, 64.5, 64.3, 47.4, 42.8, 40.7, 30.1;

HRMS (ESI) m/z: [M+H]$^+$ calculated for $C_{17}H_{17}O_5^+$: 301.1071, found: 301.1068;

HPLC analysis: Daicel CHIRALCEL® OD-H, *n*-hexane/*i*-PrOH = 75/25, flow rate = 1.0 mL/min, λ = 254 nm, retention time: t $_{major}$ = 17.6 min, t $_{minor}$ = 24.6 min;

[α]$_D^{25}$ = -25.9 (c = 0.088, CHCl$_3$).

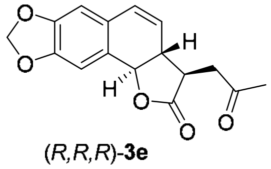

(*R*,*R*,*R*)-**3e**

According to the general procedure A: White solid, 18.3 mg, 64% yield, 14:1 rr, 13:1 dr, 92% ee;

^{1}**H NMR (600 MHz, CDCl$_3$)** δ 6.95 (s, 1H), 6.69 (s, 1H), 6.44 (dd, *J* = 9.5, 2.9 Hz, 1H), 6.20 (dd, *J* = 9.5, 2.2 Hz, 1H), 5.97 (d, *J* = 7.0 Hz, 2H), 4.91 (d, *J* = 14.7 Hz, 1H), 3.25-3.13 (m, 2H), 2.69-2.55 (m, 2H), 2.24 (s, 3H);

^{13}C NMR (151 MHz, CDCl$_3$) δ 205.3, 177.6, 147.2, 147.1, 129.4, 128.7, 126.4, 124.2, 107.8, 103.4, 101.2, 80.8, 47.3, 42.7, 40.6, 30.1;

HRMS (ESI) m/z: $[M+H]^+$ calculated for $C_{16}H_{15}O_5^+$: 287.0914, found: 287.0915;

HPLC analysis: Daicel CHIRALPAK® AD-H, *n*-hexane/*i*-PrOH = 80/20, flow rate = 1.0 mL/min, λ = 254 nm, retention time: t $_{major}$ = 15.6 min, t $_{minor}$ = 19.8 min;

$[\alpha]_D^{25}$ = +56.5 (c = 0.1, CHCl$_3$).

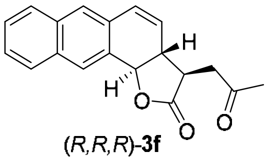

(*R*,*R*,*R*)-**3f**

According to the general procedure A, when Zn(OTf)$_2$ as the co-catalyst: White solid, 19.8 mg, 68% yield, 8:1 dr, 92% ee;

Yb(OTf)$_3$ was used as the co-catalyst instead of Zn(OTf)$_2$: 20.4 mg, 70% yield, >20:1 dr, 92% ee;

^{1}H NMR (400 MHz, CDCl$_3$) δ 7.90-7.76 (m, 3H), 7.63 (s, 1H), 7.56-7.43 (m, 2H), 6.73 (dd, *J* = 9.6, 3.0 Hz, 1H), 6.33 (dd, *J* = 9.6, 2.2 Hz, 1H), 5.16 (d, *J* = 13.2 Hz, 1H), 3.34-3.20 (m, 2H), 2.81-2.62 (m, 2H), 2.26 (s, 3H);

^{13}C NMR (101 MHz, CDCl$_3$) δ 205.3, 177.6, 132.9, 132.6, 131.8, 130.5, 129.7, 128.1, 127.9, 126.7, 126.4, 126.2, 126.1, 120.3, 80.6, 47.2, 42.8, 40.8, 30.1;

HRMS (ESI) m/z: $[M+H]^+$ calculated for $C_{19}H_{17}O_3^+$: 293.1172, found: 293.1176;

HPLC analysis: Daicel CHIRALCEL® OD-H, *n*-hexane/*i*-PrOH = 80/20, flow rate = 1.0 mL/min, λ = 254 nm, retention time: t $_{major}$ = 17.8 min, t $_{minor}$ = 22.2 min;

$[\alpha]_D^{25}$ = +45.0 (c = 0.1, CHCl$_3$).

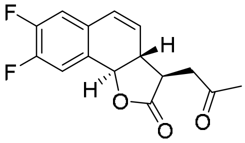

(*R*,*R*,*R*)-**3g**

According to the general procedure A: White solid, 20.8 mg, 75% yield, 11:1 dr, 90% ee;

^{1}H NMR (500 MHz, CDCl$_3$) δ 7.29-7.23 (m, 1H), 7.02 (dd, *J* = 10.5, 7.5 Hz, 1H), 6.48 (dd, *J* = 9.6, 3.0 Hz, 1H), 6.36 (dd, *J* = 9.7, 2.2 Hz, 1H), 4.94 (d, *J* = 14.5 Hz, 1H), 3.25 (dd, *J* = 18.6, 3.6 Hz, 1H), 3.19 (ddd, *J* = 12.2, 8.3, 3.5 Hz, 1H), 2.71-2.61 (m, 2H), 2.25 (s, 3H);

^{13}C NMR (126 MHz, CDCl$_3$) δ 205.3, 177.1, 150.7 (dd, J = 27.6, 13.0 Hz), 148.7 (dd, J = 31.1, 13.0 Hz), 130.9 (dd, J = 6.4, 3.6 Hz), 129.0 (dd, J = 6.4, 4.3 Hz), 128.0, 127.0 (d, J = 2.1 Hz), 116.2 (d, J = 18.4 Hz), 111.8 (d, J = 19.7 Hz), 79.4, 46.7, 42.7, 40.4, 30.1;

^{19}F NMR (471 MHz, CDCl$_3$) δ -136.72, -138.97;

HRMS (ESI) m/z: $[M+H]^+$ calculated for $C_{15}H_{13}F_2O_3^+$: 279.0827, found: 279.0827;

HPLC analysis: Daicel CHIRALCEL® OD-H, *n*-hexane/*i*-PrOH = 80/20, flow rate = 1.0 mL/min, λ = 254 nm, retention time: t_{major} = 15.1 min, t_{minor} = 18.0 min;

$[\alpha]_D^{25}$ = -4.8 (c = 0.1, $CHCl_3$).

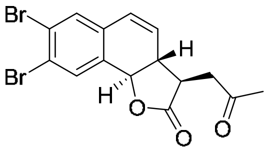

(*R*,*R*,*R*)-**3h**

According to the general procedure A, when $Zn(OTf)_2$ was used as the co-catalyst: White solid, 31 mg, 78% yield, 8:1 dr, 94% ee;

$Lu(OTf)_3$ was used as the co-catalyst at 50 °C: 29 mg, 73% yield, 11:1 dr, 94% ee;

^{1}H NMR (400 MHz, CDCl$_3$) δ 7.67 (s, 1H), 7.44 (s, 1H), 6.51-6.37 (m, 2H), 4.92 (d, J = 14.4 Hz, 1H), 3.34-3.07 (m, 2H), 2.77-2.53 (m, 2H), 2.25 (s, 3H);

^{13}C NMR (101 MHz, CDCl$_3$) δ 205.2, 176.8, 134.4, 132.8, 131.7, 128.2, 127.8, 127.0, 124.1, 123.9, 79.1, 46.7, 42.7, 40.4, 30.1;

HRMS (ESI) m/z: $[M+H]^+$ calculated for $C_{15}H_{13}Br_2O_3^+$: 398.9226, found: 398.9222;

HPLC analysis: Daicel CHIRALCEL® OD-H, *n*-hexane/*i*-PrOH = 80/20, flow rate = 1.0 mL/min, λ = 254 nm, retention time: t_{major} = 18.3 min, t_{minor} = 21.4 min;

$[\alpha]_D^{25}$ = +6.2 (c = 0.1, $CHCl_3$).

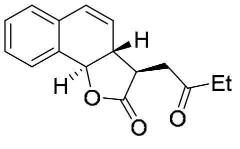

(*R*,*R*,*R*)-**3i**

According to the general procedure A, when $Zn(OTf)_2$ was used as the co-catalyst: White solid, 17.9 mg, 70% yield, 7:1 dr, 91% ee;

$Yb(OTf)_3$ was used as the co-catalyst: 18.9 mg, 74% yield, 10:1 dr, 91% ee;

^{1}H NMR (400 MHz, $CDCl_3$) δ 7.48-7.40 (m, 1H), 7.34-7.27 (m, 2H), 7.23-7.16 (m, 1H), 6.57 (dd, *J* = 9.6, 3.0 Hz, 1H), 6.30 (dd, *J* = 9.5, 2.2 Hz, 1H), 5.04 (d, *J* = 14.4 Hz, 1H), 3.31-3.14 (m, 2H), 2.77-2.41 (m, 4H), 1.11 (t, *J* = 7.3 Hz, 3H);
^{13}C NMR (101 MHz, $CDCl_3$) δ 208.3, 177.8, 134.1, 132.3, 129.6, 128.0, 127.9, 126.9, 126.1, 121.6, 80.6, 47.1, 41.4, 40.6, 36.2, 7.7;
HRMS (ESI) m/z: $[M+H]^+$ calculated for $C_{16}H_{17}O_3^+$: 257.1172, found: 257.1174;
HPLC analysis: Daicel CHIRALCEL® OD-H, *n*-hexane/*i*-PrOH = 90/10, flow rate = 1.0 mL/min, λ = 254 nm, retention time: t_{major} = 14.1 min, t_{minor} = 17.0 min;
$[\alpha]_D^{25}$ = -4.75 (c = 0.08, $CHCl_3$).

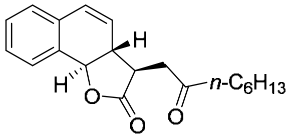

(*R*,*R*,*R*)-**3j**

According to the general procedure A, when $Zn(OTf)_2$ was used as the co-catalyst: White solid, 26.6 mg, 85% yield, 7:1 dr, 83% ee;
$Al(OTf)_3$ was used as the co-catalyst: 25.0 mg, 80% yield, 15:1 dr, 89% ee;
^{1}H NMR (600 MHz, $CDCl_3$) δ 7.47-7.40 (m, 1H), 7.36-7.27 (m, 2H), 7.22-7.15 (m, 1H), 6.56 (dd, *J* = 9.5, 3.1 Hz, 1H), 6.29 (dd, *J* = 9.5, 2.2 Hz, 1H), 5.03 (d, *J* = 14.4 Hz, 1H), 3.31-3.14 (m, 2H), 2.67 (ddt, *J* = 14.3, 12.7, 2.6 Hz, 1H), 2.64-2.58 (m, 1H), 2.49 (tq, *J* = 16.6, 7.5 Hz, 2H), 1.62-1.55 (m, 2H), 1.36-1.26 (m, 6H), 0.92-0.82 (m, 3H);
^{13}C NMR (151 MHz, $CDCl_3$) δ 208.0, 177.8, 134.1, 132.3, 129.6, 128.0, 127.9, 126.9, 126.1, 121.6, 80.6, 47.1, 43.0, 41.8, 40.6, 31.5, 28.8, 23.7, 22.4, 14.0;
HRMS (ESI) m/z: $[M+H]^+$ calculated for $C_{20}H_{25}O_3^+$: 313.1798, found: 313.1795;
HPLC analysis: Daicel CHIRALPAK® AD-H, *n*-hexane/*i*-PrOH = 95/5, flow rate = 1.0 mL/min, λ = 254 nm, retention time: t_{major} = 11.2 min, t_{minor} = 15.6 min;
$[\alpha]_D^{25}$ = -13.53 (c = 0.068, $CHCl_3$).

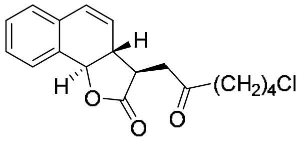

(*R*,*R*,*R*)-**3k**

According to the general procedure A, when $Zn(OTf)_2$ was used as the co-catalyst: White solid, 20.7 mg, 65% yield, 10:1 dr, 98% ee;

$Lu(OTf)_3$ was used as the co-catalyst: 21.0 mg, 66% yield, 10:1 dr, 94% ee;

^{1}H NMR (500 MHz, CDCl$_3$) δ 7.48-7.41 (m, 1H), 7.35-7.27 (m, 2H), 7.22-7.17 (m, 1H), 6.58 (dd, *J* = 9.5, 3.1 Hz, 1H), 6.28 (dd, *J* = 9.5, 2.3 Hz, 1H), 5.04 (d, *J* = 14.4 Hz, 1H), 3.60-3.50 (m, 2H), 3.27-3.20 (m, 1H), 3.18 (dd, *J* = 18.2, 4.1 Hz, 1H), 2.74-2.47 (m, 4H), 1.86-1.73 (m, 4H);

^{13}C NMR (101 MHz, CDCl$_3$) δ 207.1, 177.7, 134.0, 132.2, 129.7, 128.0, 127.9, 127.0, 125.9, 121.6, 80.6, 46.9, 44.5, 42.0, 41.7, 40.6, 31.7, 20.9;

HRMS (ESI) m/z: $[M+H]^+$ calculated for $C_{18}H_{20}ClO_3^+$: 319.1095, found: 319.1094;

HPLC analysis: Daicel CHIRALPAK® AD-H, *n*-hexane/*i*-PrOH = 90/10, flow rate = 1.0 mL/min, λ = 254 nm, retention time: t_{major} = 18.0 min, t_{minor} = 29.7 min;

$[\alpha]_D^{25}$ = +60.2 (c = 0.1, $CHCl_3$).

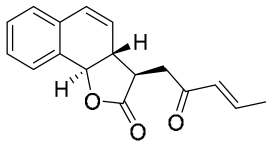

(*R*,*R*,*R*)-**3l**

According to the general procedure A, when $Zn(OTf)_2$ was used as the co-catalyst: White solid, 19 mg, 71% yield, 7:1 dr, 89% ee;

$Al(OTf)_3$ was used as the co-catalyst: 17.7 mg, 66% yield, >20:1 dr, 89% ee;

^{1}H NMR (600 MHz, CDCl$_3$) δ 7.48-7.42 (m, 1H), 7.34-7.27 (m, 2H), 7.22-7.16 (m, 1H), 6.95 (dq, *J* = 15.8, 6.8 Hz, 1H), 6.56 (dd, *J* = 9.5, 3.0 Hz, 1H), 6.32 (dd, *J* = 9.5, 2.3 Hz, 1H), 6.19 (dq, *J* = 15.7, 1.7 Hz, 1H), 5.05 (d, *J* = 14.5 Hz, 1H), 3.36 (dd, *J* = 18.1, 3.3 Hz, 1H), 3.28 (ddd, *J* = 12.4, 9.0, 3.3 Hz, 1H), 2.74 (dd, *J* = 18.1, 9.0 Hz, 1H), 2.68 (ddt, *J* = 14.9, 12.7, 2.6 Hz, 1H), 1.94 (dd, *J* = 6.8, 1.7 Hz, 3H);

^{13}C NMR (151 MHz, CDCl$_3$) δ 196.6, 178.0, 144.2, 134.1, 132.3, 131.4, 129.5, 128.0, 127.9, 126.9, 126.4, 121.6, 80.6, 47.4, 40.7, 39.4, 18.4;

HRMS (ESI) m/z: $[M+H]^+$ calculated for $C_{17}H_{17}O_3^+$: 269.1172, found: 269.1177;

HPLC analysis: Daicel CHIRALPAK® AD-H, *n*-hexane/*i*-PrOH = 90/10, flow rate = 1.0 mL/min, λ = 254 nm, retention time: t_{major} = 13.7 min, t_{minor} = 19.8 min;

$[\alpha]_D^{20}$ = +170.5 (c = 0.1, $CHCl_3$).

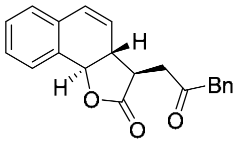

(*R*,*R*,*R*)-**3m**

According to the general procedure A: White solid, 24.8 mg, 78% yield, >20:1 dr, 90% ee;

^{1}H NMR (600 MHz, $CDCl_3$) δ 7.44-7.39 (m, 1H), 7.36-7.32 (m, 2H), 7.31-7.26 (m, 3H), 7.24-7.19 (m, 2H), 7.20-7.15 (m, 1H), 6.54 (dd, *J* = 9.5, 2.9 Hz, 1H), 6.23 (dd, *J* = 9.6, 2.2 Hz, 1H), 5.01 (d, *J* = 14.4 Hz, 1H), 3.83-3.72 (m, 2H), 3.26-3.15 (m, 2H), 2.70-2.62 (m, 1H), 2.59 (ddt, *J* = 14.9, 12.4, 2.6 Hz, 1H);

^{13}C NMR (151 MHz, $CDCl_3$) δ 205.4, 177.6, 134.0, 133.5, 132.2, 129.6, 129.4, 128.9, 128.0, 127.9, 127.3, 126.9, 126.0, 121.6, 80.6, 50.3, 47.0, 41.1, 40.7;

HRMS (ESI) m/z: $[M+H]^+$ calculated for $C_{21}H_{19}O_3^+$: 319.1329, found: 319.1324;

HPLC analysis: Daicel CHIRALPAK® AD-H, *n*-hexane/*i*-PrOH = 85/15, flow rate = 1.0 mL/min, λ = 254 nm, retention time: t_{major} = 14.4 min, t_{minor} = 19.5 min;

$[\alpha]_D^{25}$ = +17.7 (c = 0.08, $CHCl_3$).

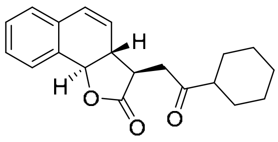

(*R*,*R*,*R*)-**3n**

According to the general procedure A, when $Zn(OTf)_2$ was used as the co-catalyst: White solid, 24.8 mg, 80% yield, 6:1 dr, 92% ee;

$Ga(OTf)_3$ was used as the co-catalyst: 22.6 mg, 73% yield, 11:1 dr, 92% ee;

^{1}H NMR (500 MHz, $CDCl_3$) δ 7.48-7.39 (m, 1H), 7.35-7.27 (m, 2H), 7.21-7.16 (m, 1H), 6.56 (dd, *J* = 9.6, 3.0 Hz, 1H), 6.27 (dd, *J* = 9.6, 2.3 Hz, 1H), 5.03 (d, *J* = 14.4 Hz, 1H), 3.28-3.16 (m, 2H), 2.72-2.59 (m, 2H), 2.40 (tt, *J* = 11.2, 3.4 Hz, 1H), 1.94-1.83 (m, 2H), 1.83-1.75 (m, 2H), 1.72-1.64 (m, 1H), 1.44-1.14 (m, 5H);

^{13}C NMR (126 MHz, $CDCl_3$) δ 211.0, 178.0, 134.1, 132.3, 129.6, 128.0, 127.9, 126.9, 126.2, 121.6, 80.5, 50.9, 47.2, 40.6, 39.9, 28.4, 28.3, 25.7, 25.6, 25.5;

HRMS (ESI) m/z: $[M+H]^+$ calculated for $C_{20}H_{23}O_3^+$: 311.1642, found: 311.1645;

HPLC analysis: Daicel CHIRALPAK® AS-H, *n*-hexane/*i*-PrOH = 90/10, flow rate = 1.0 mL/min, λ = 254 nm, retention time: t_{minor} = 16.2 min, t_{major} = 21.5 min;

$[\alpha]_D^{25}$ = +42 (c = 0.1, $CHCl_3$).

3.2 General procedure B for the synthesis of (*S,R,R*)-4

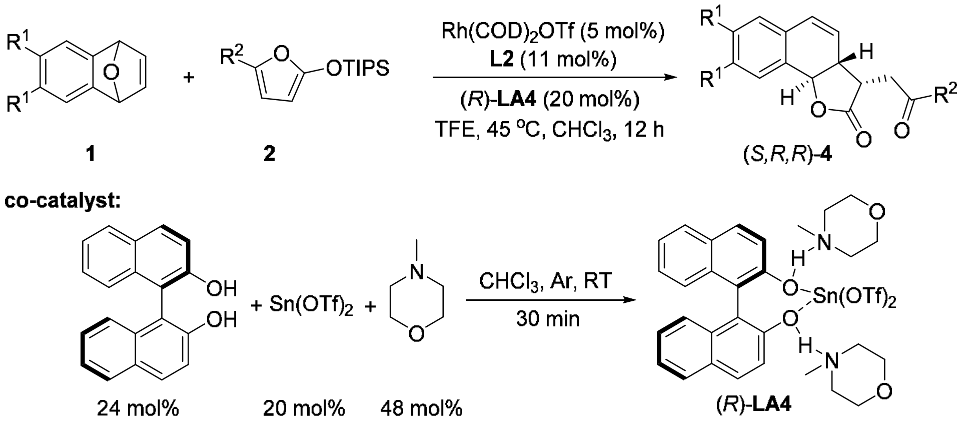

In a reaction tube, $Rh(COD)_2OTf$ (2.34 mg, 5.0 mol%) and **L2** (10.3 mg, 11 mol%) were dissolved in $CHCl_3$ (1.0 mL) and the resulting solution was stirred for 30 min at RT. In another reaction tube, (*R*)-BINOL (6.84 mg, 24 mol%), $Sn(OTf)_2$ (8.34 mg, 20 mol%), NMM (4.9 mg, 48 mol%) were dissolved in $CHCl_3$ (1.0 mL) and the resulting solution of cocatalyst (*R*)-**LA4** also was stirred for 30 min at RT. Then, the cocatalyst (*R*)-**LA4** in $CHCl_3$ were added to the first reaction tube. Subsequently, TFE (35 mg, 3.5 eq), **1** (0.1 mmol) and **2** (0.5 mmol) were added. The reaction mixture was stirred under argon atmosphere at 45 °C for 12 h. After reaction completion, the solvent was evaporated under reduced pressure. The residue was purified by flash column chromatography on silica gel (petroleum ether/ ethyl acetate = 10:1) to afford the (*S,R,R*)-**4**.

Note: All *racemic* products **4** were synthesized in the presence of the racemic ligands (**L2** and *ent*-**L2** (1:1)). Due to the error in the preparation of racemic ligand, some racemic product mixture have a low ee values but below 5%.

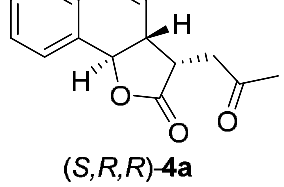

(*S,R,R*)-**4a**

According to the general procedure B: White solid, 18.4 mg, 76% yield, >20:1 dr, 95% ee;

^{1}H NMR (400 MHz, $CDCl_3$) δ 7.47-7.40 (m, 1H), 7.35-7.27 (m, 2H), 7.21-7.15 (m, 1H), 6.56 (dd, J = 9.6, 3.1 Hz, 1H), 5.99 (dd, J = 9.5, 2.4 Hz, 1H), 5.14 (d, J = 15.0

Hz, 1H), 3.41 (ddd, J = 10.7, 7.8, 3.2 Hz, 1H), 3.18 (ddt, J = 15.0, 7.8, 2.8 Hz, 1H), 3.00 (dd, J = 18.4, 10.6 Hz, 1H), 2.85 (dd, J = 18.4, 3.1 Hz, 1H), 2.28 (s, 3H);

^{13}C NMR (101 MHz, $CDCl_3$) δ 204.3, 178.8, 133.9, 132.0, 131.0, 128.2, 127.9, 127.0, 124.5, 121.8, 80.2, 44.4, 39.3, 38.6, 30.0;

HRMS (ESI) m/z: $[M+H]^+$ calculated for $C_{15}H_{15}O_3^+$: 243.1016, found: 243.1012;

HPLC analysis: Daicel CHIRALPAK® AS-H, *n*-hexane/*i*-PrOH = 85/15, flow rate = 1.0 mL/min, λ = 254 nm, retention time: t_{minor} = 26.5 min, t_{major} = 30.0 min;

$[\alpha]_D^{25}$ = +118.3 (c = 0.1, $CHCl_3$).

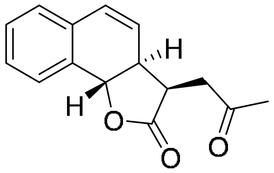

(*R,S,S*)-**4a**

According to the general procedure B: *ent*-**L2** was used as the ligand. White solid, 16.9 mg, 70% yield, >20:1 dr, 96% ee;

Spectral data were in agreement with those of the enantiomer reported above;

HPLC analysis: Daicel CHIRALPAK® AS-H, *n*-hexane/*i*-PrOH = 85/15, flow rate = 1.0 mL/min, λ = 254 nm,, retention time: t_{major} = 26.6 min, t_{minor} = 31.2 min;

HRMS (ESI) m/z: $[M+H]^+$ calculated for $C_{15}H_{15}O_3^+$: 243.1016, found: 243.1015;

$[\alpha]_D^{25}$ = -90.0 (c = 0.1, $CHCl_3$).

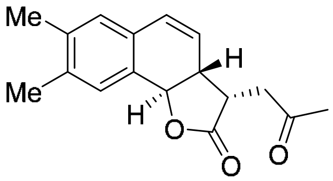

(*S,R,R*)-**4b**

According to the general procedure B: White solid, 19.2 mg, 71% yield, >20:1 dr, 93% ee;

^{1}H NMR (500 MHz, $CDCl_3$) δ 7.20 (s, 1H), 6.96 (s, 1H), 6.49 (dd, J = 9.6, 3.2 Hz, 1H), 5.90 (dd, J = 9.5, 2.4 Hz, 1H), 5.08 (d, J = 15.0 Hz, 1H), 3.39 (ddd, J = 10.7, 7.8, 3.1 Hz, 1H), 3.11 (ddt, J = 15.1, 7.8, 2.8 Hz, 1H), 2.99 (dd, J = 18.4, 10.6 Hz, 1H), 2.83 (dd, J = 18.3, 3.1 Hz, 1H), 2.28 (d, 6H), 2.25 (s, 3H);

^{13}C NMR (126 MHz, $CDCl_3$) δ 204.3, 178.9, 136.8, 136.0, 131.4, 130.8, 129.7, 128.4, 123.3, 123.1, 80.3, 44.8, 39.3, 38.6, 30.0, 19.7, 19.5;

HRMS (ESI) m/z: $[M+H]^+$ calculated for $C_{17}H_{19}O_3^+$: 271.1329, found: 271.1329;
HPLC analysis: Daicel CHIRALCEL® OD-H, *n*-hexane/*i*-PrOH = 90/10, flow rate = 1.0 mL/min, λ = 254 nm, retention time: t_{minor} = 15.4 min, t_{major} = 17.4 min;
$[\alpha]_D^{25}$ = +124.6 (c = 0.1, $CHCl_3$).

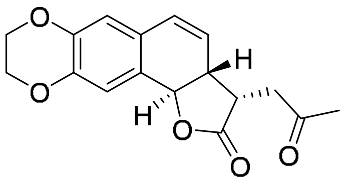

(*S*,*R*,*R*)-**4d**

According to the general procedure B: White solid, 18.3 mg, 61% yield, >20:1 dr, 94% ee;
^{1}H NMR (600 MHz, $CDCl_3$) δ 6.92 (s, 1H), 6.70 (s, 1H), 6.41 (dd, *J* = 9.5, 3.1 Hz, 1H), 5.85 (dd, *J* = 9.5, 2.4 Hz, 1H), 5.01 (d, *J* = 14.8 Hz, 1H), 4.29-4.21 (m, 4H), 3.38 (ddd, *J* = 10.7, 7.8, 3.1 Hz, 1H), 3.09 (ddt, *J* = 14.9, 7.7, 2.8 Hz, 1H), 2.97 (dd, *J* = 18.4, 10.7 Hz, 1H), 2.81 (dd, *J* = 18.4, 3.1 Hz, 1H), 2.26 (s, 3H);
^{13}C NMR (151 MHz, $CDCl_3$) δ 204.3, 178.8, 143.3, 142.8, 130.3, 127.6, 125.7, 122.6, 116.3, 111.5, 80.0, 64.5, 64.3, 44.6, 39.2, 38.6, 30.0;
HRMS (ESI) m/z: $[M+H]^+$ calculated for $C_{17}H_{17}O_5^+$: 301.1071, found: 301.1070;
HPLC analysis: Daicel CHIRALCEL® OD-H, *n*-hexane/*i*-PrOH = 80/20, flow rate = 1.0 mL/min, λ = 254 nm, retention time: t_{minor} = 25.2 min, t_{major} = 29.0 min;
$[\alpha]_D^{25}$ = +141.9 (c = 0.1, $CHCl_3$).

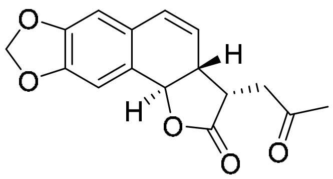

(*S*,*R*,*R*)-**4e**

According to the general procedure B, 3.0 eq **2** was used at 40 °C: White solid, 16.6 mg, 58% yield, 92% ee;
^{1}H NMR (500 MHz, $CDCl_3$) δ 6.93 (s, 1H), 6.68 (s, 1H), 6.42 (dd, *J* = 9.6, 3.1 Hz, 1H), 5.97 (dd, *J* = 5.6, 1.4 Hz, 2H), 5.89 (dd, *J* = 9.6, 2.4 Hz, 1H), 5.02 (d, *J* = 15.4 Hz, 1H), 3.38 (ddd, *J* = 10.8, 7.9, 3.1 Hz, 1H), 3.10 (ddt, *J* = 15.4, 7.8, 2.8 Hz, 1H), 2.99 (dd, *J* = 18.5, 10.7 Hz, 1H), 2.82 (dd, *J* = 18.5, 3.1 Hz, 1H), 2.27 (s, 3H);

^{13}C NMR (126 MHz, $CDCl_3$) δ 204.3, 178.7, 147.3, 147.1, 130.7, 128.4, 126.1, 122.6, 107.9, 103.6, 101.2, 80.3, 44.6, 39.3, 38.6, 30.0;

HRMS (ESI) m/z: $[M+H]^+$ calculated for $C_{16}H_{15}O_5^+$: 287.0914, found: 287.0913;

HPLC analysis: Daicel CHIRALCEL® OD-H, *n*-hexane/*i*-PrOH = 70/30, flow rate = 1.0 mL/min, λ = 254 nm, retention time: t_{minor} = 13.7 min, t_{major} = 15.8 min;

$[\alpha]_D^{25}$ = +171.8 (c = 0.084, $CHCl_3$).

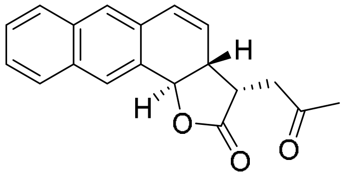

(*S*,*R*,*R*)-**4f**

According to the general procedure B: White solid, 22.2 mg, 76% yield, >20:1 dr, 94% ee;

^{1}H NMR (600 MHz, $CDCl_3$) δ 7.87-7.78 (m, 3H), 7.63 (s, 1H), 7.52-7.45 (m, 2H), 6.72 (dd, *J* = 9.5, 3.1 Hz, 1H), 6.01 (dd, *J* = 9.5, 2.4 Hz, 1H), 5.25 (d, *J* = 13.9 Hz, 1H), 3.46 (ddd, *J* = 10.7, 7.7, 3.1 Hz, 1H), 3.24 (ddt, *J* = 13.9, 7.7, 2.8 Hz, 1H), 3.02 (dd, *J* = 18.4, 10.6 Hz, 1H), 2.88 (dd, *J* = 18.4, 3.1 Hz, 1H), 2.30 (s, 3H);

^{13}C NMR (151 MHz, $CDCl_3$) δ 204.3, 178.7, 132.9, 132.8, 131.7, 131.2, 130.3, 128.1, 127.9, 126.8, 126.5, 126.4, 124.7, 120.6, 80.2, 44.4, 39.3, 38.8, 30.0;

HRMS (ESI) m/z: $[M+H]^+$ calculated for $C_{19}H_{17}O_3^+$: 293.1172, found: 293.1168;

HPLC analysis: Daicel CHIRALCEL® OD-H, *n*-hexane/*i*-PrOH = 80/20, flow rate = 1.0 mL/min, λ = 254 nm, retention time: t_{minor} = 21.3 min, t_{major} = 25.8 min;

$[\alpha]_D^{25}$ = +145 (c = 0.1, $CHCl_3$).

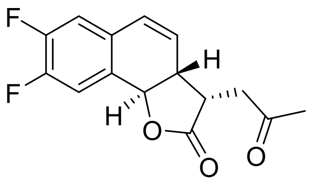

(*S*,*R*,*R*)-**4g**

According to the general procedure B, the reaction was conducted at 40 °C: White solid, 17.3 mg, 63% yield, >20:1 dr, 95% ee;

^{1}H NMR (500 MHz, $CDCl_3$) δ 7.26-7.20 (m, 1H), 7.01 (dd, *J* = 10.6, 7.5 Hz, 1H), 6.46 (dd, *J* = 9.6, 3.1 Hz, 1H), 6.04 (dd, *J* = 9.7, 2.4 Hz, 1H), 5.03 (d, *J* = 15.2 Hz,

1H), 3.41 (ddd, *J* = 10.8, 7.8, 3.1 Hz, 1H), 3.14 (ddt, *J* = 15.2, 7.8, 2.8 Hz, 1H), 2.97 (dd, *J* = 18.5, 10.7 Hz, 1H), 2.85 (dd, *J* = 18.5, 3.1 Hz, 1H), 2.28 (s, 3H);

^{13}C NMR (126 MHz, $CDCl_3$) δ 204.1, 178.1, 150.8 (dd, *J* = 13.4, 9.5 Hz), 148.8 (t, *J* = 13.1 Hz), 130.8 (dd, *J* = 5.8, 4.0 Hz), 129.3 (d, *J* = 1.9 Hz), 128.8 (dd, *J* = 6.3, 4.4 Hz), 125.5 (d, *J* = 2.3 Hz), 116.3 (d, *J* = 18.4 Hz), 112.0 (dd, *J* = 19.8 Hz), 79.0, 44.1, 39.3, 38.5, 30.0;

^{19}F NMR (471 MHz, $CDCl_3$) δ -136.4, -138.9;

HRMS (ESI) m/z: $[M+H]^+$ calculated for $C_{15}H_{13}F_2O_3^+$: 279.0827, found: 279.0827;

HPLC analysis: Daicel CHIRALCEL® OD-H, *n*-hexane/*i*-PrOH = 80/20, flow rate = 1.0 mL/min, λ = 254 nm, retention time: t_{minor} = 16.1 min, t_{major} = 18.5 min;

$[\alpha]_D^{25}$ = +92.4 (c = 0.088, $CHCl_3$).

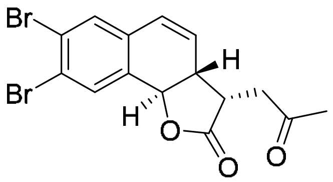

(*S*,*R*,*R*)-**4h**

According to the general procedure B: White solid, 25.9 mg, 65% yield, >20:1 dr, 90% ee;

^{1}H NMR (600 MHz, $CDCl_3$) δ 7.68 (s, 1H), 7.44 (s, 1H), 6.47 (dd, *J* = 9.5, 3.1 Hz, 1H), 6.09 (dd, *J* = 9.5, 2.4 Hz, 1H), 5.02 (d, *J* = 15.1 Hz, 1H), 3.41 (ddd, *J* = 10.7, 7.7, 3.0 Hz, 1H), 3.13 (ddt, *J* = 15.1, 7.8, 2.8 Hz, 1H), 2.96 (dd, *J* = 18.5, 10.6 Hz, 1H), 2.86 (dd, *J* = 18.5, 3.1 Hz, 1H), 2.28 (s, 3H);

^{13}C NMR (151 MHz, $CDCl_3$) δ 204.1, 177.9, 134.3, 132.5, 131.8, 129.2, 127.3, 126.8, 124.2, 124.1, 78.7, 44.0, 39.2, 38.4, 30.0;

HRMS (ESI) m/z: $[M+H]^+$ calculated for $C_{15}H_{13}Br_2O_3^+$: 398.9226, found: 398.9230;

HPLC analysis: Daicel CHIRALCEL® OD-H, *n*-hexane/*i*-PrOH = 70/30, flow rate = 1.0 mL/min, λ = 254 nm, retention time: t_{minor} = 15.4 min, t_{major} = 21.6 min;

$[\alpha]_D^{25}$ = +100.1 (c = 0.1, $CHCl_3$).

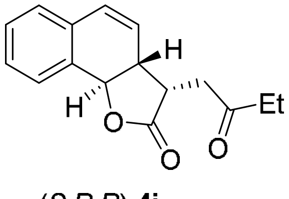

(*S*,*R*,*R*)-**4i**

According to the general procedure B: White solid, 17.7 mg, 69% yield, >20:1 dr, 95% ee;

^{1}H NMR (500 MHz, CDCl$_3$) δ 7.46-7.40 (m, 1H), 7.34-7.27 (m, 2H), 7.21-7.15 (m, 1H), 6.55 (dd, *J* = 9.5, 3.1 Hz, 1H), 5.97 (dd, *J* = 9.5, 2.4 Hz, 1H), 5.14 (d, *J* = 15.0 Hz, 1H), 3.43 (ddd, *J* = 10.8, 7.8, 3.1 Hz, 1H), 3.17 (ddt, *J* = 15.1, 7.9, 2.8 Hz, 1H), 2.97 (dd, *J* = 18.2, 10.7 Hz, 1H), 2.81 (dd, *J* = 18.2, 3.2 Hz, 1H), 2.61-2.49 (m, 2H), 1.14 (t, *J* = 7.4 Hz, 3H);

^{13}C NMR (126 MHz, CDCl$_3$) δ 207.2, 178.9, 133.9, 132.0, 131.0, 128.2, 127.9, 127.0, 124.6, 121.8, 80.1, 44.4, 38.6, 38.0, 36.1, 7.9;

HRMS (ESI) m/z: $[M+H]^+$ calculated for $C_{16}H_{17}O_3^+$: 257.1172, found: 257.1167;

HPLC analysis: Daicel CHIRALPAK® AD-H, *n*-hexane/*i*-PrOH = 85/15, flow rate = 1.0 mL/min, λ = 254 nm, retention time: t_{minor} = 10.4 min, t_{major} = 11.1 min;

$[\alpha]_D^{25}$ = +108.3 (c = 0.1, CHCl$_3$).

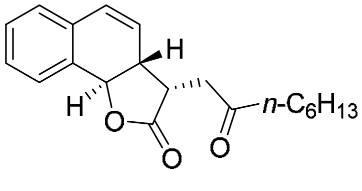

(*S*,*R*,*R*)-**4j**

According to the general procedure B: White solid, 20.3 mg, 65% yield, >20:1 dr, 95% ee;

^{1}H NMR (500 MHz, CDCl$_3$) δ 7.47-7.40 (m, 1H), 7.34-7.27 (m, 2H), 7.21-7.15 (m, 1H), 6.55 (dd, *J* = 9.6, 3.1 Hz, 1H), 5.97 (dd, *J* = 9.6, 2.5 Hz, 1H), 5.13 (d, *J* = 15.0 Hz, 1H), 3.42 (ddd, *J* = 10.8, 7.8, 3.0 Hz, 1H), 3.17 (ddt, *J* = 15.1, 7.8, 2.8 Hz, 1H), 2.97 (dd, *J* = 18.3, 10.8 Hz, 1H), 2.80 (dd, *J* = 18.3, 3.1 Hz, 1H), 2.57-2.44 (m, 2H), 1.65-1.58 (m, 2H), 1.36-1.26 (m, 6H), 0.95-0.85 (m, 3H);

^{13}C NMR (126 MHz, CDCl$_3$) δ 206.9, 178.9, 133.9, 132.0, 130.9, 128.2, 127.9, 127.0, 124.6, 121.8, 80.1, 44.4, 42.9, 38.5, 38.4, 31.5, 28.8, 23.9, 22.4, 14.0;

HRMS (ESI) m/z: $[M+H]^+$ calculated for $C_{20}H_{25}O_3^+$: 313.1798, found: 313.1801;

HPLC analysis: Daicel CHIRALCEL® OD-H, *n*-hexane/*i*-PrOH = 95/5, flow rate =

1.0 mL/min, λ = 254 nm, retention time: t $_{minor}$ = 22.9 min, t $_{major}$ = 24.1 min;
[α]$_D^{25}$ = +107.2 (c = 0.1, $CHCl_3$).

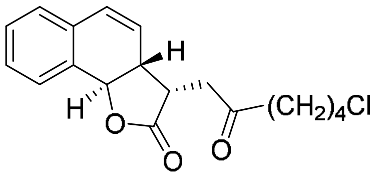

(*S,R,R*)-**4k**

According to the general procedure B: White solid, 19.1 mg, 60% yield, >20:1 dr, 97% ee;

^{1}H NMR (600 MHz, $CDCl_3$) δ 7.47-7.40 (m, 1H), 7.34-7.28 (m, 2H), 7.21-7.15 (m, 1H), 6.56 (dd, *J* = 9.5, 3.1 Hz, 1H), 5.96 (dd, *J* = 9.5, 2.4 Hz, 1H), 5.14 (d, *J* = 15.0 Hz, 1H), 3.62-3.53 (m, 2H), 3.42 (ddd, *J* = 10.8, 7.7, 3.2 Hz, 1H), 3.18 (ddt, *J* = 15.1, 7.8, 2.8 Hz, 1H), 2.97 (dd, *J* = 18.2, 10.6 Hz, 1H), 2.82 (dd, *J* = 18.1, 3.3 Hz, 1H), 2.65-2.50 (m, 2H), 1.87-1.77 (m, 4H);

^{13}C NMR (151 MHz, $CDCl_3$) δ 206.0, 178.7, 133.9, 132.0, 131.1, 128.3, 128.0, 127.1, 124.4, 121.8, 80.2, 44.5, 44.4, 41.9, 38.6, 38.5, 31.8, 21.1;

HRMS (ESI) m/z: $[M+H]^+$ calculated for $C_{18}H_{20}ClO_3^+$: 319.1095, found: 319.1098;

HPLC analysis: Daicel CHIRALPAK® AD-H, *n*-hexane/*i*-PrOH = 90/10, flow rate = 1.0 mL/min, λ = 254 nm, retention time: t $_{minor}$ = 28.6 min, t $_{major}$ = 31.8 min;
[α]$_D^{25}$ = +113.9 (c = 0.1, $CHCl_3$).

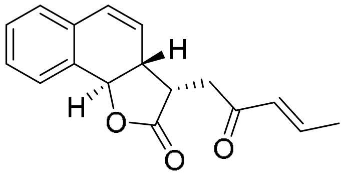

(*S,R,R*)-**4l**

According to the general procedure B, 3.0 equiv of **2** was used at 40 °C: White solid, 16.6 mg, 62% yield, >20:1 dr, 93% ee;

^{1}H NMR (500 MHz, $CDCl_3$) δ 7.47-7.41 (m, 1H), 7.34-7.27 (m, 2H), 7.21-7.14 (m, 1H), 6.97 (dq, *J* = 15.9, 6.9 Hz, 1H), 6.53 (dd, *J* = 9.6, 3.1 Hz, 1H), 6.23 (dd, *J* = 15.9, 1.7 Hz, 1H), 6.00 (dd, *J* = 9.5, 2.4 Hz, 1H), 5.16 (d, *J* = 15.1 Hz, 1H), 3.48 (ddd, *J* = 10.7, 7.8, 2.8 Hz, 1H), 3.20 (ddt, *J* = 15.1, 7.8, 2.8 Hz, 1H), 3.11 (dd, *J* = 18.0, 11.0 Hz, 1H), 2.94 (dd, *J* = 17.9, 2.9 Hz, 1H), 1.96 (dd, *J* = 6.9, 1.7 Hz, 3H);

^{13}C NMR (126 MHz, CDCl$_3$) δ 195.5, 179.1, 144.5, 134.0, 132.1, 131.1, 130.7, 128.1, 127.9, 127.0, 125.0, 121.8, 80.2, 44.5, 38.6, 35.9, 18.4;

HRMS (ESI) m/z: $[M+H]^+$ calculated for $C_{17}H_{17}O_3^+$: 269.1172, found: 269.1174;

HPLC analysis: Daicel CHIRALPAK® AD-H, *n*-hexane/*i*-PrOH = 90/10, flow rate = 1.0 mL/min, λ = 254 nm, retention time: t_{major} = 16.8 min, t_{minor} = 17.8 min;

$[\alpha]_D^{25}$ = +230.0 (c = 0.024, CHCl$_3$).

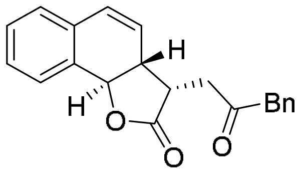

(*S*,*R*,*R*)-**4m**

According to the general procedure B, 3.0 equiv of **2** was used at 40 °C: White solid, 20.1 mg, 63% yield, >20:1 dr, 98% ee;

^{1}H NMR (600 MHz, CDCl$_3$) δ 7.42-7.38 (m, 1H), 7.38-7.32 (m, 2H), 7.32-7.27 (m, 3H), 7.26-7.20 (m, 2H), 7.19-7.13 (m, 1H), 6.47 (dd, *J* = 9.6, 3.1 Hz, 1H), 5.84 (dd, *J* = 9.5, 2.4 Hz, 1H), 5.04 (d, *J* = 15.1 Hz, 1H), 3.80 (s, 2H), 3.39 (ddd, *J* = 10.8, 7.8, 3.0 Hz, 1H), 3.13 (ddt, *J* = 15.1, 7.7, 3.0 Hz, 1H), 3.01 (dd, *J* = 18.5, 10.9 Hz, 1H), 2.82 (dd, *J* = 18.5, 3.0 Hz, 1H);

^{13}C NMR (151 MHz, CDCl$_3$) δ 204.3, 178.7, 133.8, 133.3, 132.0, 130.8, 129.4, 129.0, 128.2, 127.9, 127.5, 127.0, 124.3, 121.8, 80.1, 50.2, 44.3, 38.5, 37.6;

HRMS (ESI) m/z: $[M+H]^+$ calculated for $C_{21}H_{19}O_3^+$: 319.1329, found: 319.1326;

HPLC analysis: Daicel CHIRALPAK® AS-H, *n*-hexane/*i*-PrOH = 85/15, flow rate = 1.0 mL/min, λ = 254 nm, retention time: t_{major} = 19.7 min, t_{minor} = 26.5 min;

$[\alpha]_D^{25}$ = +69.1 (c = 0.08, CHCl$_3$).

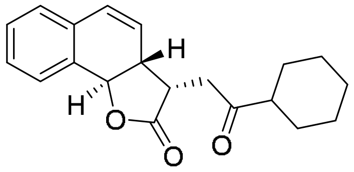

(*S*,*R*,*R*)-**4n**

According to the general procedure B: White solid, 23.2 mg, 75% yield, >20:1 dr, 94% ee;

^{1}H NMR (500 MHz, CDCl$_3$) δ 7.47-7.38 (m, 1H), 7.35-7.27 (m, 2H), 7.21-7.14 (m, 1H), 6.54 (dd, J = 9.5, 3.1 Hz, 1H), 5.92 (dd, J = 9.5, 2.4 Hz, 1H), 5.13 (d, J = 15.1 Hz, 1H), 3.42 (ddd, J = 10.8, 7.8, 2.8 Hz, 1H), 3.16 (ddt, J = 15.1, 7.8, 2.8 Hz, 1H), 3.03 (dd, J = 18.4, 11.0 Hz, 1H), 2.81 (dd, J = 18.4, 2.8 Hz, 1H), 2.43 (tt, J = 11.4, 3.4 Hz, 1H), 1.95-1.85 (m, 2H), 1.85-1.77 (m, 2H), 1.73-1.66 (m, 1H), 1.47-1.35 (m, 2H), 1.35-1.16 (m, 3H);

^{13}C NMR (126 MHz, CDCl$_3$) δ 209.8, 179.1, 133.9, 132.0, 130.9, 128.2, 127.9, 127.0, 124.6, 121.8, 80.1, 50.9, 44.3, 38.4, 36.4, 28.7, 28.5, 25.7, 25.5, 25.4;

HRMS (ESI) m/z: $[M+H]^+$ calculated for $C_{20}H_{23}O_3^+$: 311.1642, found: 311.1647;

HPLC analysis: Daicel CHIRALCEL® OD-H, *n*-hexane/*i*-PrOH = 90/10, flow rate = 1.0 mL/min, λ = 254 nm, retention time: t_{major} = 11.3 min, t_{minor} = 12.6 min;

$[\alpha]_D^{25}$ = +98 (c = 0.1, CHCl$_3$).

4. Mechanism studies

4.1 Discovery and isolation of intermediates

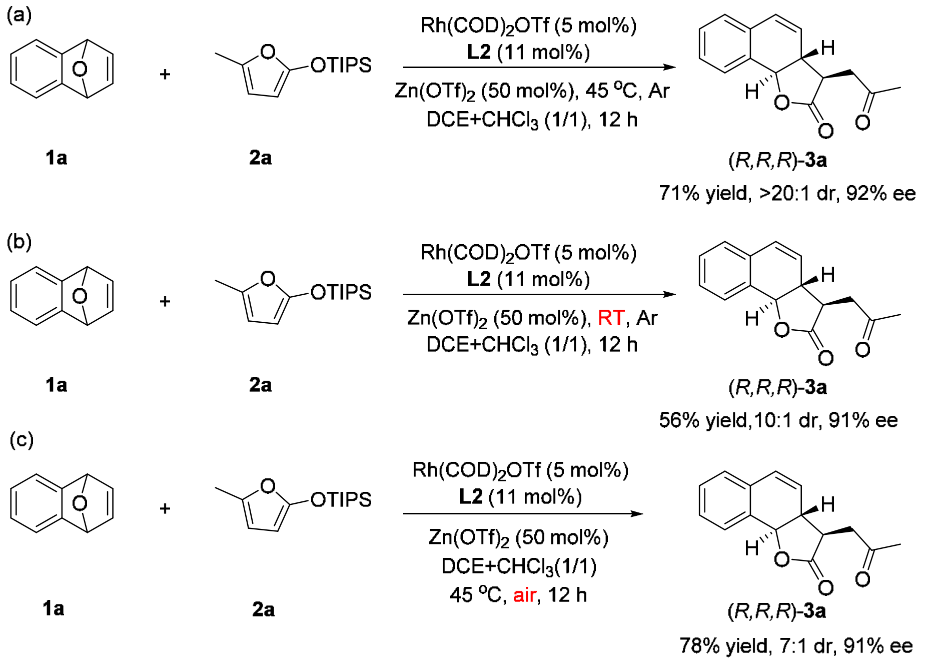

Scheme S1. An unusual decrease of disastereoselectivity for (*R,R,R*)-**3a**

In the cooperative Rh(I)/Zn(OTf)$_2$-catalysis, we observed the erosion of diastereomeric ratio, when the reaction was exposed to the atmosphere of air or at room temperature (Scheme S1). During these two processes, a non-isolatable transient adduct would be initially detected and finally converted to the product **3a** or **4a**. We

suspected that the detected transient adduct is either **6** or its and a certain Cα-racemization of the lactone motif in the transient adduct occurred with the assistance of water or at a lower temperature. Therefore, the reaction was then conducted in the air at room temperature, and we detected that **6** can be produced in up to 89% nmr yield with 11:1 dr in the mixture of reaction before purification (Scheme S2). Upon flash chromatography for isolation, a variable dr of **6 & 7** ranging from 2:1 to 3:1 was obtained in 89% yield, affirming that **6** is readily prone to Cα-racemization.

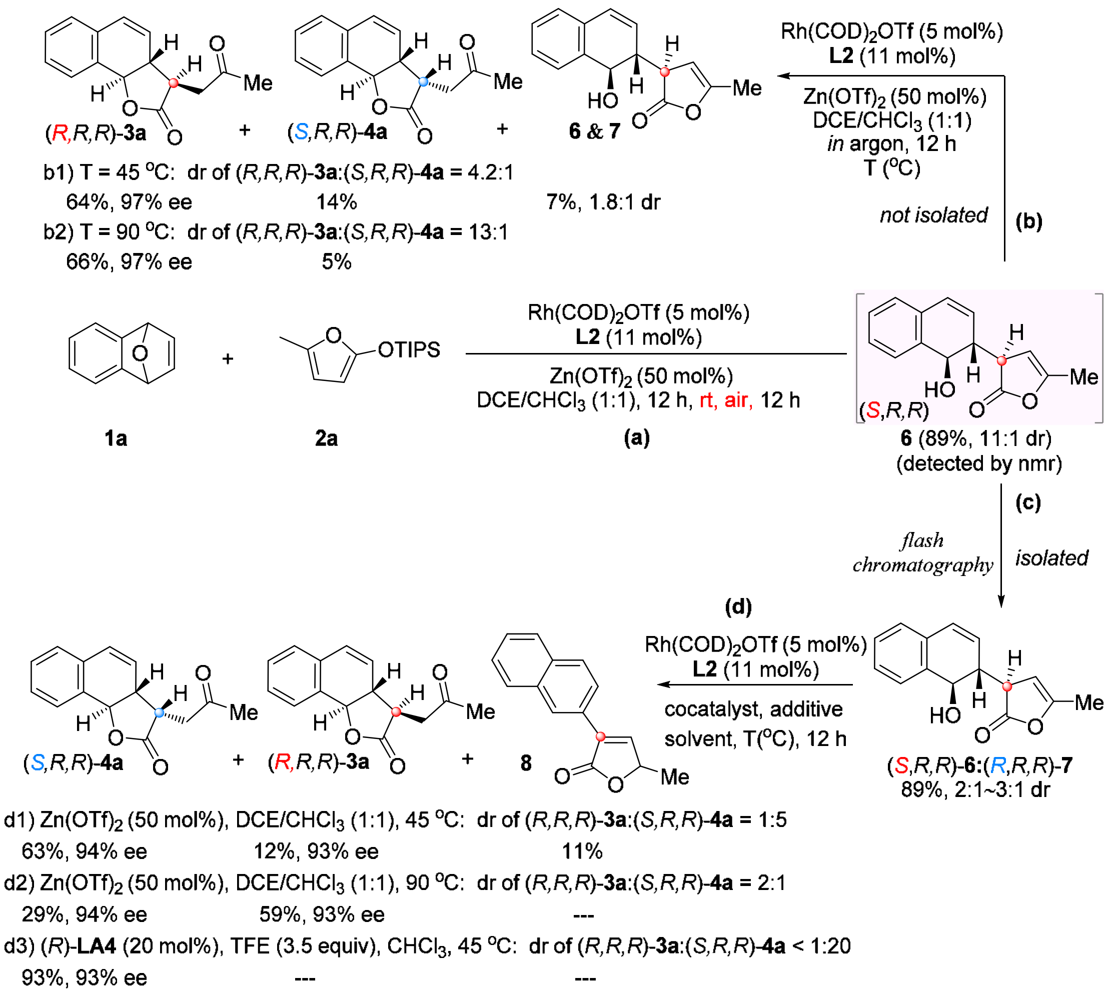

Scheme S2. Intramolecular cyclizations of **6**

Scheme S2-a: Under an air atmosphere, Rh(COD)$_2$OTf (4.68 mg, 5.0 mol%) and **L2** (20.6 mg, 11 mol%) were dissolved in the mixture solvents of DCE (2.0 mL) and $CHCl_3$ (2.0 mL) and the resulting solution was stirred for 30 min at RT. Subsequently, $Zn(OTf)_2$ (36.4 mg, 50 mol%), **1a** (28.8 mg, 0.2 mmol) and **2a** (256 mg, 1.0 mmol) were added. The reaction mixture was stirred at RT under air for 12 h. The reaction mixture was directly determined by ^{1}H NMR spectroscopy, giving **6** in 89% nmr yield with 11:1 dr, when 1,4-benzodioxan was used as the internal standard.

Scheme S2-b: The reaction mixture from *Scheme S2-a* was further stirred and heated at 45 °C or 90 °C for 12 h. After reaction completion, the solvent was evaporated under reduced pressure. The residue was purified by flash column chromatography on silica gel (petroleum ether/ ethyl acetate = 10:1) to afford the product. At 45 °C, the reaction gave the mixture diastereomers of (*R,R,R*)-**3a** and (*S,R,R*)-**4a** with 4.2:1, along with the rest of **6 & 7** in the yield of 7% with 1.8:1 dr (**6:7**) (Scheme S2-b1). At 90 °C, the reaction gave the mixture diastereomers of (*R,R,R*)-**3a** and *(S,R,R)*-**4a** with 13:1, which is almost consistent with the dr of non-isolatable **6** (11:1 dr) (Scheme S2-b2).

Scheme S2-c: The reaction mixture from *Scheme S2-a* was purified by quickly flash column chromatography on silica gel (petroleum ether/ ethyl acetate = 6:1), affording the **6 & 7** as a white liquid (21.5 mg, 89% yield, 2:1 to 3:1 dr).

Scheme S2-d: Under an argon atmosphere, $Rh(COD)_2OTf$ (2.34 mg, 5.0 mol%) and **L2** (10.3 mg, 11 mol%) were dissolved in indicated (additives and) solvent, and the resulting solution was stirred for 30 min at RT. Subsequently, the corresponding cocatalyst and **6 & 7 (**0.1 mmol, 24.2 mg) were added. The reaction mixture was stirred at either 45 °C or 90 °C for 12 h. After reaction completion, the solvent was evaporated under reduced pressure. The residue was purified by flash column chromatography on silica gel (petroleum ether/ ethyl acetate = 10:1) to afford the product. As the Scheme S2-d1 shown, the reaction was conducted with the cocatalyst of $Zn(OTf)_2$ (18.2 mg, 50 mol%) in the mixture solvents of DCE (1.0 mL) and $CHCl_3$ (1.0 mL) at 45 °C. That gave the mixture diastereomers of (*R,R,R*)-**3a** and (*S,R,R*)-**4a** with the dr value of 1:5, in which (*S,R,R*)-**4a** is the major diastereomer. When the reaction was conducted at a higher temperature of 90 °C (Scheme S2-d2), the reaction gave the mixture diastereomers of (*R,R,R*)-**3a** and (*S,R,R*)-**4a** with the dr value of 2:1. The value is closed to that of *isolated* **6** and (*R,R,R*)-**3a** is the major diastereomer.

Scheme S2-d3: Under an argon atmosphere, in a reaction tube, $Rh(COD)_2OTf$ (2.34 mg, 5.0 mol%) and **L2** (10.3 mg, 11 mol%) were dissolved in $CHCl_3$ (1.0 mL), and the resulting solution was stirred for 30 min at RT. Meanwhile, in another reaction tube, (*R*)-BINOL (6.84 mg, 24 mol%), $Sn(OTf)_2$ (8.34 mg, 20 mol%), NMM (4.9 mg, 48 mol%) were dissolved in $CHCl_3$ (1.0 mL) and stirred for 30 min at RT, to give the solution of cocatalyst (*R*)-**LA4**. Then, the solution of cocatalyst (*R*)-**LA4** in $CHCl_3$

were added to the first reaction tube. Subsequently, TFE (35 mg, 3.5 eq), **6** (0.1 mmol, 24.2 mg) were added. The reaction mixture was stirred at 45 °C for 12 h. After reaction completion, the solvent was evaporated under reduced pressure. The residue was purified by flash column chromatography on silica gel (petroleum ether/ ethyl acetate = 10:1) to afford the product (*S,R,R*)-**4a** as the single stereoisomer in 93% yield with 93% ee.

4.2 Detection of the reaction processes

4.2.1 Detection of the reaction processes by ^{1}H-NMR

Table S9. ^{1}H-NMR detection of the reaction process (General Procedure A) for (*R,R,R*)-**3a** [a]

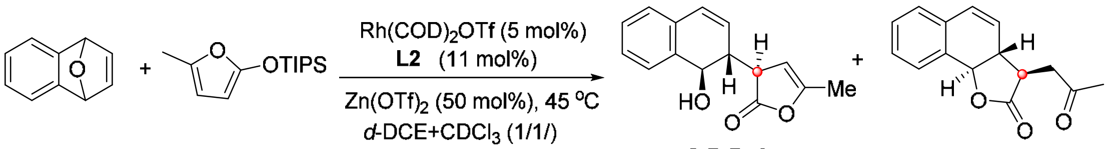

entry	time (min)	(*S,R,R*)-**6** yield(%)[b]	dr[c]	**3a+4a** yield(%)[b]	dr[d]	**1a** (%)[b]	**2a** (%)[b]
1	5	33	>20:1	-	-	67	60
2	10	31	>20:1	11	>20:1	58	59
3	20	25	>20:1	22	>20:1	53	54
4	30	28	>20:1	28	>20:1	44	52
5	40	9	>20:1	45	>20:1	33	49
6	50	6	>20:1	55	>20:1	25	47
7	60	-	-	63	>20:1	18	46
8	90	-	-	65	>20:1	13	45
9	120	-	-	70	>20:1	-	45
10	180	-	-	71	>20:1	-	44

[a] As the *General Procedure A* shown: **1a** (0.1 mmol), **2a** (0.5 mmol), $Zn(OTf)_2$ (50 mol%), $Rh(COD)_2OTf$ (5 mol%) and **L2** (11 mol%) in the mixture solvent of *d*-DCE (1.0 mL) and $CDCl_3$ (1.0 mL) react at 45 °C for stirring. At the indicated time, the reaction was monitored after quenching with trace H_2O. Then, 1,4-benzodioxan as the internal standard was added to the

quenched mixture for the further detection by ^{1}H-NMR. [b] NMR yield, 1,4-benzodioxan as the internal standard, was determined by ^{1}H NMR spectroscopy. [c] Dr refers to the ratio of (*S,R,R*)-**6** and (*R,R,R*)-**7**, determined by ^{1}H NMR. [d] The dr value was determined by ^{1}H-NMR spectroscopy and show **3a**/**4a**.

4.2.2 HRMS (ESI) analysis of reaction solution

Under the General Procedure A, the reaction mixture was stirred for 30 min, then the reaction system was analyzed by high resolution mass spectrometry.

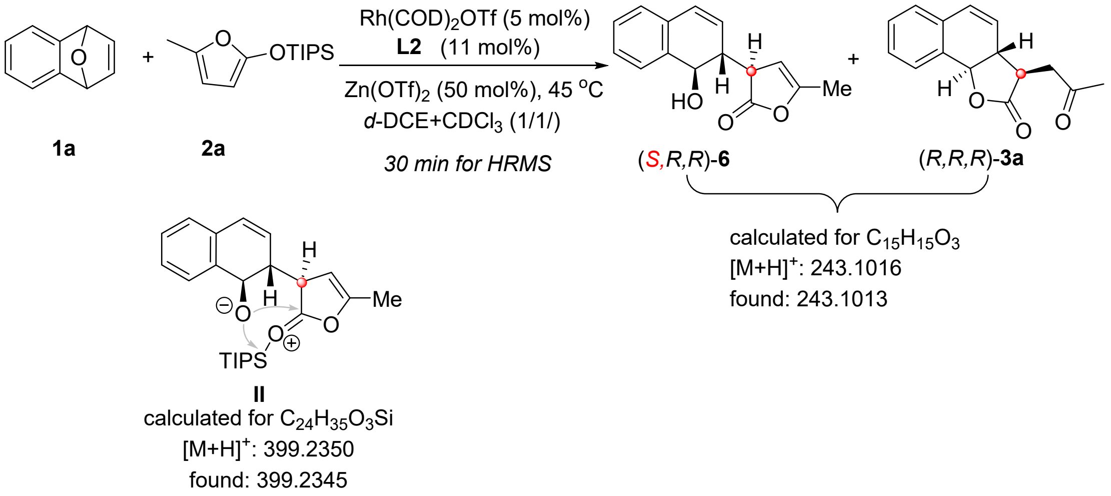

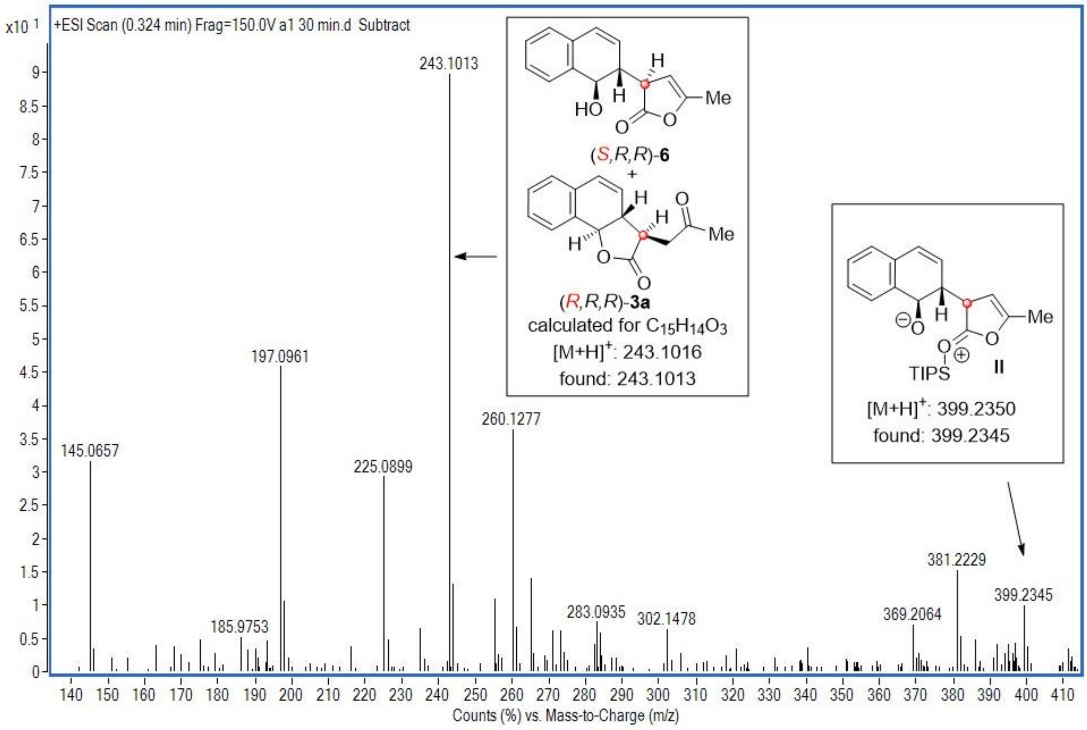

Figure S2. HRMS analysis for general procedure A.

Table S10. ^{1}H-NMR detection of the reaction process (General Procedure B) for (*S*,*R*,*R*)-**4a** [a]

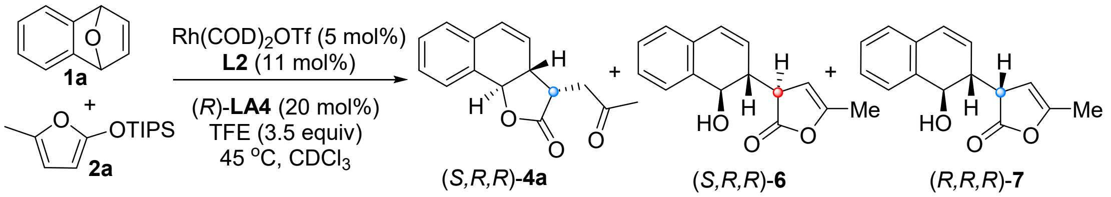

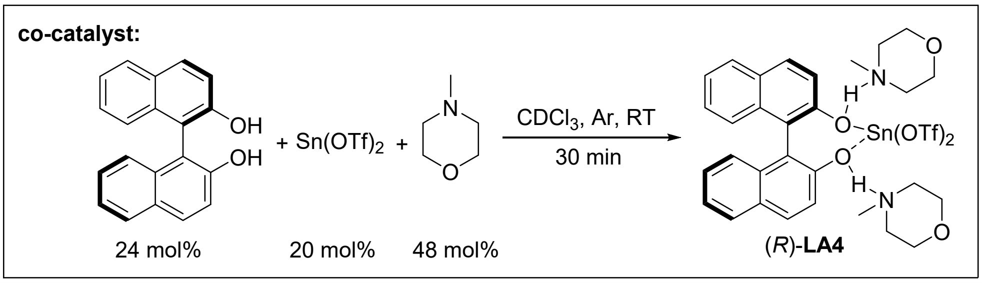

entry	time (min)	6 & 7		3a+4a		1a (%)[b]	2a (%)[b]
		yield(%)[b]	dr[c]	yield(%)[b]	dr[d]		
1	10	15	>20:1	-	-	78	93
2	20	31	9:1	-	-	56	90
3	30	37	5:1	-	-	48	86
4	60	70	2.5:1	trace	-	-	78
5	120	58	1.4:1	19	<1:20	-	74
6	180	45	1.5:1	32	<1:20	-	74
7	240	38	1.3:1	39	<1:20	-	73
8	300	28	1.3:1	49	<1:20	-	73
9	360	20	1:1	57	<1:20	-	73
10	420	12	1:1	65	<1:20	-	73
11	480	6	1:1	70	<1:20	-	73
12	540	trace	-	72	<1:20	-	73
13	600	trace	-	73	<1:20	-	73
14	660	trace	-	74	<1:20	-	73
15	720	-	-	76	<1:20	-	73

[a] General reaction conditions: **1a** (0.1 mmol), **2a** (0.5 mmol), Rh(COD)$_2$OTf (5 mol%), **L2** (11 mol%), cocatalyst (*R*)-**LA4** (20 mol% Lewis acid salt in $CDCl_3$) and TFE (3.5 equiv) in $CDCl_3$ (1.0 mL) under argon atmosphere at 45 °C. The solution of cocatalyst was prepared freshly in a separate reaction tube with the Sn(OTf)$_2$ (20 mol%), (*R*)-BINOL (24 mol%) and NMM (48 mol%) in $CDCl_3$ (1.0 mL) at RT for 30 min. [b] NMR yield, 1,4-benzodioxan as the internal standard, determined by ^{1}H NMR spectroscopy. [c] Dr of **6 & 7** refers to the ratio of (*S*,*R*,*R*)-**6** and (*R*,*R*,*R*)-**7**, determined by ^{1}H NMR. [d] The dr value was determined by ^{1}H-NMR spectroscopy and show **3a**/**4a**.

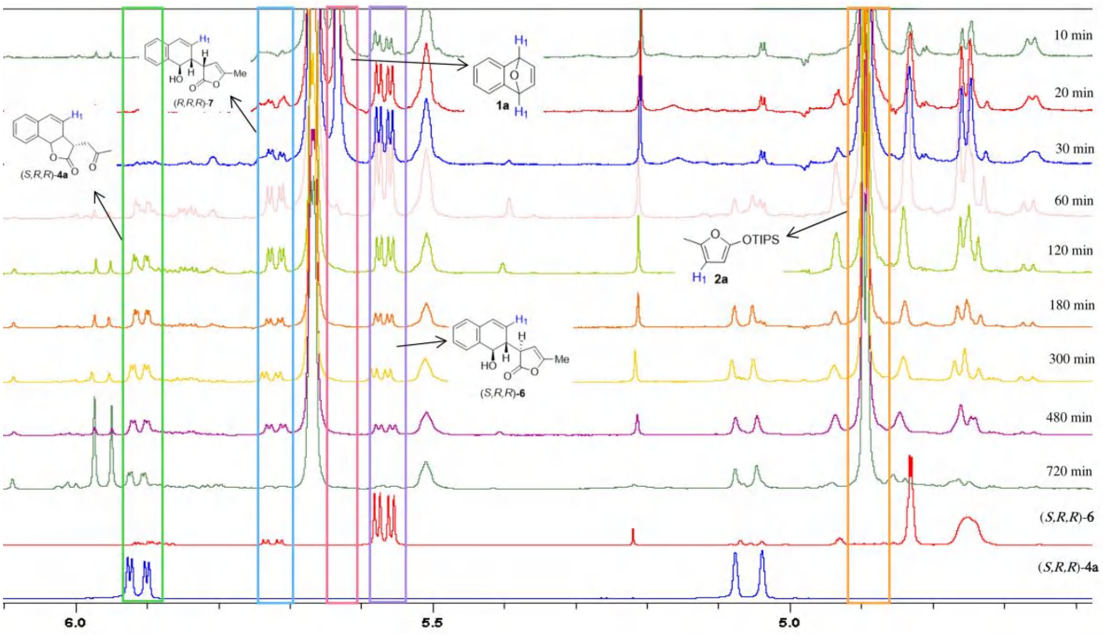

Figure S3. Comparison of ^{1}H-NMR spectra at different time points in general procedure B.

4.3 Deuterium-labeled experiments

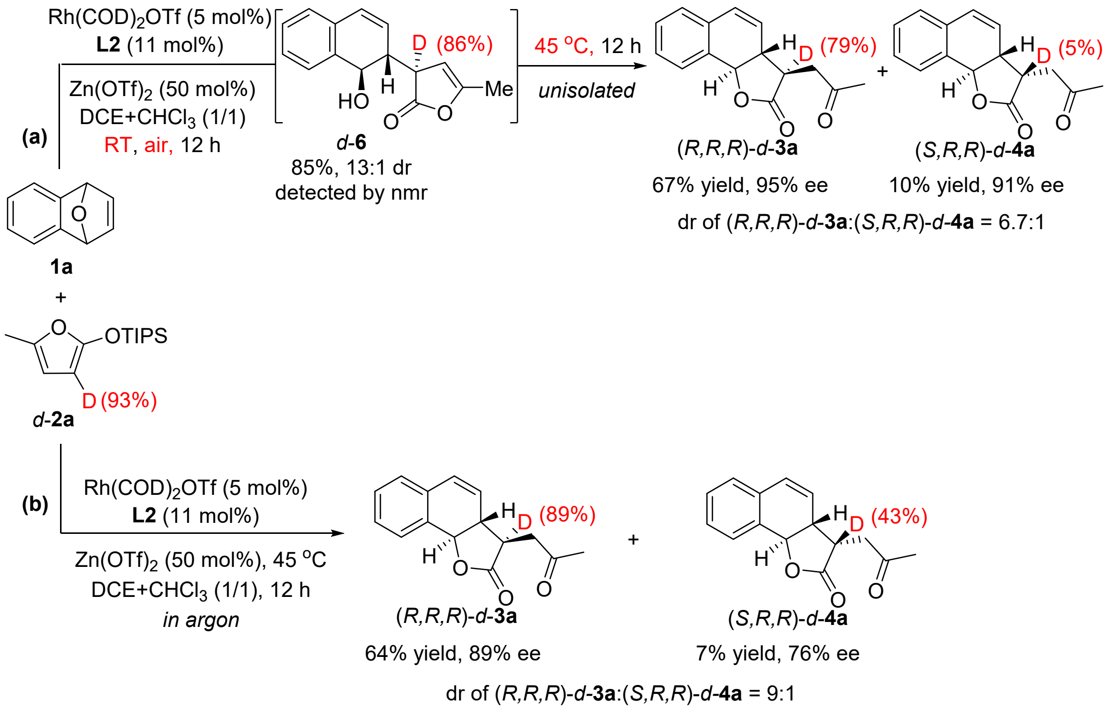

Path (a): Under an argon atmosphere, Rh(COD)$_2$OTf (2.34 mg, 5.0 mol%) and **L2** (10.3 mg, 11 mol%) were dissolved in the mixture solvent of DCE (1.0 mL) and $CHCl_3$ (1.0 mL), and the resulting solution was stirred for 30 min at RT. Subsequently, Zn(OTf)$_2$ (18.2 mg, 50 mol%), **1a** (14.4 mg, 0.2 mmol) and *d*-**2a** (128 mg, 0.5 mmol)

were added. The reaction mixture was stirred at RT under air for 12 h. The reaction mixture was directly determined by ^{1}H NMR spectroscopy, giving *d*-**6** in 89% yield with 13:1 dr, when 1,4-benzodioxan was used as the internal standard. Reaction mixture was further stirred and heated at 45 °C for 12 h. After reaction completion, the solvent was evaporated under reduced pressure. The residue was purified by flash column chromatography on silica gel (petroleum ether/ ethyl acetate = 10:1) to afford the product.

Path (b): Under an argon atmosphere, $Rh(COD)_2OTf$ (2.34 mg, 5.0 mol%) and **L2** (10.3 mg, 11 mol%) were dissolved in the mixture solvent of DCE (1.0 mL) and $CHCl_3$ (1.0 mL), and the resulting solution was stirred for 30 min at RT. Subsequently, $Zn(OTf)_2$ (18.2 mg, 50 mol%), **1a** (14.1 mg, 0.1 mmol) and *d*-**2a** (128 mg, 0.5 mmol) were added. The reaction mixture was stirred at 45 °C for 12 h. After reaction completion, the solvent was evaporated under reduced pressure. The residue was purified by flash column chromatography on silica gel (petroleum ether/ ethyl acetate = 10:1) to afford the product.

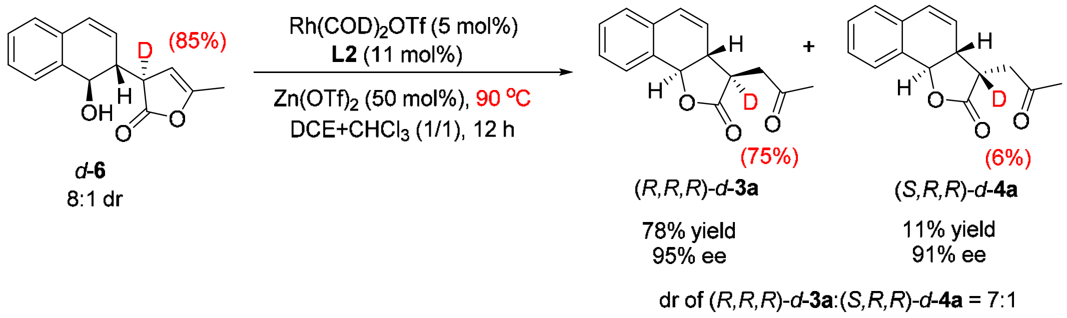

Under an argon atmosphere, $Rh(COD)_2OTf$ (2.34 mg, 5.0 mol%) and **L2** (10.3 mg, 11 mol%) were dissolved in the mixture solvent of DCE (1.0 mL) and $CHCl_3$ (1.0 mL), and the resulting solution was stirred for 30 min at RT. Subsequently, $Zn(OTf)_2$ (18.2 mg, 50 mol%), *d*-**6** (0.1 mmol, 24.2 mg, 8:1 dr) were added. The reaction mixture was stirred at 90 °C for 12 h. After reaction completion, the solvent was evaporated under reduced pressure. The residue was purified by flash column chromatography on silica gel (petroleum ether/ ethyl acetate = 10:1) to afford the product.

4.4 Linear effect study

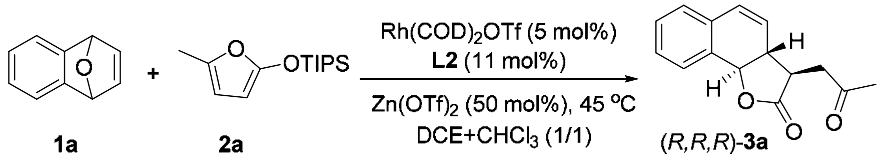

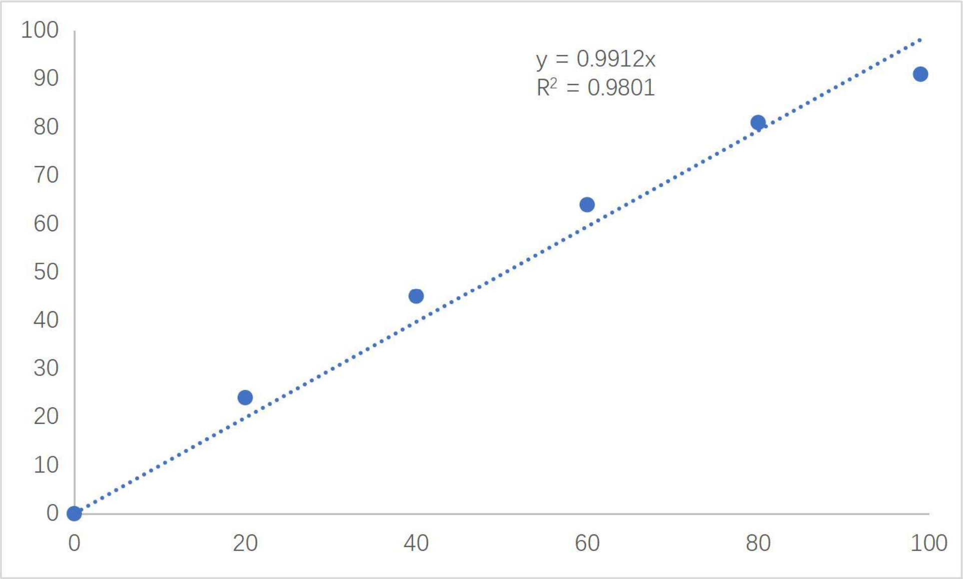

**L2** (ee,%)	0	20	40	60	80	99
(*R*,*R*,*R*)-**3a** (ee,%)	0	24	45	64	81	91

Under an argon atmosphere, Rh(COD)$_2$OTf (2.34 mg, 5.0 mol%) and enantiomeric composition of **L2** (10.3 mg, 11 mol%) were dissolved in the mixture solvents of DCE (1.0 mL) and $CHCl_3$ (1.0 mL) and the resulting solution was stirred for 30 min at RT. Subsequently, Zn(OTf)$_2$ (18.2 mg, 50 mol%), **1a** (0.1 mmol) and **2a** (0.5 mmol) were added. The reaction mixture was stirred under argon atmosphere at 45 °C for 12 h. After reaction completion, the solvent was evaporated under reduced pressure. The residue was purified by flash column chromatography on silica gel (petroleum ether/ ethyl acetate = 10:1) to afford (*R*,*R*,*R*)-**3a**.

4.5 Thermal stability investigation

4.5.1 Thermal stability investigation for (*R*,*R*,*R*)-3a and (*S*,*R*,*R*)-4a under standard conditions

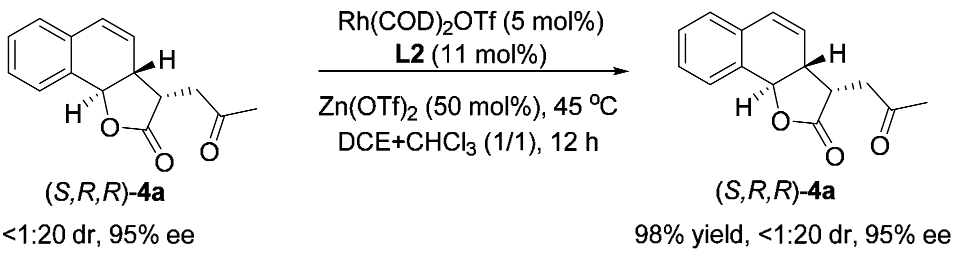

Under an argon atmosphere, $Rh(COD)_2OTf$ (2.34 mg, 5.0 mol%) and **L2** (10.3 mg, 11 mol%) were dissolved in DCE+$CHCl_3$ (1.0+1.0 mL) and the resulting solution was stirred for 30 min at RT. Subsequently, $Zn(OTf)_2$ (18.2 mg, 50 mol%), (*S,R,R*)-**4a** (24.2 mg, 0.1 mmol) were added. The reaction mixture was stirred at 45 °C for 12 h. After reaction completion, the solvent was evaporated under reduced pressure. The residue was purified by flash column chromatography on silica gel (petroleum ether/ ethyl acetate = 10:1) to afford the product (white solid, 23.7 mg, 98% yield, <1:20 dr, 95% ee).

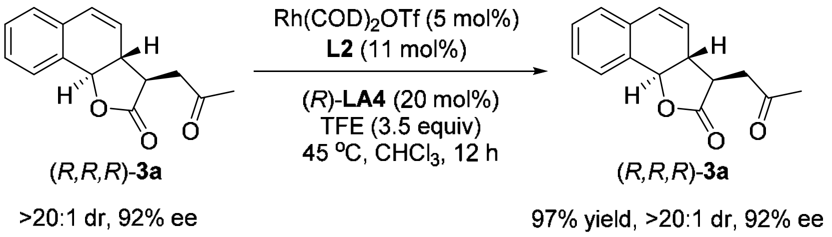

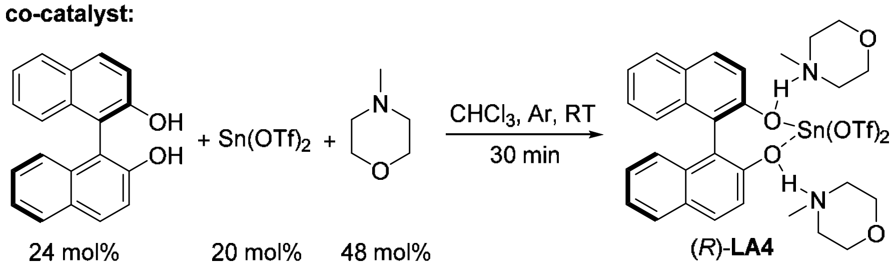

In a reaction tube, $Rh(COD)_2OTf$ (2.34 mg, 5.0 mol%) and **L2** (10.3 mg, 11 mol%) were dissolved in $CHCl_3$ (1.0 mL) and the resulting solution was stirred for 30 min at RT. In another reaction tube, (*R*)-BINOL (6.84 mg, 24 mol%), $Sn(OTf)_2$ (8.34 mg, 20 mol%), NMM (4.9 mg, 48 mol%) were dissolved in $CHCl_3$ (1.0 mL) and stirred for 30 min at RT, to give the solution of cocatalyst (*R*)-**LA4**. Then, the solution of cocatalyst (*R*)-**LA4** in $CHCl_3$ were added to the first reaction tube. Subsequently, TFE (35 mg, 3.5 equiv) and (*R,R,R*)-**3a** were added. The reaction mixture was stirred at 45 °C for 12 h. After reaction completion, the solvent was evaporated under reduced pressure. The residue was purified by flash column chromatography on silica

gel (petroleum ether/ ethyl acetate = 10:1) to afford the product (white solid, 23.7 mg, 98% yield, >20:1 dr, 95% ee).

4.5.2 Base-assisted Cα-epimerization between (*R,R,R*)-3a and (*S,R,R*)-4a

Table S11. Base-assisted Cα-epimerization of (*S,R,R*)-**4a** [a]

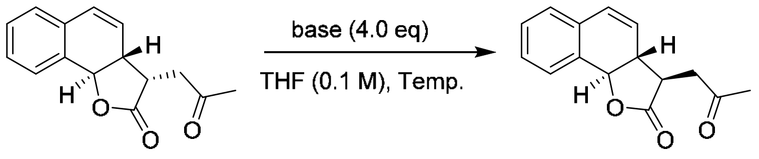

entry	base	temp. (°C)	time (h)	Product of **3a**+**4a** yield (%)[b]	dr[c]	ee (%)[d]
1	LiHMDS	-78	5	>99	<1:20	-/96
2	LiHMDS	RT	12	88	1:4	96/96
3	LiHMDS	70	6	48	1:1.3	96/96
4	LiHMDS	70	24	45	1:1.3	96/96
5	LiHMDS	90	24	43	15:1	97/96
6	LDA	RT	12	decomposed	-	-
7	KHMDS	70	24	decomposed	-	-
8	DBU	70	24	decomposed	-	-

[a] General reaction conditions: (*S,R,R*)-**4a** is dissolved in THF and subsequently added base under argon atmosphere. [b] Yield of isolated product refers to the total yield **3a**+**4a**. [c] The dr value was determined by ^{1}H-NMR spectroscopy and show **3a**/**4a**. [d] The ee value of **3a** or **4a** was determined by HPLC analysis on a chiral stationary phase and show **3a**/**4a**.

Table S12. Base-assisted Cα-epimerization of (*R,R,R*)-**3a** [a]

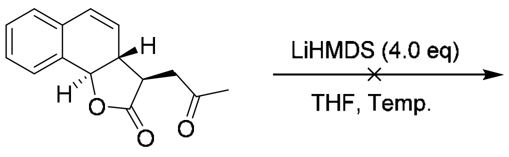

entry	base	temp. (°C)	time (h)	Product of **3a**+**4a** yield (%)[b]	dr[c]	ee (%)[d]
1	LiHMDS	-78	5	100	>20:1	92/-
2	LiHMDS	RT	12	98	>20:1	92/-
3	LiHMDS	70	6	90	>20:1	92/-
4	LiHMDS	90	24	85	>20:1	92/-

[a] General reaction conditions: (*R,R,R*)-**3a** is dissolved in THF and subsequently added LiHMDS under argon atmosphere. [b] Yield of isolated product refers to the total yield **3a**+**4a**. [c] The dr value was determined by ^{1}H-NMR spectroscopy and show **3a**/**4a**. [d] The ee value of **3a** or **4a** was determined by HPLC analysis on a chiral stationary phase and show **3a**/**4a**.

4.6 Computational details

All of the calculations were performed using the Gaussian 16 program.[3] Structures were optimized at the (U)B3LYP level of density functional theory[4] with Grimme's D3(BJ) dispersion correction[5] in gas phase. For optimizations, Ahlrichs's def2SVP basis set was used for all atoms.[6] Frequency calculations have been performed to verify the optimized structures as local minima or transition state and to obtain Gibbs free energy at 298 K. Intrinsic reaction coordinate (IRC) calculations were carried out to make sure that every transition state links relevant intermediates.[7] The electronic energies were further refined by carrying out single-point energy calculations using (U)B3LYP functional with Grimme's D3(BJ) dispersion correction. The def2TZVP basis set was applied for all atoms.[6] The SMD solvation model with chloroform as the solvent was employed to account for solvation effect.[8-10]

Additional computational results

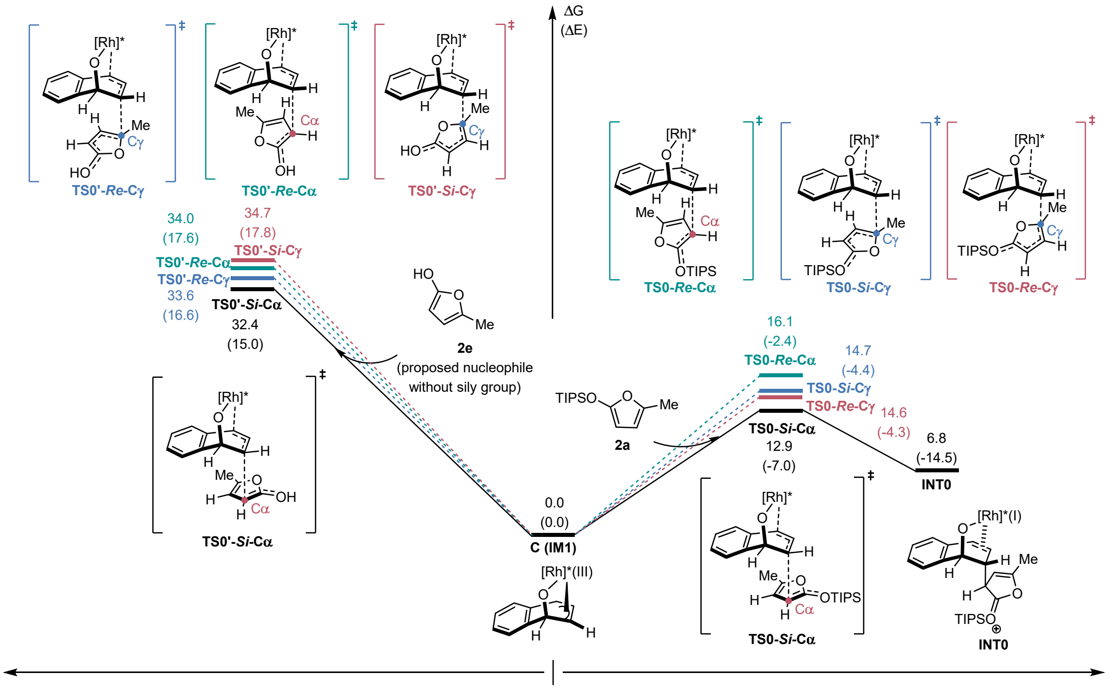

Figure S4. Calculated energy profiles for nucleophilic attack of **2a** or **2e** at **C** at the B3LYP-D3(BJ)/PCM(chloroform)/ def2TZVP level of theory.

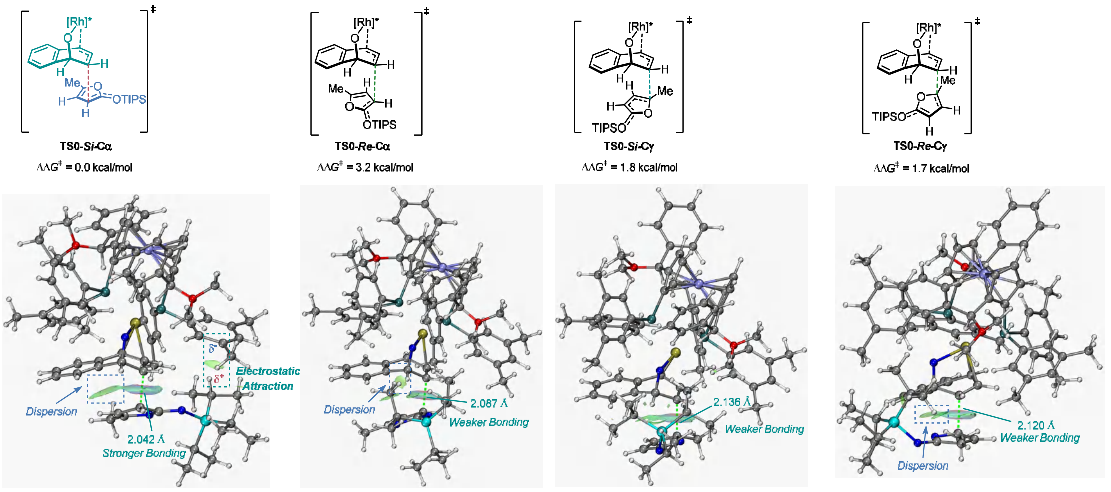

Figure S5. IGMH analysis for stereo-determining and regio-determining transition states in the nucleophilic attack process. Stronger non-covalent interaction between catalyst and **2a** was discovered in the most favorable transition state **TS0-*Si*-Cα**. The calculations were performed at the B3LYP-D3(BJ)/PCM/def2TZVP level of theory.

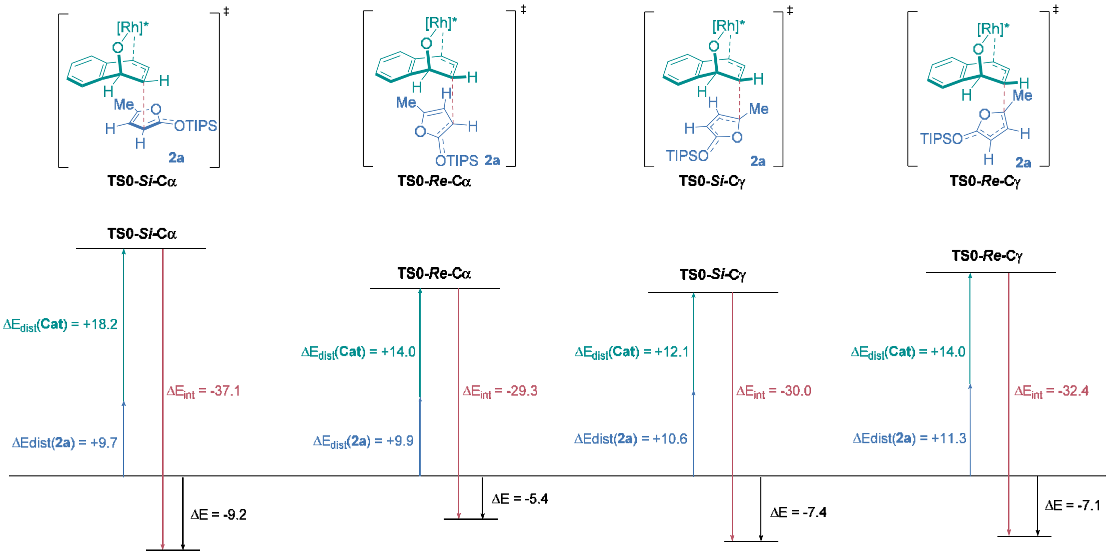

Figure S6. Distortion/interaction (DI) analysis on stereo-determining and regio-determining transition states in the nucleophilic attack process indicate that the interactions between catalyst and substrate **2a** dominates the energy difference among different TSs. The DI analysis was performed based on the calculations at the B3LYP-D3(BJ)/def2TZVP level of theory. Energies are given in kcal/mol.

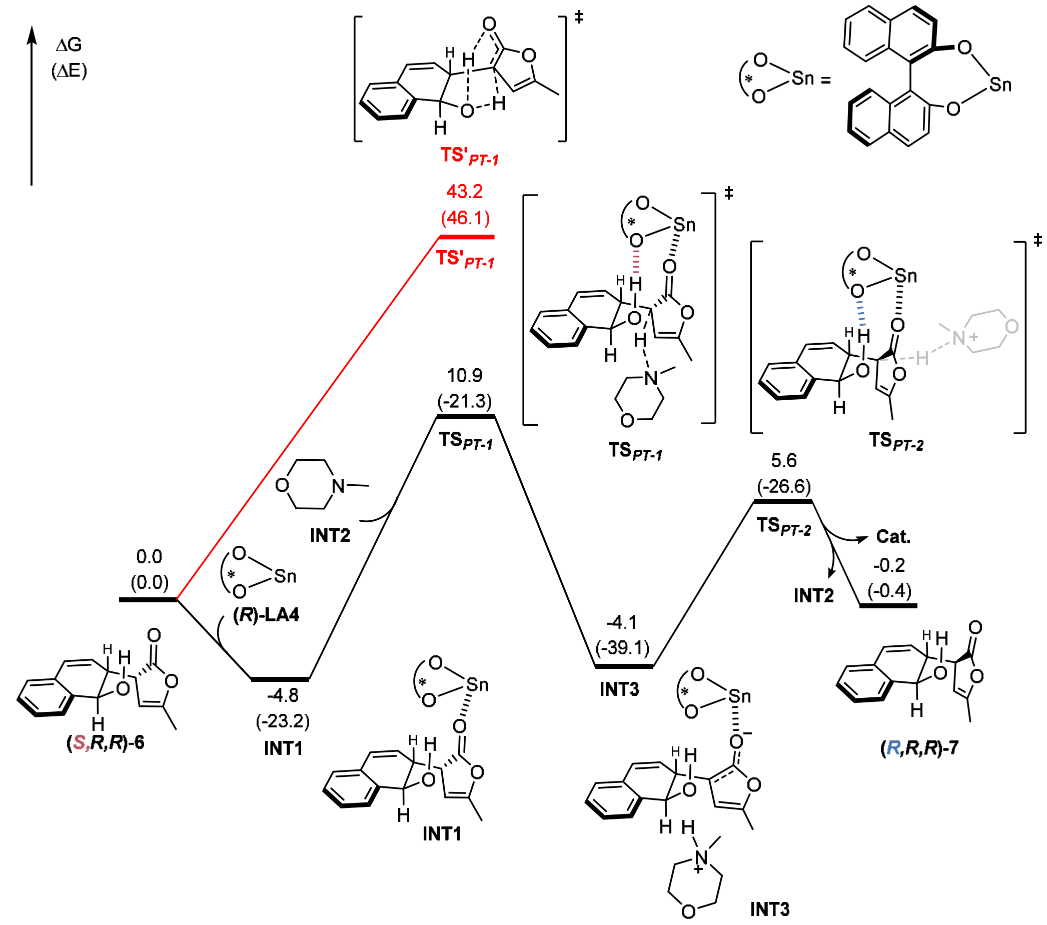

Figure S7. Calculated energy profiles on the isomerization. All energies are given in kcal/mol.

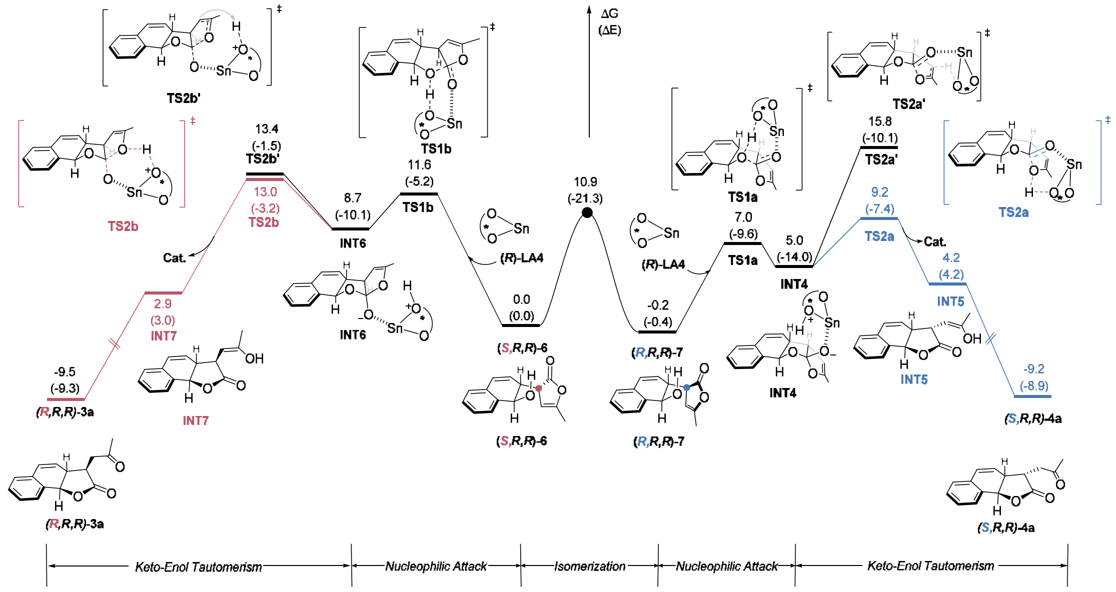

Figure S8. Computational studies for the diastereodivergent synthesis. All energies are given in kcal/mol.

Density functional theory (DFT) calculations validate the proposed mechanism of the epimerization/lactonization process ((*S*,*R*,*R*)-**6**→(*R*,*R*,*R*)-**7**→(*S*,*R*,*R*)-**4a**),

thereby elucidating the observed diastereoselective reversal. DFT results confirm that the combined catalyst (*R*)-**LA4** acts as a multifunctional catalytic system, integrating the capabilities of both a Lewis acid and a proton-transfer catalyst across the process. It facilitates epimerization via the key transition state **TSPT-1** ((*S,R,R*)-**6** to (*R,R,R*)-**7**) and lactonization via **TS1a** and **TS2a**, ultimately yielding the product (*S,R,R*)-**4a**. Computational data indicate that these two diastereomers **6** and **7** are thermodynamically similar, which is consistent with the experimental observation that the epimerization from **6** to **7** occurs relatively easily while maintaining a consistently low dr. Conversely, the pathway from **6** to (*R,R,R*)-**3a** was calculated to be disfavored in energy. Additionally, both non-covalent interaction (NCI) analysis and distortion/interaction analysis of the highest energy transition states reveal that a significantly stronger interactions between the substrate and catalyst (*R*)-**LA4** presents in the favored transition state **TS2a**, compared to **TS2b**.

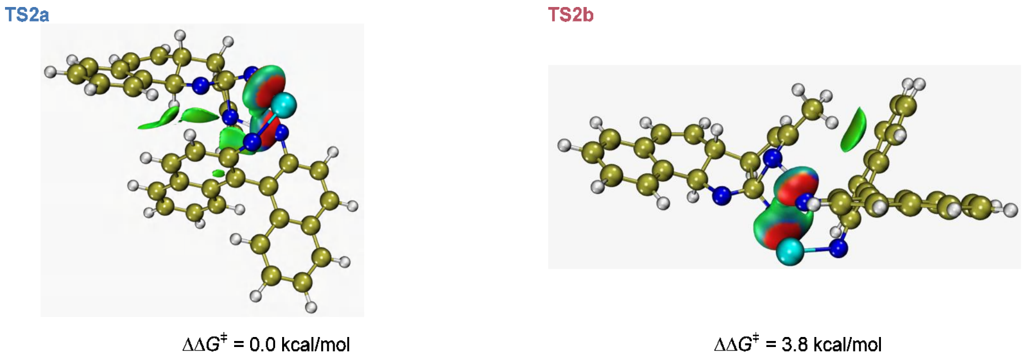

Figure S9. IGM analysis for enantio-determining transition states reveals that the prominent lone-pair-π interaction between oxygen and naphthalene sorely exists in **TS2a**.

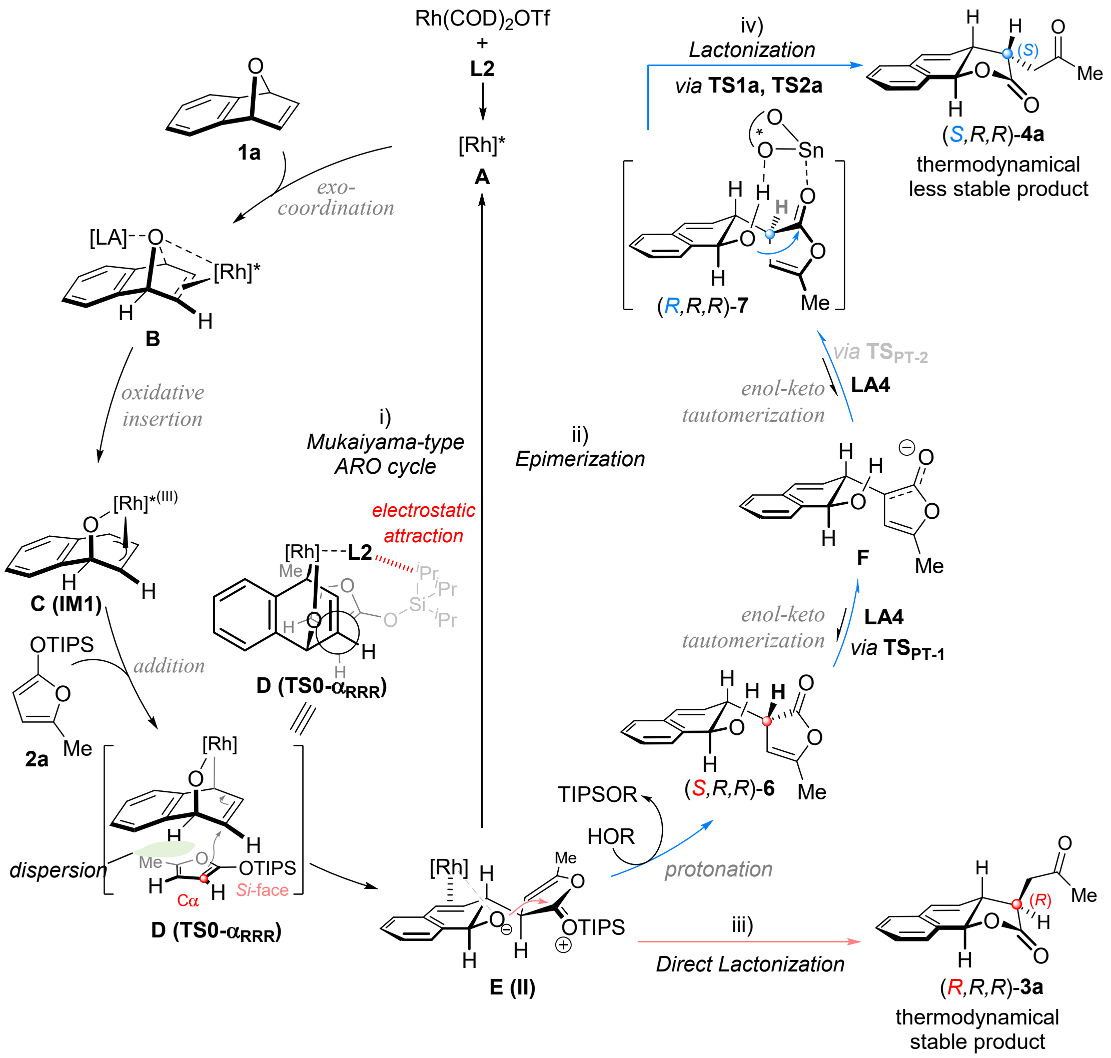

Figure S10. Possible reaction mechanism.

XYZ coordinate

2a

E = -989.527574588 G = -989.199814

6	1.59176500	-0.89229700	-0.26559000
6	2.22948400	-1.56876900	0.73800800
6	3.57849700	-1.06279000	0.75269100
6	3.66493400	-0.12122400	0.23314200
8	2.44693600	-0.01579500	-0.86234700
1	1.78737700	-2.32784100	1.37729000
1	4.38617900	-1.36658800	1.41559800
8	0.35107300	-0.91877200	-0.73721900
6	4.75779000	0.74990900	-0.73595900
1	4.49452200	1.81853100	-0.65675900
1	5.67111100	0.57658000	-0.14993700
1	4.98439700	0.54602000	-1.79649100
14	-0.94278300	0.06946900	-0.16917600
6	-2.24896400	-0.12755500	-1.52863700
1	-1.94280900	0.60658200	-2.29890000
6	-0.27106300	1.84402200	-0.06896700
1	0.36041900	1.91916100	-0.97423500
6	-1.39263200	-0.63429600	1.53270600

1	-0.42751200	-0.59622000	2.07352300
6	-3.66665300	0.24283100	-1.06262600
1	-4.37039100	0.24058800	-1.91235600
1	-4.04664600	-0.48534200	-0.32810900
1	-3.71655700	1.23889600	-0.59648000
6	-2.22741800	-1.51838800	-2.18314500
1	-2.93098100	-1.55808800	-3.03252100
1	-1.22529400	-1.77845800	-2.55177800
1	-2.53060000	-2.30250600	-1.47092300
6	0.63893500	2.07392600	1.14931100
1	1.44646800	1.33044600	1.22028000
1	1.11044200	3.07050600	1.09979700
1	0.06866500	2.03315200	2.09154900
6	-1.35800300	2.92669900	-0.16299400
1	-1.95609200	2.83871300	-1.08310600
1	-2.05399600	2.88524600	0.69106900
1	-0.90611200	3.93356500	-0.16072400
6	-2.40263800	0.20909400	2.32581100
1	-2.07470000	1.25331600	2.44222900
1	-3.39043300	0.22615500	1.83959000

1	-2.54745900	-0.20378400	3.33910000
6	-1.82091900	-2.10758800	1.45486500
1	-1.09848700	-2.71368500	0.88675000
1	-1.91339300	-2.54747700	2.46280200
1	-2.80191700	-2.21495300	0.96372700

C

E = -4955.28457767 G = -4954.120258

6	2.43694800	1.95356300	1.73743400
6	-1.12295400	2.19920600	1.76752000
6	-1.18682100	0.76745500	1.55666400
6	-1.06372900	0.14454700	2.84945500
6	-0.90972500	1.15644100	3.83116000
6	-0.93471600	2.41165600	3.16829600
15	-1.44682700	-0.32231700	0.10835000
15	2.04040300	0.12224600	-0.53917900
6	-2.22976300	0.58878100	-1.28899500
6	-2.84087300	-1.37065000	0.69903400
6	-3.20414000	-2.49941400	-0.04320300
6	-4.31364000	-3.27648900	0.32542100
6	-5.06345900	-2.89033400	1.43994700
6	-4.74573000	-1.74054600	2.18017300
6	-3.63294700	-0.98725500	1.79715500
6	-1.44812800	1.31779700	-2.19369800
6	-2.01949300	1.89629000	-3.33429600
6	-3.39115900	1.71703100	-3.56233700
6	-4.19533700	0.99816800	-2.67128800
6	-3.60157600	0.44145700	-1.53043900
26	0.60605300	1.20877000	2.42887400
6	2.12993900	0.66853600	1.18493400
6	2.46120200	1.83213400	3.15276500
6	1.98922100	-0.26858800	2.28273100
6	2.19923800	0.47294600	3.48831400
1	-2.62292200	-2.78514400	-0.91102800
1	-3.40431100	-0.08435000	2.35785400
1	-0.38093000	1.42985100	-2.02543100
1	-4.22350100	-0.12775500	-0.84170000
1	-0.75770600	0.98869800	4.89542000
1	-0.82498100	3.38720400	3.63408000
1	2.62268600	2.64479900	3.85834800
1	2.11913000	0.07919900	4.49679500
1	2.62701000	2.86222700	1.17587000
1	-5.92837800	-3.49156300	1.73564300
1	-3.84242400	2.14828900	-4.46012100
45	0.42179100	-1.56022800	-0.66663000
6	3.77810200	-0.45361700	-0.79135100
6	4.11246300	-1.80282700	-0.91599000
6	4.79676800	0.51183000	-0.76214300
6	5.45118200	-2.20543600	-1.02949500
1	3.33418700	-2.55840000	-0.89662300
6	6.14137600	0.14038100	-0.86939000
1	4.54581500	1.56991900	-0.66109800
6	6.44717900	-1.22271800	-1.00670600
1	7.49506000	-1.52449200	-1.09715500
6	2.02062600	1.57544200	-1.65214100
6	1.52529200	2.83028400	-1.29280000
6	2.50238200	1.37076100	-2.95850200
6	1.52387300	3.89679600	-2.21000100
1	1.12151700	2.98200600	-0.29683900
6	2.51626900	2.41186500	-3.88966600
1	2.90632700	0.39833600	-3.24730700
6	2.03144500	3.66904900	-3.49168700
1	2.03748000	4.49103900	-4.21289300
6	5.80962400	-3.66628200	-1.13895900
1	6.62946900	-3.82866800	-1.85487700
1	6.14669200	-4.06536900	-0.16704800
1	4.94993400	-4.27163300	-1.46198200
6	7.23940200	1.17294500	-0.85342500
1	7.99483500	0.93913100	-0.08641400
1	7.76463200	1.20681000	-1.82224900
1	6.84633100	2.17900500	-0.64879800
6	0.97192100	5.24393300	-1.82249900
1	1.54272400	5.69336200	-0.99569600
1	0.99372000	5.94302600	-2.66998100
1	-0.06782800	5.16410500	-1.46937600
6	3.03341500	2.20272500	-5.28908600
1	2.24118200	2.38373200	-6.03406800
1	3.85318300	2.90268200	-5.51665200
1	3.40922200	1.18020200	-5.43530900
6	-5.66345200	0.78298100	-2.93371200
1	-6.02003600	1.39120700	-3.77700700
1	-5.86457000	-0.27535700	-3.17038700
1	-6.26874200	1.03450500	-2.04860800
6	-1.17875100	2.69312200	-4.29668100
1	-0.96340000	3.69697200	-3.89495900
1	-0.20624700	2.21281800	-4.47397200
1	-1.68732600	2.82286100	-5.26254900
6	-5.58549900	-1.33480000	3.36468100
1	-5.27371800	-0.35982900	3.76625200
1	-6.65201600	-1.26900100	3.09654700
1	-5.50374000	-2.07437800	4.17857100
6	-4.67223300	-4.50157000	-0.47517900
1	-5.55110800	-5.01457900	-0.05966300
1	-4.88773000	-4.23913100	-1.52254100
1	-3.83367600	-5.21577300	-0.49394600
1	-1.07298800	-0.92166600	3.03914300
6	1.75967100	-1.75562100	2.11118800
1	1.10610300	-1.84726200	1.19071400
6	-1.44303700	3.30821200	0.78685700
1	-0.82492400	3.15859900	-0.11318200
7	-2.81301800	3.16935500	0.31883100
6	-1.05951300	4.67315700	1.37283500
6	0.29101900	5.01065600	1.54802700
6	-2.02746600	5.60105700	1.77599100
6	0.66723900	6.23548700	2.09947100
1	1.06061300	4.29352300	1.26840200
6	-1.65749700	6.83285700	2.32687100
1	-3.08394500	5.36578900	1.66393100
6	-0.30979800	7.15662900	2.48991700
1	1.72643900	6.47260500	2.22670200
1	-2.43141100	7.54131800	2.63101400
1	-0.02102200	8.11814900	2.91995200
6	-3.17736200	3.98011800	-0.81736600
1	-3.43924100	5.02908800	-0.56602000
1	-2.34634300	4.00782500	-1.53731600
1	-4.04262900	3.53080500	-1.32853900
6	-3.89716800	2.87400100	1.22571700
1	-4.65877600	2.26671600	0.70404900
1	-3.53780100	2.28895100	2.08155100
1	-4.41585500	3.76803800	1.63097100
7	2.99067000	-2.44692300	1.77674000
6	0.92626000	-2.44555300	3.18459900
6	-0.25393900	-3.10924500	2.81787100
6	1.34268400	-2.49717000	4.52322600
6	-1.03633900	-3.75505500	3.77794400
1	-0.56669800	-3.12614500	1.77079900
6	0.56349500	-3.14878900	5.48309200
1	2.28339400	-2.03600200	4.82608700
6	-0.63472400	-3.76883300	5.11612900
1	-1.96118600	-4.24941400	3.47227100
1	0.89636800	-3.17426400	6.52334400
1	-1.24395200	-4.27294000	5.86967600
6	2.82068600	-3.85970900	1.51190500
1	3.71373500	-4.24278600	0.99433000

1	2.66596000	-4.46980300	2.42422700
1	1.94614300	-4.01231700	0.86002600
6	4.20344400	-2.11512800	2.49361200
1	4.24287200	-2.52632500	3.52431600
1	5.06316600	-2.52316000	1.93970400
1	4.33621200	-1.02680600	2.54521000
6	-1.20056900	-3.61914500	-2.74265600
6	-2.26703400	-4.41205200	-3.15044100
1	-2.31882500	-5.45693600	-2.83456900
6	-3.29143700	-3.86086200	-3.93538900
1	-4.12917800	-4.48616400	-4.25218900
6	-3.24898000	-2.51291600	-4.29714200
1	-4.05298600	-2.08029000	-4.89649400
6	-2.16924000	-1.71351700	-3.90627400
1	-2.12796100	-0.66443200	-4.19964800
6	-1.13439500	-2.26505100	-3.14479800
6	0.07539900	-1.50628300	-2.77265600
6	1.30827700	-2.27284800	-2.64001500
6	1.21202300	-3.51360000	-2.06015300
6	-0.18422800	-4.03200500	-1.69475200
1	-0.17829800	-5.12706100	-1.55625900
1	0.15522800	-0.50063200	-3.18730400
1	2.09682400	-4.11585500	-1.84346600
1	2.27324700	-1.84304300	-2.91198800
8	-0.52007500	-3.38709300	-0.49390700

INT1

E = -1940.83469135 G = -1940.370066

6	-2.37058800	2.42057500	-3.15017000
6	-1.17664800	1.69026300	-3.16250500
6	-0.13994400	2.02095400	-2.29291000
6	-0.29251000	3.09252300	-1.38527300
6	-1.47530400	3.84440300	-1.41331500
6	-2.51242200	3.50932900	-2.28660200
1	-3.18602800	2.14220000	-3.82180300
1	-1.03859900	0.85487200	-3.84997100
6	0.75811200	3.33502700	-0.39241600
1	-1.58975300	4.68072600	-0.72016600
1	-3.43618700	4.09285600	-2.28554300
6	1.71686900	2.42566900	-0.14597900
1	0.68702100	4.23590800	0.22096900
1	2.42369700	2.57776800	0.67252600
6	1.20131500	1.31293800	-2.34083200
1	1.89625400	1.98085400	-2.89012600
6	1.76524300	1.13256800	-0.91723500
1	1.10753500	0.40533000	-0.40946800
8	1.18584600	0.10192400	-3.04817800
1	0.64392800	-0.52793300	-2.51520900
6	3.17702800	0.48583600	-1.02450600
1	3.18486200	-0.01072600	-2.01489900
8	4.56039300	-0.34707600	0.65993000
6	4.36533400	1.37491100	-0.80457300
1	4.56251400	2.30337900	-1.33511900
6	5.11325800	0.86325400	0.17787000
6	3.44155100	-0.61241500	-0.02445800
8	2.82520800	-1.64748100	0.17786300
6	6.36331500	1.27944700	0.85476500
1	6.19211700	1.41815400	1.93487500
1	7.14548200	0.51106200	0.74171200
1	6.72713700	2.22344600	0.42810900
6	-5.97162700	-0.84840100	1.12227600
6	-5.51666800	-1.15667000	-0.14143300
6	-4.12537600	-1.20001000	-0.42951400
6	-3.17931400	-0.89735900	0.60663300
6	-3.68564900	-0.60343300	1.90519700
6	-5.04130000	-0.57997700	2.15584200
1	-4.36290200	-1.77396800	-2.51152100
1	-7.04354300	-0.82063200	1.33191700
1	-6.22214500	-1.38158300	-0.94598800
6	-3.64353400	-1.55222600	-1.71892100
6	-1.77127700	-0.91209800	0.32162500
1	-2.98032800	-0.40104300	2.71079100
1	-5.40113000	-0.35570300	3.16301900
6	-1.34923000	-1.32727400	-0.94262100
6	-2.29451600	-1.63469700	-1.96066700
1	-1.90775100	-1.92517100	-2.93981600
6	-0.77891500	-0.46982400	1.34375000
6	-0.78940500	0.88136100	1.83772400
6	0.17521400	-1.36512000	1.83459300
6	0.16652600	1.28767800	2.82952400
6	-1.71980000	1.85643200	1.37978200
6	1.12735500	-0.94166900	2.80824300
6	1.12238900	0.34220100	3.29161900
6	0.13743200	2.61541000	3.33297800
6	-1.73627800	3.13141300	1.90384400
1	-2.42438100	1.59063500	0.59391400
1	1.85415600	-1.68003900	3.15116600
1	1.85218200	0.65284900	4.04433900
6	-0.80249600	3.52223500	2.89206100
1	0.87384700	2.90131700	4.08920000
1	-2.47083500	3.85175400	1.53747000
1	-0.82534900	4.53700300	3.29661700
8	-0.02988600	-1.45141000	-1.25242600
8	0.24894200	-2.64031700	1.40727300
50	1.03730700	-3.08295900	-0.43238100

INT2

E = -327.262288517 G = -327.130251

6	0.23668900	-1.19673400	0.60336000
6	-0.97918900	-1.17405100	-0.32700400
6	-0.97886700	1.17429100	-0.32683300
6	0.23728400	1.19677600	0.60318300
1	-0.65593500	-1.23764900	-1.38751600
1	-1.64429700	-2.02883400	-0.12778600
1	-0.12903400	-1.25802600	1.64450800
1	0.85269800	-2.09135600	0.41477400
1	-0.65599800	1.23812300	-1.38742400
1	-1.64377000	2.02913000	-0.12720400
1	0.85376300	2.09095200	0.41407000
1	-0.12790100	1.25853700	1.64447800
8	-1.74387400	0.00017000	-0.12207100
7	1.06264000	-0.00023700	0.47714700
6	1.97191000	-0.00023000	-0.65173700
1	2.62222700	0.88807100	-0.60029800
1	2.62074200	-0.88962600	-0.60125000
1	1.49304800	0.00066700	-1.65561900

INT3

E = -2268.12227208 G = -2267.499262

6	1.50197300	-0.08859000	4.36494200
6	0.44627600	-0.11212900	3.44577400
6	0.17771000	1.00003300	2.64953400
6	0.98559200	2.15343800	2.75402400
6	2.01737900	2.17845000	3.70254200
6	2.27717300	1.06494700	4.50390900
1	1.71516600	-0.97144900	4.97222900
1	-0.17174400	-1.00429200	3.33748900
6	0.76934000	3.25031000	1.80973400
1	2.64142000	3.07147800	3.78274200
1	3.09564200	1.09245600	5.22741000
6	0.03679200	3.07672900	0.69676300
1	1.31441800	4.18294500	1.97092300
1	-0.01035700	3.86504400	-0.05608200

6	-1.00319000	1.05223100	1.69863500
1	-1.80385200	1.64090500	2.19105900
6	-0.64841300	1.76885600	0.38052800
1	0.09807700	1.12930500	-0.12367200
8	-1.54500100	-0.24037700	1.47092500
1	-0.89333000	-0.76580600	0.89350300
6	-1.82271500	1.93124500	-0.54815100
1	-3.00573100	-0.31715000	1.04957200
8	-3.41777600	1.54099100	-2.09190900
6	-2.56375600	3.14540600	-0.80404400
1	-2.38877400	4.11710400	-0.34681600
6	-3.51277600	2.86783400	-1.74357700
6	-2.39275900	0.96880700	-1.36439100
8	-2.26190100	-0.31764300	-1.51093000
6	-4.53890700	3.68074500	-2.44634600
1	-4.36189200	3.70612000	-3.53586400
1	-5.55946900	3.28625100	-2.29546700
1	-4.51448500	4.71447900	-2.07327400
6	6.29248500	-2.79552000	-0.20260300
6	5.23650600	-3.41575000	0.42903500
6	3.91596800	-2.89861200	0.32979100
6	3.68007200	-1.69943200	-0.42359200
6	4.79299700	-1.09730700	-1.07826700
6	6.06052900	-1.62931600	-0.97207400
1	2.99487400	-4.45708100	1.53128900
1	7.30256400	-3.20417900	-0.12089300
1	5.39644000	-4.32555100	1.01432800
6	2.81509400	-3.54944200	0.94890800
6	2.35342900	-1.15207900	-0.51375700
1	4.62743800	-0.20315000	-1.67839000
1	6.89381100	-1.14799700	-1.49003400
6	1.29670100	-1.86894200	0.05401600
6	1.54173600	-3.05895400	0.79594000
1	0.67975200	-3.56166600	1.24047300
6	2.12585000	0.17271600	-1.16184800
6	2.75545600	1.34780100	-0.61829100
6	1.31997300	0.29676500	-2.30265800
6	2.61301400	2.61228800	-1.28281200
6	3.51213400	1.32050100	0.58745800
6	1.16096000	1.57250800	-2.92558700
6	1.79697300	2.68692100	-2.44467100
6	3.26253700	3.76034900	-0.75929600
6	4.12956000	2.45357600	1.07331500
1	3.58900100	0.38760500	1.14390800
1	0.52000700	1.61507000	-3.80813900
1	1.67507500	3.65225700	-2.94363100
6	4.01654400	3.68865600	0.39299600
1	3.14446600	4.71037200	-1.28808400
1	4.69939900	2.39611900	2.00345900
1	4.51446700	4.57862900	0.78568600
8	0.01015600	-1.47369200	-0.10657300
8	0.69189400	-0.73890300	-2.86508500
50	-0.85143100	-1.85310700	-2.05354300
6	-4.24270900	-1.80543600	0.28028300
6	-5.56385700	-2.48669300	0.59611500
6	-5.61109600	-1.59917000	2.75853300
6	-4.29820300	-0.85720200	2.54186400
1	-6.41812700	-1.86720400	0.25571300
1	-5.61608400	-3.44477000	0.05993500
1	-3.40545500	-2.46501500	0.55177100
1	-4.12670300	-1.52846600	-0.77452800
1	-6.47604000	-0.93946400	2.54294900
1	-5.68396300	-1.90215300	3.81294700
1	-4.26707300	0.09214600	3.09474000
1	-3.44665900	-1.46942000	2.86666000
8	-5.67504200	-2.76919200	1.97616200
7	-4.06804300	-0.55807500	1.09197800
6	-4.85921900	0.59967800	0.60148200
1	-4.45923300	1.51353200	1.05683800
1	-4.74412100	0.67740800	-0.48389900
1	-5.91863300	0.48070700	0.86083200

INT4

E = -1940.82006683 G = -1940.354517

6	-0.86643700	2.74758300	-2.81393500
6	0.02642000	1.78627700	-2.32393300
6	1.04759600	2.16005700	-1.45585000
6	1.20291800	3.51515300	-1.06599400
6	0.31762000	4.46686500	-1.58935700
6	-0.71288000	4.08736400	-2.45392100
1	-1.67544800	2.44530900	-3.48276500
1	-0.07472900	0.74309700	-2.62820600
6	2.26840200	3.88060300	-0.11758300
1	0.42872600	5.51449700	-1.29789100
1	-1.40145700	4.84119500	-2.84290200
6	2.89808800	2.95745200	0.63731800
1	2.49613500	4.94384700	-0.00189300
1	3.64338400	3.24885300	1.38179600
6	2.10811900	1.23723700	-0.94071700
1	3.01430800	1.38112500	-1.56437400
6	2.45864900	1.53013700	0.50696400
1	1.52334500	1.41895800	1.08329300
6	3.34120500	0.32450400	0.86122300
1	3.24858100	0.05520900	1.92365300
8	3.78529300	-1.32020800	-0.83798200
6	4.76000100	0.34620600	0.37030300
1	5.54435500	1.01000500	0.72898000
6	4.91976800	-0.59925500	-0.56972600
6	2.71174300	-0.82204200	-0.01734100
8	2.09541200	-1.79845500	0.62686500
6	6.11488800	-1.00227200	-1.35760400
1	6.35111400	-2.06425500	-1.17879200
1	5.91893100	-0.89260200	-2.43701300
1	6.98605000	-0.39076900	-1.08737300
6	-1.03160100	3.91000900	2.00445000
6	-0.40740700	2.97221900	2.79879600
6	-0.48741900	1.58660000	2.49682800
6	-1.22889800	1.15515300	1.34650600
6	-1.85515800	2.15178500	0.54664500
6	-1.76052900	3.48907300	0.86735900
1	0.73756600	0.93785800	4.16930900
1	-0.95844500	4.97331500	2.24387100
1	0.16836800	3.28160300	3.67521800
6	0.17414900	0.60882500	3.29193200
6	-1.29433200	-0.24325900	1.02875200
1	-2.40292800	1.84999200	-0.34463900
1	-2.24134800	4.23020000	0.22653000
6	-0.60051600	-1.16955800	1.81566800
6	0.13140300	-0.71981300	2.95526200
1	0.65307200	-1.47540900	3.54471500
6	-2.13507700	-0.73025600	-0.10005100
6	-3.56530900	-0.57586700	-0.07482800
6	-1.57708300	-1.37230300	-1.19459600
6	-4.35658800	-1.03339900	-1.18161500
6	-4.24037900	-0.00202200	1.03943000
6	-2.35157900	-1.84323100	-2.28008500
6	-3.71412200	-1.66553500	-2.27977200
6	-5.76824100	-0.86391900	-1.14763600
6	-5.61164300	0.13963800	1.04568600
1	-3.65557700	0.32252200	1.89900200
1	-1.83453000	-2.33179900	-3.10777600
1	-4.31846400	-2.01467900	-3.12041700
6	-6.38632600	-0.28518000	-0.06119900
1	-6.35572500	-1.21072700	-2.00176000

1	-6.10574300	0.58069000	1.91458900
1	-7.47178600	-0.16215100	-0.04565500
8	-0.60545500	-2.47796900	1.52775000
8	-0.21604800	-1.63605700	-1.27461600
50	0.70865000	-3.28155900	0.14104500
8	1.78734500	-0.14889100	-0.93246500
1	0.40072700	-0.85427700	-1.10245100

INT5

E = -806.074452955 G = -805.857419

6	-3.95646000	1.15044500	0.72652800
6	-2.58156600	1.36034200	0.55983200
6	-1.76164300	0.30400200	0.17627400
6	-2.30165700	-0.98898900	-0.05248400
6	-3.67613500	-1.18421100	0.14445600
6	-4.49918700	-0.12147700	0.52800700
1	-4.60218100	1.98195800	1.01862800
1	-2.13855800	2.34435100	0.72536600
6	-1.41264000	-2.07362600	-0.50722000
1	-4.10437800	-2.17668500	-0.01977300
1	-5.56986500	-0.28738200	0.66884500
6	-0.20000400	-1.82050300	-1.04148000
1	-1.79224300	-3.09766300	-0.44831500
1	0.43347300	-2.62354300	-1.42541200
6	-0.27243100	0.37923000	0.05142000
1	0.17076500	-0.08596100	0.95216500
6	0.20592600	-0.38131800	-1.17667800
1	-0.34632500	0.04434300	-2.03971600
6	1.65831200	0.11836500	-1.28445800
1	1.96417600	0.22550900	-2.33561600
8	2.46888200	0.31248500	1.50192000
6	2.70765600	-0.71065800	-0.58878900
1	3.18992500	-1.48568000	-1.18719600
6	3.10157400	-0.58683100	0.68833100
6	1.50033800	1.56092400	-0.73107300
8	2.25203500	2.47927000	-0.86636900
6	4.18257800	-1.40005000	1.33283100
1	4.98784900	-0.74485500	1.71288700
1	3.78533000	-1.96523800	2.19344400
1	4.63091200	-2.10727200	0.62299400
8	0.30068200	1.67796900	-0.08733700
1	3.05036700	0.58646000	2.22301700

INT6

E = -1940.81375958 G = -1940.348623

6	5.99814300	-2.60280100	-1.67739300
6	4.87867400	-1.94745600	-1.14872200
6	5.02849400	-0.71392700	-0.52305400
6	6.30556600	-0.10331800	-0.41967700
6	7.41732900	-0.78610300	-0.93337800
6	7.26525400	-2.02633700	-1.56012200
1	5.87937600	-3.56756800	-2.17626800
1	3.88282800	-2.38880700	-1.22243400
6	6.42021600	1.23118400	0.19516100
1	8.40830900	-0.33131200	-0.85327400
1	8.14023000	-2.54212900	-1.96271800
6	5.34784200	2.03433800	0.35528300
1	7.42101900	1.57855000	0.46727000
1	5.45249400	3.04717000	0.75328700
6	3.92462700	0.03578500	0.15251200
1	4.04447700	-0.11030100	1.24965500
6	4.01795400	1.52952700	-0.12629700
1	3.98164700	1.64752500	-1.22731900
8	2.59919200	-0.30918100	-0.21412600
1	-0.28310100	-0.27369800	-0.77041700
6	2.66592700	2.02214600	0.42613500
1	2.74108600	2.30770400	1.48652400
8	0.79783800	1.04086000	-0.72183100
6	1.91543600	2.99870900	-0.42996000
1	2.16969300	4.04877200	-0.56213600
6	0.91180300	2.37399700	-1.06384200
6	1.78053700	0.73569700	0.31722900
8	1.16728600	0.36211000	1.43901400
6	-0.06761900	2.85469400	-2.07050600
1	0.03768100	2.28532300	-3.00909300
1	-1.09697000	2.71342600	-1.71277000
1	0.09050700	3.92012000	-2.28187600
6	-3.68494600	4.22292500	-1.24193500
6	-3.05047100	4.17157700	-0.01772600
6	-2.66852300	2.93254400	0.56055600
6	-2.95267300	1.71118600	-0.13728200
6	-3.60310300	1.80039400	-1.40165700
6	-3.95959300	3.02145900	-1.93785700
1	-1.76664400	3.79766000	2.33627300
1	-3.97094600	5.18348800	-1.67642900
1	-2.82415400	5.09173500	0.52775300
6	-1.98665100	2.86627100	1.80793400
6	-2.54564000	0.46108400	0.43336100
1	-3.81341300	0.88443600	-1.95444900
1	-4.45407400	3.06046800	-2.91157500
6	-1.86001600	0.43845800	1.65323100
6	-1.59089600	1.66364700	2.33317700
1	-1.04817600	1.60447600	3.27750800
6	-2.83370000	-0.82716500	-0.25375100
6	-4.17340700	-1.32207000	-0.40069700
6	-1.79817300	-1.60573600	-0.74437300
6	-4.40390500	-2.57507900	-1.06215800
6	-5.29522000	-0.61682700	0.11853600
6	-2.01232400	-2.84835700	-1.38245900
6	-3.29383000	-3.31974700	-1.54433100
6	-5.73653800	-3.05102400	-1.20426000
6	-6.57423500	-1.10927700	-0.03026100
1	-5.12732900	0.32302900	0.64384700
1	-1.14421200	-3.40081900	-1.74604000
1	-3.47216800	-4.27409000	-2.04553100
6	-6.80160600	-2.33424500	-0.70448300
1	-5.89898800	-4.00359100	-1.71528500
1	-7.42033500	-0.55177100	0.37836700
1	-7.82068200	-2.71104100	-0.81888600
8	-1.43728300	-0.71214300	2.19103000
8	-0.46926300	-1.23209500	-0.57535200
50	0.37442600	-1.56864200	1.65109000

INT7

E = -806.076338193 G = -805.859571

6	3.91573100	1.15220000	0.99194900
6	2.65536400	1.40865200	0.43776900
6	1.93188600	0.37213700	-0.14318200
6	2.45133900	-0.94849300	-0.17837400
6	3.72594300	-1.17891800	0.35935300
6	4.45203700	-0.13716100	0.94317700
1	4.48170400	1.96368200	1.45541700
1	2.22726800	2.41284800	0.45337100
6	1.63246800	-2.03448100	-0.74668300
1	4.14319400	-2.18910000	0.33229500
1	5.44026400	-0.33446800	1.36531400
6	0.30051200	-1.90225200	-0.92048400
1	2.13157600	-2.98274000	-0.96563300
1	-0.31613100	-2.73455000	-1.26941500
6	0.61992100	0.53310900	-0.84389600
1	0.81835000	0.50582200	-1.93696600
6	-0.32501800	-0.60493300	-0.50271800
1	-0.43621200	-0.61706600	0.59846700
8	-0.12459300	1.71901000	-0.56624800

1	-3.02542600	0.40050100	2.37352900
6	-1.63980200	-0.05080500	-1.06569400
1	-1.61108600	-0.11958300	-2.16864700
8	-2.51966100	0.10462900	1.60603300
6	-2.92675400	-0.63526900	-0.56707900
1	-3.56767200	-1.16848300	-1.26917700
6	-3.34018800	-0.51533600	0.70495400
6	-1.45738800	1.45711700	-0.74127100
8	-2.29192300	2.30649100	-0.66039300
6	-4.63871600	-1.03183500	1.24277600
1	-4.46734700	-1.74851200	2.06476900
1	-5.24709400	-0.20271200	1.64806900
1	-5.22597600	-1.52907400	0.45989600

INT8

E = -5944.83525721 G = -5943.309167

6	1.59028900	-3.42939800	1.39073600
6	4.18558100	-1.31904300	0.16753500
6	3.49098400	-0.16809400	0.70307900
6	3.86453900	-0.05982800	2.08572100
6	4.73695100	-1.12816400	2.41407100
6	4.93003300	-1.90366300	1.24131900
15	2.28844300	1.07567700	0.05972100
15	-0.23109300	-1.42105700	0.27146300
6	2.49832400	1.25778900	-1.77331700
6	3.10675200	2.62055100	0.64492800
6	2.32133600	3.75964700	0.83296600
6	2.90756200	4.98243900	1.19635500
6	4.29348500	5.03763500	1.37157400
6	5.10598100	3.90641000	1.18276900
6	4.49929300	2.70299400	0.81144200
6	1.84138600	0.37739800	-2.64372600
6	1.87002100	0.57588800	-4.03007200
6	2.54133900	1.69641900	-4.53639200
6	3.19059400	2.60123600	-3.69018400
6	3.17066000	2.36361500	-2.30917200
26	2.90874600	-1.83925400	1.74963800
6	0.90514100	-2.17500000	1.47434900
6	2.28971900	-3.64052200	2.60766100
6	1.16209700	-1.61253900	2.79001800
6	2.02239400	-2.53739600	3.46592900
1	1.23881600	3.68032600	0.73713800
1	5.12389000	1.82570300	0.64449100
1	1.28168300	-0.46392800	-2.24356100
1	3.67099600	3.07176600	-1.65055700
1	5.15382800	-1.32729000	3.39926100
1	5.54586500	-2.79478700	1.15572300
1	2.93749600	-4.48530700	2.83398600
1	2.44583500	-2.40755100	4.45620400
1	1.56683600	-4.11053800	0.54651700
1	4.75955600	5.98406800	1.66271200
1	2.55133300	1.87098700	-5.61614200
45	-0.12141600	0.84002500	0.41382900
6	-1.81135200	-2.13967900	0.93221400
6	-2.58210600	-1.41337100	1.84451300
6	-2.12332500	-3.48557700	0.68256900
6	-3.63641800	-2.02640800	2.54010500
1	-2.34469700	-0.36854600	2.03133800
6	-3.18393700	-4.11495700	1.34371100
1	-1.52640300	-4.05953200	-0.02924900
6	-3.92136700	-3.37075700	2.27766000
1	-4.74355100	-3.85564700	2.81247600
6	-0.10238600	-2.29419000	-1.33844100
6	1.04213400	-2.94845200	-1.80341500
6	-1.22000100	-2.18069100	-2.18583400
6	1.07795400	-3.51978300	-3.08798400
1	1.92556100	-3.00278700	-1.17510500
6	-1.21216400	-2.73770100	-3.46827300
1	-2.11459100	-1.66485300	-1.83366500
6	-0.05918700	-3.41377900	-3.89439400
1	-0.03872500	-3.84730500	-4.89798800
6	-4.40867600	-1.26652800	3.58760700
1	-5.38368100	-1.73130000	3.79355500
1	-3.85377600	-1.24942400	4.53914900
1	-4.57452800	-0.21924500	3.29409300
6	-3.49881500	-5.56794600	1.09241400
1	-2.96608500	-6.21530200	1.80993700
1	-4.57354000	-5.77544900	1.20643500
1	-3.19245100	-5.87893000	0.08256000
6	2.32306900	-4.19612900	-3.59882800
1	2.65608000	-4.99939000	-2.92570100
1	2.16241500	-4.62488700	-4.59792100
1	3.16348800	-3.48673900	-3.66228900
6	-2.41111100	-2.62472800	-4.37616300
1	-2.11366600	-2.65108300	-5.43476500
1	-3.11398500	-3.45980700	-4.21462500
1	-2.96636300	-1.69214700	-4.19819500
6	3.87052000	3.83270100	-4.23082100
1	3.97587200	3.79362200	-5.32473900
1	3.29083700	4.73716300	-3.97919300
1	4.87279000	3.96263300	-3.79344900
6	1.18069500	-0.38911200	-4.95893400
1	1.73649700	-1.33822800	-5.03276400
1	0.17618500	-0.64923200	-4.59483200
1	1.09030600	0.02408600	-5.97406200
6	6.59674200	3.98985100	1.39363100
1	7.10867100	3.09563400	1.00918700
1	7.02612000	4.87260300	0.89403600
1	6.83924400	4.07677400	2.46642600
6	2.03825900	6.20125200	1.37820400
1	2.57930100	7.02062300	1.87351400
1	1.68122400	6.57487200	0.40350100
1	1.14480400	5.96339400	1.97686000
1	3.52201800	0.70717400	2.76778200
6	0.55830400	-0.32879500	3.32974500
1	0.27384700	0.25734300	2.42503900
6	4.38761900	-1.68321500	-1.29061900
1	3.40099700	-1.69875000	-1.78203700
7	5.10842300	-0.59947300	-1.94355100
6	4.98766100	-3.08960100	-1.44554000
6	4.37325800	-4.19954200	-0.84289400
6	6.14307200	-3.31630800	-2.20379000
6	4.88111700	-5.48870700	-1.00303300
1	3.50045000	-4.04365900	-0.21364300
6	6.65576900	-4.60751600	-2.37212500
1	6.65823500	-2.47999200	-2.66985200
6	6.02584300	-5.70116000	-1.77740800
1	4.38292900	-6.33174700	-0.51739900
1	7.55812500	-4.75385100	-2.97045900
1	6.42695400	-6.70879900	-1.90735500
6	5.04734800	-0.51917700	-3.38257700
1	5.86257700	-1.05861800	-3.90931100
1	4.09425800	-0.93075900	-3.73947700
1	5.09200400	0.53492600	-3.70107100
6	6.31724000	-0.06772500	-1.36212400
1	6.44277100	0.98225800	-1.68003700
1	6.25819500	-0.07787600	-0.26587300
1	7.24421400	-0.60970400	-1.64878900
7	-0.71504400	-0.56193800	4.02114800
6	1.49651600	0.59851100	4.09509800
6	1.60528700	1.93212500	3.67274800
6	2.22072100	0.19705100	5.22858400
6	2.47194500	2.82041300	4.31328300
1	1.01714100	2.27756100	2.82128400

6	3.08123000	1.08658300	5.87716700
1	2.12963800	-0.81684300	5.61504100
6	3.22304800	2.39753100	5.41204400
1	2.56878000	3.84017600	3.93530500
1	3.64740300	0.75100800	6.74941800
1	3.90873900	3.08633500	5.91079000
6	-1.39940900	0.68000800	4.33207300
1	-2.43273600	0.46476300	4.63967000
1	-0.91792800	1.26118200	5.14454900
1	-1.42533900	1.32454400	3.43883800
6	-0.70852800	-1.49590700	5.12752100
1	-0.19659000	-1.11326700	6.03608300
1	-1.74814300	-1.72456600	5.41121200
1	-0.23552300	-2.44101500	4.82971400
6	-1.00348300	3.75141800	-0.85688100
6	-0.66199700	5.05273400	-1.21352400
1	-0.79630800	5.86115900	-0.48958500
6	-0.14408200	5.32531700	-2.48790300
1	0.13365900	6.34639600	-2.75936200
6	0.01888500	4.28435400	-3.40505200
1	0.42210600	4.48609200	-4.39986500
6	-0.31592700	2.97460300	-3.04772300
1	-0.17821800	2.16456200	-3.76290000
6	-0.81094700	2.69079200	-1.77079000
6	-1.17232800	1.32148700	-1.34763800
6	-2.12024900	1.12100500	-0.28668600
6	-2.67500600	2.34415700	0.40278200
6	-1.50358300	3.36318800	0.52101800
1	-1.84225200	4.26674300	1.06766200
1	-1.05629200	0.55110800	-2.11406300
1	-2.98987000	2.08308800	1.42451700
1	-2.74869700	0.22627200	-0.30186000
8	-0.48490800	2.72491200	1.20725200
6	-4.87364900	1.80332300	-0.63840700
6	-3.97903900	2.94574000	-0.29671300
6	-3.77499300	3.54801700	-1.65948900
6	-4.31060000	2.75019200	-2.58362800
8	-4.98059000	1.65191900	-1.93091700
1	-4.45919200	3.62057400	0.43023600
1	-3.22054100	4.45644700	-1.86800200
8	-5.43996900	1.01104500	0.15476400
6	-4.34857800	2.70756700	-4.05891700
1	-5.38261300	2.69525800	-4.43917100
1	-3.82856100	3.58775200	-4.45884100
1	-3.83694800	1.80514000	-4.43240500
14	-6.64211000	-0.34827600	-0.08961200
6	-7.38570000	-0.45560300	1.63953500
1	-6.68347400	-1.10907400	2.18380300
6	-5.56312500	-1.82426000	-0.53406500
1	-4.71427000	-1.75869900	0.16803800
6	-7.76713200	0.27215600	-1.47447700
1	-7.08507000	0.45731300	-2.32123900
6	-8.75892800	-1.15269600	1.62831400
1	-9.09597400	-1.34378600	2.65990100
1	-9.52582800	-0.52434000	1.14936900
1	-8.74381500	-2.12055000	1.10433100
6	-7.45078200	0.88529000	2.38812600
1	-7.84228000	0.73179100	3.40690700
1	-6.46244300	1.35837300	2.47791400
1	-8.12143500	1.60082800	1.88652200
6	-5.00589700	-1.78139000	-1.96255300
1	-4.42115100	-0.87040900	-2.15727200
1	-4.34221900	-2.64298500	-2.12943500
1	-5.80450700	-1.83078900	-2.72039100
6	-6.28062700	-3.15630600	-0.25157500
1	-6.58724200	-3.24704600	0.80060400
1	-7.17511400	-3.29040000	-0.88073600
1	-5.60120500	-3.99667400	-0.46216700
6	-8.76761900	-0.80839400	-1.92728200
1	-8.26555000	-1.73720900	-2.23765300
1	-9.48771600	-1.06517300	-1.13657300
1	-9.34895700	-0.44707300	-2.79119600
6	-8.47518100	1.59298200	-1.13419100
1	-7.77308100	2.38685900	-0.83192700
1	-9.03692400	1.96787900	-2.00520000
1	-9.19759200	1.46481800	-0.31313900

(*R*)-**LA4**

E = -1134.71663293 G = -1134.498246

6	-4.42851400	-2.01492500	1.07787600
6	-3.64587800	-2.82991900	0.28812600
6	-2.34100000	-2.43239300	-0.10803200
6	-1.83247900	-1.15613600	0.30353700
6	-2.66106100	-0.35027300	1.13569300
6	-3.92136500	-0.76687000	1.51118100
1	-1.90462900	-4.24802500	-1.22040400
1	-5.42990000	-2.33217100	1.37803900
1	-4.01600400	-3.80406200	-0.04271700
6	-1.51479000	-3.27911300	-0.89771900
6	-0.51413700	-0.73903400	-0.09966400
1	-2.28335400	0.60889000	1.48815800
1	-4.53153900	-0.12810500	2.15440800
6	0.27949000	-1.63516200	-0.82306700
6	-0.24268900	-2.89663300	-1.23429700
1	0.41260200	-3.53966300	-1.82480700
6	-0.04894400	0.63761600	0.22575100
6	-0.74535400	1.78456600	-0.29375600
6	1.04408300	0.83849700	1.07812400
6	-0.37238900	3.10270600	0.13408000
6	-1.79517700	1.66651800	-1.24862900
6	1.40591300	2.15708600	1.49134400
6	0.70576600	3.25164700	1.04987700
6	-1.07518000	4.22879300	-0.37311100
6	-2.45227600	2.77971600	-1.72930600
1	-2.07412100	0.67549300	-1.60550000
1	2.24499400	2.25416800	2.18371500
1	0.97989600	4.25546200	1.38478700
6	-2.09893700	4.07613000	-1.28329400
1	-0.78172400	5.22429900	-0.02934600
1	-3.25165400	2.65967500	-2.46453100
1	-2.63089600	4.94949500	-1.66765800
8	1.54986900	-1.37488800	-1.19370400
8	1.80484600	-0.20414600	1.50823400
50	3.07916200	-0.64960200	-0.06957300

(*R*,*R*,*R*)-**3a**

E = -806.095919999 G = -805.879311

6	3.87505700	1.26036100	0.95173000
6	2.60848200	1.46464300	0.39001800
6	1.91482300	0.39029200	-0.15682800
6	2.47076100	-0.91565800	-0.15075800
6	3.75082600	-1.09392100	0.39334000
6	4.44704000	-0.01437000	0.94380500
1	4.41729900	2.10126300	1.39024100
1	2.15268500	2.45649100	0.37588500
6	1.68346800	-2.04062500	-0.68637900
1	4.19589900	-2.09244200	0.39826700
1	5.43970900	-0.17065500	1.37245600
6	0.34828800	-1.95227400	-0.86326500
1	2.20955500	-2.97944300	-0.88095300
1	-0.23805500	-2.81368100	-1.19442800
6	0.59885200	0.49008000	-0.86062900
1	0.79545300	0.43045900	-1.95251200
6	-0.31554600	-0.66327800	-0.47500000

1	-0.41963400	-0.64071500	0.62497300
8	-0.17763200	1.66924200	-0.62750800
1	-3.78818300	-0.27755400	-1.12461800
6	-1.63434000	-0.15193700	-1.05322300
1	-1.61096600	-0.26823700	-2.15348100
8	-2.37916000	-0.05637200	1.69883800
6	-2.95714500	-0.70406500	-0.53956800
1	-3.00979600	-1.79633700	-0.69944900
6	-3.23822800	-0.43942100	0.93550500
6	-1.49476300	1.35890300	-0.78403300
8	-2.37247000	2.16963700	-0.72255100
6	-4.66350600	-0.66978600	1.38678600
1	-5.08142200	-1.59506700	0.95983200
1	-4.70990900	-0.69504000	2.48306400
1	-5.28296400	0.16724300	1.02149000

(*R*,*R*,*R*)-**7**
E = -806.081788339 G = -805.864416

6	-3.90703800	-1.64971500	-0.23707600
6	-2.50771600	-1.62042400	-0.19551000
6	-1.82847800	-0.40404200	-0.16012200
6	-2.55508000	0.80974000	-0.17363100
6	-3.95538200	0.76772000	-0.23718700
6	-4.63020100	-0.45517700	-0.26430700
1	-4.43064100	-2.60849700	-0.25731000
1	-1.92957100	-2.54617700	-0.19962200
6	-1.81618100	2.07382400	-0.09797700
1	-4.51745600	1.70534500	-0.25380200
1	-5.72174900	-0.47473000	-0.30641700
6	-0.52466500	2.10776800	0.27393100
1	-2.36013200	3.00036700	-0.30216200
1	-0.00108700	3.06231700	0.38139000
6	-0.31281200	-0.33172400	-0.20618600
1	-0.02736400	-0.13376000	-1.25616000
6	0.21022200	0.84041100	0.63591700
1	-0.00639900	0.61551000	1.70175000
6	1.74192600	0.97681200	0.54035200
1	2.02421500	1.87981200	1.11436200
8	3.38695900	-0.69416500	0.32751400
6	2.36222500	0.97580100	-0.82674100
1	2.11225300	1.64894900	-1.64417600
6	3.28118700	0.00184400	-0.87306900
6	2.45928500	-0.20011600	1.20849800
8	2.30247500	-0.65015600	2.30631000
6	4.18925300	-0.47175400	-1.94574100
1	5.24098700	-0.40495000	-1.62248100
1	3.98566300	-1.53024100	-2.17670100
1	4.05496600	0.12627500	-2.85690200
8	0.32695700	-1.54840500	0.11283800
1	0.20591800	-1.72422700	1.05803900

(*S*,*R*,*R*)-**4a**
E = -806.095225244 G = -805.878863

6	-4.33737500	0.59106500	0.26234200
6	-3.03275200	1.09649700	0.32491100
6	-1.95068700	0.23146100	0.19822500
6	-2.14818800	-1.16029300	0.00421800
6	-3.46217100	-1.64930400	-0.02977200
6	-4.54914300	-0.77982300	0.09496700
1	-5.18846200	1.27002400	0.35179200
1	-2.84942600	2.16275000	0.47088400
6	-0.98064400	-2.03831900	-0.18662400
1	-3.63075500	-2.72003700	-0.17155900
1	-5.56697600	-1.17489200	0.05747700
6	0.23467400	-1.55218800	-0.51587100
1	-1.14533500	-3.11767600	-0.12451400
1	1.07430500	-2.20819500	-0.74979800

6	-0.51647300	0.63224500	0.33817300
1	-0.18563800	0.32104500	1.35134000
6	0.35688200	-0.06494400	-0.69492400
1	-0.07666100	0.18138900	-1.68556500
6	1.64859700	0.75108000	-0.57744400
1	2.19406500	0.81605400	-1.52790400
8	3.43841800	-1.35124400	-1.01750400
6	2.63727800	0.31315000	0.50541300
1	3.32847700	1.14548500	0.72950500
6	3.48821800	-0.88255200	0.09837400
6	1.07920100	2.14289700	-0.23951800
8	1.64051600	3.19676000	-0.30384000
6	4.39326900	-1.44958300	1.17061500
1	5.07659000	-2.18775000	0.73184400
1	4.96257500	-0.65207600	1.67364900
1	3.77609900	-1.94027100	1.94246800
8	-0.21252300	2.02087300	0.19259300
1	2.13573300	0.08471700	1.46188600

(*S*,*R*,*R*)-**6**
E = -806.081101518 G = -805.864167

6	4.58360800	0.49823400	-0.29574600
6	3.32171900	1.07631200	-0.47960900
6	2.16267000	0.33108400	-0.26458600
6	2.26097300	-1.02264100	0.13369000
6	3.53067200	-1.59555900	0.29658600
6	4.68710900	-0.84036100	0.08769600
1	5.48445300	1.09371800	-0.46178600
1	3.22909400	2.11463600	-0.80333900
6	1.03148900	-1.78077200	0.38389600
1	3.60707300	-2.64278800	0.60094700
1	5.66949100	-1.29847200	0.22442700
6	-0.15660000	-1.16716100	0.51064000
1	1.11462300	-2.86339700	0.51351200
1	-1.05460300	-1.74448300	0.74695400
6	0.78610100	0.91510400	-0.53572600
1	0.50785900	0.63288800	-1.56861600
6	-0.27327800	0.33342400	0.41373500
1	-0.07809400	0.75346200	1.42308400
8	0.77167900	2.32863600	-0.53002000
1	1.07240000	2.62089700	0.34307900
6	-1.68492800	0.81178000	0.02122400
1	-1.62171700	1.90823600	-0.09313900
8	-3.73481400	-0.20588800	0.57680500
6	-2.32400600	0.15644100	-1.16913800
1	-1.92829100	0.13905100	-2.18227000
6	-3.48150700	-0.39466300	-0.78139500
6	-2.69482100	0.50930400	1.12741900
8	-2.64969000	0.78623000	2.28903300
6	-4.52914500	-1.15268800	-1.50861200
1	-4.66244100	-2.15227200	-1.06320000
1	-5.49989200	-0.63476600	-1.43998400
1	-4.25768000	-1.26557000	-2.56665800

TS1a
E = -1940.81296969 G = -1940.351203

6	-1.39386100	2.09039300	-3.04330100
6	-0.28278900	1.39836800	-2.54580600
6	0.57569400	2.01353000	-1.63979000
6	0.34500700	3.34965900	-1.22562300
6	-0.74268000	4.04316900	-1.77163400
6	-1.61368600	3.41673900	-2.66673500
1	-2.07828900	1.58996400	-3.73167000
1	-0.09020600	0.37536000	-2.86922700
6	1.21801400	3.95434800	-0.20846600
1	-0.92455700	5.07538600	-1.46223400
1	-2.47067400	3.96438200	-3.06573400

6	2.03865400	3.21090600	0.55930600
1	1.12560600	5.03054900	-0.04022700
1	2.61815200	3.66287400	1.36822900
6	1.83966800	1.40175900	-1.10815400
1	2.68327300	1.83461900	-1.67993900
6	2.04457600	1.72400800	0.36197800
1	1.18237700	1.30784700	0.91255100
6	3.25408700	0.86207200	0.73618200
1	3.29666600	0.69741600	1.82439600
8	4.15634800	-0.84384500	-0.66450000
6	4.58130800	1.24211700	0.13985900
1	5.11060800	2.17717900	0.31216500
6	5.01760600	0.23885000	-0.63604800
6	2.99991400	-0.50582600	0.04045500
8	2.43612400	-1.46633200	0.67349000
6	6.25056300	0.07188900	-1.44613900
1	6.80993900	-0.81878300	-1.11579600
1	5.99501600	-0.08077800	-2.50761800
1	6.89722500	0.95467700	-1.35584800
6	-1.65485200	3.75826900	2.23010600
6	-0.88274400	2.92531200	3.01144500
6	-0.70204300	1.55898200	2.66952900
6	-1.33194100	1.03180600	1.49121600
6	-2.09498500	1.92964300	0.69290700
6	-2.25881100	3.24917400	1.05695800
1	0.59318100	1.09824300	4.35151300
1	-1.79078500	4.80665800	2.50581800
1	-0.39385400	3.30451000	3.91303700
6	0.10688800	0.69518300	3.45893900
6	-1.16410700	-0.35529000	1.15112100
1	-2.54182700	1.56971600	-0.23237000
1	-2.84889800	3.91052000	0.41946400
6	-0.35798100	-1.16921800	1.95588000
6	0.28213800	-0.61869800	3.10703400
1	0.90546500	-1.29105700	3.69901500
6	-1.87389400	-0.93604000	-0.02355500
6	-3.31130400	-0.94707100	-0.08311200
6	-1.16980800	-1.47808000	-1.09400000
6	-3.97856400	-1.42042700	-1.26246400
6	-4.11562800	-0.51916000	1.01137900
6	-1.83235700	-1.96427800	-2.25063000
6	-3.20199300	-1.91682200	-2.34369600
6	-5.39943000	-1.40407900	-1.31673000
6	-5.49197300	-0.52629900	0.93242400
1	-3.62569200	-0.18820600	1.92618000
1	-1.21666100	-2.36352700	-3.05913400
1	-3.71037400	-2.27731300	-3.24150300
6	-6.14524200	-0.96154700	-0.24634600
1	-5.89017900	-1.76029300	-2.22643600
1	-6.08439500	-0.19655000	1.78926400
1	-7.23653800	-0.95632100	-0.29875100
8	-0.16814400	-2.46826100	1.69129200
8	0.19674500	-1.59493000	-1.07373200
50	1.13353100	-3.12633800	0.22863700
8	1.96686800	-0.01378100	-1.20056500
1	0.98468900	-0.67302700	-1.14180500

TS1b

E = -1940.80606878 G = -1940.343848

6	0.3834000	4.19882100	-0.72609400
6	0.68860000	2.83719900	-0.61526600
6	1.96185700	2.38539300	-0.94569000
6	2.96131300	3.28716200	-1.39034600
6	2.62788100	4.64266500	-1.52313900
6	1.34786900	5.09625200	-1.19072800
1	-0.61217500	4.55225100	-0.44884300
1	-0.06152700	2.13430100	-0.25382200
6	4.31591800	2.77773900	-1.66370600
1	3.38567900	5.34859200	-1.87320800
1	1.10599600	6.15715000	-1.28874200
6	4.72926100	1.58245100	-1.19545900
1	5.00250400	3.42665000	-2.21460800
1	5.75409900	1.23455800	-1.34811800
6	2.37316600	0.94395000	-0.93813600
1	2.36675500	0.57130600	-1.98275500
6	3.76648500	0.77743500	-0.36635700
1	3.73636300	1.21924800	0.64848500
8	1.57205900	0.06760600	-0.15063800
1	0.62967500	-0.18166500	-0.73717200
6	3.92515300	-0.74807300	-0.18582700
1	4.44946600	-1.20199600	-1.04057700
8	2.28466800	-1.83432000	1.16071000
6	4.45037400	-1.14387900	1.16638900
1	5.45291100	-0.93950900	1.53739200
6	3.45591400	-1.68866200	1.88196500
6	2.48480000	-1.34758600	-0.12254600
8	2.05425300	-2.09811000	-1.07359900
6	3.37818400	-2.16484600	3.28510000
1	2.61782500	-1.58850600	3.83586200
1	3.07083800	-3.22282000	3.31714400
1	4.34919400	-2.05631200	3.78594900
6	-1.14129900	2.58490100	3.95900700
6	-0.68950100	1.30617400	4.21053300
6	-0.80307000	0.28112500	3.23486100
6	-1.40914600	0.57215600	1.96806300
6	-1.86676600	1.90286900	1.74433400
6	-1.73203900	2.88127800	2.70814100
1	0.10846100	-1.27004900	4.45167700
1	-1.04276900	3.36583200	4.71654800
1	-0.22714700	1.06116400	5.17086100
6	-0.32337900	-1.03670700	3.47429100
6	-1.51505900	-0.45722900	0.97024100
1	-2.32801100	2.15039500	0.78831100
1	-2.08545100	3.89470700	2.50207000
6	-0.94464500	-1.71351800	1.21385900
6	-0.38126100	-1.99342400	2.49577300
1	0.01396000	-2.99792200	2.65242000
6	-2.22992500	-0.17833800	-0.30604200
6	-3.63317500	0.13375800	-0.31730200
6	-1.54916800	-0.20201000	-1.51825200
6	-4.27994500	0.47533700	-1.55253800
6	-4.42596500	0.09430400	0.86465100
6	-2.19104700	0.12936100	-2.74023500
6	-3.52112000	0.47301600	-2.75461900
6	-5.66561000	0.79385100	-1.55000600
6	-5.77018500	0.39983200	0.83217000
1	-3.95372600	-0.18948900	1.80468800
1	-1.59145700	0.11313500	-3.65244900
1	-4.01393300	0.73995900	-3.69289900
6	-6.39890300	0.76291200	-0.38381500
1	-6.14119200	1.05885600	-2.49811800
1	-6.35681200	0.35798100	1.75308300
1	-7.46359600	1.00780800	-0.39515800
8	-0.92141100	-2.69230200	0.30163700
8	-0.23763400	-0.58900300	-1.58147800
50	0.03089600	-2.78901200	-1.53861500

TS2a'

E = -1940.79696341 G = -1940.337234

6	-7.66028100	0.45546300	-1.08321100
6	-6.26392600	0.35172500	-1.12174600
6	-5.54358500	0.28092100	0.06601700
6	-6.20522300	0.31106200	1.32164300
6	-7.60129500	0.44087600	1.33874400

6	-8.32471400	0.50885800	0.14465300
1	-8.22810100	0.50145100	-2.01544100
1	-5.72693400	0.32430700	-2.07182200
6	-5.41418900	0.18476500	2.55922400
1	-8.12412100	0.47409600	2.29831600
1	-9.41295300	0.60096900	0.17388000
6	-4.15467600	-0.29977500	2.55166200
1	-5.90431000	0.44435700	3.50185600
1	-3.59718600	-0.44755100	3.47997100
6	-4.05087800	0.25518100	0.15752700
1	-3.72522500	1.27498400	0.45138100
6	-3.58948000	-0.72798700	1.22788800
1	-4.05122100	-1.70064900	0.96499600
6	-2.08924600	-0.85556600	0.91636600
1	-1.76650100	-1.90374900	0.99378900
8	-1.38600300	1.07988900	-0.42838400
6	-1.12061200	0.08640200	1.60245600
1	-1.08128400	0.18657000	2.69033200
6	-0.98373400	1.22099400	0.78500100
6	-2.05158800	-0.41969900	-0.60171000
8	-1.35315800	-1.06039600	-1.47412900
6	-0.41676100	2.53593600	1.19176900
1	0.03828200	3.04681200	0.33637000
1	-1.24475900	3.15911000	1.57117800
1	0.32355000	2.42074500	1.99294600
6	2.63683800	4.79201200	0.17862600
6	2.09574400	4.34613000	-1.00949000
6	2.00790500	2.95913800	-1.30363800
6	2.50409300	2.00184200	-0.35535300
6	3.05180100	2.49846500	0.86305000
6	3.11449300	3.85218500	1.12329800
1	1.01370100	3.21734600	-3.21752200
1	2.69475600	5.86155600	0.39331200
1	1.71347400	5.05831400	-1.74587800
6	1.40131700	2.48850700	-2.50096400
6	2.40381900	0.59919100	-0.64092400
1	3.41836800	1.78959900	1.60543700
1	3.53595300	4.20158400	2.06920200
6	1.78376000	0.17917400	-1.82403800
6	1.27983000	1.14557300	-2.74470900
1	0.79630700	0.76916600	-3.64747900
6	2.96969700	-0.40318300	0.30511900
6	4.38128400	-0.45426500	0.57229700
6	2.14517800	-1.31395000	0.95786700
6	4.89363800	-1.37365100	1.54868100
6	5.30990800	0.37243600	-0.12170500
6	2.65283200	-2.22798400	1.91656500
6	3.99347400	-2.24699600	2.21709100
6	6.29036000	-1.40289700	1.81379600
6	6.65987700	0.31317100	0.15169600
1	4.93724900	1.05470300	-0.88508400
1	1.94688000	-2.90501700	2.40159800
1	4.38523800	-2.94440600	2.96172700
6	7.15840700	-0.57547100	1.13548500
1	6.66400900	-2.10371100	2.56505800
1	7.35223400	0.95479900	-0.39852600
1	8.22987500	-0.60908000	1.34636700
8	1.65995800	-1.11502500	-2.13644800
50	0.30199100	-2.40977700	-1.27969400
8	-3.34003100	-0.13363300	-1.00690800
8	0.80285800	-1.38622600	0.66424000
1	0.05977000	-0.58582100	1.18756700

TS2a

E = -1940.80947738 G = -1940.347715

6	-5.06673600	1.31523200	-2.77361100
6	-4.07616000	0.61623500	-2.07202700
6	-4.20340500	0.41827200	-0.70094200
6	-5.34252300	0.90105500	-0.00401400
6	-6.31000700	1.62144600	-0.71888400
6	-6.17429000	1.82803100	-2.09450400
1	-4.96682000	1.46360300	-3.85132100
1	-3.20071600	0.22233200	-2.58809500
6	-5.49217900	0.60601800	1.43232400
1	-7.18564300	2.00843200	-0.19103300
1	-6.94048500	2.38514000	-2.63867600
6	-4.78663700	-0.37434100	2.03234400
1	-6.24528200	1.17043700	1.98922100
1	-4.94651300	-0.62879300	3.08261900
6	-3.17967500	-0.22252400	0.18104700
1	-2.64614600	0.57907000	0.72732100
6	-3.83652800	-1.16623000	1.18057900
1	-4.43089500	-1.88829500	0.58485200
6	-2.60925200	-1.91799000	1.73957200
1	-2.82440900	-2.98366400	1.91114200
8	-0.47257100	-0.63600400	1.31553400
6	-1.95235600	-1.27404200	2.92580900
1	-2.37703400	-1.32365200	3.92735700
6	-0.82334200	-0.61205400	2.62510300
6	-1.63970400	-1.81053500	0.51968900
8	-0.91090500	-2.78281400	0.13423100
6	0.09077600	0.14922800	3.52677500
1	1.10202800	-0.29157500	3.50471700
1	0.18804000	1.18935200	3.17593200
1	-0.27528200	0.14340800	4.56239400
6	0.02779200	4.42019800	1.30648000
6	-0.65172500	3.84550500	0.25236000
6	-0.17685100	2.65994700	-0.36775500
6	1.03610400	2.05350600	0.09926100
6	1.71151900	2.67284800	1.18917300
6	1.21979400	3.82065100	1.77829200
1	-1.79306000	2.51511700	-1.81446000
1	-0.35096700	5.32958600	1.77875000
1	-1.57808000	4.29153100	-0.11989500
6	-0.88262600	2.04254100	-1.43759900
6	1.50081000	0.83428100	-0.49850600
1	2.62689400	2.21985400	1.57054100
1	1.75597800	4.26897500	2.61853100
6	0.71582700	0.20213200	-1.46749900
6	-0.45058600	0.85383800	-1.96337400
1	-0.99428500	0.34649000	-2.76071000
6	2.78146500	0.22247600	-0.05196000
6	4.04050900	0.87290700	-0.28757500
6	2.78805700	-0.99878500	0.60734600
6	5.25493700	0.27321500	0.18820500
6	4.13233200	2.09825700	-1.00598700
6	3.98519300	-1.59955500	1.06979100
6	5.19172600	-0.97166400	0.87127300
6	6.49410500	0.93034200	-0.04557500
6	5.35060200	2.70687700	-1.22139200
1	3.21867500	2.55051700	-1.39089600
1	3.91338700	-2.55574100	1.59164800
1	6.11744200	-1.42489800	1.23434300
6	6.54509500	2.12419800	-0.73148600
1	7.41012400	0.46471500	0.32757300
1	5.39571500	3.64603900	-1.77795800
1	7.50349300	2.61895300	-0.90559600
8	1.00968100	-1.01109400	-1.96033200
8	1.62000000	-1.70329500	0.80539200
50	0.94310800	-2.85287500	-1.01763800
8	-2.19610500	-1.04425600	-0.45039200
1	0.69526700	-1.10256000	1.08171300

TS2b'

E = -1940.80014385 G = -1940.341047

6	-7.23506400	-0.17093500	-0.47842000
6	-5.90607200	-0.60900100	-0.54209900
6	-4.87614900	0.25033400	-0.17372100
6	-5.15277200	1.56985700	0.27213200
6	-6.48798200	1.99686000	0.30464500
6	-7.52248200	1.13222500	-0.06483900
1	-8.04534600	-0.84758900	-0.76004100
1	-5.66094300	-1.61855700	-0.87759700
6	-4.04011500	2.43506300	0.70498400
1	-6.71641600	3.01300000	0.63729400
1	-8.55833100	1.47768900	-0.02627900
6	-2.82974600	1.93151400	1.02374800
1	-4.24109000	3.50476700	0.81261400
1	-2.02129400	2.56879700	1.38724700
6	-3.41913400	-0.06845800	-0.27943300
1	-3.02269700	0.45934800	-1.17327900
6	-2.65722800	0.44280000	0.93116200
1	-3.12925800	-0.01385800	1.82484900
8	-3.06192100	-1.44445600	-0.38689500
1	0.61872800	-1.19459200	1.40929700
6	-1.30548000	-0.23918700	0.71372900
1	-0.68206700	0.33515900	0.02490900
8	-1.78504000	-2.54189400	1.34873000
6	-0.57545000	-0.70290800	1.95146800
1	-0.26051100	-0.00725400	2.73424500
6	-1.11045900	-1.95993500	2.27995500
6	-1.74576800	-1.56110500	0.01246400
8	-0.98642500	-2.16207300	-0.84719800
6	-0.88659700	-2.71571900	3.54634400
1	-1.85670200	-2.89833800	4.03579300
1	-0.44903200	-3.70053700	3.31955900
1	-0.23071000	-2.16520700	4.23271600
6	-0.06860700	4.53796600	0.44420300
6	-0.50233400	3.88222200	-0.68868200
6	0.06708600	2.64239500	-1.08235700
6	1.12819000	2.06967900	-0.30250800
6	1.53909400	2.76610200	0.87114200
6	0.95651300	3.96324200	1.23448500
1	-1.21831700	2.36423100	-2.81108900
1	-0.5169580	5.48974200	0.73825400
1	-1.30827800	4.29996400	-1.29738600
6	-0.40963000	1.92958000	-2.21780100
6	1.70153000	0.81247800	-0.69069000
1	2.32757100	2.33718100	1.48993100
1	1.29151700	4.47370000	2.14088800
6	1.16931200	0.11868500	-1.78550000
6	0.11396900	0.70534500	-2.54936500
1	-0.26288400	0.13377400	-3.39917300
6	2.84996900	0.23697400	0.06452700
6	4.12699700	0.89645700	0.07088000
6	2.71953100	-0.95292300	0.77240400
6	5.21010700	0.35271700	0.84049600
6	4.36980900	2.07833800	-0.68516700
6	3.79314400	-1.49447800	1.52491300
6	5.00673400	-0.85176000	1.56685200
6	6.46506300	1.02104600	0.84796700
6	5.60081900	2.69886600	-0.66231700
1	3.56469000	2.48765400	-1.29459400
1	3.61932300	-2.42689300	2.06559100
1	5.83264500	-1.26415300	2.15182400
6	6.65972700	2.17280400	0.11727700
1	7.27805000	0.59714100	1.44337300
1	5.76234800	3.60268000	-1.25464100
1	7.62925600	2.67625400	0.12980200
8	1.60493000	-1.09560000	-2.13176300
8	1.54005200	-1.66011300	0.76235300
50	1.02569500	-2.80707700	-1.10006400

TS2b

E = -1940.80291188 G = -1940.341743

6	6.57037100	-2.27746000	-1.46646500
6	5.42541900	-1.82842600	-0.79625500
6	5.33475900	-0.49930400	-0.39664900
6	6.38793500	0.41475000	-0.66298900
6	7.53621100	-0.06085300	-1.31213200
6	7.62607600	-1.39655000	-1.71398600
1	6.63890400	-3.31901200	-1.78890900
1	4.59791000	-2.50769800	-0.58268400
6	6.23278500	1.82961600	-0.27924200
1	8.35917400	0.62894000	-1.51708700
1	8.52380100	-1.74971400	-2.22673100
6	5.02308200	2.36166900	-0.00544100
1	7.12852400	2.45696400	-0.28479600
1	4.90253100	3.42699800	0.20664800
6	4.21045500	0.08214600	0.39933400
1	4.56057200	0.18592200	1.44873900
6	3.82992900	1.45802000	-0.11534000
1	3.59410000	1.34010000	-1.19076200
8	2.98124900	-0.65201000	0.43447600
1	0.14445000	-0.49994600	-0.77589400
6	2.48098700	1.69616100	0.59232500
1	2.61294000	2.07571400	1.61828500
8	1.08839200	0.31308400	-0.90080100
6	1.47273000	2.45395400	-0.22159500
1	1.37509200	3.53760600	-0.21481200
6	0.78710500	1.63964100	-1.04152900
6	1.96990500	0.24122800	0.67568000
8	1.08840700	-0.10160100	1.52700400
6	-0.17108200	1.98210300	-2.12888300
1	0.30267300	1.81913100	-3.11200700
1	-1.06160100	1.34145500	-2.08152500
1	-0.49808000	3.02709100	-2.05371100
6	-3.21700400	4.49981100	-0.84834800
6	-2.62032600	4.28127000	0.37600000
6	-2.38484200	2.96555900	0.85562200
6	-2.76485600	1.83926100	0.05170500
6	-3.38118100	2.10346200	-1.20581700
6	-3.60232400	3.39340900	-1.64205400
1	-1.48435300	3.59290600	2.72949500
1	-3.39473600	5.51740400	-1.20395500
1	-2.31847700	5.12440000	1.00354400
6	-1.77211900	2.73349200	2.11804900
6	-2.49468000	0.50850700	0.52084200
1	-3.67375200	1.26687400	-1.83952600
1	-4.07515200	3.56107100	-2.61293100
6	-1.89239300	0.32653300	1.76912400
6	-1.53707600	1.45817800	2.56067800
1	-1.06181000	1.26539900	3.52380200
6	-2.84519300	-0.67678900	-0.31260000
6	-4.21361600	-0.98345600	-0.62830000
6	-1.85379900	-1.51793100	-0.81013300
6	-4.52212500	-2.08051900	-1.50080200
6	-5.29606800	-0.24114400	-0.07616500
6	-2.15991500	-2.60696600	-1.66518600
6	-3.46141900	-2.87222400	-2.01719600
6	-5.88052200	-2.36218400	-1.81358200
6	-6.60316500	-0.54639500	-0.39038400
1	-5.07551600	0.57563200	0.61049100
1	-1.33087600	-3.21519400	-2.03183700
1	-3.69637900	-3.70471300	-2.68504700
6	-6.90239700	-1.61082300	-1.27577300
1	-6.09865400	-3.19585300	-2.48629400
1	-7.41604400	0.03569500	0.05028800

1	-7.94225100	-1.83882000	-1.52152600
8	-1.62895300	-0.89693400	2.25402500
8	-0.53386600	-1.37630300	-0.43962100
50	0.01110600	-1.98569400	1.64926000

TS$_{PT\text{-}1}$

E = -2268.09396792 G = -2267.475365

6	1.89393100	-0.51963400	4.29446200
6	0.74847800	-0.62670700	3.49708500
6	0.25571400	0.48454000	2.81537500
6	0.92115300	1.72495800	2.91772100
6	2.04503200	1.83031900	3.74908400
6	2.53297200	0.71468000	4.43291200
1	2.28276100	-1.40098100	4.80998000
1	0.22718400	-1.57921700	3.39495300
6	0.46365300	2.83753900	2.08363900
1	2.55572500	2.79245800	3.83164900
1	3.41998900	0.80656000	5.06444600
6	-0.38066900	2.64241600	1.05728500
1	0.91387600	3.81973300	2.24316200
1	-0.61152600	3.46312700	0.37523000
6	-1.03361100	0.42213400	2.01332000
1	-1.82831800	0.85627600	2.65489300
6	-0.95620400	1.28072600	0.73852900
1	-0.24134700	0.77365300	0.06632400
8	-1.42941000	-0.89835400	1.73313200
1	-0.77536000	-1.26079500	1.07973400
6	-2.28878900	1.38268600	-0.01559600
1	-3.24552300	0.42566400	0.44393100
8	-3.31411000	1.69620200	-2.03659100
6	-3.10256200	2.61880100	-0.00351300
1	-3.21421800	3.29605700	0.84066300
6	-3.70401400	2.74510400	-1.20221000
6	-2.40401400	0.92664500	-1.37889700
8	-1.93320700	-0.05848600	-1.98578900
6	-4.67853400	3.70113100	-1.78175400
1	-4.29637300	4.13650100	-2.71973700
1	-5.63822900	3.20918800	-2.01884000
1	-4.87313300	4.51601900	-1.07094200
6	6.60309900	-1.96689400	-0.23460500
6	5.63886100	-2.74302800	0.37113100
6	4.25869000	-2.42448500	0.25119800
6	3.86342000	-1.26415600	-0.49598700
6	4.88640100	-0.49717100	-1.12434300
6	6.21631300	-0.83828700	-0.99805000
1	3.55322500	-4.11439700	1.42030500
1	7.66065700	-2.22360700	-0.13690500
1	5.92071800	-3.62597400	0.95143200
6	3.25353700	-3.23622600	0.84228800
6	2.47411000	-0.91663300	-0.60458300
1	4.60119100	0.37000700	-1.71944700
1	6.97916400	-0.23386000	-1.49507900
6	1.52276200	-1.78365300	-0.06196600
6	1.92472600	-2.93508200	0.67157200
1	1.13893700	-3.56116200	1.09997600
6	2.05073600	0.35648900	-1.25619200
6	2.45166200	1.62480600	-0.71012900
6	1.25296800	0.33017600	-2.40471000
6	2.04686000	2.84175000	-1.35650600
6	3.24207500	1.73562300	0.46894400
6	0.83322300	1.54926800	-3.01527700
6	1.22257200	2.76480800	-2.51206400
6	2.47091800	4.09161700	-0.83212600
6	3.65043000	2.96332200	0.94443600
1	3.51854200	0.83208300	1.00952400
1	0.19923300	1.47175400	-3.90035600
1	0.90439100	3.69213900	-2.99642700
6	3.26899100	4.15831800	0.28990900
1	2.15323800	5.00468100	-1.34344500
1	4.26229900	3.01146200	1.84793900
1	3.59900700	5.12500400	0.67814200
8	0.19074400	-1.56368000	-0.21068700
8	0.84601000	-0.82161900	-2.96485200
50	-0.67858000	-1.91949500	-2.12541800
6	-3.99984900	-1.71516800	0.14849500
6	-4.97572900	-2.79068300	0.61557300
6	-5.39269900	-1.63733900	2.61028700
6	-4.41901400	-0.52988200	2.21885700
1	-5.99165700	-2.61064100	0.20677100
1	-4.64241800	-3.77199500	0.24702700
1	-2.98543100	-1.96439600	0.48277100
1	-4.00478300	-1.61671700	-0.94624200
1	-6.43488000	-1.37729700	2.33121100
1	-5.36849700	-1.77773400	3.70089800
1	-4.71767100	0.43977900	2.64450400
1	-3.41517900	-0.79381800	2.57442000
8	-5.01989800	-2.86669400	2.02401700
7	-4.30730600	-0.38801800	0.74453800
6	-5.47973200	0.25726100	0.12941600
1	-5.60763000	1.25542300	0.56609000
1	-5.30057800	0.36635300	-0.94803800
1	-6.40546100	-0.31995400	0.27673100

TS$_{PT\text{-}2}$

E = -2268.10184256 G = -2267.483733

6	3.33109200	-0.30766600	3.40553400
6	2.14755900	-0.96159400	3.04327100
6	0.93436700	-0.27913700	3.04587400
6	0.89614800	1.08091100	3.43276600
6	2.07681500	1.71051400	3.84895800
6	3.29403300	1.02479600	3.82290200
1	4.28123800	-0.84448700	3.36105000
1	2.16237000	-2.00964400	2.74588300
6	-0.38188800	1.79153700	3.33778900
1	2.04198500	2.75633300	4.16558400
1	4.21411000	1.53303200	4.12089500
6	-1.38140000	1.31128500	2.57641900
1	-0.48509600	2.74805600	3.85791500
1	-2.32116700	1.86241300	2.49943700
6	-0.38158300	-0.94954100	2.68159000
1	-0.95798700	-1.06715900	3.62117600
6	-1.20141000	0.02859200	1.79621500
1	-0.59066600	0.25024100	0.89945200
8	-0.25630400	-2.22571700	2.14051300
1	0.30176500	-2.17448200	1.32568900
6	-2.48039100	-0.62891900	1.32132500
1	-3.21787600	0.40535200	0.61223200
8	-3.41206300	-2.49896100	0.40497300
6	-3.48363000	-1.20351400	2.23598200
1	-3.75688300	-0.81512500	3.21557100
6	-4.02591200	-2.28650900	1.63902700
6	-2.43356500	-1.56902400	0.23952600
8	-1.75999500	-1.57147200	-0.82209000
6	-5.08681500	-3.25493400	2.01053000
1	-5.92028400	-3.24756900	1.28697500
1	-4.68898700	-4.28315100	2.03808600
1	-5.48617500	-3.01051100	3.00452800
6	6.49285600	1.07935500	-1.31280000
6	6.24745800	-0.12358000	-0.68658400
6	4.92412500	-0.62158200	-0.54496700
6	3.81853900	0.14796900	-1.04266000
6	4.11403200	1.37337800	-1.70708800
6	5.41038300	1.82600500	-1.83696900
1	5.51024600	-2.46996800	0.43523900

1	7.51515900	1.45082600	-1.41669000
1	7.07301400	-0.72272700	-0.29236300
6	4.66818700	-1.88100300	0.06182100
6	2.47305600	-0.33400500	-0.87520400
1	3.29527800	1.95719100	-2.12551200
1	5.60338300	2.76899600	-2.35466900
6	2.27825100	-1.58905800	-0.29894600
6	3.38581000	-2.35805100	0.16022200
1	3.17056900	-3.33005300	0.60923600
6	1.32123800	0.50351900	-1.31975300
6	1.11060700	1.80580900	-0.75122900
6	0.46755900	0.06464000	-2.34094600
6	0.13358400	2.69337600	-1.31890300
6	1.83481600	2.26085500	0.38807300
6	-0.49991800	0.96037000	-2.88931900
6	-0.64496100	2.24143900	-2.41930100
6	-0.04155200	3.99116400	-0.76556900
6	1.62803000	3.51901900	0.91213300
1	2.54867500	1.58889600	0.86308400
1	-1.10560700	0.58367100	-3.71683100
1	-1.39089600	2.90816400	-2.85664800
6	0.68925300	4.40328600	0.32879500
1	-0.76947200	4.66119500	-1.23289300
1	2.19180800	3.83005100	1.79370600
1	0.54652300	5.40320300	0.74590900
8	1.04456000	-2.11044500	-0.13027700
8	0.51630400	-1.17301400	-2.84694200
50	-0.12821700	-2.80978300	-1.74260300
6	-3.29264200	2.60438200	0.01127000
6	-3.90070400	3.55733600	-1.01256700
6	-4.54183800	1.77380900	-2.39558600
6	-3.94612100	0.75873800	-1.42746400
1	-4.94036900	3.82568500	-0.73329800
1	-3.31300600	4.48607400	-1.03476200
1	-2.22504400	2.45772100	-0.19936200
1	-3.38583700	3.01452600	1.02570500
1	-5.62713100	1.91165600	-2.21090700
1	-4.42398300	1.40894400	-3.42602500
1	-4.49371300	-0.19412700	-1.44827600
1	-2.90004400	0.55692100	-1.69350200
8	-3.87119800	3.01387900	-2.31449300
7	-3.94053400	1.26784100	-0.02767400
6	-5.25413300	1.22488200	0.63419800
1	-5.63533800	0.19644600	0.60015000
1	-5.12713800	1.50908600	1.68774700
1	-5.98530100	1.90131600	0.1666850

TS'$_{PT-1}$

E = -806.003110837 G = -805.790114

6	4.2584520	-1.26592100	-0.06039400
6	2.92066100	-1.39607100	0.33357000
6	2.08678000	-0.28257800	0.33058200
6	2.57618000	0.99384600	-0.05599800
6	3.92547000	1.10724500	-0.41947200
6	4.75899100	-0.01478700	-0.42785300
1	4.91009000	-2.14248400	-0.07103600
1	2.52316200	-2.36411500	0.64574900
6	1.65725600	2.14622600	-0.07613100
1	4.31998700	2.08414500	-0.71082500
1	5.80537900	0.08928900	-0.72397300
6	0.31805100	1.98313900	-0.10028600
1	2.09775600	3.14525500	-0.13350900
1	-0.36265700	2.83339500	-0.18317500
6	0.67051600	-0.26226600	0.80663900
1	0.64713100	0.19641300	1.81389600
6	-0.20915400	0.57541000	-0.12827900
1	-0.00190800	0.18721500	-1.14888000
8	-0.02812500	-1.51814400	0.97520200
1	-0.41054000	-1.87724600	0.03255800
6	-1.65141400	0.21163100	0.18307800
1	-1.05587500	-0.93749700	1.09646100
8	-3.52051100	-0.85550100	-0.56512500
6	-2.82529400	1.03092300	0.46529300
1	-2.84963900	1.99854900	0.96284300
6	-3.90926000	0.35079100	0.01880500
6	-2.16639700	-0.93083300	-0.51619200
8	-1.52613100	-1.92443700	-0.93381100
6	-5.36995200	0.60970100	0.05190400
1	-5.79908500	0.59635200	-0.96425500
1	-5.90381300	-0.15598000	0.64056100
1	-5.56718100	1.59263900	0.50185100

TS0-*Re*-Cα

E = -5944.81596737 G = -5943.294337

6	-3.67396600	2.57849700	1.30494700
6	-4.20779800	-0.93278000	1.18926700
6	-2.78992800	-1.13921500	1.39958100
6	-2.56247700	-1.07846900	2.81865600
6	-3.79351300	-0.81241000	3.47042900
6	-4.79943700	-0.71506800	2.47344800
15	-1.32823600	-1.44483900	0.32516100
15	-1.33633100	2.04933400	-0.40860100
6	-1.86906100	-2.17311500	-1.28409900
6	-0.62495900	-2.92427200	1.17450900
6	0.72482300	-3.23240500	0.99334900
6	1.28429800	-4.38360300	1.57064900
6	0.46158100	-5.22559100	2.32333300
6	-0.90469400	-4.94939400	2.50475000
6	-1.43642900	-3.79574400	1.92240700
6	-2.28130600	-1.33273000	-2.32663400
6	-2.57368000	-1.84696800	-3.59529300
6	-2.41834500	-3.22320100	-3.81402900
6	-1.99630500	-4.08273300	-2.79444200
6	-1.73045800	-3.54453600	-1.52737400
26	-3.30018200	0.69753400	2.14521100
6	-2.31560700	2.16844900	1.11538100
6	-3.94119400	2.60249100	2.69996900
6	-1.72404100	1.96137600	2.42385900
6	-2.74860000	2.23502600	3.38583900
1	1.35606300	-2.54373900	0.43881800
1	-2.49648300	-3.58455300	2.05083300
1	-2.36054200	-0.26167500	-2.16231800
1	-1.39033100	-4.21036600	-0.73527400
1	-3.92694100	-0.67243100	4.54121500
1	-5.85284700	-0.50785000	2.64092700
1	-4.89947100	2.83346300	3.16119500
1	-2.65379200	2.13367200	4.46230700
1	-4.37601200	2.83934500	0.52002900
1	0.88787300	-6.12357100	2.78110700
1	-2.62523800	-3.63234900	-4.80701300
45	0.19076900	0.35148400	-0.19873700
6	-0.56955800	3.73401300	-0.40339300
6	0.77377500	3.92772900	-0.07611800
6	-1.39146100	4.84795900	-0.63239100
6	1.31799000	5.21749500	-0.00061400
1	1.40419700	3.06914300	0.13642900
6	-0.87892200	6.14774500	-0.55667800
1	-2.44589100	4.70737100	-0.87991700
6	0.48008000	6.31115400	-0.24583200
1	0.89439700	7.32282100	-0.19378500
6	-2.43781400	2.19383100	-1.86558900
6	-3.77043700	1.77838500	-1.88601500
6	-1.85143000	2.68499000	-3.04701800
6	-4.54016600	1.86929200	-3.05967400

1	-4.21583400	1.36298600	-0.98751500
6	-2.59205000	2.78998400	-4.22673700
1	-0.81179100	3.01926900	-3.04173200
6	-3.93752100	2.38609000	-4.20938500
1	-4.52590100	2.46194300	-5.12815300
6	2.76810100	5.42208900	0.36233100
1	3.22767500	6.21989600	-0.24117800
1	2.87563800	5.71589400	1.42034000
1	3.35386000	4.50182000	0.21815400
6	-1.75536300	7.34823300	-0.80962600
1	-1.76707400	8.02282700	0.06187700
1	-1.38769200	7.93648100	-1.66639200
1	-2.79274500	7.05350000	-1.02317100
6	-5.97196100	1.40078700	-3.07694600
1	-6.59895500	1.98782800	-2.38863100
1	-6.40576700	1.47873100	-4.08366600
1	-6.05422400	0.35361500	-2.74689100
6	-1.97247400	3.32074900	-5.49375800
1	-2.01598600	2.57060900	-6.30040000
1	-2.51091200	4.21170500	-5.85515800
1	-0.91943400	3.59899700	-5.34445800
6	-1.79144400	-5.55519000	-3.04189500
1	-2.16094900	-5.85741700	-4.03233300
1	-0.72051700	-5.81359100	-2.98590300
1	-2.30784700	-6.16425400	-2.28311300
6	-3.03941300	-0.93905900	-4.70345200
1	-4.10713100	-0.68838900	-4.58828600
1	-2.49416800	0.01533400	-4.69793300
1	-2.91477400	-1.40992100	-5.68939600
6	-1.77005200	-5.87431700	3.32286300
1	-2.83195800	-5.59652800	3.25704500
1	-1.66856800	-6.91951100	2.98960400
1	-1.48234900	-5.84659200	4.38731200
6	2.74722400	-4.68414900	1.36065300
1	3.08825900	-5.51830900	1.99076000
1	2.94496100	-4.94956800	0.30886000
1	3.36682600	-3.80227400	1.58822100
1	-1.60299500	-1.20068000	3.30589100
6	-0.26788500	1.61592500	2.66340900
1	0.05451300	1.06545700	1.74178000
6	-5.01399900	-1.15981600	-0.07379900
1	-4.55553800	-0.57209000	-0.88542300
7	-4.87616800	-2.54426900	-0.49959500
6	-6.45034100	-0.64692200	0.09782100
6	-6.70040000	0.72780200	0.22676600
6	-7.54295500	-1.52076200	0.15667700
6	-7.99487600	1.21786800	0.39942400
1	-5.86445000	1.42394600	0.21705100
6	-8.84463500	-1.03639500	0.32669200
1	-7.38387600	-2.59373100	0.07239700
6	-9.07768400	0.33422200	0.44683500
1	-8.15980300	2.29358200	0.50005400
1	-9.67990200	-1.73944200	0.36691300
1	-10.09367800	0.71248300	0.57984000
6	-5.33286900	-2.85312000	-1.83193800
1	-6.42408100	-3.04479000	-1.90863300
1	-5.09262600	-2.02248500	-2.51059500
1	-4.80811700	-3.74626900	-2.20555800
6	-4.97354900	-3.63609700	0.43920600
1	-4.29301600	-4.45137900	0.13290700
1	-4.67388000	-3.31087000	1.44333200
1	-5.98952100	-4.07643000	0.52608900
7	0.57392000	2.80739100	2.68589400
6	0.02187100	0.62771300	3.78726800
6	0.71629000	-0.55322400	3.48336700
6	-0.33397800	0.87157300	5.12223200
6	0.98239300	-1.50317500	4.47172300
1	1.04748500	-0.73877700	2.46010400
6	-0.05924900	-0.07320500	6.11501300
1	-0.82971000	1.80156100	5.40065400
6	0.58575400	-1.27093400	5.79114500
1	1.49131200	-2.43069600	4.19929900
1	-0.35366700	0.12724900	7.14799400
1	0.78616100	-2.01309600	6.56733600
6	1.98684300	2.50211300	2.74250100
1	2.56831500	3.39862700	2.47843800
1	2.33054500	2.15463100	3.73873800
1	2.22389500	1.70619300	2.01977000
6	0.18248100	3.92144500	3.52033200
1	0.35473600	3.75529000	4.60574500
1	0.76709600	4.80772400	3.22504200
1	-0.87746700	4.16304900	3.36744600
6	2.52423000	-1.49472300	-1.68804100
6	3.15902100	-2.72071200	-1.85536500
1	4.03354200	-2.96294600	-1.24799600
6	2.66727500	-3.64815500	-2.78508200
1	3.15996300	-4.61612000	-2.90431500
6	1.54315800	-3.33382200	-3.55124100
1	1.15252900	-4.05215500	-4.27527300
6	0.90417400	-2.10057800	-3.38800500
1	0.02232600	-1.86207400	-3.98153100
6	1.37990000	-1.17677000	-2.45171600
6	0.74073900	0.13788600	-2.23487900
6	1.53224600	1.22857200	-1.71752500
6	2.77696200	0.92743900	-1.08099800
6	2.87402800	-0.51542600	-0.58883800
1	3.87865600	-0.72257100	-0.19028200
1	-0.08263700	0.37897300	-2.91085200
1	3.19181600	1.66906900	-0.39346900
1	1.30673900	2.25553900	-2.00430700
8	1.92748500	-0.61877700	0.42766400
6	5.37039900	0.59389200	-1.77057500
6	4.32145500	1.26618000	-2.44285000
6	4.16058500	0.51417000	-3.68596100
6	4.99567000	-0.54603200	-3.64417700
8	5.72897100	-0.50556500	-2.44107600
1	4.19028200	2.34291200	-2.35556900
1	3.46043300	0.73710100	-4.48687200
8	5.90654300	0.86851300	-0.63693900
6	5.28777600	-1.68633400	-4.53993600
1	5.08251200	-2.64330700	-4.03677000
1	4.65077600	-1.62444100	-5.43194100
1	6.34336400	-1.68410500	-4.85785400
14	7.21432600	0.24959000	0.38431100
6	6.72026000	0.96481800	2.06370000
1	7.04936700	2.02046400	2.01581500
6	8.72255500	1.10924900	-0.36555000
1	8.35435900	2.14006300	-0.53182700
6	7.24103200	-1.63995100	0.25008300
1	7.17206000	-1.83607900	-0.83416500
6	7.47798500	0.29191200	3.22275300
1	7.27238900	0.81364900	4.17162700
1	7.16275300	-0.75463800	3.35566300
1	8.56907600	0.29440300	3.07728500
6	5.20206300	0.96309700	2.31055500
1	4.96970300	1.41635600	3.28813800
1	4.66393100	1.53498500	1.54249600
1	4.77917900	-0.05332800	2.31727900
6	9.11752500	0.52702100	-1.73272100
1	8.27141900	0.49198100	-2.43618900
1	9.91084600	1.13370300	-2.19941400
1	9.50732800	-0.49900000	-1.63841500
6	9.92625200	1.20084100	0.58710500
1	9.67211500	1.71109200	1.52866400

1	10.32810900	0.20793300	0.84234000
1	10.74506500	1.77046600	0.11748500
6	8.57660100	-2.22421500	0.74893300
1	9.44136000	-1.83246400	0.19434200
1	8.74210700	-2.01656600	1.81761800
1	8.58042700	-3.32016500	0.6286910
6	6.05674600	-2.33975200	0.93620600
1	5.08305300	-1.94382900	0.61782400
1	6.06244600	-3.41905400	0.71042500
1	6.10589100	-2.23903900	2.03124000

TS0-*Si*-Cα

E = -5944.82335317 G = -5943.299493

6	1.70228500	-3.32842300	1.62983100
6	4.22250500	-1.22135200	0.27118800
6	3.47669200	-0.07077100	0.73081400
6	3.84261200	0.15455500	2.10151000
6	4.75953400	-0.84950500	2.50161200
6	4.98647100	-1.69666700	1.38499600
15	2.25830000	1.09715400	-0.00749400
15	-0.17073200	-1.43422200	0.40469300
6	2.45366300	1.13681200	-1.85009200
6	3.06969600	2.68041000	0.45921000
6	2.29285700	3.82057900	0.66522100
6	2.90125200	5.05598000	0.94598000
6	4.29542600	5.11859900	1.01216200
6	5.09894900	3.98395800	0.79629400
6	4.47266300	2.76772200	0.51569500
6	1.83931600	0.16119800	-2.64932800
6	1.87915400	0.23976800	-4.04761500
6	2.52645600	1.33328000	-4.64113000
6	3.12969900	2.33041500	-3.86930900
6	3.09011800	2.21703600	-2.47244000
26	2.96512700	-1.67953600	1.88988900
6	0.98085700	-2.09207700	1.64117000
6	2.40544400	-3.44426500	2.85810800
6	1.21856400	-1.44472400	2.91926000
6	2.10578400	-2.29936300	3.64928400
1	1.20611800	3.73790100	0.64181000
1	5.08220500	1.88373900	0.32176200
1	1.30399700	-0.66276300	-2.18623200
1	3.55197400	2.99989300	-1.87349000
1	5.18331900	-0.96248200	3.49740800
1	5.64339700	-2.56249900	1.36535400
1	3.07724500	-4.25443400	3.13559200
1	2.52382400	-2.09503400	4.62942900
1	1.71074500	-4.05264300	0.82180000
1	4.77787400	6.07526600	1.23498400
1	2.54913200	1.41263900	-5.73163900
45	-0.09833000	0.84775800	0.41892500
6	-1.74075200	-2.11905500	1.11215300
6	-2.57661400	-1.32943300	1.90360300
6	-1.98956700	-3.49754600	1.00289000
6	-3.65242500	-1.90207600	2.60212800
1	-2.38500900	-0.26255700	1.99162900
6	-3.05932900	-4.09157600	1.67853400
1	-1.33843900	-4.12187900	0.38772800
6	-3.87387600	-3.27680100	2.48172200
1	-4.71174100	-3.73035100	3.01940100
6	-0.04309300	-2.40062600	-1.14773400
6	1.10699100	-3.06229500	-1.59266900
6	-1.18831200	-2.37966000	-1.96413000
6	1.11228700	-3.73839400	-2.82581000
1	2.01552700	-3.04606800	-0.99508300
6	-1.20919400	-3.04371000	-3.19439600
1	-2.08700400	-1.86132500	-1.62603200
6	-0.05421400	-3.72878000	-3.59837000
1	-0.05567200	-4.25061400	-4.55925100
6	-4.53583400	-1.05485300	3.48163000
1	-5.52598300	-1.51283500	3.61914000
1	-4.09559700	-0.93446800	4.48493600
1	-4.67462100	-0.04568900	3.06525100
6	-3.34315600	-5.56640300	1.55099400
1	-3.38994700	-6.05091700	2.53933800
1	-4.31570300	-5.74360000	1.06147500
1	-2.57128100	-6.07709600	0.95784200
6	2.35715200	-4.42026800	-3.32723600
1	2.80813000	-5.06578900	-2.56099600
1	2.14819600	-5.02820600	-4.21861800
1	3.12934200	-3.68120800	-3.59491800
6	-2.44674700	-3.03949300	-4.05574000
1	-2.19723800	-3.18045800	-5.11752900
1	-3.13144200	-3.85555000	-3.76866300
1	-3.00616500	-2.09781100	-3.95354400
6	3.79712000	3.52277700	-4.50374700
1	3.78260100	3.46216900	-5.60137100
1	3.29223600	4.45646100	-4.20560200
1	4.84730100	3.60943700	-4.18119200
6	1.22941100	-0.82048000	-4.89782500
1	1.81945300	-1.75115200	-4.89469200
1	0.23279000	-1.08517100	-4.51690800
1	1.13031100	-0.49227500	-5.94255800
6	6.60128900	4.08382400	0.87620200
1	7.08381900	3.14759200	0.56009400
1	6.98450800	4.89856600	0.24112700
1	6.92914600	4.29856300	1.90716400
6	2.04119000	6.27603100	1.15906700
1	2.63223600	7.14175200	1.49095100
1	1.52379700	6.55590600	0.22614600
1	1.25947800	6.08260300	1.91120400
1	3.46377000	0.95073600	2.72954800
6	0.56553200	-0.15423500	3.37607800
1	0.30777800	0.38808400	2.43224800
6	4.48617900	-1.61769500	-1.16990300
1	3.54756800	-1.49975300	-1.73277100
7	5.39118300	-0.60638800	-1.71985600
6	4.93122500	-3.07854900	-1.34374700
6	4.47653600	-4.10284600	-0.49850400
6	5.76610700	-3.44220700	-2.41176900
6	4.83498400	-5.43642700	-0.71071800
1	3.83947300	-3.85069800	0.34406500
6	6.12421000	-4.77478400	-2.63196000
1	6.14447700	-2.68011100	-3.08865900
6	5.65966000	-5.78190300	-1.78318600
1	4.46642600	-6.20857900	-0.03061700
1	6.77575100	-5.02454800	-3.47264200
1	5.94282700	-6.82310700	-1.95255500
6	5.32093900	-0.28892000	-3.12785500
1	6.04124100	-0.85175000	-3.75910300
1	4.31372700	-0.48570300	-3.51151800
1	5.53153700	0.78236400	-3.28500300
6	6.72049100	-0.49786900	-1.16266800
1	7.14897900	0.47911800	-1.44176300
1	6.69163100	-0.54284900	-0.06551900
1	7.42484700	-1.28132600	-1.51571400
7	-0.72570900	-0.39054900	4.02183400
6	1.45328200	0.82923500	4.13054400
6	1.56818800	2.13889500	3.64110300
6	2.11892400	0.49986400	5.32111100
6	2.38605000	3.07457800	4.27759900
1	1.01989100	2.42817300	2.74339100
6	2.93052700	1.43744100	5.96563300
1	2.01343200	-0.49226000	5.75847200
6	3.07999700	2.72356500	5.43798200

1	2.48700800	4.07547300	3.85258400
1	3.45097800	1.15968500	6.88533800
1	3.72601200	3.44985600	5.93639400
6	-1.44427300	0.84007000	4.29085800
1	-2.48696400	0.60722000	4.55044800
1	-1.01226900	1.43568100	5.12048700
1	-1.44170600	1.47750900	3.39216200
6	-0.77449700	-1.33517200	5.11715600
1	-0.31986000	-0.95807700	6.05819700
1	-1.82743600	-1.57546100	5.33489100
1	-0.27653100	-2.27312300	4.83843000
6	-1.03968100	3.67207000	-1.01998300
6	-0.75454500	4.97194500	-1.42469500
1	-0.95617000	5.80447900	-0.74518000
6	-0.21368400	5.21283100	-2.69589200
1	0.01414800	6.23427100	-3.00898200
6	0.03450200	4.14157000	-3.55710100
1	0.45500800	4.32013300	-4.54913200
6	-0.23921700	2.83271500	-3.14786700
1	-0.03498400	1.99934900	-3.81898600
6	-0.76430100	2.58337800	-1.87635500
6	-1.08584100	1.21947700	-1.40589200
6	-2.11772500	1.03728200	-0.41253000
6	-2.60040900	2.19645800	0.28955700
6	-1.54563600	3.31992600	0.36187000
1	-1.96316600	4.21379800	0.86219500
1	-0.92420700	0.42150700	-2.13410200
1	-3.13860200	2.01042000	1.22462200
1	-2.70608100	0.11935400	-0.39550900
8	-0.49675600	2.78423300	1.10105800
6	-4.91922800	1.82302200	-0.81030400
6	-4.20442500	3.04160600	-0.64903100
6	-3.79201500	3.38584400	-2.01165400
6	-4.06356900	2.32952200	-2.80341300
8	-4.75502300	1.35687000	-2.04360100
1	-4.46711700	3.76021900	0.12613700
1	-3.27303400	4.28671400	-2.32038400
8	-5.55362600	1.11962300	0.05228800
6	-3.77537200	1.97016900	-4.20858200
1	-4.69553600	1.73538700	-4.76782700
1	-3.26340800	2.80836900	-4.69883700
1	-3.11279900	1.08984600	-4.25980300
14	-6.72985000	-0.21441000	-0.13534900
6	-7.65221400	-0.10984900	1.51229000
1	-7.02376000	-0.69386600	2.20716400
6	-5.68400900	-1.77904000	-0.27937300
1	-4.87480000	-1.61315100	0.45170800
6	-7.71086200	0.21683000	-1.69266500
1	-6.94385800	0.27499200	-2.48411600
6	-9.03265300	-0.78843800	1.45009100
1	-9.47692600	-0.84556000	2.45716200
1	-9.73247300	-0.21625700	0.82124000
1	-8.98897800	-1.81377600	1.05225300
6	-7.76319100	1.31604700	2.07560800
1	-8.26654000	1.30103700	3.05634600
1	-6.77779700	1.78491500	2.20882100
1	-8.35585700	1.96935500	1.41573000
6	-5.03124200	-1.98168700	-1.65363700
1	-4.43497500	-1.11167200	-1.96351000
1	-4.36232900	-2.85644000	-1.62695200
1	-5.77906400	-2.16498600	-2.44229300
6	-6.44194900	-3.03963500	0.16920200
1	-6.82173600	-2.95353900	1.19801700
1	-7.29700700	-3.26655800	-0.48788700
1	-5.76930000	-3.91168000	0.14469600
6	-8.69330200	-0.89872200	-2.09325400
1	-8.19371800	-1.87265300	-2.21008000
1	-9.49904500	-1.02430400	-1.35510100
1	-9.17085900	-0.65868400	-3.05748800
6	-8.40724900	1.58394200	-1.60728200
1	-7.71019700	2.39807400	-1.35113300
1	-8.87502100	1.84402000	-2.57108400
1	-9.20578700	1.58415400	-0.84870100

TS0-*Re*- Cα

E = -5944.81596737 G = -5943.294337

6	-3.67396600	2.57849700	1.30494700
6	-4.20779800	-0.93278000	1.18926700
6	-2.78992800	-1.13921500	1.39958100
6	-2.56247700	-1.07846900	2.81865600
6	-3.79351300	-0.81241000	3.47042900
6	-4.79943700	-0.71506800	2.47344800
15	-1.32823600	-1.44483900	0.32516100
15	-1.33633100	2.04933400	-0.40860100
6	-1.86906100	-2.17311500	-1.28409900
6	-0.62495900	-2.92427200	1.17450900
6	0.72482300	-3.23240500	0.99334900
6	1.28429800	-4.38360300	1.57064900
6	0.46158100	-5.22559100	2.32333300
6	-0.90469400	-4.94939400	2.50475000
6	-1.43642900	-3.79574400	1.92240700
6	-2.28130600	-1.33273000	-2.32663400
6	-2.57368000	-1.84696800	-3.59529300
6	-2.41834500	-3.22320100	-3.81402900
6	-1.99630500	-4.08273300	-2.79444200
6	-1.73045800	-3.54453600	-1.52737400
26	-3.30018200	0.69753400	2.14521100
6	-2.31560700	2.16844900	1.11538100
6	-3.94119400	2.60249100	2.69996900
6	-1.72404100	1.96137600	2.42385900
6	-2.74860000	2.23502600	3.38583900
1	1.35606300	-2.54373900	0.43881800
1	-2.49648300	-3.58455300	2.05083300
1	-2.36054200	-0.26167500	-2.16231800
1	-1.39033100	-4.21036600	-0.73527400
1	-3.92694100	-0.67243100	4.54121500
1	-5.85284700	-0.50785000	2.64092700
1	-4.89947100	2.83346300	3.16119500
1	-2.65379200	2.13367200	4.46230700
1	-4.37601200	2.83934500	0.52002900
1	0.88787300	-6.12357100	2.78110700
1	-2.62523800	-3.63234900	-4.80701300
45	0.19076900	0.35148400	-0.19873700
6	-0.56955800	3.73401300	-0.40339300
6	0.77377500	3.92772900	-0.07611800
6	-1.39146100	4.84795900	-0.63239100
6	1.31799000	5.21749500	-0.00061400
1	1.40419700	3.06914300	0.13642900
6	-0.87892200	6.14774500	-0.55667800
1	-2.44589100	4.70737100	-0.87991700
6	0.48008000	6.31115400	-0.24583200
1	0.89439700	7.32282100	-0.19378500
6	-2.43781400	2.19383100	-1.86558900
6	-3.77043700	1.77838500	-1.88601500
6	-1.85143000	2.68499000	-3.04701800
6	-4.54016600	1.86929200	-3.05967400
1	-4.21583400	1.36298600	-0.98751500
6	-2.59205000	2.78998400	-4.22673700
1	-0.81179100	3.01926900	-3.04173200
6	-3.93752100	2.38609000	-4.20938500
1	-4.52590100	2.46194300	-5.12815300
6	2.76810100	5.42208900	0.36233100
1	3.22767500	6.21989600	-0.24117800
1	2.87563800	5.71589400	1.42034000

1	3.35386000	4.50182000	0.21815400
6	-1.75536300	7.34823300	-0.80962600
1	-1.76707400	8.02282700	0.06187700
1	-1.38769200	7.93648100	-1.66639200
1	-2.79274500	7.05350000	-1.02317100
6	-5.97196100	1.40078700	-3.07694600
1	-6.59895500	1.98782800	-2.38863100
1	-6.40576700	1.47873100	-4.08366600
1	-6.05422400	0.35361500	-2.74689100
6	-1.97247400	3.32074900	-5.49375800
1	-2.01598600	2.57060900	-6.30040000
1	-2.51091200	4.21170500	-5.85515800
1	-0.91943400	3.59899700	-5.34445800
6	-1.79144400	-5.55519000	-3.04189500
1	-2.16094900	-5.85741700	-4.03233300
1	-0.72051700	-5.81359100	-2.98590300
1	-2.30784700	-6.16425400	-2.28311300
6	-3.03941300	-0.93905900	-4.70345200
1	-4.10713100	-0.68838900	-4.58828600
1	-2.49416800	0.01533400	-4.69793300
1	-2.91477400	-1.40992100	-5.68939600
6	-1.77005200	-5.87431700	3.32286300
1	-2.83195800	-5.59652800	3.25704500
1	-1.66856800	-6.91951100	2.98960400
1	-1.48234900	-5.84659200	4.38731200
6	2.74722400	-4.68414900	1.36065300
1	3.08825900	-5.51830900	1.99076000
1	2.94496100	-4.94956800	0.30886000
1	3.36682600	-3.80227400	1.58822100
1	-1.60299500	-1.20068000	3.30589100
6	-0.26788500	1.61592500	2.66340900
1	0.05451300	1.06545700	1.74178000
6	-5.01399900	-1.15981600	-0.07379900
1	-4.55553800	-0.57209000	-0.88542300
7	-4.87616800	-2.54426900	-0.49959500
6	-6.45034100	-0.64692200	0.09782100
6	-6.70040000	0.72780200	0.22676600
6	-7.54295500	-1.52076200	0.15667700
6	-7.99487600	1.21786800	0.39942400
1	-5.86445000	1.42394600	0.21705100
6	-8.84463500	-1.03639500	0.32669200
1	-7.38387600	-2.59373100	0.07239700
6	-9.07768400	0.33422200	0.44683500
1	-8.15980300	2.29358200	0.50005400
1	-9.67990200	-1.73944200	0.36691300
1	-10.09367800	0.71248300	0.57984000
6	-5.33286900	-2.85312000	-1.83193800
1	-6.42408100	-3.04479000	-1.90863300
1	-5.09262600	-2.02248500	-2.51059500
1	-4.80811700	-3.74626900	-2.20555800
6	-4.97354900	-3.63609700	0.43920600
1	-4.29301600	-4.45137900	0.13290700
1	-4.67388000	-3.31087000	1.44333200
1	-5.98952100	-4.07643000	0.52608900
7	0.57392000	2.80739100	2.68589400
6	0.02187100	0.62771300	3.78726800
6	0.71629000	-0.55322400	3.48336700
6	-0.33397800	0.87157300	5.12223200
6	0.98239300	-1.50317500	4.47172300
1	1.04748500	-0.73877700	2.46010400
6	-0.05924900	-0.07320500	6.11501300
1	-0.82971000	1.80156100	5.40065400
6	0.58575400	-1.27093400	5.79114500
1	1.49131200	-2.43069600	4.19929900
1	-0.35366700	0.12724900	7.14799400
1	0.78616100	-2.01309600	6.56733600
6	1.98684300	2.50211300	2.74250100

1	2.56831500	3.39862700	2.47843800
1	2.33054500	2.15463100	3.73873800
1	2.22389500	1.70619300	2.01977000
6	0.18248100	3.92144500	3.52033200
1	0.35473600	3.75529000	4.60574500
1	0.76709600	4.80772400	3.22504200
1	-0.87746700	4.16304900	3.36744600
6	2.52423000	-1.49472300	-1.68804100
6	3.15902100	-2.72071200	-1.85536500
1	4.03354200	-2.96294600	-1.24799600
6	2.66727500	-3.64815500	-2.78508200
1	3.15996300	-4.61612000	-2.90431500
6	1.54315800	-3.33382200	-3.55124100
1	1.15252900	-4.05215500	-4.27527300
6	0.90417400	-2.10057800	-3.38800500
1	0.02232600	-1.86207400	-3.98153100
6	1.37990000	-1.17677000	-2.45171600
6	0.74073900	0.13788600	-2.23487900
6	1.53224600	1.22857200	-1.71752500
6	2.77696200	0.92743900	-1.08099800
6	2.87402800	-0.51542600	-0.58883800
1	3.87865600	-0.72257100	-0.19028200
1	-0.08263700	0.37897300	-2.91085200
1	3.19181600	1.66906900	-0.39346900
1	1.30673900	2.25553900	-2.00430700
8	1.92748500	-0.61877700	0.42766400
6	5.37039900	0.59389200	-1.77057500
6	4.32145500	1.26618000	-2.44285000
6	4.16058500	0.51417000	-3.68596100
6	4.99567000	-0.54603200	-3.64417700
8	5.72897100	-0.50556500	-2.44107600
1	4.19028200	2.34291200	-2.35556900
1	3.46043300	0.73710100	-4.48687200
8	5.90654300	0.86851300	-0.63693900
6	5.28777600	-1.68633400	-4.53993600
1	5.08251200	-2.64330700	-4.03677000
1	4.65077600	-1.62444100	-5.43194100
1	6.34336400	-1.68410500	-4.85785400
14	7.21432600	0.24959000	0.38431100
6	6.72026000	0.96481800	2.06370000
1	7.04936700	2.02046400	2.01581500
6	8.72255500	1.10924900	-0.36555000
1	8.35435900	2.14006300	-0.53182700
6	7.24103200	-1.63995100	0.25008300
1	7.17206000	-1.83607900	-0.83416500
6	7.47798500	0.29191200	3.22275300
1	7.27238900	0.81364900	4.17162700
1	7.16275300	-0.75463800	3.35566300
1	8.56907600	0.29440300	3.07728500
6	5.20206300	0.96309700	2.31055500
1	4.96970300	1.41635600	3.28813800
1	4.66393100	1.53498500	1.54249600
1	4.77917900	-0.05332800	2.31727900
6	9.11752500	0.52702100	-1.73272100
1	8.27141900	0.49198100	-2.43618900
1	9.91084600	1.13370300	-2.19941400
1	9.50732800	-0.49900000	-1.63841500
6	9.92625200	1.20084100	0.58710500
1	9.67211500	1.71109200	1.52866400
1	10.32810900	0.20793300	0.84234000
1	10.74506500	1.77046600	0.11748500
6	8.57660100	-2.22421500	0.74893300
1	9.44136000	-1.83246400	0.19434200
1	8.74210700	-2.01656600	1.81761800
1	8.58042700	-3.32016500	0.62869100
6	6.05674600	-2.33975200	0.93620600
1	5.08305300	-1.94382900	0.61782400

1	6.06244600	-3.41905400	0.71042500
1	6.10589100	-2.23903900	2.03124000

TS0-*Si*-Cγ

E = -5944.81910403 G = -5943.296634

6	3.51887500	2.20337700	-1.85836100
6	3.85108600	-1.29623100	-1.40463500
6	2.40854700	-1.41663700	-1.38572400
6	1.97417300	-1.49345200	-2.75560600
6	3.10830700	-1.39697100	-3.60178200
6	4.25609300	-1.26650100	-2.77602900
15	1.11872600	-1.50051900	-0.08105200
15	1.39486600	2.05063600	0.18896400
6	1.86004100	-2.05985500	1.51457000
6	0.22323100	-3.02363900	-0.61516800
6	-1.05887800	-3.26730400	-0.11536000
6	-1.74000400	-4.45440100	-0.42756400
6	-1.11009300	-5.39686000	-1.24482400
6	0.18593000	-5.18669700	-1.74461700
6	0.84327900	-3.99722800	-1.41813900
6	2.45457200	-1.12509700	2.37189700
6	2.91356900	-1.49913900	3.64012500
6	2.73978900	-2.82812100	4.05127700
6	2.13805000	-3.77880200	3.22005700
6	1.70828200	-3.38290400	1.94578400
26	2.90733700	0.28068400	-2.41119700
6	2.17593000	1.93051300	-1.44419000
6	3.58763800	2.05164700	-3.26983500
6	1.39562700	1.62967700	-2.62762300
6	2.28899400	1.70861900	-3.74326600
1	-1.54575200	-2.50967200	0.48997200
1	1.85418000	-3.84196200	-1.78932100
1	2.54688200	-0.08756900	2.06384600
1	1.23230800	-4.11969900	1.30011700
1	3.08869600	-1.38551700	-4.68963800
1	5.28293800	-1.15640400	-3.11401300
1	4.48536600	2.14974500	-3.87716900
1	2.03297400	1.50142200	-4.77789600
1	4.34155200	2.48753300	-1.21103600
1	-1.63399900	-6.32400800	-1.49666700
1	3.07548200	-3.12622000	5.04860800
45	-0.21766700	0.42919500	0.41341700
6	0.68320800	3.75500700	0.05692500
6	-0.68723200	3.96930400	-0.11177700
6	1.55971400	4.84934000	0.02477100
6	-1.20106600	5.26313300	-0.27186400
1	-1.36638800	3.12172600	-0.13370500
6	1.07861100	6.15239300	-0.15048500
1	2.63414100	4.69205800	0.14188800
6	-0.30518400	6.33889100	-0.28808400
1	-0.69367500	7.35444500	-0.41330800
6	2.68355600	2.32057400	1.46174100
6	3.97878200	1.80668600	1.37159200
6	2.29713600	3.01507300	2.62276800
6	4.90881100	1.99874400	2.40812500
1	4.26622200	1.23185800	0.49643300
6	3.20030700	3.22302400	3.66860800
1	1.28842800	3.42572900	2.70302700
6	4.50392700	2.71635300	3.53690400
1	5.21777000	2.87166700	4.35075600
6	-2.68429600	5.49655100	-0.42411800
1	-3.06634200	6.16801800	0.36212100
1	-2.91931400	5.97188700	-1.39039900
1	-3.25099000	4.55587400	-0.37087200
6	2.01706600	7.33185400	-0.18697000
1	3.06209800	7.02168900	-0.04473900
1	1.94981200	7.86269700	-1.15067400
1	1.77002900	8.06260100	0.60025100
6	6.29897100	1.42652700	2.30776000
1	6.87367600	1.89938400	1.49634700
1	6.85722300	1.56610600	3.24402700
1	6.27356600	0.35013500	2.07864400
6	2.79593100	3.97123000	4.91259300
1	2.91397400	3.34156300	5.80957400
1	3.42657500	4.86261600	5.06119800
1	1.74857100	4.30179000	4.86457200
6	1.91229700	-5.19534200	3.68222500
1	2.45517100	-5.41184900	4.61349100
1	0.83978800	-5.37679900	3.86652400
1	2.23430800	-5.92198600	2.91988700
6	3.57124300	-0.49232000	4.54748000
1	4.62814000	-0.34058100	4.27138600
1	3.08612000	0.49137600	4.47666600
1	3.54882000	-0.82079900	5.59678200
6	0.84498800	-6.22016500	-2.62271100
1	1.90209500	-5.97909500	-2.80580200
1	0.79607900	-7.22308900	-2.16959100
1	0.34241400	-6.28477300	-3.60247000
6	-3.12063200	-4.68971400	0.13081100
1	-3.59468100	-5.57850300	-0.31002200
1	-3.08394400	-4.83010100	1.22362100
1	-3.77158400	-3.82234700	-0.05542400
1	0.94684800	-1.59376000	-3.08362900
6	-0.10501400	1.42464700	-2.63676300
1	-0.36229200	1.06418300	-1.60344400
6	4.82128000	-1.42911400	-0.24868200
1	4.52261500	-0.71334900	0.53434600
7	4.67346700	-2.73674300	0.37129500
6	6.24120800	-1.04916400	-0.68741900
6	6.56501300	0.28978100	-0.95144800
6	7.23869500	-2.01517100	-0.86985500
6	7.84133900	0.65801200	-1.37765500
1	5.79909200	1.05463800	-0.84117700
6	8.52177200	-1.65326000	-1.29407500
1	7.01790300	-3.06418300	-0.68248600
6	8.82980400	-0.31600500	-1.54908200
1	8.06578300	1.70865900	-1.57817200
1	9.28341900	-2.42523700	-1.42628300
1	9.83152400	-0.03343600	-1.88028800
6	5.33853200	-2.91514600	1.63815100
1	6.41915400	-3.15783000	1.55562300
1	5.24723900	-1.99929400	2.23981900
1	4.85248700	-3.72792500	2.19978000
6	4.54316500	-3.93947500	-0.41604600
1	3.86482400	-4.64735800	0.09438000
1	4.11039500	-3.71405200	-1.39858600
1	5.49927800	-4.47613100	-0.59267900
7	-0.80896300	2.69287400	-2.77469500
6	-0.62604200	0.31581300	-3.54376400
6	-1.31828700	-0.76261800	-2.97362700
6	-0.47285100	0.35479000	-4.93777600
6	-1.78328000	-1.81466700	-3.76657400
1	-1.49728300	-0.78944900	-1.89684900
6	-0.94668200	-0.69137000	-5.73390000
1	0.01706500	1.20472800	-5.41420300
6	-1.59076800	-1.78730800	-5.14986200
1	-2.28789900	-2.65955500	-3.29196900
1	-0.81079700	-0.65112600	-6.81729300
1	-1.94884200	-2.60908900	-5.77422300
6	-2.24578100	2.56666300	-2.73930800
1	-2.69795000	3.55196600	-2.55055300
1	-2.68301200	2.16660000	-3.67714800
1	-2.54197200	1.88734600	-1.92420700
6	-0.32222000	3.70203400	-3.68759900

1	-0.50650000	3.47438500	-4.76004800
1	-0.83072200	4.65285800	-3.46097000
1	0.75334100	3.86734300	-3.54700100
6	-2.36068300	-1.04465600	2.49057400
6	-3.00763700	-2.19690400	2.92843400
1	-3.96541900	-2.47681400	2.48641400
6	-2.42259200	-3.00433800	3.91201400
1	-2.92985000	-3.91261000	4.24548000
6	-1.18684300	-2.64867500	4.45960000
1	-0.72366100	-3.27564200	5.22452600
6	-0.53480700	-1.49164000	4.02671100
1	0.43084200	-1.21884100	4.45062600
6	-1.10908000	-0.68607800	3.03548600
6	-0.46322800	0.54120700	2.53440700
6	-1.28627400	1.58750600	1.98106100
6	-2.58843800	1.26360300	1.50969300
6	-2.82769300	-0.22916800	1.30401200
1	-3.88785700	-0.44396800	1.09923900
1	0.46189200	0.83023700	3.03760800
1	-3.01979300	1.90667100	0.73954000
1	-0.97229800	2.62821900	2.04381200
8	-2.04998700	-0.55127600	0.19002000
6	-5.89781900	0.77580800	2.12303500
6	-5.45129900	0.22429800	3.32759100
6	-4.36181900	1.00412900	3.72651300
6	-4.15103100	2.01022600	2.75932600
8	-5.18376600	1.87249700	1.81686500
1	-5.84495000	-0.67261700	3.79529900
1	-3.74077700	0.85925300	4.60858900
8	-6.79778100	0.37416000	1.28694600
6	-3.68530300	3.41315500	2.98742700
1	-4.48322200	4.02175700	3.44265100
1	-2.82449500	3.40406100	3.67004400
1	-3.37785000	3.89461200	2.0479810
14	-6.71661600	-0.27727200	-0.35097500
6	-6.08680600	-2.05947000	-0.16282300
1	-4.98467500	-1.95503100	-0.15707200
6	-5.46755400	0.77104900	-1.31138700
1	-4.56620000	0.79091800	-0.6738620
6	-8.51224100	-0.05151300	-0.87756600
1	-8.70080900	1.01684300	-0.65953900
6	-6.46488400	-2.93645100	-1.37062800
1	-5.97101900	-3.91970900	-1.30033900
1	-7.54992800	-3.12070600	-1.40359100
1	-6.17272700	-2.49025500	-2.33220300
6	-6.50749300	-2.74037700	1.14805600
1	-6.04978200	-3.74063000	1.22709700
1	-6.20581100	-2.16486300	2.03343100
1	-7.59851700	-2.87434000	1.20222400
6	-5.94219300	2.21800600	-1.51003800
1	-6.19905000	2.69999800	-0.55464700
1	-5.15489200	2.82445200	-1.98826800
1	-6.82834400	2.26871800	-2.16288600
6	-5.05225200	0.10551700	-2.63619000
1	-4.55311400	-0.86006900	-2.47186900
1	-5.90956100	-0.06119800	-3.30691300
1	-4.33547900	0.73957800	-3.17855200
6	-8.75595000	-0.27213900	-2.37946300
1	-8.13959900	0.39100900	-3.00486100
1	-8.54300900	-1.30888800	-2.68283400
1	-9.81060600	-0.07135800	-2.63029700
6	-9.48035500	-0.87963800	-0.01676300
1	-9.31974400	-0.71376000	1.05934700
1	-10.52599100	-0.61414300	-0.24379000
1	-9.37114600	-1.95842500	-0.21307500

TS0-*Re*-Cγ

E = -5944.81900091 G = -5943.296731

6	3.50816000	2.19339600	-1.92495000
6	3.95047500	-1.27100000	-1.28229300
6	2.51237700	-1.44603500	-1.30297300
6	2.12942200	-1.60598400	-2.68057700
6	3.28596000	-1.50347500	-3.49450800
6	4.39936300	-1.29052100	-2.64028200
15	1.17724200	-1.50507400	-0.03912800
15	1.36356700	2.06081800	0.09551700
6	1.88293600	-1.98744500	1.59642500
6	0.32524000	-3.06114900	-0.54891200
6	-0.99606300	-3.26941900	-0.14730500
6	-1.67260400	-4.45498600	-0.47520800
6	-0.99236600	-5.43738900	-1.19909900
6	0.34667500	-5.26601400	-1.59206800
6	0.99485500	-4.07336300	-1.25943400
6	2.44659900	-1.01059400	2.42750400
6	2.88008000	-1.32695800	3.72001800
6	2.71192600	-2.64017500	4.18167100
6	2.13866200	-3.63110600	3.37785800
6	1.73368400	-3.29276600	2.07887500
26	2.98392400	0.22120100	-2.39262300
6	2.16934800	1.88676600	-1.52124100
6	3.61141700	1.97638300	-3.32529700
6	1.42438900	1.49670200	-2.70332800
6	2.33697000	1.55939900	-3.80463500
1	-1.52138900	-2.47872000	0.37919500
1	2.03436000	-3.94266400	-1.55493200
1	2.53596000	0.01454000	2.07851900
1	1.27718400	-4.05961700	1.45417400
1	3.30223600	-1.54455800	-4.58170900
1	5.43229900	-1.15913100	-2.95071100
1	4.51608600	2.08186000	-3.92095700
1	2.11332400	1.28866600	-4.83179400
1	4.30541000	2.54265300	-1.27732400
1	-1.50974700	-6.36470200	-1.46367800
1	3.02874900	-2.89308000	5.19747400
45	-0.21554300	0.41772100	0.35120700
6	0.62423800	3.74589500	-0.11017600
6	-0.74646300	3.92629500	-0.30746400
6	1.47980100	4.85631600	-0.16206500
6	-1.28223900	5.20357200	-0.52184800
1	-1.40815000	3.06504600	-0.30675600
6	0.97624100	6.14290400	-0.38615200
1	2.55460200	4.72466200	-0.01916100
6	-0.40863000	6.29664800	-0.55530500
1	-0.81482300	7.29973700	-0.71868700
6	2.62058800	2.40428200	1.38259300
6	3.93611200	1.93972200	1.33311600
6	2.18151100	3.10998400	2.51829900
6	4.83361900	2.19059100	2.38636400
1	4.26674800	1.36044800	0.47666100
6	3.05086400	3.37597100	3.57899000
1	1.15652400	3.48372400	2.56611600
6	4.37540800	2.91641200	3.48890000
1	5.06360500	3.11720400	4.31478500
6	-2.76452800	5.39124700	-0.73459800
1	-3.14138100	6.27412100	-0.19500600
1	-2.99780700	5.54442400	-1.80193700
1	-3.33433000	4.51249700	-0.39761300
6	1.89052600	7.34070000	-0.43682800
1	2.94194200	7.05300100	-0.29438100
1	1.80983800	7.86153700	-1.40480600
1	1.63122900	8.07332400	0.34493200
6	6.24659500	1.67067900	2.33165400
1	6.81414300	2.12608200	1.50559000

1	6.78457700	1.87661000	3.26752800
1	6.26685200	0.58437200	2.15444300
6	2.58917500	4.13508400	4.79611600
1	2.70216500	3.52696900	5.70856900
1	3.18710000	5.04887700	4.94365700
1	1.53354500	4.43072200	4.71336500
6	1.91543900	-5.02912000	3.89425100
1	2.44332800	-5.20247200	4.84301700
1	0.84115400	-5.21127300	4.06735000
1	2.25611600	-5.78401400	3.16840200
6	3.50569000	-0.27584100	4.59926600
1	4.55871400	-0.10179300	4.32173700
1	2.99308500	0.69116200	4.49741000
1	3.48800600	-0.57329500	5.65778800
6	1.05987500	-6.34742000	-2.36381200
1	1.02751300	-7.31001900	-1.82828700
1	0.58700800	-6.51057000	-3.34652100
1	2.11462200	-6.09025600	-2.53791900
6	-3.10609600	-4.63393500	-0.04093400
1	-3.54546100	-5.55695900	-0.44572600
1	-3.18116900	-4.67280700	1.05807900
1	-3.72660900	-3.78522600	-0.37127700
1	1.11916100	-1.76348200	-3.03697500
6	-0.06008600	1.19477200	-2.72306700
1	-0.29698800	0.82770400	-1.69056300
6	4.89097400	-1.32055900	-0.09504500
1	4.55008800	-0.58011800	0.64644700
7	4.76533000	-2.60343700	0.57936700
6	6.31109500	-0.91730600	-0.51347900
6	6.59677800	0.41183800	-0.85965700
6	7.34832100	-1.85467600	-0.59492600
6	7.87420300	0.79837200	-1.26561200
1	5.80005800	1.15234500	-0.83294800
6	8.63281200	-1.47402400	-0.99817000
1	7.15777800	-2.89640800	-0.34495400
6	8.90275000	-0.14648200	-1.33373700
1	8.06805300	1.84048400	-1.53183200
1	9.42544200	-2.22403000	-1.05074600
1	9.90547000	0.15083100	-1.64861100
6	5.39477400	-2.70676300	1.87265200
1	6.48093700	-2.93533300	1.83447300
1	5.27271100	-1.76350700	2.42399100
1	4.90462600	-3.49811600	2.46071600
6	4.69483800	-3.84329300	-0.15619400
1	4.02473200	-4.54927100	0.36767100
1	4.28407900	-3.67592500	-1.15960800
1	5.67194900	-4.35598300	-0.28245900
7	-0.85509700	2.40929800	-2.86717300
6	-0.50465000	0.04615700	-3.62083600
6	-1.17133500	-1.04361600	-3.04167800
6	-0.31290900	0.05318400	-5.01071700
6	-1.56987500	-2.13557600	-3.81590600
1	-1.37731200	-1.04562700	-1.97016400
6	-0.72081100	-1.03332500	-5.78951800
1	0.15737900	0.90729000	-5.49844900
6	-1.33662200	-2.13860600	-5.19320900
1	-2.05462900	-2.98608900	-3.33128000
1	-0.55427400	-1.01716200	-6.86925100
1	-1.64139600	-2.99185500	-5.80344400
6	-2.27654000	2.17423600	-2.75533700
1	-2.79772600	3.12970400	-2.59225500
1	-2.71982400	1.69299300	-3.65183500
1	-2.48002400	1.51571700	-1.89606000
6	-0.49925900	3.36887800	-3.88846100
1	-0.77341900	3.05567200	-4.91908800
1	-1.02288000	4.31616100	-3.68109700
1	0.57785000	3.57818400	-3.86186200

6	-2.37723100	-1.05008800	2.41654300
6	-3.03375900	-2.17407500	2.90845700
1	-3.99328800	-2.47002900	2.47952300
6	-2.46159700	-2.92748100	3.94322400
1	-2.97494900	-3.81455700	4.32106500
6	-1.23341900	-2.54221500	4.48499900
1	-0.78046900	-3.12595400	5.28930100
6	-0.57748100	-1.40549700	4.00303900
1	0.37648000	-1.10382300	4.43343600
6	-1.13856700	-0.65363400	2.96572400
6	-0.51220300	0.57858500	2.44554000
6	-1.34932100	1.59582200	1.85279300
6	-2.65886800	1.24264300	1.40600900
6	-2.84511500	-0.27019400	1.20906600
1	-3.89168900	-0.51073600	0.98724500
1	0.39283900	0.90251100	2.96425500
1	-3.10947000	1.84556100	0.61499800
1	-1.05825400	2.64431100	1.90832000
8	-2.03718500	-0.57194900	0.11515700
6	-5.94269700	1.69508600	1.47008200
6	-5.51871800	3.03478800	1.45406000
6	-4.43275100	3.10087400	2.32525900
6	-4.15234100	1.79641600	2.80540000
8	-5.18958700	0.98071700	2.31412700
1	-5.96138300	3.82255300	0.85097800
1	-3.83237200	3.97920100	2.55972000
8	-6.87438900	1.10158100	0.80447500
6	-3.63340600	1.42798000	4.15930200
1	-3.47480500	0.34650200	4.23890200
1	-2.66962800	1.92918800	4.32816800
1	-4.33557200	1.74380600	4.94739700
14	-6.81925300	-0.28172900	-0.30041400
6	-6.41792000	-1.84657100	0.69144500
1	-5.31656300	-1.84696000	0.76883500
6	-5.42629000	0.20592100	-1.48841900
1	-4.61552700	0.56790700	-0.82961900
6	-8.54832800	-0.17770200	-1.04138600
1	-8.62284000	0.88162000	-1.35055800
6	-6.81628100	-3.11708800	-0.08207000
1	-6.43447600	-4.01358900	0.43361200
1	-7.91042700	-3.22151700	-0.14600200
1	-6.41739200	-3.13500200	-1.10757300
6	-6.98830500	-1.86460100	2.11784200
1	-6.68564000	-2.78929900	2.63816000
1	-6.63237100	-1.01486900	2.71578400
1	-8.08881100	-1.84054100	2.11555700
6	-5.84607700	1.37514800	-2.39319800
1	-6.21254900	2.23655900	-1.81200300
1	-4.99842200	1.72061500	-3.00496300
1	-6.64734600	1.08236100	-3.08963300
6	-4.84282000	-0.96946000	-2.28721500
1	-4.41404400	-1.73793700	-1.62698700
1	-5.59760400	-1.44927100	-2.93104900
1	-4.02550100	-0.62442000	-2.93881700
6	-8.73032300	-1.04510000	-2.29849800
1	-7.97427800	-0.83207900	-3.06972500
1	-8.67315700	-2.11928300	-2.06642800
1	-9.71942600	-0.86368300	-2.75038500
6	-9.65238500	-0.43884600	-0.00439200
1	-9.53253700	0.18825000	0.89245900
1	-10.64575000	-0.22419500	-0.43175200
1	-9.66019900	-1.49202900	0.31893600

TS0'-*Si*-Cα

E = -5300.01076115 G = -5298.747321

6	1.24289500	-3.17034500	1.72294700
6	3.43418700	-0.59215000	0.60856100

6	2.42716300	0.38153300	0.95997200
6	2.57636900	0.66709600	2.36003100
6	3.62421600	-0.13487100	2.87918300
6	4.14323500	-0.91304000	1.81033900
15	1.10838200	1.30304200	0.06629800
15	-0.83323500	-1.66865100	0.28510500
6	1.47002300	1.38291900	-1.74830500
6	1.57691800	3.00369600	0.58903900
6	0.60082600	3.98763200	0.74502600
6	0.96545800	5.30494800	1.07534000
6	2.31962200	5.60789600	1.23486100
6	3.32177700	4.63397500	1.06733600
6	2.93697200	3.33221600	0.74179700
6	1.16164600	0.29633000	-2.58029400
6	1.32323100	0.37868800	-3.96918400
6	1.77236300	1.58632200	-4.52299700
6	2.05879400	2.69472500	-3.72085600
6	1.90970800	2.57716800	-2.33152800
26	2.11439600	-1.30210000	2.08459900
6	0.28941400	-2.10597300	1.63635000
6	1.81879900	-3.14382600	3.02150500
6	0.25573500	-1.42681200	2.91704100
6	1.21234400	-2.08410700	3.75480000
1	-0.45023200	3.71881800	0.62975100
1	3.70180900	2.56995700	0.58383900
1	0.77393700	-0.62350600	-2.15191500
1	2.12887500	3.44291700	-1.70901000
1	3.94424300	-0.16811800	3.91867300
1	4.95648900	-1.63089400	1.88165600
1	2.60646700	-3.80187200	3.38383100
1	1.47212500	-1.79836400	4.76908300
1	1.48540200	-3.87449900	0.93349900
1	2.61211100	6.63051200	1.49230600
1	1.88668900	1.66636100	-5.60770200
45	-1.18268300	0.59659500	0.24120500
6	-2.32366700	-2.64505800	0.80176600
6	-3.43901600	-2.03352400	1.38133500
6	-2.27808300	-4.04337600	0.70489200
6	-4.51888300	-2.79945400	1.84348600
1	-3.46060900	-0.95268500	1.49130500
6	-3.34011900	-4.83440100	1.15911700
1	-1.40676600	-4.53139000	0.26316900
6	-4.45512500	-4.19271200	1.71806300
1	-5.29442300	-4.79949600	2.07218300
6	-0.36001500	-2.58539500	-1.23098600
6	0.93901400	-2.99314500	-1.54899500
6	-1.39995400	-2.81334100	-2.15228100
6	1.20520800	-3.66189300	-2.75892600
1	1.76135900	-2.78740200	-0.86700900
6	-1.16248100	-3.47114000	-3.3604940
1	-2.41895000	-2.50506500	-1.90892500
6	0.14455700	-3.90217800	-3.63723300
1	0.34404300	-4.42453300	-4.57709900
6	-5.71023300	-2.14428000	2.49989700
1	-6.65920700	-2.59847600	2.16969400
1	-5.67410100	-2.26502700	3.59567300
1	-5.74648200	-1.06436500	2.29727100
6	-3.28646100	-6.33820700	1.06570900
1	-3.17631800	-6.79160400	2.06508200
1	-4.21066800	-6.74678300	0.62737500
1	-2.43920100	-6.67495400	0.45134800
6	2.60734700	-4.07709500	-3.11579900
1	3.08762700	-4.63901500	-2.30258700
1	2.62241700	-4.69524000	-4.02427600
1	3.24511200	-3.19650500	-3.29357700
6	-2.27329600	-3.70252200	-4.35268700
1	-2.12285700	-3.09666200	-5.26234400
1	-2.31257300	-4.75542200	-4.67307500
1	-3.25412900	-3.44024600	-3.92919100
6	2.49330900	4.00753300	-4.31854700
1	2.68960500	3.91974000	-5.39673500
1	1.71394100	4.77491100	-4.17610500
1	3.40734200	4.38467500	-3.83312000
6	1.00687000	-0.79995600	-4.85259000
1	1.78424800	-1.57708600	-4.77506600
1	0.06113100	-1.27755800	-4.55939200
1	0.93785600	-0.50281000	-5.90898000
6	4.77546200	4.99362100	1.24280700
1	5.43238000	4.15381800	0.97355900
1	5.05313600	5.85929100	0.62015200
1	4.99226000	5.26768000	2.28883100
6	-0.10744600	6.35042500	1.24869000
1	0.31632200	7.33876700	1.47763800
1	-0.71673100	6.44250100	0.33455500
1	-0.79738200	6.07784800	2.06451300
1	1.97259600	1.36473500	2.92784900
6	-0.70204200	-0.30636000	3.26694500
1	-0.91852500	0.20409400	2.29436800
6	3.92882800	-0.91137800	-0.78995400
1	3.05299300	-0.96895700	-1.45395400
7	4.67431700	0.26533300	-1.24301800
6	4.66391800	-2.25530700	-0.90408500
6	4.33670100	-3.35568700	-0.09649400
6	5.65236600	-2.43977400	-1.88344000
6	4.96687900	-4.59202200	-0.25953700
1	3.58243500	-3.24021200	0.67646700
6	6.28258000	-3.67505300	-2.05418600
1	5.93781600	-1.61240900	-2.52886000
6	5.94313000	-4.76054700	-1.24344400
1	4.69159200	-5.42736400	0.38930000
1	7.04827900	-3.78616200	-2.82552700
1	6.43878500	-5.72507100	-1.37372400
6	4.68777800	0.59301800	-2.65091300
1	5.56234800	0.18913300	-3.20407800
1	3.78061100	0.21773300	-3.13717500
1	4.70562700	1.68793000	-2.78412600
6	5.89710500	0.60856500	-0.55236700
1	6.15901500	1.65481400	-0.78211600
1	5.76773500	0.53326200	0.53586300
1	6.76804500	-0.02056900	-0.83498800
7	-1.99782700	-0.82472200	3.70121300
6	-0.15143000	0.80926300	4.14631700
6	-0.20179200	2.12681800	3.66734800
6	0.35531400	0.58387800	5.43504900
6	0.30417500	3.18579800	4.42351900
1	-0.63303600	2.32577200	2.68477200
6	0.85357200	1.64356200	6.19815600
1	0.36476200	-0.42086000	5.85737500
6	0.84311000	2.94607400	5.68999700
1	0.28351700	4.19742800	4.01230300
1	1.25386600	1.44940100	7.19609500
1	1.24425900	3.77015300	6.28426900
6	-2.99198900	0.21303100	3.87760500
1	-3.98918600	-0.24597600	3.95878100
1	-2.83121800	0.83860300	4.77927200
1	-2.98600900	0.88626900	3.00511800
6	-2.02321900	-1.83061500	4.74088000
1	-1.80894700	-1.43537000	5.75691700
1	-3.02631700	-2.28622300	4.76924500
1	-1.30572400	-2.63131100	4.51845500
6	-2.52996400	3.13971300	-1.44549400
6	-2.51482100	4.44368600	-1.93044000
1	-2.93570700	5.25058400	-1.32415200
6	-1.95644000	4.72258600	-3.18646000

1	-1.93821700	5.74853900	-3.56093200
6	-1.42052100	3.68507500	-3.95294000
1	-0.98246600	3.89503200	-4.93116500
6	-1.43284600	2.37381000	-3.46759600
1	-1.01160800	1.56711200	-4.06646800
6	-1.97572500	2.08950200	-2.21065400
6	-2.04853700	0.71632900	-1.67402900
6	-3.10133700	0.37627800	-0.74575200
6	-3.84967300	1.45120400	-0.13475200
6	-3.03401800	2.76187900	-0.07072500
1	-3.64716700	3.57796600	0.35655500
1	-1.68021400	-0.06516400	-2.34259000
1	-4.35065300	1.21398000	0.81015100
1	-3.51024900	-0.63372400	-0.73981500
8	-1.96525000	2.46167400	0.75994000
6	-6.03667000	0.60756400	-1.00547900
6	-5.45943400	1.89088500	-1.15909500
6	-5.08804400	1.91890300	-2.57862000
6	-5.23226400	0.67130300	-3.06683600
8	-5.82807500	-0.14519200	-2.05992500
1	-5.80403900	2.75242600	-0.58797400
1	-4.66572000	2.76440200	-3.11195800
8	-6.58129500	0.08569700	0.06011300
6	-4.90804000	-0.01061000	-4.33809600
1	-5.80361700	-0.44939200	-4.80653500
1	-4.46538900	0.71645900	-5.03164700
1	-4.17782600	-0.82031900	-4.17467700
1	-6.72997700	-0.87047000	-0.04850100

TS0'-*Re*- Cα

E = -5300.00817248 G = -5298.745322

6	-2.39743900	2.79793900	1.35065600
6	-3.45946700	-0.55471500	0.82397300
6	-2.12355900	-1.00914100	1.14973700
6	-2.05044800	-1.10762600	2.58322800
6	-3.29085400	-0.69854200	3.13523100
6	-4.15056600	-0.35217700	2.05992300
15	-0.61771200	-1.47068200	0.20015100
15	-0.00251900	2.01235500	-0.17319700
6	-1.07004800	-1.96644400	-1.52096800
6	-0.26006300	-3.10832100	0.96628900
6	1.03635500	-3.62225100	0.89738200
6	1.32864900	-4.90275700	1.39443100
6	0.29508600	-5.65816400	1.95461200
6	-1.02130300	-5.16993300	2.02262100
6	-1.28659200	-3.89293500	1.52134100
6	-1.23343900	-0.99088400	-2.51329400
6	-1.45080600	-1.35010900	-3.84893500
6	-1.47490300	-2.71152300	-4.18315800
6	-1.30294000	-3.70561500	-3.21435000
6	-1.10740800	-3.31890800	-1.88106000
26	-2.42207900	0.81624000	2.02558300
6	-1.10917000	2.18070900	1.25323100
6	-2.81248400	2.74852800	2.70826900
6	-0.70880600	1.76831800	2.58524000
6	-1.77862300	2.12811900	3.46570300
1	1.83316500	-3.00173800	0.49525000
1	-2.30714400	-3.51634900	1.55809900
1	-1.17651800	0.06283600	-2.25481200
1	-0.96148400	-4.09163700	-1.12768000
1	-3.52108600	-0.63025800	4.19650800
1	-5.17146200	0.01025600	2.14416300
1	-3.76740000	3.09861600	3.09553000
1	-1.82232500	1.92071500	4.53019900
1	-2.95653000	3.23936600	0.53238200
1	0.51291900	-6.65571200	2.34825400
1	-1.62525000	-3.00205700	-5.22673800
45	1.21566300	0.07281500	0.02508100
6	1.02116700	3.53958600	0.04639200
6	2.33429500	3.48388200	0.51878300
6	0.42161400	4.78788900	-0.17805500
6	3.06976100	4.65626300	0.74187700
1	2.78908100	2.52095300	0.73155200
6	1.12707000	5.97586300	0.04521100
1	-0.60765700	4.84278400	-0.53896300
6	2.45259900	5.88859400	0.49767600
1	3.01783200	6.81071100	0.66495000
6	-0.89921200	2.44220900	-1.71203200
6	-2.27351200	2.27783500	-1.89371200
6	-0.11214200	2.89860500	-2.78613000
6	-2.88393800	2.58646600	-3.12298800
1	-2.87989400	1.89198900	-1.08051100
6	-0.69096700	3.21693100	-4.01666100
1	0.96273300	3.03904500	-2.65387700
6	-2.07998300	3.06329100	-4.16131100
1	-2.54338300	3.30812100	-5.12115800
6	4.48619400	4.59284100	1.25801700
1	5.13709900	5.31607300	0.74276400
1	4.52871600	4.83162900	2.33421300
1	4.91889600	3.58934700	1.12975800
6	0.48875000	7.31977200	-0.19871400
1	0.47255900	7.92471700	0.72248600
1	1.04970900	7.89653400	-0.95213400
1	-0.54659600	7.21716300	-0.55378000
6	-4.36447900	2.38631000	-3.31648100
1	-4.95171600	3.00461000	-2.62081600
1	-4.67136900	2.63876100	-4.34104100
1	-4.65592100	1.34321500	-3.11743900
6	0.14481700	3.70964300	-5.16962700
1	0.07875400	3.02170200	-6.02864500
1	-0.20387300	4.69428200	-5.52018700
1	1.20415100	3.80453400	-4.89125300
6	-1.28957100	-5.16733600	-3.58061900
1	-1.58047900	-5.32779400	-4.62863600
1	-0.28245800	-5.59428100	-3.43870400
1	-1.97555000	-5.74608300	-2.94203200
6	-1.64962400	-0.29526500	-4.90572600
1	-2.66370500	0.13426800	-4.85158800
1	-0.95065500	0.54285300	-4.77393200
1	-1.51739800	-0.70765400	-5.91639300
6	-2.11660900	-6.00522900	2.63576200
1	-3.10520600	-5.54798900	2.48493300
1	-2.13861100	-7.01857300	2.20415100
1	-1.96390000	-6.12140500	3.72192400
6	2.73687800	-5.43360000	1.30161800
1	2.86488500	-6.35835200	1.88254800
1	3.00317700	-5.65185600	0.25393900
1	3.46272400	-4.69051400	1.66803800
1	-1.18221400	-1.42635300	3.14698900
6	0.63936900	1.16920700	2.93008300
1	0.96506800	0.63296200	2.00018700
6	-4.14375700	-0.54472000	-0.52844900
1	-3.50875600	0.01877500	-1.23096000
7	-4.17590000	-1.89637900	-1.06618900
6	-5.49137400	0.18641700	-0.45418200
6	-5.53623000	1.56233000	-0.18109500
6	-6.70625600	-0.48608200	-0.63435900
6	-6.74810000	2.24806400	-0.10005900
1	-4.60728100	2.09957100	-0.00242300
6	-7.92582400	0.19575300	-0.55749500
1	-6.70960500	-1.55531200	-0.83463300
6	-7.95334200	1.56547300	-0.29271400
1	-6.75298100	3.31915200	0.11724900
1	-8.85930700	-0.35251200	-0.70420800

1	-8.90521500	2.09768500	-0.23223900
6	-4.50217800	-2.02598600	-2.46498900
1	-5.59111900	-2.04193100	-2.68170300
1	-4.06354100	-1.19117700	-3.02929200
1	-4.06816700	-2.95702800	-2.86207800
6	-4.56548700	-3.02177800	-0.25055400
1	-3.99830600	-3.91830300	-0.56027500
1	-4.33804700	-2.83146600	0.80586500
1	-5.64345700	-3.28158100	-0.31264600
7	1.65032600	2.20185900	3.12968300
6	0.64290100	0.07402700	3.98987900
6	1.15286100	-1.18887200	3.65296400
6	0.19986200	0.29008900	5.30339100
6	1.15314300	-2.23298900	4.58017400
1	1.54857600	-1.36290300	2.65080500
6	0.20870000	-0.75074200	6.23621700
1	-0.15516700	1.27356600	5.61180000
6	0.67077500	-2.01986600	5.87402400
1	1.52492100	-3.21549100	4.28039700
1	-0.14938800	-0.56878600	7.25233900
1	0.66467100	-2.83402300	6.60233400
6	2.98345200	1.66842500	3.31128500
1	3.72015900	2.47686600	3.18505500
1	3.14900300	1.20877600	4.30725700
1	3.17963100	0.89253000	2.55394000
6	1.34554700	3.31707400	3.99890800
1	1.36526800	3.06485900	5.08094600
1	2.09146000	4.11075700	3.83119500
1	0.36089700	3.73705100	3.75570700
6	3.39887200	-1.99418200	-1.41631200
6	3.85763000	-3.28949000	-1.64061200
1	4.61147400	-3.71531900	-0.97354500
6	3.34556900	-4.04623100	-2.70191000
1	3.70276200	-5.06497600	-2.86934800
6	2.37217700	-3.49670000	-3.54128600
1	1.96693400	-4.08209000	-4.36941900
6	1.90483400	-2.19907100	-3.31913400
1	1.13725800	-1.77762400	-3.96721000
6	2.40370600	-1.44099100	-2.25247000
6	1.93740900	-0.06977600	-1.96657400
6	2.82740000	0.84030300	-1.28535300
6	3.92803200	0.30764100	-0.55576200
6	3.77182400	-1.17441700	-0.19850500
1	4.69094800	-1.57355800	0.26693100
1	1.23047800	0.34808600	-2.68635700
1	4.35958300	0.93099600	0.23202400
1	2.76391900	1.91160300	-1.46768600
8	2.71322700	-1.21303300	0.71027400
6	7.24295700	-0.74596300	-0.72575900
6	6.78001400	-1.44719100	-1.83355100
6	5.83348300	-0.60525700	-2.42778400
6	5.70466300	0.56868900	-1.65082200
8	6.66687600	0.44668400	-0.61347000
1	7.07622600	-2.45064700	-2.12357200
1	5.25533700	-0.81057100	-3.32629900
8	8.09449700	-1.12612000	0.19892600
6	5.54105200	1.96890300	-2.15649600
1	6.48245200	2.33732900	-2.59453200
1	4.76785700	1.98214800	-2.93600900
1	5.23525000	2.65645900	-1.35418500
1	8.24072800	-0.41417400	0.84335700

TS0’-*Si*-Cγ

E = -5300.00667254 G = -5298.744643

6	-2.82876800	2.65553300	0.77203900
6	-3.43018200	-0.83756800	0.56464800
6	-2.10717200	-1.08267600	1.10108300
6	-2.21460200	-1.00794500	2.53367900
6	-3.55395700	-0.69531700	2.87859200
6	-4.29665700	-0.58297500	1.67412400
15	-0.44574700	-1.44415700	0.39859100
15	-0.17137000	2.03892800	-0.34360700
6	-0.62683500	-2.17908900	-1.28734900
6	-0.00475000	-2.93360800	1.39245300
6	1.33999400	-3.29030100	1.51374800
6	1.71500200	-4.45941800	2.19485400
6	0.71584200	-5.26562800	2.74690200
6	-0.64530800	-4.93727300	2.62649500
6	-0.99256900	-3.76916800	1.94238100
6	-0.76159800	-1.34193200	-2.40271800
6	-0.77743400	-1.86576100	-3.70073100
6	-0.62644100	-3.24968900	-3.86808600
6	-0.47775300	-4.10679000	-2.77279700
6	-0.48594100	-3.55793500	-1.48261600
26	-2.71702600	0.77885300	1.69210800
6	-1.47646300	2.20487700	0.90563200
6	-3.40942400	2.70900700	2.06746100
6	-1.20930900	2.00209200	2.31681600
6	-2.41928400	2.31875700	3.01359300
1	2.10299300	-2.62873400	1.11235000
1	-2.04627700	-3.51898600	1.83372900
1	-0.83761700	-0.26653000	-2.26874200
1	-0.35897300	-4.22262500	-0.62911100
1	-3.92797500	-0.53530600	3.88777800
1	-5.35273300	-0.34159500	1.59014200
1	-4.44077400	2.97346900	2.29270000
1	-2.57819900	2.23177500	4.08374900
1	-3.32304000	2.92304900	-0.15590200
1	0.99760700	-6.17652200	3.28394000
1	-0.61858100	-3.66726200	-4.87893500
45	1.21719200	0.30278300	0.22468400
6	0.61740700	3.70466700	-0.17353500
6	1.85136100	3.87405600	0.45718500
6	-0.10202300	4.83267300	-0.59689200
6	2.39255300	5.15336800	0.64816500
1	2.39371600	3.00470700	0.81844400
6	0.40868800	6.12228200	-0.41344300
1	-1.07266600	4.71116900	-1.08254900
6	1.66091500	6.26097100	0.20525700
1	2.07512200	7.26423600	0.34548500
6	-0.89440500	2.19127200	-2.02020000
6	-2.19806800	1.81544900	-2.34852900
6	-0.03422800	2.65166600	-3.03453300
6	-2.67079900	1.91640000	-3.66933500
1	-2.85220000	1.42309700	-1.57620700
6	-0.47658900	2.76544100	-4.35463800
1	0.98504700	2.95683000	-2.78829400
6	-1.80130000	2.40219300	-4.64924100
1	-2.15738800	2.48556200	-5.67992300
6	3.72092600	5.33394100	1.34002600
1	4.32658400	6.11563200	0.85627700
1	3.58340000	5.63796600	2.39159200
1	4.30407800	4.40085300	1.34485200
6	-0.35728200	7.33766600	-0.87088300
1	-0.57516600	8.01102900	-0.02589800
1	0.22199200	7.92089600	-1.60525800
1	-1.31342400	7.06095400	-1.33760900
6	-4.07374200	1.49198300	-4.01771200
1	-4.82403900	2.11611300	-3.50860700
1	-4.25401400	1.55803800	-5.09977900
1	-4.26994200	0.45687700	-3.69825100
6	0.43627900	3.26534600	-5.44448900
1	0.55054100	2.51316200	-6.24220700
1	0.02888200	4.17384000	-5.91675800

1	1.43663600	3.50577600	-5.05689400
6	-0.27999800	-5.58968600	-2.95553600
1	-0.40519700	-5.89138000	-4.00534800
1	0.73109100	-5.89080600	-2.63360800
1	-0.99433100	-6.16627000	-2.34655000
6	-0.94631900	-0.96057500	-4.89297000
1	-2.00283700	-0.67461000	-5.02716600
1	-0.38444300	-0.02380800	-4.77054000
1	-0.61641400	-1.45038300	-5.82068000
6	-1.70316800	-5.82177000	3.23640500
1	-2.71231200	-5.52093700	2.91968000
1	-1.55626800	-6.87684900	2.95585900
1	-1.67071700	-5.77399000	4.33795100
6	3.17447800	-4.82243200	2.30456400
1	3.33880700	-5.64694000	3.01329800
1	3.57424900	-5.13336300	1.32483900
1	3.77132100	-3.95750800	2.63478600
1	-1.39975400	-1.15195800	3.23224100
6	0.14202600	1.62418400	2.89050400
1	0.65734600	1.05568700	2.07176500
6	-3.92976800	-1.05724300	-0.84915600
1	-3.27913800	-0.49283400	-1.53649300
7	-3.74149100	-2.45021700	-1.22516800
6	-5.35046600	-0.50108300	-1.01507400
6	-5.57736900	0.88302200	-0.97875700
6	-6.45745200	-1.34345300	-1.17652700
6	-6.86146500	1.41260400	-1.10691500
1	-4.73712700	1.55640900	-0.82353200
6	-7.74809800	-0.81958100	-1.30886000
1	-6.31903600	-2.42245000	-1.19723200
6	-7.95670000	0.55990200	-1.27649400
1	-7.00906300	2.49494600	-1.07200900
1	-8.59453500	-1.49847300	-1.43609400
1	-8.96420200	0.96898300	-1.37936600
6	-3.90319300	-2.76062600	-2.62395300
1	-4.95697700	-2.91327000	-2.93955200
1	-3.48661600	-1.94872200	-3.23697300
1	-3.34361500	-3.67738000	-2.86681000
6	-4.07789100	-3.52815600	-0.32616600
1	-3.36705400	-4.36323600	-0.46426900
1	-4.00596300	-3.19820800	0.71768500
1	-5.09806200	-3.94284800	-0.47020600
7	0.98448200	2.79506300	3.10390100
6	0.13035300	0.63879600	4.05338800
6	0.82780400	-0.57116600	3.92086400
6	-0.51669200	0.91037600	5.26827500
6	0.81711000	-1.52123800	4.94429000
1	1.37895600	-0.78079800	3.00257100
6	-0.52002100	-0.03449900	6.29808500
1	-1.02499300	1.86223900	5.42260000
6	0.13312000	-1.26016500	6.13419100
1	1.33824900	-2.47030500	4.79923800
1	-1.03819700	0.18799000	7.23395100
1	0.11708200	-2.00155000	6.93634300
6	2.33487600	2.46022300	3.50293500
1	2.98050900	3.34402800	3.38584600
1	2.41779300	2.11362400	4.55342800
1	2.72296800	1.65424500	2.85941400
6	0.43387200	3.93422600	3.80445500
1	0.33325900	3.78490200	4.90109900
1	1.09755100	4.80005400	3.64894400
1	-0.54997200	4.19900400	3.39585500
6	3.76943600	-1.63440500	-0.70072400
6	4.37840900	-2.88457500	-0.71959700
1	5.08605300	-3.15183200	0.06953500
6	4.06501500	-3.80798400	-1.72747900
1	4.53091800	-4.79623100	-1.72848800
6	3.14876000	-3.46142400	-2.72276500
1	2.89555700	-4.17514300	-3.50969900
6	2.54020400	-2.20213000	-2.71162600
1	1.81939500	-1.94054400	-3.48529400
6	2.83171400	-1.28402300	-1.69733100
6	2.20312700	0.05224100	-1.62792800
6	2.88134200	1.12241800	-0.93483400
6	3.97541300	0.78701400	-0.06295500
6	3.90438600	-0.65771400	0.44689200
1	4.78980200	-0.90088900	1.06321300
1	1.56321100	0.31431000	-2.47356600
1	4.23101400	1.51141100	0.71633700
1	2.76282000	2.15319800	-1.26927000
8	2.75051400	-0.70951300	1.22097800
6	6.61243400	0.26056400	-0.46591500
6	5.66013100	1.09083600	-1.09746000
6	5.56510600	0.50676700	-2.44199000
6	6.30569400	-0.61783700	-2.47297900
8	6.94675700	-0.77234800	-1.21508900
1	5.65634500	2.16426500	-0.91115300
1	4.94176600	0.87575600	-3.25195600
8	7.12902100	0.38064800	0.73069000
6	6.55874200	-1.68706300	-3.46126900
1	6.17266100	-2.65083900	-3.09606400
1	6.04421800	-1.44320800	-4.39977400
1	7.63594600	-1.79702700	-3.66506700
1	7.71267400	-0.37059800	0.93407300

TS0’-*Re*-Cγ

E = -5300.00627905 G = -5298.743659

6	1.95994900	-2.99797800	1.45791200
6	3.47200000	0.17529700	0.93920100
6	2.19668600	0.80942900	1.20270700
6	2.08009200	0.94014100	2.63068600
6	3.23103000	0.37834700	3.23911200
6	4.07934000	-0.09727400	2.20520400
15	0.80480900	1.43932000	0.17887800
15	-0.25444200	-1.94891400	-0.17752400
6	1.40036500	1.83188000	-1.52499000
6	0.62205500	3.12801800	0.89466700
6	-0.58204400	3.81061900	0.70803600
6	-0.74027400	5.12833100	1.16619500
6	0.33394000	5.74940600	1.80906600
6	1.56163000	5.09100100	1.99491900
6	1.69404500	3.78018400	1.52888200
6	1.46879900	0.82074700	-2.49225900
6	1.79478000	1.11491800	-3.82148100
6	2.02475700	2.45167900	-4.17636000
6	1.95141100	3.48200000	-3.23310800
6	1.64473100	3.15703600	-1.90421100
26	2.21312800	-1.02555000	2.11006800
6	0.76728900	-2.22252800	1.29581100
6	2.31935000	-2.97851800	2.83211400
6	0.36746700	-1.73713100	2.60337900
6	1.34306500	-2.21660700	3.53504300
1	-1.41709500	3.29763500	0.23946100
1	2.64842100	3.27269300	1.65669300
1	1.25007200	-0.20778000	-2.21950400
1	1.57702200	3.95897500	-1.17034600
1	3.40673400	0.29769400	4.30989600
1	5.03797000	-0.59163500	2.33633100
1	3.20396200	-3.44096000	3.26566200
1	1.36768700	-1.99868100	4.59807100
1	2.49240300	-3.52104500	0.67059000
1	0.22000800	6.77485800	2.17368900
1	2.26019800	2.69463400	-5.21639500
45	-1.20715700	0.13577100	-0.07089300

6	-1.47294800	-3.32671700	0.03937000
6	-2.78030600	-3.09089800	0.47053600
6	-1.03248200	-4.64724500	-0.13512300
6	-3.66435500	-4.15374800	0.70303200
1	-3.11395900	-2.07199100	0.64412200
6	-1.88969700	-5.72916500	0.09614600
1	-0.00917500	-4.84303400	-0.46241700
6	-3.20428400	-5.46110000	0.50754400
1	-3.88657200	-6.29872900	0.68288400
6	0.63419800	-2.53258700	-1.66976600
6	2.02382500	-2.54042800	-1.80221600
6	-0.16372400	-2.92151100	-2.76206800
6	2.63567900	-2.95676800	-2.99833600
1	2.64255500	-2.20441900	-0.97620700
6	0.41559700	-3.34323400	-3.96105800
1	-1.25171800	-2.92665000	-2.66849800
6	1.81716700	-3.36345300	-4.05506400
1	2.28121700	-3.69003300	-4.98993400
6	-5.07233700	-3.89580600	1.18023000
1	-5.80154200	-4.52926400	0.65078000
1	-5.17500000	-4.11954100	2.25548000
1	-5.35871300	-2.84400600	1.03601600
6	-1.42130900	-7.14978900	-0.09234800
1	-0.38033100	-7.18898400	-0.44355300
1	-1.48122500	-7.71573600	0.85169900
1	-2.04736300	-7.68238700	-0.82659300
6	4.13556000	-2.94314800	-3.13892700
1	4.61595400	-3.63006900	-2.42561500
1	4.44443300	-3.23371900	-4.15274500
1	4.54795600	-1.94465600	-2.92636000
6	-0.43238500	-3.76369600	-5.13361600
1	-0.25605500	-3.11017500	-6.00386500
1	-0.19073800	-4.79101500	-5.45012400
1	-1.50447200	-3.72619600	-4.89295000
6	2.15844400	4.92244800	-3.62469600
1	2.53583600	5.01520300	-4.65316100
1	1.21037500	5.48267200	-3.55994000
1	2.87237200	5.42189700	-2.95090100
6	1.88956100	0.01863800	-4.85025200
1	2.84458800	-0.52575100	-4.76270300
1	1.09331000	-0.72743800	-4.71788700
1	1.82980000	0.41927200	-5.87256900
6	2.70370800	5.78461600	2.69343700
1	3.63043700	5.19574400	2.63407900
1	2.89967300	6.77649400	2.25591600
1	2.47595000	5.94318200	3.76094300
6	-2.05087900	5.84074800	0.94667000
1	-2.09198700	6.79739100	1.48717400
1	-2.20780400	6.04841300	-0.12479700
1	-2.89698000	5.21837200	1.27876900
1	1.24132800	1.38158500	3.15419700
6	-0.90404800	-0.96035400	2.88186600
1	-1.13302700	-0.42414900	1.92366300
6	4.20385900	0.04538800	-0.38162300
1	3.53208300	-0.45069600	-1.10067800
7	4.43479500	1.36656900	-0.94518100
6	5.44220800	-0.84857700	-0.23073600
6	5.29880300	-2.22125800	0.02226700
6	6.73974000	-0.32815900	-0.31113200
6	6.40907200	-3.05067600	0.18088700
1	4.30085100	-2.64312400	0.12138700
6	7.85825500	-1.15439700	-0.15624500
1	6.88684700	0.73428600	-0.49359200
6	7.69914500	-2.51905200	0.08834600
1	6.26777400	-4.11598000	0.37996400
1	8.85984100	-0.72373000	-0.22604500
1	8.57213900	-3.16416000	0.20958100
6	4.85850200	1.40973200	-2.32281500
1	5.95006700	1.26816900	-2.47020100
1	4.34143300	0.62800800	-2.89724900
1	4.58320700	2.38007600	-2.76464200
6	4.90749300	2.46327400	-0.13458100
1	4.46211600	3.40930500	-0.49291300
1	4.60704700	2.32918000	0.91205100
1	6.00981100	2.59843300	-0.14533800
7	-2.04492400	-1.84735100	3.08329300
6	-0.79772800	0.16665100	3.90269600
6	-1.12650700	1.47122700	3.50484400
6	-0.42237600	-0.05316800	5.23653400
6	-1.00993300	2.54297800	4.39247300
1	-1.46773600	1.65715600	2.48503800
6	-0.31528800	1.01639700	6.12985200
1	-0.20936000	-1.06168000	5.59132100
6	-0.59295200	2.32023200	5.70717500
1	-1.23674500	3.55332700	4.04446100
1	-0.01069300	0.82967800	7.16242100
1	-0.49492700	3.15525300	6.40465300
6	-3.30137100	-1.13951400	3.20682200
1	-4.13416300	-1.84875400	3.08288000
1	-3.43186100	-0.62712200	4.18204500
1	-3.37390300	-0.37139300	2.42016700
6	-1.91160200	-2.96111300	3.99627800
1	-1.92357100	-2.67056800	5.06886800
1	-2.75222400	-3.65482600	3.83322800
1	-0.98616100	-3.51596400	3.79461200
6	-3.03929200	2.43650400	-1.65026800
6	-3.33439000	3.76406000	-1.94418900
1	-4.06659100	4.30116300	-1.33558700
6	-2.67977900	4.41540200	-2.99934100
1	-2.90627200	5.46079300	-3.22101900
6	-1.73204100	3.72572600	-3.75843400
1	-1.21459100	4.22758000	-4.57889100
6	-1.43797800	2.38929400	-3.47148700
1	-0.69795500	1.85512700	-4.06611800
6	-2.08232300	1.73492700	-2.41646400
6	-1.82692500	0.31776400	-2.08613700
6	-2.85244300	-0.44769300	-1.41604400
6	-3.90855500	0.24393600	-0.74314300
6	-3.56898400	1.71465500	-0.43193800
1	-4.44686800	2.24074700	-0.01939400
1	-1.16073700	-0.21210800	-2.77058500
1	-4.40101500	-0.26782600	0.08773400
1	-2.93029500	-1.52147600	-1.58424800
8	-2.55384000	1.62870400	0.51741000
6	-7.21173600	0.38970200	-0.34695100
6	-7.05727800	-0.97881400	-0.58276500
6	-6.10403700	-1.06379400	-1.59677100
6	-5.62998000	0.23714400	-1.90443400
8	-6.44050500	1.11773200	-1.13858900
1	-7.57755600	-1.77550400	-0.05911300
1	-5.71154400	-1.97568300	-2.04491300
8	-7.96528700	0.99240000	0.54109800
6	-5.26122100	0.74944900	-3.26248100
1	-4.43903100	0.14208400	-3.66609900
1	-6.11869200	0.68331600	-3.95067500
1	-4.92042700	1.78971700	-3.21366500
1	-7.88046200	1.95767600	0.46700500

4.7 The key parameter for the diastereodivergent lactonization of 6 & 7

Table S13. The role of components in the catalysis system A for the direct lactonization from **6 & 7** to **3a**[a]

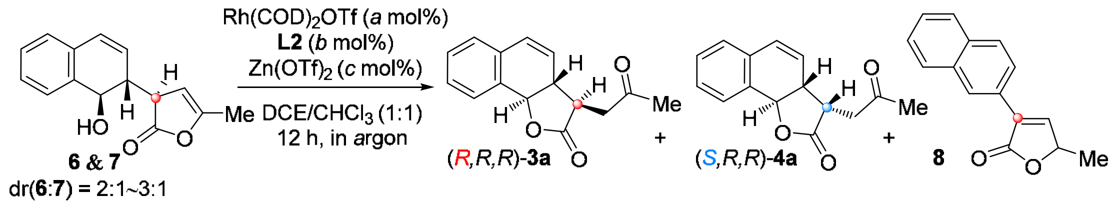

entry	Rh salt (*a* mol%)	**L2** (*b* mol%)	$Zn(OTf)_2$ (*c* mol%)	Temp.	(*R,R,R*)-**3a** yield (%), ee	(*S,R,R*)-**4a** yield (%), ee	**6 & 7** (recoveryed) or **8**
1	5	11	50	45 °C	12, 93%	63, 94%	**8**: 11%
2	5	11	50	90 °C	59, 93%	29, 94%	--
3	0	11	50	90 °C	61, 95%	30, 94%	--
4[b]	0	11	50	90 °C	57, 95%	33, 91%	--
5	5	11	0	90 °C	--	18, 94%	**6+7**: 72% (no loss dr)
6	0	11	0	90 °C	--	79, 94%	**6+7**: 18%, 1.5:1 dr
7	0	0	50	90 °C	--	--	**8**: 87%
8	5	0	0	90 °C	--	--	**8**: 90%

[a] General reaction conditions: without otherwise noted, **6 & 7** (0.1 mmol), $Rh(COD)_2OTf$ (*a* mol%), **L2** (*b* mol%) and $Zn(OTf)_2$ (*c* mol%) are dissolved in the mixture solvent of DCE (1 mL) and $CHCl_3$ (1 mL), and subsequently added additive under argon atmosphere. Yield refers to the ^{1}H-NMR yield, in which 1,4-benzodioxan was used as the internal standard. Dr values were determined by ^{1}H-NMR and show **3a**/**4a** and dr of **7** refers to the diastereoselective ratio of (*S,R,R*)-**6** and (*R,R,R*)-**7**. Ee values was determined by HPLC analysis on a chiral stationary phase and show **3a**/**4a**. [b] Racemic ligand **L2** was used.

Under condition A, when the temperature increased from 45 °C to 90 °C, the main product obtained from the intermediate changed from **4a** to **3a**, (entries 1-2) further indicating that **3a** is a thermodynamically controlled product. When there is a lack of $Rh(COD)_2OTf$ or a change in ligand configuration, the conversion of intermediates is not affected. (entries 3-4) When lacking **L2** or $Zn(OTf)_2$, there is no generation of product **3a,** only product **4a**, recovered intermediates, and isomerized/dehydrated product **8** can be detected. (entries 5-8) These experiments indicate that **3a** is not easily obtainable, and the isomerization of intermediates,

followed by the formation of product **4a**, is relatively easy to occur.

Table S14. The role of components in the catalyst system B on the Cα-epimerization/lactonization of **6 & 7** [a]

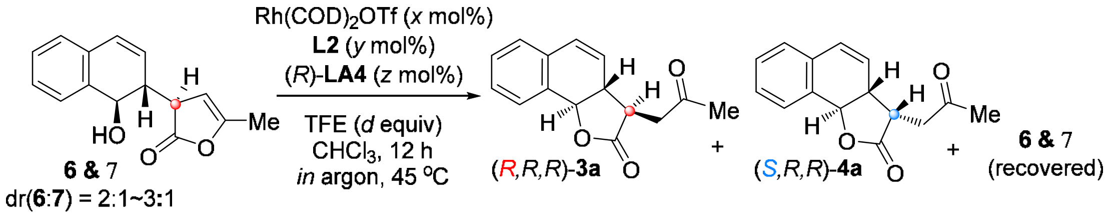

entry	Rh salt (x mol%)	**L2** (y mol%)	**LA4** (z mol%)	TFE (d equiv)	(R,R,R)-**3a** yield (%), ee	(S,R,R)-**4a** yield (%), ee	**6 & 7** (recovered)
1	5	11	20	0	-	93, 92%	--
2	5	11	20	3.5	-	93, 93%	-
3	0	11	20	0	-	95, 94%	-
4	0	0	20	0	-	93, 95%	-
5	0	11	0	0	-	23, 94%	67%, 2.4:1 dr
6	5	0	0	0	-	-	94%, 3:1 dr
7[b]	0	0	20	0	8, 94%	84, 95%	-

[a] General reaction conditions: without otherwise noted, **6 & 7** (0.1 mmol), Rh(COD)$_2$OTf (x mol%), **L2** (y mol%), (R)-**LA4** (based on z mol% Sn(OTf)$_2$), and TFE (d equiv) are dissolved in $CHCl_3$, and subsequently added additive under argon atmosphere at 45 °C. Yield refers to the ^{1}H-NMR yield, in which 1,4-benzodioxan was used as the internal standard. Dr values were determined by ^{1}H NMR spectroscopy and show **3a**/**4a** and dr of **6 & 7** refers to the diastereoselective ratio of (S,R,R)-**6** and (R,R,R)-**7**. Ee values was determined by HPLC analysis on a chiral stationary phase and show **3a**/**4a**. [b] At 90 °C.

Under condition B, we investigated the effects of each component on the intramolecular lactonization of the intermediate. It was found that the cocatalyst **LA4** plays a crucial role in the lactonization of the intermediate. In the presence of **LA4**, the intermediate could still afford the product **4a** in high yield even when one or more other components were absent from the system. (entries 1-4) Only a trace amount of the lactonized product was obtained with a large excess of the intermediate remaining when only the **L2** was present in the system. (entry 5) No conversion of the intermediate into the product was observed when only the Rh(COD)$_2$OTf was present. (entry 6) When **LA4** was the sole component and the temperature was elevated to 90 °C, a small amount of product **3a** was detected along with **4a**. (entry 7) These

experimental results demonstrate that **LA4** is the only effective component responsible for the transformation of the intermediate to the product, whereas the $Rh(COD)_2OTf$, **L2** and **TFE** do not contribute to this process.

Table S15. The roles of components of combined Lewis acid **LA4** in the Cα-epimerization/lactonization of **6 &·7**

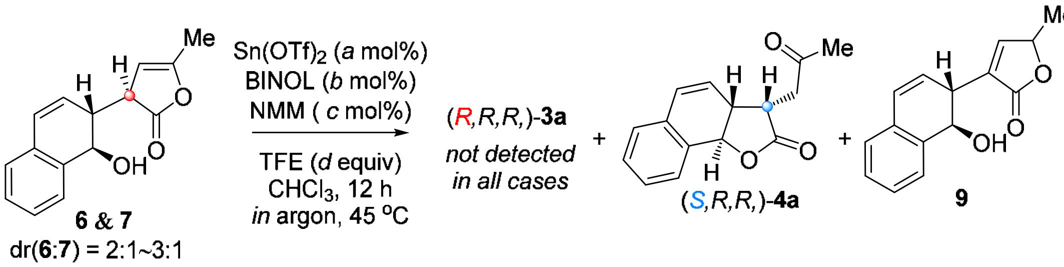

entry	$Sn(OTf)_2$ (*a* mol%)	BINOL (*b* mol%)	NMM (*c* mol%)	TFE (*d* equiv)	(*S,R,R*)-**4a** yield (%), ee	**6 & 7** (recovered) or **9**
1	20	24	48	0	93, 95%	--
2	0	0	48	0	44, 95%	**9**: 41%, 1:1 dr
3	0	24	0	0	--	**6+7**: 95%, 3:1 dr
4	20	0	0	0	--	complex
5	0	0	0	3.5	--	**6+7**: 94%, 3:1 dr
6	20	24	0	0	--	complex
7	20	0	48	0	45, 95%	**6+7**: 45%, 1:1 dr
8	0	24	48	0	41, 92%	**6+7**: 18%, 1:1 dr<br>**9**: 33%, 1:1 dr
9	0	24	48	3.5	70, 93%	**6+7**: 21%, 1:1 dr
10	0	0	48	3.5	41, 93%	**6+7:** 54%,1:1.2 dr

[a] General reaction conditions: without otherwise noted, **6 & 7** (0.1 mmol), $Sn(OTf)_2$ (*a* mol%), (*R*)-BINOL (*b* mol%), NMM (*c* mol%) and TFE (*d* equiv) are dissolved in $CHCl_3$ and subsequently added additive under argon atmosphere at 45 °C. Yield refers to the ^{1}H-NMR yield, in which 1,4-benzodioxan was used as the internal standard. Dr values were determined by ^{1}H NMR spectroscopy and show **3a**/**4a** and dr of **6 & 7** refers to the diastereoselective ratio of (*S,R,R*)-**6** and (*R,R,R*)-**7**; drs of **3a**/**4a** in all cases are >20:1. Ee values was determined by HPLC analysis on a chiral stationary phase and show **3a**/**4a**.

Finally, to elucidate the specific roles of the combined cocatalyst **LA4** for the Cα-epimerization/lactonization of **6 & 7** to (*S,R,R*)-**4a**, several control experiments were conducted using **6 & 7** with 2:1~3:1 dr as the substrate. As summarized in Table S15,

all the three components in **LA4** ($Sn(OTf)_2$, BINOL, and NMM) were essential for the efficient formation of (*S,R,R*)-**4a** (entry 1). Systematic omission experiments revealed distinct functions for each component. The absence of NMM completely suppressed the formation of either cyclized product (*S,R,R*)-**4a** or (*R,R,R*)-**3a** (entries 3-6) and with no change in the diastereomeric ratio (dr) of recovered **6 & 7**. In contrast, all reactions conducted in the presence of NMM led to the recovered **7** with a decreased dr of approximately 1:1, along with varying yields of cyclized products **4a**. These observations suggest that NMM involves the deprotonation step to promote keto-enol tautomerization or epimerization that is the rate-determining step. Without BINOL, the yield of (*S,R,R*)-**4a** dropped to 45%, and 45% of **6 & 7** was recovered (entry 7). Without $Sn(OTf)_2$, (*S,R,R*)-**4a** was obtained in 41% yield along with the formation of olefin isomerization byproduct **9** (33%) (entry 8). These control results revealed that **LA4** serves as a multifunctional catalyst that promote both epimerization via a proton-shuttle catalysis and the following lactonization. Moreover, TFE can effectively prevent the olefin isomerization of **6 & 7** to **9** (entries 8 vs 9; 2 vs 10). No (*R,R,R*)-**3a** formed in these control experiments further confirms that the direct lactonization of (*S,R,R*)-**6** to (*R,R,R*)-**3a** is an unfavorable process, likely involving a high-energy transition state. In contrast, the transformation pathway—epimerization of (*S,R,R*)-**6** to (*R,R,R*)-**7**, followed by lactonization to afford (*S,R,R*)-**4a**—is kinetically driven.

5. Gram-scale reaction

5.1 Gram-scale reaction of (*R,R,R*)-3a

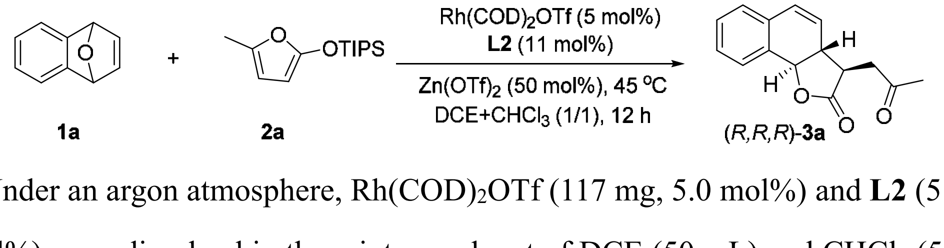

Under an argon atmosphere, $Rh(COD)_2OTf$ (117 mg, 5.0 mol%) and **L2** (515 mg, 11 mol%) were dissolved in the mixture solvent of DCE (50 mL) and $CHCl_3$ (50 mL) and the resulting solution was stirred for 30 min at RT. Subsequently, $Zn(OTf)_2$ (910 mg, 50 mol%), **1a** (720 mg, 5 mmol) and **2a** (6.4 g, 25 mmol) were added. The

reaction mixture was stirred at 45 °C for 12 h. After reaction completion, the solvent was evaporated under reduced pressure. The residue was purified by flash column chromatography on silica gel (petroleum ether/ ethyl acetate = 10:1) to afford the (*R,R,R*)-**3a** (white solid, 0.82 g, 68% yield, 12:1 dr, 91% ee).

5.2 Gram-scale reaction of (*S,R,R*)-4a

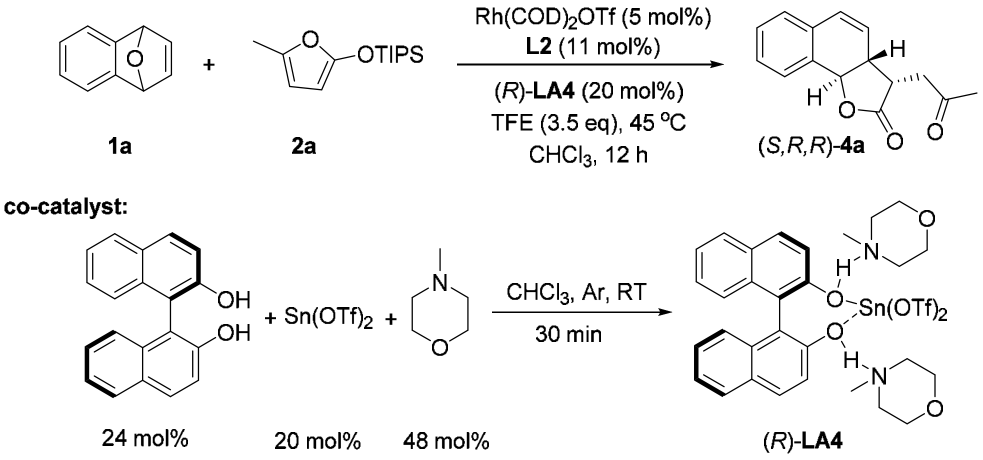

In a round-bottom flask, $Rh(COD)_2OTf$ (117 mg, 5.0 mol%) and **L2** (515 mg, 11 mol%) were dissolved in $CHCl_3$ (50 mL) and the resulting solution was stirred for 30 min at RT. In another round-bottom flask, (*R)*-BINOL (342 mg, 24 mol%), $Sn(OTf)_2$ (417 mg, 20 mol%), NMM (245 mg, 48 mol%) were dissolved in $CHCl_3$ (50.0 mL) and stirred for 30 min at RT, to give the solution of cocatalyst (*R*)-**LA4**. Then, the solution of cocatalyst (*R*)-**LA4** in $CHCl_3$ were added to the first round-bottom flask. Subsequently, TFE (1.75 g, 3.5 eq), **1a** (720 mg, 5 mmol) and **2a** (6.4 g, 25 mmol) were added. The reaction mixture was stirred at 45 °C for 12 h. After reaction completion, the solvent was evaporated under reduced pressure. The residue was purified by flash column chromatography on silica gel (petroleum ether/ ethyl acetate = 10:1) to afford the (*S,R,R*)-**4a** (white solid, 0.88 g, 73% yield, 94% ee).

6. Synthetic transformations

We have successfully transformed the highly functionalized tricyclic γ-lactones **3a** into privileged chiral scaffolds including terpenoid tricyclic γ-lactones, monocyclic γ-lactones, dihydropyridazin-3(2H)-ones, and fused dioxabicyclo[3.2.1]octanes – structural motifs which are widely represented in natural products and pharmaceutical agents.

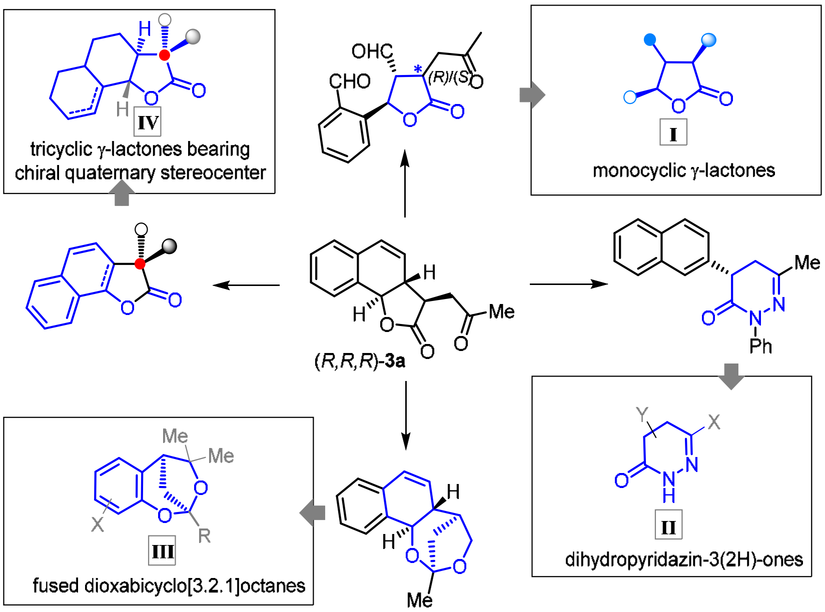

Figure S11. Skeletal transformation in the late stage.

6.1 Late-stage functionalization of alkene moiety

In addition to quaternary carbonization at the Cα-position, we can also modify the cyclic skeleton of the product, transforming it from the original 6,6,5-tricyclic skeleton to monocyclic γ-aryl-γ-lactone, dihydropyridazin-3(*2H*)-ones, fused dioxabicyclo[3.2.1]octanes. These scaffolds are not only found in numerous natural products but can also serve as key intermediates in the synthesis of certain natural products.

6.1.1 Stereodivergent synthesis of two stereoisomers of monocyclic γ-lactones

The skeleton containing monocyclic γ-aryl-γ-lactone exists in the natural products nicotlactone B,[11] solanamide L,[12] isohydroxymatairesinol, *epi*-isohydroxymatairesinol,[13] pycnanolide B[14], gymnothelignan $X_{5\text{-}8}$,[15] pharbilignanol D,[16] eupomatilone 4/7,[17] aminolactone,[18] paraconic acids,[19] and it also a butanolides-

intermediate of (±)-porosin.[20]

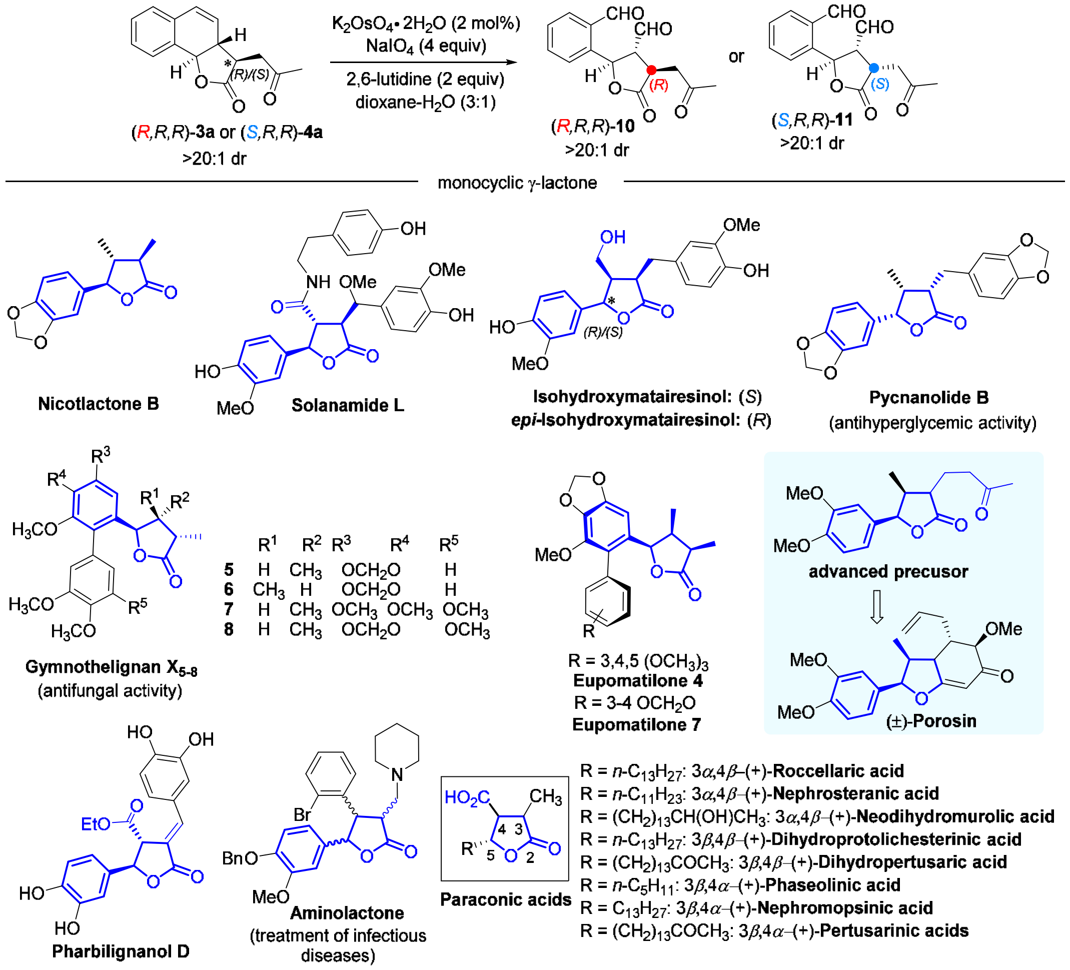

To a solution of compound (*R,R,R*)-**3a** (24.2 mg, 0.1 mmol, 1.0 equiv) or (*S,R,R*)-**4a** (24.2 mg, 0.1 mmol, 1.0 equiv) in dioxane-water (3:1, 1.0 mL) were added 2,6-lutidine (23 µL, 0.2 mmol, 2.0 equiv), $K_2OsO_4 \cdot 2H_2O$ (0.6 mg, 0.002 mmol, 2 mol%), and $NaIO_4$ (86.4 mg, 0.4 mmol, 4.0 equiv). The reaction was stirred at 25°C and monitored by TLC. After the reaction was complete, water (10 mL) and CH_2Cl_2 (20 mL) were added. The organic layer was separated, and the water layer was extracted by CH_2Cl_2 (10 mL) three times. The combined organic layer was washed with brine and dried over Na_2SO_4. The solvent was removed, and the product was purified with silica gel column chromatography to afford aldehyde **10** or **11** as a colorless oil.[21]

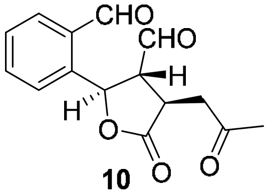

10

17.5 mg, 64% yield, >20:1 dr.

^{1}H NMR (600 MHz, CDCl$_3$) δ 10.00 (s, 1H), 9.86 (d, *J* = 3.2 Hz, 1H), 7.91 (d, *J* = 7.9 Hz, 1H), 7.85 (dd, *J* = 7.6, 1.4 Hz, 1H), 7.74 (td, *J* = 7.6, 1.5 Hz, 1H), 7.62 (t, *J* = 7.5 Hz, 1H), 6.45 (d, *J* = 8.3 Hz, 1H), 3.34 (ddd, *J* = 10.2, 7.1, 3.3 Hz, 1H), 3.12 – 2.98 (m, 3H), 2.16 (s, 3H);

^{13}C NMR (151 MHz, CDCl$_3$) δ 205.1, 197.0, 193.8, 175.8, 139.7, 136.0, 134.9, 132.4, 129.3, 127.3, 75.6, 61.3, 42.1, 37.9, 29.6;

HRMS (ESI) m/z: [M+Na]$^+$ calculated for $C_{15}H_{14}O_5Na^+$: 297.0733, found: 297.0736;

$[\alpha]_D^{25}$ = +4.34 (c = 0.08, $CHCl_3$).

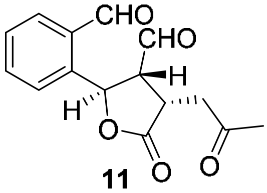

11

16 mg, 58% yield, >20:1 dr.

^{1}H NMR (600 MHz, CDCl$_3$) δ 10.07 (d, *J* = 0.8 Hz, 1H), 10.03 (dd, *J* = 1.8, 0.7 Hz, 1H), 7.97 (dd, *J* = 7.4, 1.5 Hz, 1H), 7.70 (td, *J* = 7.6, 1.5 Hz, 1H), 7.65 (tdd, *J* = 7.4, 1.3, 0.6 Hz, 1H), 7.58 (dd, *J* = 7.7, 1.1 Hz, 1H), 6.42 (s, 1H), 3.34 (dt, *J* = 9.5, 1.8 Hz, 1H), 3.15 – 3.07 (m, 2H), 2.97 – 2.90 (m, 1H), 2.17 (s, 3H).

^{13}C NMR (151 MHz, CDCl$_3$) δ 206.1, 200.5, 193.8, 176.8, 140.4, 137.1, 134.6, 131.7, 129.0, 124.8, 54.8, 39.7, 35.3, 29.7.

HRMS (ESI) m/z: [M+Na]$^+$ calculated for $C_{15}H_{14}O_5Na^+$: 297.0733, found: 297.0735;

$[\alpha]_D^{25}$ = -3.50 (c = 0.08, $CHCl_3$).

6.1.2 Functionalization of (*R,R,R*)-3a olefins.

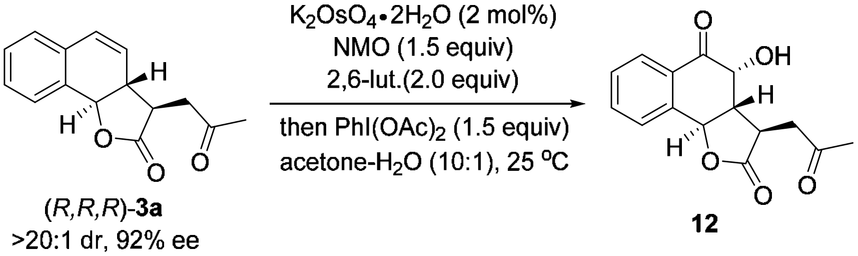

(*R,R,R*)-**3a**
>20:1 dr, 92% ee

12

To a solution of (*R,R,R*)-**3a** (24.2 mg, 0.1 mmol, 1.0 equiv) in 10:1 acetone:water (0.1 M) was added 2,6-lutidine (23 μL, 0.2 mmol, 2.0 equiv), 4-methylmorpholine *N*-oxide (17.6 mg, 0.15 mmol, 1.5 equiv), and $K_2OsO_4 \cdot 2H_2O$ (0.6 mg, 0.002 mmol, 2 mol%). When the starting material had been consumed as monitored by TLC, $PhI(OAc)_2$ (48.3 mg, 0.15 mmol, 1.5 equiv) was added. After stirring for 2 h, the reaction was quenched with saturated aqueous sodium thiosulfate (5 mL). The mixture was extracted with ethyl acetate (3 × 10 mL), washed with saturated aqueous copper sulfate (2 × 20 mL), dried over sodium sulfate, and concentrated in vacuo. The crude residue was purified by flash column chromatography to give the product **12** (17.7 mg, 65% yield, >20:1 dr, 88% ee).[22]

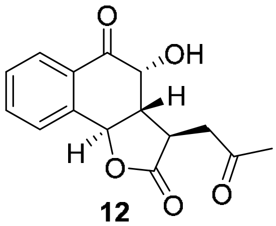

12

^{1}H NMR (600 MHz, Acetone-*d*$_6$) δ 7.99 (dd, *J* = 7.8, 1.3 Hz, 1H), 7.73 (td, *J* = 7.6, 1.4 Hz, 1H), 7.57 – 7.51 (m, 2H), 5.67 (d, *J* = 11.1 Hz, 1H), 5.44 – 5.38 (m, 1H), 4.44 (dd, *J* = 4.6, 2.7 Hz, 1H), 3.45 (ddd, *J* = 12.5, 6.8, 4.6 Hz, 1H), 3.13 (dd, *J* = 18.5, 4.6 Hz, 1H), 2.91 (dd, *J* = 18.5, 6.8 Hz, 1H), 2.77 (ddd, *J* = 13.1, 11.1, 2.7 Hz, 1H), 2.20 (s, 3H);

^{13}C NMR (151 MHz, Acetone-*d*$_6$) δ 206.1, 194.7, 177.0, 142.1, 134.9, 130.2, 129.3, 129.1, 123.9, 73.7, 70.2, 53.0, 41.4, 38.2, 30.2, 30.1, 29.9, 29.8, 29.7, 3.5, 29.4;

HPLC analysis: Daicel CHIRALCEL® OD-H, *n*-hexane/*i*-PrOH = 80/20, flow rate = 1.0 mL/min, λ = 254 nm, retention time: t $_{minor}$ = 18.0 min, t $_{major}$ = 20.1 min;

HRMS (ESI) m/z: $[M+Na]^+$ calculated for $C_{15}H_{14}O_5Na^+$: 297.0733, found: 297.0730;

[α]$_D$^{25} = -9.63 (c = 0.08, $CHCl_3$).

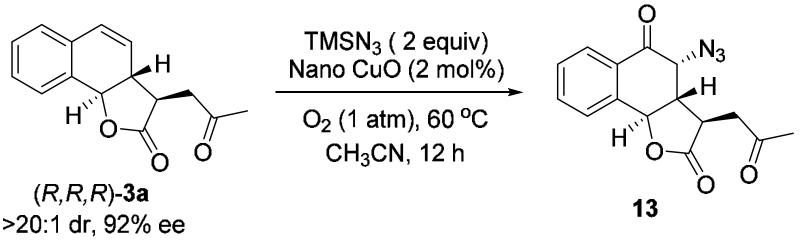

The solution of (*R,R,R*)-**3a** (24.2 mg, 0.1 mmol, 1.0 equiv), $TMSN_3$ (23 mg, 0.2 mmol, 2.0 equiv), and nano CuO (0.2 mg, 2 mol%) in MeCN (1 mL) was stirred at 60 °C for 12 h under the atmosphere of O_2 (1 atm). After cooling down to room temperature, the mixture was concentrated in vacuum, the residue was purified through column chromatography on silica gel to afford pure product **13** (10.8 mg, 36% yield, 5:1 dr, 78% ee).[23]

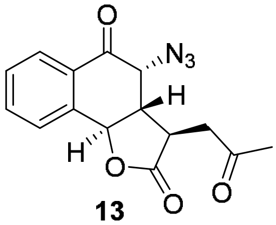

13

^{1}H NMR (600 MHz, $CDCl_3$) δ 8.16 – 8.02 (m, 1H), 7.68 (t, *J* = 7.4 Hz, 1H), 7.58 (d, *J* = 7.8 Hz, 1H), 7.50 (t, *J* = 8.1 Hz, 1H), 5.46 (d, *J* = 10.9 Hz, 1H), 4.41 (s, 1H), 3.35 (ddd, *J* = 12.3, 8.7, 3.8 Hz, 1H), 3.24 (dd, *J* = 18.7, 3.7 Hz, 1H), 2.72 (dt, *J* = 18.7, 5.3 Hz, 1H), 2.66 – 2.57 (m, 1H), 2.25 (s, 3H);

^{13}C NMR (151 MHz, $CDCl_3$) δ 205.2, 190.4, 175.7, 140.2, 134.9, 129.3, 128.9, 128.4, 123.4, 73.2, 62.1, 50.9, 42.2, 4.25, 30.0;

HPLC analysis: Daicel CHIRALCEL® OD-H, *n*-hexane/*i*-PrOH = 85/15, flow rate = 1.0 mL/min, λ = 254 nm, retention time: t_{minor} = 29.6 min, t_{major} = 32.2 min;

HRMS (ESI) m/z: $[M+Na]^+$ calculated for $C_{15}H_{13}O_4N_3Na^+$: 322.0798, found: 322.0796;

[α]$_D$^{25} = +162.86 (c = 0.07, $CHCl_3$).

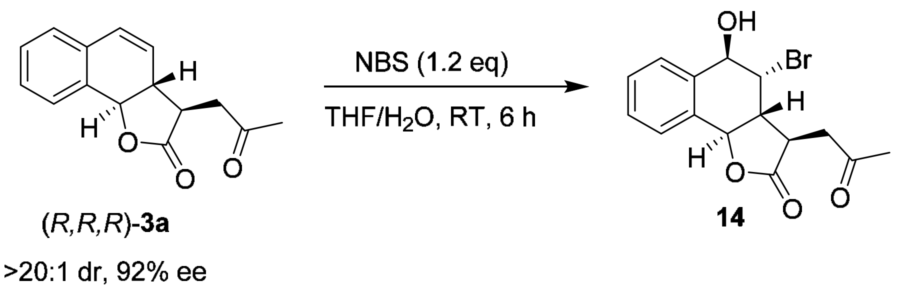

To a stirred solution of (*R,R,R*)-**3a** (24.2 mg, 0.1 mmol, 1.0 eq.) in THF/H_2O (1.0 mL, v/v = 9:1) was added *N*-bromosuccinimide (NBS, 21.4 mg, 0.12 mmol, 1.2 eq.) at RT. After stirring for 6 h at RT, the reaction was extracted with ethyl acetate (2 × 20 mL) and then washed with H_2O and brine, dried over Na_2SO_4 and concentrated in vacuum. The crude product was purified by flash column chromatography on silica gel to yield the pure product **14** as white liquid (20.3 mg, 60% yield, >20:1 dr, 93% ee).[24]

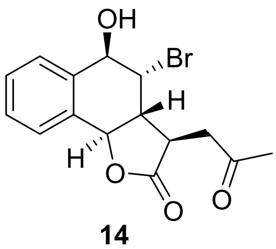

14

^{1}H NMR (500 MHz, DMSO-*d*$_6$) δ 7.51-7.46 (m, 1H), 7.43-7.37 (m, 2H), 7.37-7.33 (m, 1H), 6.34 (d, *J* = 6.1 Hz, 1H), 5.25 (d, *J* = 10.9 Hz, 1H), 4.94 (dd, *J* = 6.1, 2.0 Hz, 1H), 4.63 (t, *J* = 2.4 Hz, 1H), 3.13-2.99 (m, 2H), 2.88 (dd, *J* = 18.1, 5.7 Hz, 1H), 2.70 (td, *J* = 11.3, 2.8 Hz, 1H), 2.16 (s, 3H).

^{13}C NMR (126 MHz, DMSO-*d*$_6$) ^{13}C NMR (126 MHz, DMSO) δ 206.0, 176.4, 135.2, 133.45, 130.4, 128.1, 127.9, 122.9, 76.0, 73.3, 54.4, 45.2, 40.0, 39.9, 29.9.

HRMS (ESI) m/z: $[M+Na]^+$ calculated for $C_{15}H_{15}BrO_4Na^+$: 361.0046, found: 361.0049;

HPLC analysis: Daicel CHIRALPAK® AS-H, *n*-hexane/*i*-PrOH = 70/30, flow rate = 1.0 mL/min, λ = 210 nm, retention time: t_{minor} = 8.7 min, t_{major} = 11.5 min;

$[\alpha]_D^{25}$ = +7.63 (c = 0.08, $CHCl_3$).

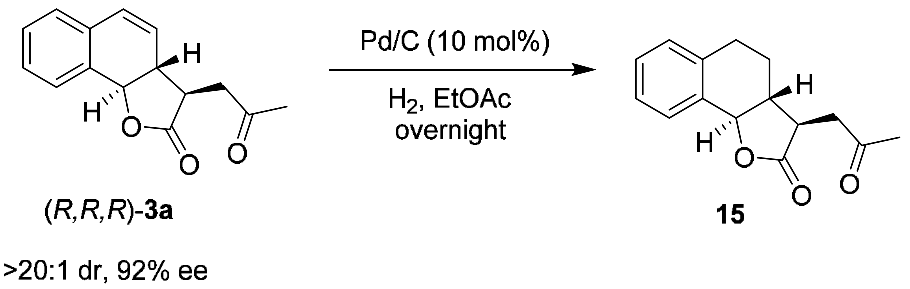

To a stirring solution of (*R,R,R*)-**3a** (24.2 mg, 0.1 mmol, 1.0 equiv) in ethyl acetate (1.0 mL) was slowly added palladium on-activated-charcoal (10%, 20.0 mg) at room temperature. The resulting mixture was stirred at room temperature in an atmosphere of hydrogen gas for overnight. The mixture was filtered and concentrated under reduced pressure. The residue was purified by column chromatography on silica gel to give **15** (24 mg, 98% yield).

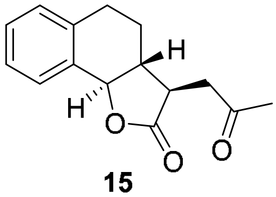

15

^{1}H NMR (500 MHz, CDCl$_3$) δ 7.41-7.37 (m, 1H), 7.35-7.18 (m, 2H), 7.16-7.10 (m, 1H), 4.96 (d, J = 10.6 Hz, 1H), 3.11 (dd, J = 18.3, 4.3 Hz, 1H), 3.06-3.01 (m, 1H), 3.00-2.89 (m, 2H), 2.61 (dd, J = 18.3, 7.5 Hz, 1H), 2.24 (s, 3H), 2.14-2.07 (m, 1H), 2.00-1.94 (m, 1H), 1.91-1.81 (m, 1H);

^{13}C NMR (126 MHz, CDCl$_3$) δ 205.4, 178.1, 134.8, 134.5, 128.8, 127.7, 125.9, 123.2, 80.8, 47.3, 42.2, 42.2, 30.1, 27.6, 23.3;

HRMS (ESI) m/z: $[M+H]^+$ calculated for $C_{15}H_{17}O_3^+$: 245.1172, found: 245.1175.

$[\alpha]_D^{25}$ = -89.50 (c = 0.10, CHCl$_3$).

6.2 Construction of fused dihydropyridazin-3(2H)-ones.

The skeleton containing dihydropyridazin-3(2H)-ones exists in the natural products pimobendan[25], levosimendan[26], meribendan[27], bemoradan[28], it can also be transformed into herbicides **41**.[29]

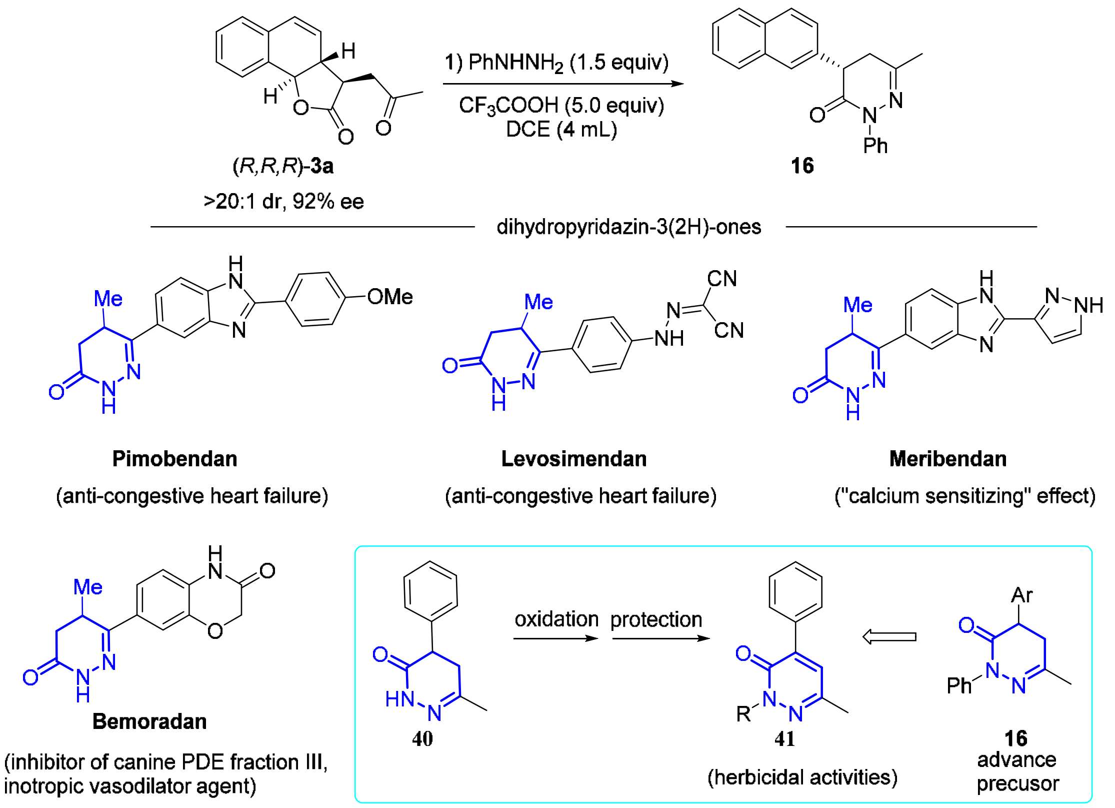

A solution of (*R,R,R*)-**3a** (24.2 mg, 0.1 mmol, 1.0 equiv), phenylhydrazine (15 μL, 0.15 mmol), and trifluoroacetic acid (38 μL, 0.5 mmol) in DCE (5.0 mL) was allowed to react under an argon atmosphere at 40 °C for 16 h, and then cooled to RT. The reaction was extracted with ethyl acetate (2 × 20 mL) and then washed with brine, dried over Na_2SO_4 and concentrated in vacuum. The crude product was purified by flash column chromatography on silica gel to yield the pure product **16** as white liquid (29.3 mg, 93% yield, 86% ee).[30]

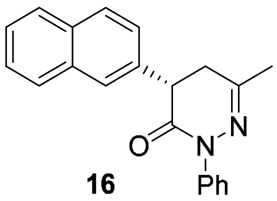

16

^{1}H NMR (500 MHz, CDCl$_3$) δ 7.85-7.76 (m, 3H), 7.68 (d, *J* = 1.9 Hz, 1H), 7.58-7.52 (m, 2H), 7.50-7.43 (m, 2H), 7.43-7.36 (m, 3H), 7.28-7.22 (m, 1H), 4.04 (t, *J* = 7.3 Hz, 1H), 2.97 (qdd, *J* = 17.0, 7.3, 1.7 Hz, 2H), 2.13 (s, 3H);

^{13}C NMR (126 MHz, CDCl$_3$) δ 165.8, 153.9, 141.1, 134.8, 133.3, 132.7, 128.7, 128.6, 128.6, 127.8, 127.6, 126.7, 126.6, 126.3, 126.2, 126.0, 125.6, 125.0, 43.4, 33.9, 23.5;

HRMS (ESI) m/z: $C_{21}H_{19}ON_2^+$ calculated for $[M+H]^+$: 319.1492, found: 319.1495;

HPLC analysis: Daicel CHIRALPAK® ID, *n*-hexane/*i*-PrOH = 80/20, flow rate = 1.0 mL/min, λ = 254 nm, retention time: t_{major} = 16.7 min, t_{minor} = 40.3 min;

$[\alpha]_D^{25}$ = -83.00 (c = 0.07, $CHCl_3$).

6.3 Construction of fused dioxabicyclo[3.2.1]octanes.

The skeleton containing fused dioxabicyclo[3.2.1]octanes exists in the natural products cystophloroketals A-E,[31] bullataketals A, B,[32] falandiosides A.[33]

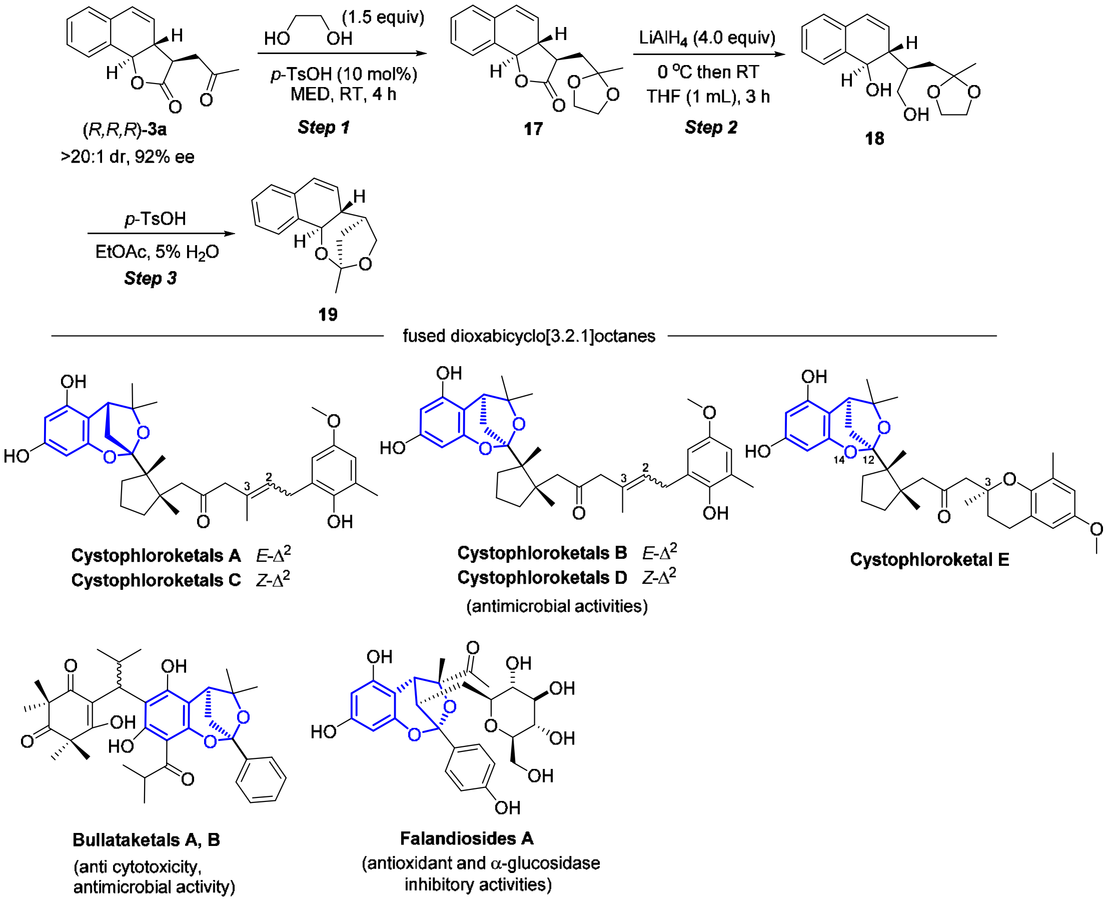

Step 1: (*R,R,R*)-**3a** (100 mg, 0.41 mmol, 1.0 equiv) were dissolved in 4 mL of 2-ethyl-2-methyl-1,3-dioxolane (MED) in a 25 mL flask, and catalytic amounts of *p*-toluenesulfonic acid (7 mg, 0.041 mmol, 10 mol%) and ethylene glycol (38.4 mg, 0.62 mmol, 1.5 eq) were added. The reaction was stirred overnight at room temperature. Then, the mixture was neutralized with 0.1 mL of triethylamine to

eliminate the excess acid; then, 20 mL of aqueous Na_2CO_3 was added and extracted three times with 20 mL of ethyl acetate. The organic layers were combined and dried with anhydrous Na_2SO_4, and the solvent was evaporated under reduced pressure. The products were purified by column chromatography to give the ketal derivatives **17** (88 mg, 75% yield, 90% ee).[34]

Step 2: To a solution of **17** (28.6 mg, 0.1 mmol, 1.0 equiv) in THF (1 mL) at 0 °C was added $LiAlH_4$ (15 mg, 0.4 mmol). The mixture was stirred at room temperature for 3 h, then H_2O (1 mL) was added carefully. The aqueous layer was extracted with CH_2Cl_2 (3 × 20 mL). The combined organics were then dried ($NaSO_4$) and concentrated in vacuo. The residue was purified by flash column chromatography to give the product **18** (27.6 mg, 95% yield, >20:1 dr, 89% ee).[35]

Step 3: The **18** (29 mg, 0.1 mmol, 1.0 equiv) was dissolved in 1.0 mL of ethyl acetate, and a catalytic amount of *p*-TsOH (32.6 mg, 0.1 mmol, 10 mol%) was added. Then, 50 μL of distilled water was added. The mixture was stirred overnight at room temperature. The crude product was extracted three times with ethyl acetate, dried with anhydrous Na_2SO_4, and evaporated under reduced pressure. The products were purified by flash column chromatography on silica gel to yield the pure product **19** (12.6 mg, 55% yield, 11:1 dr, 89% ee).[34]

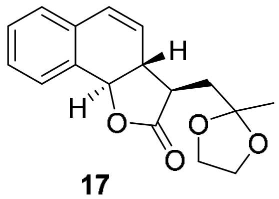

17

^{1}H NMR (500 MHz, $CDCl_3$) δ 7.45 (dd, *J* = 5.2, 3.5 Hz, 1H), 7.34 – 7.27 (m, 2H), 7.23 – 7.16 (m, 1H), 6.58 (dd, *J* = 9.5, 2.9 Hz, 1H), 6.43 (dd, *J* = 9.5, 2.2 Hz, 1H), 4.95 (d, *J* = 14.2 Hz, 1H), 4.07 – 3.93 (m, 4H), 2.79 (ddd, *J* = 12.7, 8.3, 3.0 Hz, 1H), 2.70 (ddt, *J* = 15.0, 12.5, 2.5 Hz, 1H), 2.57 (dd, *J* = 14.9, 2.9 Hz, 1H), 1.84 (dd, *J* = 15.0, 8.3 Hz, 1H), 1.41 (s, 3H);

^{13}C NMR (126 MHz, $CDCl_3$) δ 178.5, 134.3, 132.4, 129.2, 127.9, 127.8, 127.2, 126.8,

121.6, 108.8, 80.4, 64.6, 64.3, 47.8, 41.6, 38.4, 23.9;

HPLC analysis: Daicel CHIRALPAK® ID, *n*-hexane/*i*-PrOH = 80/20, flow rate = 1.0 mL/min, λ = 254 nm, retention time: t_{major} = 9.5 min, t_{minor} = 11.8 min;

HRMS (ESI) m/z: $[M+Na]^+$ calculated for $C_{17}H_{18}O_4^+$: 309.1097, found: 309.1102;

$[\alpha]_D^{25}$ = -18.95 (c = 0.08, $CHCl_3$).

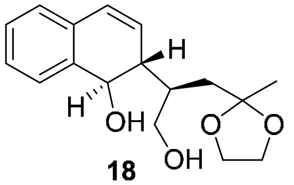

18

^{1}H NMR (600 MHz, $CDCl_3$) δ 7.48 – 7.39 (m, 1H), 7.21 – 7.11 (m, 2H), 6.98 (dd, *J* = 7.0, 1.7 Hz, 1H), 6.44 (dd, *J* = 9.8, 2.5 Hz, 1H), 5.73 (dd, *J* = 9.7, 3.3 Hz, 1H), 4.69 (d, *J* = 9.9 Hz, 1H), 3.99 – 3.86 (m, 4H), 3.52 (qd, *J* = 10.6, 6.4 Hz, 2H), 2.92 (d, *J* = 25.0 Hz, 2H), 2.62 (dtd, *J* = 9.6, 3.5, 2.5 Hz, 1H), 2.07 (dt, *J* = 9.6, 3.6 Hz, 1H), 1.92 (dt, *J* = 15.3, 2.9 Hz, 1H), 1.73 (ddd, *J* = 15.3, 7.3, 2.7 Hz, 1H), 1.25 (s, 3H);

^{13}C NMR (151 MHz, $CDCl_3$) δ 137.4, 132.6, 128.6, 128.6, 128.0, 127.8, 127.6, 126.0, 125.3, 110.2, 65.3, 65.3, 64.6, 64.3, 45.2, 45.2, 37.7, 36.2, 36.1, 23.7;

HPLC analysis: Daicel CHIRALPAK® OD-H, *n*-hexane/*i*-PrOH = 70/30, flow rate = 1.0 mL/min, λ = 254 nm, retention time: t_{minor} = 11.0 min, t_{major} = 13.0 min;

HRMS (ESI) m/z: $[M+Na]^+$ calculated for $C_{17}H_{22}O_4Na^+$: 313.1410, found: 313.1410;

$[\alpha]_D^{25}$ = +130.57 (c = 0.07, $CHCl_3$).

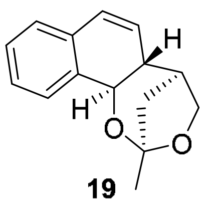

19

^{1}H NMR (500 MHz, $CDCl_3$) δ 7.45 (d, *J* = 7.4 Hz, 1H), 7.26 – 7.18 (m, 2H), 7.08 (dd, *J* = 7.4, 1.5 Hz, 1H), 6.54 (dd, *J* = 9.5, 3.0 Hz, 1H), 5.79 (dd, *J* = 9.5, 2.3 Hz, 1H), 4.77 (d, *J* = 14.5 Hz, 1H), 4.24 (d, *J* = 8.2 Hz, 1H), 3.98 (ddd, *J* = 8.2, 4.1, 1.3 Hz, 1H), 2.61 (dq, *J* = 4.0, 1.9 Hz, 1H), 2.44 (dq, *J* = 14.6, 1.5 Hz, 1H), 1.91 – 1.88 (m, 2H), 1.61 (s, 3H);

^{13}C NMR (126 MHz, CDCl$_3$) δ 138.0, 132.7, 130.32, 129.6, 127.6, 126.6, 125.8, 121.7, 106.0, 72.3, 70.6, 43.1, 42.2, 38.4, 23.3;

HPLC analysis: Daicel CHIRALPAK® OD-H, *n*-hexane/*i*-PrOH = 98/2, flow rate = 1.0 mL/min, λ = 254 nm, retention time: t_{minor} = 5.4 min, t_{major} = 6.3 min;

HRMS (ESI) m/z: $[M+Na]^+$ calculated for $C_{15}H_{16}O_2Na^+$: 251.1043, found: 251.1042;

$[\alpha]_D^{25}$ = +88.70 (c = 0.1, $CHCl_3$).

6.4 Late-stage Cα functionalization of γ-lactone for quaternary stereocenters

Upon protection of the ketone carbonyl group in product **3a**, we treated it with a series of electrophiles under basic conditions, affording products bearing a quaternary carbon at the Cα-position. The structure of terpenoid tricyclic γ-lactones bearing all-carbon quaternary stereocenter (**IV**) widely present in various natural products and has a wide range of biological activities.[36] Such as artemiprincepsolides D-F,[37] tetrachyrin,[38] rosenonolactones, desoxyrosenonolactone, rosololactone,[39] vlasouliolide D/H[34], borneolactone B,[40] coleon A lactone[41], BE-42472B,[42] lysidice E,[43] and tricyclic core of galanthamine.[44]

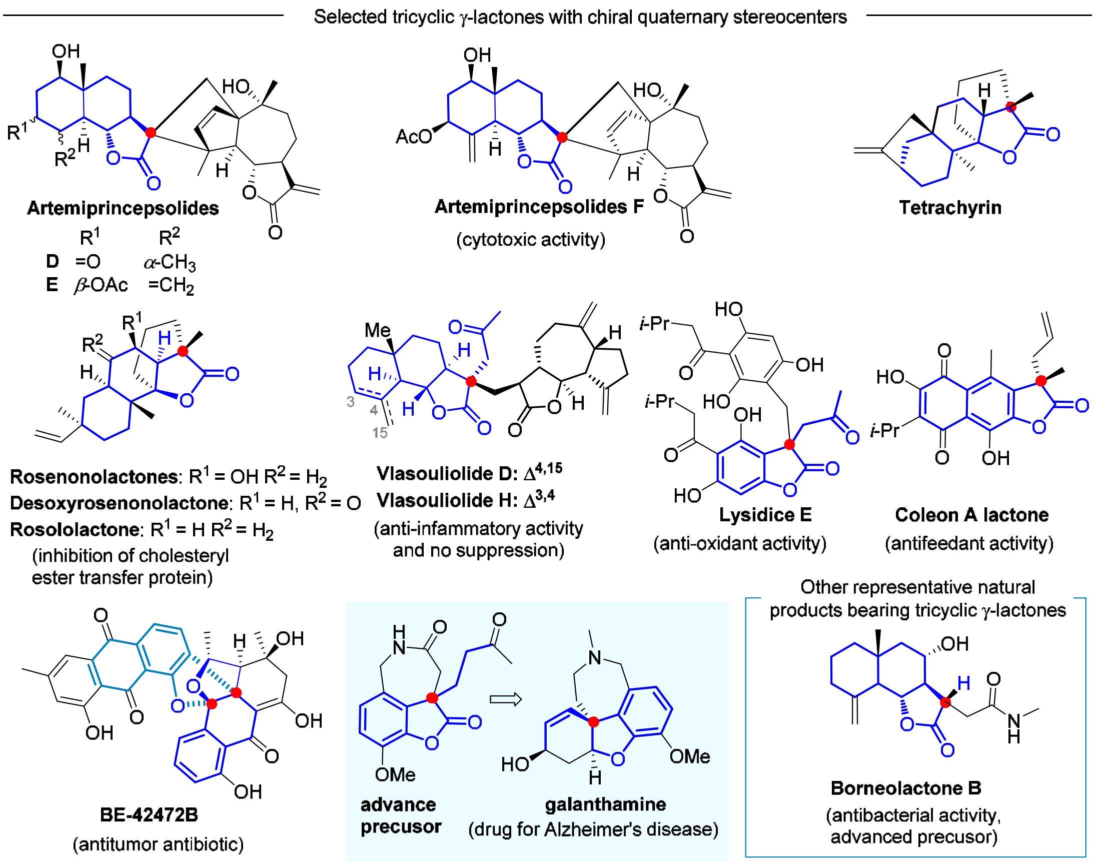

Figure S12. Representative natural products bearing tricyclic γ-lactones.

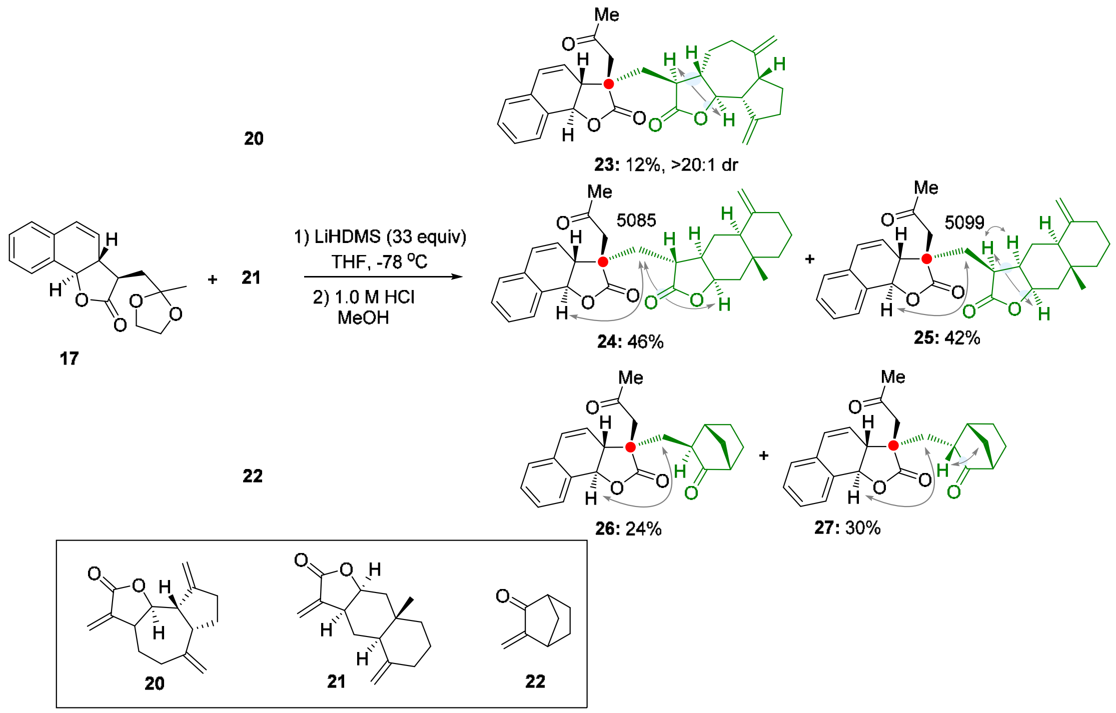

The ketal derivative (0.1 mmol) was dissolved in 1 mL of dry tetrahydrofuran (THF), and carried out under a nitrogen atmosphere and at -78 °C. After reaching this temperature, 33 equiv of LiHDMS was slowly added to the mixture and allowed to react for 30 min. After the enolate was formed, 1.0 equiv of the second monomer **20**, **21** and **22** (dissolved in THF) was added and the mixture was stirred at -78 °C for 2 h. Then, aqueous NH_4Cl was added to quench the reaction and warm to room temperature. The solution was extracted with ethyl acetate (EA) for three times. The organic layer was combined, washed with brine, dried with Na_2SO_4 and concentrated in vacuum. The residue was then purified by column chromatography to give the corresponding product.

And then the product (0.1 mmol) was dissolved in 20 mL of methanol in a 50 mL flask, 5 mL of 1.0 M HCl was added. The mixture was stirred overnight at room temperature. The crude product was extracted three times with ethyl acetate, dried with anhydrous Na_2SO_4, and evaporated under reduced pressure. The products were purified by HPLC and can be obtained with quantitative yield.[34]

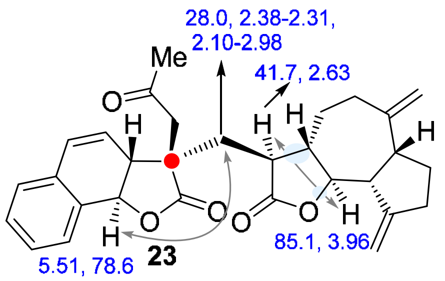

23

White solid, 5.7 mg, 12% yield, >20:1 dr;

^{1}H NMR (600 MHz, $CDCl_3$) δ 7.49 (dt, J = 7.2, 1.4 Hz, 1H), 7.32 – 7.26 (m, 2H), 7.16 (dd, J = 7.1, 1.6 Hz, 1H), 6.59 (dd, J = 9.6, 3.1 Hz, 1H), 6.42 (dd, J = 9.6, 2.4 Hz, 1H), 5.51 (d, J = 14.7 Hz, 1H), 5.15 (q, J = 2.4 Hz, 1H), 5.04 (q, J = 2.3 Hz, 1H), 4.89 (d, J = 1.3 Hz, 1H), 4.79 (d, J = 1.3 Hz, 1H), 3.96 (t, J = 9.4 Hz, 1H), 3.14 (dt, J = 14.7, 2.7 Hz, 1H), 3.08 (d, J = 18.5 Hz, 1H), 2.84 (td, J = 8.2, 4.5 Hz, 1H), 2.80 – 2.70 (m, 2H), 2.63 (ddd, J = 12.3, 8.7, 1.3 Hz, 1H), 2.57 – 2.44 (m, 3H), 2.38 – 2.31 (m, 1H), 2.16 (s, 3H), 2.10 – 1.98 (m, 4H), 1.97 – 1.90 (m, 1H), 1.85 (dddd, J = 13.2, 8.8, 5.7, 4.4 Hz, 1H), 1.39 – 1.33 (m, 1H);

^{13}C NMR (151 MHz, $CDCl_3$) δ 205.7, 178.9, 177.9, 151.4, 149.5, 134.4, 132.0, 130.4, 128.1, 127.7, 126.6, 125.0, 122.0, 112.1, 109.3, 85.1, 78.6, 51.8, 50.1, 48.7, 47.4, 47.0, 46.4, 41.7, 37.6, 32.4, 32.3, 31.1, 30.1, 28.0;

HRMS (ESI) m/z: $[M+H]^+$ calculated for $C_{30}H_{33}O_5^+$: 473.2323, found: 473.2330;

$[\alpha]_D^{25}$ = -20.50 (c = 0.07, $CHCl_3$).

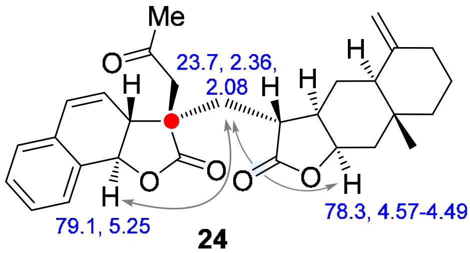

24

White solid, 20.8 mg, 46% yield;

^{1}H NMR (600 MHz, $CDCl_3$) δ 7.48 – 7.42 (m, 1H), 7.34 – 7.27 (m, 2H), 7.21 – 7.16 (m, 1H), 6.62 (dd, J = 9.6, 3.1 Hz, 1H), 6.45 (dd, J = 9.5, 2.5 Hz, 1H), 5.25 (d, J = 14.8 Hz, 1H), 4.73 (q, J = 1.6 Hz, 1H), 4.57 – 4.49 (m, 1H), 4.38 (q, J = 1.6 Hz, 1H), 3.35 (dt, J = 14.8, 2.8 Hz, 1H), 3.14 (d, J = 18.7 Hz, 1H), 3.08 (td, J = 6.7, 4.2 Hz, 1H), 2.88 (d, J = 18.7 Hz, 1H), 2.52 (dtd, J = 12.3, 6.1, 4.0 Hz, 1H), 2.36 (dd, J = 15.3,

4.2 Hz, 1H), 2.30 (dt, J = 13.2, 2.2 Hz, 1H), 2.23 – 2.11 (m, 4H), 2.08 (dd, J = 15.3, 6.9 Hz, 1H), 1.97 (td, J = 12.8, 5.6 Hz, 1H), 1.80 – 1.75 (m, 1H), 1.53 (ddd, J = 13.5, 11.3, 4.0 Hz, 2H), 1.49 – 1.41 (m, 2H), 1.23 (td, J = 13.3, 4.9 Hz, 2H), 1.09 (q, J = 12.6 Hz, 1H), 0.77 (s, 3H);

^{13}C NMR (151 MHz, $CDCl_3$) δ 205.1, 179.2, 178.4, 149.1, 134.2, 132.2, 130.3, 128.1, 127.9, 126.9, 124.8, 121.6, 106.5, 79.1, 78.3, 48.9, 46.4, 46.3, 45.8, 42.6, 42.1, 41.4, 39.6, 36.7, 34.8, 30.6, 23.7, 22.6, 21.5, 17.7;

HRMS (ESI) m/z: $[M+Na]^+$ calculated for $C_{30}H_{34}O_5Na^+$: 497.2298, found: 497.2298;

$[\alpha]_D^{25}$ = +4.5 (c = 0.07, $CHCl_3$).

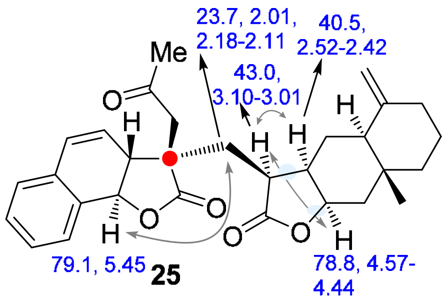

25

White solid, 20.8 mg, 42% yield;

^{1}H NMR (600 MHz, $CDCl_3$) δ 7.49 – 7.42 (m, 1H), 7.34 – 7.26 (m, 2H), 7.20 – 7.14 (m, 1H), 6.61 (dd, J = 9.6, 3.1 Hz, 1H), 6.43 (dd, J = 9.5, 2.4 Hz, 1H), 5.45 (d, J = 14.7 Hz, 1H), 4.81 (q, J = 1.6 Hz, 1H), 4.57 – 4.44 (m, 2H), 3.19 (dt, J = 14.8, 2.8 Hz, 1H), 3.10 – 3.01 (m, 2H), 2.80 (d, J = 18.4 Hz, 1H), 2.52 – 2.42 (m, 2H), 2.35 (ddt, J = 13.1, 4.4, 2.0 Hz, 1H), 2.18 – 2.11 (m, 4H), 2.01 (td, J = 12.7, 5.8 Hz, 1H), 1.84 – 1.78 (m, 1H), 1.59 – 1.52 (m, 4H), 1.51 – 1.44 (m, 1H), 1.29 – 1.20 (m, 2H), 1.10 (q, J = 12.6 Hz, 1H), 0.78 (s, 3H);

^{13}C NMR (126 MHz, $CDCl_3$) δ 205.2, 178.9, 177.9, 149.1, 134.3, 132.0, 130.4, 128.1, 127.8, 126.9, 124.8, 121.7, 106.6, 79.1, 77.8, 49.7, 47.6, 46.6, 43.0, 42.1, 41.4, 40.5, 36.7, 34.8, 30.9, 23.7, 22.6, 21.6, 17.7;

HRMS (ESI) m/z: $[M+Na]^+$ calculated for $C_{30}H_{34}O_5Na^+$: 497.2298, found: 497.2297;

$[\alpha]_D^{25}$ = -3.9 (c = 0.1, $CHCl_3$).

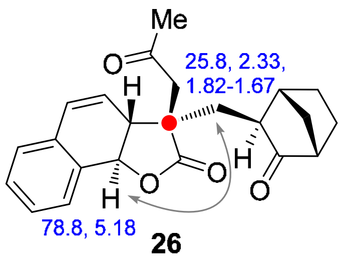

26

White solid, 9.8 mg, 24% yield;

^{1}H NMR (500 MHz, $CDCl_3$) δ 7.40 (m, 1H), 7.31 – 7.26 (m, 2H), 7.16 (dd, J = 7.0, 1.8 Hz, 1H), 6.58 (dd, J = 9.6, 3.0 Hz, 1H), 6.47 (dd, J = 9.6, 2.4 Hz, 1H), 5.37 (d, J = 14.8 Hz, 1H), 3.16 (d, J = 18.7 Hz, 1H), 3.11 (dt, J = 14.8, 2.8 Hz, 1H), 2.75 (d, J = 18.7 Hz, 1H), 2.67 (dq, J = 4.5, 2.1 Hz, 1H), 2.64 (d, J = 4.9 Hz, 1H), 2.32 (dd, J = 8.8, 4.1 Hz, 1H), 2.26 (dd, J = 15.2, 3.2 Hz, 1H), 2.15 (s, 3H), 1.90 (dd, J = 15.2, 9.1 Hz, 1H), 1.83 (tt, J = 12.5, 4.8 Hz, 1H), 1.77 (dp, J = 10.4, 2.0 Hz, 1H), 1.67 – 1.60 (m, 2H), 1.55 (ddt, J = 11.1, 4.7, 2.3 Hz, 1H), 1.38 (dddd, J = 13.3, 9.0, 4.7, 2.1 Hz, 1H);

^{13}C NMR (151 MHz, $CDCl_3$) δ 219.2, 205.4, 179.2, 134.4, 132.2, 130.0, 128.0, 127.8, 126.83, 125.3, 121.6, 78.8, 50.2, 49.1, 48.9, 46.8, 46.1, 40.2, 37.2, 30.6, 25.9, 25.8, 21.4;

HRMS (ESI) m/z: $[M+Na]^+$ calculated for $C_{23}H_{24}O_4Na^+$: 387.1567, found: 387.1568;

$[\alpha]_D^{25}$ = -40.72 (c = 0.083, $CHCl_3$).

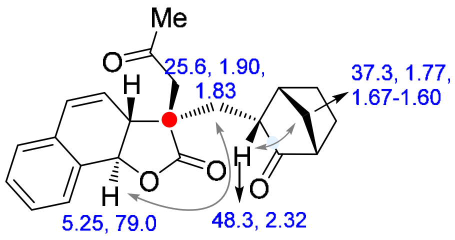

27

White solid, 9.8 mg, 30% yield;

^{1}H NMR (600 MHz, $CDCl_3$) δ 7.47 – 7.40 (m, 1H), 7.31 – 7.26 (m, 2H), 7.16 (dd, *J* = 7.0, 1.8 Hz, 1H), 6.58 (dd, *J* = 9.6, 3.0 Hz, 1H), 6.47 (dd, *J* = 9.6, 2.4 Hz, 1H), 5.37 (d, *J* = 14.8 Hz, 1H), 3.16 (d, *J* = 18.7 Hz, 1H), 3.11 (dt, *J* = 14.8, 2.8 Hz, 1H), 2.75 (d, *J* = 18.7 Hz, 1H), 2.67 (dq, *J* = 4.5, 2.1 Hz, 1H), 2.64 (d, *J* = 4.9 Hz, 1H), 2.32 (dd, *J* = 8.8, 4.1 Hz, 1H), 2.26 (dd, *J* = 15.2, 3.2 Hz, 1H), 2.15 (s, 3H), 1.90 (dd, *J* = 15.2,

9.1 Hz, 1H), 1.83 (tt, J = 12.5, 4.8 Hz, 1H), 1.77 (dp, J = 10.4, 2.0 Hz, 1H), 1.67 – 1.60 (m, 2H), 1.55 (ddt, J = 11.1, 4.7, 2.3 Hz, 1H), 1.38 (dddd, J = 13.3, 9.0, 4.7, 2.1 Hz, 1H);

^{13}C NMR (151 MHz, $CDCl_3$) δ 218.1, 205.4, 179.0, 134.6, 132.2, 130.0, 128.0, 127.8, 126.81, 125.5, 121.6, 79.0, 50.2, 50.1, 49.4, 48.3, 46.9, 40.2, 37.3, 30.8, 25.6, 25.2, 21.2;

HRMS (ESI) m/z: $[M+Na]^+$ calculated for $C_{23}H_{24}O_4Na^+$: 387.1567, found: 387.1567;

$[\alpha]_D^{25}$ = +39.52 (c = 0.083, $CHCl_3$).

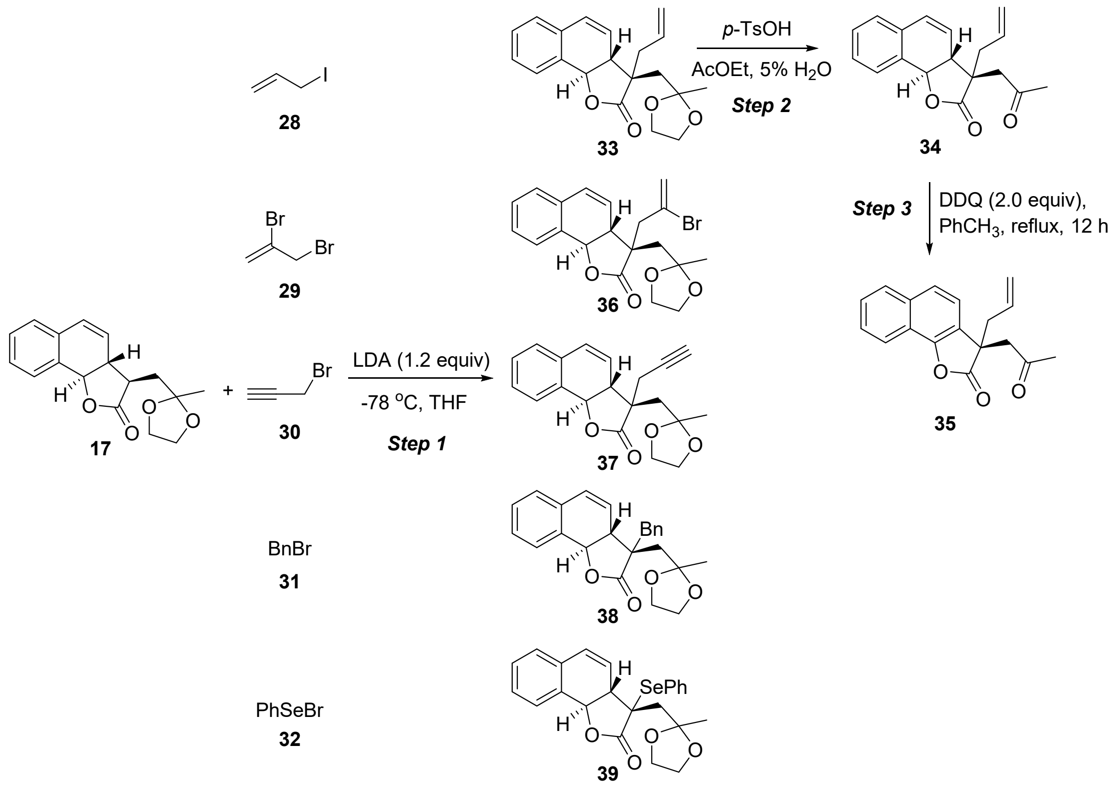

Step 1: A solution of freshly prepared lithium diisopropylamide (1.2 equiv.; 1.0 M in anhydrous THF) was added dropwise to the ketal derivatives (1.0 equiv) in THF (0.1 M) under -78 °C and Ar protection. The whole reaction was stirred for 1 h before the addition of electrophile (1.2 equiv) dissolved with few THF. When the starting material was full consumed monitored by TLC, aqueous NH_4Cl was added to quench the reaction and warm to room temperature. The solution was extracted with ethyl acetate (EA) for three times. The organic layer was combined, washed with brine, dried with Na_2SO_4 and concentrated in vacuum. The residue was then purified by

column chromatography to give the product.[45]

Step 2: The **33** (32.6 mg, 0.1 mmol, 1.0 equiv) was dissolved in 0.95 mL of ethyl acetate, and a catalytic amount of *p*-TsOH (32.6 mg, 0.1 mmol, 20 mol%) was added. Then, 50 μL of distilled water was added. The mixture was stirred overnight at room temperature. The crude product was extracted three times with ethyl acetate, dried with anhydrous Na_2SO_4, and evaporated under reduced pressure. The products were purified by flash column chromatography on silica gel to yield the pure product **34** (21.2 mg, 75% yield, 90% ee).[34]

Step 3: To a flame-dried and N_2-purged schlenk tube were added **34** (28.2 mg, 0.1 mmol, 1.0 equiv) and DDQ (45.4 mg, 0.2 mmol, 2.0 equiv). The vial was then sealed, purged and backfilled with N_2 three times before adding toluene (1.0 mL) at room temperature. The resulting mixture was reflux for 12 hours. After concentrated under reduced pressure, the residue was purified by column chromatography on silica gel to give **35** (23 mg, 82% yield, 89% ee).[46]

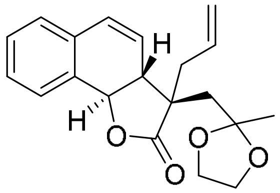

Compound **33**: 55% yield, 20:1 dr, 90% ee.

^{1}H NMR (500 MHz, CDCl$_3$) δ 7.51-7.38 (m, 1H), 7.33-7.27 (m, 2H), 7.21-7.13 (m, 1H), 6.58 (dd, *J* = 9.5, 3.1 Hz, 1H), 6.23 (dd, *J* = 9.5, 2.5 Hz, 1H), 5.91 (dddd, *J* = 16.9, 10.3, 8.1, 6.7 Hz, 1H), 5.28 (d, *J* = 15.0 Hz, 1H), 5.23-5.12 (m, 2H), 3.94-3.84 (m, 2H), 3.83-3.75 (m, 2H), 3.45 (dt, *J* = 15.0, 2.8 Hz, 1H), 2.56 (ddt, *J* = 14.3, 6.7, 1.4 Hz, 1H), 2.44 (ddt, *J* = 14.2, 8.2, 1.1 Hz, 1H), 2.32 (d, *J* = 15.3 Hz, 1H), 2.11 (d, *J* = 15.3 Hz, 1H), 1.28 (s, 3H);

^{13}C NMR (126 MHz, CDCl$_3$) δ 179.3, 135.0, 132.4, 132.3, 129.5, 127.9, 127.6, 126.9, 125.3, 121.6, 119.6, 108.7, 78.3, 64.1, 63.7, 47.3, 46.6, 41.8, 36.4, 25.8;

HPLC analysis: Daicel CHIRALPAK® ID, *n*-hexane/*i*-PrOH = 80/20, flow rate =

1.0 mL/min, λ = 254 nm, retention time: t $_{major}$ = 9.0 min, t $_{minor}$ = 10.6 min;

HRMS (ESI) m/z: $[M+Na]^+$ calculated for $C_{20}H_{22}O_4Na^+$: 349.1410, found: 349.1415;

$[\alpha]_D^{25}$ = -0.60 (c = 0.1, $CHCl_3$).

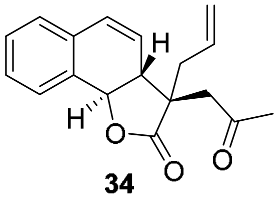

34

Compound **34**: 75% yield, 20:1 dr, 90% ee.

^{1}H NMR (500 MHz, CDCl$_3$) δ 7.43 – 7.31 (m, 1H), 7.25 – 7.16 (m, 2H), 7.12 – 7.05 (m, 1H), 6.49 (dd, *J* = 9.6, 3.1 Hz, 1H), 6.42 (dd, *J* = 9.6, 2.4 Hz, 1H), 5.80 (dddd, *J* = 16.7, 10.1, 8.4, 6.6 Hz, 1H), 5.21 (d, *J* = 14.8 Hz, 1H), 5.16 – 5.06 (m, 2H), 3.11 (dt, *J* = 14.9, 2.7 Hz, 1H), 3.02 (d, *J* = 18.6 Hz, 1H), 2.63 (d, *J* = 18.6 Hz, 1H), 2.57 – 2.47 (m, 2H), 2.05 (s, 3H);

^{13}C NMR (126 MHz, CDCl$_3$) δ 205.4, 178.7, 134.5, 132.1, 129.7, 127.9, 127.7, 126.8, 125.6, 121.5, 119.8, 78.8, 48.9, 47.1, 33.8, 30.7;

HPLC analysis: Daicel CHIRALCEL® OD-H, *n*-hexane/*i*-PrOH = 95/5, flow rate = 1.0 mL/min, λ = 254 nm, retention time: t $_{minor}$ = 14.9 min, t $_{major}$ = 17.1 min;

HRMS (ESI) m/z: $[M+Na]^+$ calculated for $C_{18}H_{18}O_3Na^+$: 305.1148, found: 305.1151;

$[\alpha]_D^{25}$ = +52.71 (c = 0.07, $CHCl_3$).

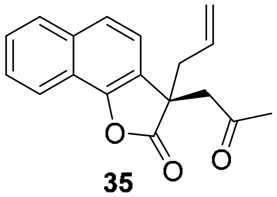

35

Compound **35**: 82% yield, 20:1 dr, 89% ee.

^{1}H NMR (500 MHz, CDCl$_3$) δ 8.06 – 7.97 (m, 1H), 7.76 (d, J = 8.1 Hz, 1H), 7.55 (d, J = 8.3 Hz, 1H), 7.45 (dddd, J = 24.7, 8.2, 6.8, 1.3 Hz, 2H), 7.16 (d, J = 8.3 Hz, 1H), 5.49 – 5.35 (m, 1H), 5.05 – 4.90 (m, 2H), 3.28 – 3.09 (m, 2H), 2.60 – 2.44 (m, 2H), 1.95 (s, 3H);

^{13}C NMR (126 MHz, CDCl$_3$) δ 203.6, 179.4 149.2 134.0, 130.4, 128.0, 126.6, 126.6,

123.9, 123.3, 121.5, 120.6, 119.8, 119.2, 49.8, 49.3, 42.4, 29.6;

HPLC analysis: Daicel CHIRALPAK® AS-H, *n*-hexane/*i*-PrOH = 70/30, flow rate = 1.0 mL/min, λ = 254 nm, retention time: t $_{minor}$ = 7.4 min, t $_{major}$ = 9.0 min;

HRMS (ESI) m/z: $[M+Na]^+$ calculated for $C_{18}H_{16}O_3Na^+$: 303.0992, found: 303.0991;

$[\alpha]_D^{25}$ = -41.10 (c = 0.08, $CHCl_3$).

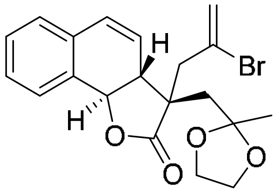

Compound **36**: 22% yield, 20:1 dr, 89% ee.

^{1}H NMR (500 MHz, $CDCl_3$) δ 7.46 (dt, *J* = 6.9, 1.2 Hz, 1H), 7.33 – 7.27 (m, 2H), 7.21 – 7.16 (m, 1H), 6.58 (dd, *J* = 9.5, 3.1 Hz, 1H), 6.21 (dd, *J* = 9.5, 2.5 Hz, 1H), 5.83 (d, *J* = 1.8 Hz, 1H), 5.68 (d, *J* = 1.9 Hz, 1H), 5.35 (d, *J* = 14.9 Hz, 1H), 3.98 – 3.79 (m, 4H), 3.46 (dt, *J* = 15.0, 2.8 Hz, 1H), 3.09 – 2.95 (m, 2H), 2.39 (s, 2H), 1.30 (s, 3H);

^{13}C NMR (126 MHz, $CDCl_3$) δ 178.6, 134.8, 132.2, 129.6, 128.0, 127.7, 126.9, 126.9, 125.1, 123.0, 121.8, 108.8, 78.4, 63.9, 63.7, 47.8, 47.6, 43.5, 42.3, 25.7;

HPLC analysis: Daicel CHIRALPAK® ID, *n*-hexane/*i*-PrOH = 80/20, flow rate = 1.0 mL/min, λ = 254 nm, retention time: t $_{major}$ = 11.3 min, t $_{minor}$ = 14.2 min;

HRMS (ESI) m/z: $[M+Na]^+$ calculated for $C_{20}H_{21}O_4BrNa^+$: 427.0515, found: 427.0522;

$[\alpha]_D^{25}$ = +17.86 (c = 0.07, $CHCl_3$).

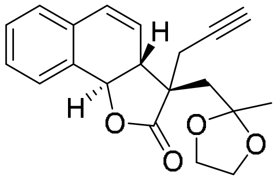

Compound **37**: 49% yield, 20:1 dr, 89% ee.

^{1}H NMR (500 MHz, $CDCl_3$) δ 7.49 – 7.42 (m, 1H), 7.33 – 7.27 (m, 2H), 7.22 – 7.17 (m, 1H), 6.59 (dd, *J* = 9.6, 3.1 Hz, 1H), 6.45 (dd, *J* = 9.6, 2.5 Hz, 1H), 5.28 (d, *J* =

15.0 Hz, 1H), 3.95 – 3.85 (m, 2H), 3.84 – 3.76 (m, 2H), 3.43 (dt, *J* = 15.1, 2.8 Hz, 1H), 2.75 (dd, *J* = 17.1, 2.7 Hz, 1H), 2.61 – 2.47 (m, 2H), 2.35 (d, *J* = 15.3 Hz, 1H), 2.18 (t, *J* = 2.7 Hz, 1H), 1.31 (s, 3H);

^{13}C NMR (126 MHz, $CDCl_3$) δ 178.7, 134.7, 132.2, 129.5, 128.0, 127.7, 126.9, 124.9, 121.7, 108.5, 79.1, 78.5, 72.6, 64.2, 63.7, 46.5, 46.2, 41.6, 25.7, 21.8;

HPLC analysis: Daicel CHIRALPAK® ID, *n*-hexane/*i*-PrOH = 80/20, flow rate = 1.0 mL/min, λ = 254 nm, retention time: t_{major} = 12.2 min, t_{minor} = 13.8 min;

HRMS (ESI) m/z: $[M+Na]^+$ calculated for $C_{20}H_{20}O_4Na^+$: 347.1254, found: 347.1258;

$[\alpha]_D^{25}$ = +1.30 (c = 0.1, $CHCl_3$).

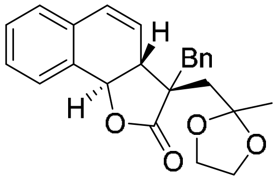

Compound **38**: 34% yield, 20:1 dr, 89% ee.

^{1}H NMR (500 MHz, $CDCl_3$) δ 7.38 (dd, *J* = 5.3, 3.6 Hz, 1H), 7.30 – 7.20 (m, 7H), 7.16 (dt, *J* = 4.4, 3.3 Hz, 1H), 6.57 (dd, *J* = 9.5, 3.1 Hz, 1H), 6.28 (dd, *J* = 9.5, 2.5 Hz, 1H), 4.95 (d, *J* = 14.9 Hz, 1H), 3.95 – 3.77 (m, 4H), 3.51 (dt, *J* = 15.1, 2.8 Hz, 1H), 3.14 (d, *J* = 13.9 Hz, 1H), 3.04 (d, *J* = 13.9 Hz, 1H), 2.23 (s, 2H), 1.27 (s, 3H);

^{13}C NMR (126 MHz, $CDCl_3$) δ 179.5, 135.8, 135.3, 132.3, 130.9, 129.2, 128.3, 127.8, 127.5, 127.1, 126.8, 125.7, 121.6, 108.8, 78.4, 64.0, 63.7, 48.9, 46.9, 42.9, 39.0, 25.8;

HPLC analysis: Daicel CHIRALPAK® ID, *n*-hexane/*i*-PrOH = 80/20, flow rate = 1.0 mL/min, λ = 254 nm, retention time: t_{major} = 13.3 min, t_{minor} = 15.1 min;

HRMS (ESI) m/z: $[M+Na]^+$ calculated for $C_{24}H_{24}O_4Na^+$: 399.1567, found: 399.1570;

$[\alpha]_D^{25}$ = -174.00 (c = 0.07, $CHCl_3$).

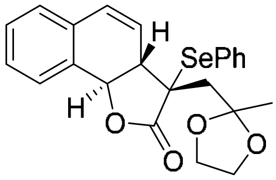

Compound **39**: 45% yield, 20:1 dr, 90% ee.

^{1}H NMR (500 MHz, CDCl$_3$) δ 7.60 (dd, J = 8.1, 1.4 Hz, 2H), 7.41 (dt, J = 7.0, 1.5 Hz, 1H), 7.38 – 7.34 (m, 1H), 7.30 – 7.20 (m, 4H), 7.16 – 7.12 (m, 1H), 6.58 (dd, J = 9.5, 3.0 Hz, 1H), 6.25 (dd, J = 9.4, 2.3 Hz, 1H), 5.50 (d, J = 14.5 Hz, 1H), 3.80 – 3.64 (m, 4H), 3.44 (dt, J = 14.4, 2.7 Hz, 1H), 2.48 (d, J = 15.1 Hz, 1H), 2.31 (d, J = 15.1 Hz, 1H), 1.17 (s, 3H);

^{13}C NMR (126 MHz, CDCl$_3$) δ 175.4, 138.3, 134.7, 132.4, 130.2, 129.9, 129.1, 128.0, 127.6, 127.1, 124.6, 124.1, 121.8, 108.6, 78.8, 64.3, 64.0, 49.3, 48.0, 43.4, 25.7;

HPLC analysis: Daicel CHIRALCEL® OD-H, *n*-hexane/*i*-PrOH = 95/5, flow rate = 1.0 mL/min, λ = 254 nm, retention time: t_{major} = 7.6 min, t_{minor} = 9.5 min;

HRMS (ESI) m/z: $[M+Na]^+$ calculated for $C_{23}H_{22}O_4SeNa^+$: 465.0576, found: 465.0578;

$[\alpha]_D^{25}$ = -8.95 (c = 0.08, $CHCl_3$).

7. Single-crystal x-ray diffraction date

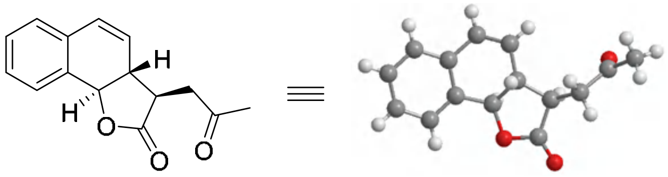

X-ray of (*R*,*R*,*R*)-**3a**

(*R*,*R*,*R*)-**3a** (CCDC NO.: 2330703)

Identification code	cu_230615B	
Chemical formula	$C_{15}H_{14}O_3$	
Formula weight	242.26 g/mol	
Wavelength	1.54178 Å	
Crystal size	0.180 x 0.220 x 0.240 mm	
Crystal system	monoclinic	
Space group	P 1 21 1	
Unit cell dimensions	a = 5.1343(4) Å	α = 90°
	b = 9.3898(7) Å	β = 100.387(2)°
	c = 12.4989(10) Å	γ = 90°
Volume	592.70(8) Å^3	
Z	2	
Density (calculated)	1.357 g/cm^3	

Absorption coefficient	0.766 mm^{-1}
F(000)	256
Theta range for data collection	3.60 to 77.31°
Index ranges	-6<=h<=6, -11<=k<=11, -15<=l<=15
Reflections collected	14995
Independent reflections	2439 [R(int) = 0.0380]
Max. and min. transmission	0.7541 and 0.6271
Structure solution technique	direct methods
Structure solution program	SHELXT 2018/2 (Sheldrick, 2018)
Refinement method	Full-matrix least-squares on F^2
Refinement program	SHELXL 2018/3 (Sheldrick, 2015)
Function minimized	Σ w(F_o^2- F_c^2)^2
Data / restraints / parameters	2439 / 1 / 164
Goodness-of-fit on F^2	1.038
Final R indices	2433 data; I>2σ(I) R1 = 0.0336, wR2 = 0.0875
	all data R1 = 0.0337, wR2 = 0.0875
Weighting scheme	w=1/[$\sigma^2(F_o^2)+(0.0588P)^2+0.1215P$] where P=($F_o^2+2F_c^2$)/3
Absolute structure parameter	-0.14(14)
Largest diff. peak and hole	0.194 and -0.177 eÅ^{-3}
R.M.S. deviation from mean	0.039 eÅ^{-3}

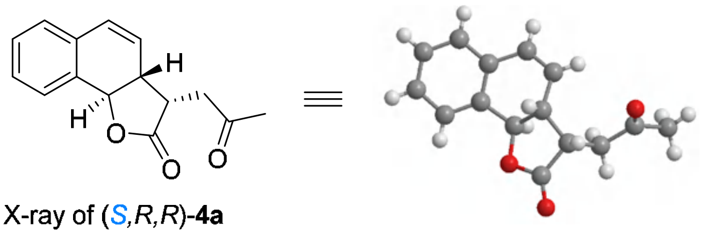

X-ray of (*S*,*R*,*R*)-**4a**

(*S,R,R*)-**4a** (CCDC NO.: 2330704)

Identification code	cu_240201D
Chemical formula	$C_{15}H_{14}O_3$
Formula weight	242.26 g/mol
Wavelength	1.54178 Å
Crystal size	0.100 x 0.200 x 0.210 mm
Crystal system	monoclinic
Space group	P 1 21 1
Unit cell dimensions	a = 5.4496(8) Å α = 90°

	b = 9.7142(15) Å	β = 99.517(5)°
	c = 11.8110(18) Å	γ = 90°
Volume	616.65(16) $Å^3$	
Z	2	
Density (calculated)	1.305 g/cm^3	
Absorption coefficient	0.736 mm^{-1}	
F(000)	256	
Theta range for data collection	8.88 to 77.69°	
Index ranges	-6<=h<=6, -12<=k<=11, -14<=l<=14	
Reflections collected	14724	
Independent reflections	2548 [R(int) = 0.0309]	
Max. and min. transmission	0.7541 and 0.5725	
Structure solution technique	direct methods	
Structure solution program	SHELXT 2018/2 (Sheldrick, 2018)	
Refinement method	Full-matrix least-squares on F^2	
Refinement program	SHELXL 2018/3 (Sheldrick, 2015)	
Function minimized	$\Sigma\ w(F_o^2 - F_c^2)^2$	
Data / restraints / parameters	2548 / 1 / 163	
Goodness-of-fit on F^2	1.055	
Final R indices	2492 data; I>2σ(I)	R1 = 0.0319, wR2 = 0.0847
	all data	R1 = 0.0329, wR2 = 0.0856
Weighting scheme	w=1/[σ2(Fo2)+(0.0519P)2+0.0354P]<br>where P=(Fo2+2Fc2)/3	
Absolute structure parameter	-0.03(9)	
Largest diff. peak and hole	0.103 and -0.120 eÅ-3	
R.M.S. deviation from mean	0.032 eÅ-3	

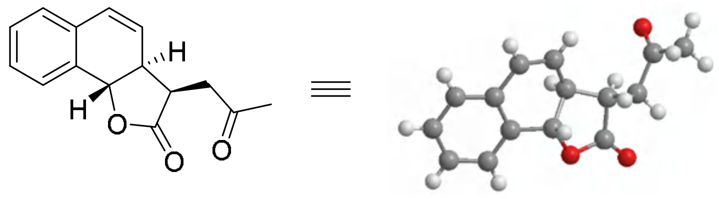

X-ray of (*R*,*S*,*S*)-**4a**

(*R,S,S*)-**4a** (CCDC NO.: 2333474)

Identification code	cu_230614B

Chemical formula	$C_{15}H_{14}O_3$
Formula weight	242.26 g/mol
Wavelength	1.54178 Å
Crystal size	0.180 x 0.190 x 0.210 mm
Crystal system	monoclinic
Space group	P 1 21 1
Unit cell dimensions	a = 5.3990(4) Å α = 90°
	b = 9.4977(8) Å β = 99.880(2)°
	c = 11.7771(9) Å γ = 90°
Volume	594.95(8) $Å^3$
Z	2
Density (calculated)	1.352 g/cm^3
Absorption coefficient	0.763 mm^{-1}
F(000)	256

Theta range for data collection	7.64 to 77.25°
Index ranges	-6<=h<=5, -11<=k<=11, -14<=l<=14
Reflections collected	9302
Independent reflections	2440 [R(int) = 0.0220]
Max. and min. transmission	0.7541 and 0.6158
Structure solution technique	direct methods
Structure solution program	SHELXT 2018/2 (Sheldrick, 2018)
Refinement method	Full-matrix least-squares on F^2
Refinement program	SHELXL 2018/3 (Sheldrick, 2015)
Function minimized	$\Sigma w(F_o^2 - F_c^2)^2$
Data / restraints / parameters	2440 / 1 / 164
Goodness-of-fit on F^2	1.059
Final R indices	2431 data; I>2σ(I) R1 = 0.0272, wR2 = 0.0732
	all data R1 = 0.0273, wR2 = 0.0734
Weighting scheme	$w=1/[\sigma^2(F_o^2)+(0.0455P)^2+0.0994P]$ where $P=(F_o^2+2F_c^2)/3$
Absolute structure parameter	0.02(5)
Largest diff. peak and hole	0.206 and -0.165 $eÅ^{-3}$
R.M.S. deviation from mean	0.034 $eÅ^{-3}$

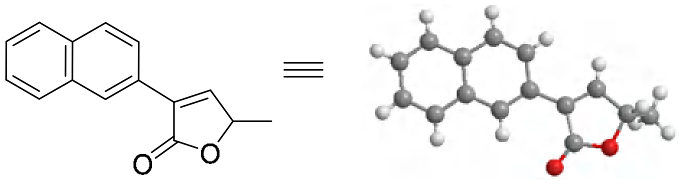

9 (CCDC NO.: 2341397)

Identification code	mo_231017B	
Chemical formula	$C_{15}H_{12}O_2$	
Formula weight	224.25 g/mol	
Wavelength	0.71073 Å	
Crystal size	0.060 x 0.100 x 0.130 mm	
Crystal system	monoclinic	
Space group	P 1 21/n 1	
Unit cell dimensions	a = 12.0381(14) Å	α = 90°
	b = 6.3338(6) Å	β = 108.132(4)°
	c = 15.2736(19) Å	γ = 90°
Volume	1106.7(2) $Å^3$	
Z	4	
Density (calculated)	1.346 g/cm^3	
Absorption coefficient	0.089 mm^{-1}	
F(000)	472	
Theta range for data collection	2.59 to 25.00°	
Index ranges	-14<=h<=14, -7<=k<=6, -18<=l<=18	
Reflections collected	6394	
Independent reflections	1953 [R(int) = 0.0775]	
Max. and min. transmission	0.7457 and 0.6849	
Structure solution technique	direct methods	
Structure solution program	SHELXT 2018/2 (Sheldrick, 2018)	
Refinement method	Full-matrix least-squares on F^2	
Refinement program	SHELXL 2018/3 (Sheldrick, 2015)	
Function minimized	$\Sigma\ w(F_o^2 - F_c^2)^2$	
Data / restraints / parameters	1953 / 0 / 155	
Goodness-of-fit on F2	1.092	
Final R indices	1372 data; I>2σ(I)	R1 = 0.0781, wR2 = 0.1855
	all data	R1 = 0.1116, wR2 = 0.2052
Weighting scheme	$w=1/[\sigma^2(F_o^2)+(0.0819P)^2+1.3542P]$	

	where $P=(F_o^2+2F_c^2)/3$	
Largest diff. peak and hole	0.741 and -0.376 eÅ^{-3}	
R.M.S. deviation from mean	0.071 eÅ^{-3}	

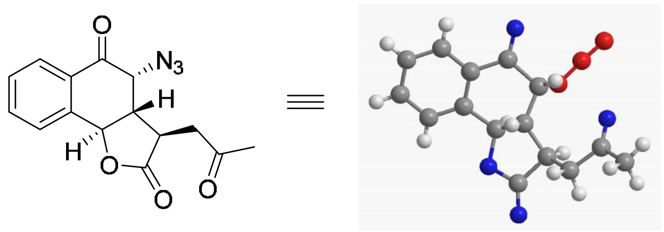

13 (CCDC NO.: 2505939)

Identification code	cu_250420A	
Chemical formula	$C_{15}H_{13}N_3O_4$	
Formula weight	299.28 g/mol	
Wavelength	1.54178 Å	
Crystal size	0.130 x 0.170 x 0.190 mm	
Crystal system	orthorhombic	
Space group	P 21 21 21	
Unit cell dimensions	a = 5.6634(5) Å	α = 90°
	b = 9.6041(8) Å	β = 90°
	c = 25.492(2) Å	γ = 90°
Volume	1106.7(2) Å^3	
Z	4	
Density (calculated)	1386.6(2) Å^3	
Absorption coefficient	0.089 mm^{-1}	
F(000)	624	
Theta range for data collection	4.92 to 77.96°	
Index ranges	-7<=h<=4, -12<=k<=12, -32<=l<=31	
Reflections collected	22175	
Independent reflections	2925 [R(int) = 0.0391]	
Max. and min. transmission	0.7541 and 0.6609	
Structure solution technique	direct methods	
Structure solution program	SHELXT 2018/2 (Sheldrick, 2018)	
Refinement method	Full-matrix least-squares on F^2	
Refinement program	SHELXL 2018/3 (Sheldrick, 2015)	
Function minimized	$\Sigma w(F_o^2 - F_c^2)^2$	
Data / restraints / parameters	1925 / 0 / 200	

Goodness-of-fit on F2	1.059	
Final R indices	2909 data; I>2σ(I)	R1 = 0.0291, wR2 = 0.0807
	all data	R1 = 0.0292, wR2 = 0.0808
Weighting scheme	$w=1/[\sigma^2(F_o^2)+(0.0542P)^2+0.2148P]$ where $P=(F_o^2+2F_c^2)/3$	
Absolute structure parameter	-0.04(5)	
Largest diff. peak and hole	0.161 and -0.187 eÅ^{-3}	
R.M.S. deviation from mean	0.041 eÅ^{-3}	

8. References

1. L. Zhang, C. M. Le and M. Lautens, *Angew. Chem. Int. Ed.*, 2014, **53**, 5951-5954.

2. S. Li, Q. Chen, J. Yang and J. Zhang, *Angew. Chem. Int. Ed.*, 2022, **61**, e202202046.According to Zhang's report, the absolute configuration and structure of **5a** was assigned to be *R*.

3. M. J. Frisch, G. W. Trucks, H. B. Schlegel, G. E. Scuseria, M. A. Robb, J. R. Cheeseman, G. Scalmani, V. Barone, G. A. Petersson, H. Nakatsuji, X. Li, M. Caricato, A. V. Marenich, J. Bloino, B. G. Janesko, R. Gomperts, B. Mennucci, H. P. Hratchian, J. V. Ortiz, A. F. Izmaylov, J. L. Sonnenberg, Williams, F. Ding, F. Lipparini, F. Egidi, J. Goings, B. Peng, A. Petrone, T. Henderson, D. Ranasinghe, V. G. Zakrzewski, J. Gao, N. Rega, G. Zheng, W. Liang, M. Hada, M. Ehara, K. Toyota, R. Fukuda, J. Hasegawa, M. Ishida, T. Nakajima, Y. Honda, O. Kitao, H. Nakai, T. Vreven, K. Throssell, J. A. Montgomery Jr., J. E. Peralta, F. Ogliaro, M. J. Bearpark, J. J. Heyd, E. N. Brothers, K. N. Kudin, V. N. Staroverov, T. A. Keith, R. Kobayashi, J. Normand, K. Raghavachari, A. P. Rendell, J. C. Burant, S. S. Iyengar, J. Tomasi, M. Cossi, J. M. Millam, M. Klene, C. Adamo, R. Cammi, J. W. Ochterski, R. L. Martin, K. Morokuma, O. Farkas, J. B. Foresman and D. J. Fox, Gaussian 16 Rev. A.03, Wallingford, CT, 2016.

4. A. D. Becke, *J. Chem. Phys.*, 1993, **98**, 5648-5652.

5. S. Grimme, J. Antony, S. Ehrlich and H. Krieg, *J. Chem. Phys.*, 2010, **132**, 154104.

6. F. Weigend and R. Ahlrichs, *Phys. Chem. Chem. Phys.*, 2005, **7**, 3297-3305.

7. K. Fukui, *Acc. Chem. Res.*, 1981, **14**, 363-368.

8. D. Andrae, U. Häußermann, M. Dolg, H. Stoll and H. Preuß, *Theor. Chim. Acta*, 1990, **77**, 123-141.

9. A. V. Marenich, C. J. Cramer and D. G. Truhlar, *J. Phys. Chem. B*, 2009, **113**, 6378-6396.

10. G. Scalmani and M. J. Frisch, J. Chem. Phys., 2010, 132, 114110.

11. T. D. Tran, N. B. Pham, R. Booth, P. I. Forster and R. J. Quinn, *J. Nat. Prod.*, 2016, **79**, 1514-1523.

12. S. Song, X. Huang, M. Bai, G. Yao, H. Chang and J. Li, CN Patent 115894414 A, 2023.

13. P. C. Eklund, S. M. Willfor, A. I. Smeds, F. J. Sundell, R. E. Sjoholm and B. R. Holmbom, *J. Nat. Prod.*, 2004, **67**, 927-931.

14. P. A. Onocha and M. S. Ali, *Res. J. Phytochem.*, 2011, **5**, 136-145.

15. S.-J. Xiao, D.-L. Guo, B. Xia, S. Allen, Y.-C. Gu, F. Chen, L.-S. Ding and Y. Zhou, *Planta Med.*, 2016, **82**, 723-728.

16. K. H. Kim, K. W. Woo, E. Moon, S. U. Choi, S. Y. Kim, S. Z. Choi, M. W. Son and K. R. Lee, *J. Agric. Food Chem.*, 2014, **62**, 7746-7752.

17. S. Mitra, S. R. Gurrala and R. S. Coleman, *J. Org. Chem.*, 2007, **72**, 8724-8736.

18. P. V. Ramachandran and M. Seleem, WO Patent 2023/150719 A1, 2023.

19. R. Bandichhor, B. Nosse and O. Reiser, In Natural Product Synthesis I; Topics in Current Chemistry; Springer-Verlag: Berlin, Heidelberg, 2005; Vol. 243, pp 43-72.

20. T. Matsumoto, Y. Takeda, M. Oiwamoto, K. Fujii, M. Kishida, H. Shibutani and S. Imai, *Chem. Pharm. Bull.*, 1995, **43**, 2099-2104.

21. W. Yu, Y. Mei, Y. Kang, Z. Hua and Z. Jin, *Org. Lett.*, 2004, **6**, 3217-3219.

22. K. C. Nicolaou, V. A. Adsool and C. R. H. Hale, *Org. Lett.*, 2010, **12**, 1552-1555.

23. Z. Cheng, K. Huang, C. Wang, L. Chen, X. Li, Z. Hu, X. Shan, P.-F. Cao, H. Sun, W. Chen, C. Li, Z. Zhang, H. Tan, X. Jiang, G. Zhang, Z. Zhang, M. Lin, L. Wang, A. Zheng, C. Xia, T. Wang, S. Song, X. Shu and N. Jiao, *Science*, 2025, **387**, 1083-1090.

24. H. A. McManus, M. J. Fleming and M. Lautens, *Angew. Chem. Int. Ed.*, 2007, **46**, 433-436.

25. P. Honerjager, A. Heiss, M. Schafer-Korting, G. Schonsteiner and M. Reiter, *Naunyn-Schmiedeberg's Arch. Pharmacol.*, 1984, **325**, 259-269.

26. A. F. E. Rump, D. A. R. Rosen and W. Klaus, *Pharmacol. Toxicol.*, 1994, **74**, 244-248.

27. R. Jonas, M. Klockow, I. Lues, H. Prucher, H. J. Schliep and H. Wurziger, *Eur. J. Med. Chem.*, 1993, **28**, 129-140.

28. D. W. Combs, M. S. Rampulla, S. C. Bell, D. H. Klaubert, A. J. Tobia, R. Falotico, B. Haertlein, C. Lakas-Weiss and J. B. Moore, *J. Med. Chem.*, 1990, **33**, 380-386.

29. H. Xu, M. Zhan, B. Liu and H. Yang, Chin. *J. Org. Chem*., 2014, **34**, 722-728.
30. L. Zu, B. W. Boal and N. K. Garg, *J. Am. Chem. Soc*., 2011, **133**, 8877-8879.
31. M. E. Hattab, G. Genta-Jouve, N. Bouzidi, A. Ortalo-Magne, C. Hellio, J.-P. Marechal, L. Piovetti, O. P. Thomas and G. Culioli, *J. Nat. Prod*., 2015, **78**, 1663-1670.
32. L. Larsen, M. H. Benn, M. Parvez and N. B. Perry, *Org. Biomol. Chem*., 2005, **3**, 3236-3241.
33. D. Yang, H. Xie, Y. Jiang and X. Wei, *Food Chem*., 2016, **194**, 857-863.
34. D. M. Cárdenas, F. J. R. Mejías, J. M. G. Molinillo and F. A. Macías, *J. Org. Chem*., 2020, **85**, 7322-7332.
35. M. Xue, J. Cui, X. Zhu, F. Wang, D. Lv, Z. Nie, Y. Li and H. Bao, *Angew. Chem. Int. Ed*., 2023, **62**, e202304275.
36 .Y. Li, X. Li and J.-P. Cheng, *Adv. Synth. Catal*., 2014, **356**, 1172-1198.
37. J. Chen, L. Su, T. Li, W. Ma, Y. Ma, C. Geng, W. Dong, X. He and X. Zhang, CN Patent 116589474 A, 2023.
38. N. Ohno, T. J. Mabry, V. Zabelt and W. H. Waston, *Phytochemistry*, 1979, **18**, 1687-1689.
39. Y.-K. Kim, K.-H. Son, J.-Y. Nam, S.-U. Kim, T.-S. Jeong, W.-S. Lee, S.-H. Bok, B.-M. Kwon, Y. J. Park and J. M. Shin, J. *Antibiot*., 1996, **49**, 815-816.
40. Z. Xu, L. Wu, C. Xie, Y. Liu, X. Wen, Z. Gu and J. Peng, CN Patent 115991690 A, 2023.
41. J. Wellsow, R. J. Grayer, N. C. Veitch, T. Kokubun, R. Lelli, G. C. Kite and M. S. J. Simmonds, *Phytochemistry*, 2006, **67**, 1818-1825.
42. Y. Yamashita, Y. Hirano, A. Takada, H. Takikawa and K. Suzuki, *Angew. Chem. Int. Ed*., 2013, **52**, 6658-6661.
43. X.-F. Wu, Y.-C. Hu, S. Gao, S.-S. Yu, Y.-H. Pei, W.-Z. Tang and X.-Z. Huang, *J. Asian Nat. Prod. Res.*, 2007, **9**, 471-477.
44. S. Chandrasekhar, D. Basu, M. Sailu and S. Kotamraju, *Tetrahedron Lett*., 2009, **50**, 4882-4884.
45. J. Liu, J. Flegel, F. Otte, A. Pahl, S. Sievers, C. Strohmann and H. Waldmann, *Angew. Chem. Int. Ed*., 2021, **60**, 21384-21395.

46. Y.-H. Wen, F. Yang, S. Li, X. Yao, J. Song and L.-Z. Gong, *J. Am. Chem. Soc*., 2023, **145**, 4199-4207.

9. NMR spectra

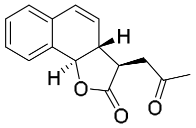

(*R*,*R*,*R*)-**3a**

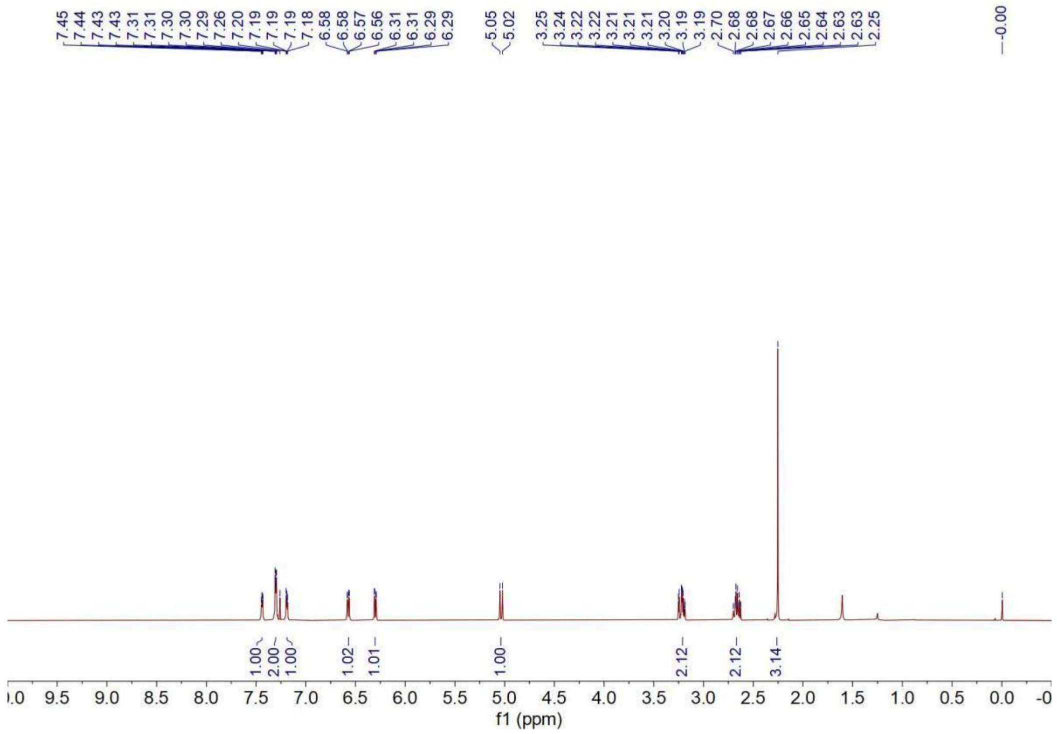

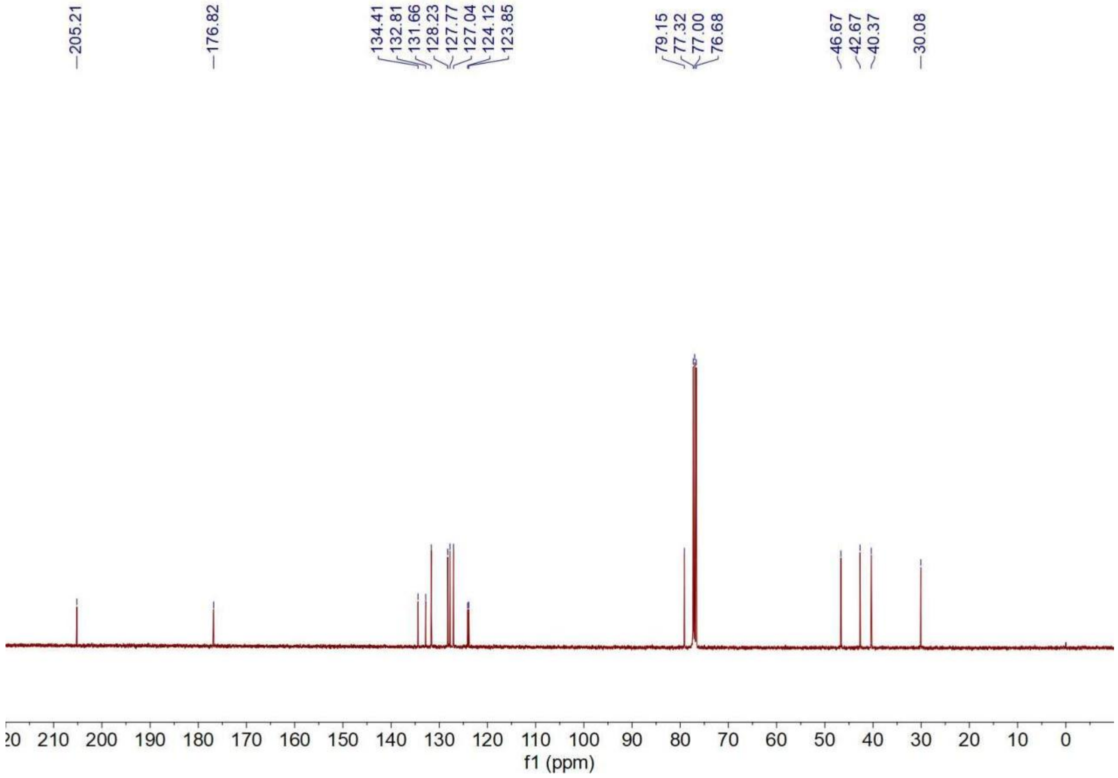

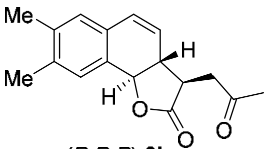
Me
Me
H
H
O
O
O

(*R*,*R*,*R*)-**3b**

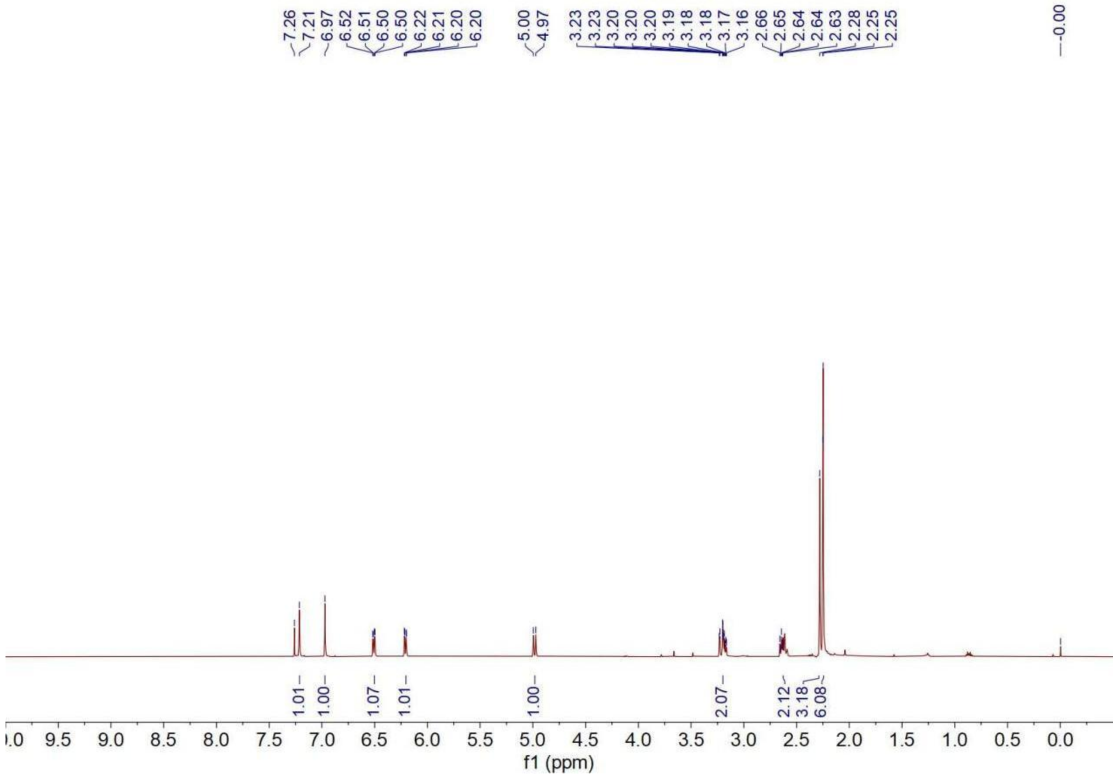
7.26
7.21
6.97
6.52
6.51
6.50
6.50
6.22
6.21
6.20
6.20
5.00
4.97
3.23
3.23
3.20
3.20
3.20
3.19
3.18
3.18
3.17
3.16
2.66
2.65
2.64
2.64
2.63
2.28
2.25
2.25
-0.00
1.01
1.00
1.07
1.01
1.00
2.07
2.12
3.18
6.08
9.5 9.0 8.5 8.0 7.5 7.0 6.5 6.0 5.5 5.0 4.5 4.0 3.5 3.0 2.5 2.0 1.5 1.0 0.5 0.0
f1 (ppm)

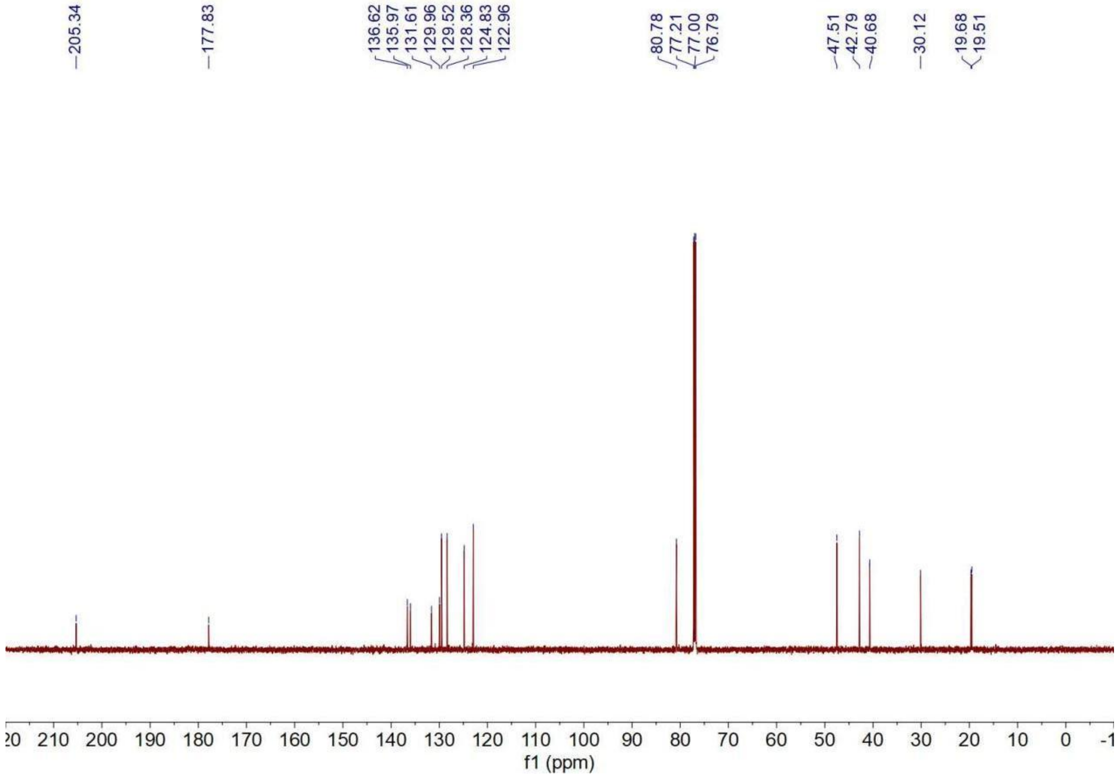
205.34
177.83
136.62
135.97
131.61
129.96
129.52
128.36
124.83
122.96
80.78
77.21
77.00
76.79
47.51
42.79
40.68
30.12
19.68
19.51
210 200 190 180 170 160 150 140 130 120 110 100 90 80 70 60 50 40 30 20 10 0
f1 (ppm)

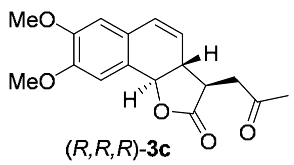
MeO
MeO
H
H
O
O
O

(*R*,*R*,*R*)-**3c**

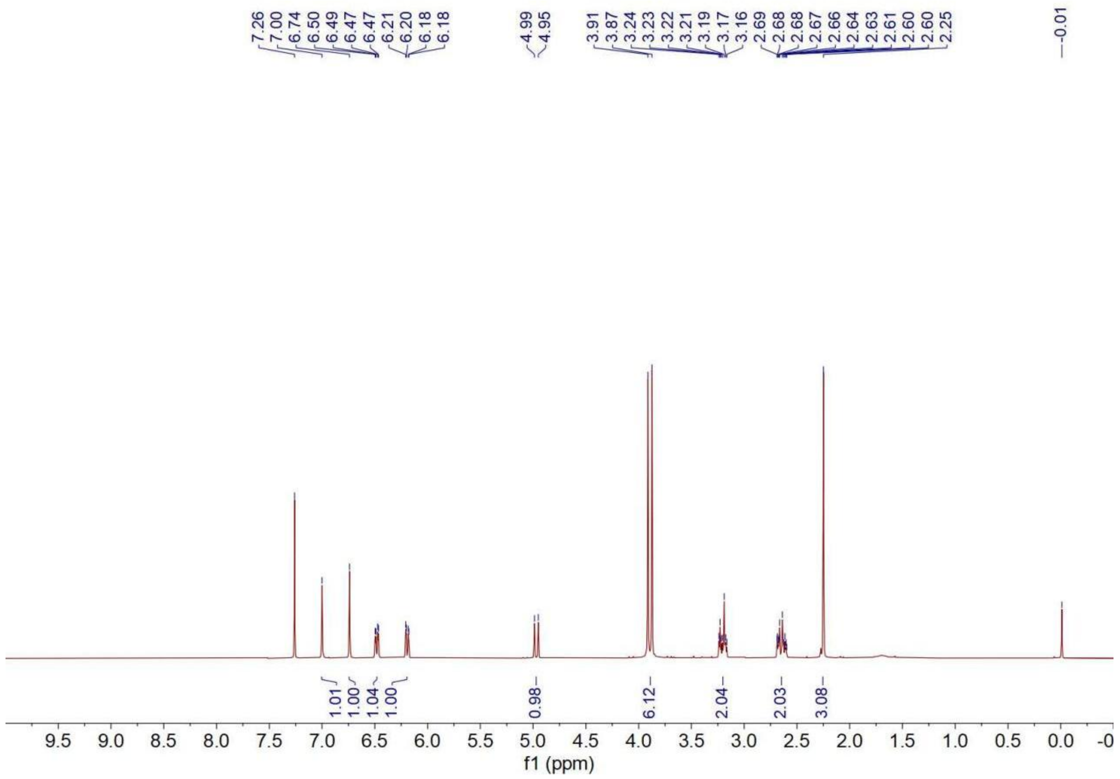
7.26
7.00
6.74
6.50
6.49
6.47
6.47
6.21
6.20
6.18
6.18
4.99
4.95
3.91
3.87
3.24
3.23
3.22
3.21
3.19
3.17
3.16
2.69
2.68
2.68
2.67
2.66
2.64
2.63
2.61
2.60
2.60
2.25
-0.01
1.01
1.00
1.04
1.00
0.98
6.12
2.04
2.03
3.08
9.5 9.0 8.5 8.0 7.5 7.0 6.5 6.0 5.5 5.0 4.5 4.0 3.5 3.0 2.5 2.0 1.5 1.0 0.5 0.0 -0
f1 (ppm)

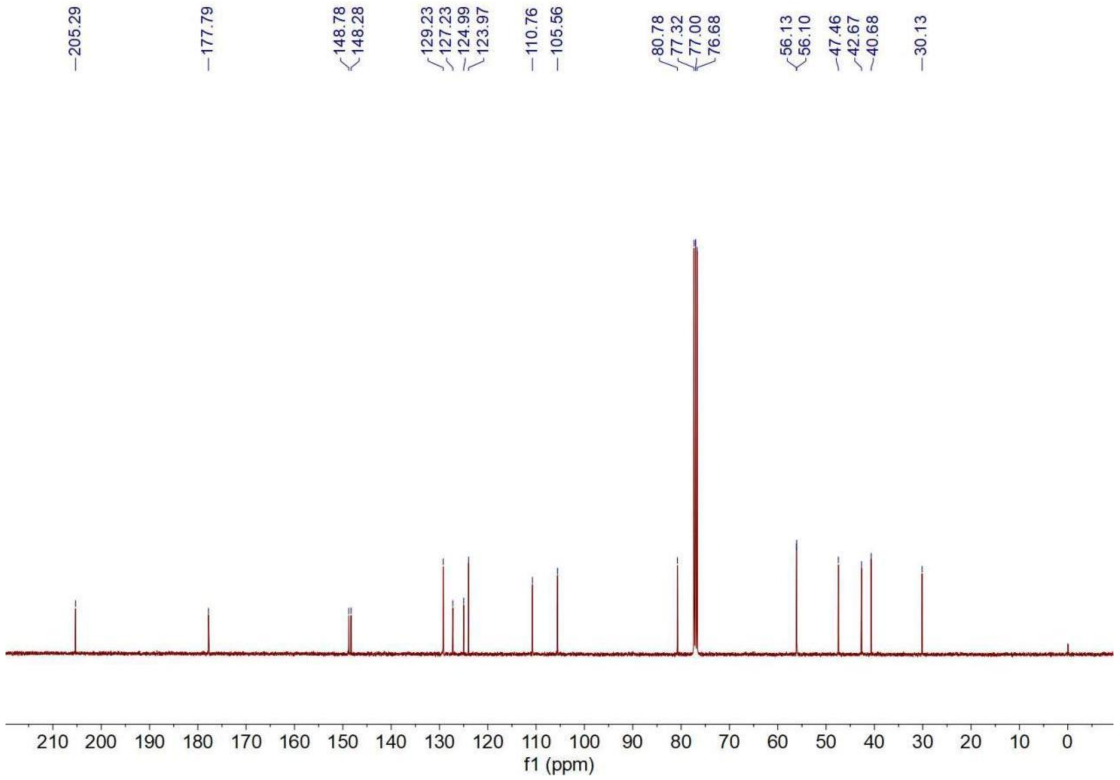
205.29
177.79
148.78
148.28
129.23
127.23
124.99
123.97
110.76
105.56
80.78
77.32
77.00
76.68
56.13
56.10
47.46
42.67
40.68
30.13
210 200 190 180 170 160 150 140 130 120 110 100 90 80 70 60 50 40 30 20 10 0
f1 (ppm)

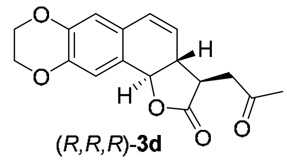
O
O
H
H
O
O
O

(*R*,*R*,*R*)-**3d**

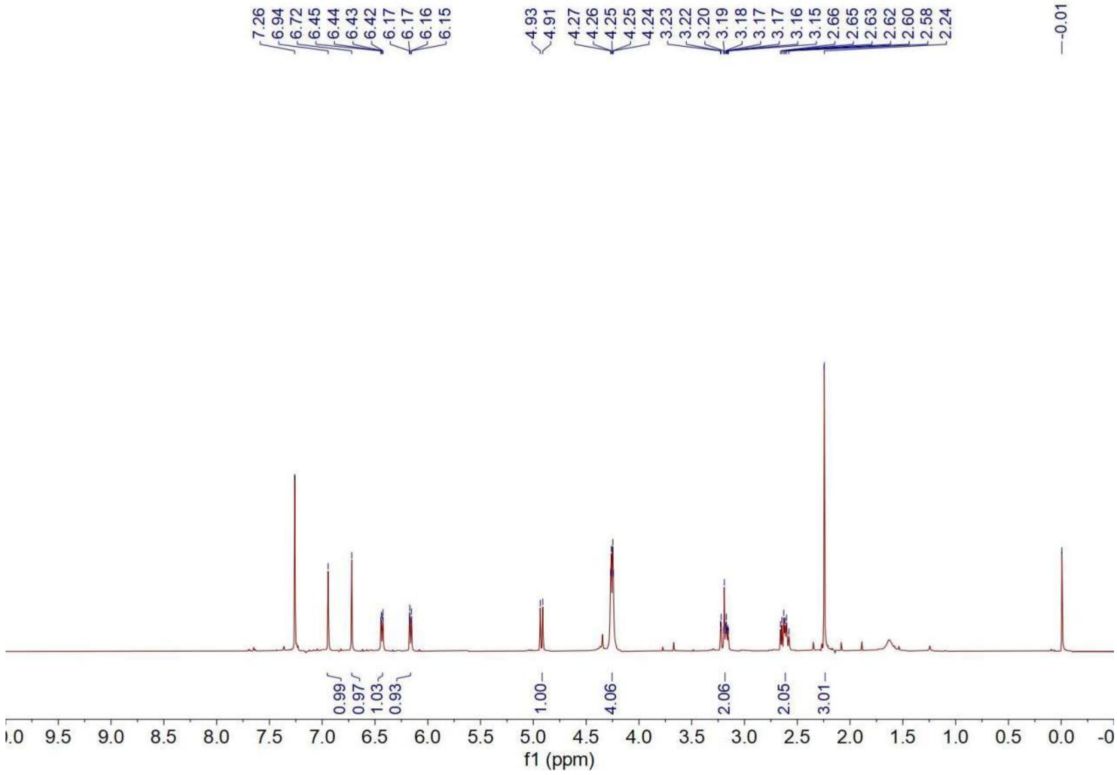
7.26
6.94
6.72
6.45
6.44
6.43
6.42
6.17
6.17
6.16
6.15
4.93
4.91
4.27
4.26
4.25
4.25
4.24
3.23
3.22
3.20
3.19
3.18
3.17
3.17
3.16
3.15
2.66
2.65
2.63
2.62
2.60
2.58
2.24
-0.01
0.99
0.97
1.03
0.93
1.00
4.06
2.06
2.05
3.01
f1 (ppm)

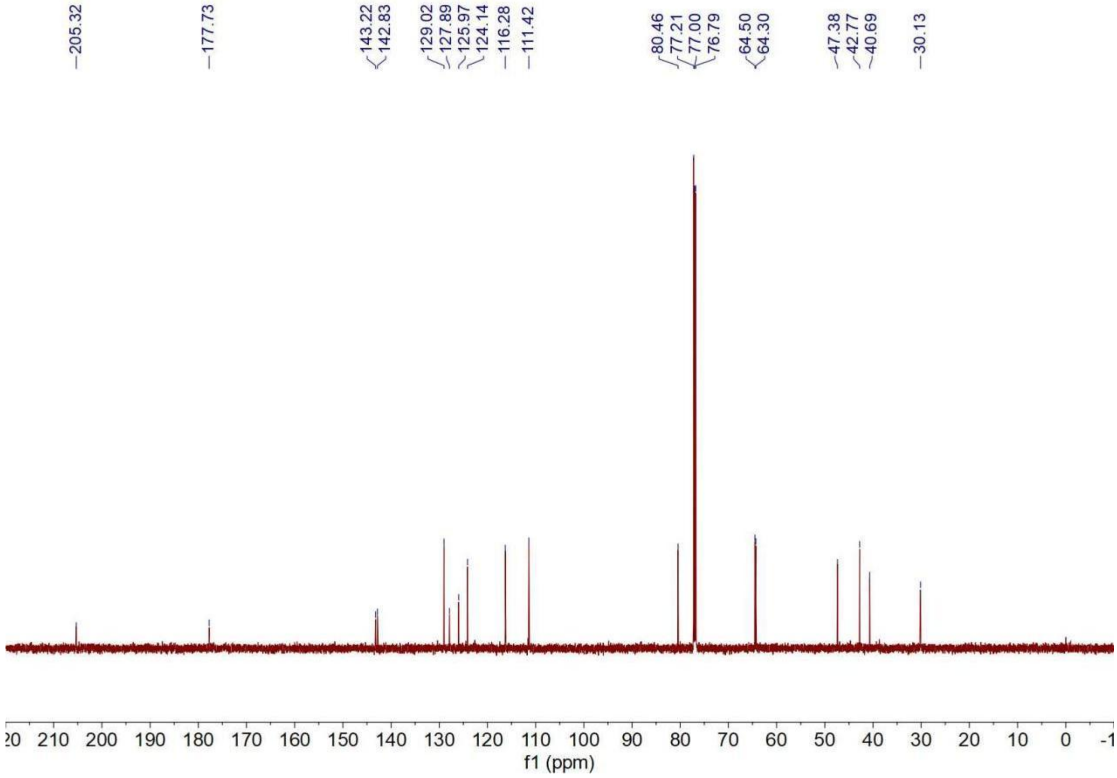
205.32
177.73
143.22
142.83
129.02
127.89
125.97
124.14
116.28
111.42
80.46
77.21
77.00
76.79
64.50
64.30
47.38
42.77
40.69
30.13
f1 (ppm)

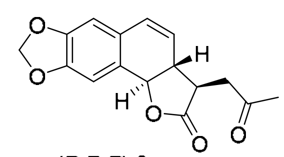

(*R*,*R*,*R*)-**3e**

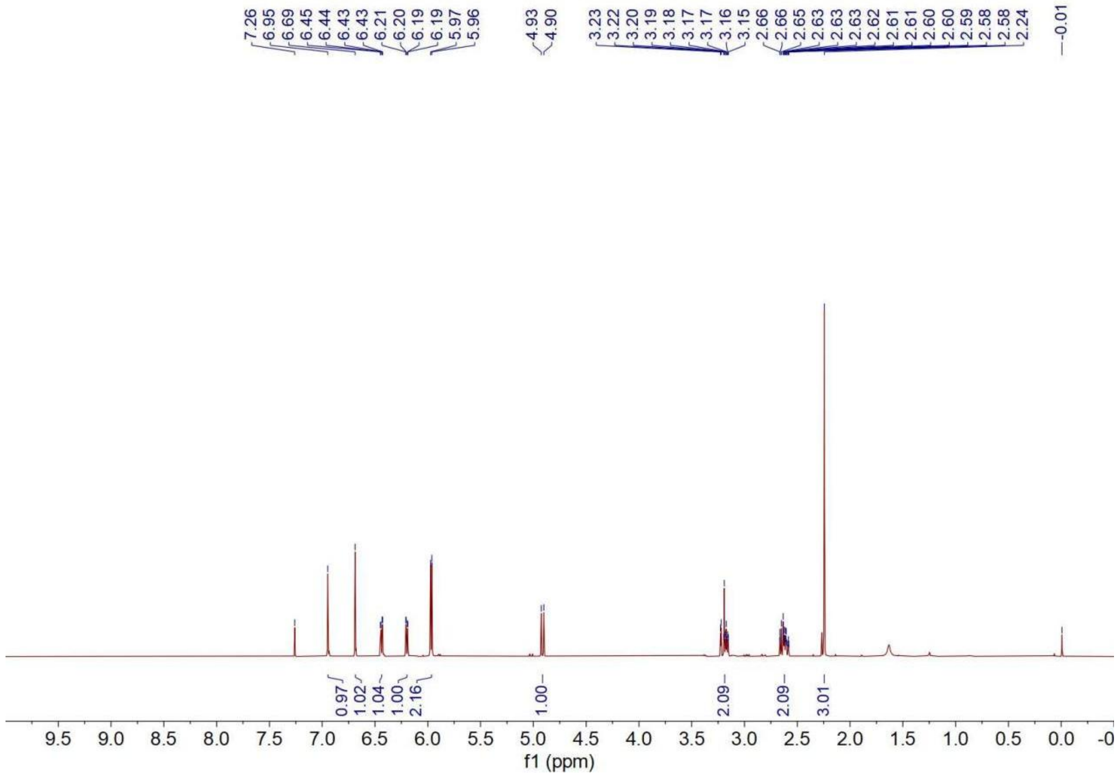
7.26
6.95
6.69
6.45
6.44
6.43
6.43
6.21
6.20
6.19
6.19
5.97
5.96
4.93
4.90
3.23
3.22
3.20
3.19
3.18
3.17
3.17
3.16
3.15
2.66
2.66
2.65
2.63
2.63
2.63
2.62
2.61
2.61
2.60
2.60
2.59
2.58
2.58
2.24
-0.01
0.97
1.02
1.04
1.00
2.16
1.00
2.09
2.09
3.01
9.5 9.0 8.5 8.0 7.5 7.0 6.5 6.0 5.5 5.0 4.5 4.0 3.5 3.0 2.5 2.0 1.5 1.0 0.5 0.0 -0
f1 (ppm)

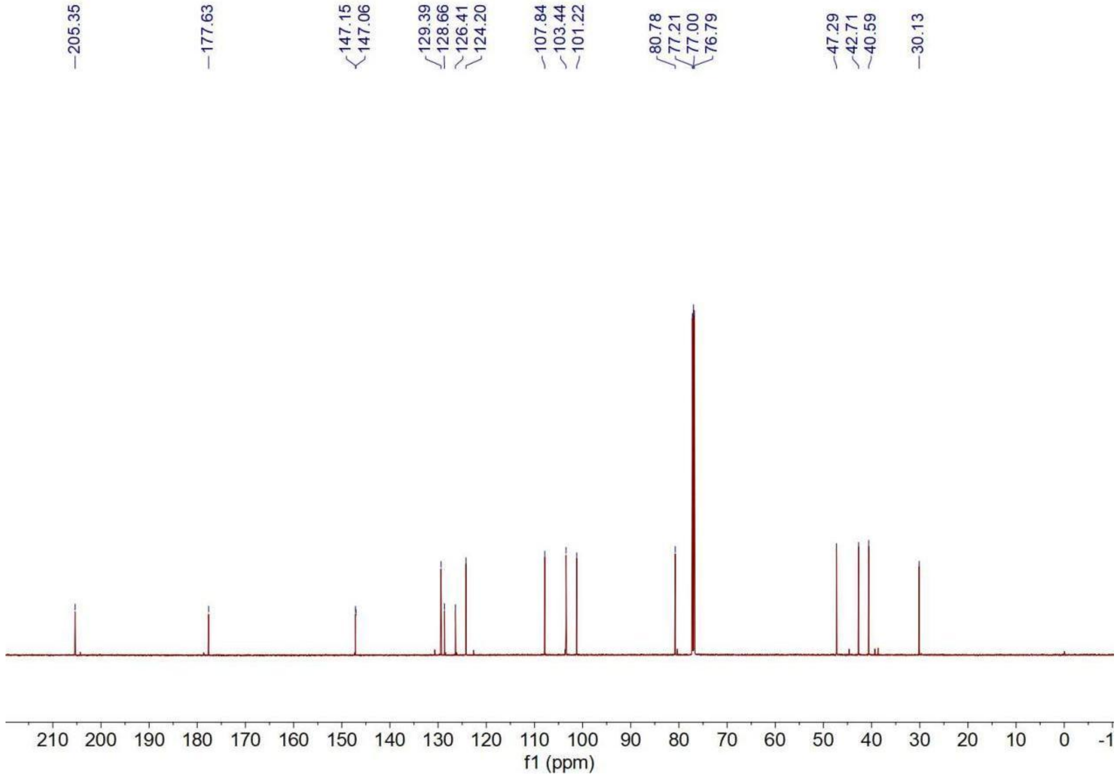
205.35
177.63
147.15
147.06
129.39
128.66
126.41
124.20
107.84
103.44
101.22
80.78
77.21
77.00
76.79
47.29
42.71
40.59
30.13
210 200 190 180 170 160 150 140 130 120 110 100 90 80 70 60 50 40 30 20 10 0 -1
f1 (ppm)

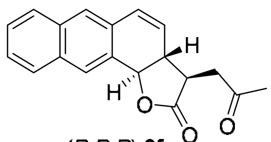
H
H
O
O
O

(*R*,*R*,*R*)-**3f**

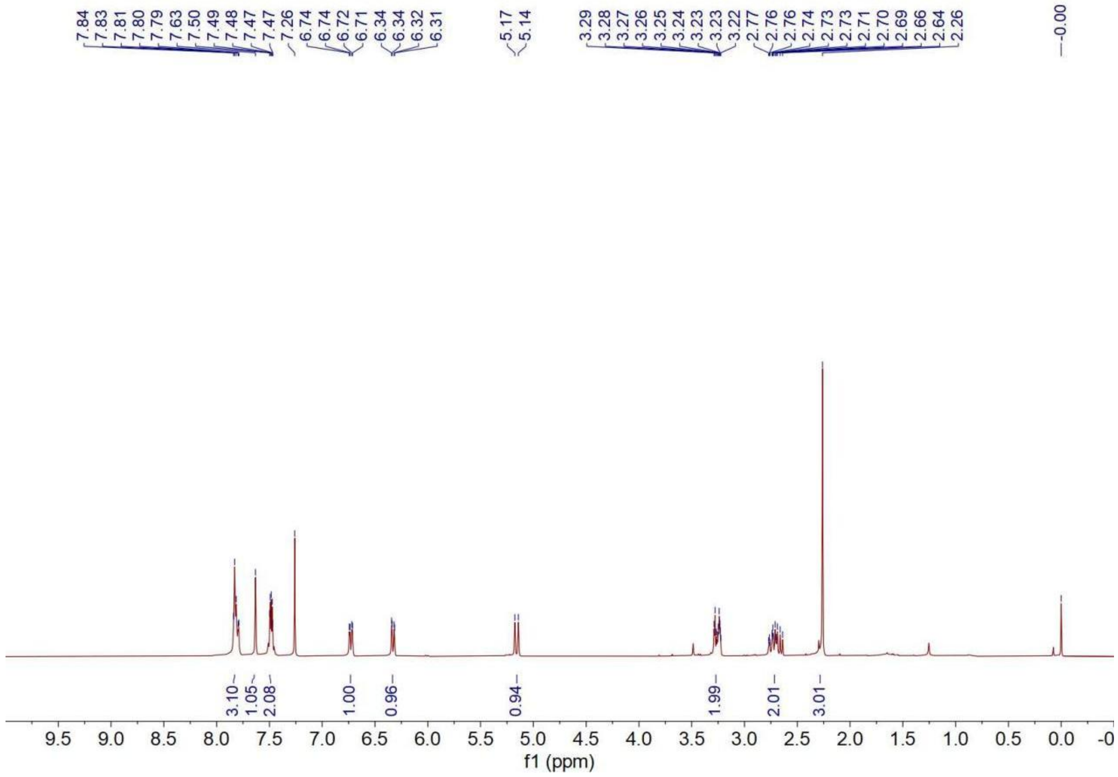
7.84
7.83
7.81
7.80
7.79
7.63
7.50
7.49
7.48
7.47
7.47
7.26
6.74
6.74
6.72
6.71
6.34
6.34
6.32
6.31
5.17
5.14
3.29
3.28
3.27
3.26
3.25
3.24
3.23
3.23
3.22
2.77
2.76
2.76
2.74
2.73
2.73
2.71
2.70
2.69
2.66
2.64
2.26
-0.00
3.10
1.05
2.08
1.00
0.96
0.94
1.99
2.01
3.01
9.5 9.0 8.5 8.0 7.5 7.0 6.5 6.0 5.5 5.0 4.5 4.0 3.5 3.0 2.5 2.0 1.5 1.0 0.5 0.0 -0
f1 (ppm)

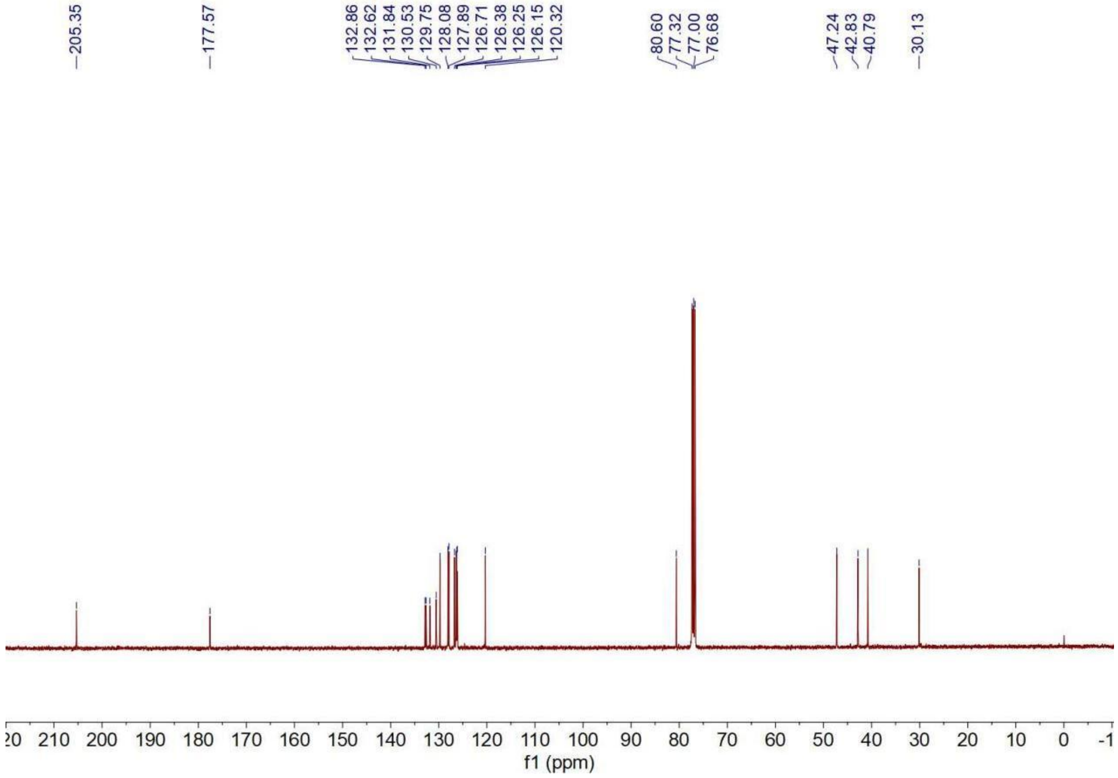
205.35
177.57
132.86
132.62
131.84
130.53
129.75
128.08
127.89
126.71
126.38
126.25
126.15
120.32
80.60
77.32
77.00
76.68
47.24
42.83
40.79
30.13
20 210 200 190 180 170 160 150 140 130 120 110 100 90 80 70 60 50 40 30 20 10 0 -1
f1 (ppm)

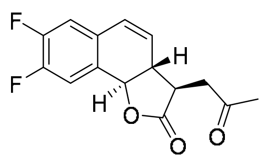
F
F
H
H
O
O
O

(*R*,*R*,*R*)-**3g**

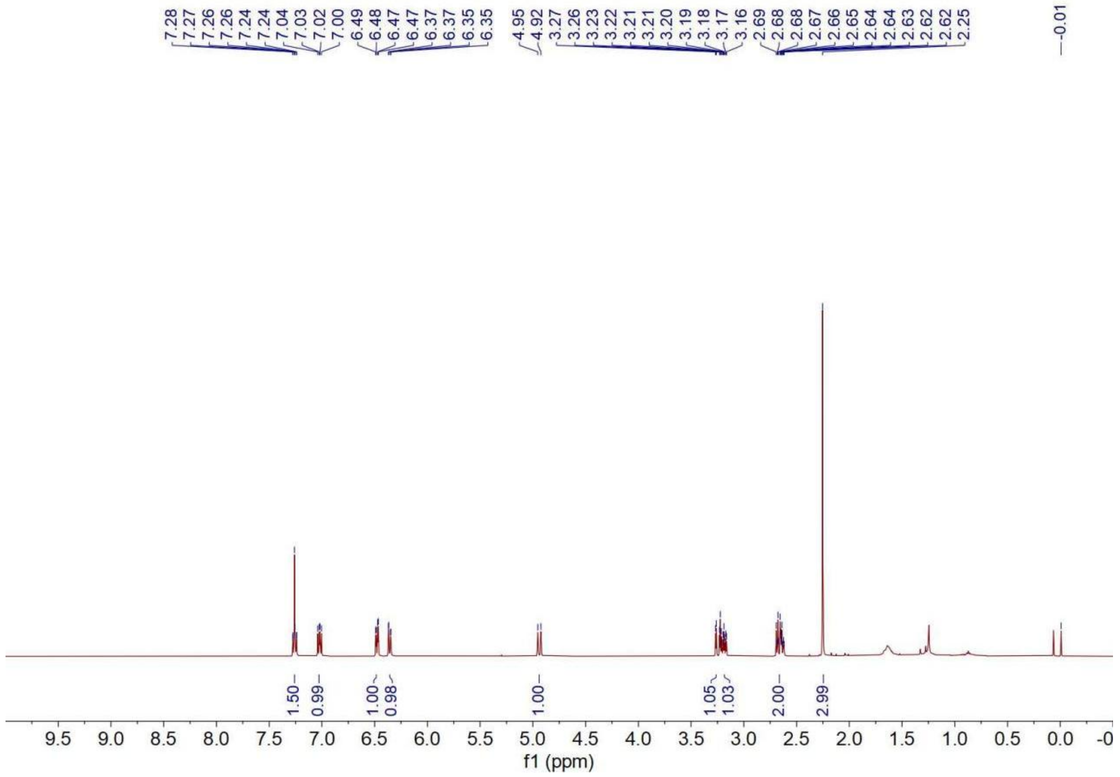
7.28
7.27
7.26
7.26
7.24
7.24
7.04
7.03
7.02
7.00
6.49
6.48
6.47
6.37
6.37
6.35
6.35
4.95
4.92
3.27
3.26
3.23
3.22
3.21
3.21
3.20
3.19
3.18
3.17
3.16
2.69
2.68
2.67
2.66
2.65
2.64
2.64
2.63
2.62
2.62
2.25
-0.01
1.50
0.99
1.00
0.98
1.00
1.05
1.03
2.00
2.99
9.5 9.0 8.5 8.0 7.5 7.0 6.5 6.0 5.5 5.0 4.5 4.0 3.5 3.0 2.5 2.0 1.5 1.0 0.5 0.0 -0
f1 (ppm)

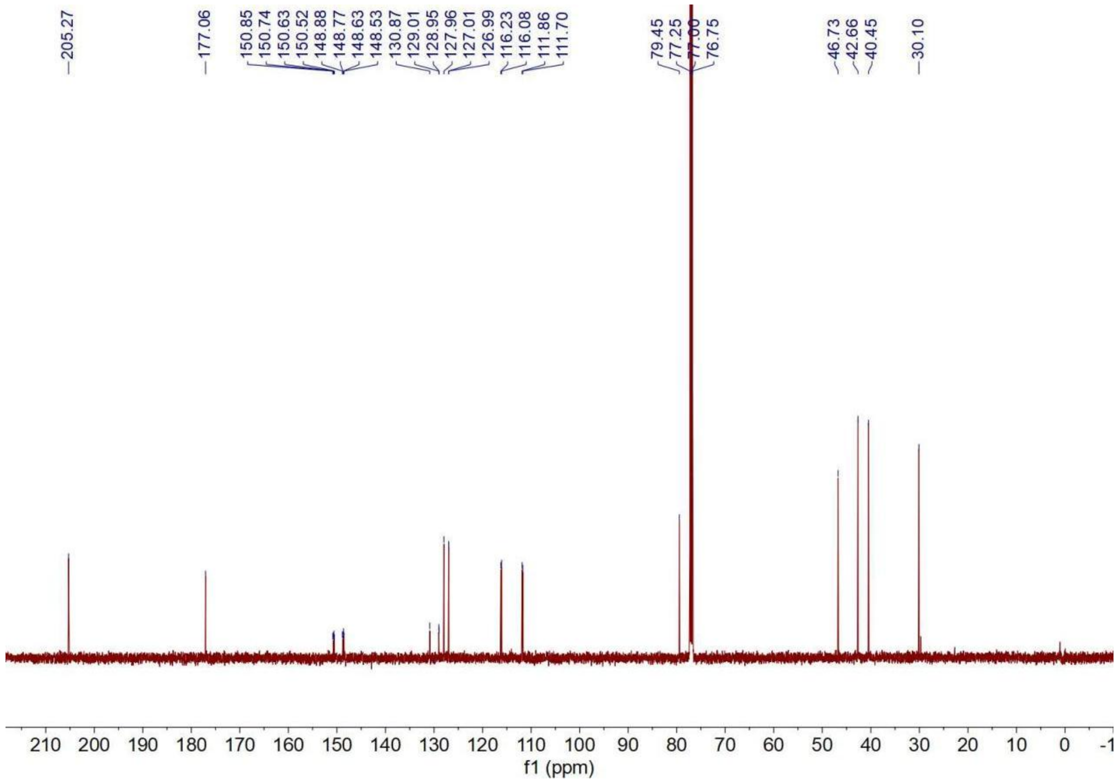
205.27
177.06
150.85
150.74
150.63
150.52
148.88
148.77
148.63
148.53
130.87
129.01
128.95
127.96
127.01
126.99
116.23
116.08
111.86
111.70
79.45
77.25
77.00
76.75
46.73
42.66
40.45
30.10
210 200 190 180 170 160 150 140 130 120 110 100 90 80 70 60 50 40 30 20 10 0 -1
f1 (ppm)

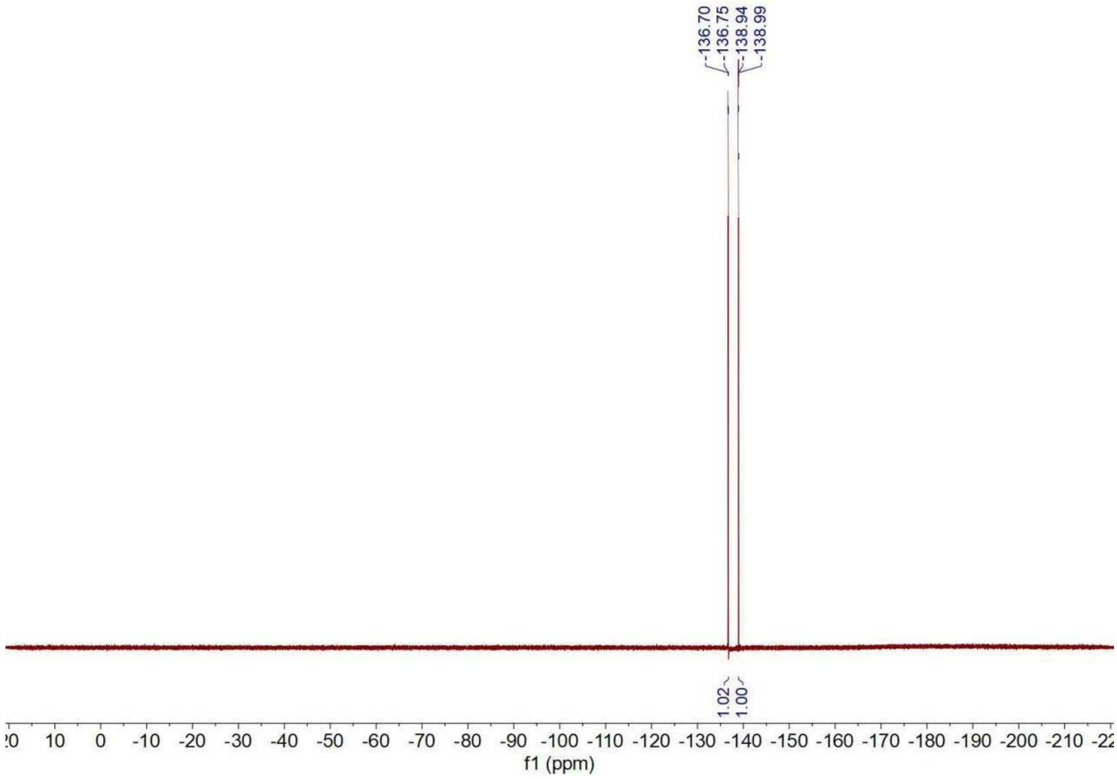
-136.70
-136.75
-138.94
-138.99
1.02
1.00
20 10 0 -10 -20 -30 -40 -50 -60 -70 -80 -90 -100 -110 -120 -130 -140 -150 -160 -170 -180 -190 -200 -210 -22
f1 (ppm)

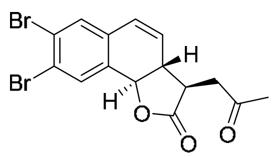
Br
Br
H
H
O
O
O

(*R*,*R*,*R*)-**3h**

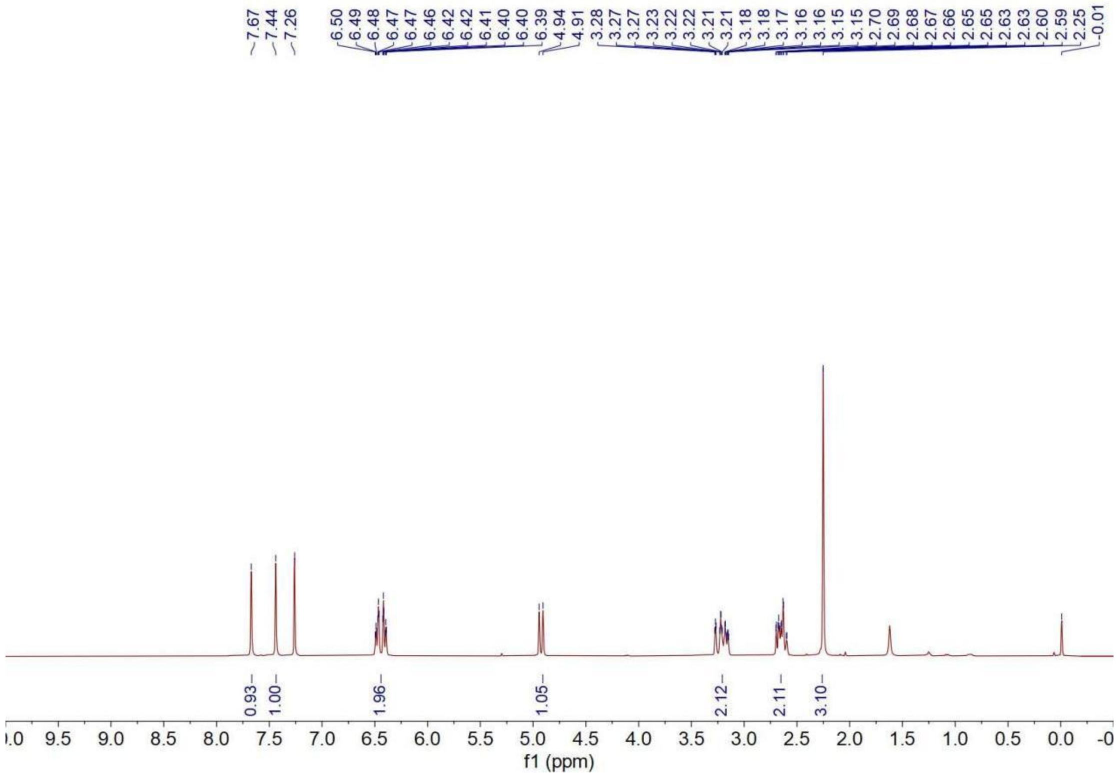
7.67
7.44
7.26
6.50
6.49
6.48
6.47
6.47
6.46
6.42
6.42
6.41
6.40
6.40
6.39
4.94
4.91
3.28
3.27
3.23
3.22
3.22
3.21
3.21
3.18
3.18
3.17
3.16
3.16
3.15
3.15
2.70
2.69
2.68
2.67
2.66
2.65
2.65
2.63
2.63
2.60
2.59
2.25
-0.01
0.93
1.00
1.96
1.05
2.12
2.11
3.10
f1 (ppm)

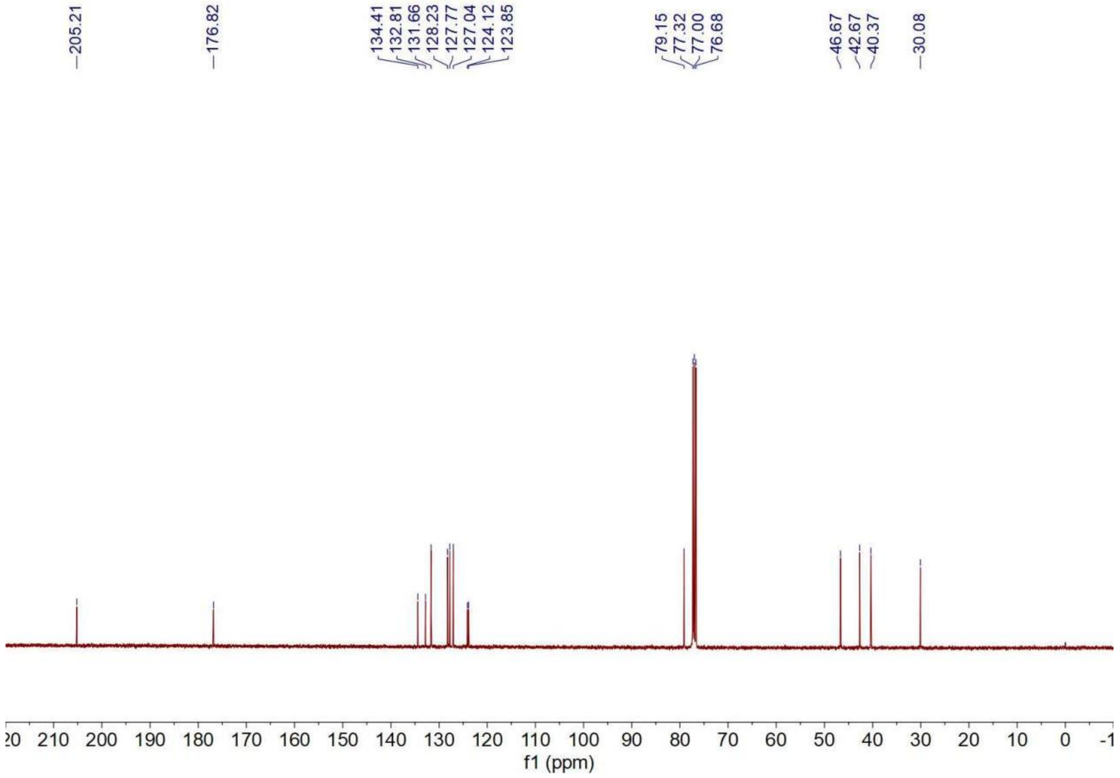
205.21
176.82
134.41
132.81
131.66
128.23
127.77
127.04
124.12
123.85
79.15
77.32
77.00
76.68
46.67
42.67
40.37
30.08
f1 (ppm)

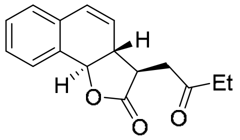
H
H
O
O
O
Et

(*R*,*R*,*R*)-**3i**

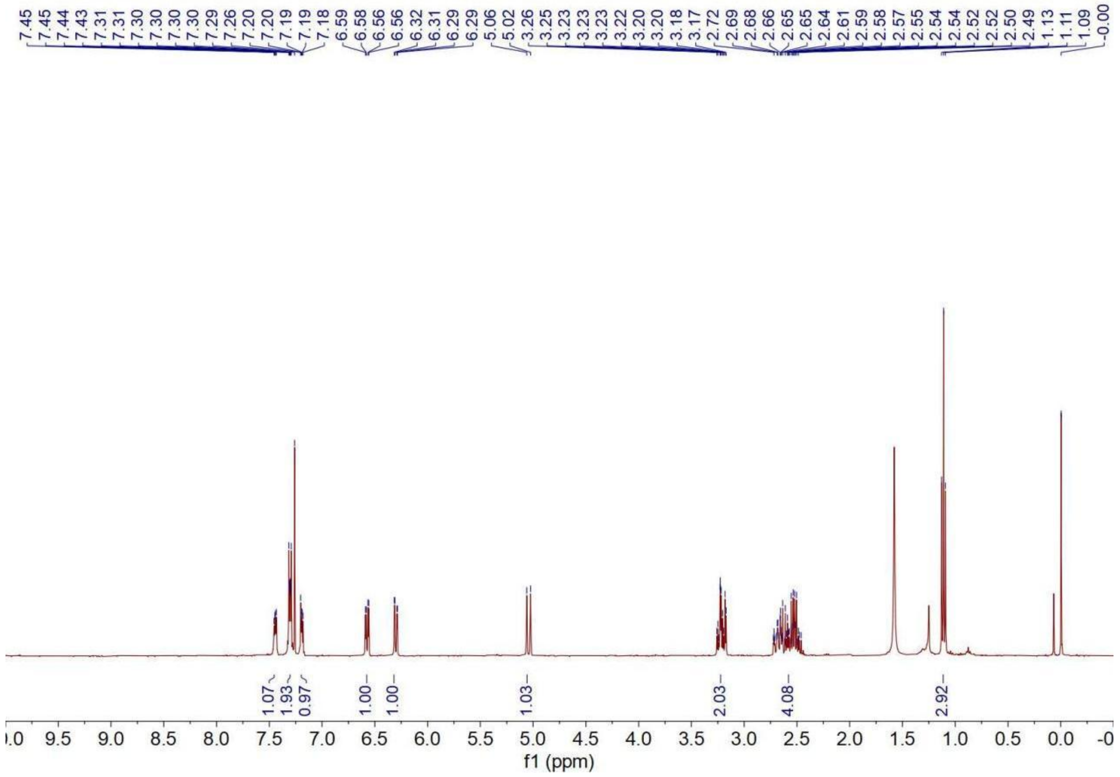
7.45
7.45
7.44
7.43
7.31
7.30
7.30
7.30
7.30
7.29
7.26
7.20
7.20
7.19
7.18
6.59
6.58
6.56
6.56
6.32
6.31
6.29
6.29
5.06
5.02
3.26
3.25
3.23
3.23
3.22
3.20
3.20
3.18
3.17
2.72
2.69
2.68
2.66
2.65
2.65
2.64
2.61
2.59
2.58
2.57
2.55
2.54
2.52
2.50
2.49
1.13
1.11
1.09
-0.00
1.07
1.93
0.97
1.00
1.00
1.03
2.03
4.08
2.92
f1 (ppm)

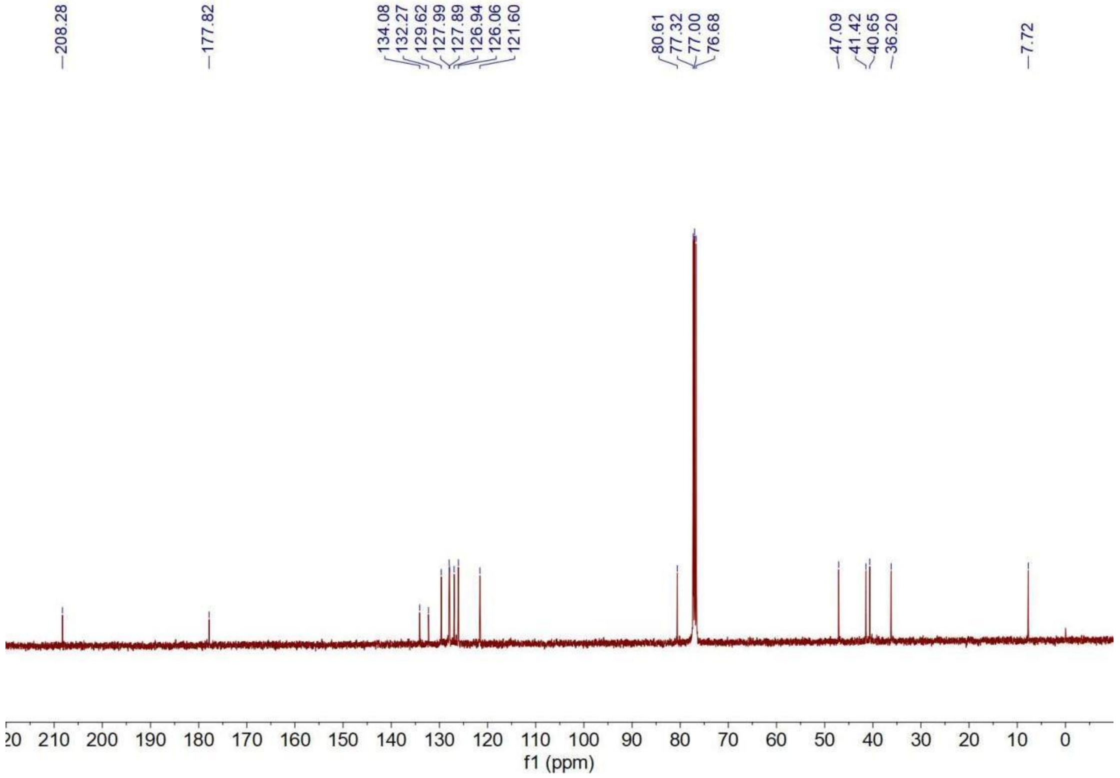
208.28
177.82
134.08
132.27
129.62
127.99
127.89
126.94
126.06
121.60
80.61
77.32
77.00
76.68
47.09
41.42
40.65
36.20
7.72
f1 (ppm)

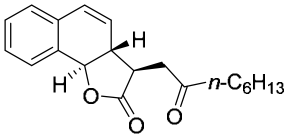
H
H
O
O
O
n-C6H13

(*R*,*R*,*R*)-**3j**

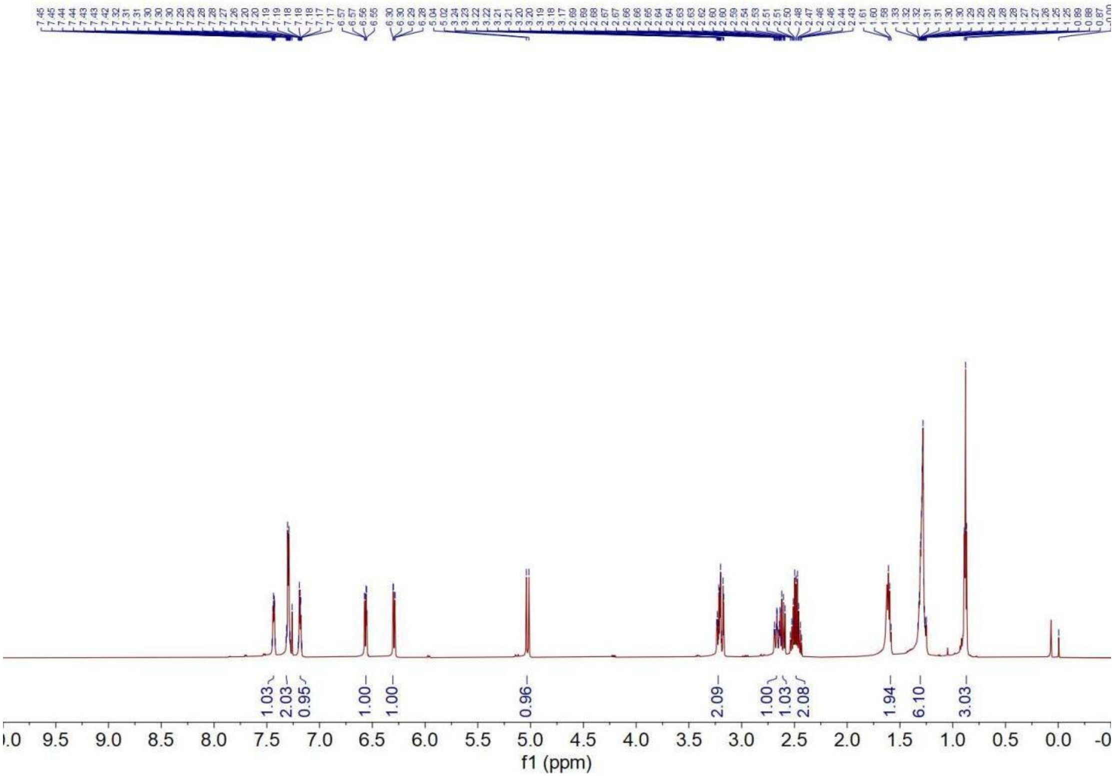
1.03
2.03
0.95
1.00
1.00
0.96
2.09
1.00
1.03
2.08
1.94
6.10
3.03
f1 (ppm)

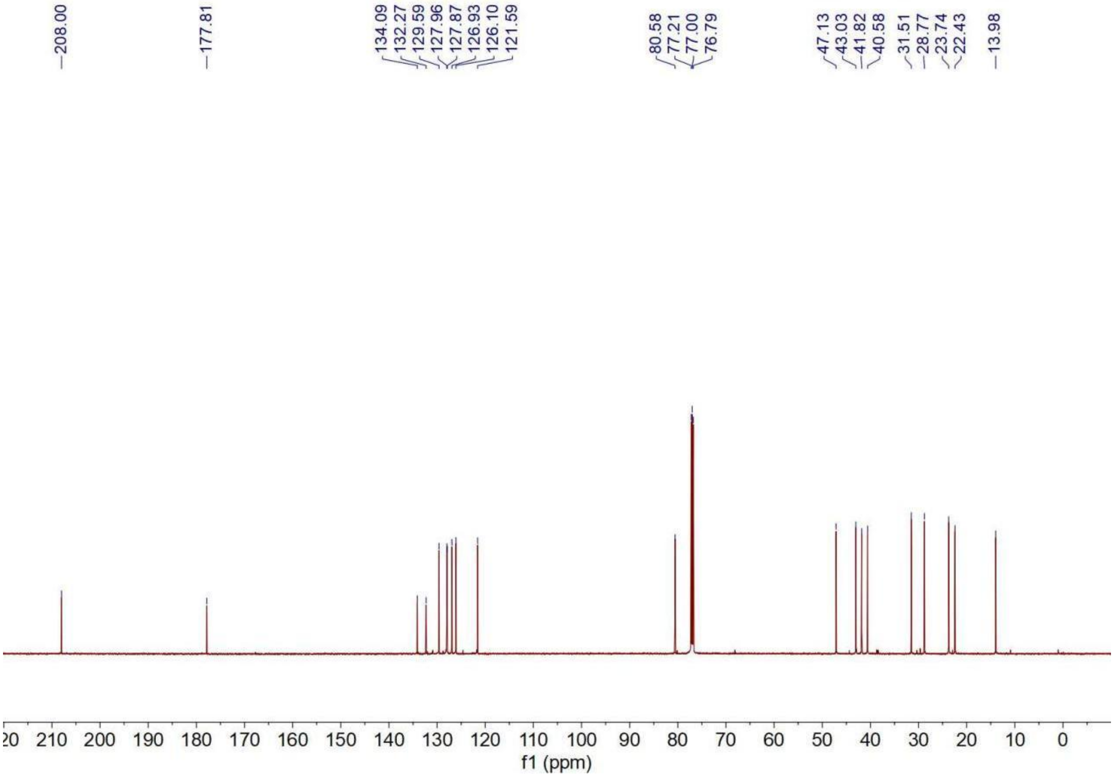
208.00
177.81
134.09
132.27
129.59
127.96
127.87
126.93
126.10
121.59
80.58
77.21
77.00
76.79
47.13
43.03
41.82
40.58
31.51
28.77
23.74
22.43
13.98
f1 (ppm)

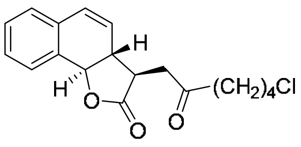
H
H
O
O
O
(CH2)4Cl

(*R*,*R*,*R*)-**3k**

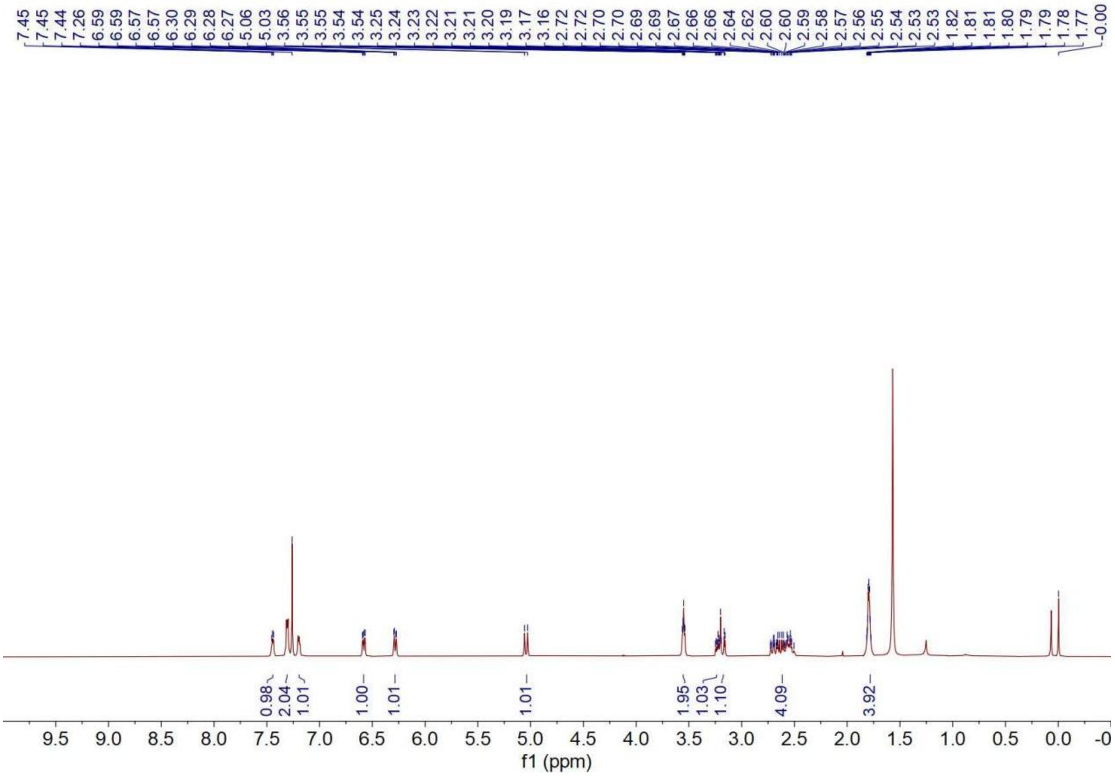
7.45
7.45
7.44
7.26
6.59
6.59
6.57
6.57
6.30
6.29
6.28
6.27
5.06
5.03
3.56
3.55
3.55
3.54
3.54
3.25
3.24
3.23
3.22
3.21
3.21
3.20
3.19
3.17
3.16
2.72
2.72
2.70
2.70
2.69
2.69
2.67
2.66
2.66
2.64
2.62
2.60
2.60
2.59
2.58
2.57
2.56
2.55
2.54
2.53
1.82
1.81
1.80
1.79
1.78
1.77
-0.00
0.98
2.04
1.01
1.00
1.01
1.01
1.95
1.03
1.10
4.09
3.92
9.5 9.0 8.5 8.0 7.5 7.0 6.5 6.0 5.5 5.0 4.5 4.0 3.5 3.0 2.5 2.0 1.5 1.0 0.5 0.0 -0
f1 (ppm)

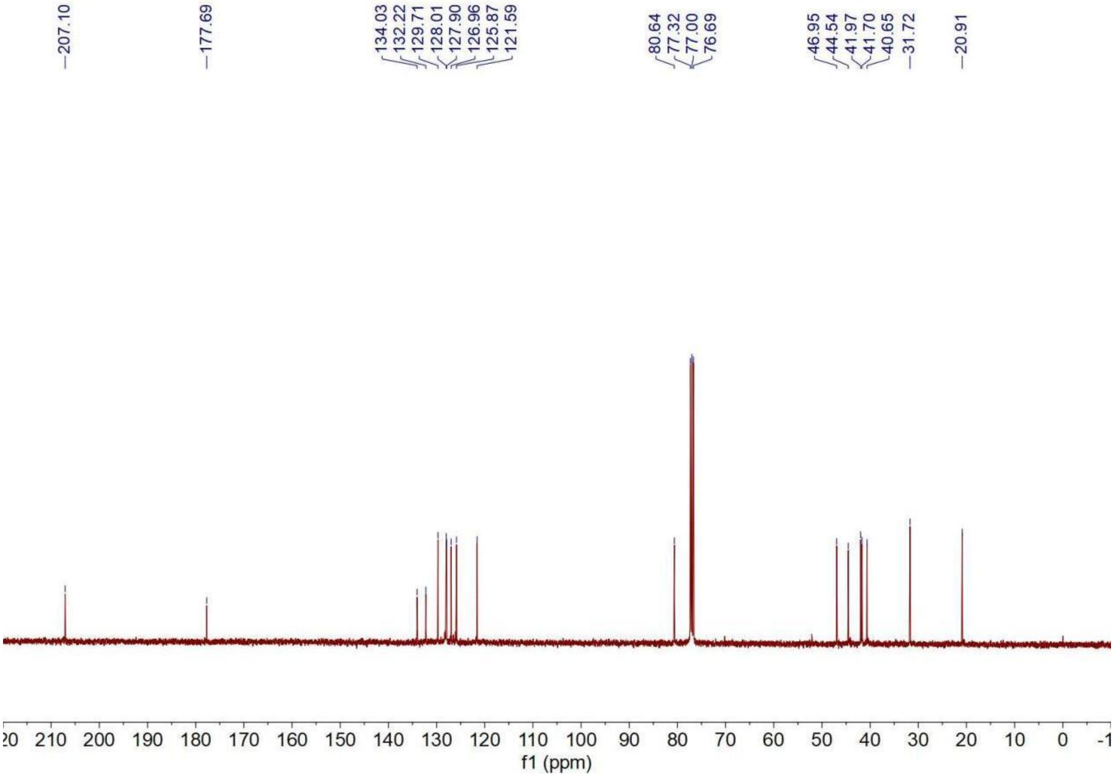
207.10
177.69
134.03
132.22
129.71
128.01
127.90
126.96
125.87
121.59
80.64
77.32
77.00
76.69
46.95
44.54
41.97
41.70
40.65
31.72
20.91
20 210 200 190 180 170 160 150 140 130 120 110 100 90 80 70 60 50 40 30 20 10 0 -1
f1 (ppm)

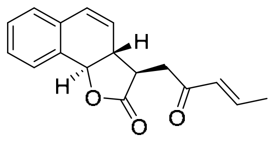

(*R*,*R*,*R*)-**3l**

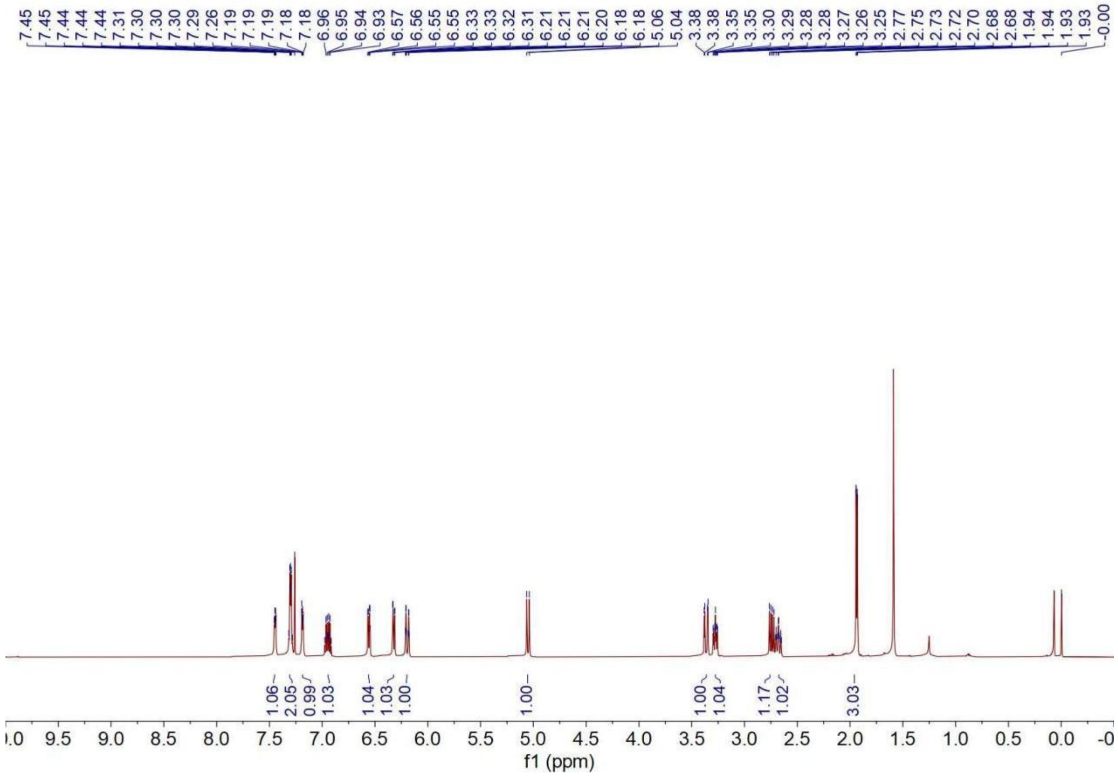
7.45 7.45 7.44 7.44 7.44 7.31 7.30 7.30 7.29 7.26 7.19 7.19 7.19 7.18 6.96 6.95 6.94 6.93 6.57 6.56 6.55 6.55 6.33 6.33 6.32 6.31 6.21 6.21 6.21 6.20 6.18 6.18 5.06 5.04 3.38 3.38 3.35 3.35 3.30 3.29 3.28 3.28 3.27 3.26 3.25 2.77 2.75 2.73 2.72 2.70 2.68 2.68 1.94 1.94 1.93 1.93 -0.00
1.06 2.05 0.99 1.03 1.04 1.03 1.00 1.00 1.00 1.04 1.17 1.02 3.03
f1 (ppm)

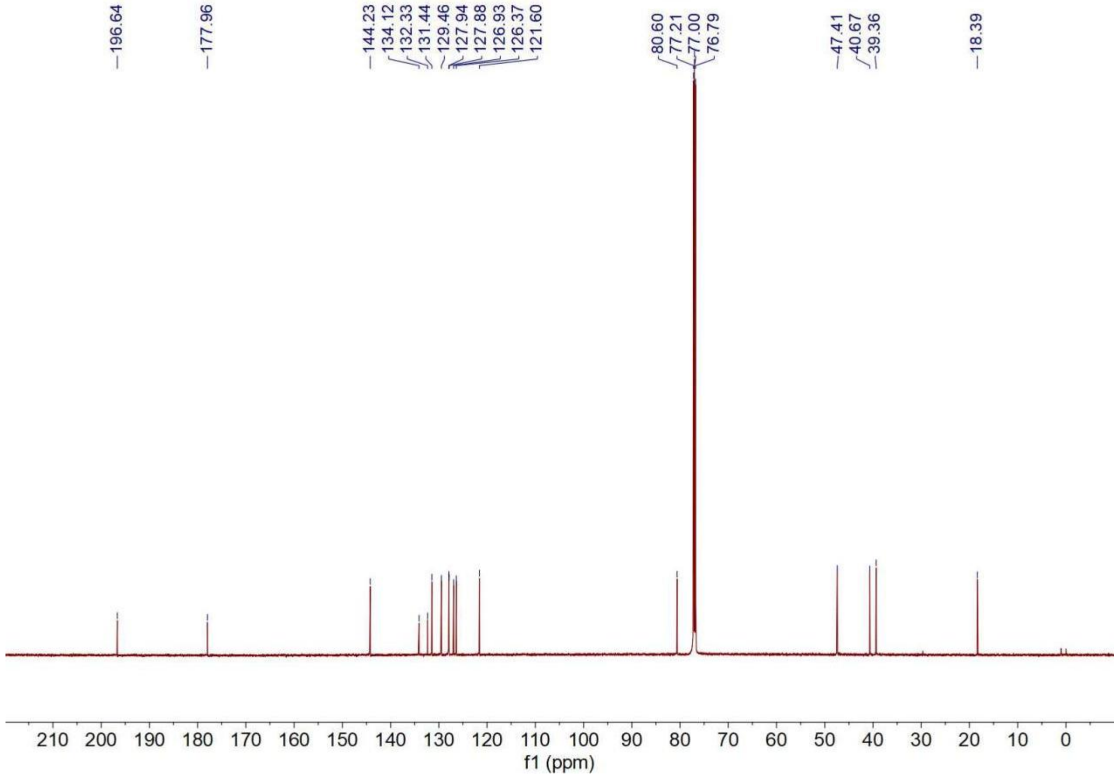
196.64 177.96 144.23 134.12 132.33 131.44 129.46 127.94 127.88 126.93 126.37 121.60 80.60 77.21 77.00 76.79 47.41 40.67 39.36 18.39
f1 (ppm)

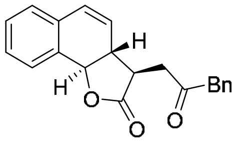
H
H
O
O
O
Bn

(*R*,*R*,*R*)-**3m**

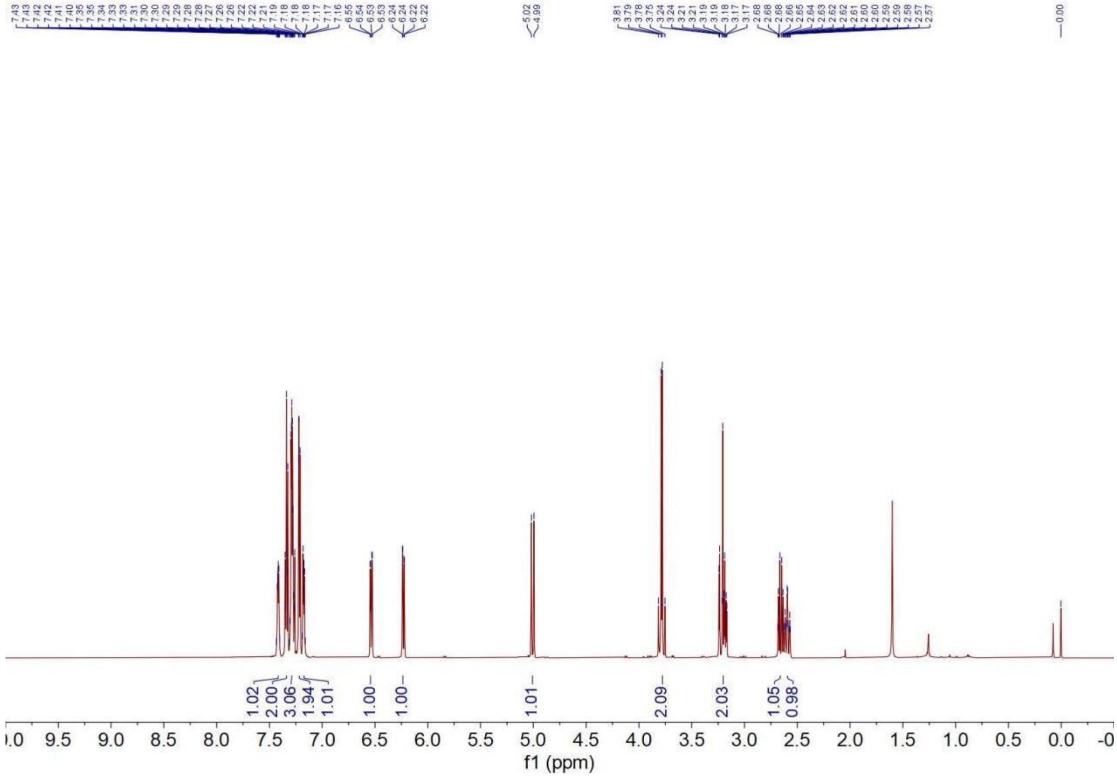
5.02
4.99
0.00
1.02
2.00
3.06
1.94
1.01
1.00
1.00
1.01
2.09
2.03
1.05
0.98
9.5 9.0 8.5 8.0 7.5 7.0 6.5 6.0 5.5 5.0 4.5 4.0 3.5 3.0 2.5 2.0 1.5 1.0 0.5 0.0
f1 (ppm)

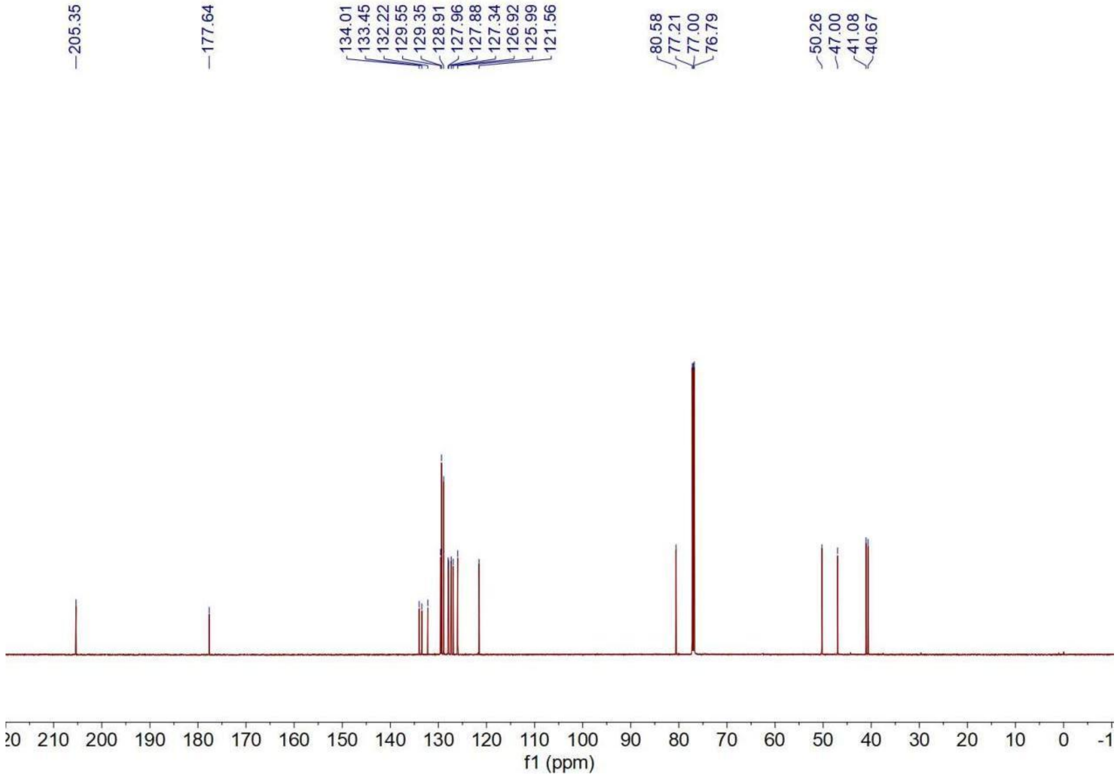
205.35
177.64
134.01
133.45
132.22
129.55
129.35
128.91
127.96
127.88
127.34
126.92
125.99
121.56
80.58
77.21
77.00
76.79
50.26
47.00
41.08
40.67
210 200 190 180 170 160 150 140 130 120 110 100 90 80 70 60 50 40 30 20 10 0
f1 (ppm)

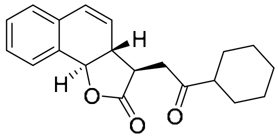

(*R*,*R*,*R*)-**3n**

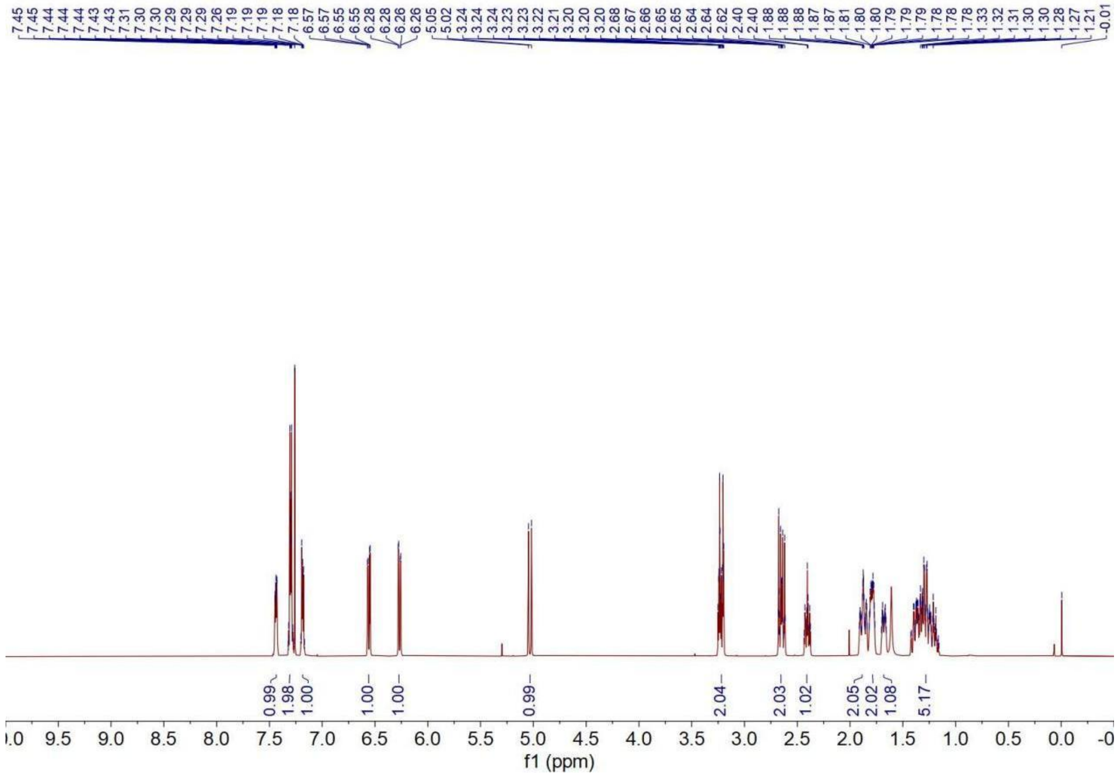
7.45 7.45 7.44 7.44 7.43 7.43 7.31 7.30 7.30 7.29 7.29 7.26 7.19 7.19 7.19 7.18 7.18 6.57 6.57 6.55 6.55 6.28 6.28 6.26 6.26 5.05 5.02 3.24 3.24 3.24 3.23 3.23 3.22 3.21 3.20 3.20 3.20 2.68 2.67 2.66 2.65 2.65 2.64 2.64 2.62 2.40 2.40 1.88 1.88 1.88 1.87 1.87 1.81 1.80 1.80 1.79 1.79 1.79 1.78 1.78 1.33 1.32 1.31 1.30 1.30 1.28 1.27 1.21 -0.01
0.99 1.98 1.00 1.00 1.00 0.99 2.04 2.03 1.02 2.05 2.02 1.08 5.17
f1 (ppm)

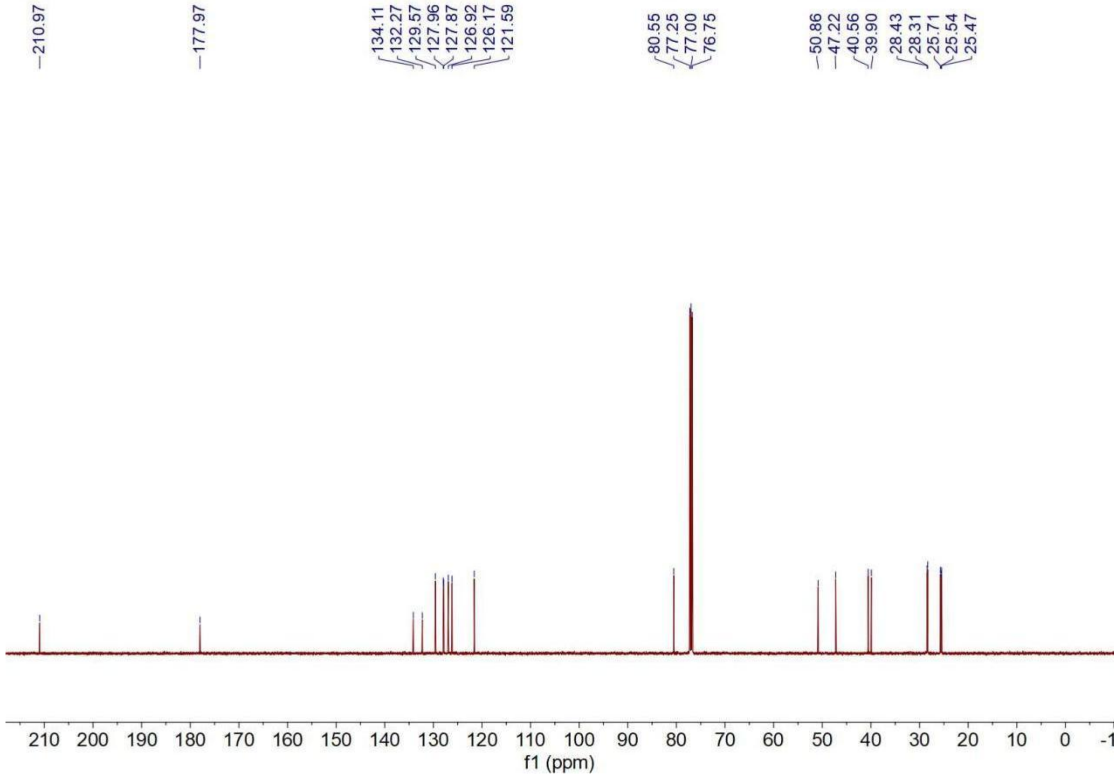
210.97
177.97
134.11 132.27 129.57 127.96 127.87 126.92 126.17 121.59
80.55 77.25 77.00 76.75
50.86 47.22 40.56 39.90 28.43 28.31 25.71 25.54 25.47
210 200 190 180 170 160 150 140 130 120 110 100 90 80 70 60 50 40 30 20 10 0 -1
f1 (ppm)

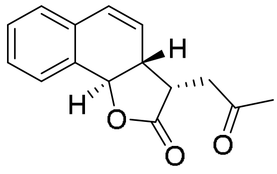
H
H
O
O
O

(*S*,*R*,*R*)-**4a**

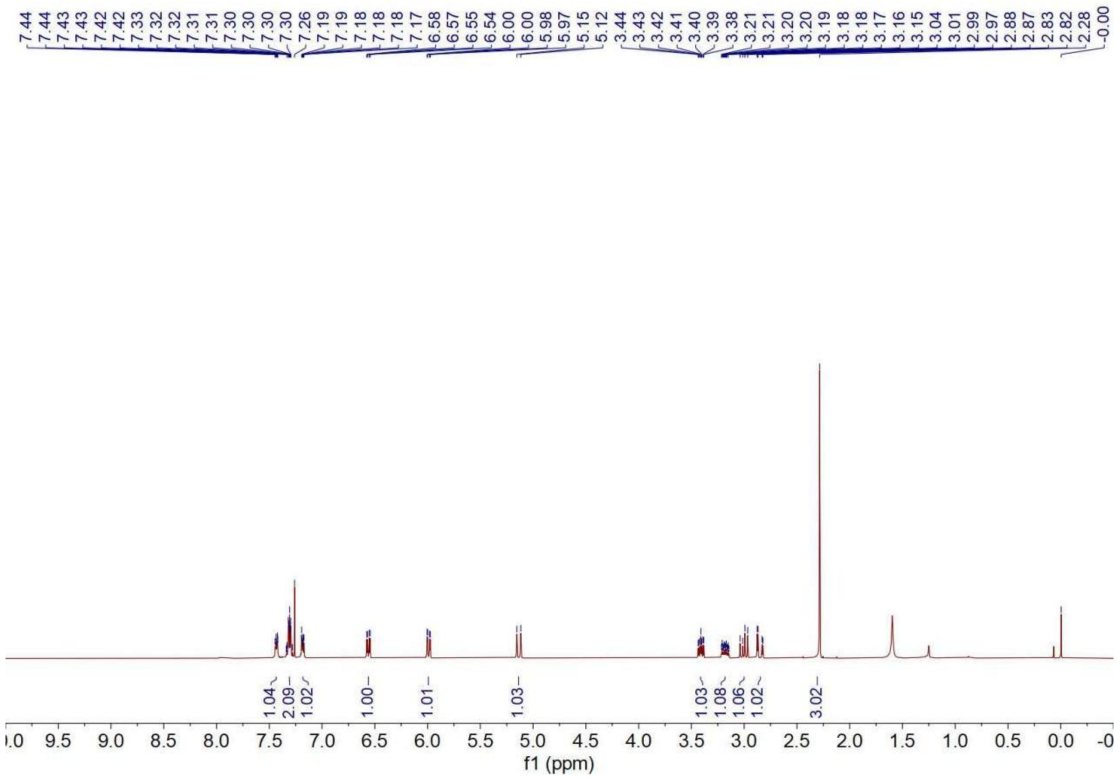
7.44
7.44
7.43
7.43
7.42
7.42
7.33
7.32
7.32
7.31
7.31
7.30
7.30
7.30
7.30
7.26
7.19
7.19
7.18
7.18
7.18
7.17
6.58
6.57
6.55
6.54
6.00
6.00
5.98
5.97
5.15
5.12
3.44
3.43
3.42
3.41
3.40
3.39
3.38
3.21
3.21
3.20
3.20
3.19
3.18
3.18
3.17
3.16
3.15
3.04
3.01
2.99
2.97
2.88
2.87
2.83
2.82
2.28
-0.00
1.04
2.09
1.02
1.00
1.01
1.03
1.03
1.08
1.06
1.02
3.02
f1 (ppm)

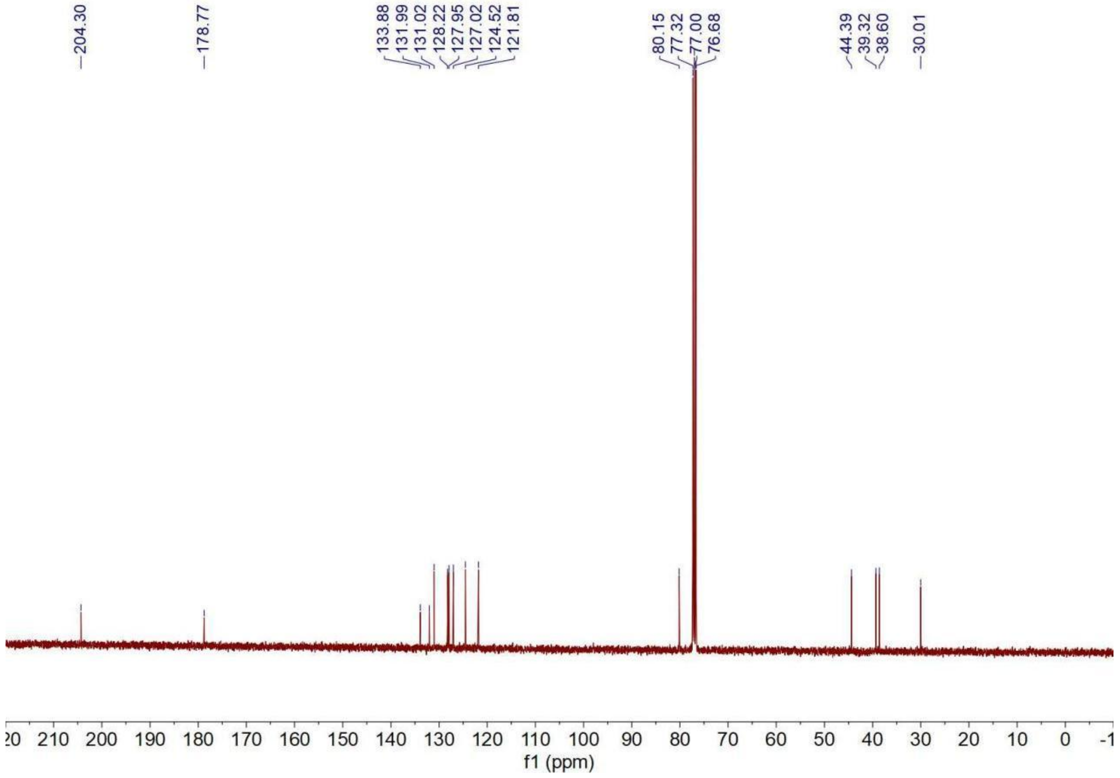
204.30
178.77
133.88
131.99
131.02
128.22
127.95
127.02
124.52
121.81
80.15
77.32
77.00
76.68
44.39
39.32
38.60
30.01
f1 (ppm)

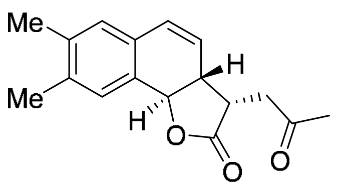

(*S*,*R*,*R*)--**4b**

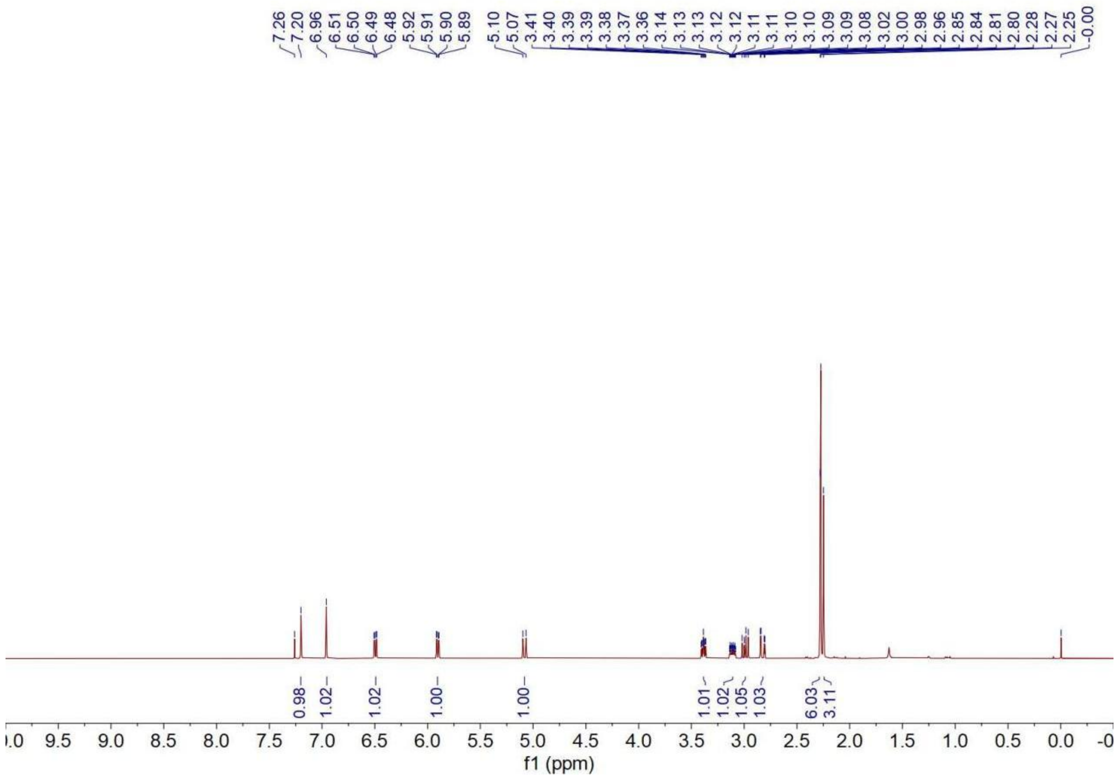

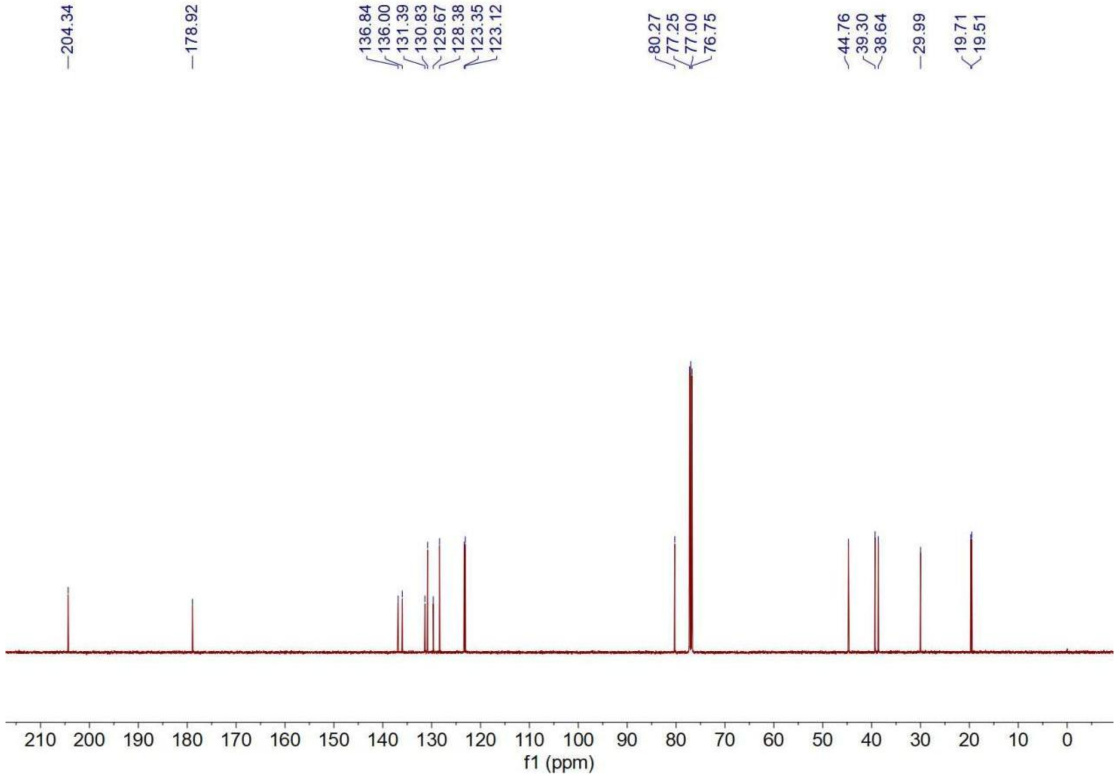

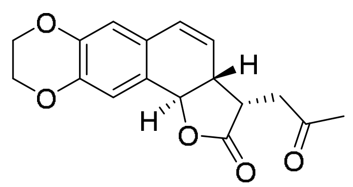
O
O
H
H
O
O
O

(*S*,*R*,*R*)-**4d**

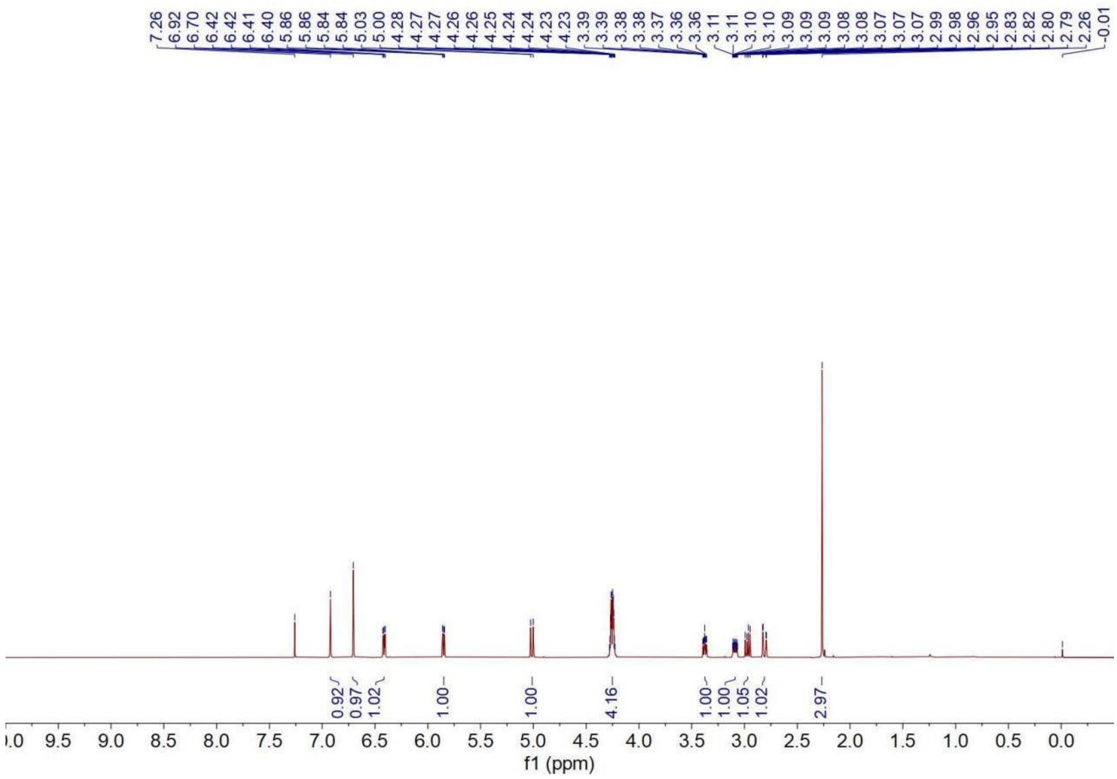
7.26
6.92
6.70
6.42
6.42
6.41
6.40
5.86
5.86
5.84
5.84
5.03
5.00
4.28
4.27
4.27
4.26
4.25
4.24
4.24
4.23
4.23
3.39
3.39
3.38
3.38
3.37
3.36
3.36
3.11
3.11
3.10
3.09
3.09
3.09
3.08
3.08
3.07
3.07
2.99
2.98
2.96
2.95
2.83
2.82
2.80
2.79
2.26
-0.01
0.92
0.97
1.02
1.00
1.00
4.16
1.00
1.00
1.05
1.02
2.97
9.5
9.0
8.5
8.0
7.5
7.0
6.5
6.0
5.5
5.0
4.5
4.0
3.5
3.0
2.5
2.0
1.5
1.0
0.5
0.0
f1 (ppm)

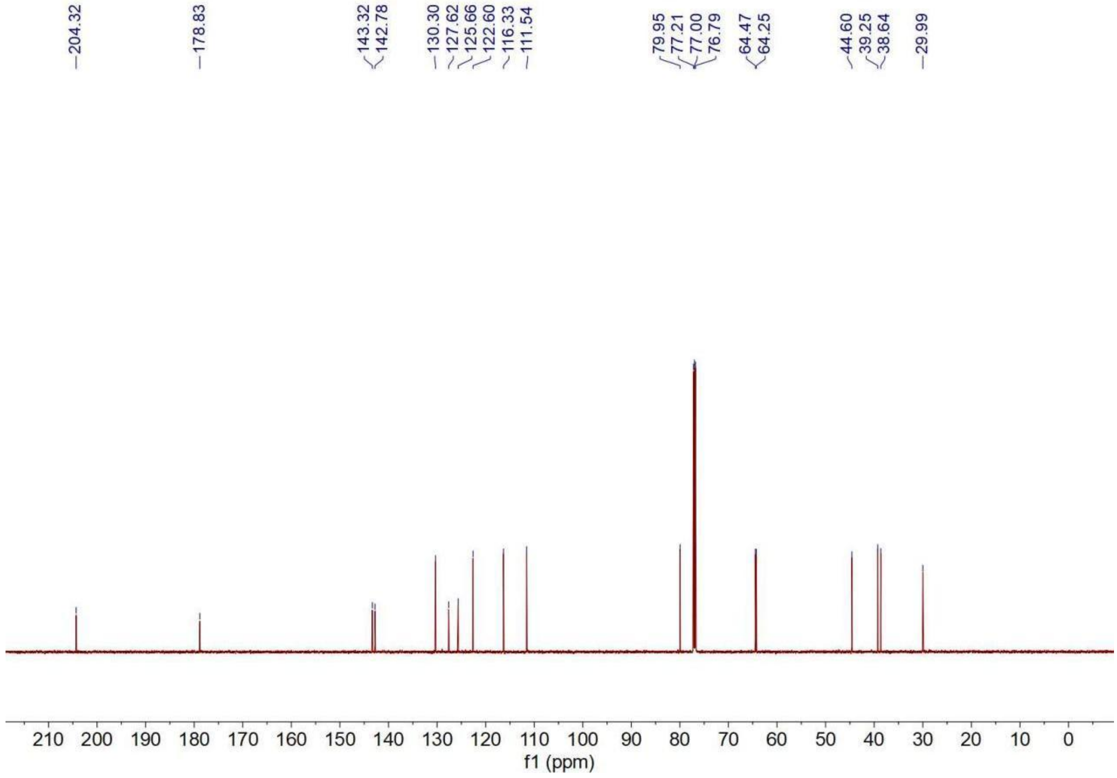
204.32
178.83
143.32
142.78
130.30
127.62
125.66
122.60
116.33
111.54
79.95
77.21
77.00
76.79
64.47
64.25
44.60
39.25
38.64
29.99
210
200
190
180
170
160
150
140
130
120
110
100
90
80
70
60
50
40
30
20
10
0
f1 (ppm)

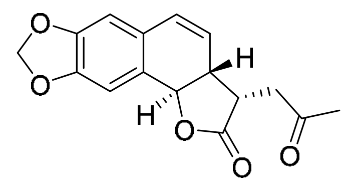

(*S*,*R*,*R*)-**4e**

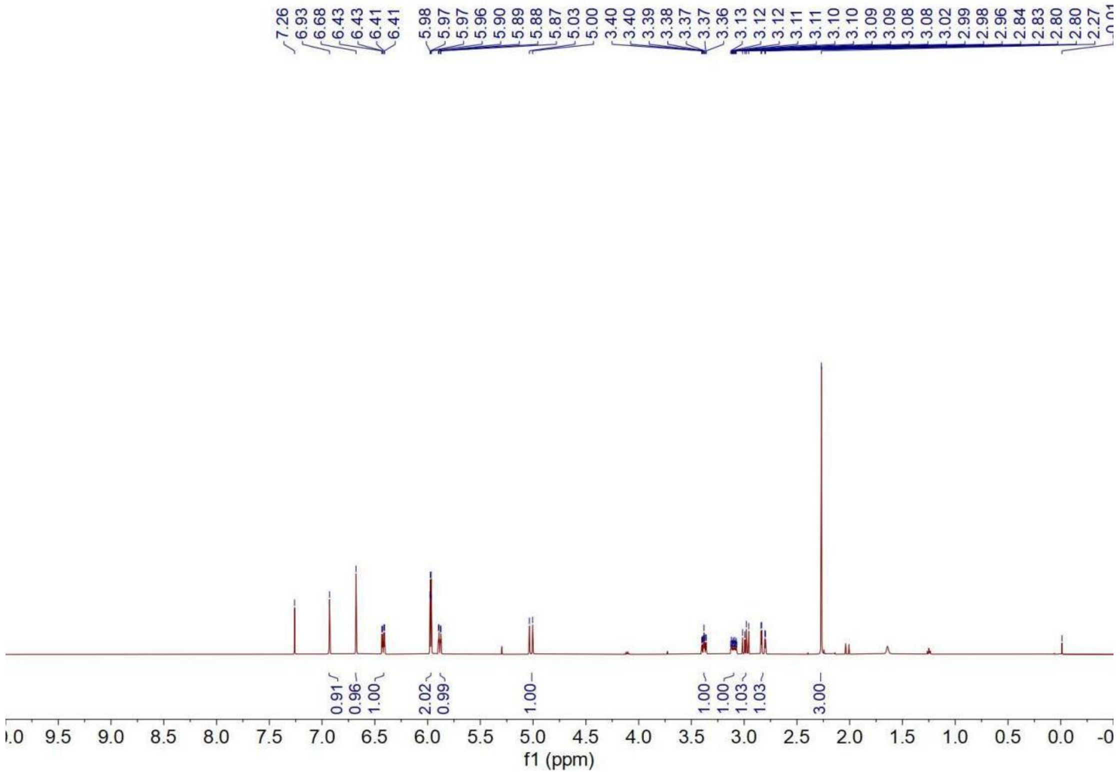
7.26
6.93
6.68
6.43
6.43
6.41
6.41
5.98
5.97
5.97
5.96
5.90
5.89
5.88
5.87
5.03
5.00
3.40
3.40
3.39
3.38
3.37
3.37
3.36
3.13
3.12
3.12
3.11
3.11
3.10
3.09
3.09
3.08
3.02
2.99
2.98
2.96
2.84
2.83
2.80
2.80
2.27
0.91
0.96
1.00
2.02
0.99
1.00
1.00
1.00
1.03
1.03
3.00
9.5 9.0 8.5 8.0 7.5 7.0 6.5 6.0 5.5 5.0 4.5 4.0 3.5 3.0 2.5 2.0 1.5 1.0 0.5 0.0
f1 (ppm)

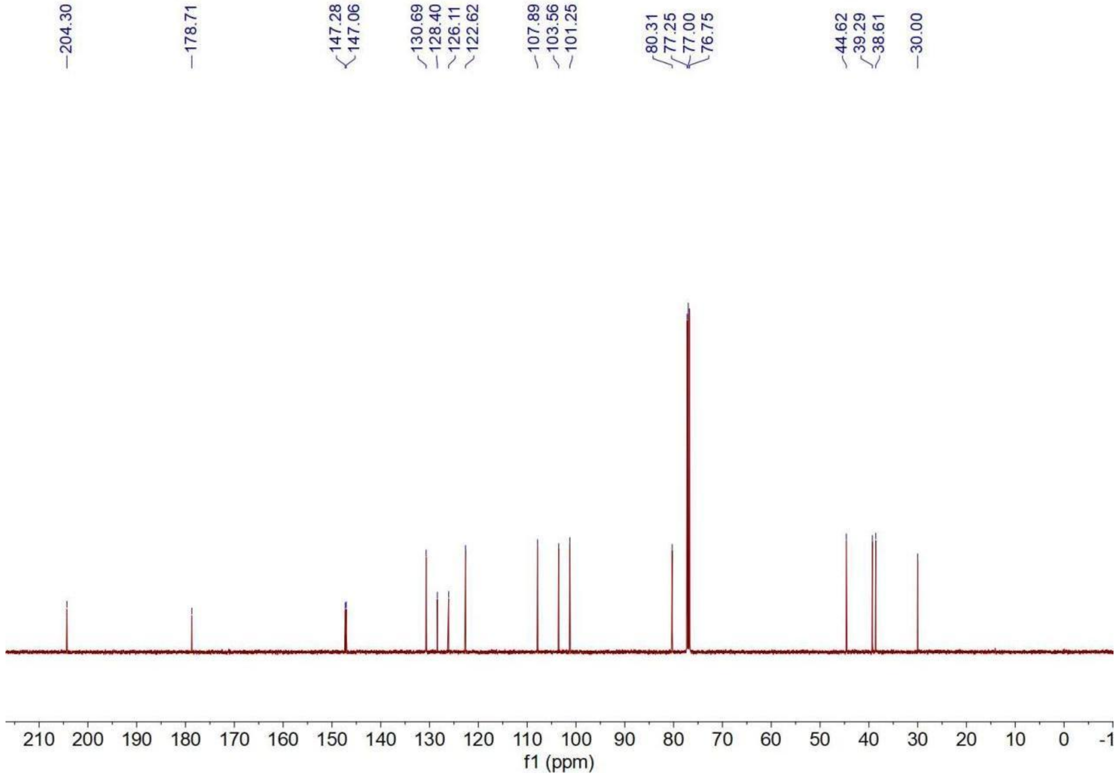
204.30
178.71
147.28
147.06
130.69
128.40
126.11
122.62
107.89
103.56
101.25
80.31
77.25
77.00
76.75
44.62
39.29
38.61
30.00
210 200 190 180 170 160 150 140 130 120 110 100 90 80 70 60 50 40 30 20 10 0
f1 (ppm)

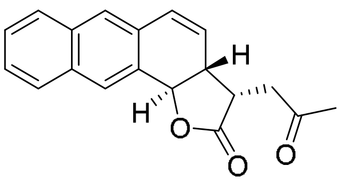

(*S*,*R*,*R*)-**4f**

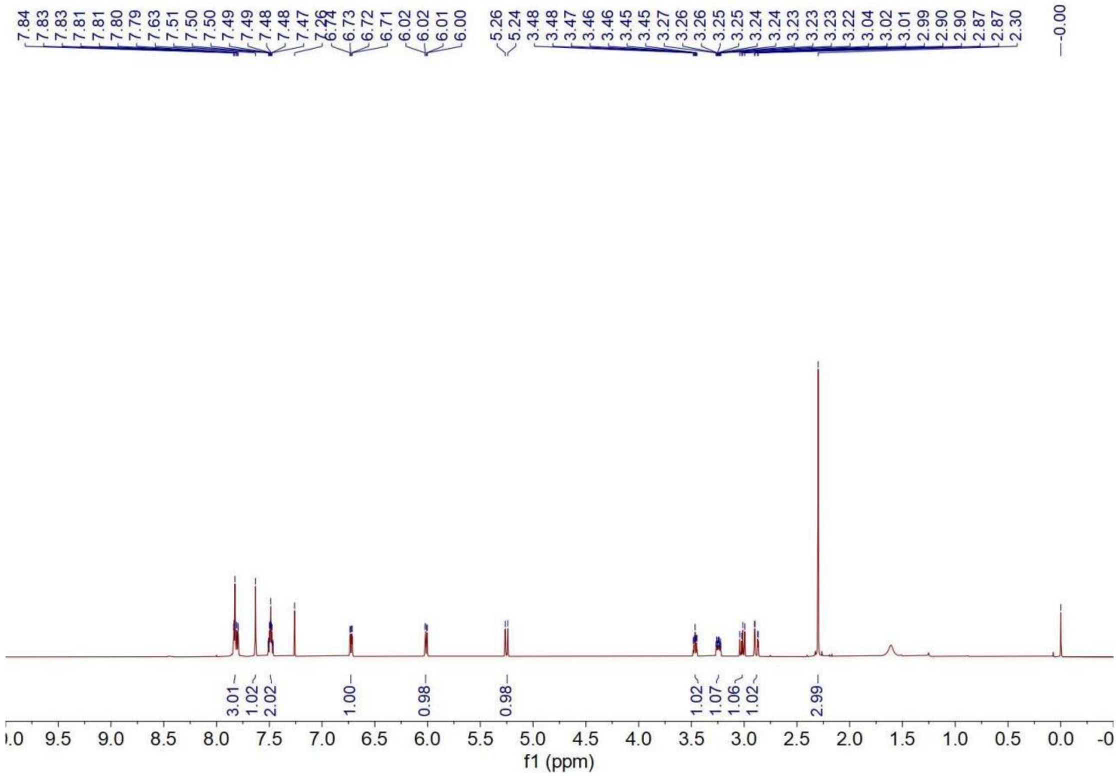
7.84 7.83 7.83 7.81 7.81 7.80 7.79 7.63 7.51 7.50 7.50 7.49 7.49 7.48 7.48 7.47 7.26 6.74 6.73 6.72 6.71 6.02 6.02 6.01 6.00
5.26 5.24 3.48 3.48 3.47 3.46 3.46 3.45 3.45 3.27 3.26 3.26 3.25 3.25 3.24 3.24 3.23 3.23 3.22 3.04 3.02 3.01 2.99 2.90 2.90 2.87 2.87 2.30
-0.00
3.01 1.02 2.02 1.00 0.98 0.98 1.02 1.07 1.06 1.02 2.99
f1 (ppm)

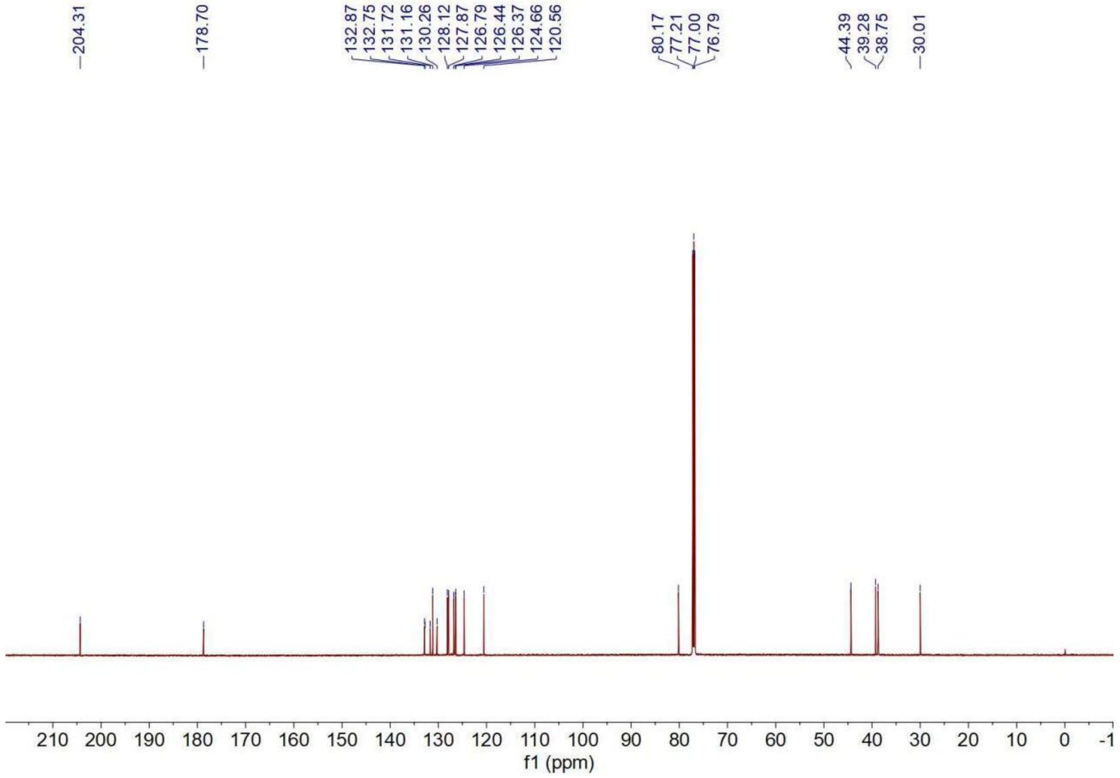
204.31
178.70
132.87 132.75 131.72 131.16 130.26 128.12 127.87 126.79 126.44 126.37 124.66 120.56
80.17 77.21 77.00 76.79
44.39 39.28 38.75
30.01
f1 (ppm)

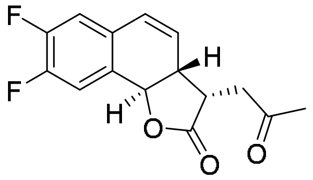
F
F
H
H
O
O
O

(*S*,*R*,*R*)-**4g**

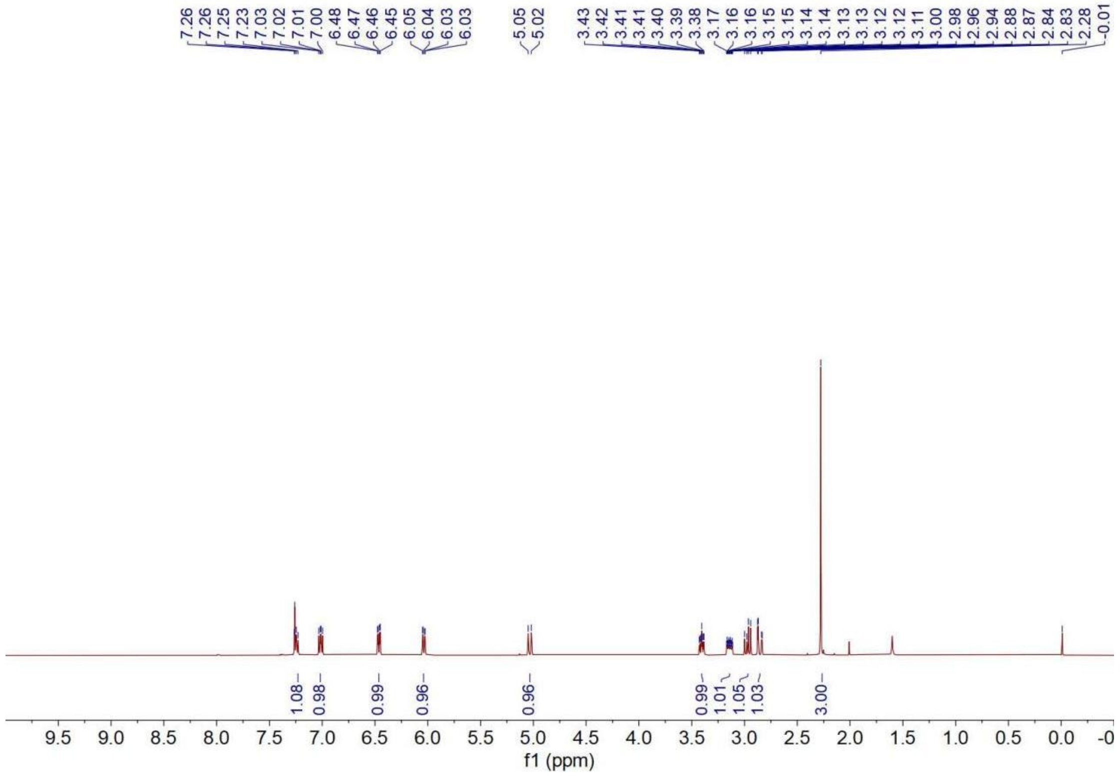
7.26
7.26
7.25
7.23
7.03
7.02
7.01
7.00
6.48
6.47
6.46
6.45
6.05
6.04
6.03
6.03
5.05
5.02
3.43
3.42
3.41
3.41
3.40
3.39
3.38
3.17
3.16
3.16
3.15
3.15
3.14
3.14
3.13
3.13
3.12
3.12
3.11
3.00
2.98
2.96
2.94
2.88
2.87
2.84
2.83
2.28
-0.01
1.08
0.98
0.99
0.96
0.96
0.99
1.01
1.05
1.03
3.00
9.5
9.0
8.5
8.0
7.5
7.0
6.5
6.0
5.5
5.0
4.5
4.0
3.5
3.0
2.5
2.0
1.5
1.0
0.5
0.0
-0
f1 (ppm)

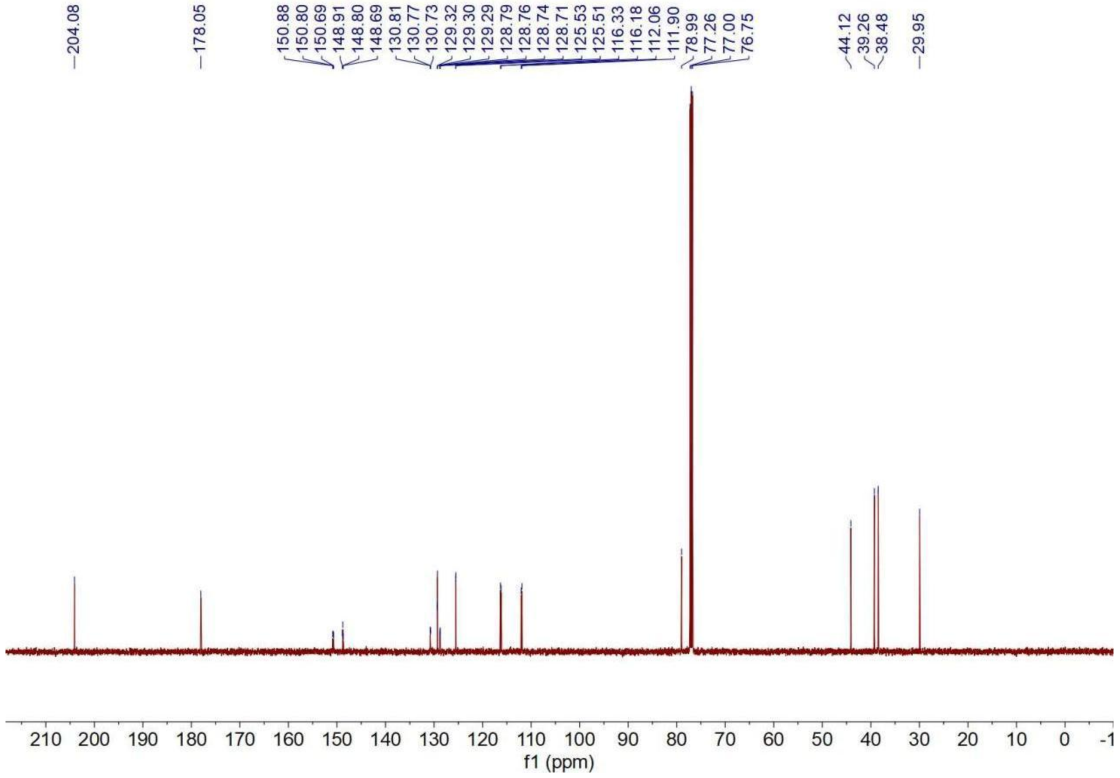
204.08
178.05
150.88
150.80
150.69
148.91
148.80
148.69
130.81
130.77
130.73
129.32
129.30
129.29
128.79
128.76
128.74
128.71
125.53
125.51
116.33
116.18
112.06
111.90
78.99
77.26
77.00
76.75
44.12
39.26
38.48
29.95
210
200
190
180
170
160
150
140
130
120
110
100
90
80
70
60
50
40
30
20
10
0
-1
f1 (ppm)

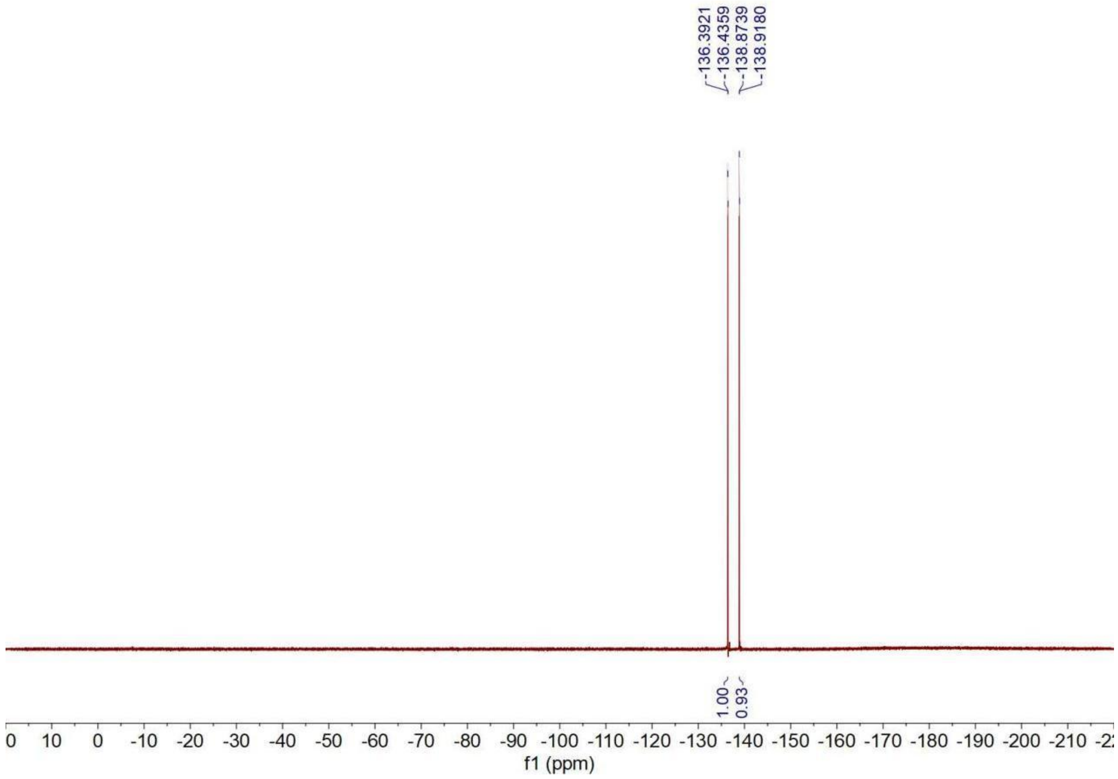

-136.3921
-136.4359
-138.8739
-138.9180
1.00
0.93
0 10 0 -10 -20 -30 -40 -50 -60 -70 -80 -90 -100 -110 -120 -130 -140 -150 -160 -170 -180 -190 -200 -210
f1 (ppm)

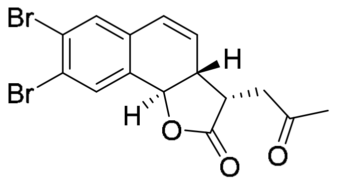
Br
Br
H
H
O
O
O

(*S*,*R*,*R*)-**4h**

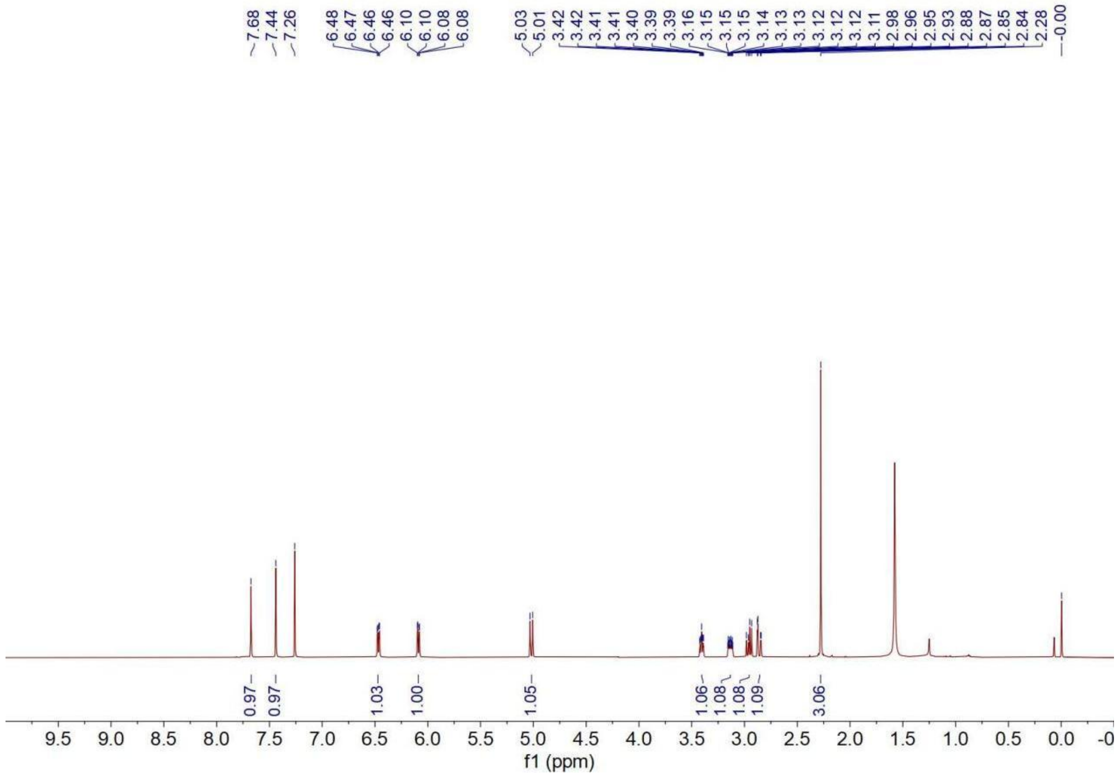
7.68
7.44
7.26
6.48
6.47
6.46
6.46
6.10
6.10
6.08
6.08
5.03
5.01
3.42
3.41
3.41
3.40
3.39
3.39
3.16
3.15
3.15
3.15
3.14
3.13
3.13
3.12
3.12
3.12
3.11
2.98
2.96
2.95
2.93
2.88
2.87
2.85
2.84
2.28
-0.00
0.97
0.97
1.03
1.00
1.05
1.06
1.08
1.08
1.09
3.06
9.5 9.0 8.5 8.0 7.5 7.0 6.5 6.0 5.5 5.0 4.5 4.0 3.5 3.0 2.5 2.0 1.5 1.0 0.5 0.0 -0
f1 (ppm)

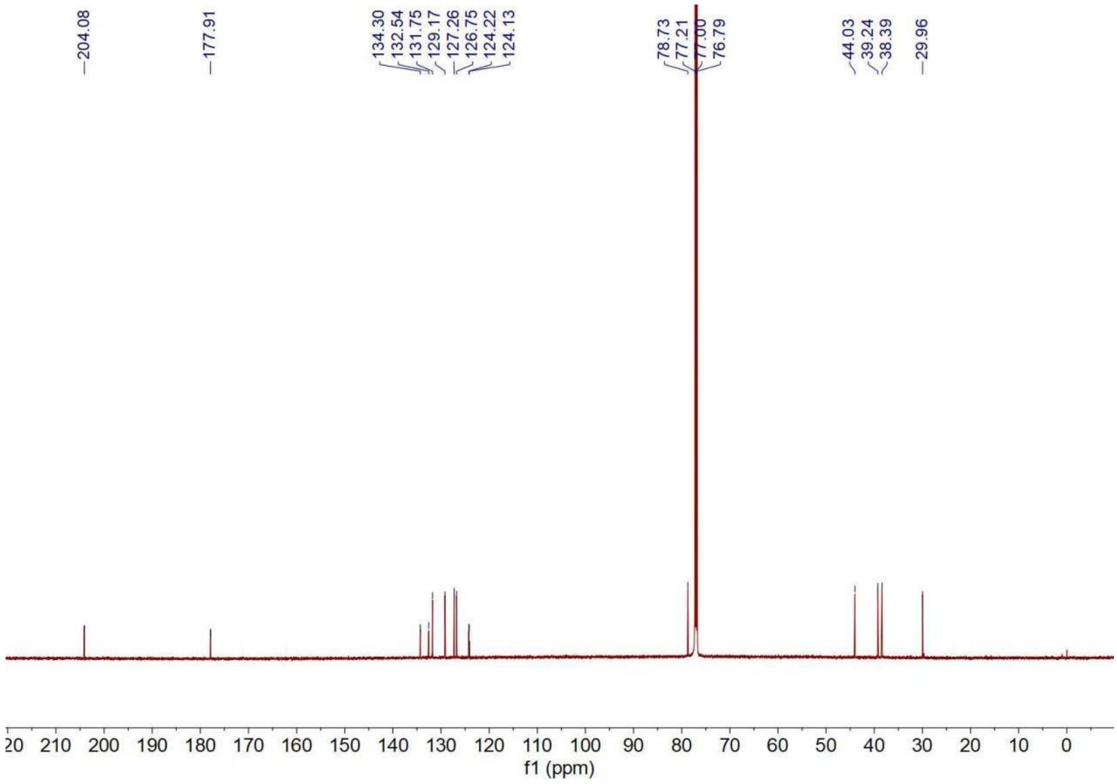
204.08
177.91
134.30
132.54
131.75
129.17
127.26
126.75
124.22
124.13
78.73
77.21
77.00
76.79
44.03
39.24
38.39
29.96
20 210 200 190 180 170 160 150 140 130 120 110 100 90 80 70 60 50 40 30 20 10 0
f1 (ppm)

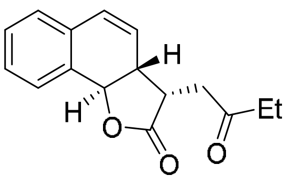

(*S*,*R*,*R*)-**4i**

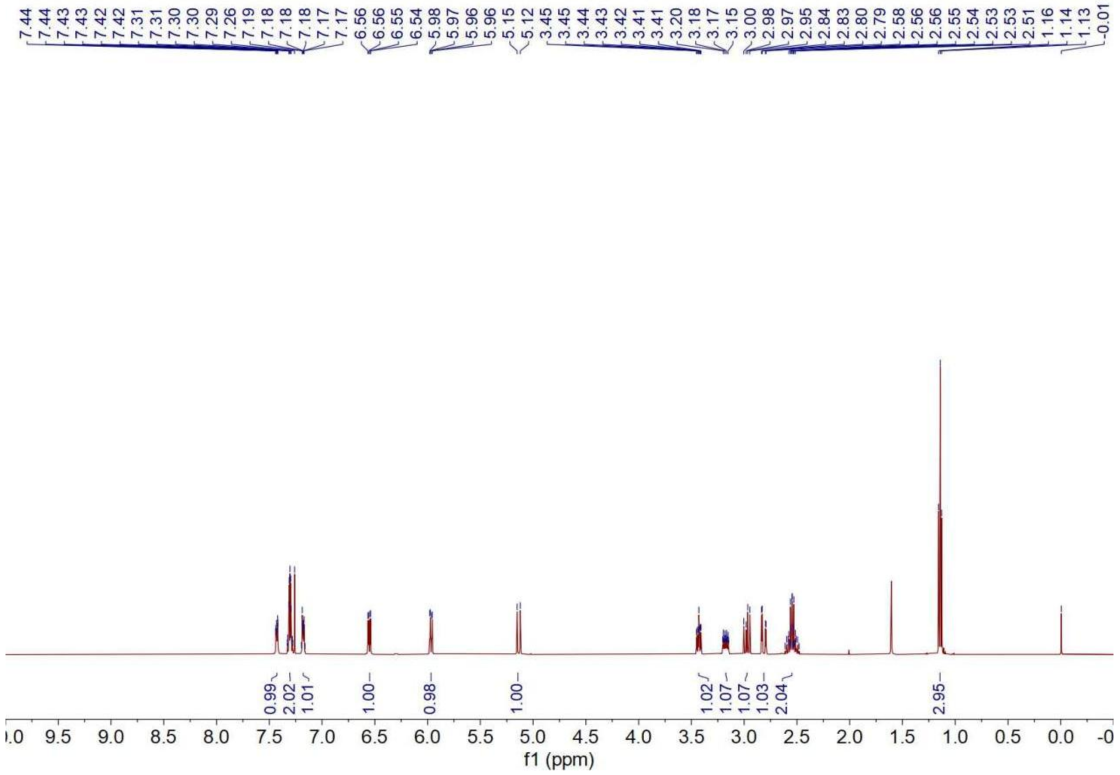

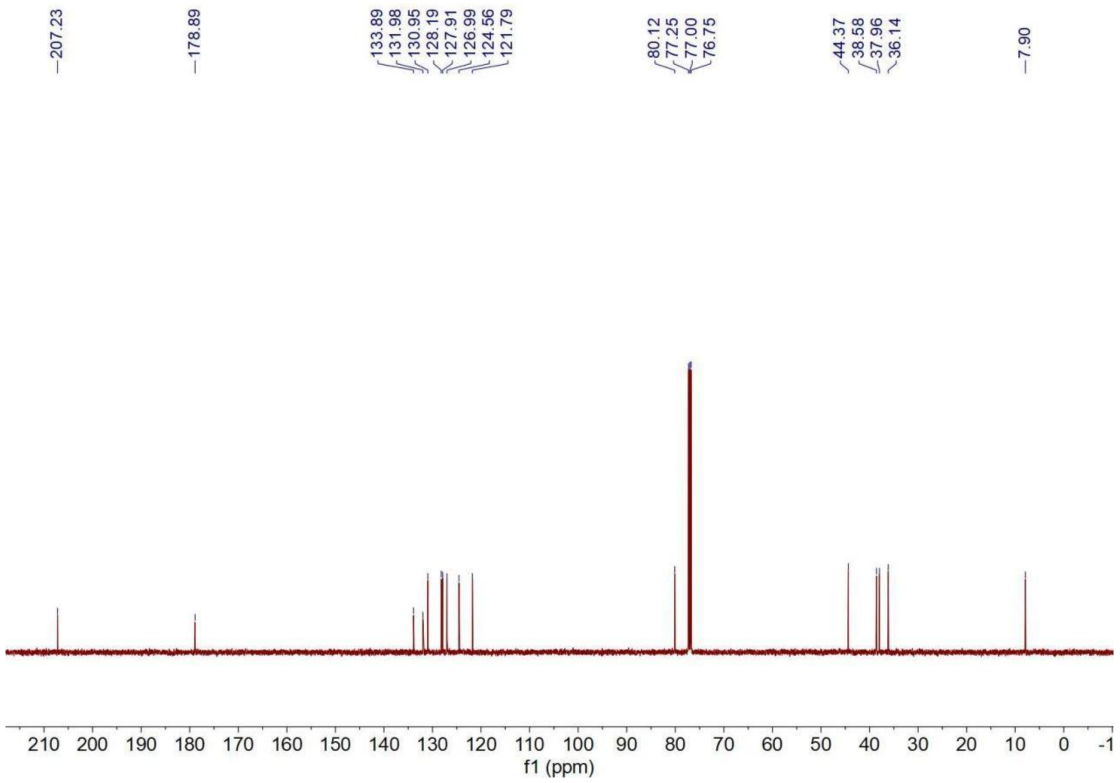

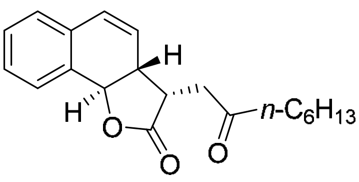

(*S*,*R*,*R*)-**4j**

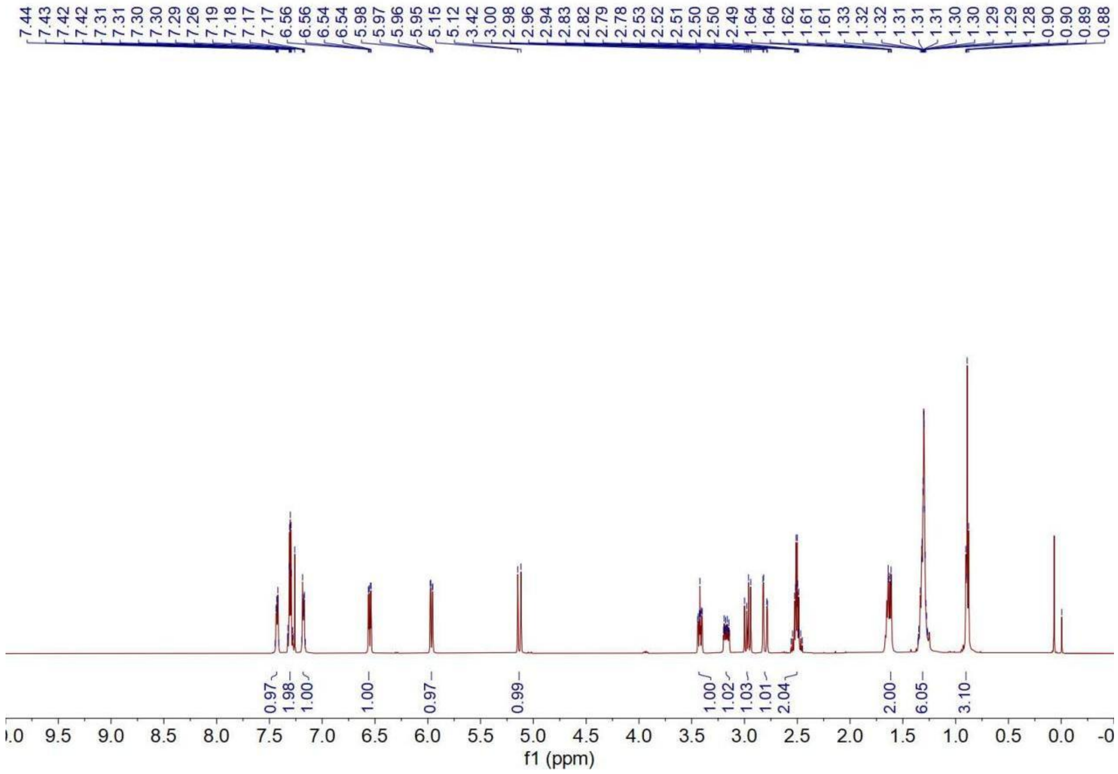

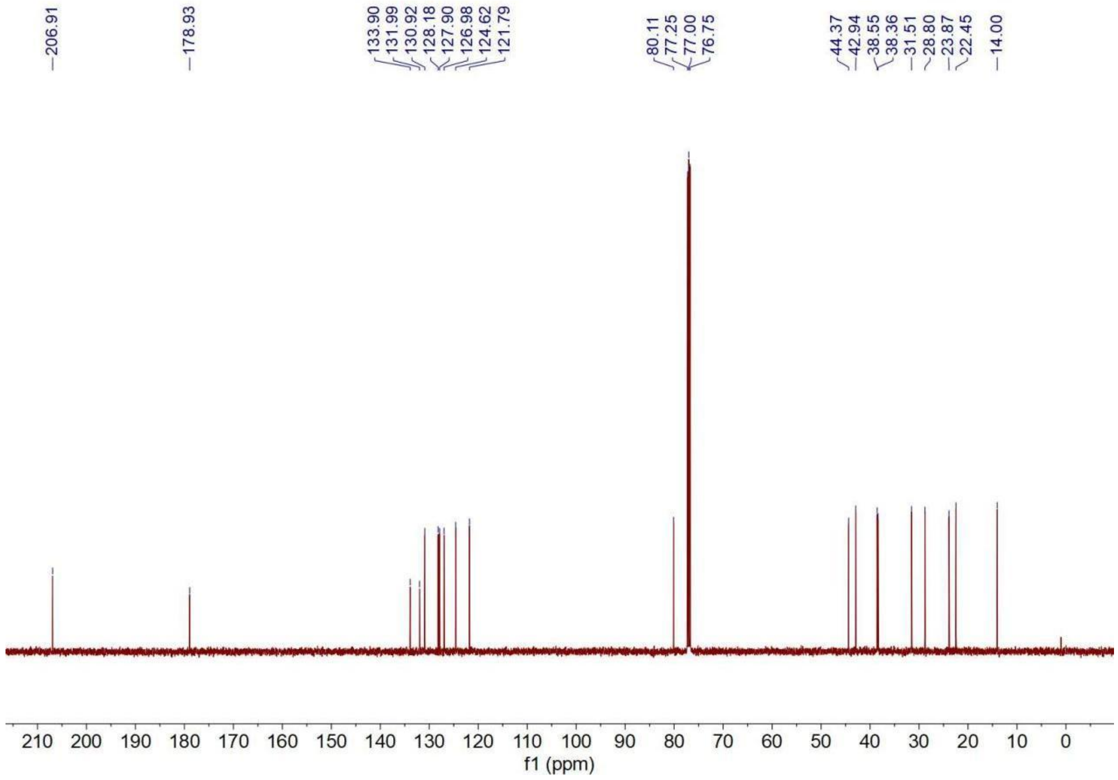

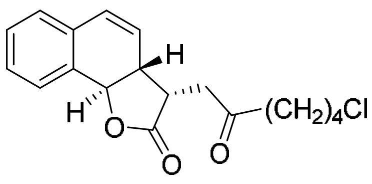
H
H
O
O
O
(CH2)4Cl

(*S*,*R*,*R*)-**4k**

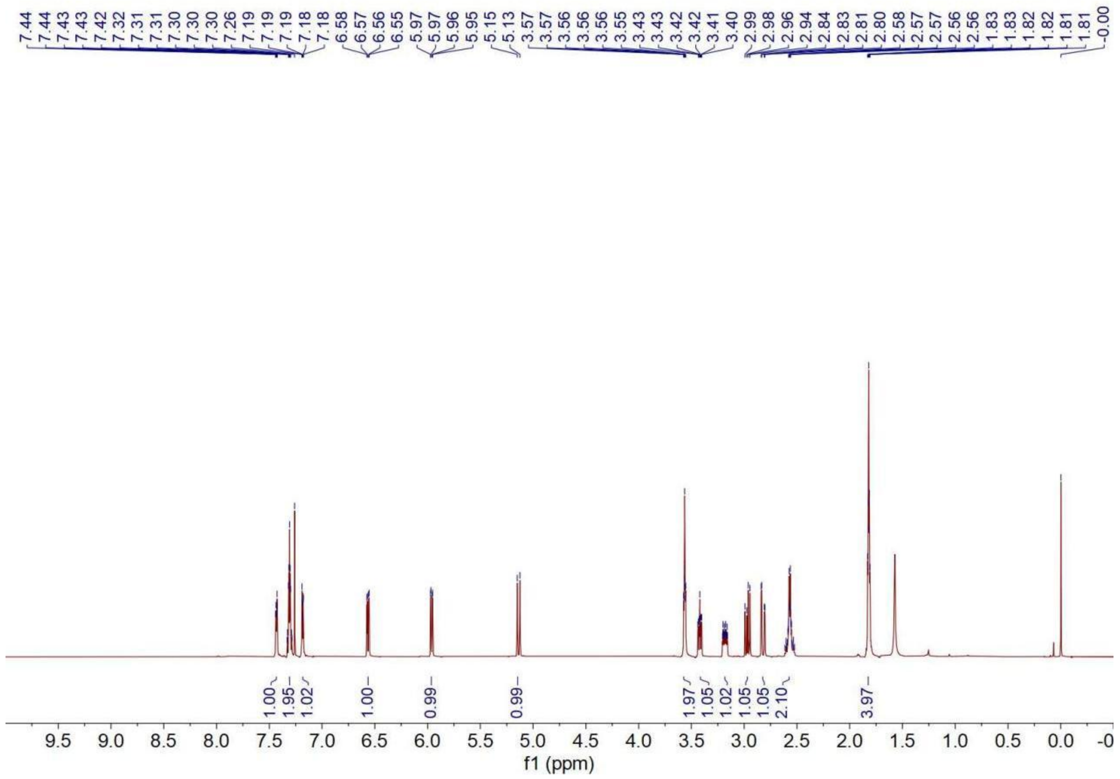
7.44
7.44
7.43
7.43
7.42
7.32
7.31
7.31
7.30
7.30
7.26
7.19
7.19
7.18
7.18
6.58
6.57
6.56
6.55
5.97
5.97
5.96
5.95
5.15
5.13
3.57
3.57
3.56
3.56
3.55
3.43
3.43
3.42
3.42
3.41
3.40
2.99
2.98
2.96
2.94
2.84
2.83
2.81
2.80
2.58
2.57
2.57
2.56
2.56
1.83
1.83
1.82
1.82
1.81
1.81
-0.00
1.00
1.95
1.02
1.00
0.99
0.99
1.97
1.05
1.02
1.05
1.05
2.10
3.97
9.5
9.0
8.5
8.0
7.5
7.0
6.5
6.0
5.5
5.0
4.5
4.0
3.5
3.0
2.5
2.0
1.5
1.0
0.5
0.0
-0
f1 (ppm)

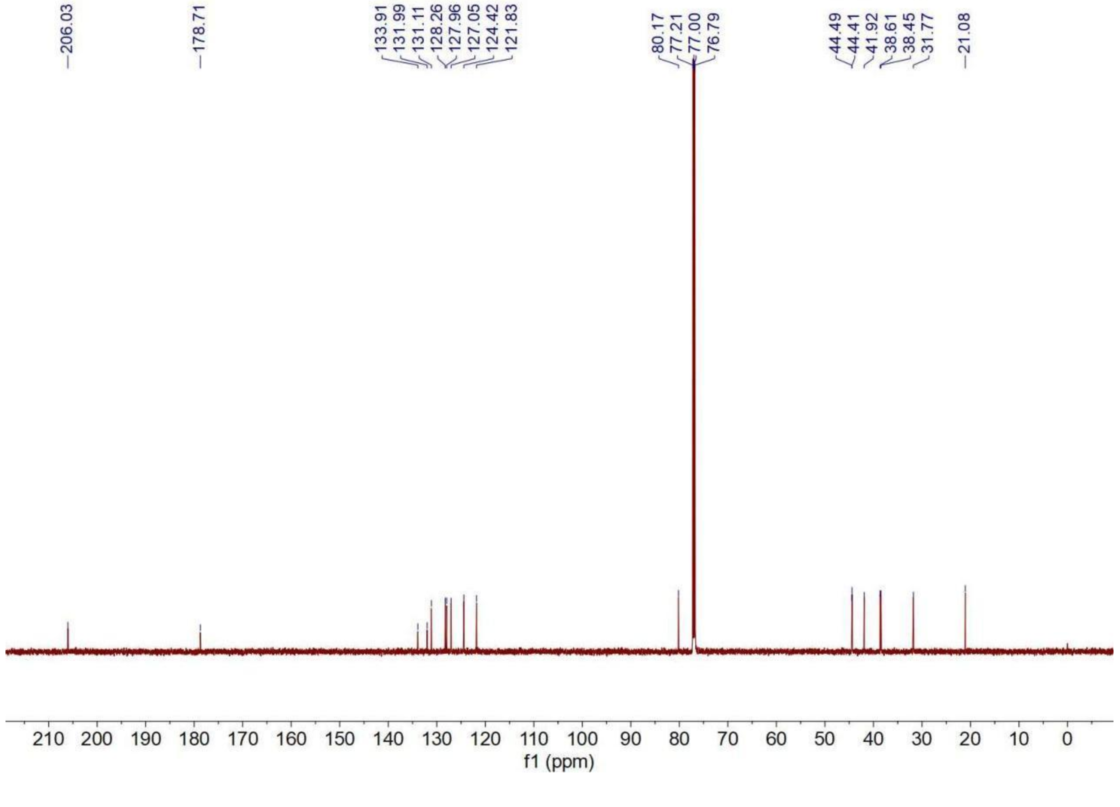
206.03
178.71
133.91
131.99
131.11
128.26
127.96
127.05
124.42
121.83
80.17
77.21
77.00
76.79
44.49
44.41
41.92
38.61
38.45
31.77
21.08
210
200
190
180
170
160
150
140
130
120
110
100
90
80
70
60
50
40
30
20
10
0
f1 (ppm)

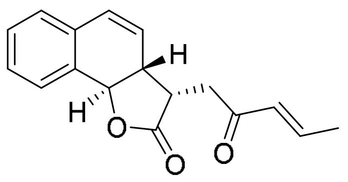
H
H
O
O
O

(*S*,*R*,*R*)-**4l**

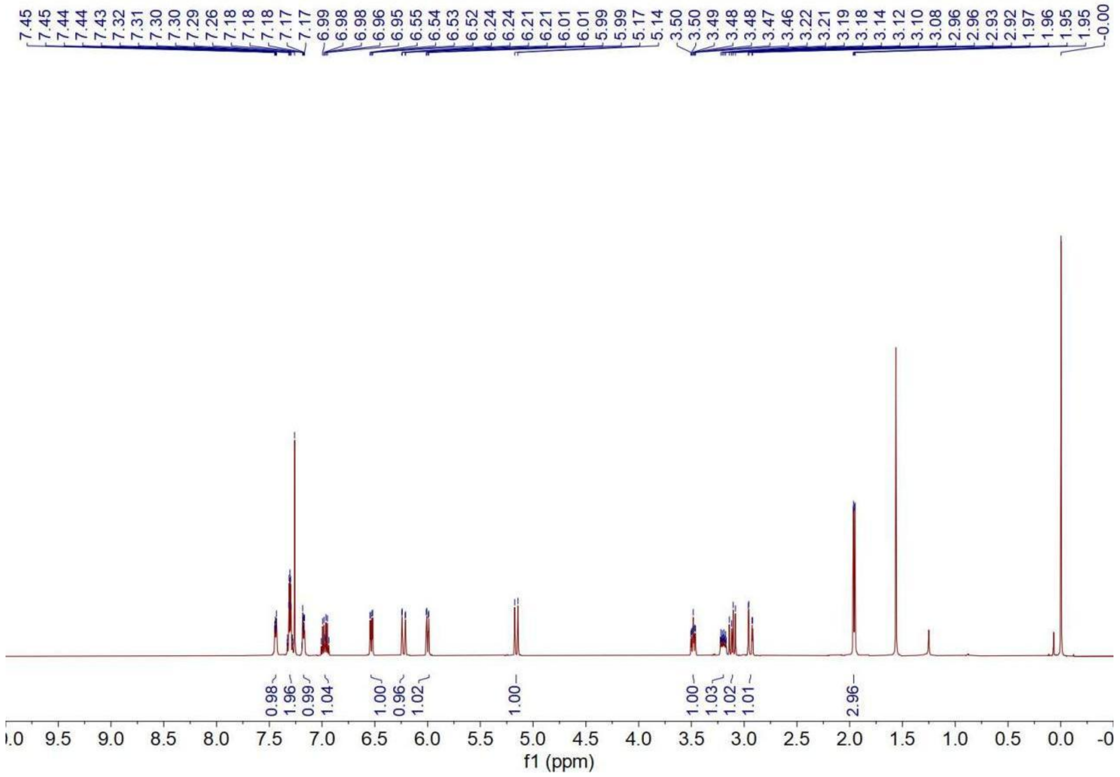
7.45 7.45 7.44 7.44 7.43 7.32 7.31 7.30 7.30 7.29 7.26 7.18 7.18 7.18 7.17 7.17 6.99 6.98 6.98 6.96 6.95 6.55 6.54 6.53 6.52 6.24 6.24 6.21 6.21 6.01 6.01 5.99 5.99 5.17 5.14 3.50 3.50 3.49 3.48 3.48 3.47 3.46 3.22 3.21 3.19 3.18 3.14 3.12 3.10 3.08 2.96 2.96 2.93 2.92 1.97 1.96 1.95 1.95 -0.00
0.98 1.96 0.99 1.04 1.00 0.96 1.02 1.00 1.00 1.03 1.02 1.01 2.96
f1 (ppm)

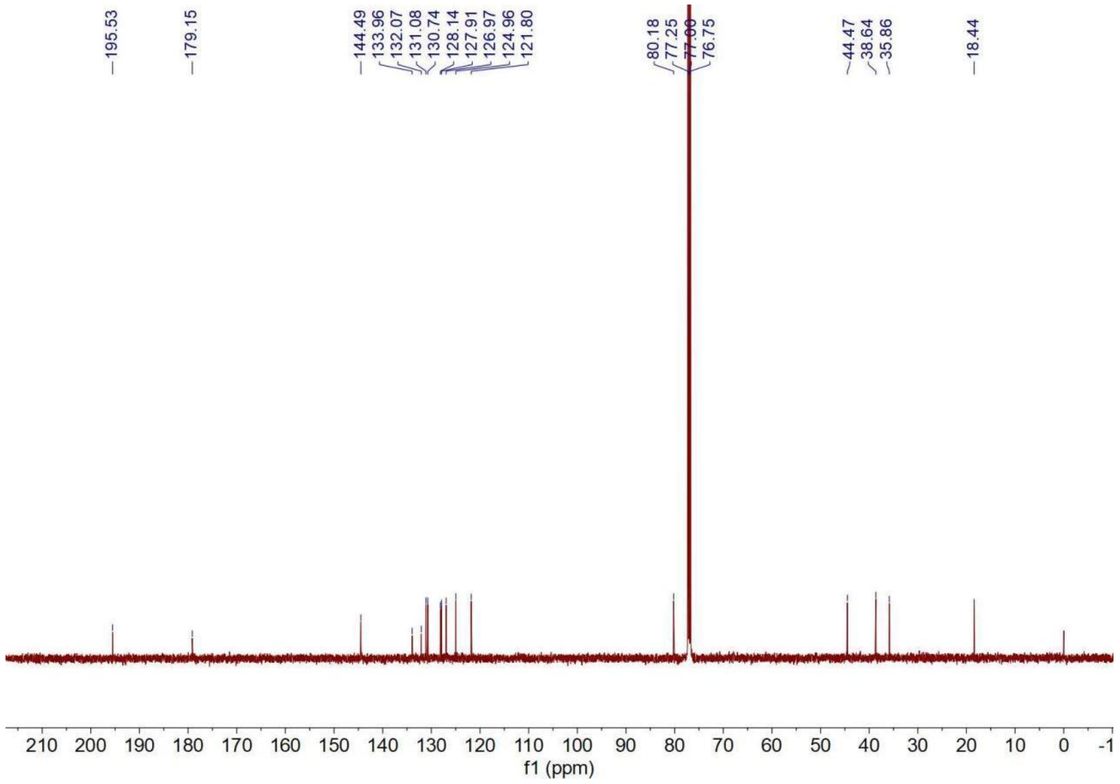
195.53 179.15 144.49 133.96 132.07 131.08 130.74 128.14 127.91 126.97 124.96 121.80 80.18 77.25 77.00 76.75 44.47 38.64 35.86 18.44
f1 (ppm)

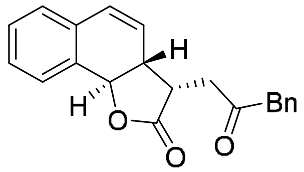
H
H
O
O
O
Bn

(*S*,*R*,*R*)-**4m**

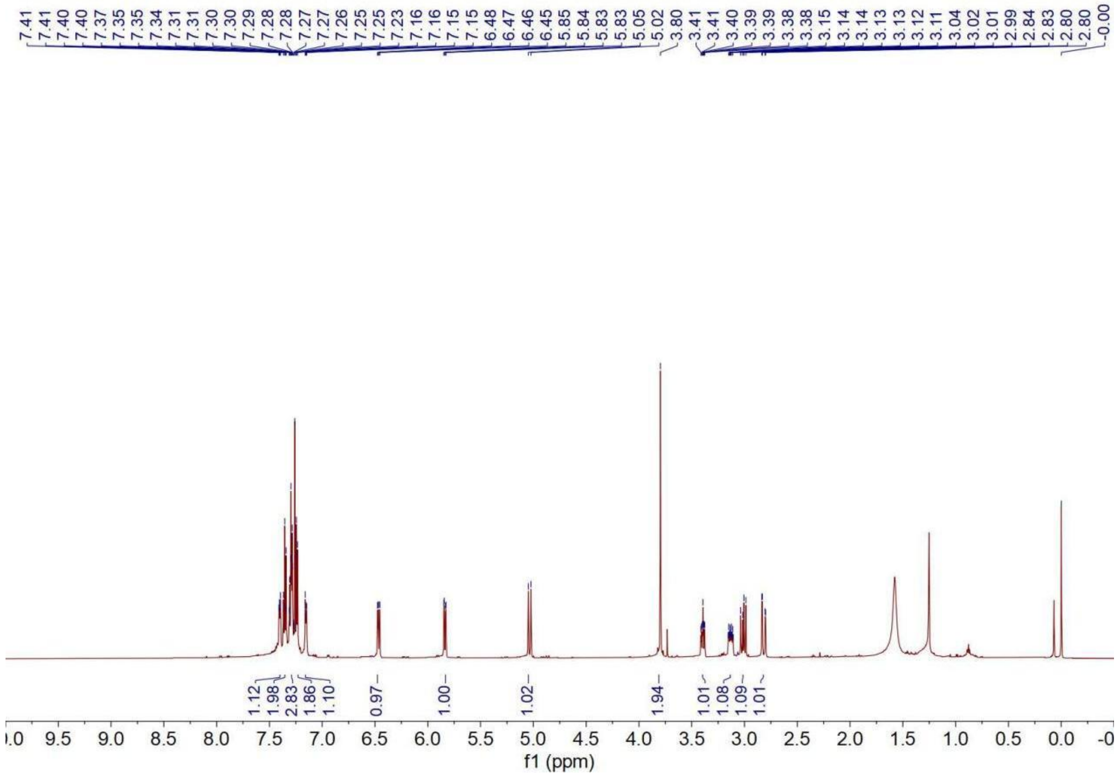
7.41
7.41
7.40
7.40
7.37
7.35
7.35
7.34
7.31
7.30
7.30
7.29
7.28
7.28
7.27
7.27
7.26
7.25
7.25
7.23
7.16
7.16
7.15
7.15
6.48
6.47
6.46
6.45
5.85
5.84
5.83
5.83
5.05
5.02
3.80
3.41
3.41
3.40
3.39
3.39
3.38
3.38
3.15
3.14
3.14
3.13
3.13
3.12
3.11
3.04
3.02
3.01
2.99
2.84
2.83
2.80
2.80
-0.00
1.12
1.98
2.83
1.86
1.10
0.97
1.00
1.02
1.94
1.01
1.08
1.09
1.01
f1 (ppm)

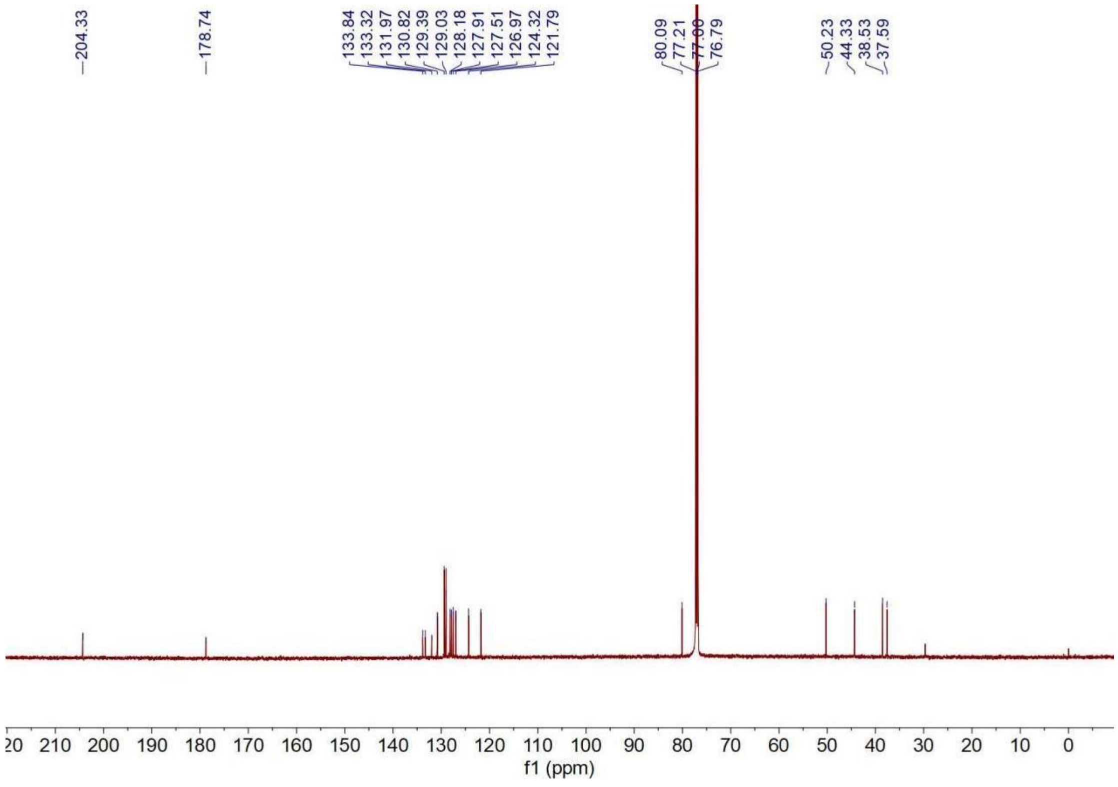
204.33
178.74
133.84
133.32
131.97
130.82
129.39
129.03
128.18
127.91
127.51
126.97
124.32
121.79
80.09
77.21
76.79
50.23
44.33
38.53
37.59
f1 (ppm)

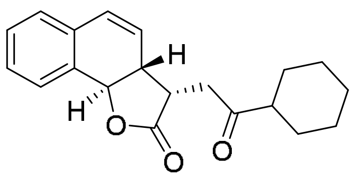
H
H
O
O
O

(*S*,*R*,*R*)-**4n**

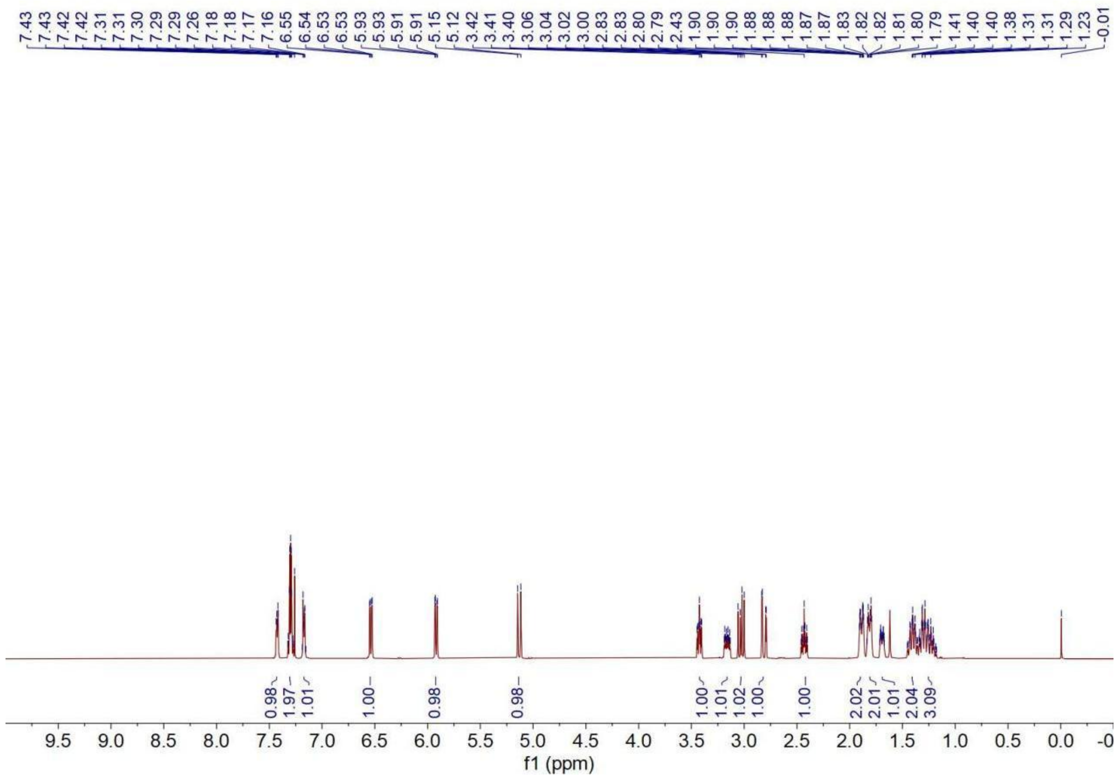
7.43
7.43
7.42
7.31
7.31
7.30
7.29
7.26
7.18
7.18
7.17
7.16
6.55
6.54
6.53
6.53
5.93
5.93
5.91
5.91
5.15
5.12
3.42
3.41
3.40
3.06
3.04
3.02
3.00
2.83
2.80
2.79
2.43
1.90
1.90
1.90
1.88
1.88
1.88
1.87
1.87
1.83
1.82
1.82
1.81
1.80
1.79
1.41
1.40
1.40
1.38
1.31
1.29
1.23
-0.01
0.98
1.97
1.01
1.00
0.98
0.98
1.00
1.01
1.02
1.00
1.00
2.02
2.01
1.01
2.04
3.09
9.5 9.0 8.5 8.0 7.5 7.0 6.5 6.0 5.5 5.0 4.5 4.0 3.5 3.0 2.5 2.0 1.5 1.0 0.5 0.0 -0
f1 (ppm)

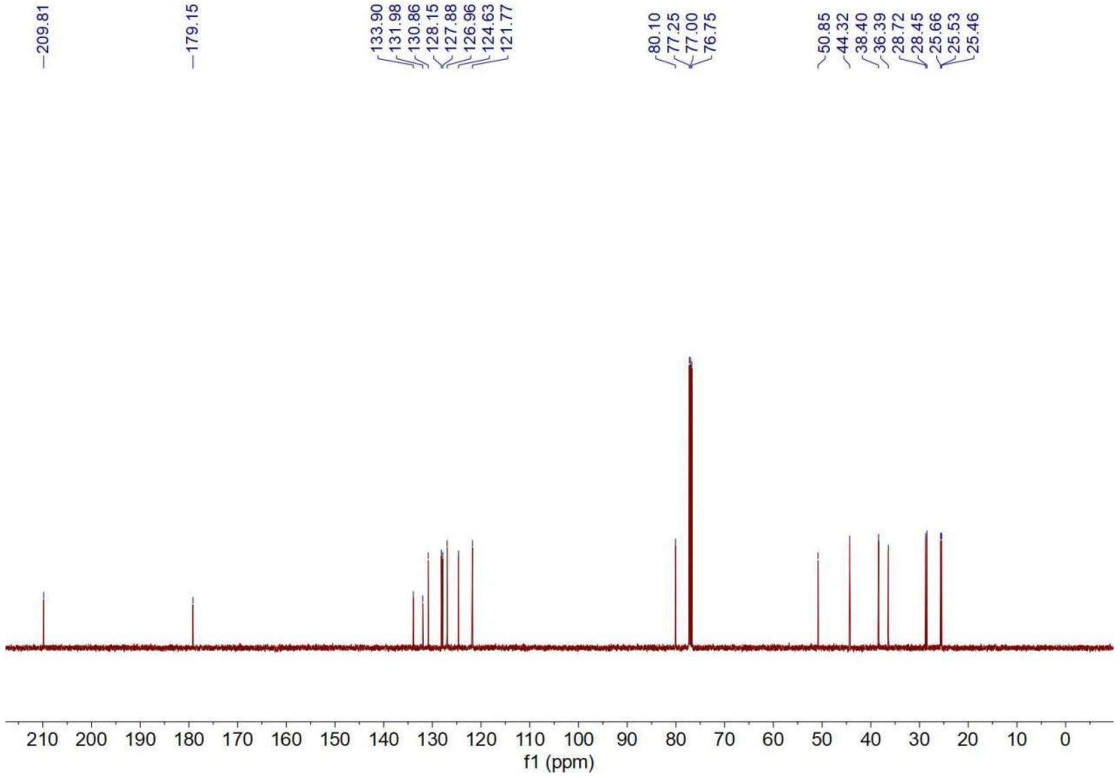
209.81
179.15
133.90
131.98
130.86
128.15
127.88
126.96
124.63
121.77
80.10
77.25
77.00
76.75
50.85
44.32
38.40
36.39
28.72
28.45
25.66
25.53
25.46
210 200 190 180 170 160 150 140 130 120 110 100 90 80 70 60 50 40 30 20 10 0
f1 (ppm)

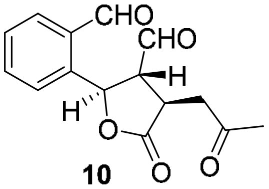

10

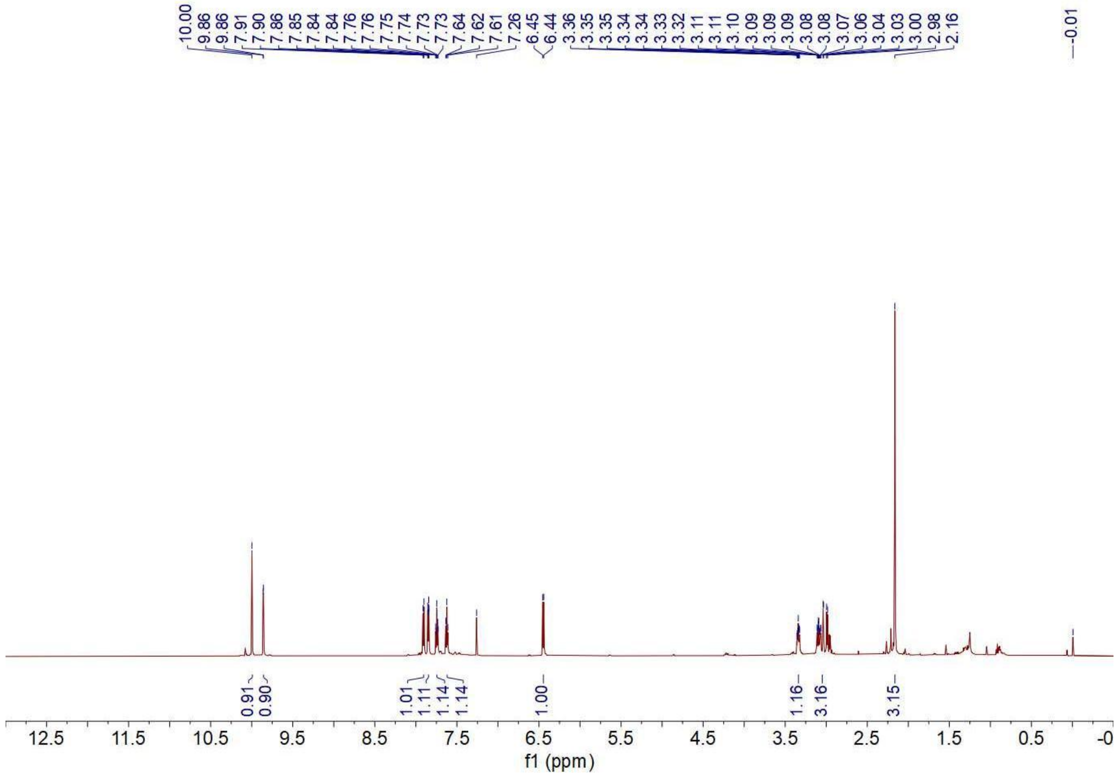

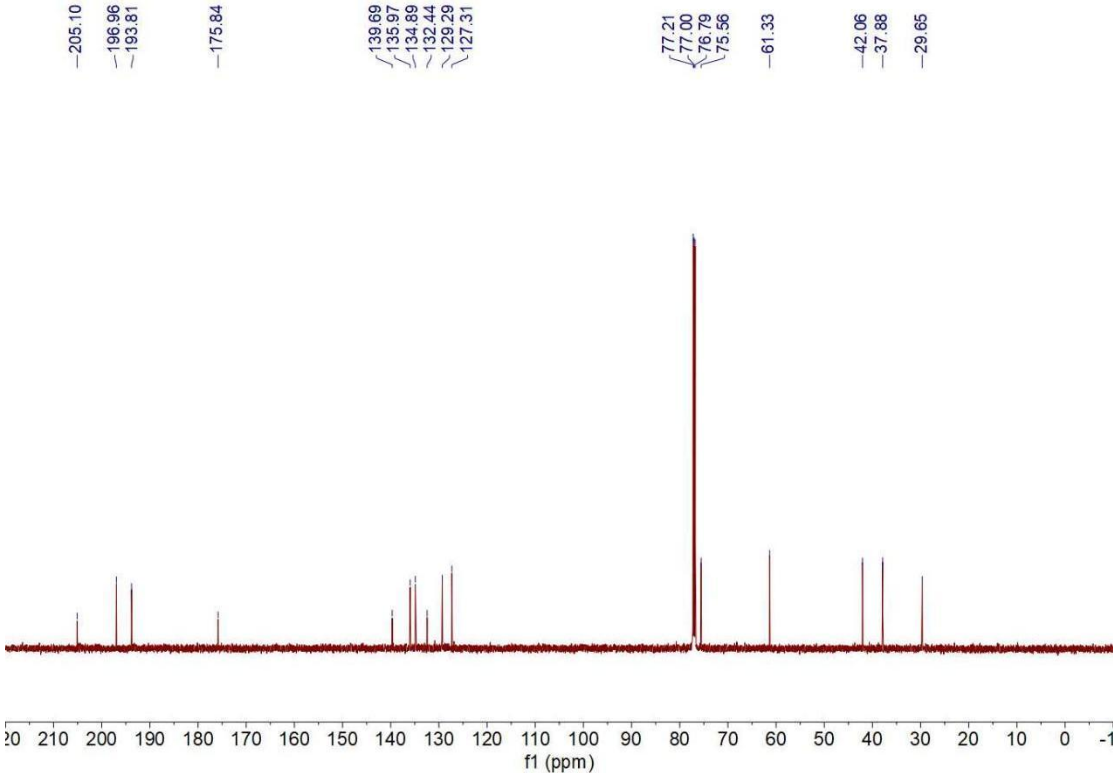

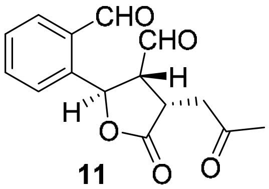
CHO
CHO
H
H
O
O
O

11

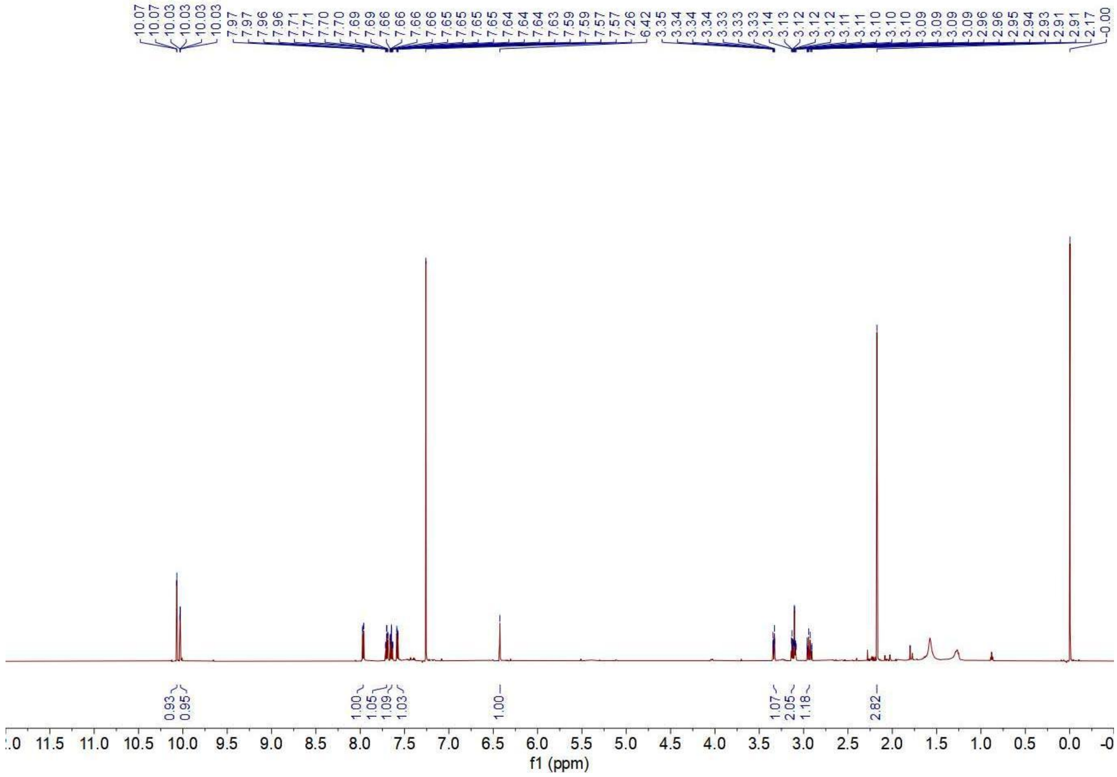
10.07 10.07 10.03 10.03 10.03 10.03 7.97 7.97 7.96 7.96 7.71 7.71 7.70 7.69 7.69 7.66 7.66 7.66 7.65 7.65 7.65 7.64 7.64 7.63 7.59 7.59 7.57 7.26 6.42 3.35 3.34 3.34 3.33 3.33 3.33 3.14 3.13 3.12 3.12 3.11 3.10 3.10 3.09 3.09 3.09 2.96 2.95 2.94 2.93 2.91 2.91 2.17 -0.00
0.93 0.95 1.00 1.05 1.09 1.03 1.00 1.07 2.05 1.18 2.82
f1 (ppm)

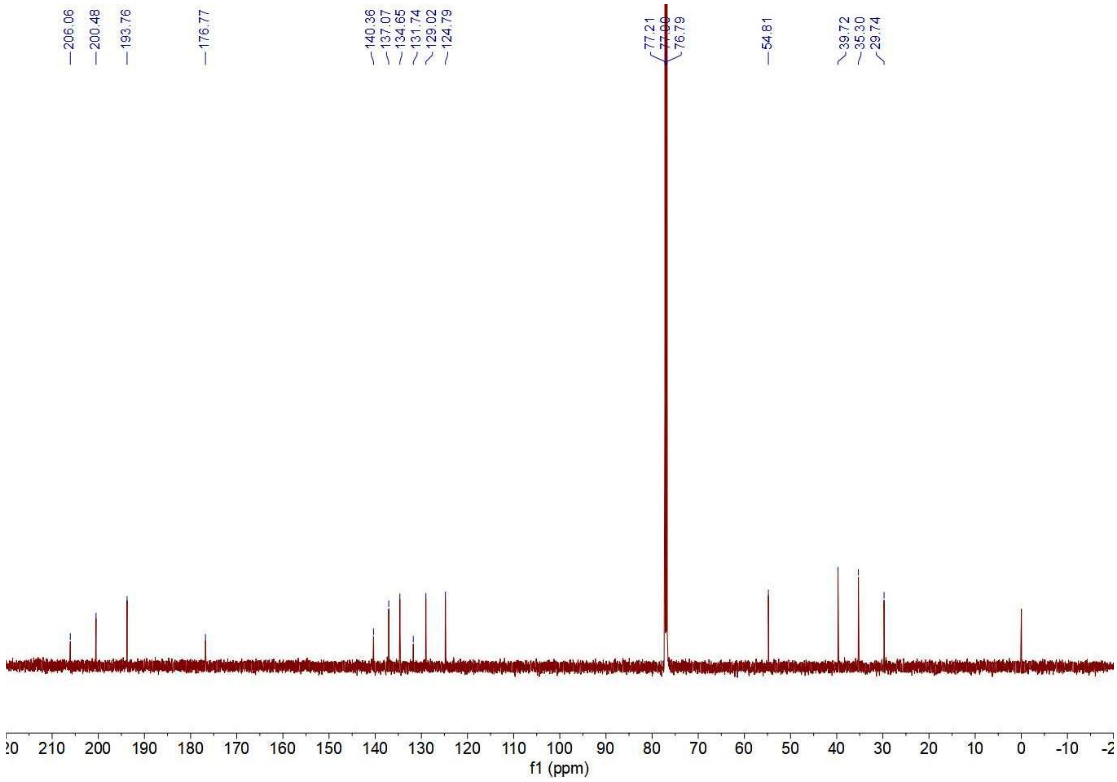
206.06 200.48 193.76 176.77 140.36 137.07 134.65 131.74 129.02 124.79 77.21 77.00 76.79 54.81 39.72 35.30 29.74
f1 (ppm)

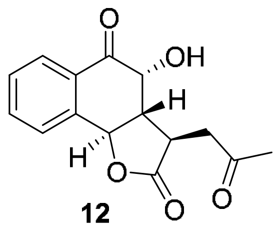

12

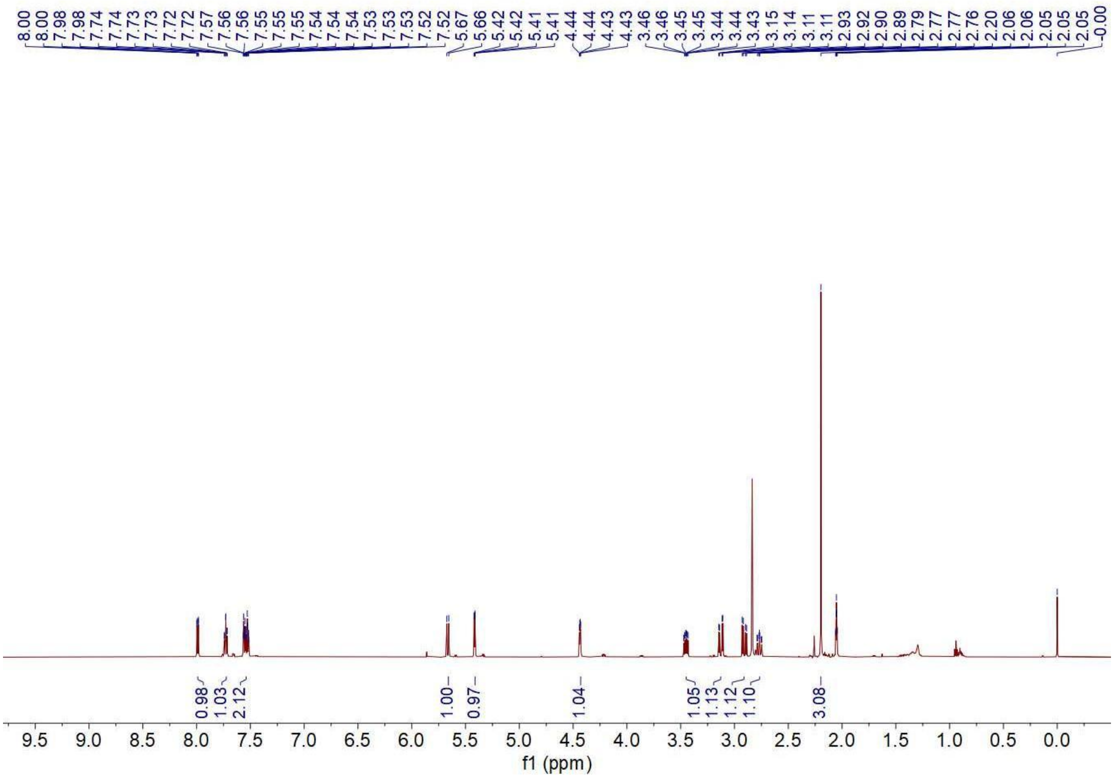
8.00 8.00 7.98 7.74 7.74 7.73 7.73 7.72 7.72 7.57 7.56 7.56 7.55 7.55 7.54 7.54 7.53 7.53 7.53 7.52 7.52 5.67 5.66 5.42 5.42 5.41 5.41 4.44 4.44 4.43 4.43 3.46 3.46 3.45 3.45 3.44 3.44 3.43 3.15 3.14 3.11 3.11 2.93 2.92 2.90 2.89 2.79 2.77 2.76 2.20 2.06 2.06 2.05 2.05 2.05 -0.00
0.98 1.03 2.12 1.00 0.97 1.04 1.05 1.13 1.12 1.10 3.08
9.5 9.0 8.5 8.0 7.5 7.0 6.5 6.0 5.5 5.0 4.5 4.0 3.5 3.0 2.5 2.0 1.5 1.0 0.5 0.0
f1 (ppm)

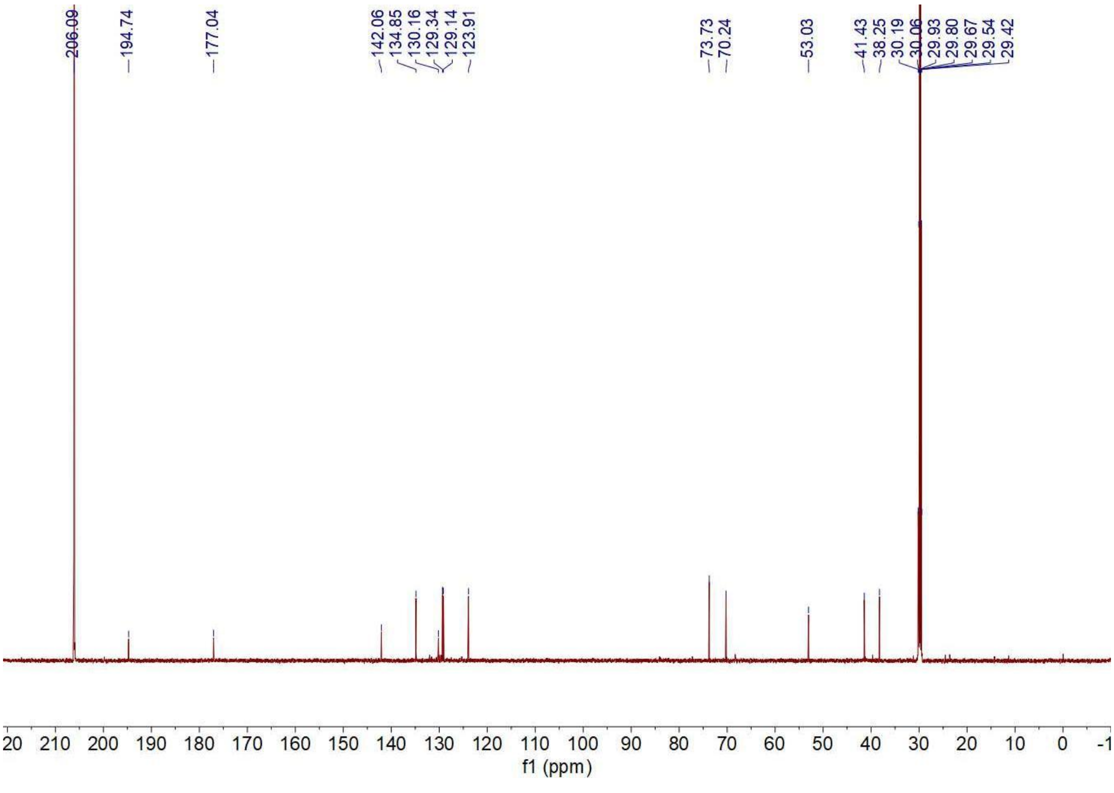
206.09 194.74 177.04 142.06 134.85 130.16 129.34 129.14 123.91 73.73 70.24 53.03 41.43 38.25 30.19 30.06 29.93 29.80 29.67 29.54 29.42
20 210 200 190 180 170 160 150 140 130 120 110 100 90 80 70 60 50 40 30 20 10 0 -1
f1 (ppm)

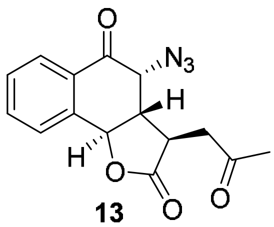
O
N3
H
H
O
O
O

13

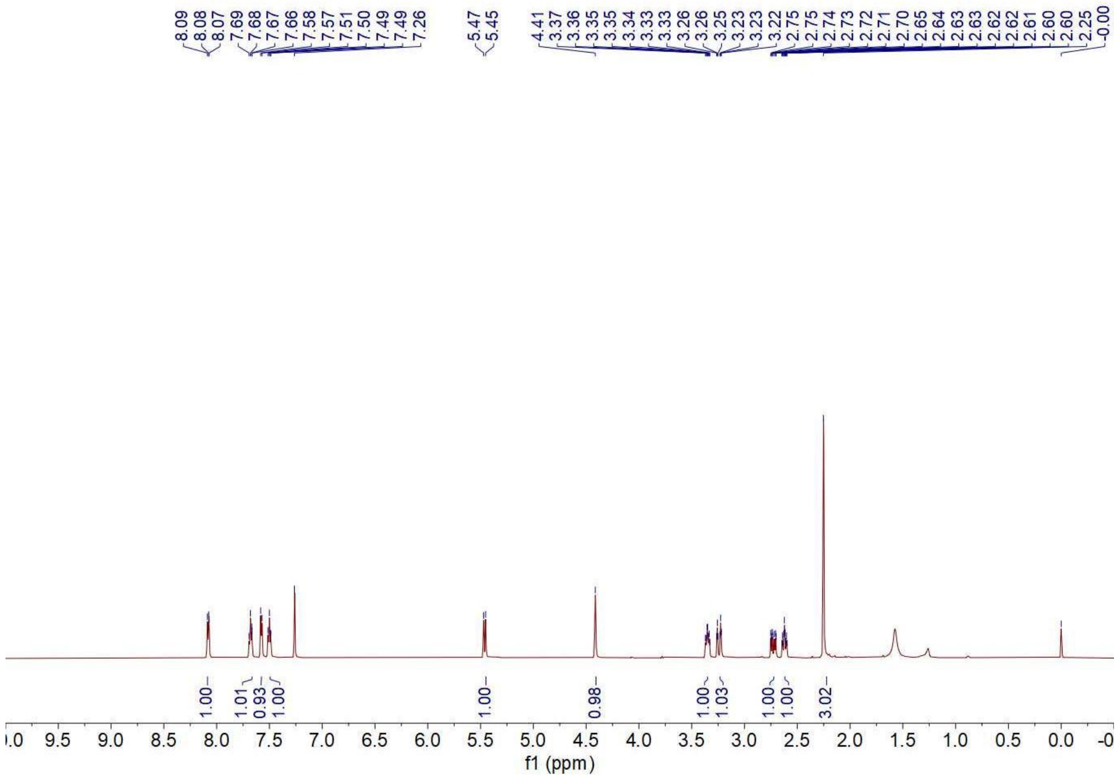
8.09
8.08
8.07
7.69
7.68
7.67
7.66
7.58
7.57
7.51
7.50
7.49
7.49
7.26
5.47
5.45
4.41
3.37
3.36
3.35
3.35
3.34
3.33
3.33
3.26
3.26
3.25
3.23
3.23
3.22
2.75
2.75
2.74
2.73
2.72
2.71
2.70
2.65
2.64
2.63
2.63
2.62
2.62
2.61
2.60
2.60
2.25
-0.00
1.00
1.01
0.93
1.00
1.00
0.98
1.00
1.03
1.00
1.00
3.02
f1 (ppm)

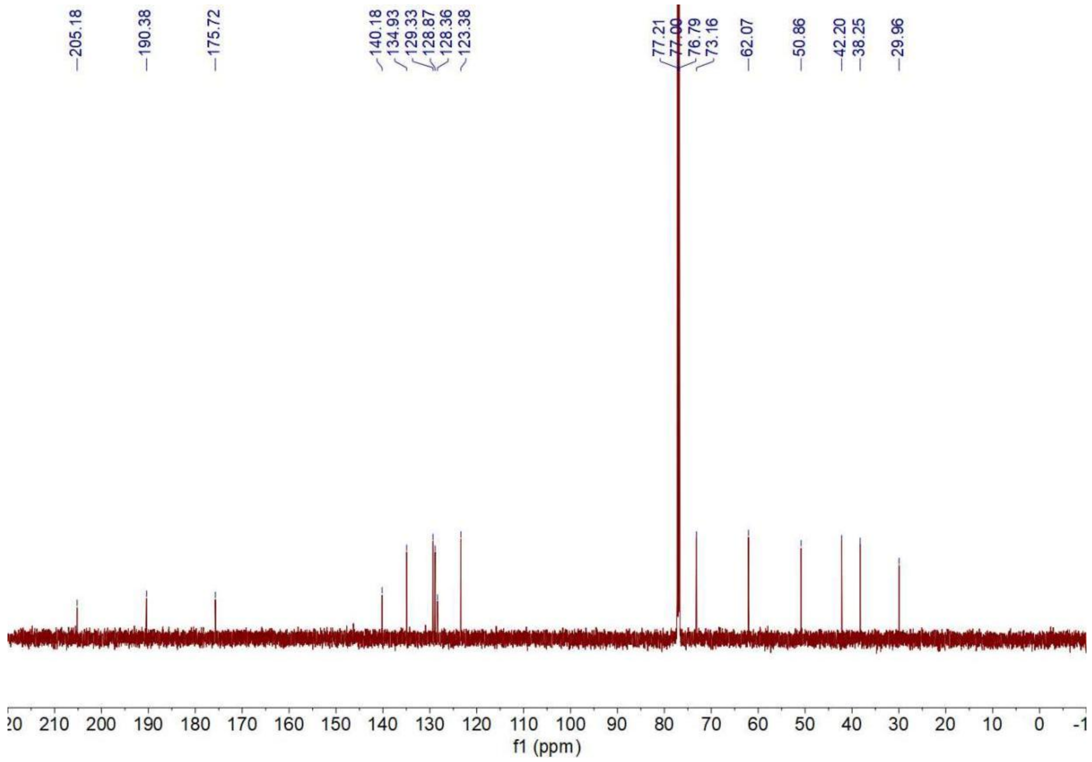
205.18
190.38
175.72
140.18
134.93
129.33
128.87
128.36
123.38
77.21
76.79
73.16
62.07
50.86
42.20
38.25
29.96
f1 (ppm)

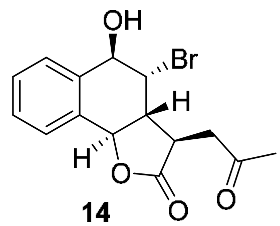
OH
Br
H
H
O
O
O

14

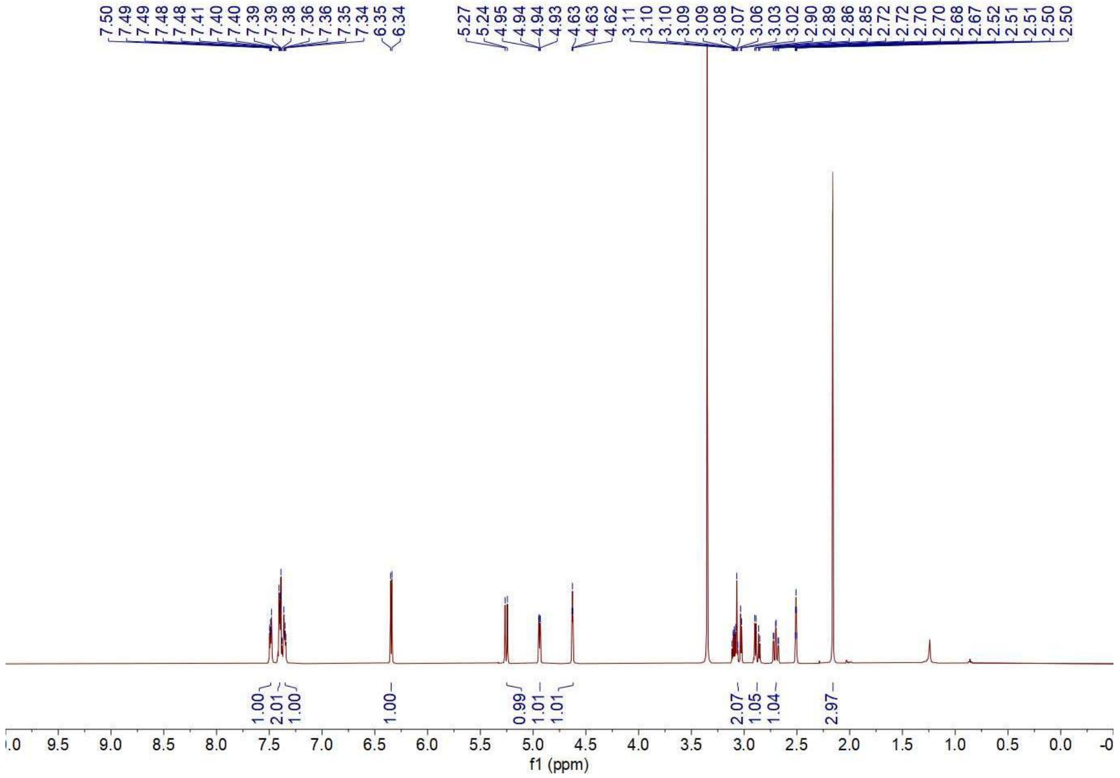
7.50 7.49 7.49 7.48 7.48 7.41 7.40 7.40 7.39 7.39 7.38 7.36 7.36 7.35 7.34 6.35 6.34
5.27 5.24 4.95 4.94 4.94 4.93 4.63 4.63 4.62 3.11 3.10 3.10 3.09 3.09 3.08 3.07 3.06 3.03 3.02 2.90 2.89 2.86 2.85 2.72 2.72 2.70 2.70 2.68 2.67 2.52 2.51 2.51 2.50 2.50
1.00 2.01 1.00 1.00 0.99 1.01 1.01 2.07 1.05 1.04 2.97
f1 (ppm)

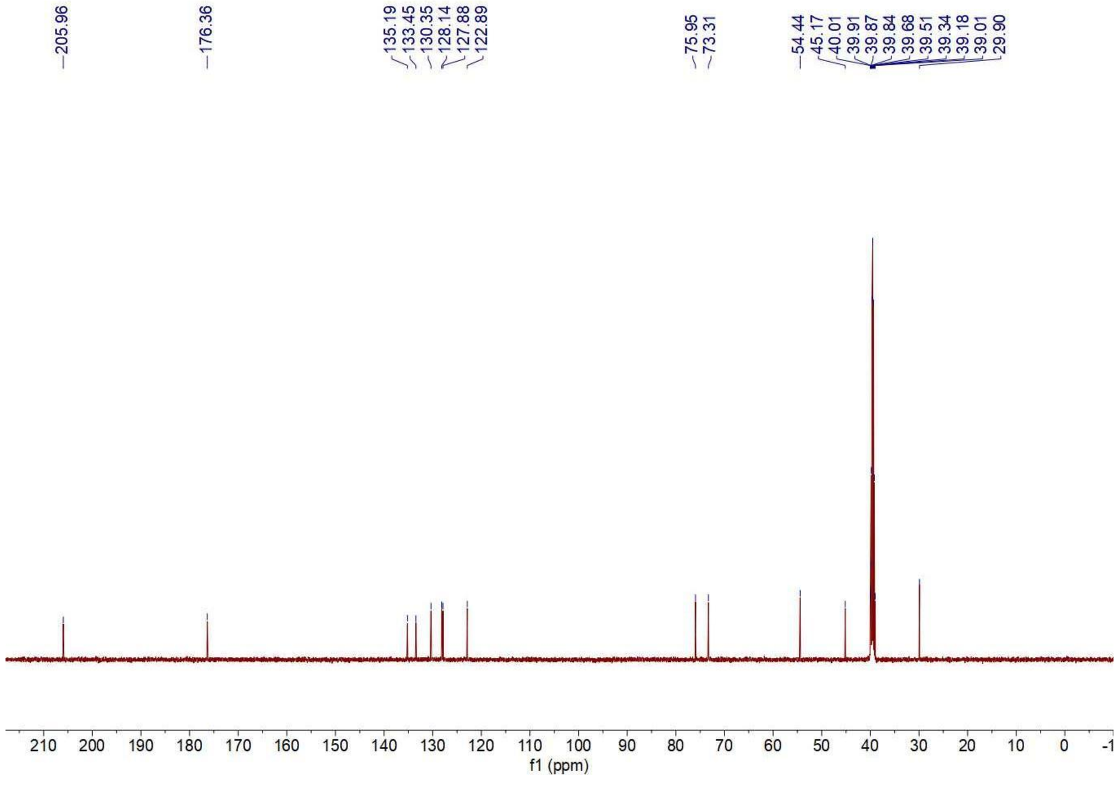
205.96
176.36
135.19 133.45 130.35 128.14 127.88 122.89
75.95 73.31
54.44 45.17 40.01 39.91 39.87 39.84 39.68 39.51 39.34 39.18 39.01 29.90
f1 (ppm)

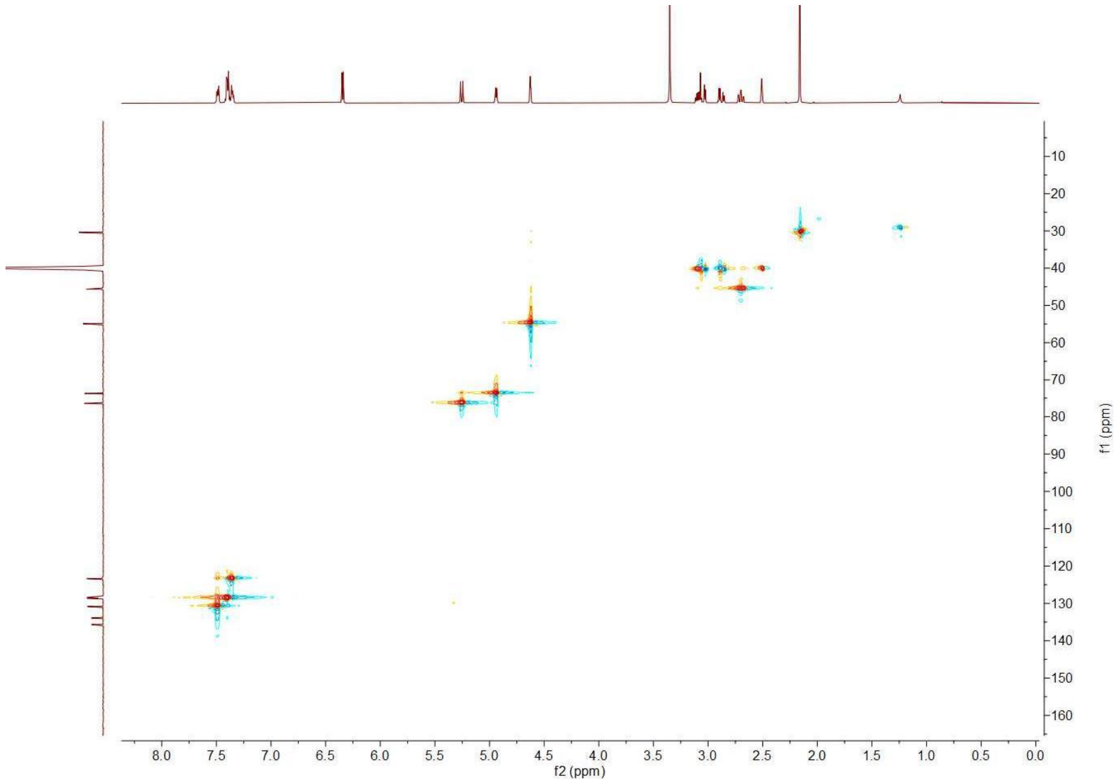

HSQC (500 MHz, DMSO-*d*6)

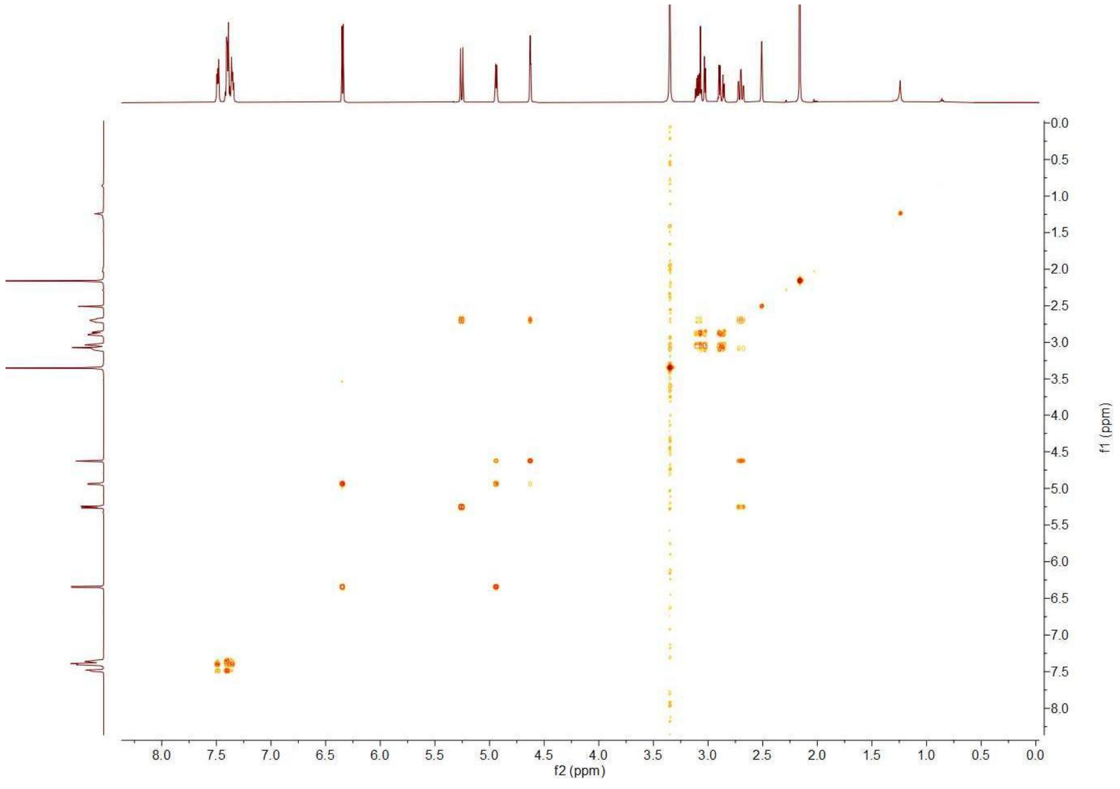

COSY (500 MHz, DMSO-*d*6)

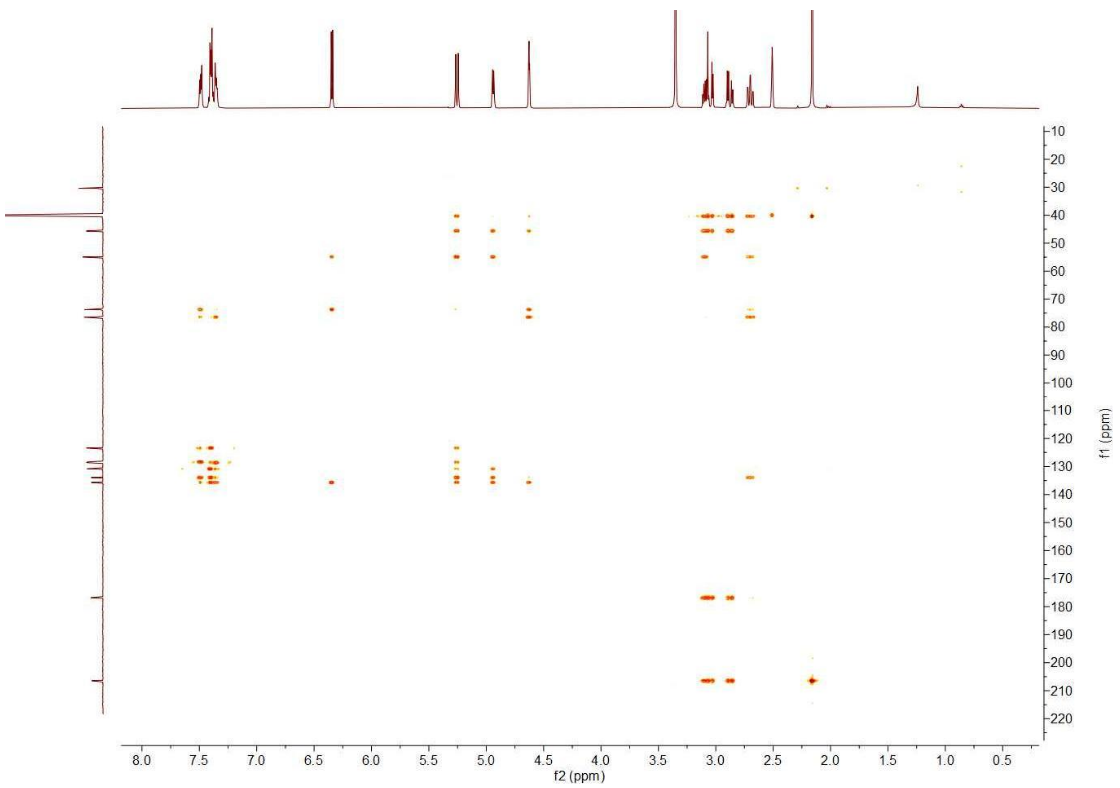

HMBC (500 MHz, DMSO-*d*6)

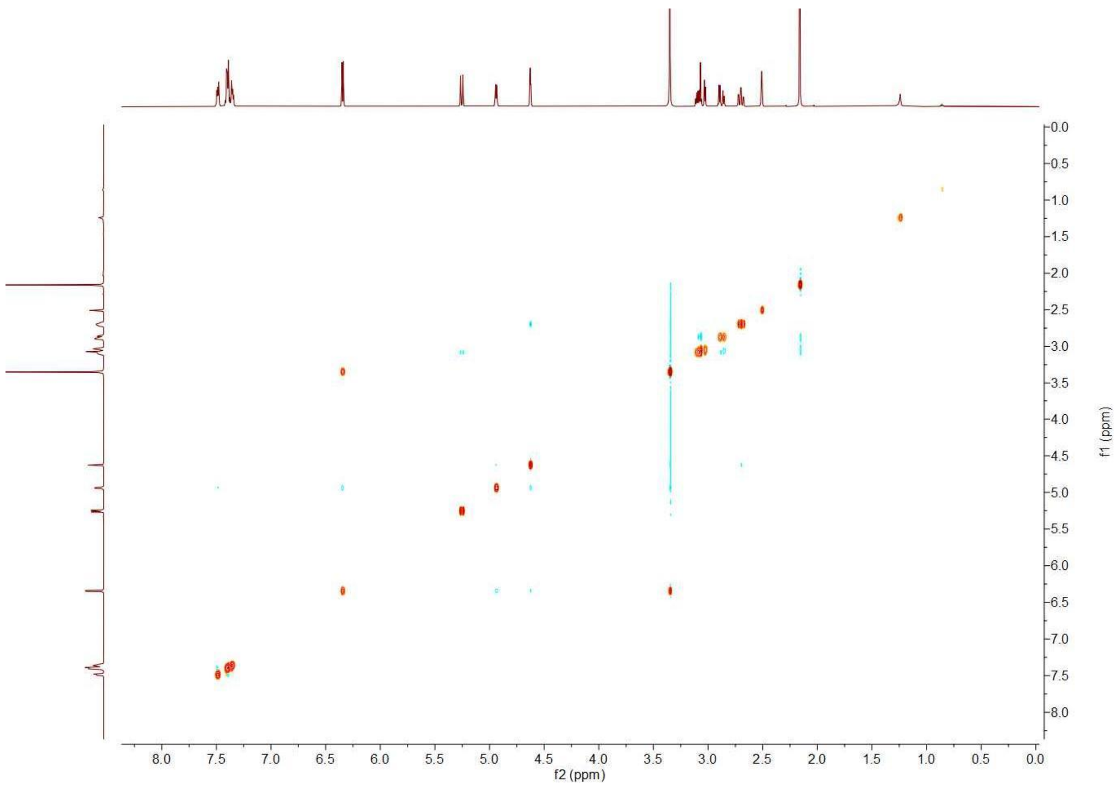

NOESY (500 MHz, DMSO-*d*6)

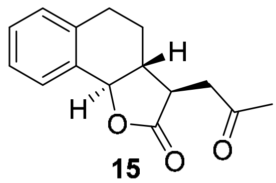

15

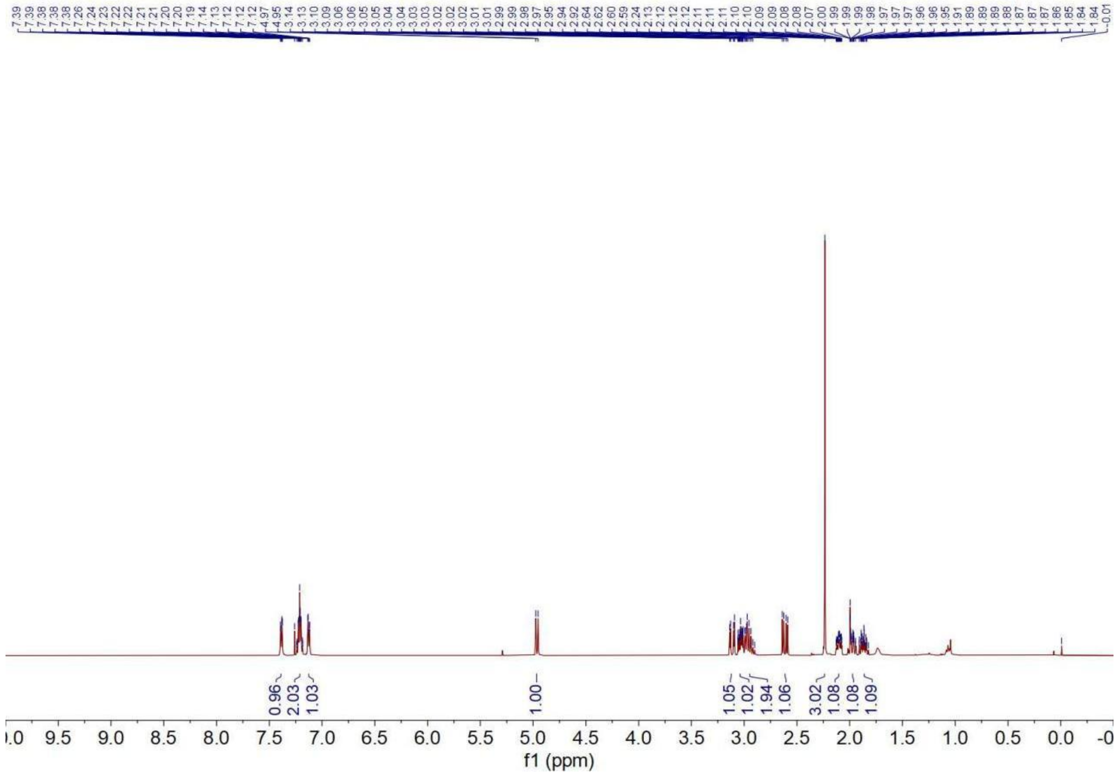
0.96
2.03
1.03
1.00
1.05
1.02
1.94
1.06
3.02
1.08
1.08
1.09
f1 (ppm)

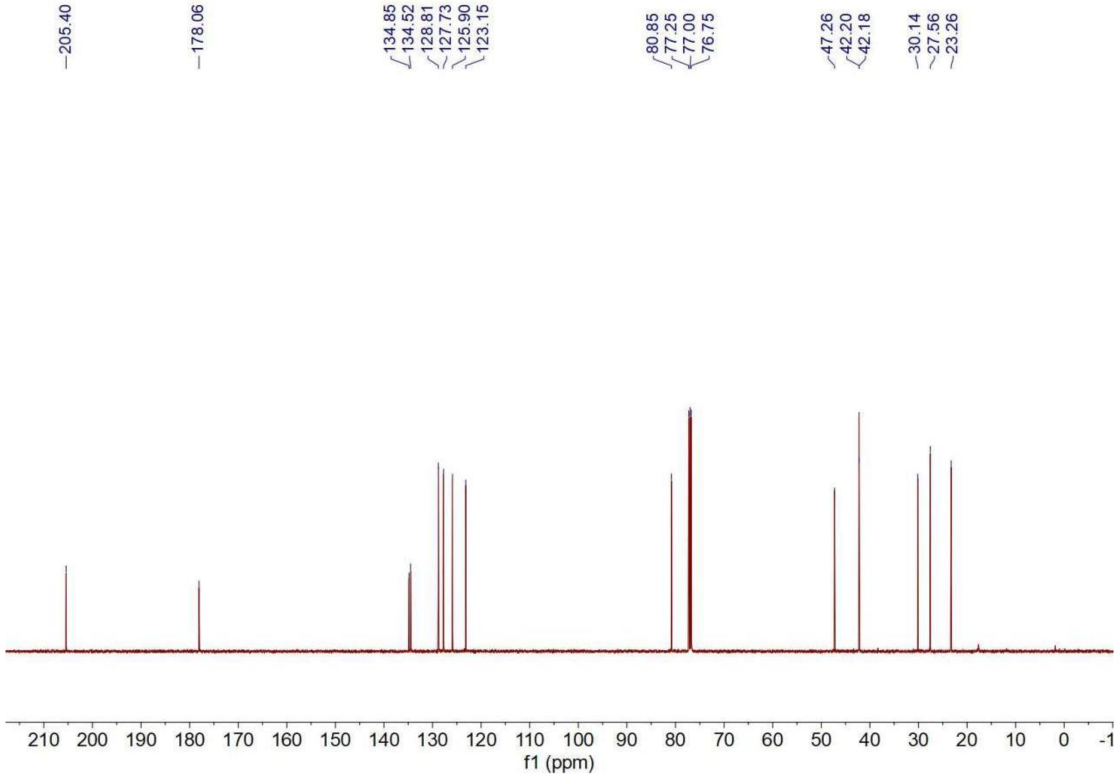
205.40
178.06
134.85
134.52
128.81
127.73
125.90
123.15
80.85
77.25
77.00
76.75
47.26
42.20
42.18
30.14
27.56
23.26
f1 (ppm)

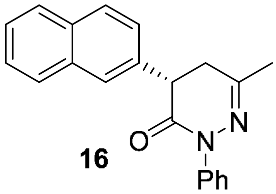
O
N
N
Ph

16

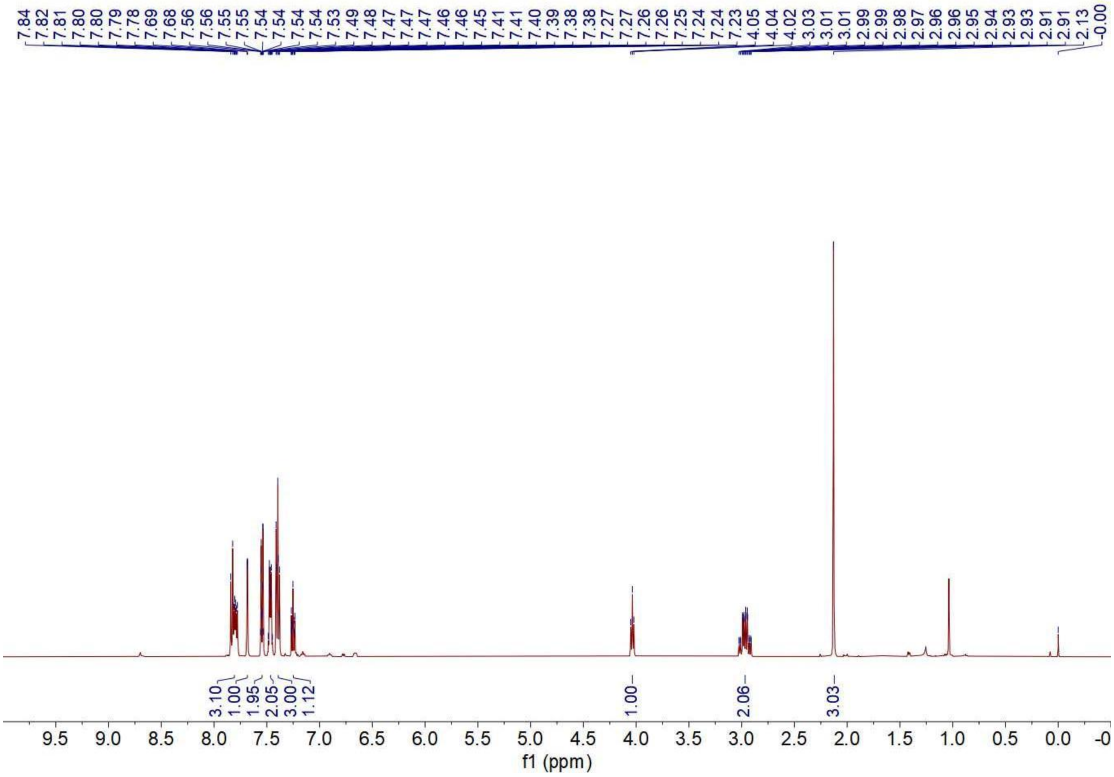
7.84 7.82 7.81 7.80 7.80 7.79 7.78 7.69 7.68 7.56 7.56 7.55 7.54 7.54 7.54 7.54 7.53 7.49 7.48 7.47 7.47 7.47 7.46 7.46 7.45 7.41 7.41 7.40 7.39 7.38 7.38 7.27 7.26 7.25 7.24 7.24 7.23 4.05 4.04 4.02 3.03 3.01 2.99 2.99 2.98 2.97 2.96 2.96 2.95 2.94 2.93 2.93 2.91 2.91 2.13 -0.00
3.10
1.00
1.95
2.05
3.00
1.12
1.00
2.06
3.03
9.5 9.0 8.5 8.0 7.5 7.0 6.5 6.0 5.5 5.0 4.5 4.0 3.5 3.0 2.5 2.0 1.5 1.0 0.5 0.0 -0
f1 (ppm)

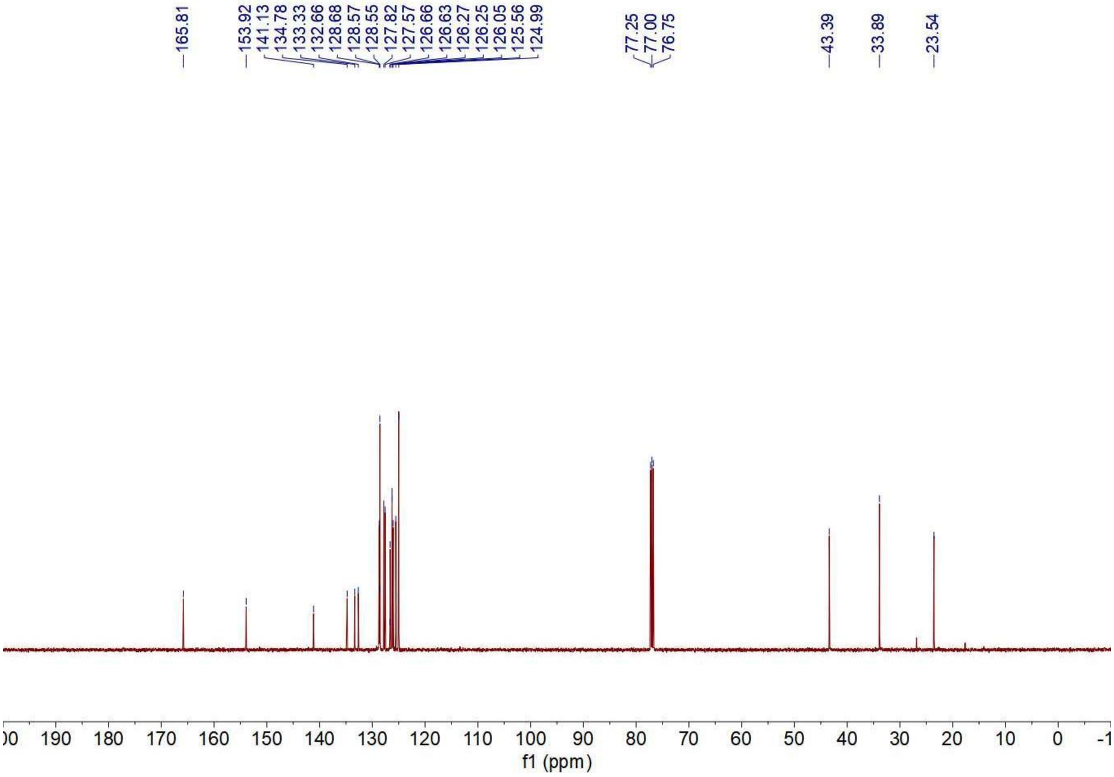
165.81
153.92 141.13 134.78 133.33 132.66 128.68 128.57 128.55 127.82 127.57 126.66 126.63 126.27 126.25 126.05 125.56 124.99
77.25 77.00 76.75
43.39
33.89
23.54
190 180 170 160 150 140 130 120 110 100 90 80 70 60 50 40 30 20 10 0 -1
f1 (ppm)

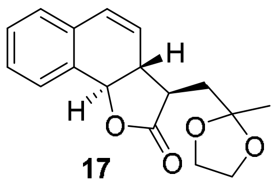

17

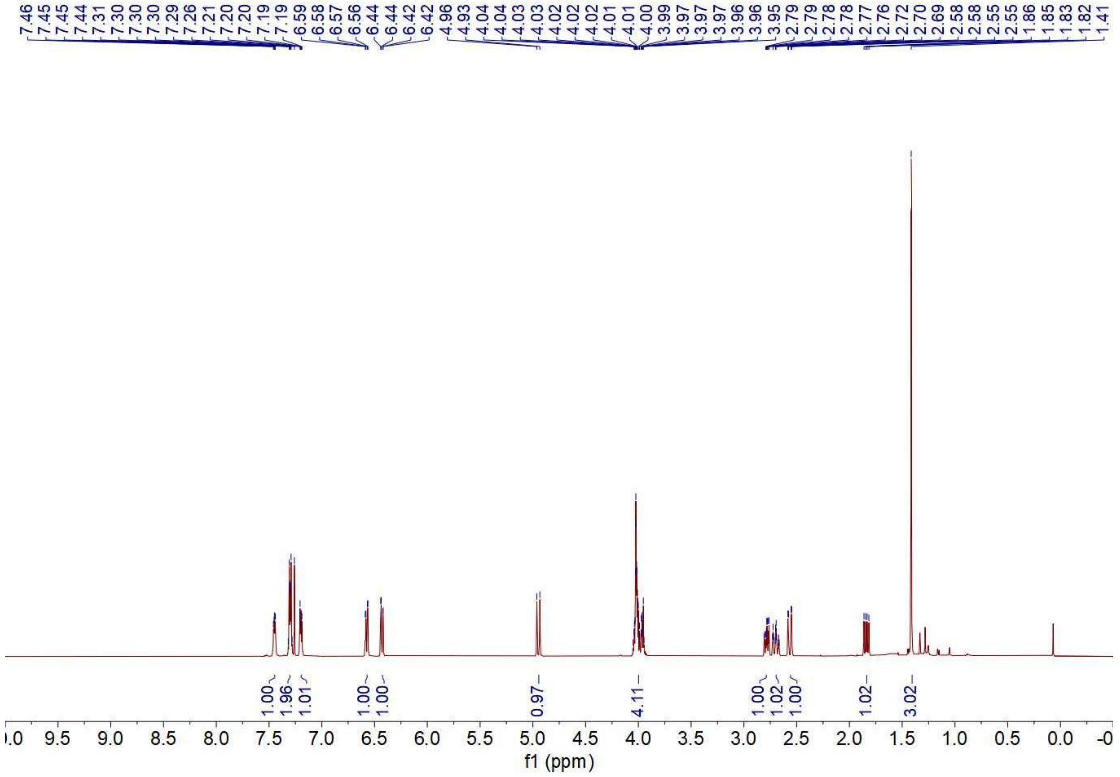
7.46
7.45
7.44
7.31
7.30
7.30
7.30
7.29
7.26
7.21
7.20
7.20
7.19
7.19
6.59
6.58
6.57
6.56
6.44
6.44
6.42
6.42
4.96
4.93
4.04
4.04
4.03
4.03
4.02
4.02
4.02
4.01
4.01
4.00
3.99
3.97
3.97
3.97
3.96
3.96
3.95
2.79
2.79
2.78
2.78
2.77
2.76
2.72
2.70
2.69
2.58
2.58
2.55
2.55
1.86
1.85
1.83
1.82
1.41
1.00
1.96
1.01
1.00
1.00
0.97
4.11
1.00
1.02
1.00
1.02
3.02
f1 (ppm)

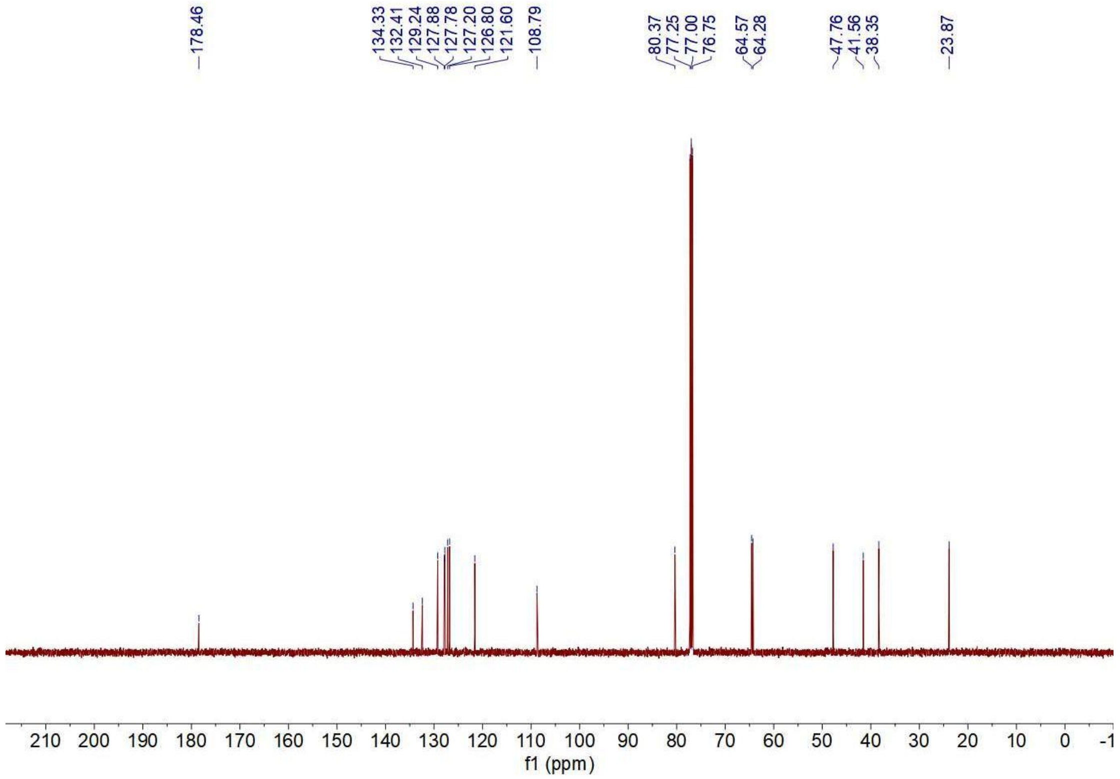
178.46
134.33
132.41
129.24
127.88
127.78
127.20
126.80
121.60
108.79
80.37
77.25
77.00
76.75
64.57
64.28
47.76
41.56
38.35
23.87
f1 (ppm)

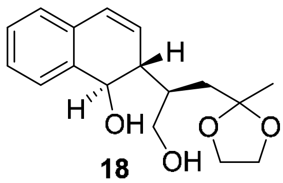
H
H
OH
O
O
OH

18

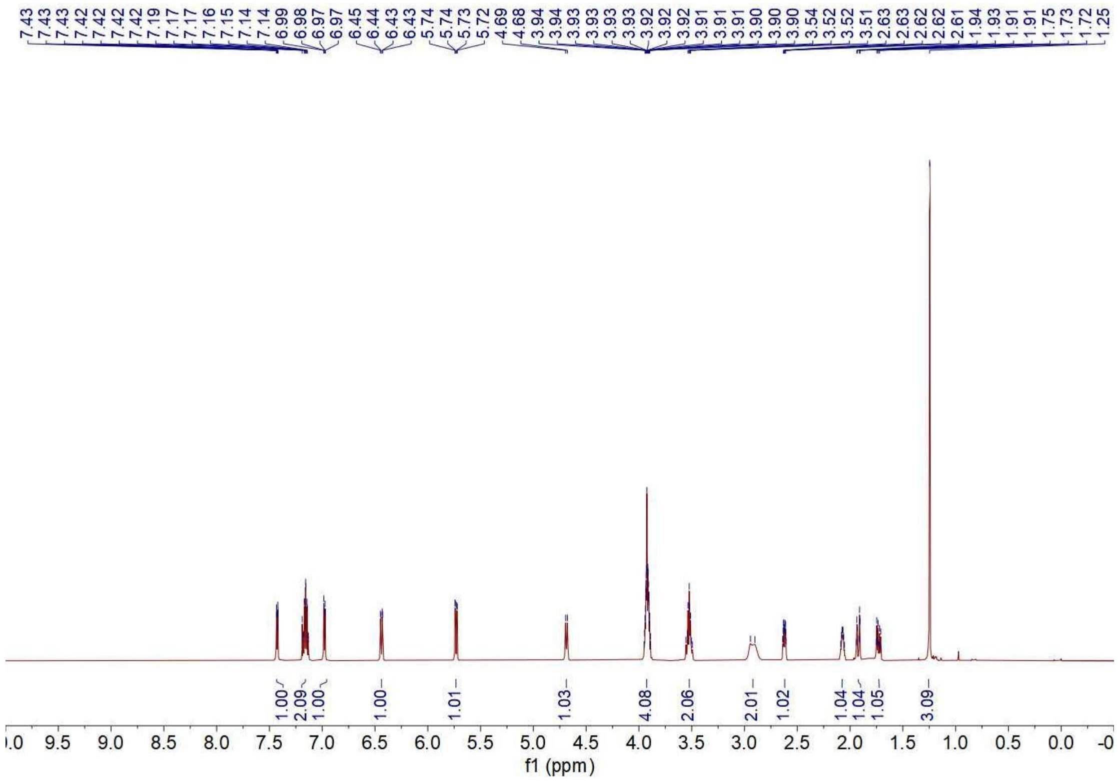
7.43
7.43
7.42
7.42
7.42
7.42
7.19
7.17
7.16
7.15
7.14
7.14
6.99
6.98
6.97
6.97
6.45
6.44
6.43
6.43
5.74
5.74
5.73
5.72
4.69
4.68
3.94
3.94
3.93
3.93
3.93
3.93
3.92
3.92
3.92
3.91
3.91
3.91
3.90
3.90
3.90
3.54
3.52
3.52
3.51
2.63
2.63
2.62
2.62
2.61
1.94
1.93
1.91
1.91
1.75
1.73
1.72
1.25
1.00
2.09
1.00
1.00
1.01
1.03
4.08
2.06
2.01
1.02
1.04
1.04
1.05
3.09
f1 (ppm)

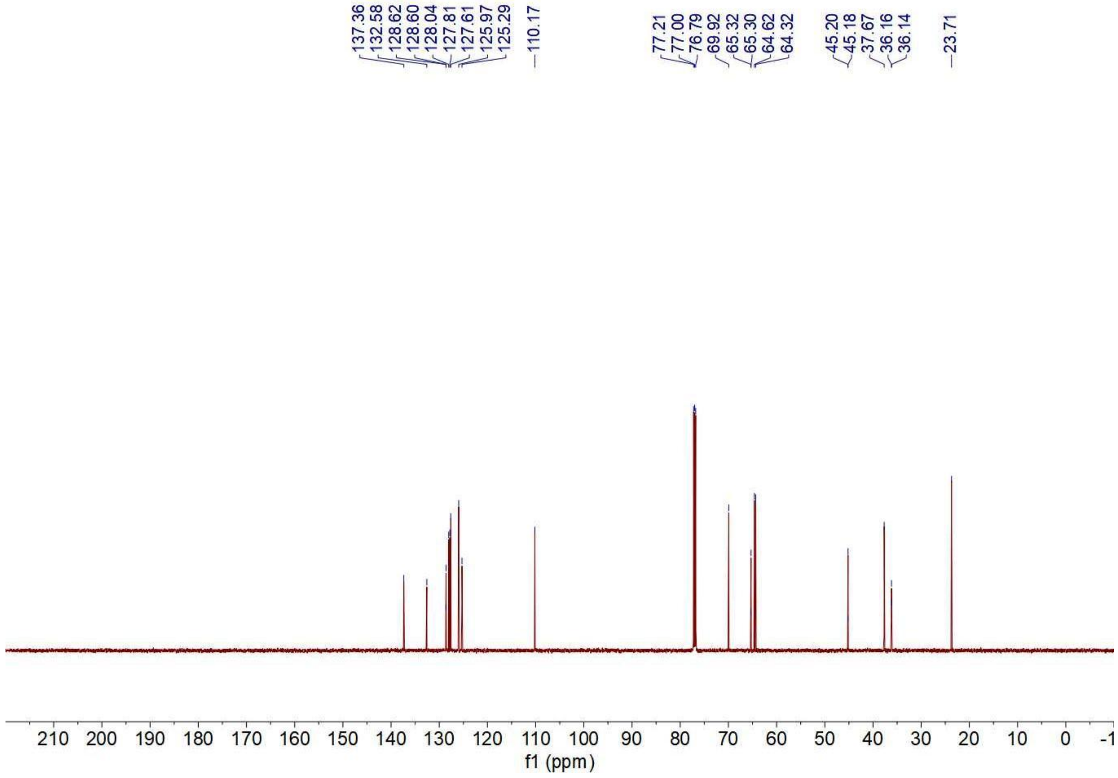
137.36
132.58
128.62
128.60
128.04
127.81
127.61
125.97
125.29
110.17
77.21
77.00
76.79
69.92
65.32
65.30
64.62
64.32
45.20
45.18
37.67
36.16
36.14
23.71
f1 (ppm)

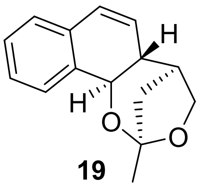
H
H
O
O

19

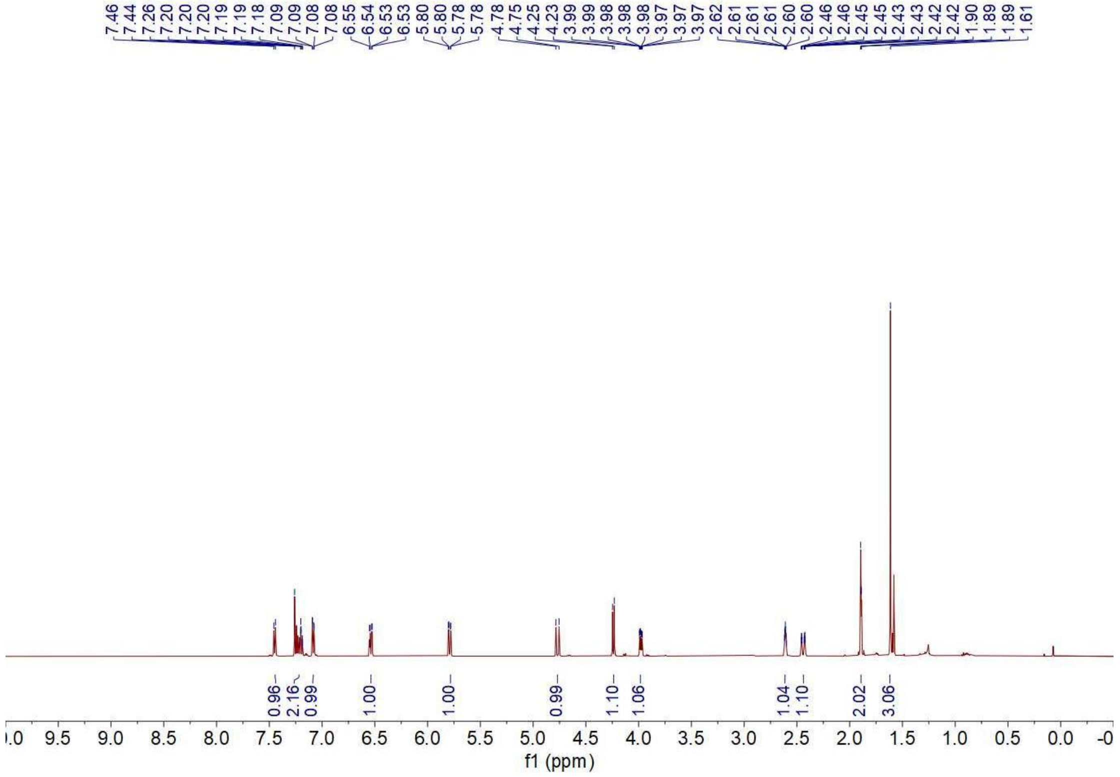
7.46
7.44
7.26
7.20
7.20
7.20
7.19
7.18
7.09
7.09
7.08
6.55
6.54
6.53
6.53
5.80
5.80
5.78
5.78
4.78
4.75
4.25
4.23
3.99
3.99
3.98
3.98
3.98
3.97
3.97
3.97
2.62
2.61
2.61
2.61
2.60
2.60
2.46
2.46
2.45
2.45
2.43
2.43
2.42
2.42
1.90
1.89
1.89
1.61
0.96
2.16
0.99
1.00
1.00
0.99
1.10
1.06
1.04
1.10
2.02
3.06
f1 (ppm)

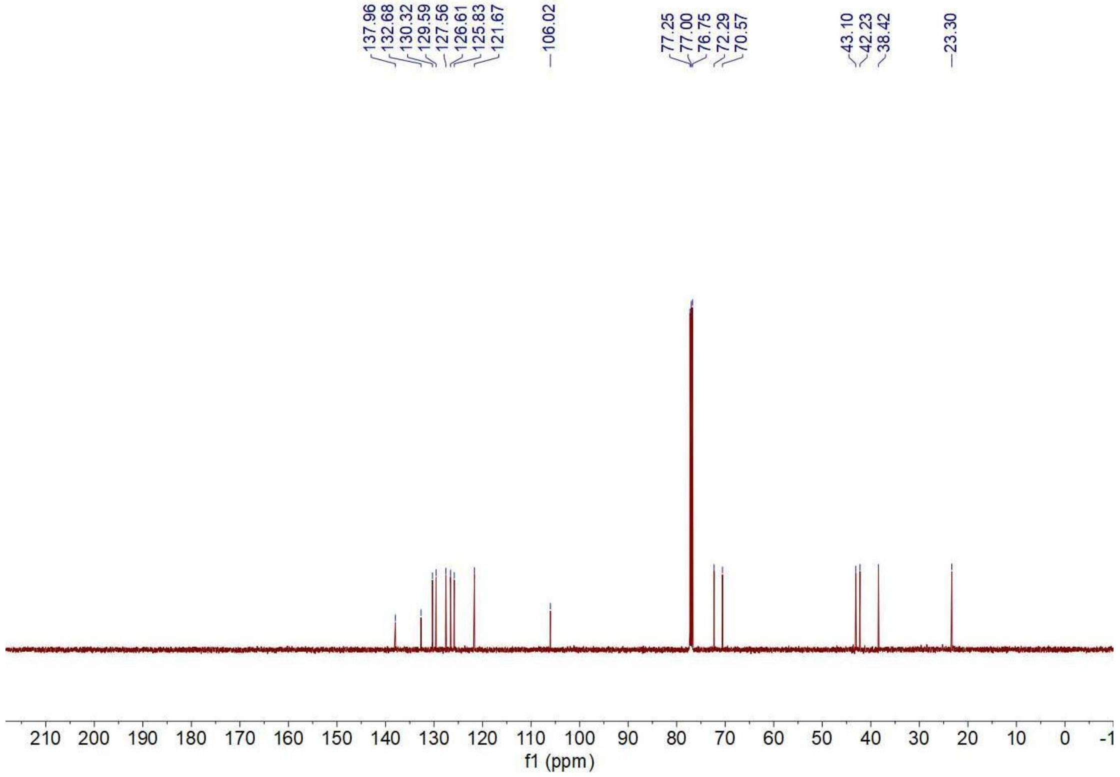
137.96
132.68
130.32
129.59
127.56
126.61
125.83
121.67
106.02
77.25
77.00
76.75
72.29
70.57
43.10
42.23
38.42
23.30
210 200 190 180 170 160 150 140 130 120 110 100 90 80 70 60 50 40 30 20 10 0
f1 (ppm)

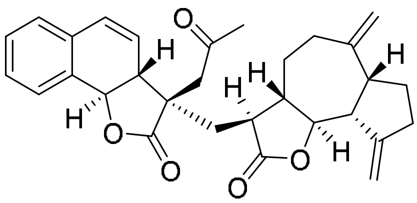

23

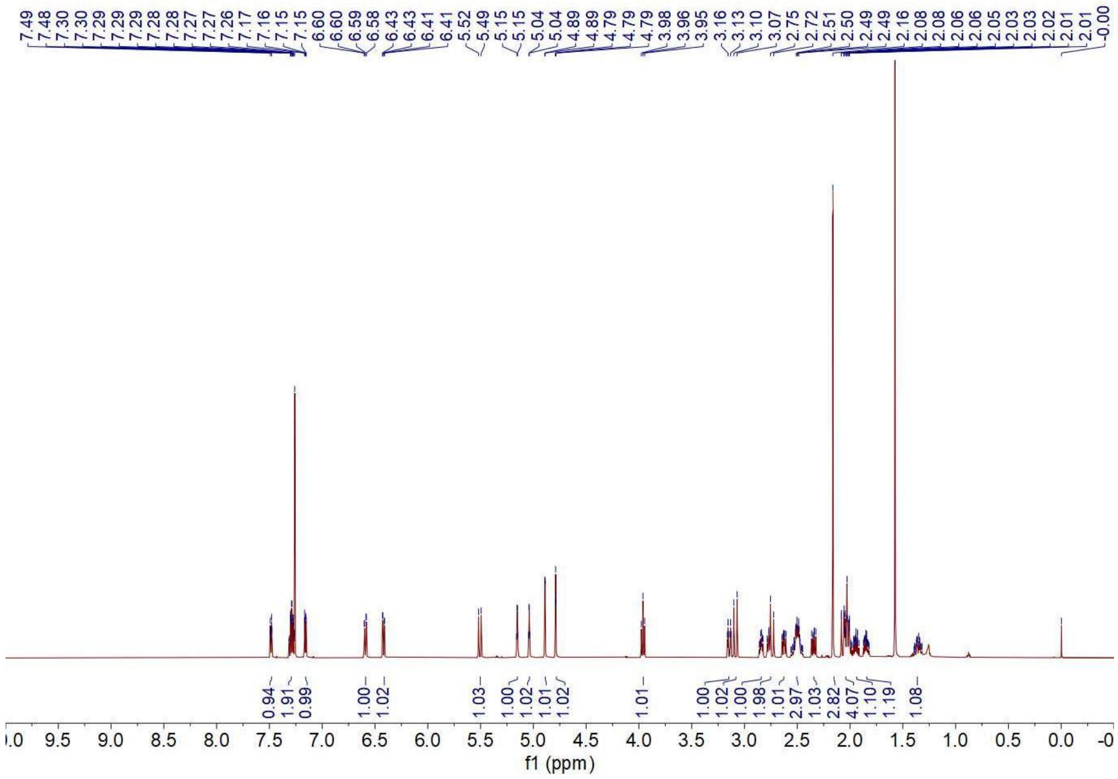
7.49 7.48 7.30 7.29 7.29 7.29 7.28 7.28 7.27 7.27 7.26 7.17 7.16 7.15 7.15 6.60 6.59 6.58 6.43 6.43 6.41 6.41 5.52 5.49 5.15 5.15 5.04 5.04 4.89 4.89 4.79 4.79 4.79 3.98 3.96 3.95 3.16 3.13 3.10 3.07 2.75 2.72 2.51 2.50 2.49 2.49 2.16 2.08 2.08 2.06 2.06 2.05 2.03 2.03 2.02 2.01 2.01 -0.00
0.94 1.91 0.99 1.00 1.02 1.03 1.00 1.02 1.01 1.02 1.01 1.00 1.02 1.00 1.98 1.01 2.97 1.03 2.82 4.07 1.10 1.19 1.08
.0 9.5 9.0 8.5 8.0 7.5 7.0 6.5 6.0 5.5 5.0 4.5 4.0 3.5 3.0 2.5 2.0 1.5 1.0 0.5 0.0 -0
f1 (ppm)

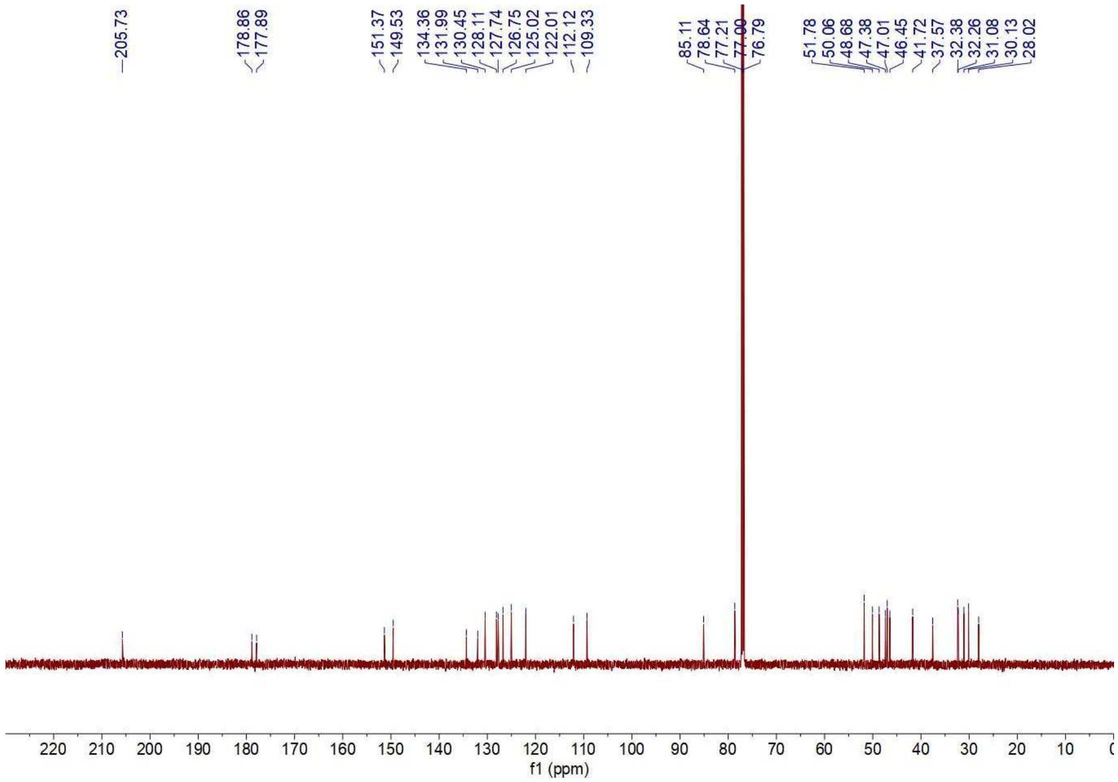
205.73 178.86 177.89 151.37 149.53 134.36 131.99 130.45 128.11 127.74 126.75 125.02 122.01 112.12 109.33 85.11 78.64 77.21 77.00 76.79 51.78 50.06 48.68 47.38 47.01 46.45 41.72 37.57 32.38 32.26 31.08 30.13 28.02
220 210 200 190 180 170 160 150 140 130 120 110 100 90 80 70 60 50 40 30 20 10 0
f1 (ppm)

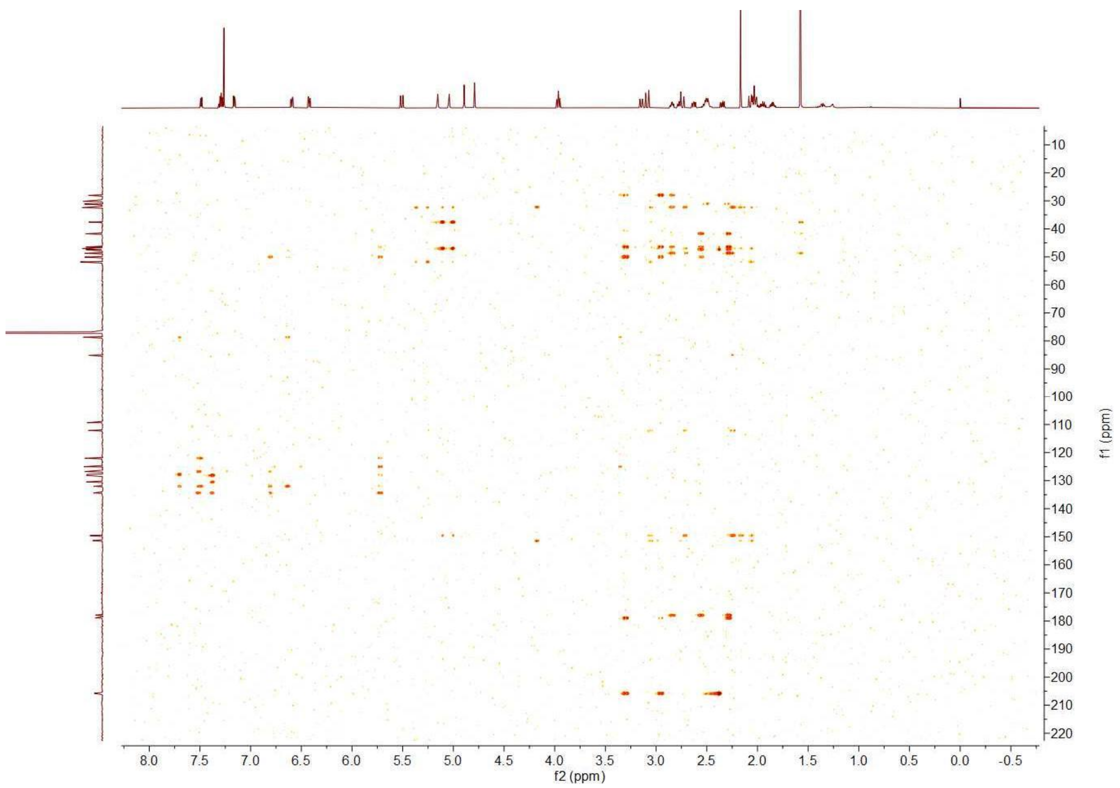

HSQC (600 MHz, $CDCl_3$)

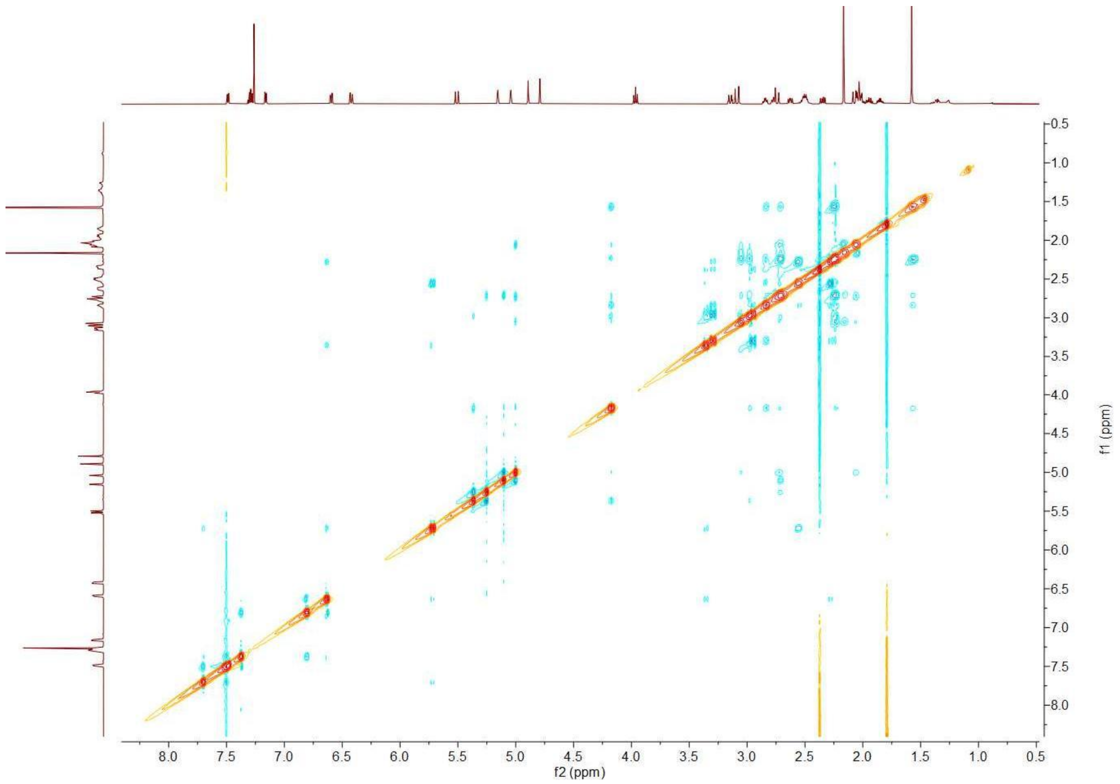

Cosy (600 MHz, $CDCl_3$)

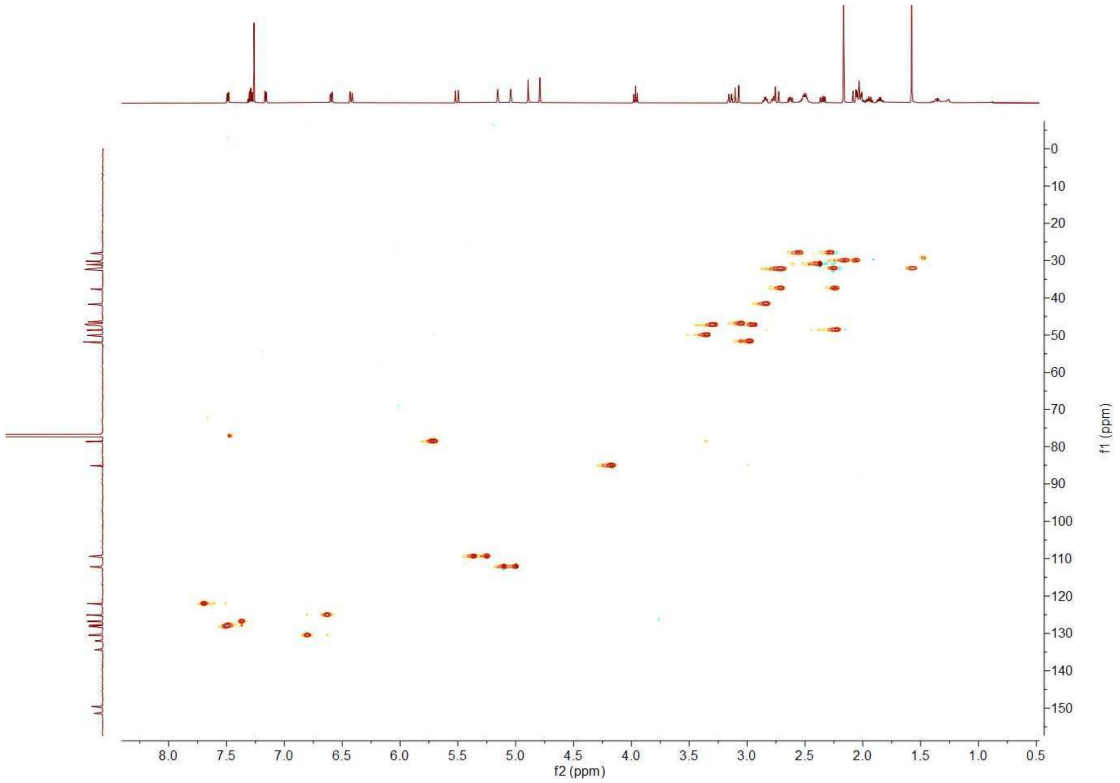

HMBC (600 MHz, $CDCl_3$)

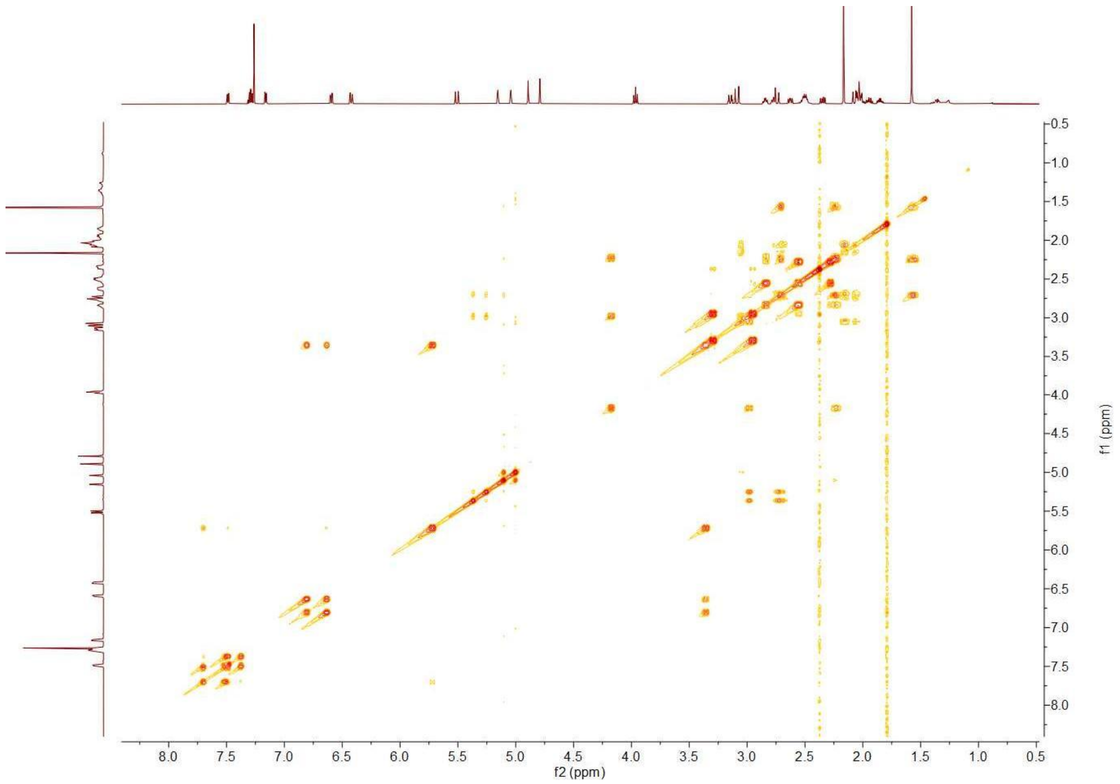

Noesy (600 MHz, $CDCl_3$)

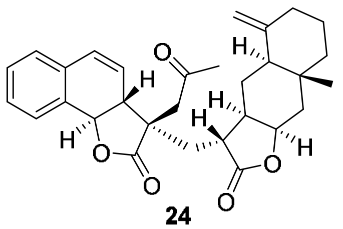

24

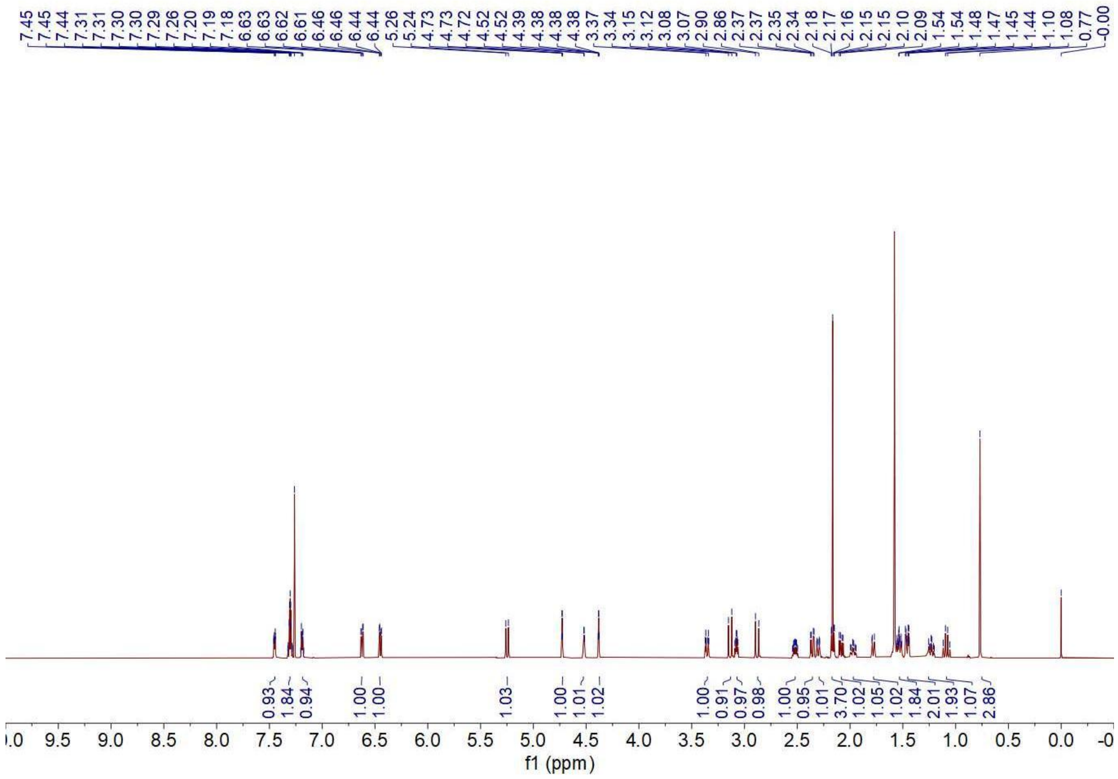
7.45 7.45 7.44 7.31 7.31 7.30 7.30 7.29 7.26 7.20 7.19 7.18 6.63 6.63 6.62 6.61 6.46 6.46 6.44 6.44 5.26 5.24 4.73 4.73 4.72 4.52 4.52 4.39 4.38 4.38 4.38 3.37 3.34 3.15 3.12 3.08 3.07 2.90 2.86 2.37 2.37 2.35 2.34 2.18 2.17 2.16 2.15 2.15 2.10 2.09 1.54 1.54 1.48 1.47 1.45 1.44 1.10 1.08 0.77 -0.00
0.93 1.84 0.94 1.00 1.00 1.03 1.00 1.01 1.02 1.00 0.91 0.97 0.98 1.00 0.95 1.01 3.70 1.02 1.05 1.02 1.84 2.01 1.93 1.07 2.86
f1 (ppm)

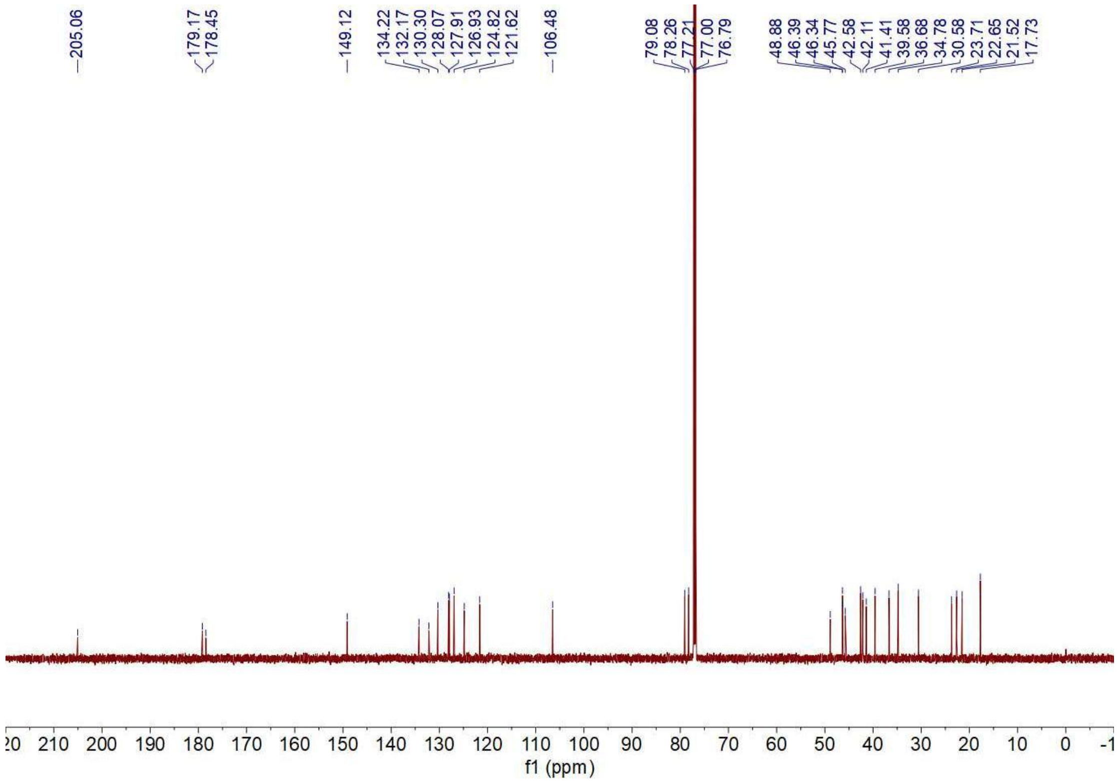
205.06 179.17 178.45 149.12 134.22 132.17 130.30 128.07 127.91 126.93 124.82 121.62 106.48 79.08 78.26 77.21 77.00 76.79 48.88 46.39 46.34 45.77 42.58 42.11 41.41 39.58 36.68 34.78 30.58 23.71 22.65 21.52 17.73
f1 (ppm)

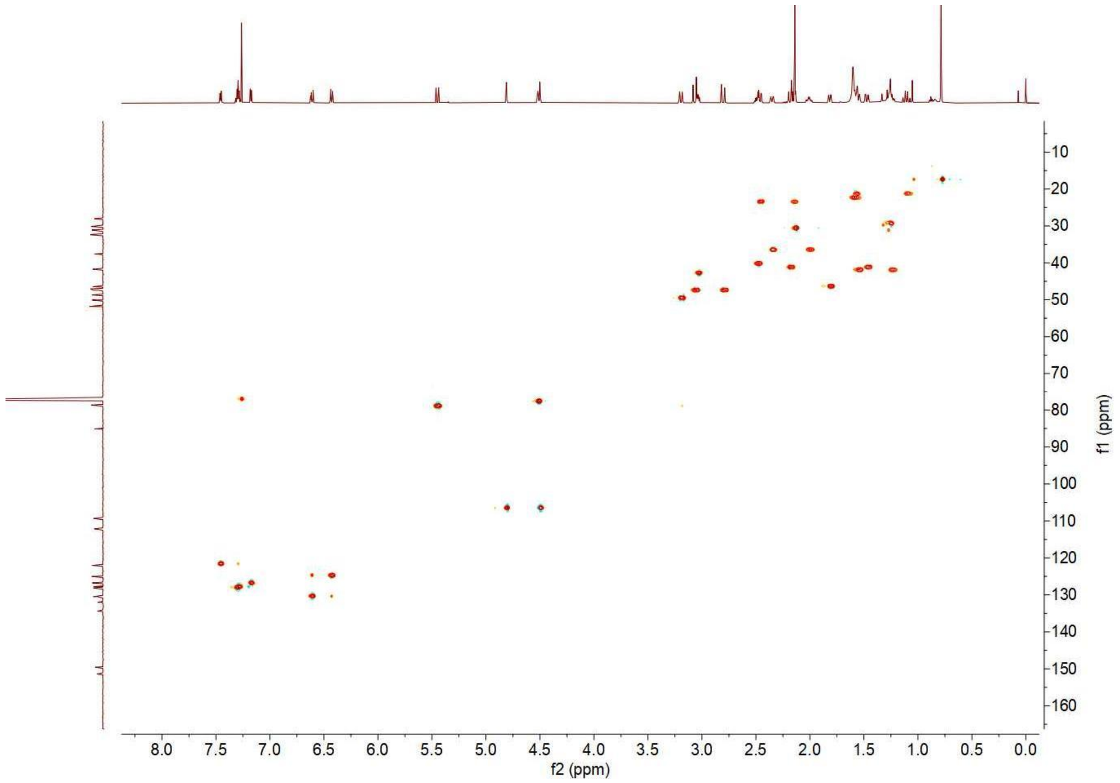

HSQC (600 MHz, $CDCl_3$)

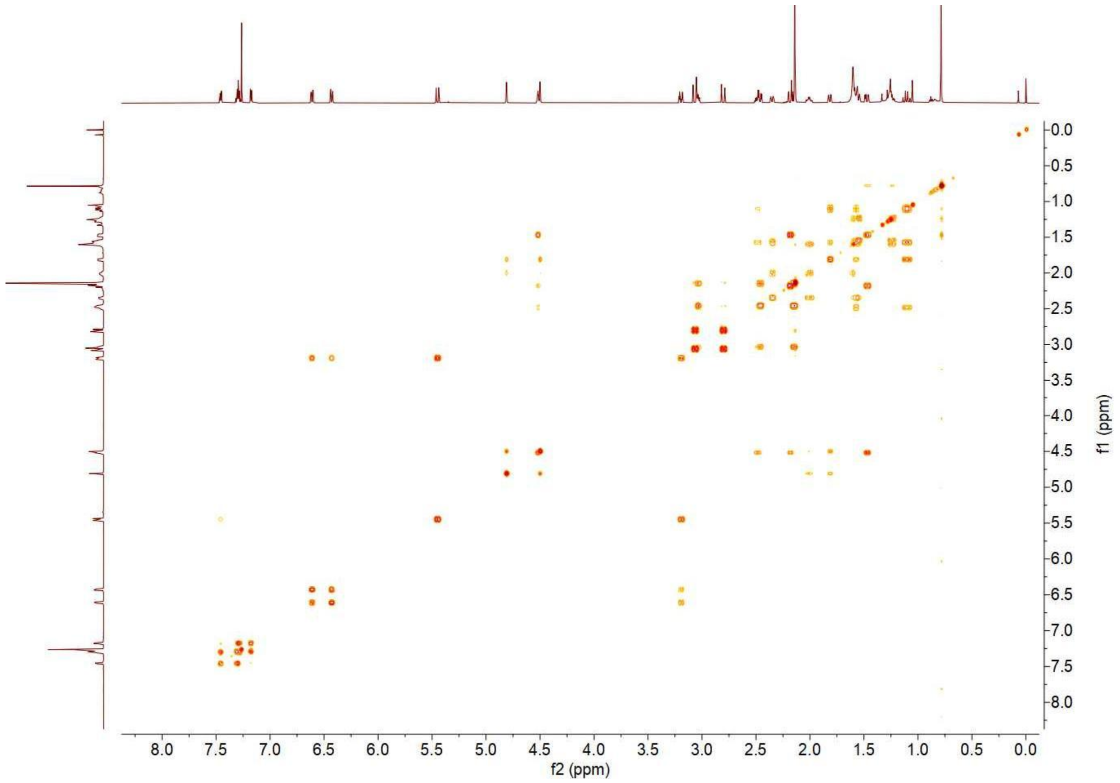

Cosy (600 MHz, $CDCl_3$)

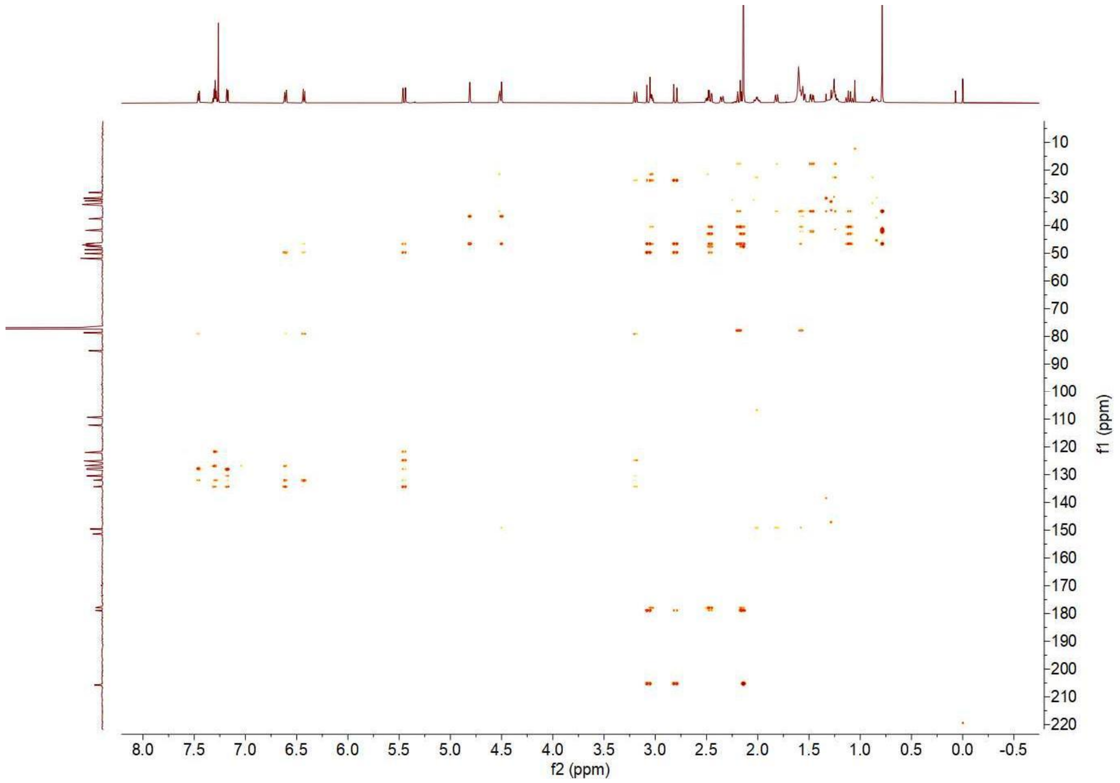

HMBC (600 MHz, $CDCl_3$)

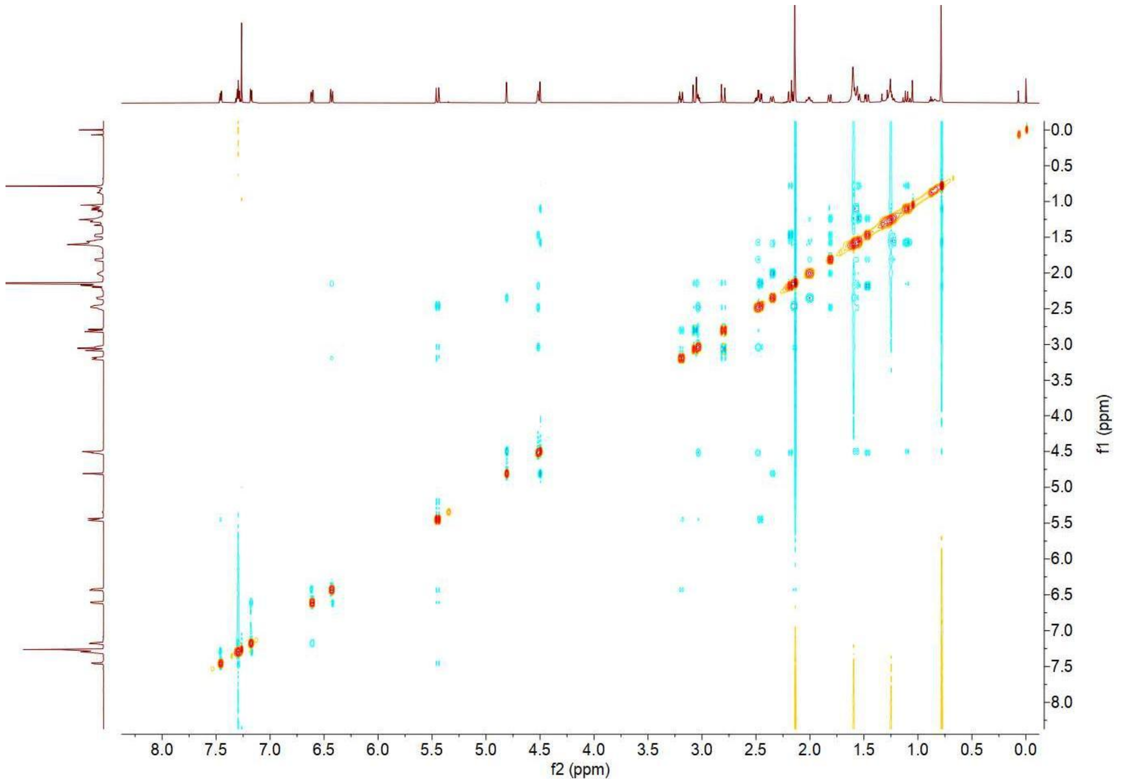

Noesy (600 MHz, $CDCl_3$)

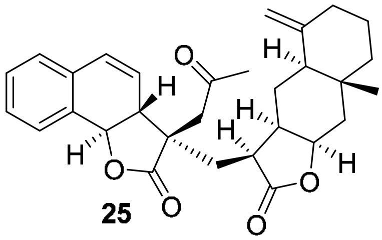

25

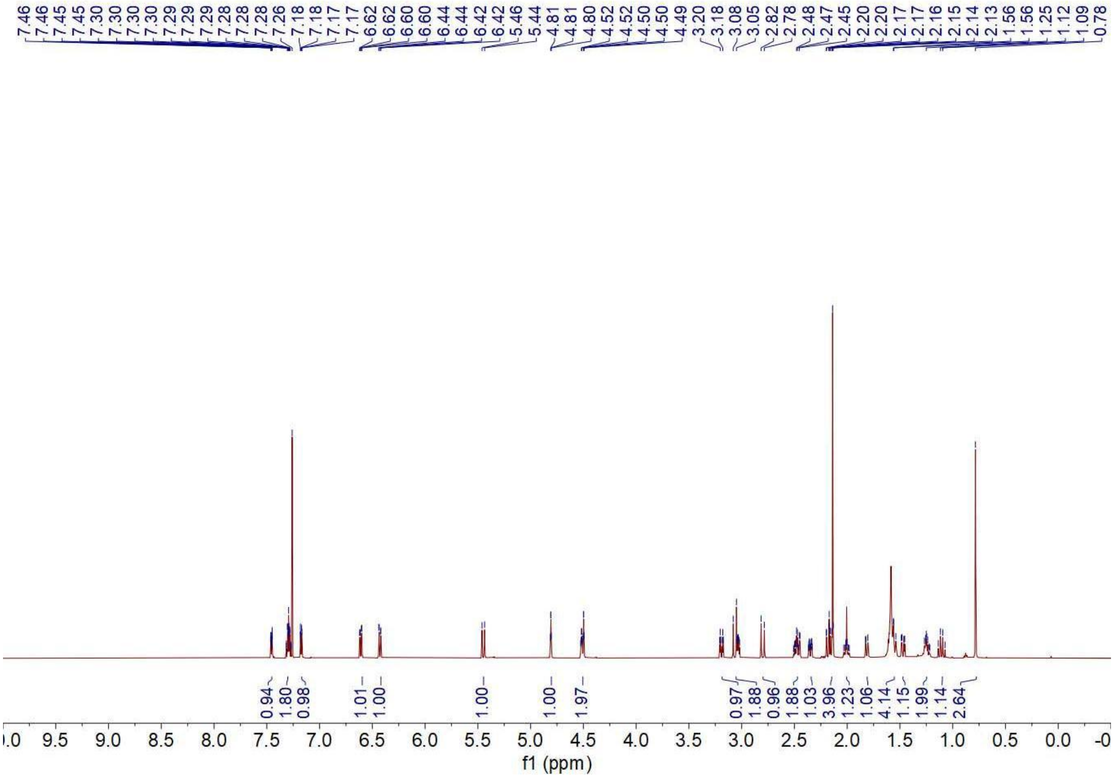
7.46 7.46 7.45 7.45 7.30 7.30 7.30 7.30 7.29 7.29 7.28 7.28 7.28 7.26 7.18 7.18 7.17 7.17 6.62 6.62 6.60 6.60 6.44 6.44 6.42 6.42 5.46 5.44 4.81 4.81 4.80 4.52 4.52 4.50 4.50 4.49 3.20 3.18 3.08 3.05 2.82 2.78 2.48 2.47 2.45 2.20 2.20 2.17 2.17 2.16 2.15 2.14 2.13 1.56 1.56 1.25 1.12 1.09 0.78
0.94 1.80 0.98 1.01 1.00 1.00 1.00 1.97 0.97 1.88 0.96 1.88 1.03 3.96 1.23 1.06 4.14 1.15 1.99 1.14 2.64
f1 (ppm)

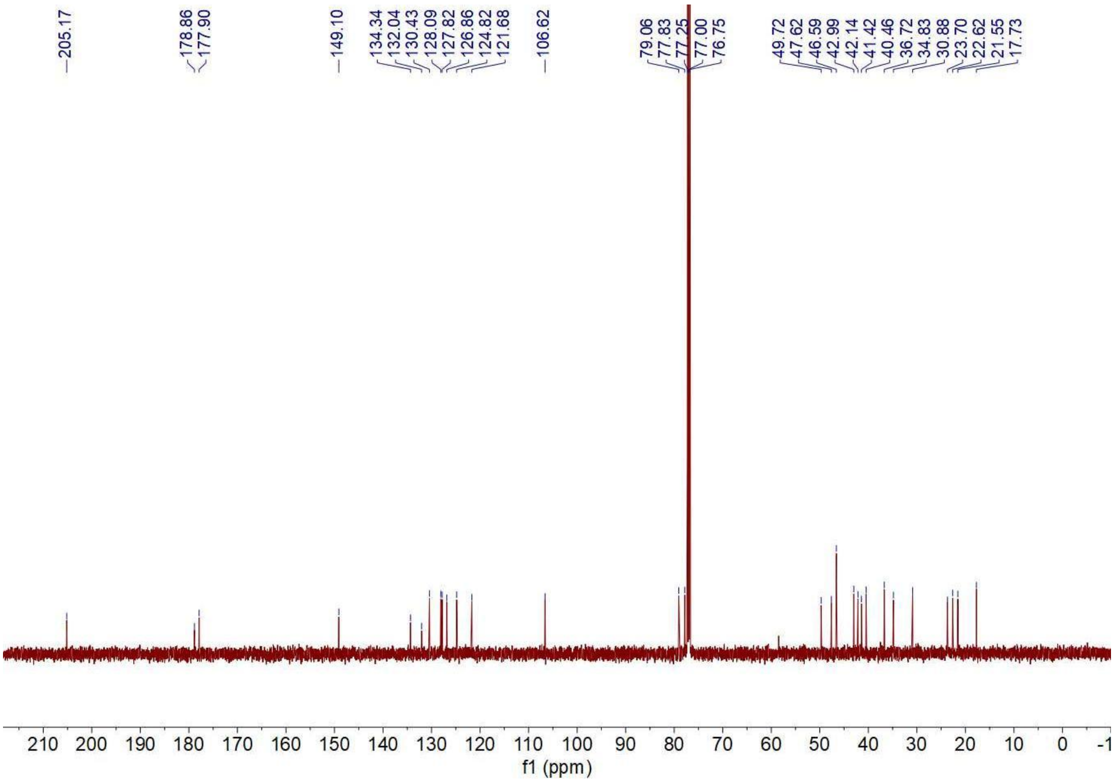
205.17 178.86 177.90 149.10 134.34 132.04 130.43 128.09 127.82 126.86 124.82 121.68 106.62 79.06 77.83 77.25 77.00 76.75 49.72 47.62 46.59 42.99 42.14 41.42 40.46 36.72 34.83 30.88 23.70 22.62 21.55 17.73
f1 (ppm)

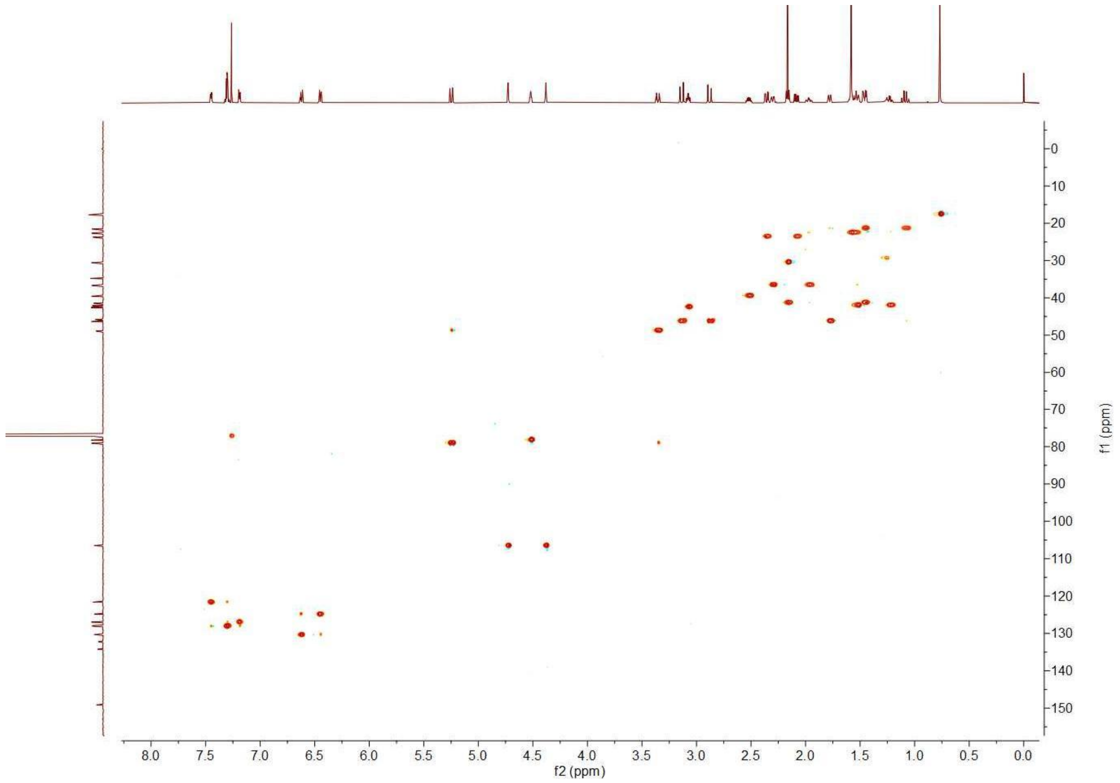

HSQC (600 MHz, $CDCl_3$)

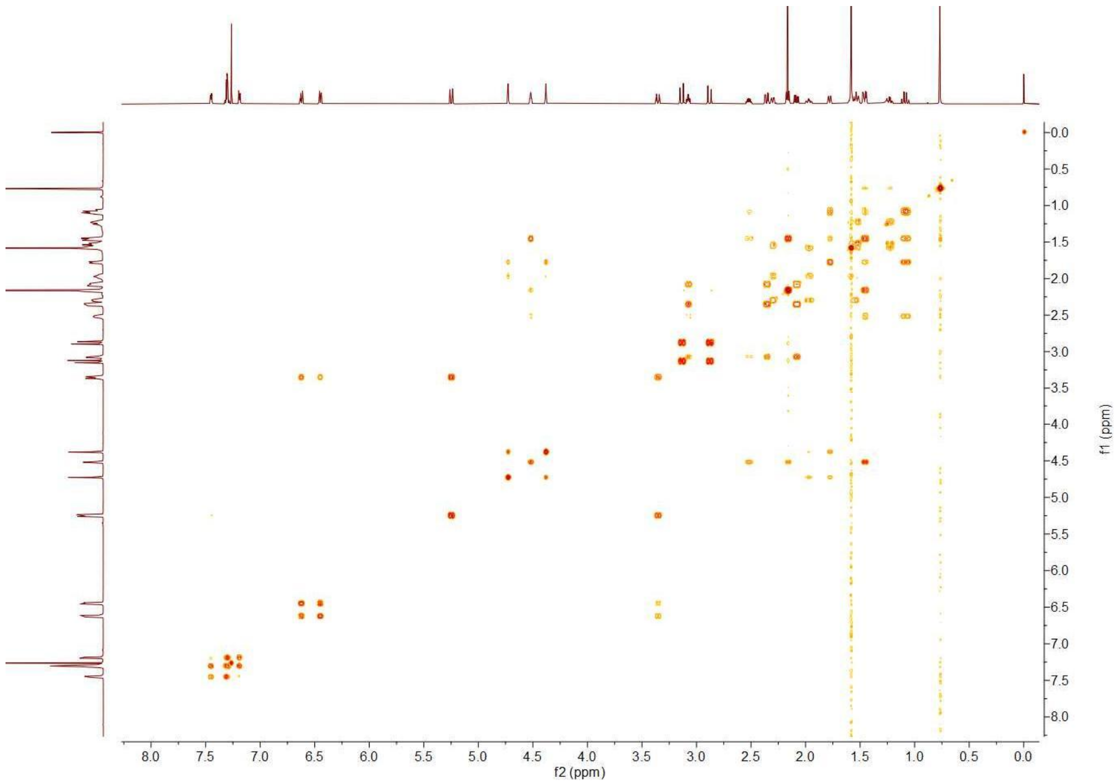

Cosy (600 MHz, $CDCl_3$)

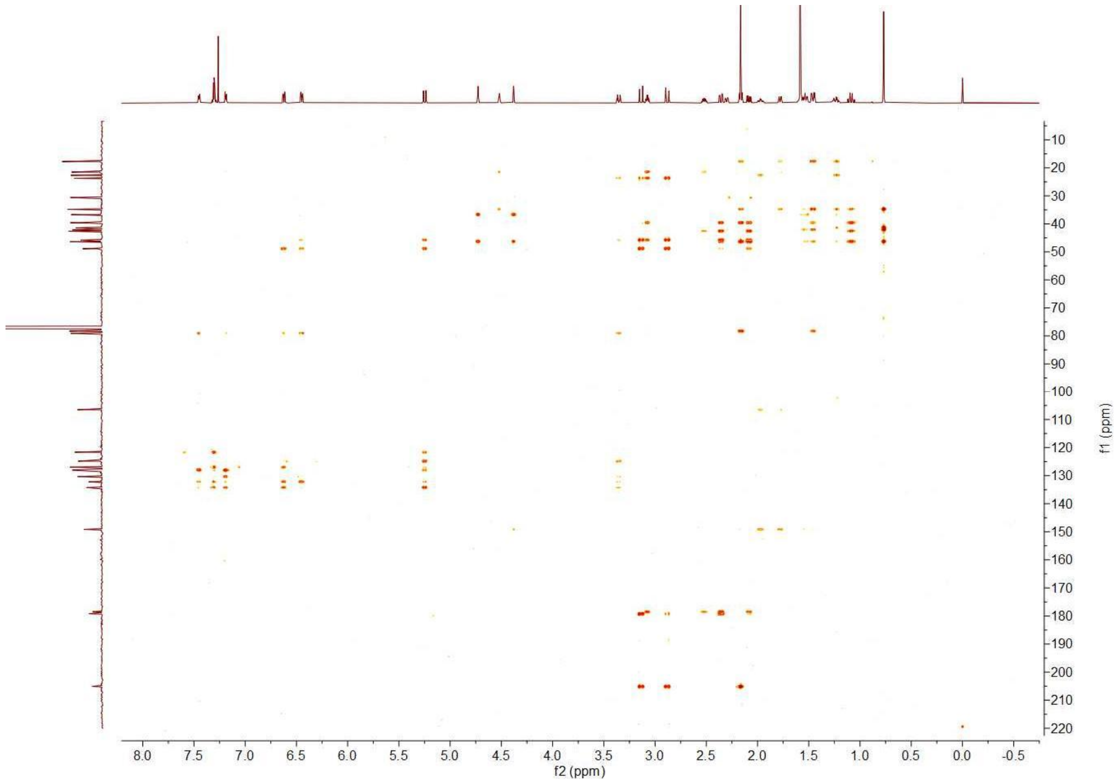

HMBC (600 MHz, $CDCl_3$)

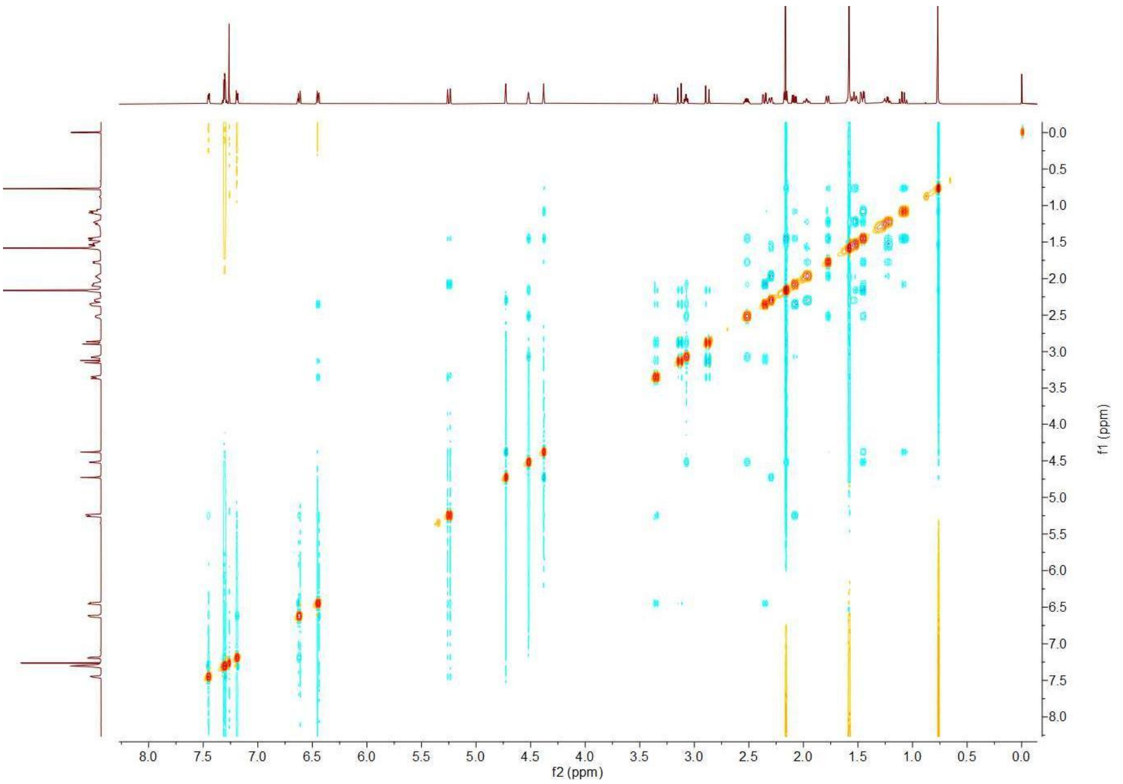

Noesy (600 MHz, $CDCl_3$)

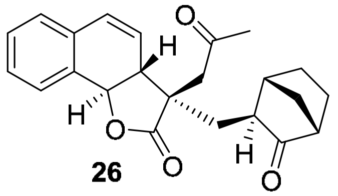

26

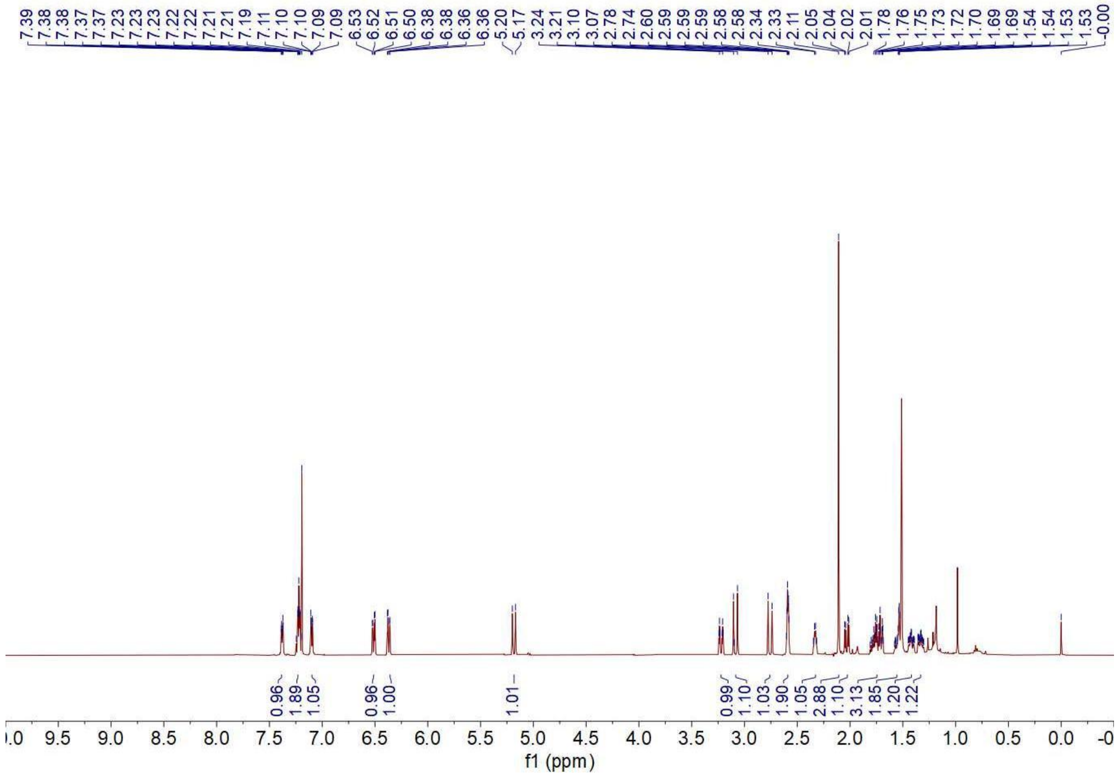
7.39 7.38 7.38 7.37 7.37 7.23 7.23 7.23 7.22 7.22 7.21 7.21 7.19 7.11 7.10 7.10 7.09 6.53 6.52 6.51 6.50 6.38 6.38 6.36 6.36 5.20 5.17 3.24 3.21 3.10 3.07 2.78 2.74 2.60 2.59 2.59 2.59 2.58 2.58 2.34 2.33 2.11 2.05 2.04 2.02 2.01 1.78 1.76 1.75 1.73 1.72 1.70 1.69 1.54 1.53 1.53 -0.00
0.96 1.89 1.05 0.96 1.00 1.01 0.99 1.10 1.03 1.90 1.05 2.88 1.10 3.13 1.85 1.20 1.22
.0 9.5 9.0 8.5 8.0 7.5 7.0 6.5 6.0 5.5 5.0 4.5 4.0 3.5 3.0 2.5 2.0 1.5 1.0 0.5 0.0 -0
f1 (ppm)

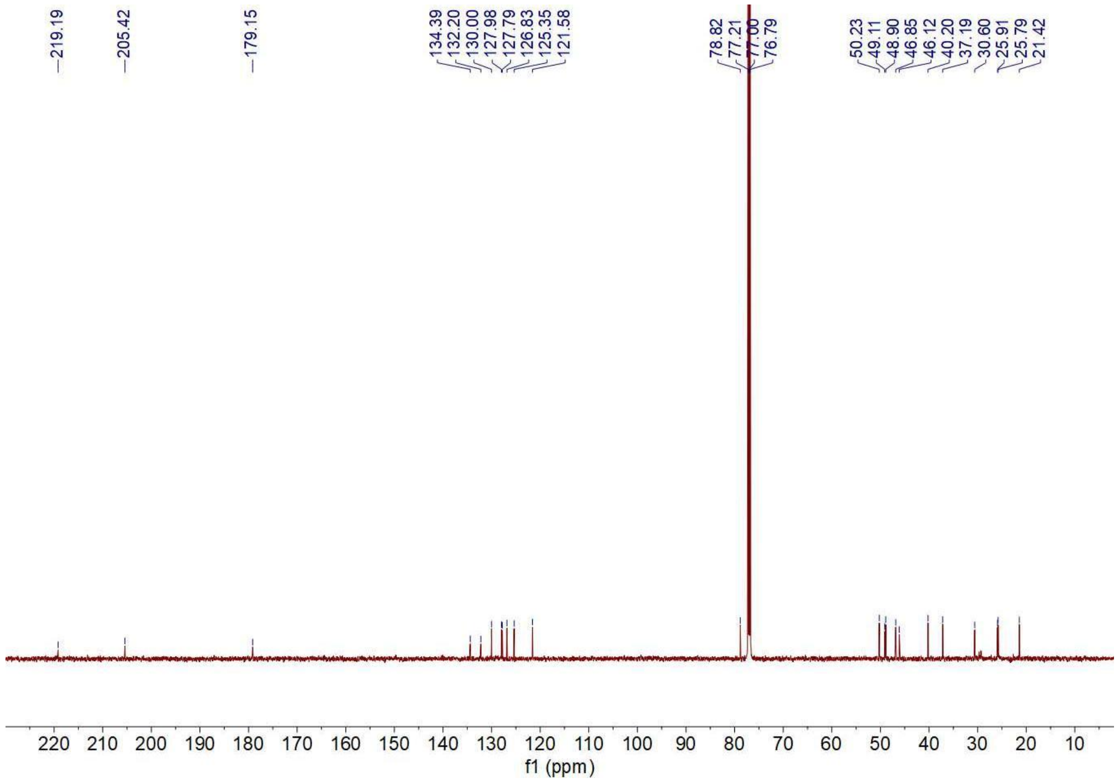
219.19 205.42 179.15 134.39 132.20 130.00 127.98 127.79 126.83 125.35 121.58 78.82 77.21 77.00 76.79 50.23 49.11 48.90 46.85 46.12 40.20 37.19 30.60 25.91 25.79 21.42
220 210 200 190 180 170 160 150 140 130 120 110 100 90 80 70 60 50 40 30 20 10
f1 (ppm)

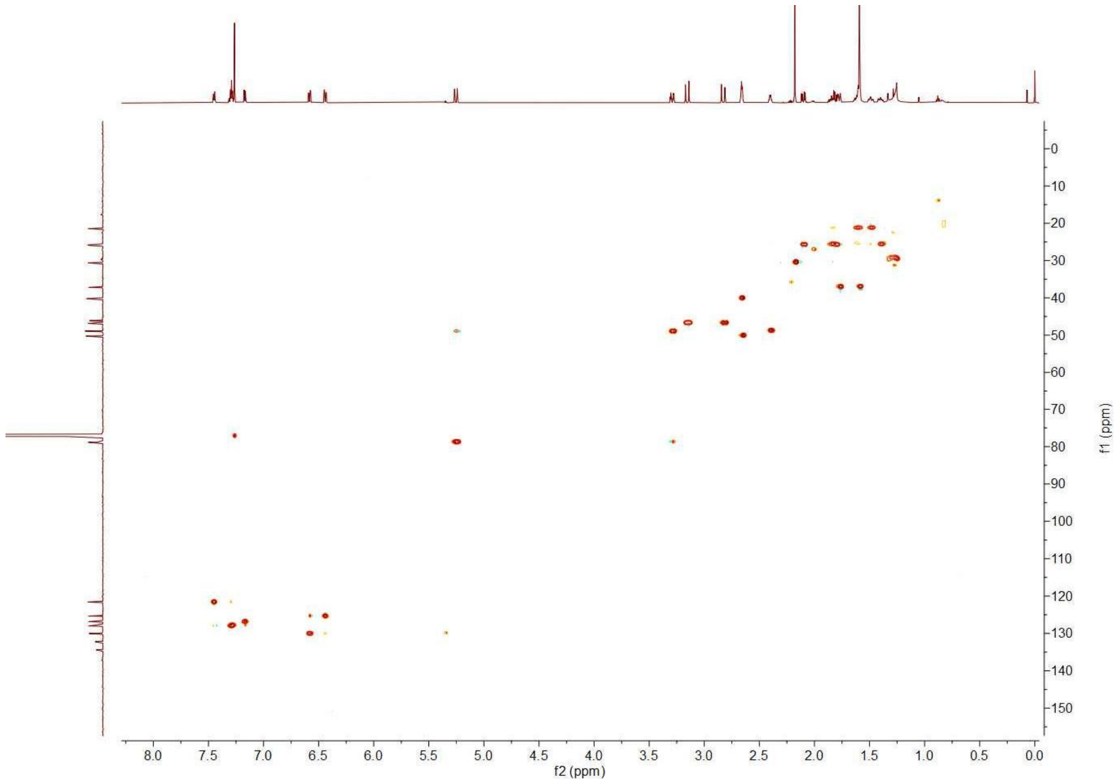

HSQC (600 MHz, $CDCl_3$)

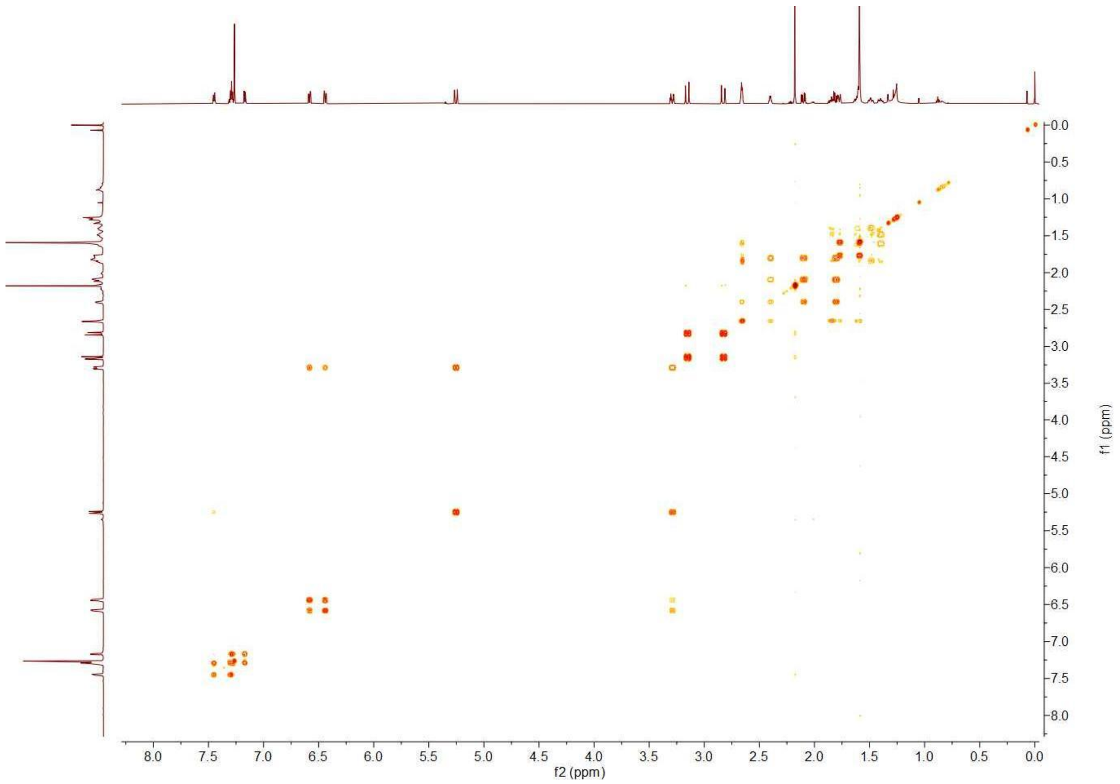

Cosy (600 MHz, $CDCl_3$)

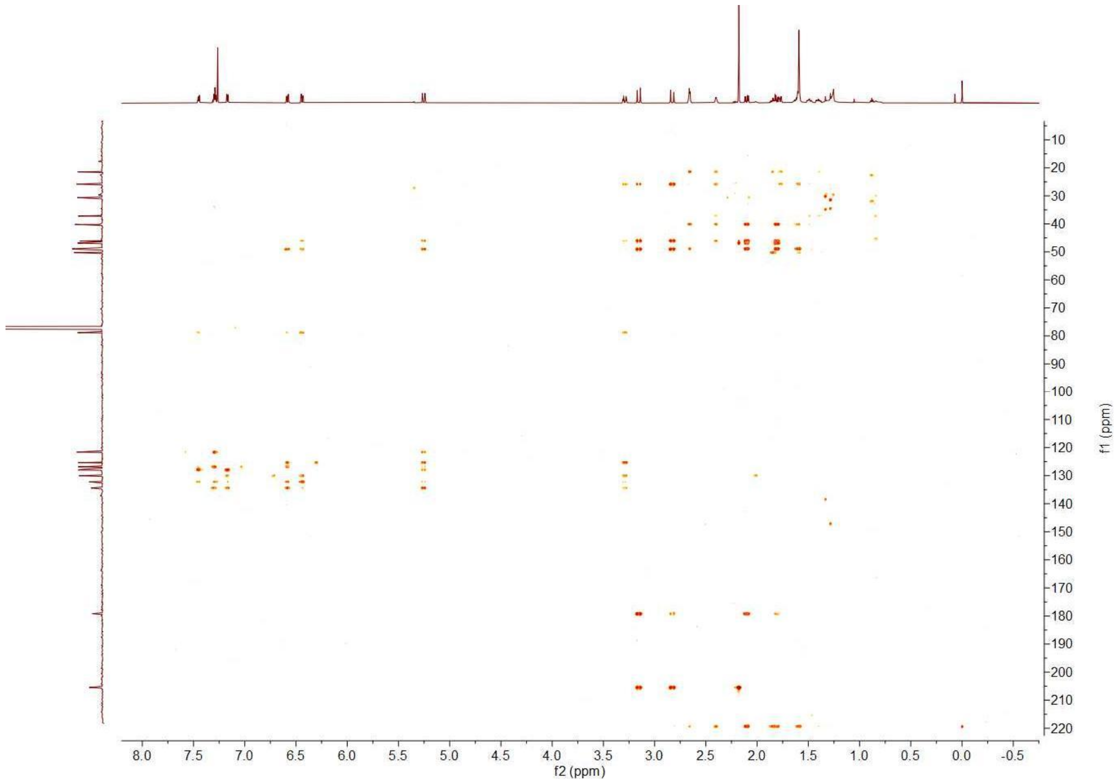

HMBC (600 MHz, $CDCl_3$)

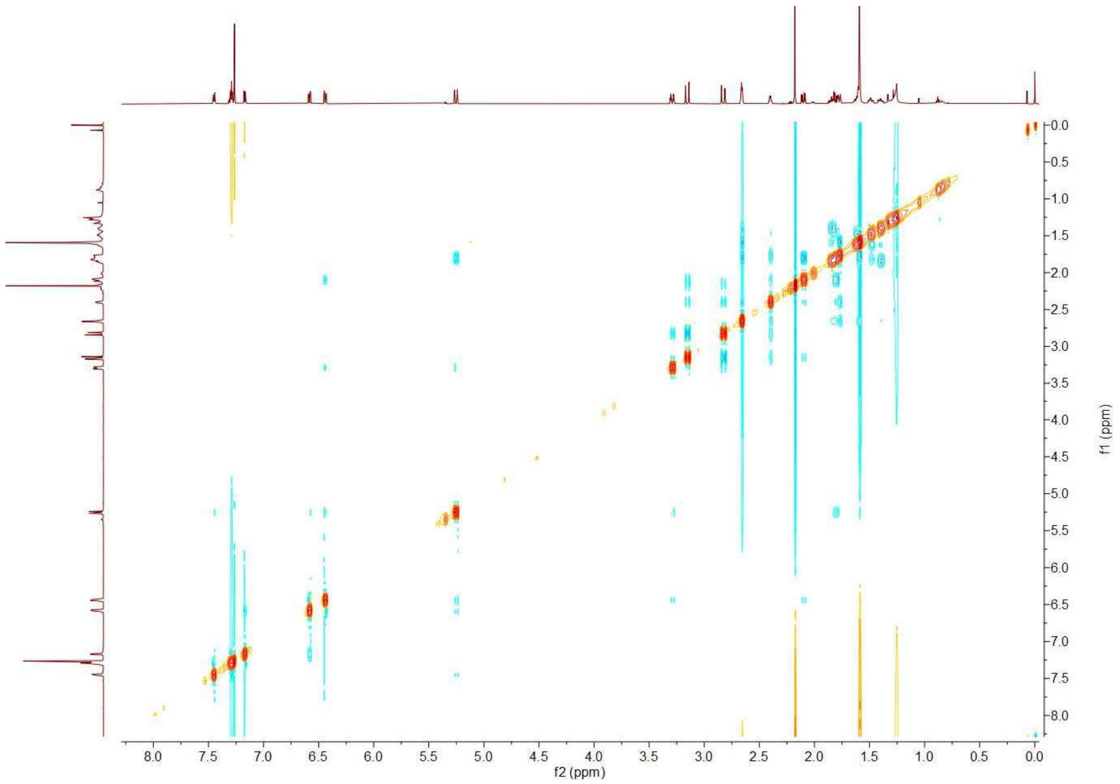

Noesy (600 MHz, $CDCl_3$)

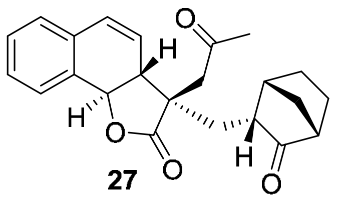

27

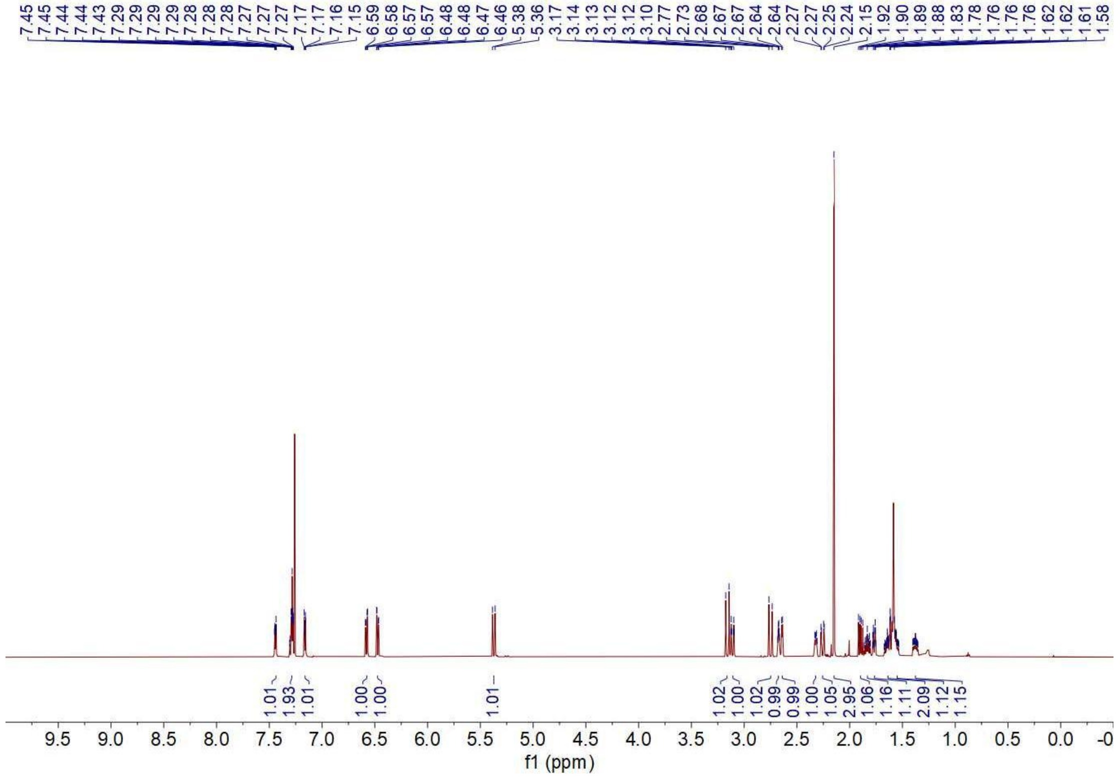
7.45 7.45 7.44 7.43 7.29 7.29 7.29 7.28 7.28 7.27 7.27 7.27 7.17 7.17 7.16 7.15 6.59 6.58 6.57 6.57 6.48 6.48 6.47 6.46 5.38 5.36 3.17 3.14 3.13 3.12 3.12 3.10 2.77 2.73 2.68 2.67 2.67 2.64 2.64 2.27 2.27 2.25 2.24 2.15 1.92 1.90 1.89 1.88 1.83 1.78 1.76 1.76 1.62 1.62 1.61 1.58
1.01 1.93 1.01 1.00 1.00 1.01 1.02 1.00 1.02 0.99 0.99 1.00 1.05 2.95 1.06 1.16 1.11 2.09 1.12 1.15
9.5 9.0 8.5 8.0 7.5 7.0 6.5 6.0 5.5 5.0 4.5 4.0 3.5 3.0 2.5 2.0 1.5 1.0 0.5 0.0 -0
f1 (ppm)

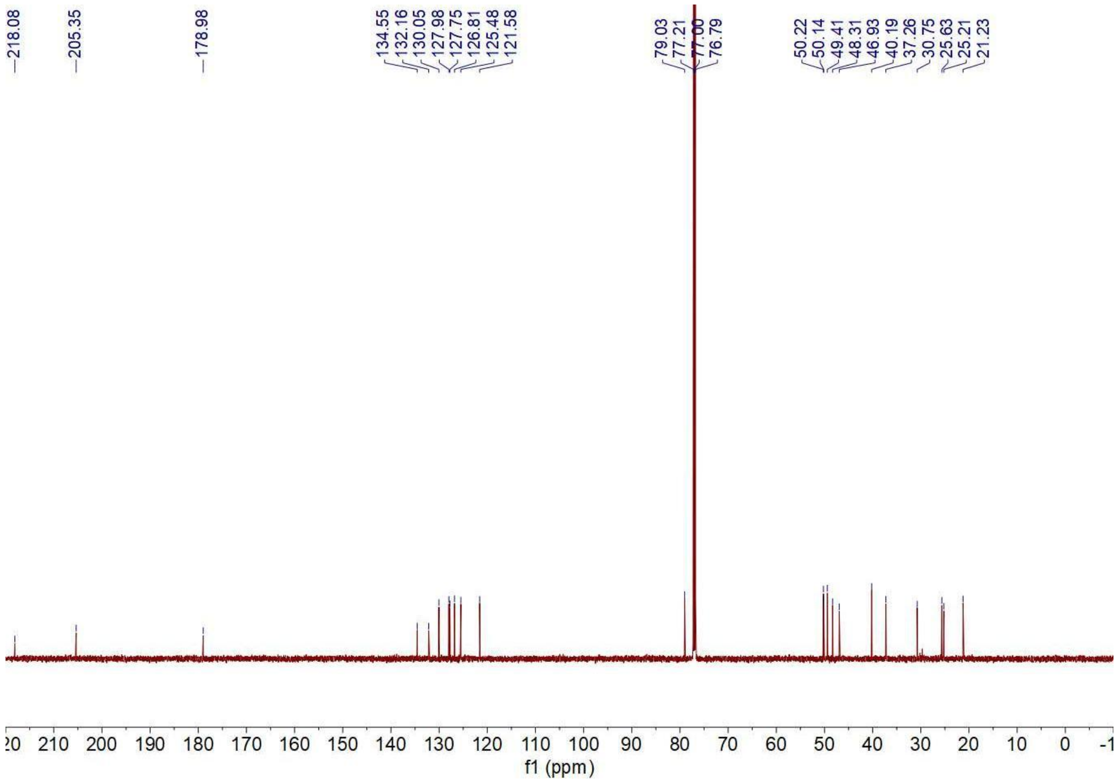
218.08 205.35 178.98 134.55 132.16 130.05 127.98 127.75 126.81 125.48 121.58 79.03 77.21 77.00 76.79 50.22 50.14 49.41 48.31 46.93 40.19 37.26 30.75 25.63 25.21 21.23
210 200 190 180 170 160 150 140 130 120 110 100 90 80 70 60 50 40 30 20 10 0
f1 (ppm)

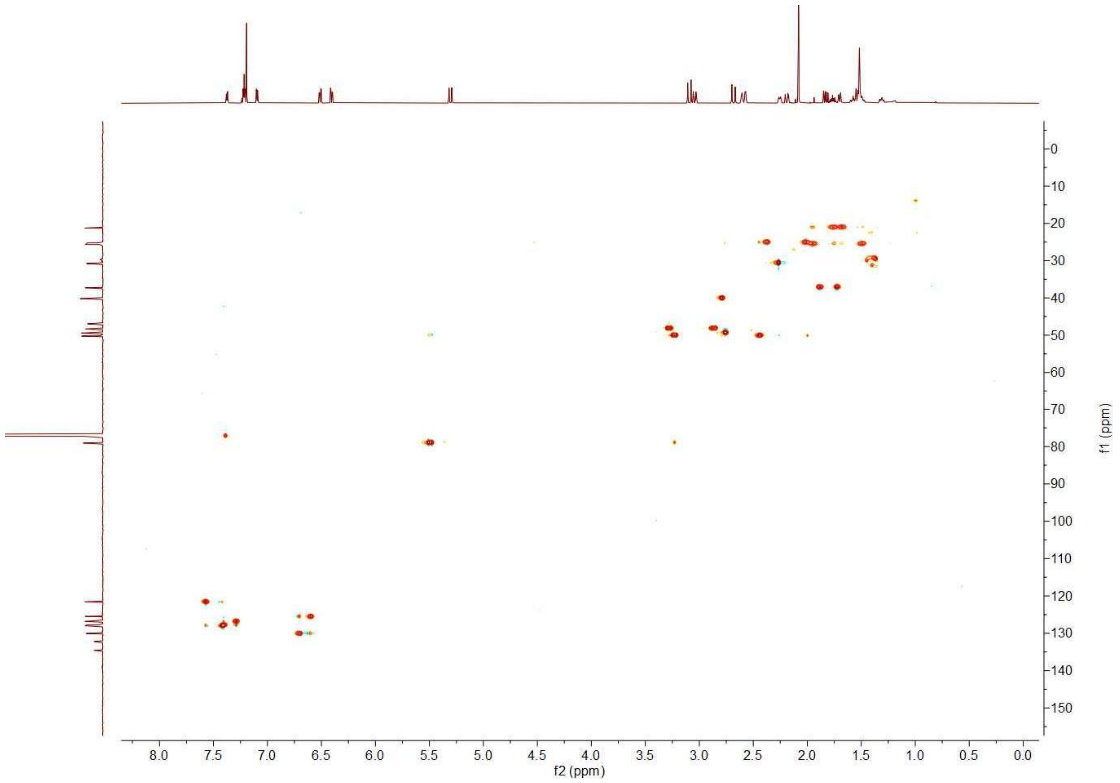

HSQC (600 MHz, $CDCl_3$)

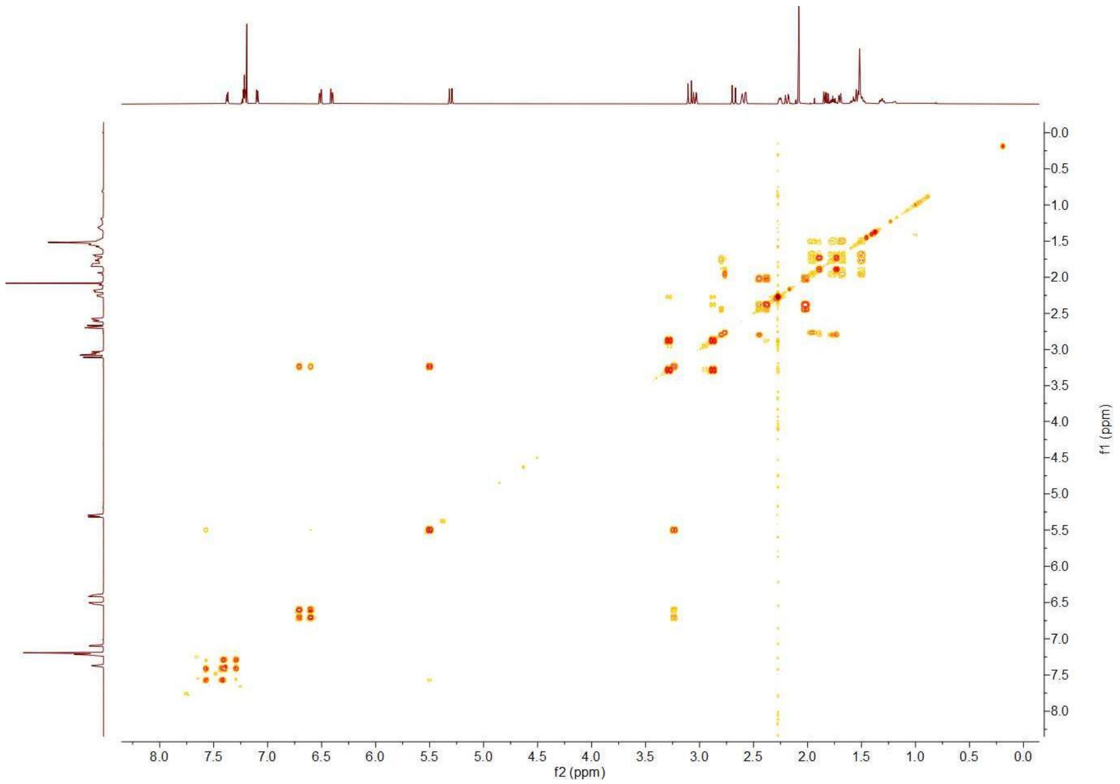

Cosy (600 MHz, $CDCl_3$)

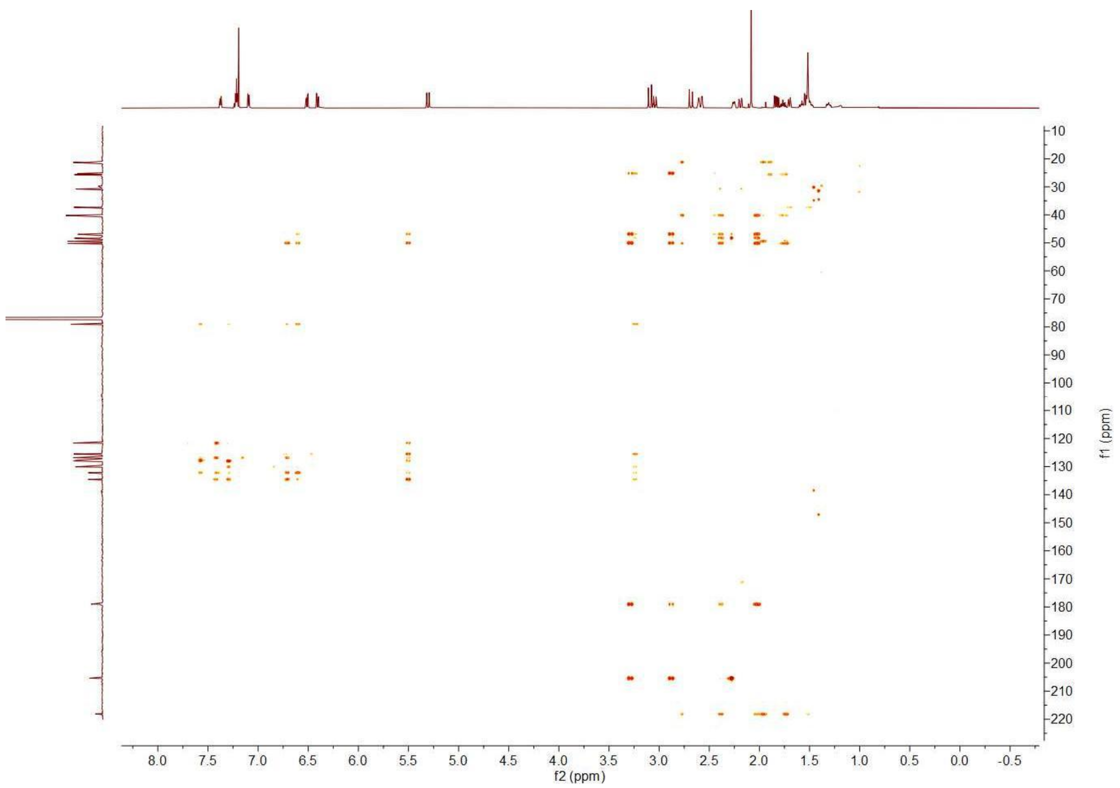

HMBC (600 MHz, $CDCl_3$)

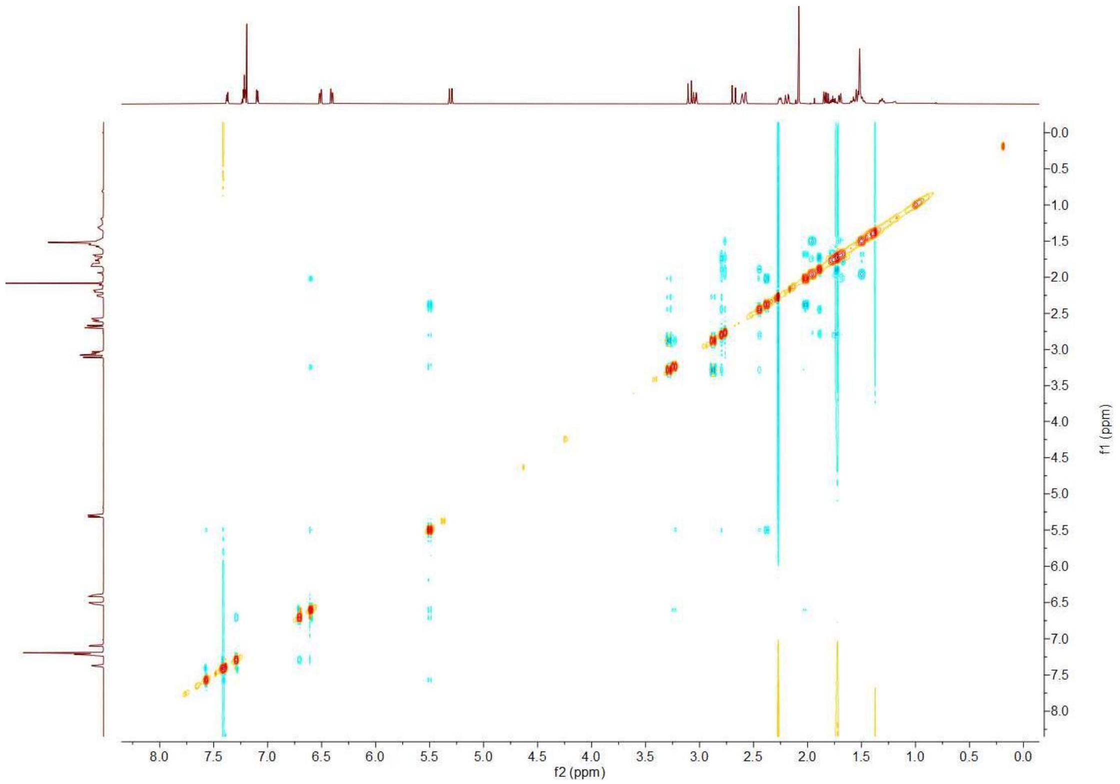

Noesy (600 MHz, $CDCl_3$)

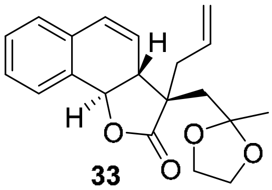

33

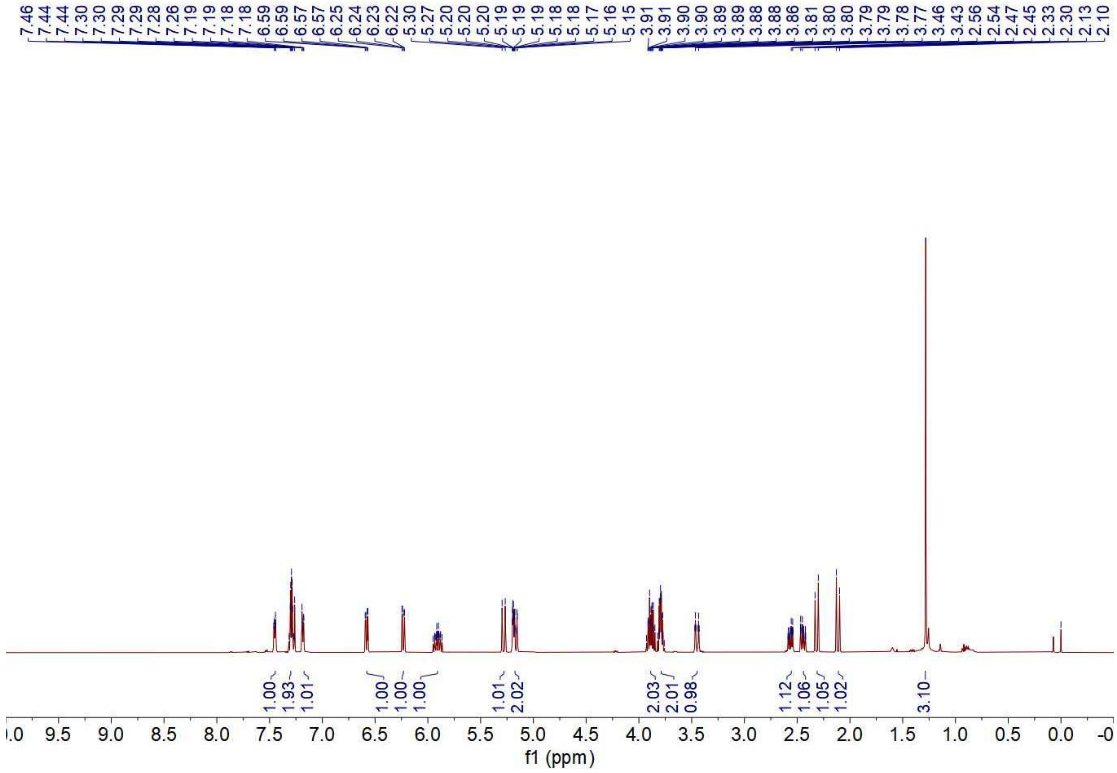
7.46
7.44
7.44
7.30
7.30
7.29
7.29
7.28
7.26
7.19
7.19
7.18
7.18
6.59
6.59
6.57
6.57
6.25
6.24
6.23
6.22
5.30
5.27
5.20
5.20
5.20
5.19
5.19
5.19
5.18
5.18
5.17
5.16
5.15
3.91
3.91
3.90
3.90
3.89
3.89
3.88
3.88
3.86
3.81
3.80
3.80
3.79
3.79
3.78
3.77
3.46
3.43
2.56
2.54
2.47
2.45
2.33
2.30
2.13
2.10
1.00
1.93
1.01
1.00
1.00
1.00
1.01
2.02
2.03
2.01
0.98
1.12
1.06
1.05
1.02
3.10
9.5 9.0 8.5 8.0 7.5 7.0 6.5 6.0 5.5 5.0 4.5 4.0 3.5 3.0 2.5 2.0 1.5 1.0 0.5 0.0
f1 (ppm)

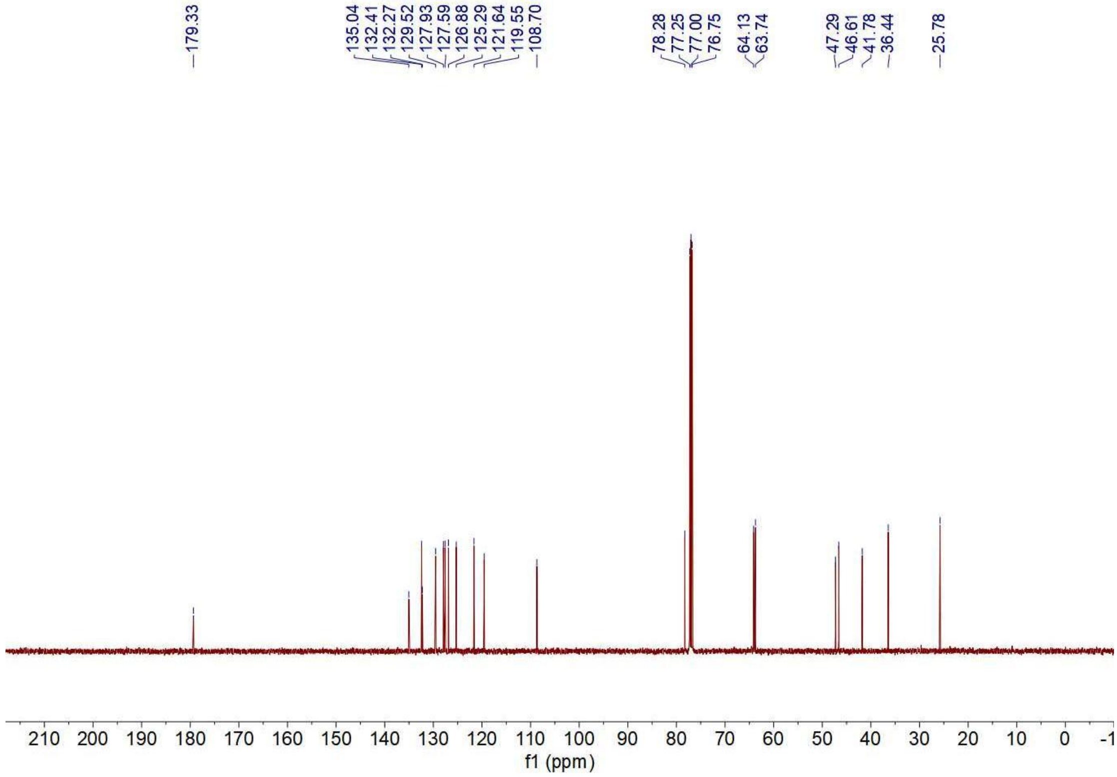
179.33
135.04
132.41
132.27
129.52
127.93
127.59
126.88
125.29
121.64
119.55
108.70
78.28
77.25
77.00
76.75
64.13
63.74
47.29
46.61
41.78
36.44
25.78
210 200 190 180 170 160 150 140 130 120 110 100 90 80 70 60 50 40 30 20 10 0
f1 (ppm)

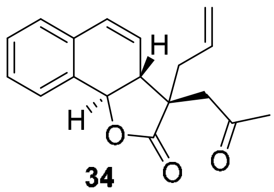

H
H
O
O
O

34

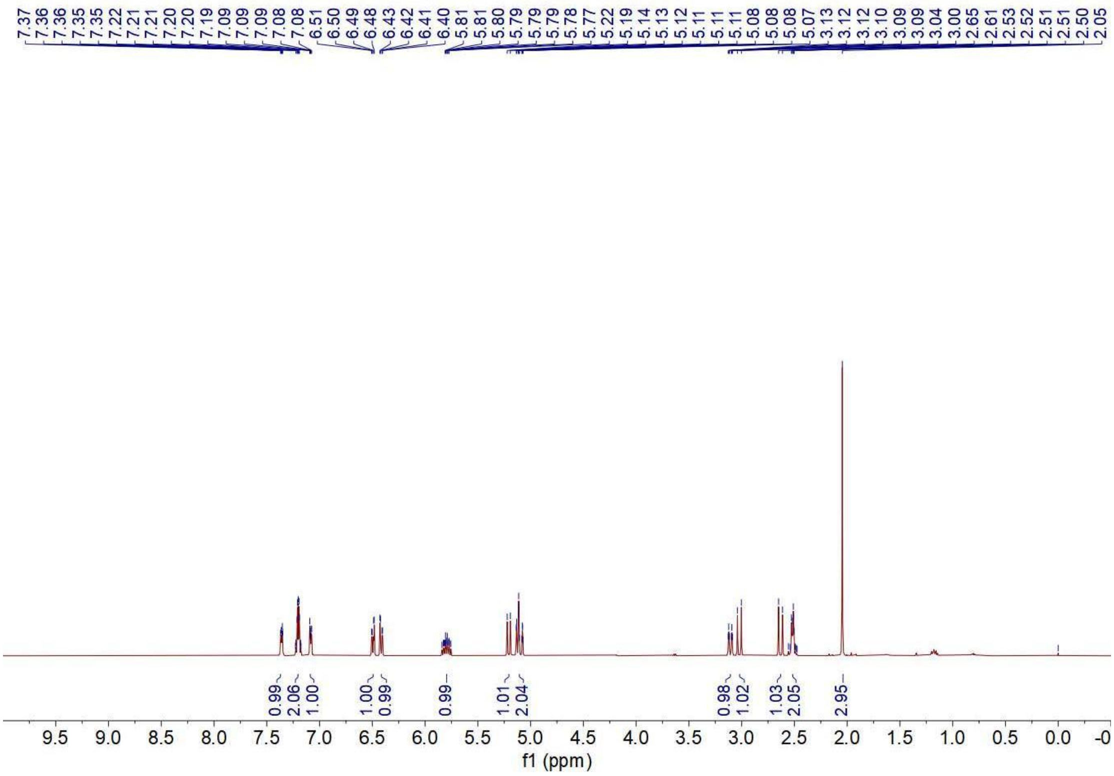

7.37
7.36
7.36
7.35
7.35
7.22
7.21
7.21
7.20
7.20
7.19
7.09
7.09
7.09
7.08
7.08
6.51
6.50
6.49
6.48
6.43
6.42
6.41
6.40
5.81
5.81
5.80
5.79
5.79
5.79
5.78
5.77
5.22
5.19
5.14
5.13
5.12
5.11
5.11
5.11
5.08
5.08
5.07
3.13
3.12
3.12
3.10
3.09
3.09
3.04
3.00
2.65
2.61
2.53
2.52
2.51
2.50
2.05
0.99
2.06
1.00
1.00
0.99
0.99
1.01
2.04
0.98
1.02
1.03
2.05
2.95
9.5 9.0 8.5 8.0 7.5 7.0 6.5 6.0 5.5 5.0 4.5 4.0 3.5 3.0 2.5 2.0 1.5 1.0 0.5 0.0 -0
f1 (ppm)

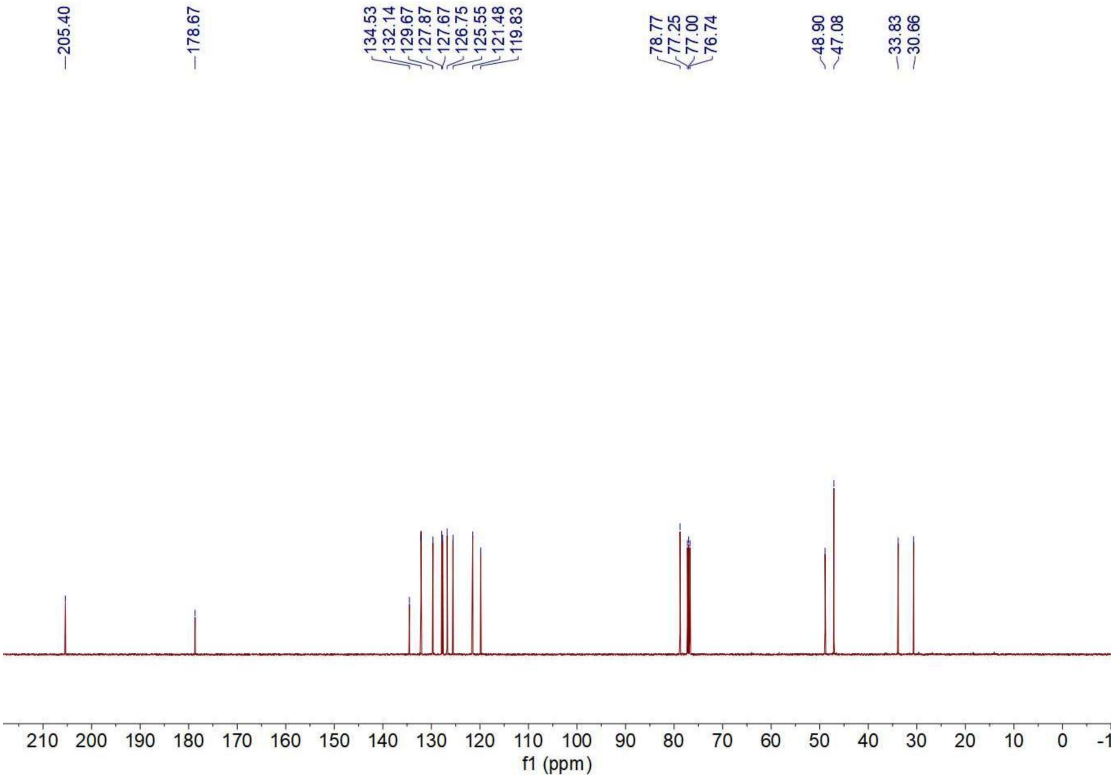

205.40
178.67
134.53
132.14
129.67
127.87
127.67
126.75
125.55
121.48
119.83
78.77
77.25
77.00
76.74
48.90
47.08
33.83
30.66
210 200 190 180 170 160 150 140 130 120 110 100 90 80 70 60 50 40 30 20 10 0 -1
f1 (ppm)

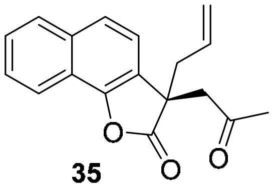

35

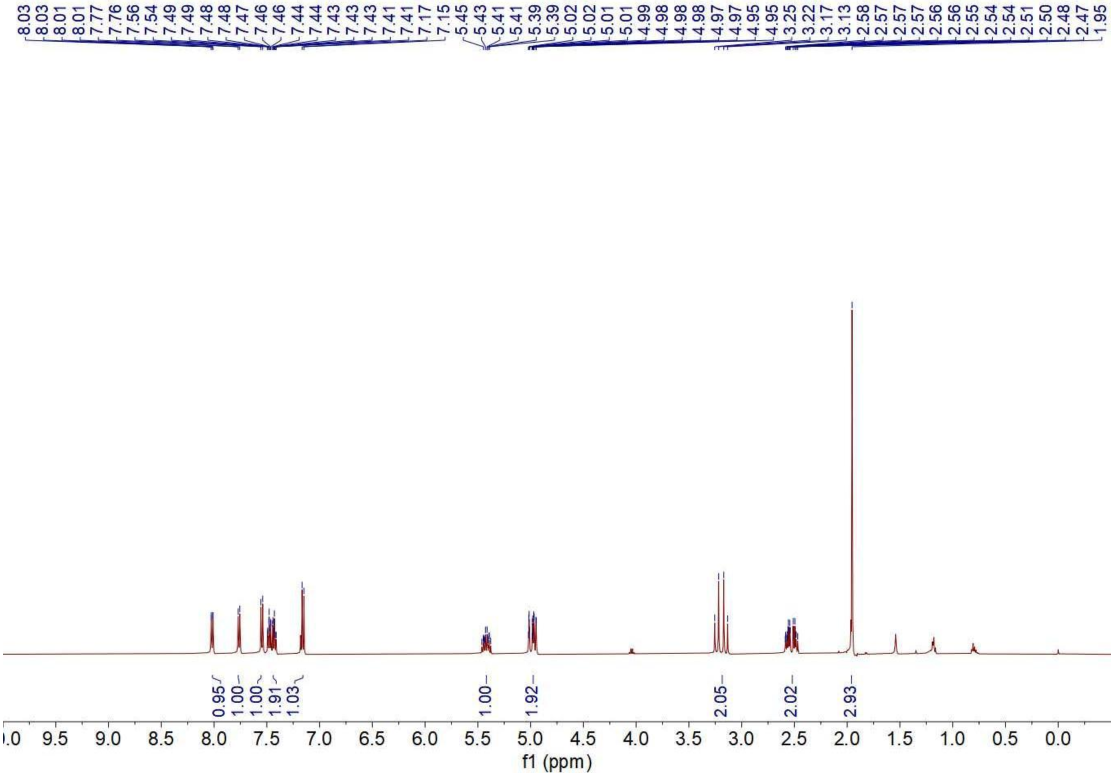
8.03 8.03 8.01 7.77 7.76 7.56 7.54 7.49 7.49 7.48 7.48 7.47 7.46 7.46 7.44 7.44 7.43 7.43 7.43 7.43 7.41 7.41 7.17 7.15 5.45 5.43 5.41 5.41 5.39 5.39 5.02 5.02 5.01 4.99 4.98 4.98 4.98 4.97 4.97 4.95 4.95 3.25 3.22 3.17 3.13 2.58 2.57 2.57 2.57 2.56 2.55 2.54 2.54 2.51 2.50 2.48 2.47 1.95
0.95
1.00
1.00
1.91
1.03
1.00
1.92
2.05
2.02
2.93
.0 9.5 9.0 8.5 8.0 7.5 7.0 6.5 6.0 5.5 5.0 4.5 4.0 3.5 3.0 2.5 2.0 1.5 1.0 0.5 0.0
f1 (ppm)

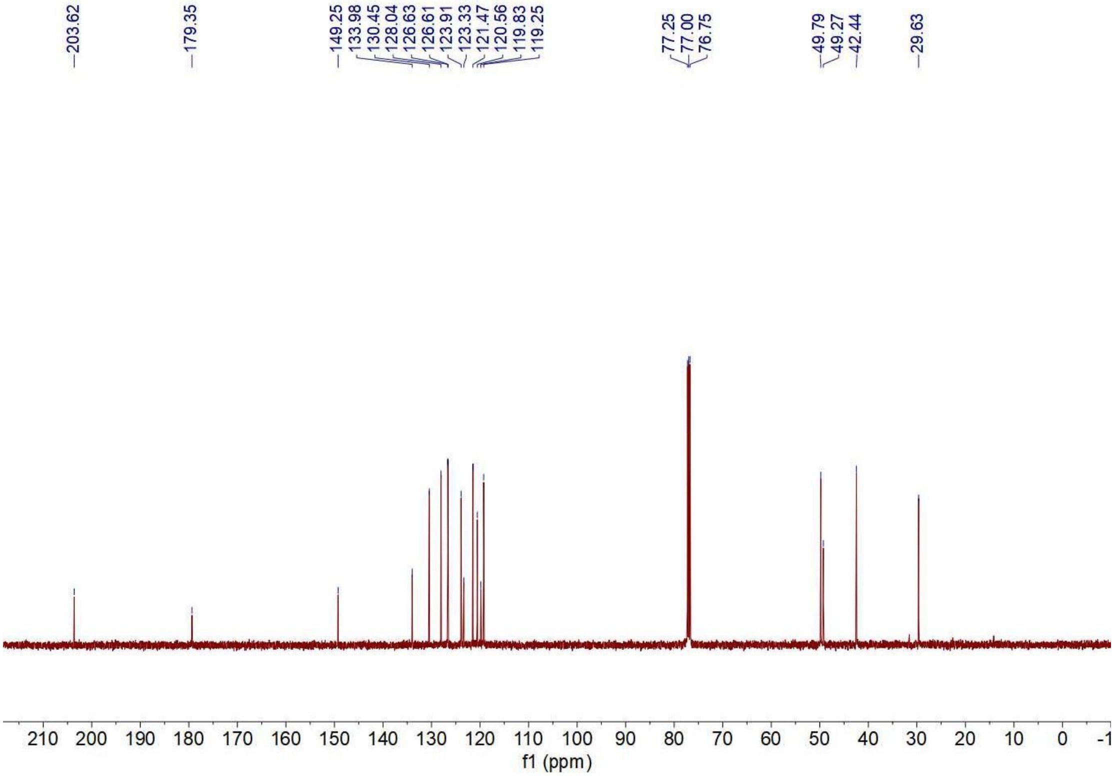
203.62
179.35
149.25 133.98 130.45 128.04 126.63 126.61 123.91 123.33 121.47 120.56 119.83 119.25
77.25 77.00 76.75
49.79 49.27 42.44
29.63
210 200 190 180 170 160 150 140 130 120 110 100 90 80 70 60 50 40 30 20 10 0 -1
f1 (ppm)

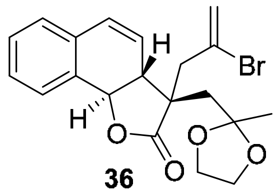
H
Br
H
O
O
O
O

36

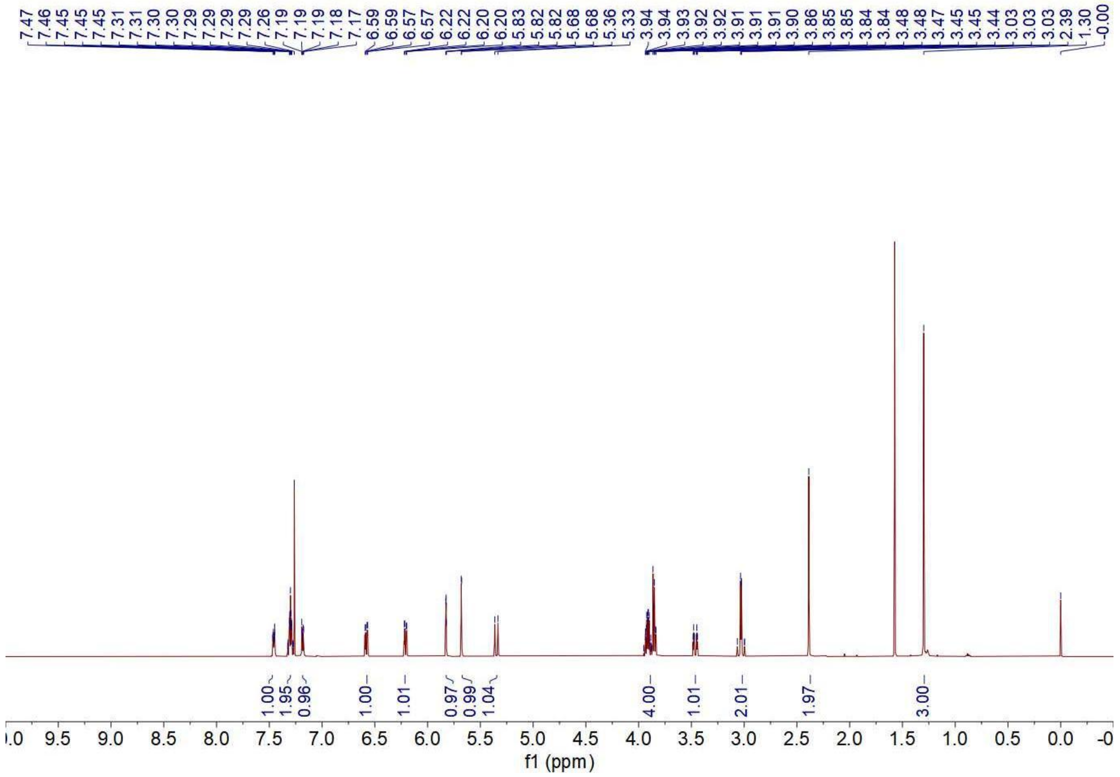
7.47
7.46
7.45
7.45
7.31
7.30
7.29
7.29
7.29
7.26
7.19
7.19
7.18
7.17
6.59
6.59
6.57
6.57
6.22
6.22
6.20
6.20
5.83
5.82
5.68
5.68
5.36
5.33
3.94
3.94
3.93
3.92
3.92
3.91
3.91
3.90
3.86
3.85
3.85
3.84
3.84
3.48
3.48
3.47
3.45
3.45
3.44
3.03
3.03
3.03
2.39
1.30
-0.00
1.00
1.95
0.96
1.00
1.01
0.97
0.99
1.04
4.00
1.01
2.01
1.97
3.00
9.5 9.0 8.5 8.0 7.5 7.0 6.5 6.0 5.5 5.0 4.5 4.0 3.5 3.0 2.5 2.0 1.5 1.0 0.5 0.0
f1 (ppm)

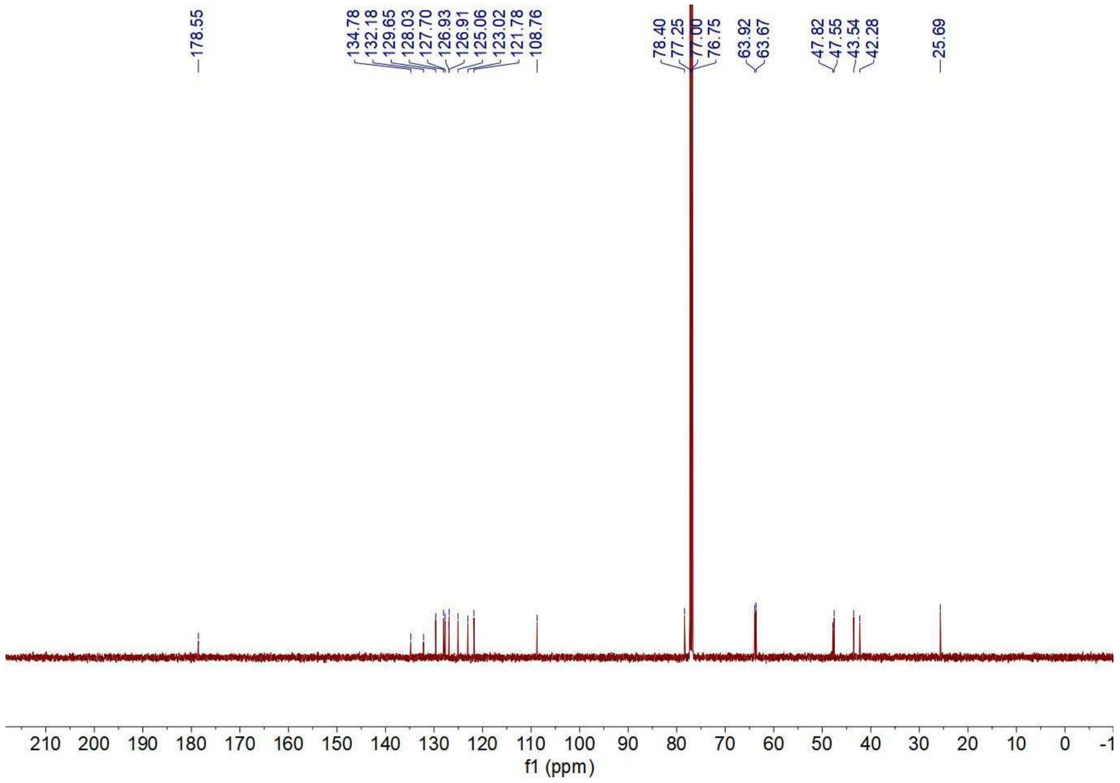
178.55
134.78
132.18
129.65
128.03
127.70
126.93
126.91
125.06
123.02
121.78
108.76
78.40
77.25
77.00
76.75
63.92
63.67
47.82
47.55
43.54
42.28
25.69
210 200 190 180 170 160 150 140 130 120 110 100 90 80 70 60 50 40 30 20 10 0
f1 (ppm)

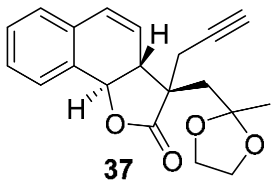
H
H
O
O
O
O

37

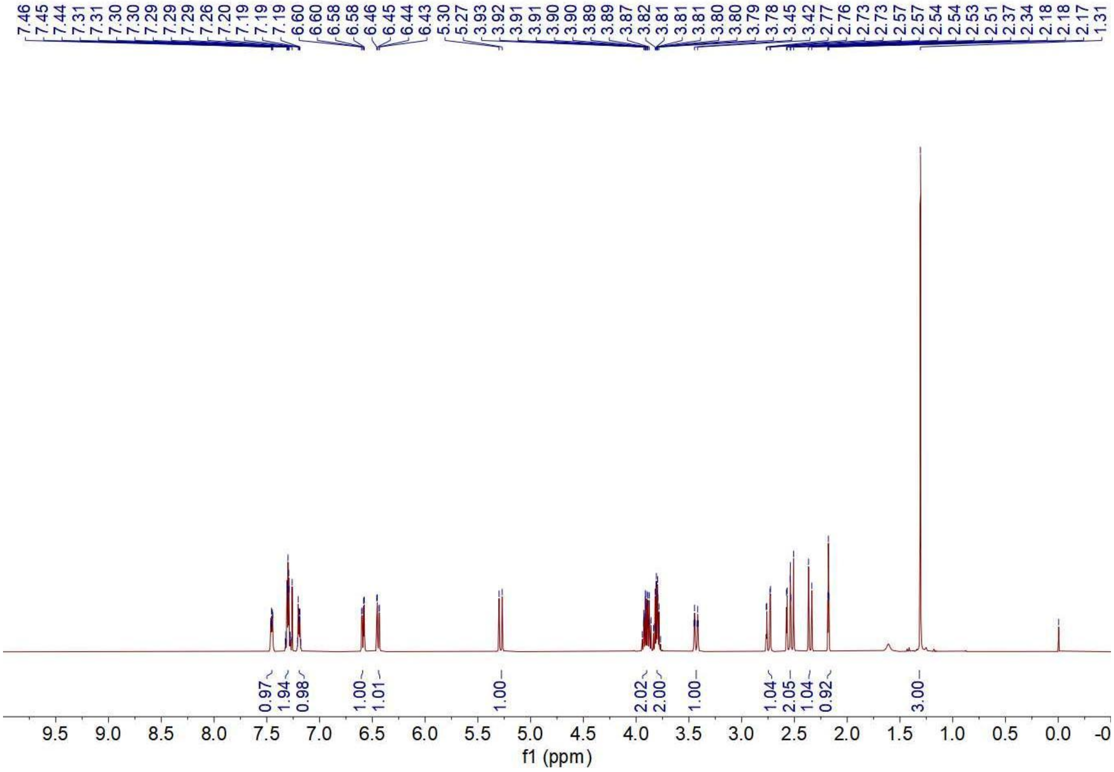
7.46
7.45
7.44
7.31
7.31
7.30
7.30
7.29
7.29
7.26
7.20
7.19
7.19
6.60
6.60
6.58
6.58
6.46
6.45
6.44
6.43
5.30
5.27
3.93
3.92
3.91
3.91
3.90
3.89
3.89
3.87
3.82
3.81
3.81
3.81
3.80
3.80
3.79
3.78
3.45
3.42
2.77
2.76
2.73
2.73
2.57
2.57
2.54
2.54
2.53
2.51
2.37
2.34
2.18
2.17
1.31
0.97
1.94
0.98
1.00
1.01
1.00
2.02
2.00
1.00
1.04
2.05
1.04
0.92
3.00
9.5 9.0 8.5 8.0 7.5 7.0 6.5 6.0 5.5 5.0 4.5 4.0 3.5 3.0 2.5 2.0 1.5 1.0 0.5 0.0 -0
f1 (ppm)

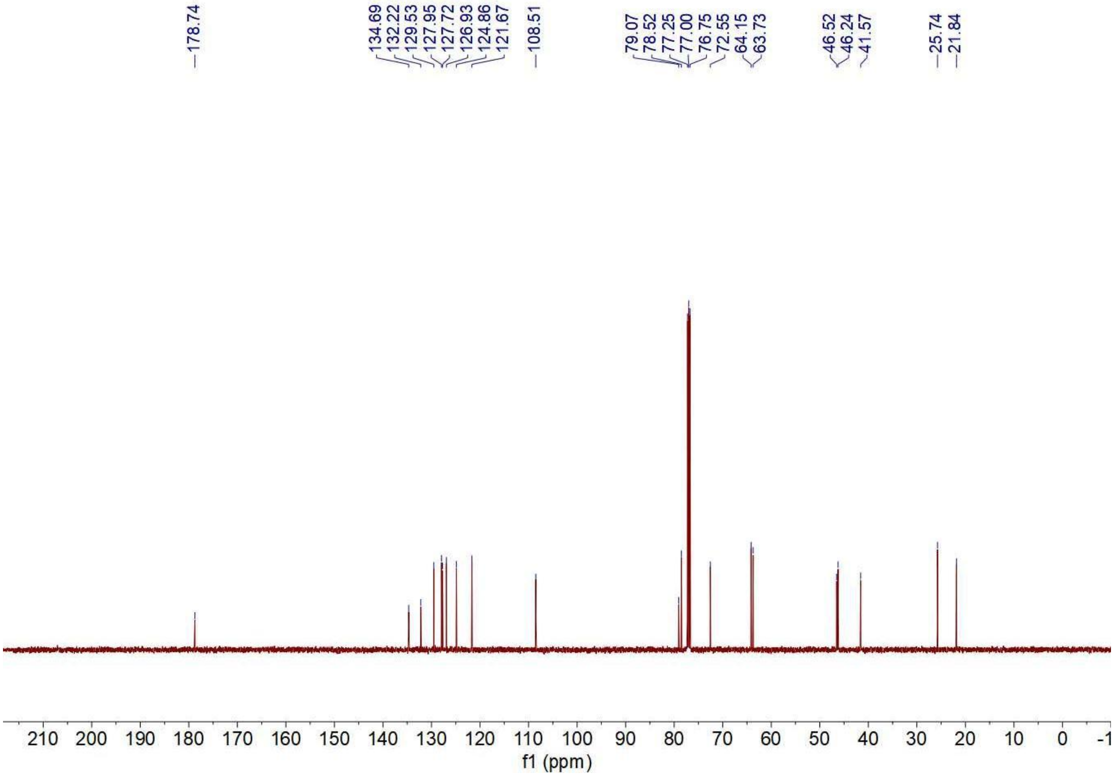
178.74
134.69
132.22
129.53
127.95
127.72
126.93
124.86
121.67
108.51
79.07
78.52
77.25
77.00
76.75
72.55
64.15
63.73
46.52
46.24
41.57
25.74
21.84
210 200 190 180 170 160 150 140 130 120 110 100 90 80 70 60 50 40 30 20 10 0 -1
f1 (ppm)

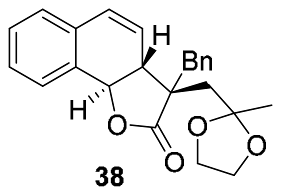

38

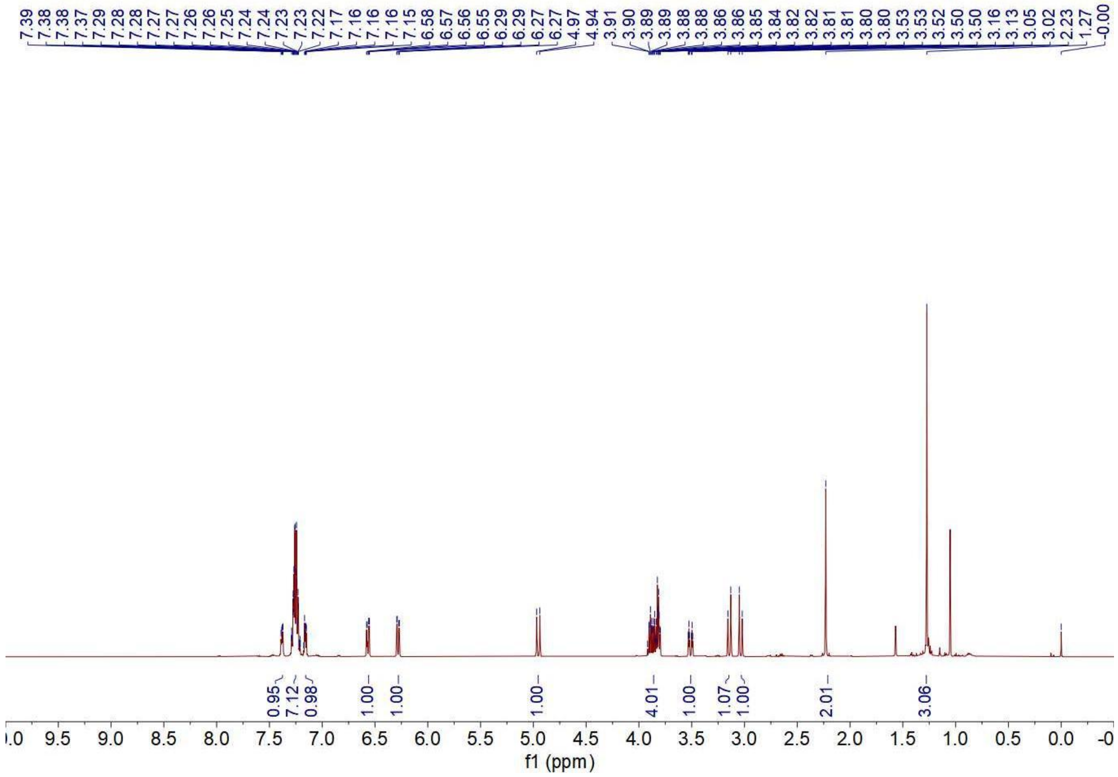

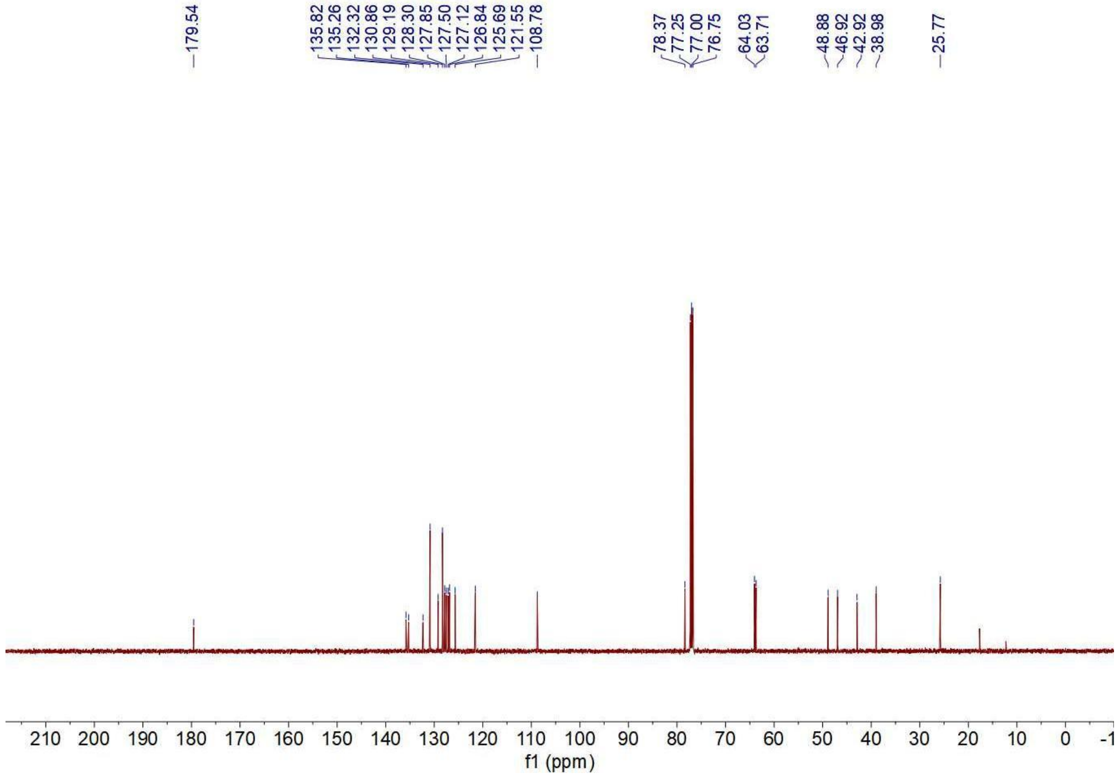

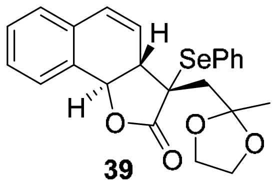
H
SePh
H
O
O
O
O

39

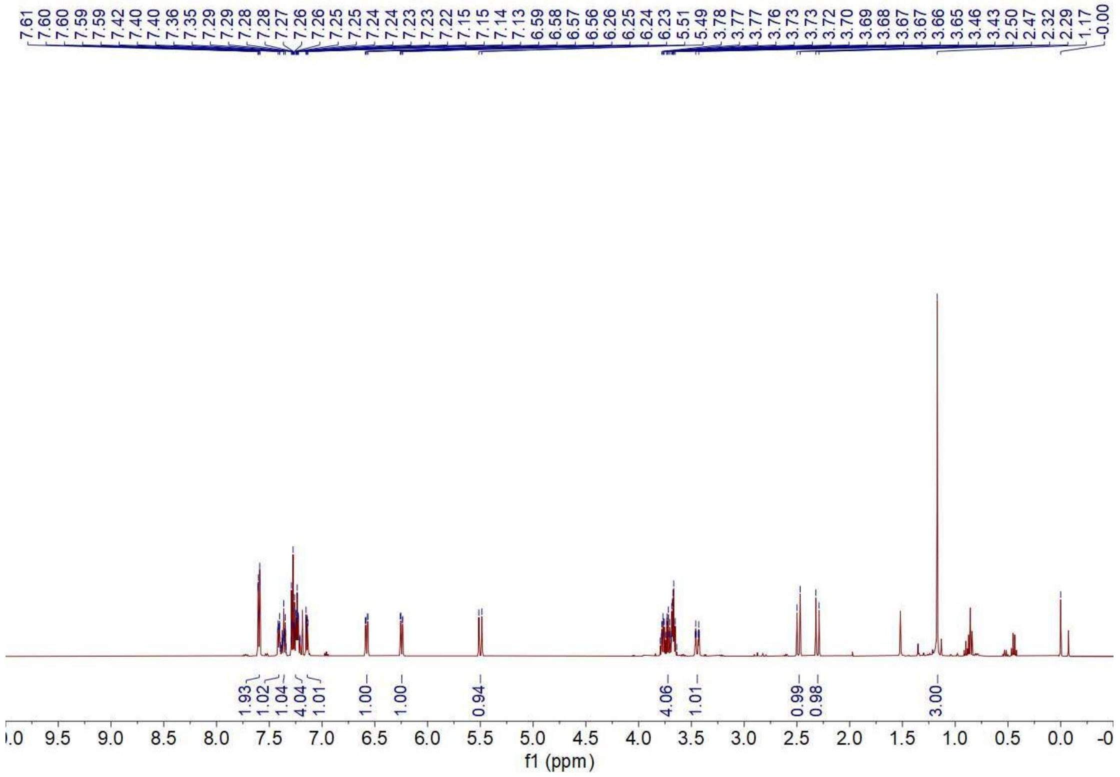
7.61
7.60
7.59
7.59
7.42
7.40
7.40
7.36
7.35
7.29
7.29
7.28
7.28
7.27
7.26
7.26
7.25
7.25
7.24
7.24
7.23
7.23
7.22
7.15
7.15
7.14
7.13
6.59
6.58
6.57
6.56
6.26
6.25
6.24
6.23
5.51
5.49
3.78
3.77
3.77
3.76
3.73
3.73
3.72
3.70
3.69
3.68
3.67
3.67
3.66
3.65
3.46
3.43
2.50
2.47
2.32
2.29
1.17
-0.00
1.93
1.02
1.04
4.04
1.01
1.00
1.00
0.94
4.06
1.01
0.99
0.98
3.00
9.5 9.0 8.5 8.0 7.5 7.0 6.5 6.0 5.5 5.0 4.5 4.0 3.5 3.0 2.5 2.0 1.5 1.0 0.5 0.0
f1 (ppm)

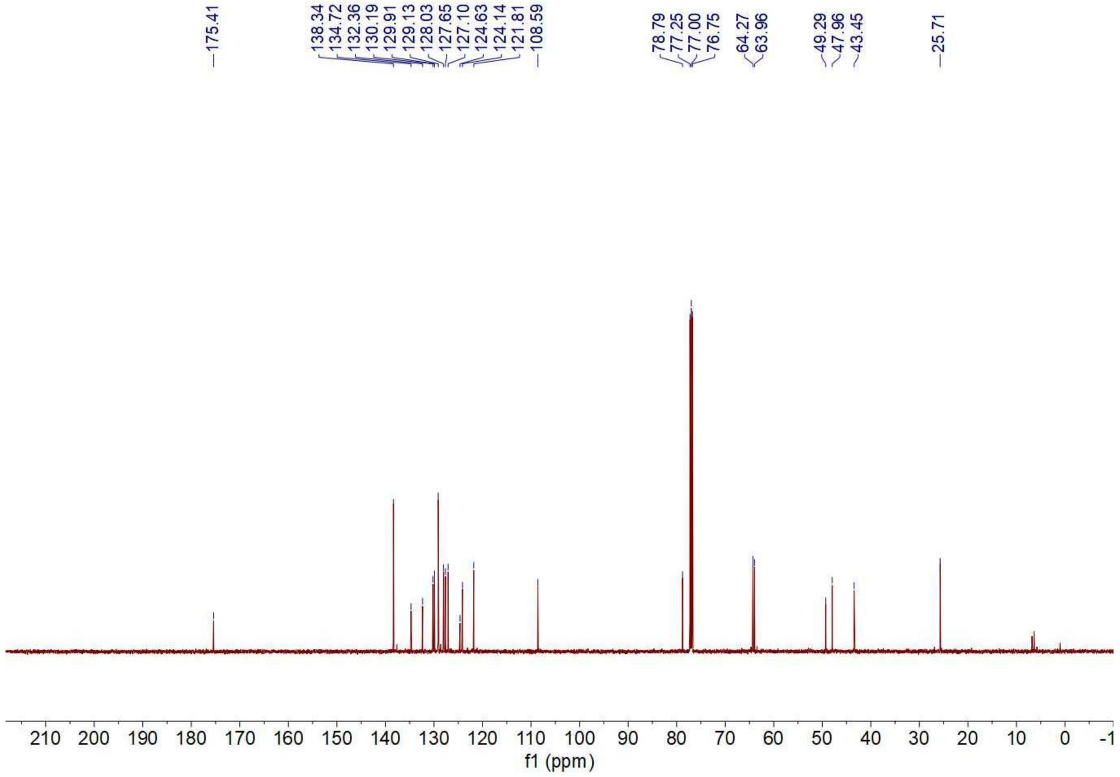
175.41
138.34
134.72
132.36
130.19
129.91
129.13
128.03
127.65
127.10
124.63
124.14
121.81
108.59
78.79
77.25
77.00
76.75
64.27
63.96
49.29
47.96
43.45
25.71
210 200 190 180 170 160 150 140 130 120 110 100 90 80 70 60 50 40 30 20 10 0
f1 (ppm)

10. HPLC spectra

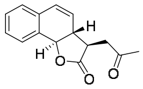

(*R*,*R*,*R*)-**3a**

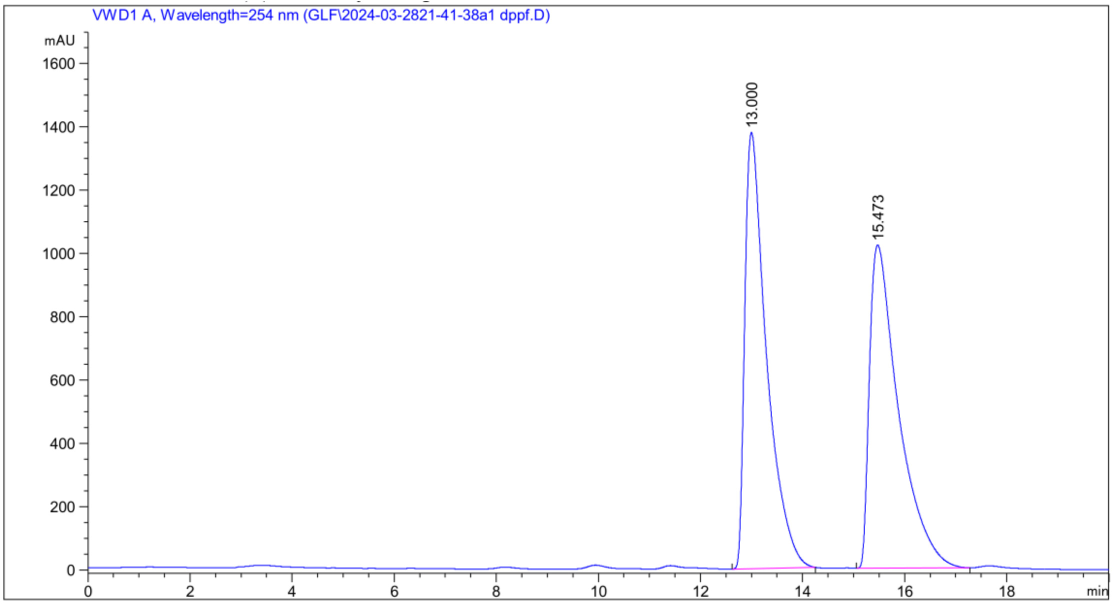

Peak #	RetTime [min]	Type	Width [min]	Area [mAU*s]	Height [mAU]	Area %
1	13.000	BBA	0.4344	4.08203e4	1378.83777	50.1907
2	15.473	VB	0.5816	4.05101e4	1021.62250	49.8093

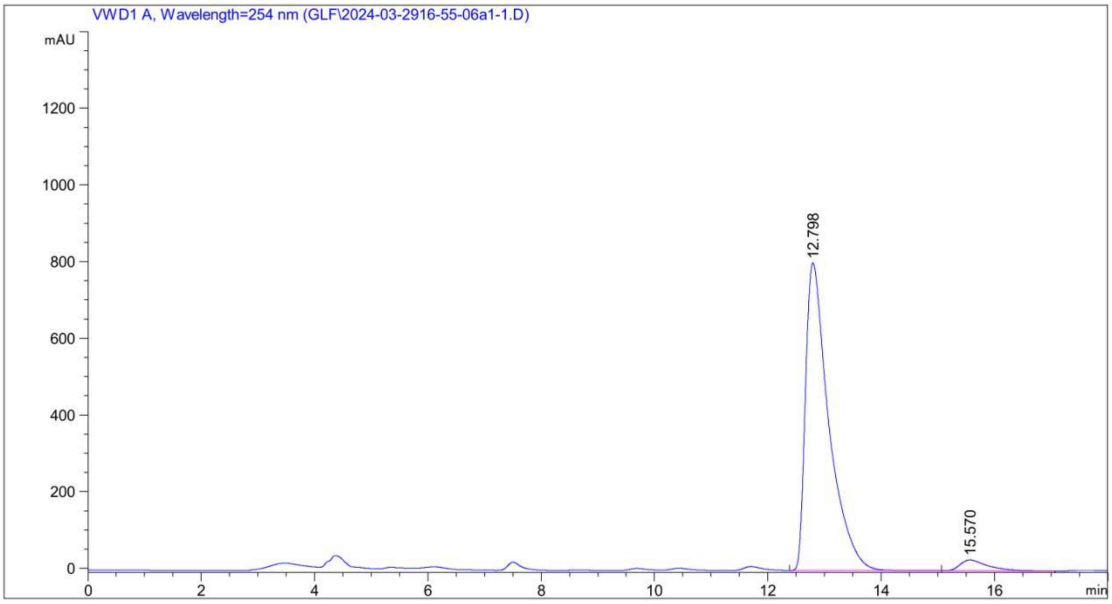

Peak #	RetTime [min]	Type	Width [min]	Area [mAU*s]	Height [mAU]	Area %
1	12.798	VBA	0.4220	2.29004e4	802.37042	96.0175
2	15.570	BB	0.5013	949.84607	28.03418	3.9825

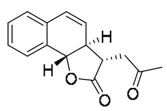

(*S*,*S*,*S*)-**3a**

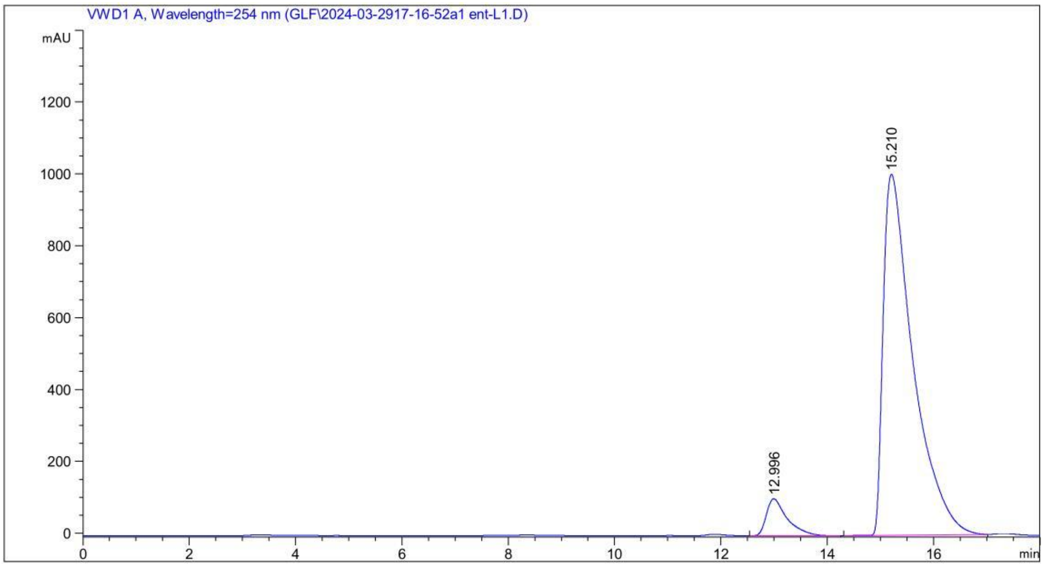

Peak #	RetTime [min]	Type	Width [min]	Area [mAU*s]	Height [mAU]	Area %
1	12.996	BB	0.3997	2816.30469	103.09399	6.7326
2	15.210	VB R	0.5739	3.90143e4	1004.90173	93.2674

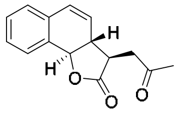

Gram-scale reaction of (*R*,*R*,*R*)-3a

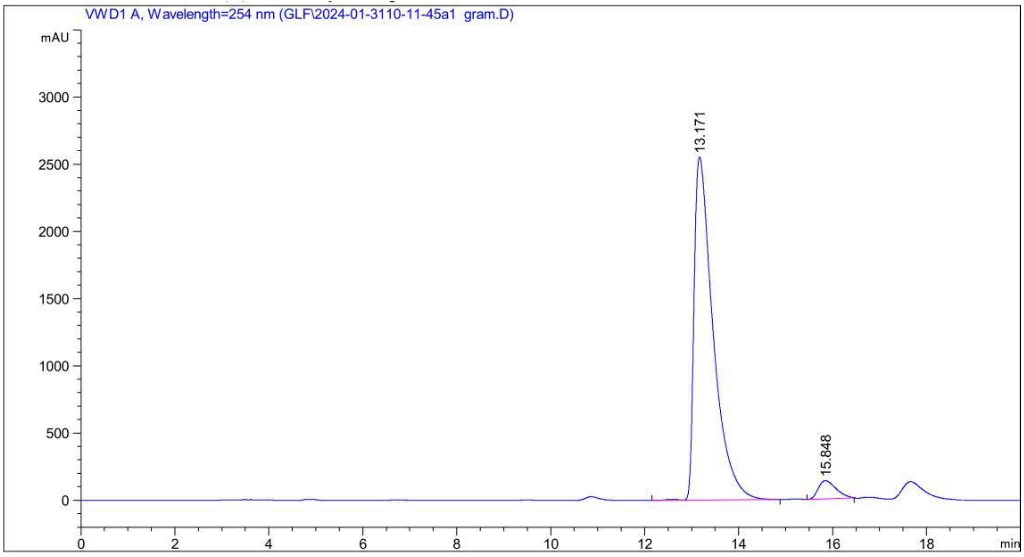

Peak #	RetTime [min]	Type	Width [min]	Area [mAU*s]	Height [mAU]	Area %
1	13.171	VB R	0.4151	7.19555e4	2553.76660	95.3345
2	15.848	BBA	0.4114	3521.35327	134.95117	4.6655

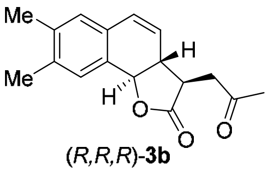

(*R*,*R*,*R*)-**3b**

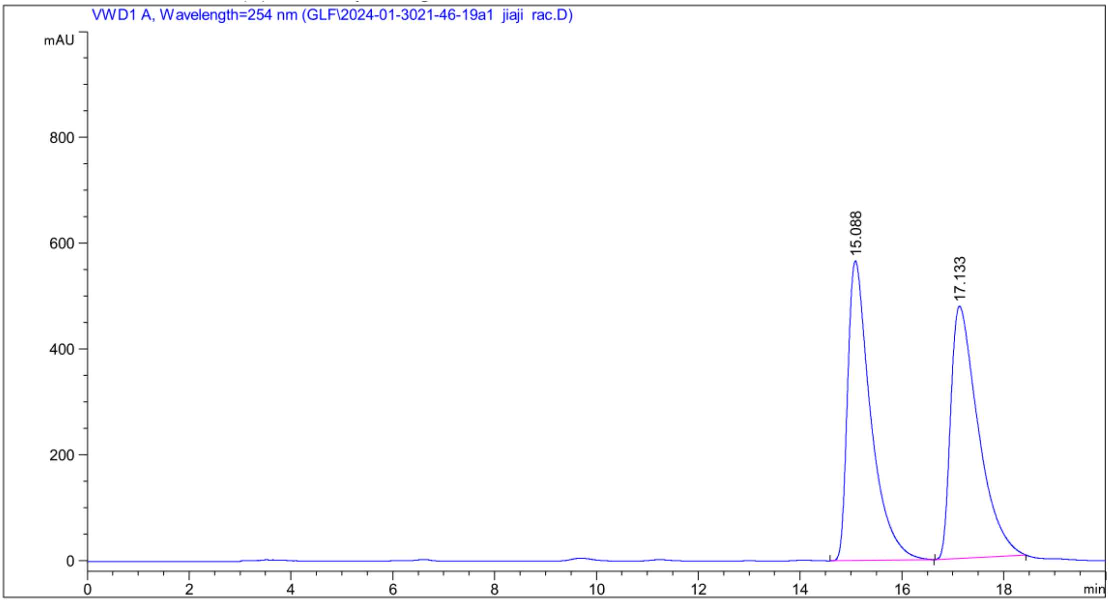

Peak #	RetTime [min]	Type	Width [min]	Area [mAU*s]	Height [mAU]	Area %
1	15.088	BB	0.4644	1.78405e4	566.51367	49.8618
2	17.133	BBA	0.5629	1.79393e4	476.83020	50.1382

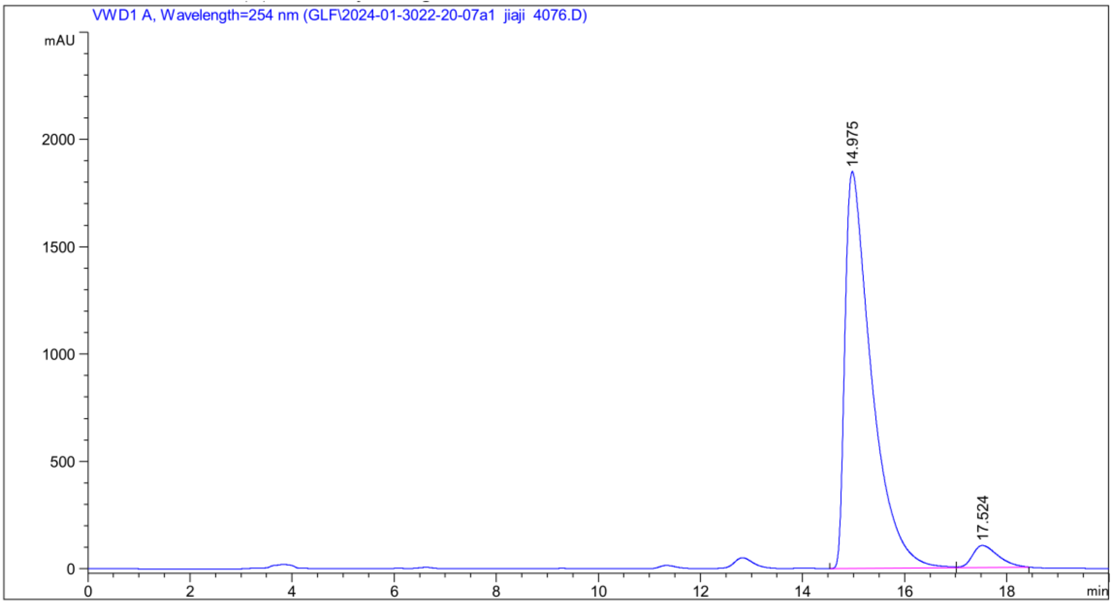

Peak #	RetTime [min]	Type	Width [min]	Area [mAU*s]	Height [mAU]	Area %
1	14.975	BV	0.5206	6.66364e4	1848.37341	94.9169
2	17.524	VBA	0.5317	3568.59790	102.63236	5.0831

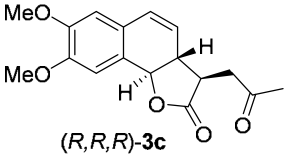

(*R*,*R*,*R*)-**3c**

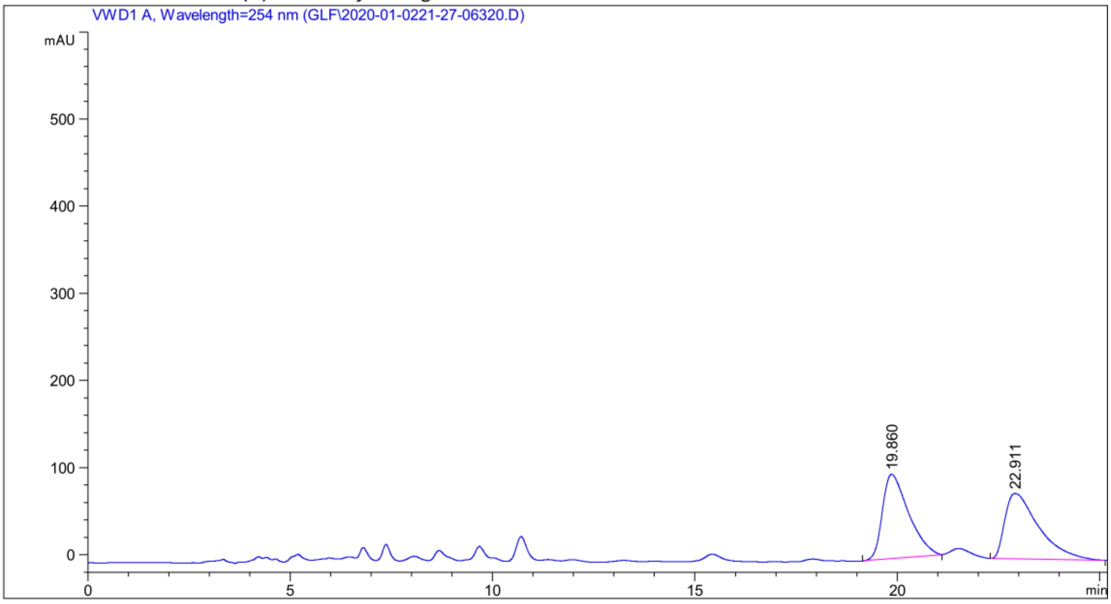

Peak #	RetTime [min]	Type	Width [min]	Area [mAU*s]	Height [mAU]	Area %
1	19.860	BB	0.6935	4343.51660	96.80798	49.7937
2	22.911	BB	0.8339	4379.51025	75.14533	50.2063

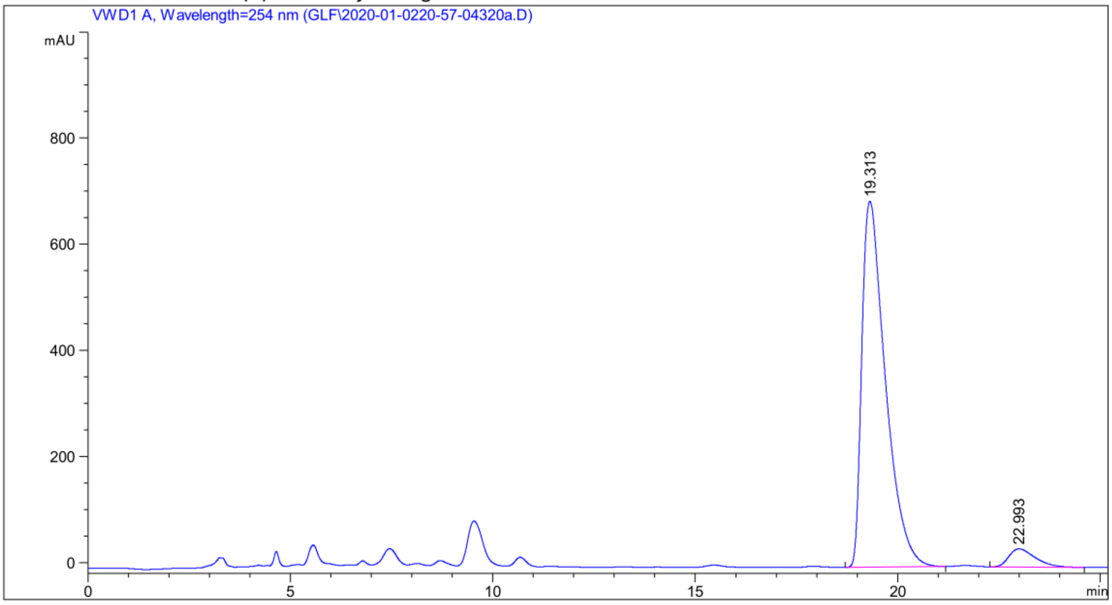

Peak #	RetTime [min]	Type	Width [min]	Area [mAU*s]	Height [mAU]	Area %
1	19.313	BBA	0.5975	2.79359e4	689.74207	94.6962
2	22.993	BB	0.6978	1564.64941	34.25805	5.3038

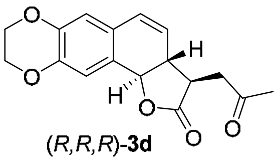

(*R*,*R*,*R*)-**3d**

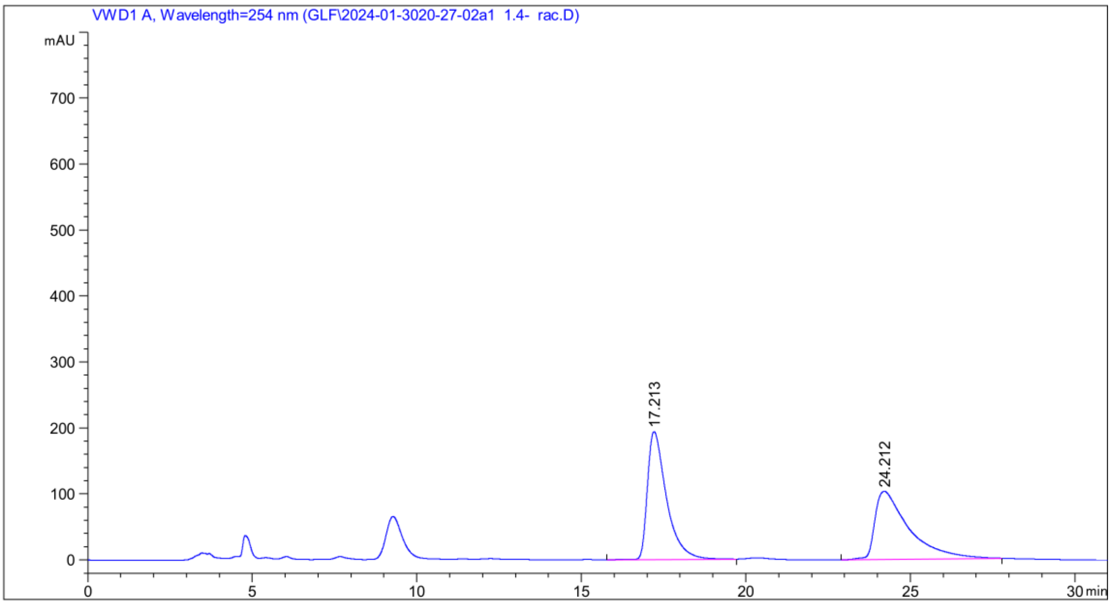

Peak #	RetTime [min]	Type	Width [min]	Area [mAU*s]	Height [mAU]	Area %
1	17.213	BB	0.6032	7816.28809	193.95479	51.2585
2	24.212	BBA	1.0350	7432.47705	103.22648	48.7415

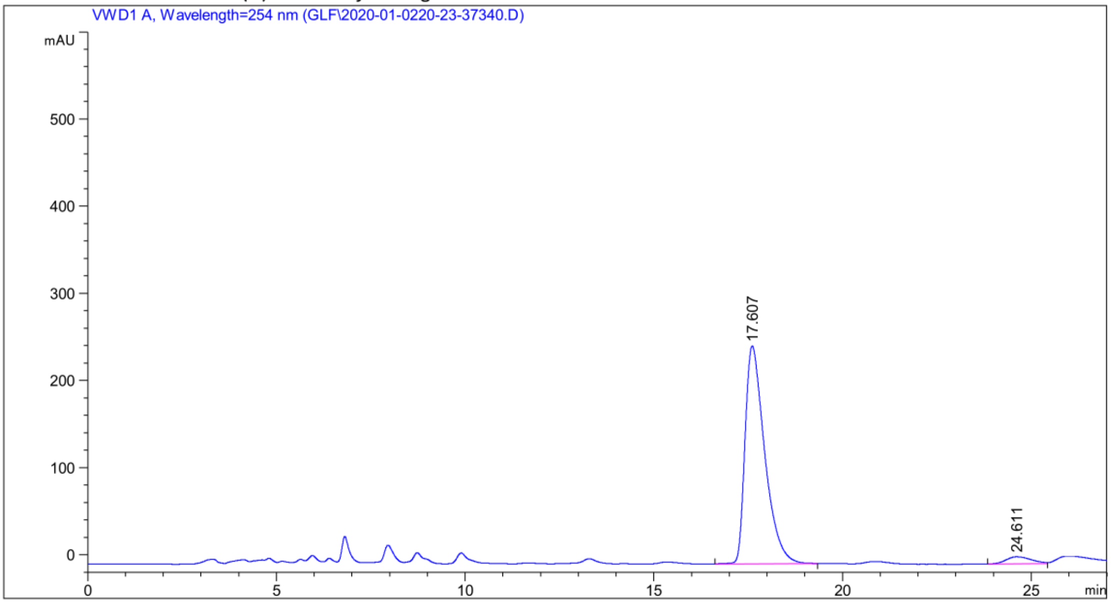

Peak #	RetTime [min]	Type	Width [min]	Area [mAU*s]	Height [mAU]	Area %
1	17.607	BB	0.5594	9029.73145	249.97353	96.1122
2	24.611	BV	0.6993	365.26108	8.00548	3.8878

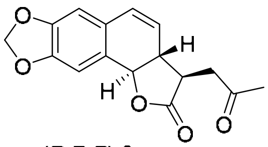

(*R*,*R*,*R*)-**3e**

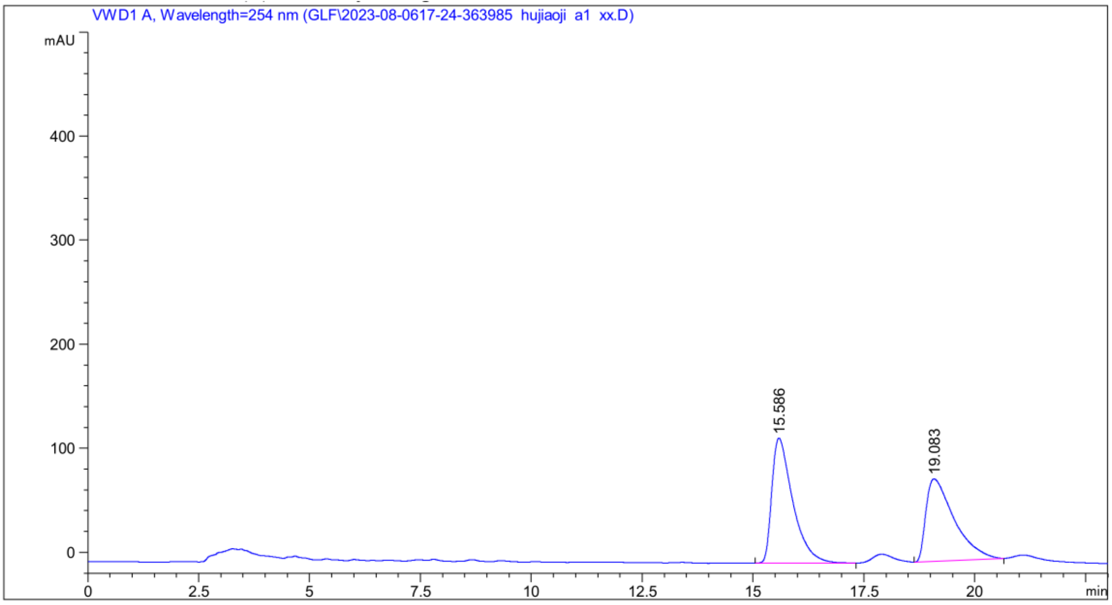

Peak #	RetTime [min]	Type	Width [min]	Area [mAU*s]	Height [mAU]	Area %
1	15.586	BB	0.4878	3952.66553	120.15372	51.0600
2	19.083	BB	0.6407	3496.76392	79.25530	48.9400

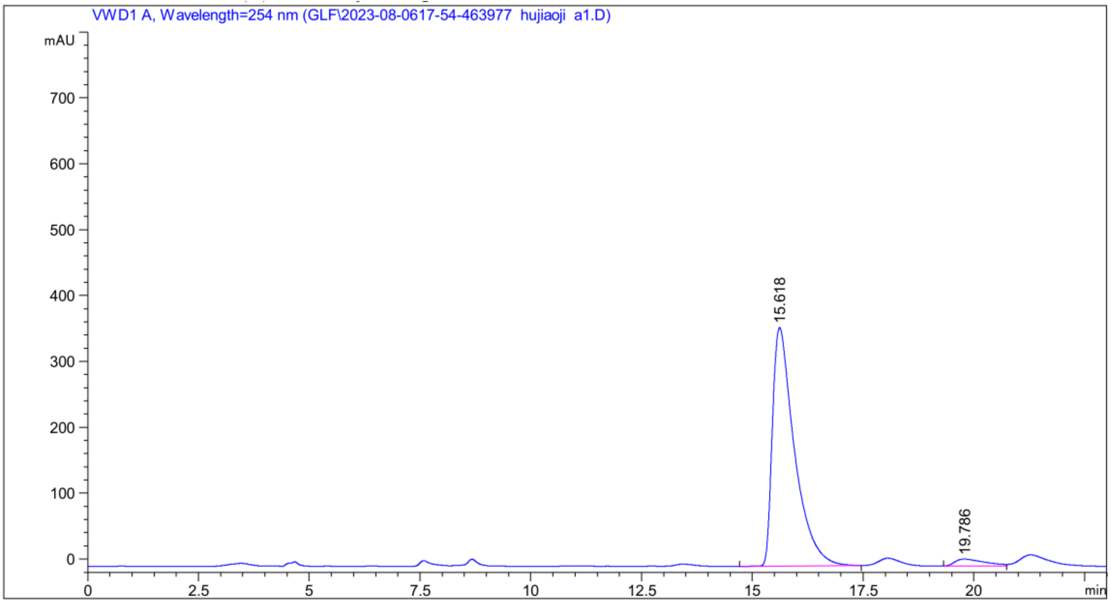

Peak #	RetTime [min]	Type	Width [min]	Area [mAU*s]	Height [mAU]	Area %
1	15.618	BB	0.5125	1.25179e4	361.92499	96.1034
2	19.786	BV	0.6701	507.55212	10.72527	3.8966

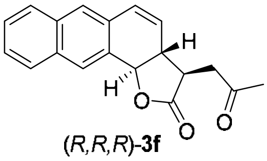

(*R*,*R*,*R*)-**3f**

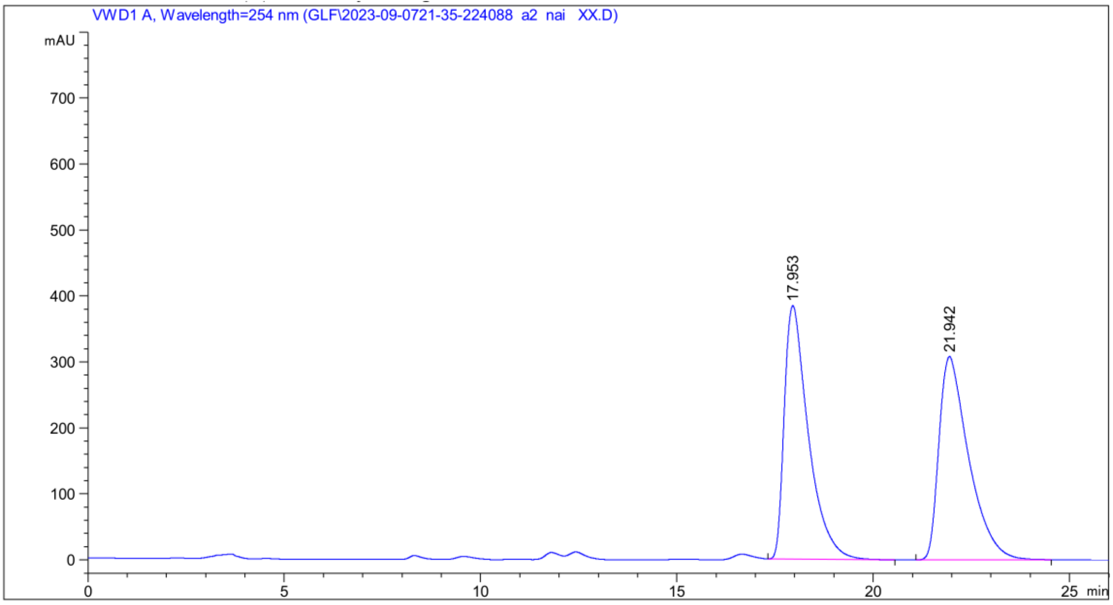

Peak #	RetTime [min]	Type	Width [min]	Area [mAU*s]	Height [mAU]	Area %
1	17.953	BB	0.6312	1.64008e4	384.06769	50.3130
2	21.942	BB	0.7932	1.61967e4	308.13519	49.6870

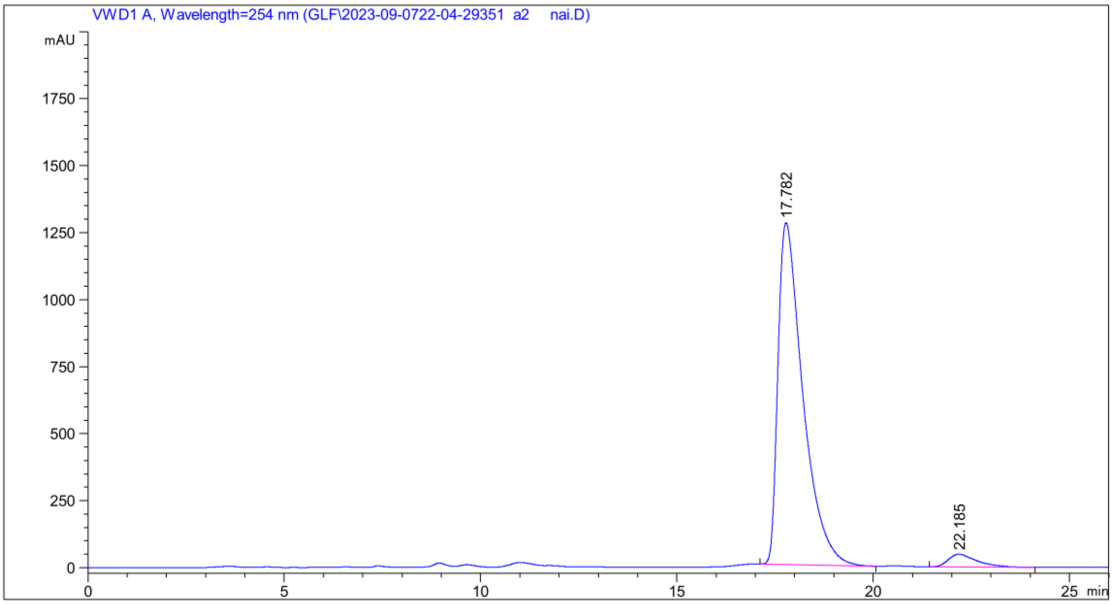

Peak #	RetTime [min]	Type	Width [min]	Area [mAU*s]	Height [mAU]	Area %
1	17.782	VB R	0.6729	5.73954e4	1283.37646	95.9614
2	22.185	BB	0.7391	2415.49951	47.51360	4.0386

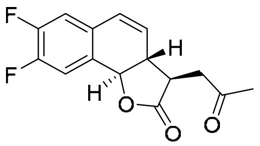

(*R*,*R*,*R*)-**3g**

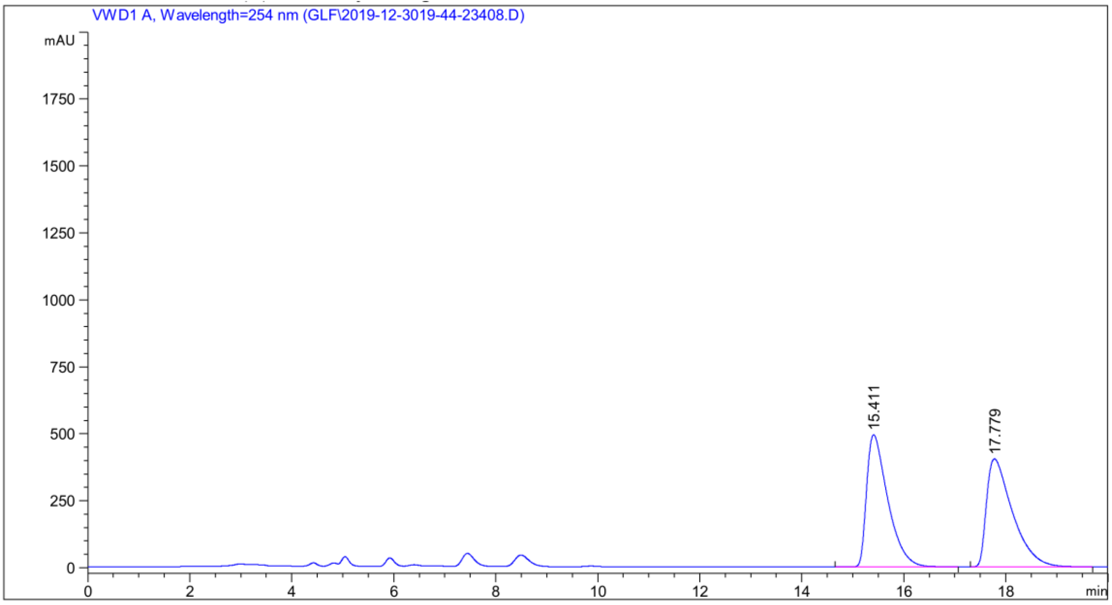

Peak #	RetTime [min]	Type	Width [min]	Area [mAU*s]	Height [mAU]	Area %
1	15.411	VB R	0.4312	1.42697e4	493.71988	49.9553
2	17.779	BB	0.5286	1.42953e4	403.19778	50.0447

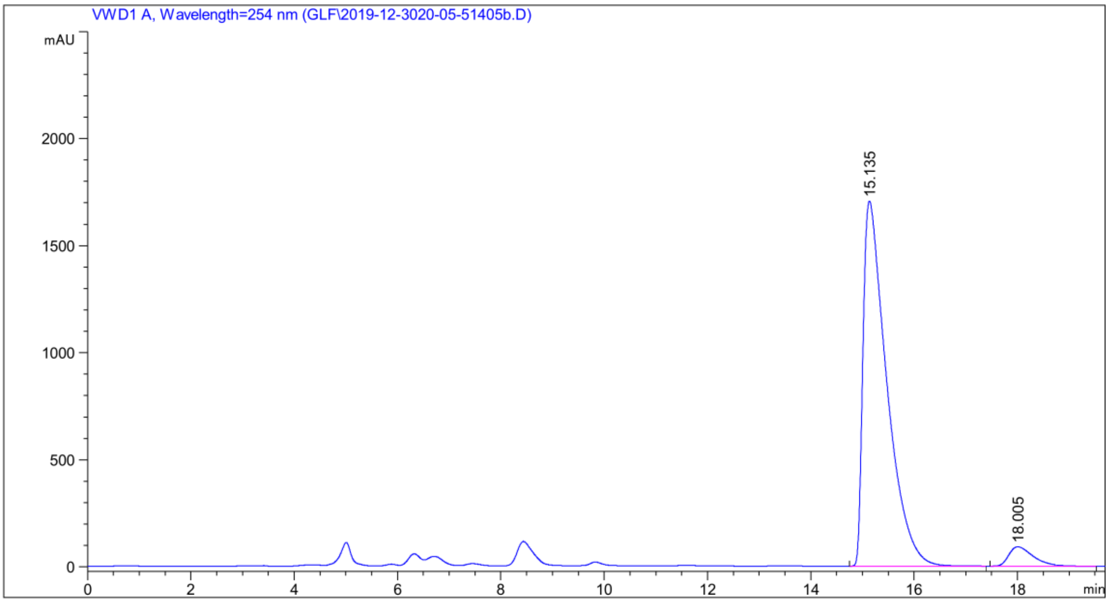

Peak #	RetTime [min]	Type	Width [min]	Area [mAU*s]	Height [mAU]	Area %
1	15.135	BB	0.4624	5.42620e4	1704.73267	94.8186
2	18.005	BB	0.5008	2965.13916	90.12718	5.1814

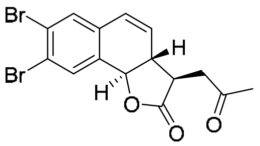

(*R*,*R*,*R*)-**3h**

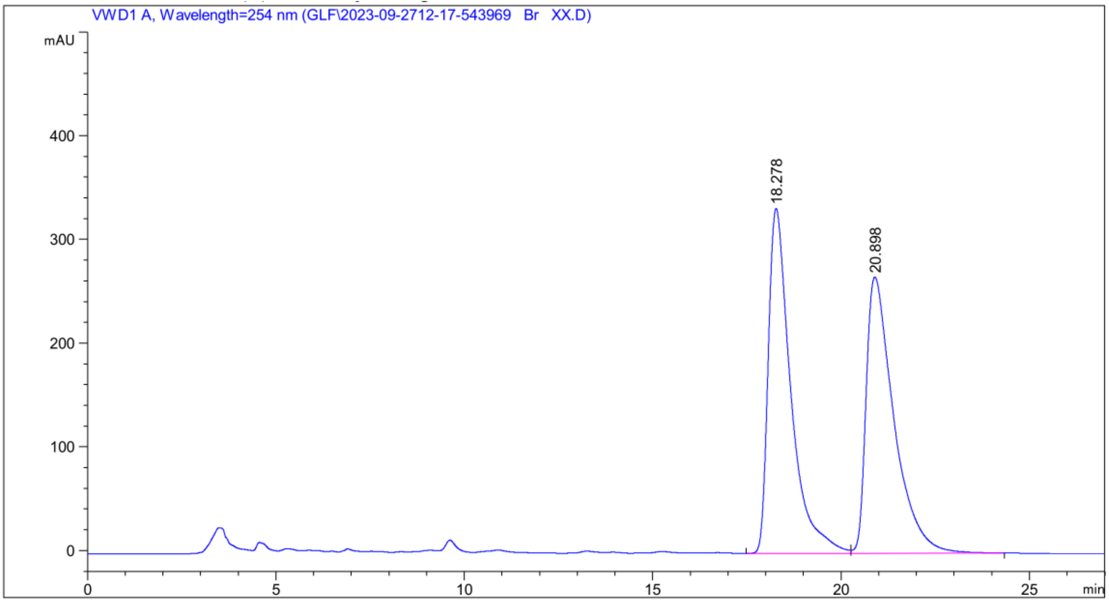

Peak #	RetTime [min]	Type	Width [min]	Area [mAU*s]	Height [mAU]	Area %
1	18.278	BV	0.6187	1.38761e4	332.63184	50.5339
2	20.898	VB	0.7451	1.35828e4	266.28275	49.4661

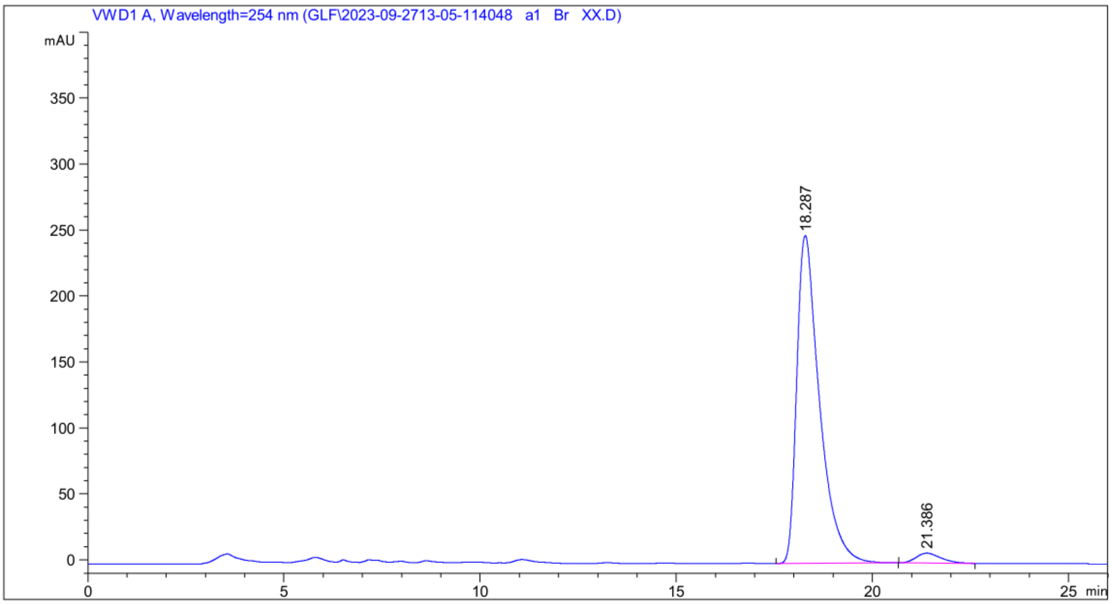

Peak #	RetTime [min]	Type	Width [min]	Area [mAU*s]	Height [mAU]	Area %
1	18.287	BB	0.5891	1.00078e4	248.37762	96.8121
2	21.386	BB	0.6250	329.54251	7.58247	3.1879

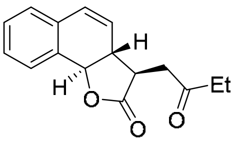

(*R*,*R*,*R*)-**3i**

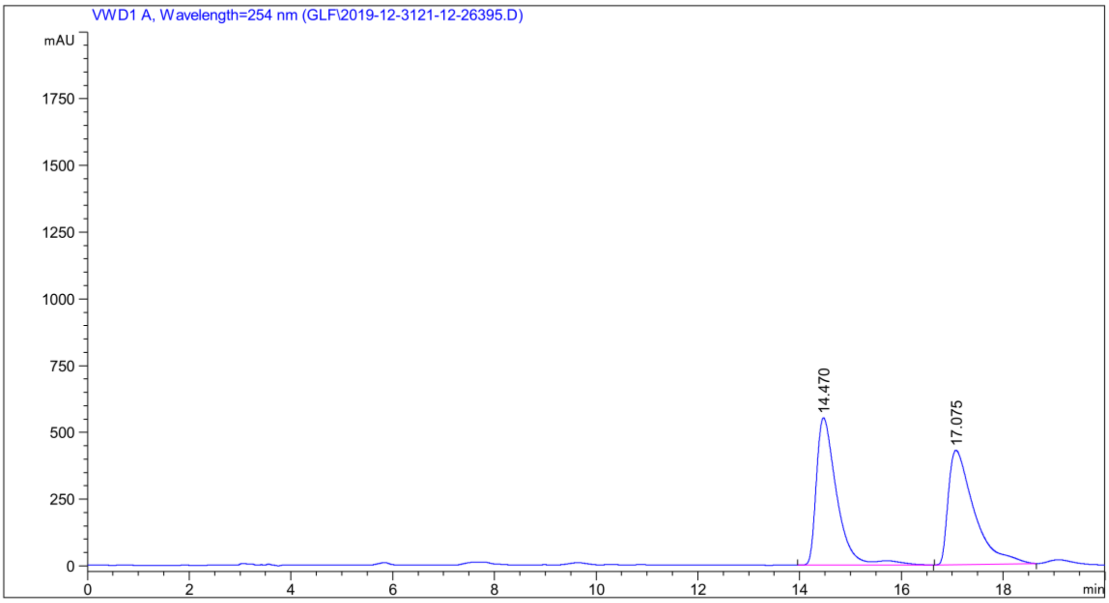

Peak #	RetTime [min]	Type	Width [min]	Area [mAU*s]	Height [mAU]	Area %
1	14.470	BV R	0.4075	1.54567e4	551.86664	50.6783
2	17.075	BBA	0.5219	1.50430e4	428.14230	49.3217

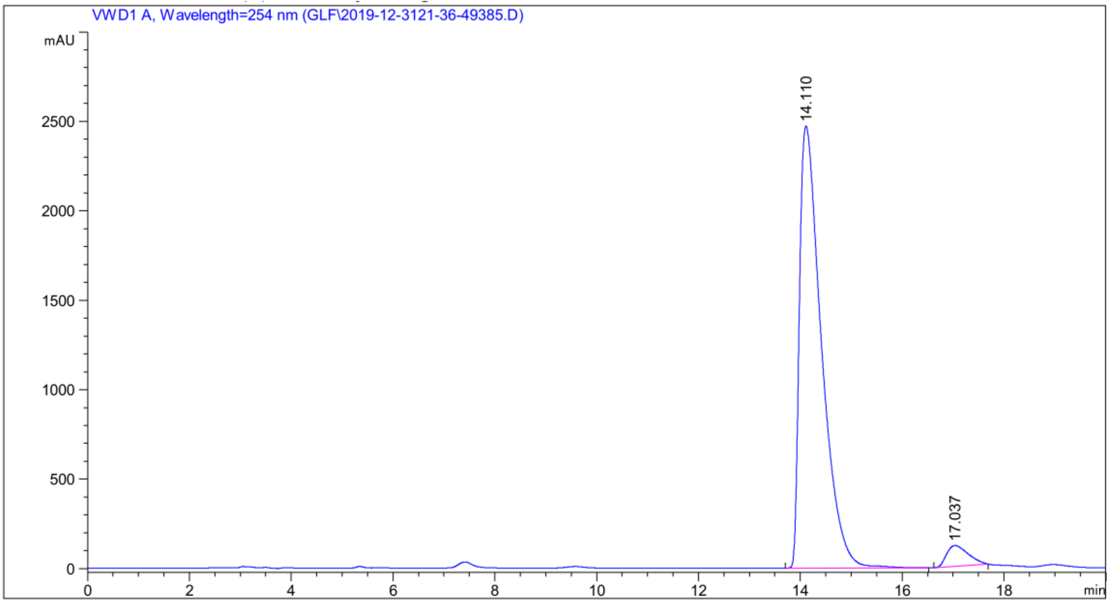

Peak #	RetTime [min]	Type	Width [min]	Area [mAU*s]	Height [mAU]	Area %
1	14.110	BB	0.4503	7.39885e4	2470.89868	95.5700
2	17.037	BBA	0.4653	3429.59985	116.22460	4.4300

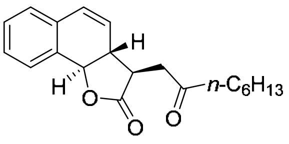

(*R*,*R*,*R*)-**3j**

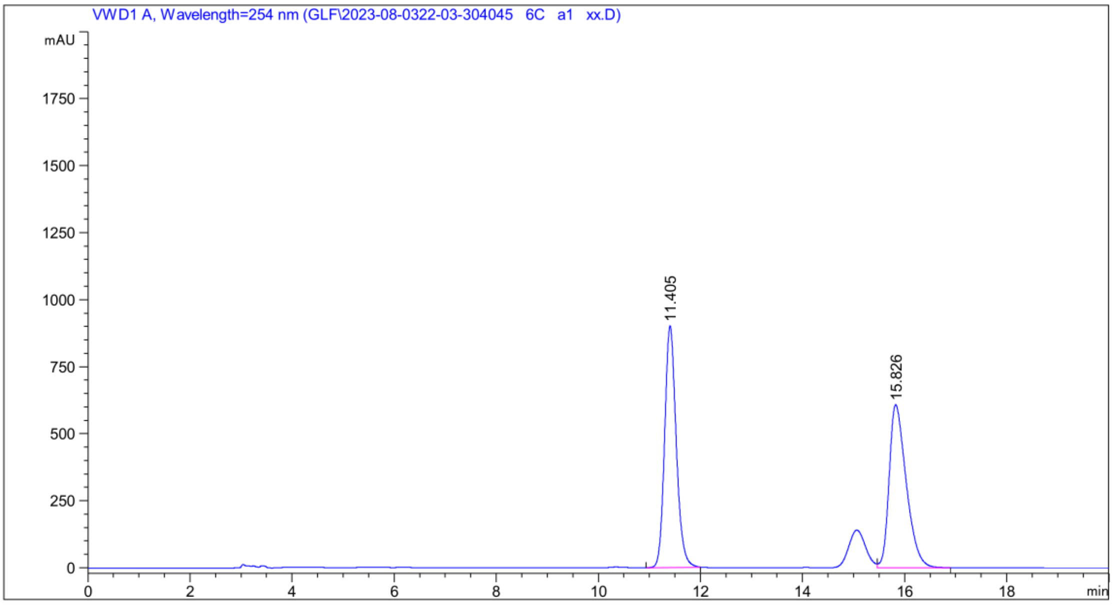

Peak #	RetTime [min]	Type	Width [min]	Area [mAU*s]	Height [mAU]	Area %
1	11.405	BBA	0.2416	1.41894e4	901.59021	50.0913
2	15.826	VB	0.3563	1.41377e4	608.05292	49.9087

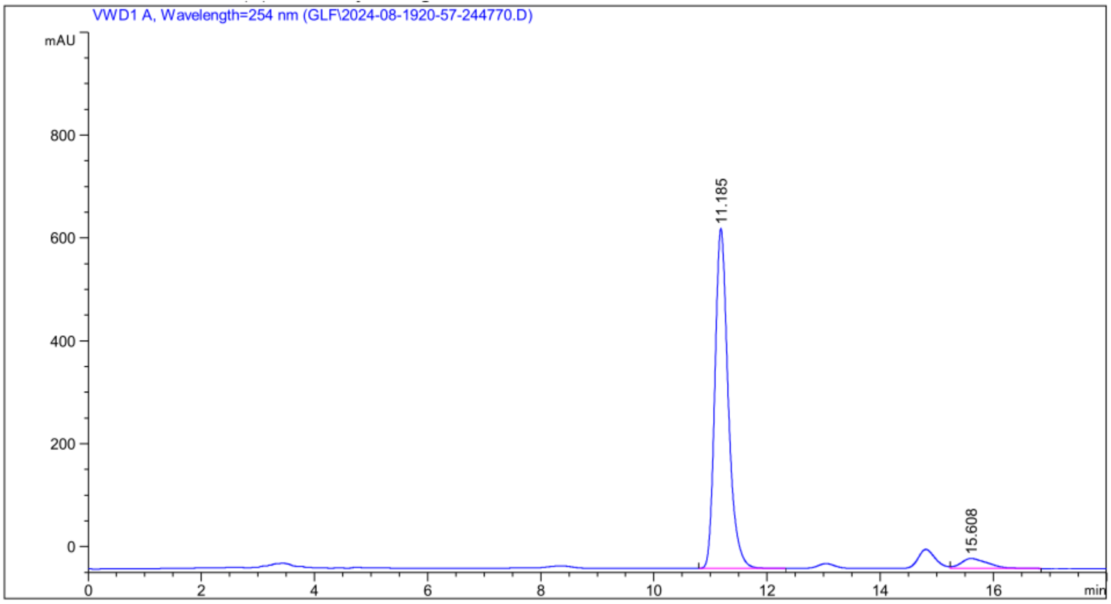

Peak #	RetTime [min]	Type	Width [min]	Area [mAU*s]	Height [mAU]	Area %
1	11.185	BB	0.2506	1.07907e4	660.61005	94.3161
2	15.608	VB	0.4738	650.30042	19.66288	5.6839

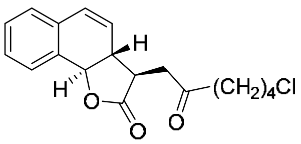

(*R*,*R*,*R*)-**3k**

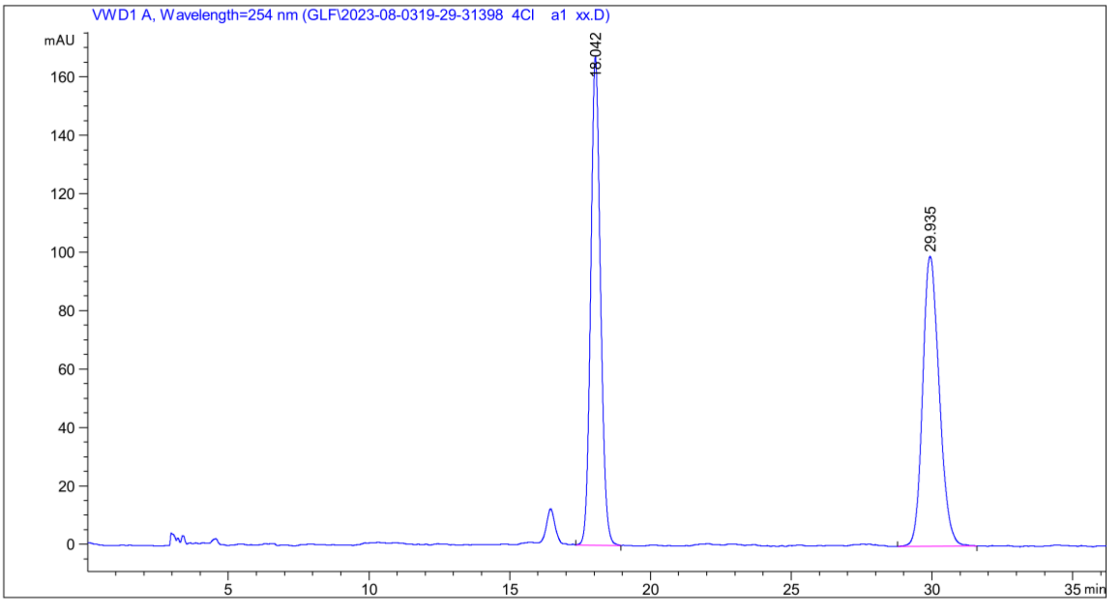

Peak #	RetTime [min]	Type	Width [min]	Area [mAU*s]	Height [mAU]	Area %
1	18.042	BB	0.3675	4035.97314	167.24492	49.7586
2	29.935	BB	0.6372	4075.12988	99.21137	50.2414

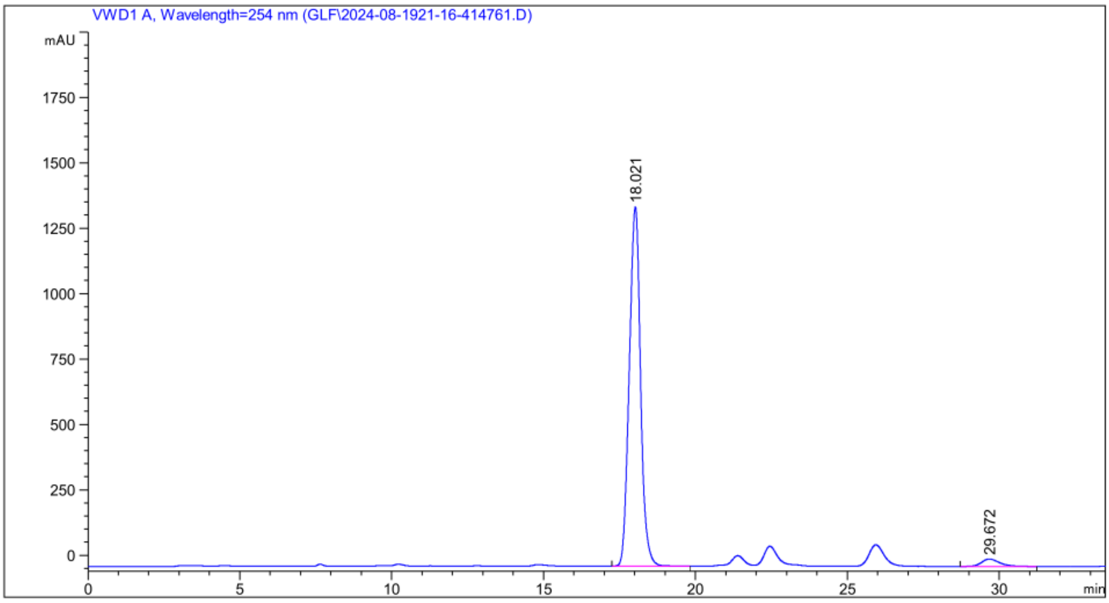

Peak #	RetTime [min]	Type	Width [min]	Area [mAU*s]	Height [mAU]	Area %
1	18.021	BB	0.3977	3.53767e4	1372.37109	96.8333
2	29.672	BB	0.6123	1156.91223	28.22020	3.1667

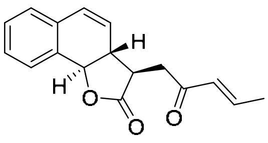

(*R*,*R*,*R*)-**3l**

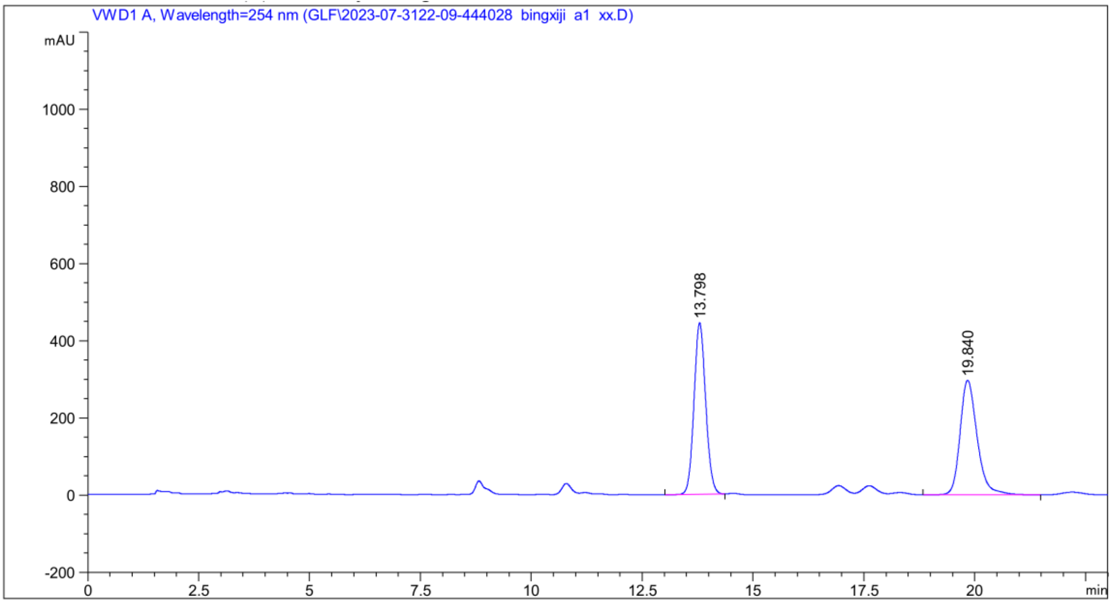

Peak #	RetTime [min]	Type	Width [min]	Area [mAU*s]	Height [mAU]	Area %
1	13.798	BB	0.2825	8131.71582	444.39746	50.5505
2	19.840	BB	0.4055	7954.60645	296.96661	49.4495

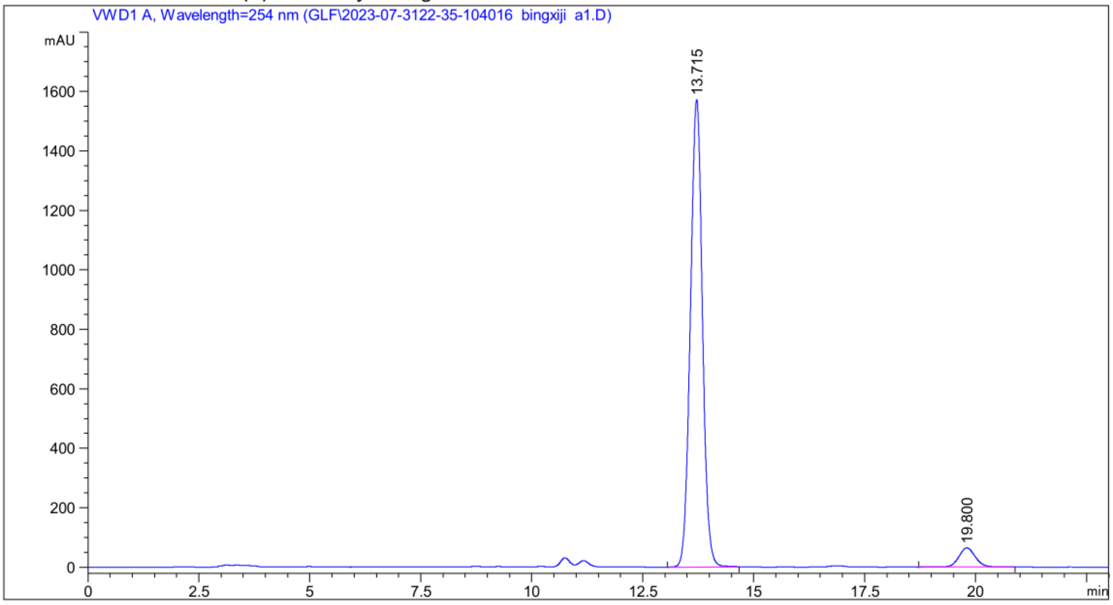

Peak #	RetTime [min]	Type	Width [min]	Area [mAU*s]	Height [mAU]	Area %
1	13.715	BBA	0.2941	2.96646e4	1572.15454	94.6519
2	19.800	BB	0.3986	1676.13782	64.61974	5.3481

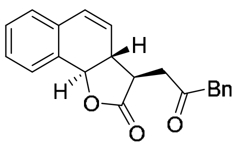

(*R*,*R*,*R*)-**3m**

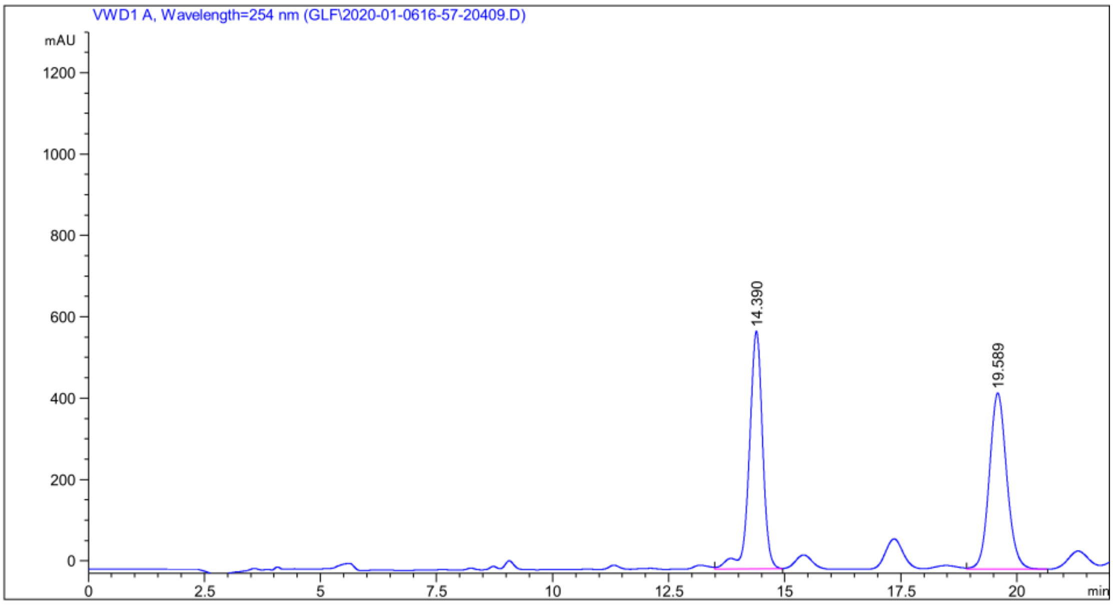

Peak #	RetTime [min]	Type	Width [min]	Area [mAU*s]	Height [mAU]	Area %
1	14.390	FM	0.3318	1.16168e4	583.56964	50.4822
2	19.589	VB	0.4061	1.13949e4	432.90631	49.5178

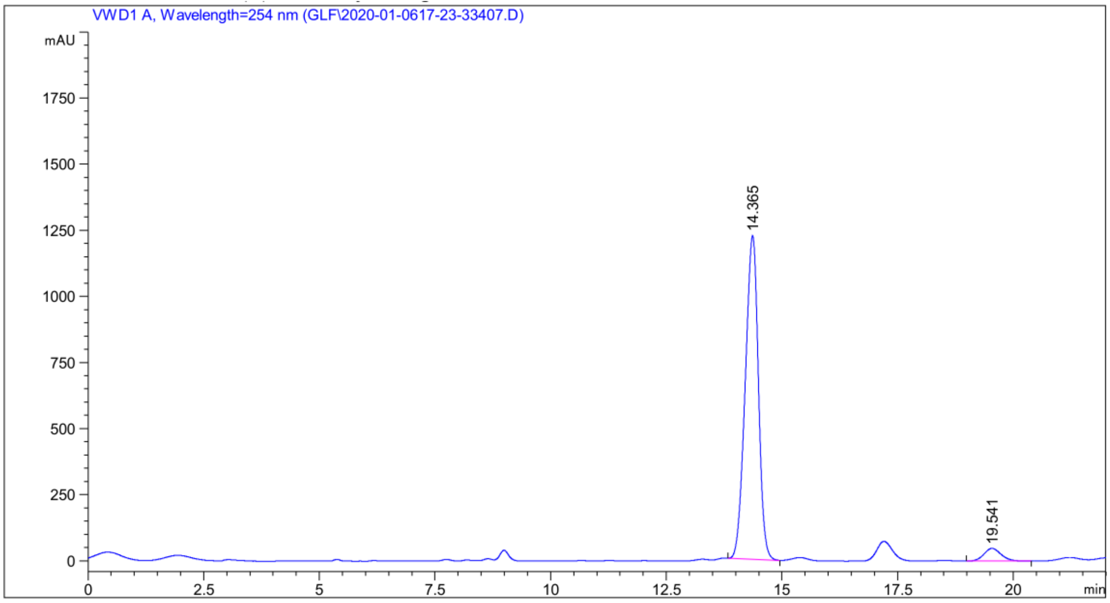

Peak #	RetTime [min]	Type	Width [min]	Area [mAU*s]	Height [mAU]	Area %
1	14.365	BB	0.3055	2.40758e4	1223.87354	95.0918
2	19.541	BB	0.3965	1242.67444	48.09024	4.9082

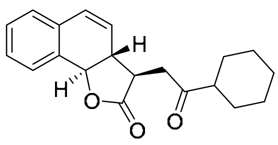

(*R*,*R*,*R*)-**3n**

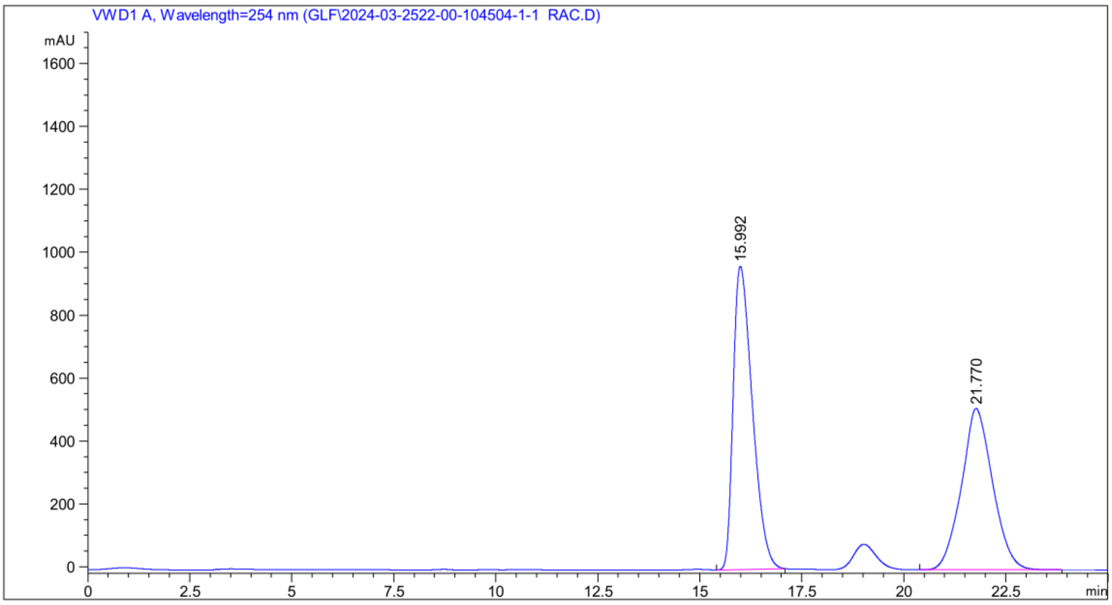

Peak #	RetTime [min]	Type	Width [min]	Area [mAU*s]	Height [mAU]	Area %
1	15.992	BBA	0.5081	3.14871e4	963.89435	51.5244
2	21.770	BB	0.7876	2.73404e4	513.15039	49.4756

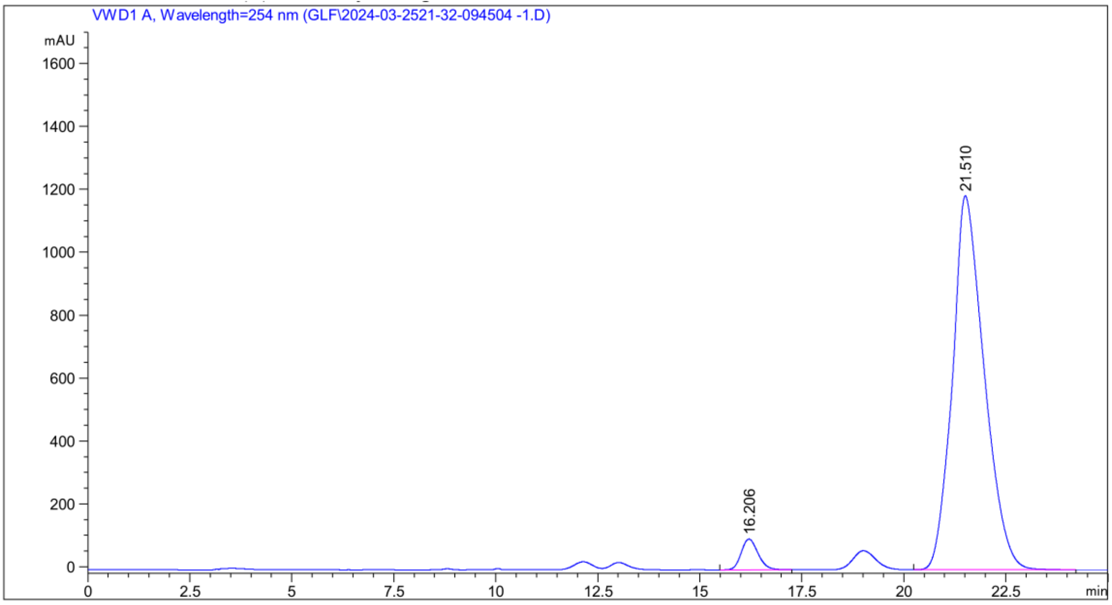

Peak #	RetTime [min]	Type	Width [min]	Area [mAU*s]	Height [mAU]	Area %
1	16.206	BB	0.4437	2793.60449	97.67126	4.1584
2	21.510	BB	0.7918	6.43867e4	1188.94397	95.8416

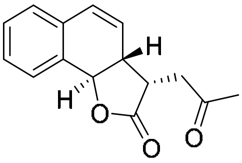

(*S*,*R*,*R*)-**4a**

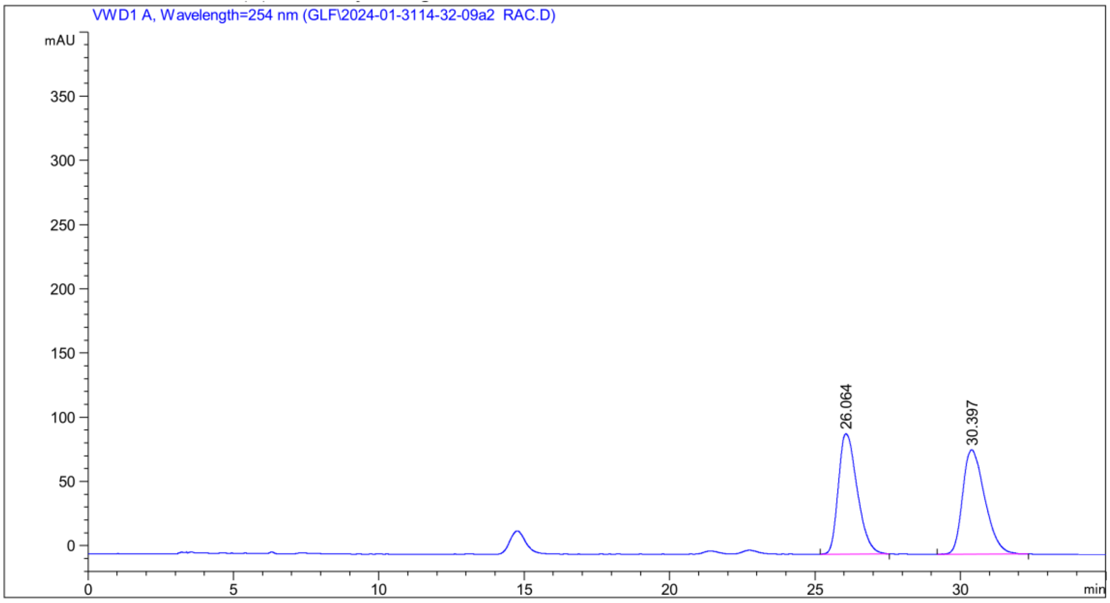

Peak #	RetTime [min]	Type	Width [min]	Area [mAU*s]	Height [mAU]	Area %
1	26.064	BB	0.6815	4242.12207	93.64829	49.4446
2	30.397	BV R	0.8131	4337.42383	81.46808	50.5554

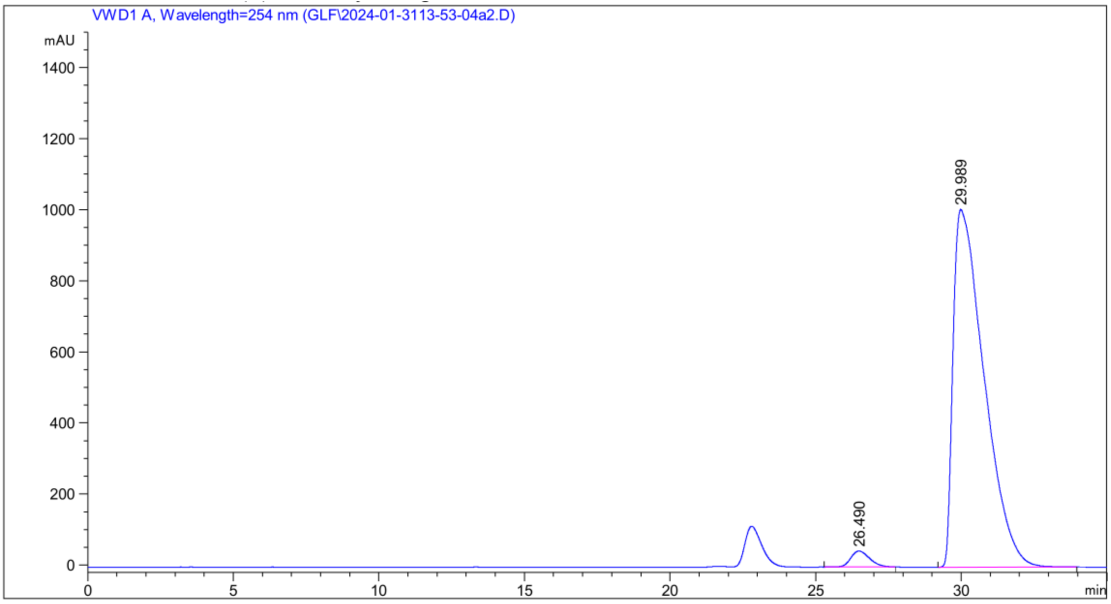

Peak #	RetTime [min]	Type	Width [min]	Area [mAU*s]	Height [mAU]	Area %
1	26.490	BB	0.6882	2031.70007	44.87866	2.6608
2	29.989	BB	1.0817	7.43253e4	1006.30078	97.3392

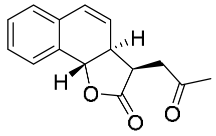

(R,S,S)-**4a**

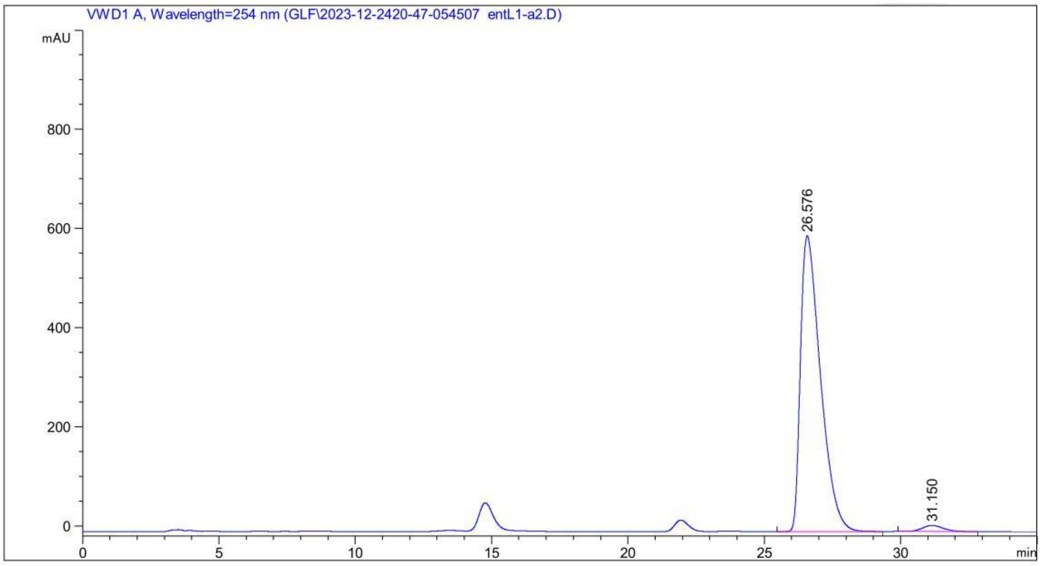

Peak #	RetTime [min]	Type	Width [min]	Area [mAU*s]	Height [mAU]	Area %
1	26.576	BB	0.7870	3.04363e4	596.79474	97.8642
2	31.150	BB	0.8203	664.23163	12.33436	2.1358

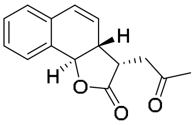

Gram-scale reaction of
(S,R,R)-**4a**

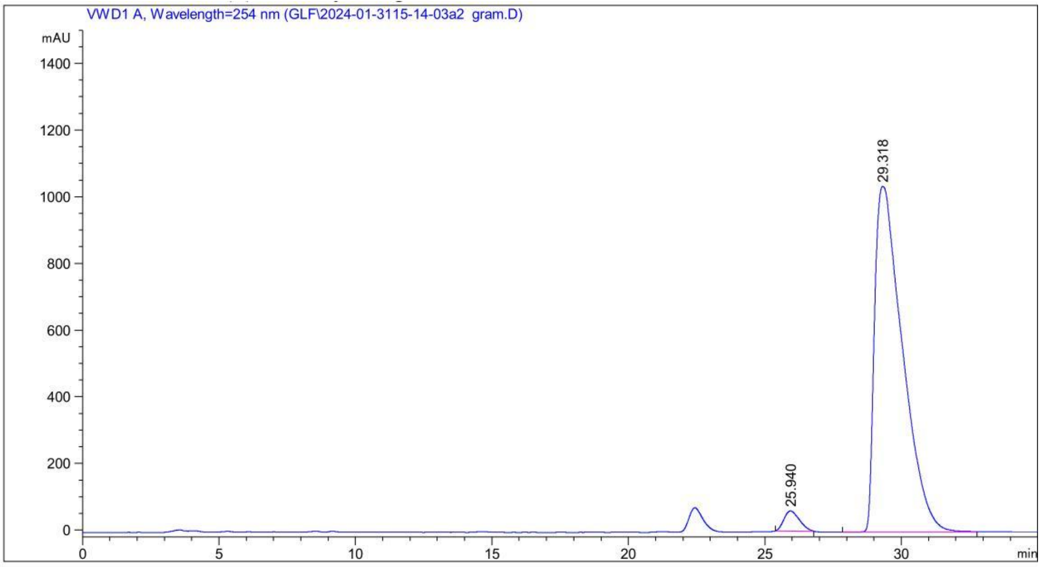

Peak #	RetTime [min]	Type	Width [min]	Area [mAU*s]	Height [mAU]	Area %
1	25.940	HBA	0.6242	2354.52246	60.33988	3.1016
2	29.318	HH	1.0055	7.35591e4	1037.08557	96.8984

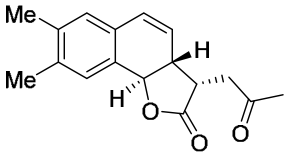

(*S*,*R*,*R*)-**4b**

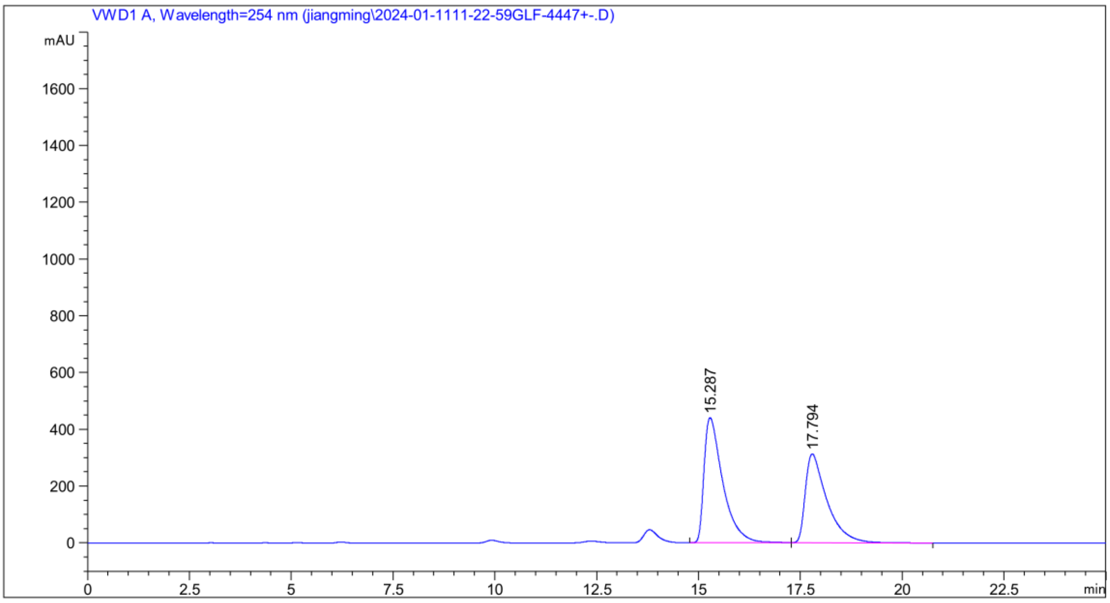

Peak #	RetTime [min]	Type	Width [min]	Area [mAU*s]	Height [mAU]	Area %
1	15.287	BV	0.4773	1.41491e4	441.03333	52.5846
2	17.794	VB	0.5359	1.15837e4	313.66385	47.4154

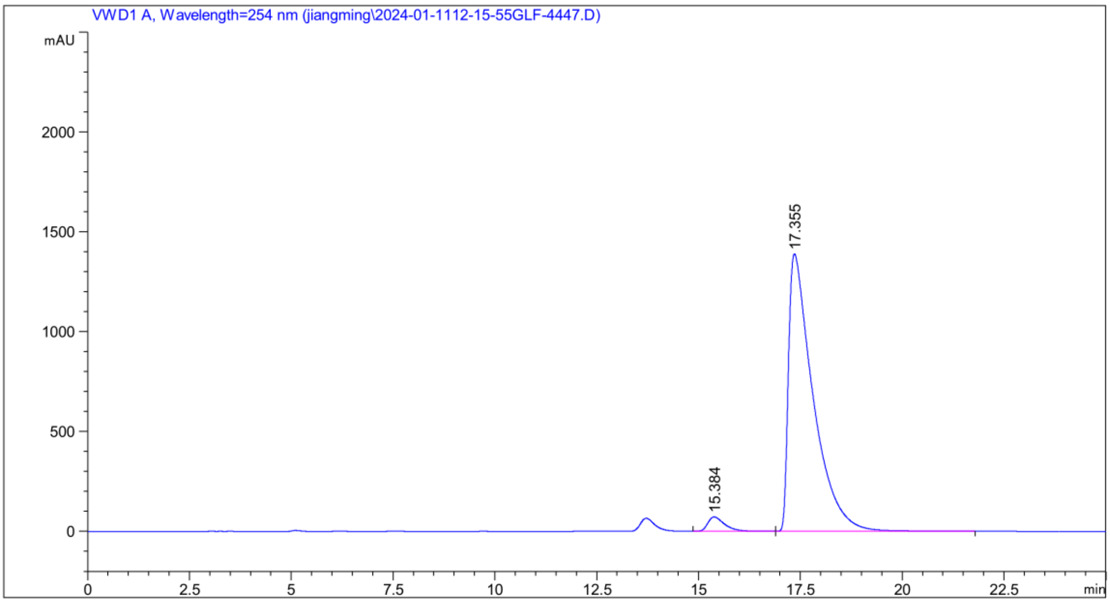

Peak #	RetTime [min]	Type	Width [min]	Area [mAU*s]	Height [mAU]	Area %
1	15.384	BB	0.4353	2104.88184	72.37424	3.4857
2	17.355	BB	0.6065	5.82814e4	1389.19360	96.5143

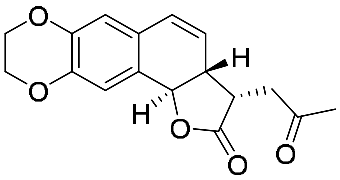

(*S*,*R*,*R*)-**4d**

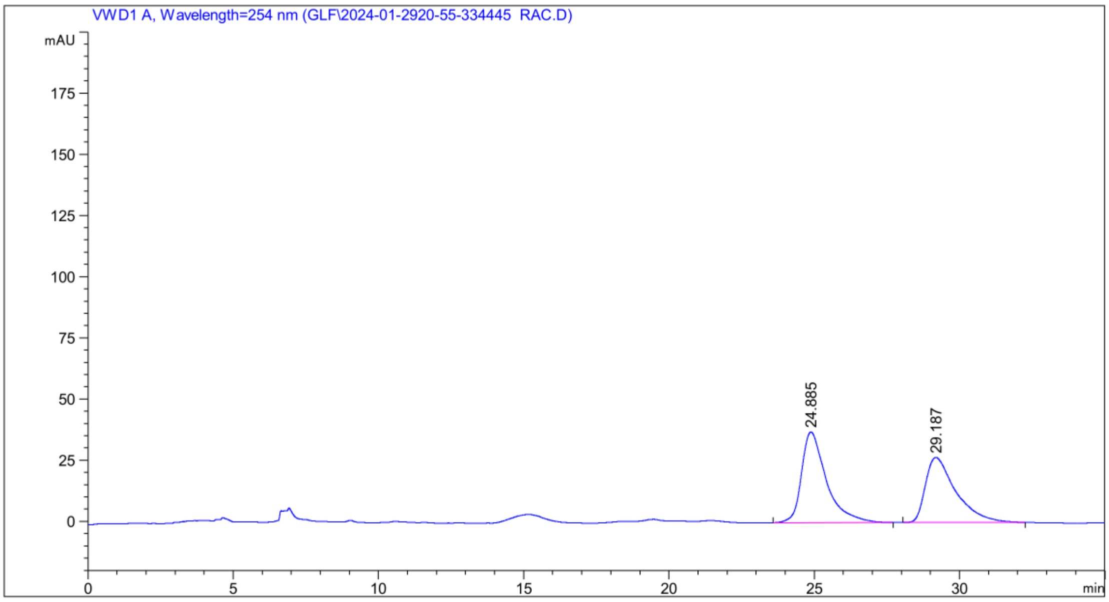

Peak #	RetTime [min]	Type	Width [min]	Area [mAU*s]	Height [mAU]	Area %
1	24.885	BB	0.8780	2210.00024	36.99292	50.2801
2	29.187	BB	1.0680	1937.89343	26.66140	49.7199

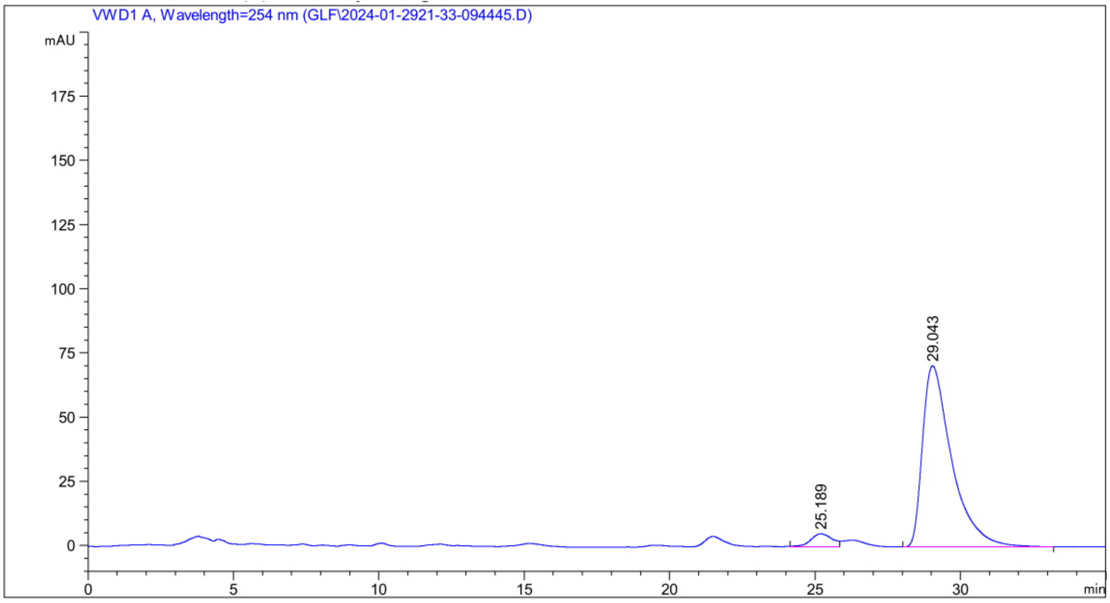

Peak #	RetTime [min]	Type	Width [min]	Area [mAU*s]	Height [mAU]	Area %
1	25.189	BB	0.5718	149.67645	3.73314	2.9465
2	29.043	BB	1.0463	4930.14111	70.54834	97.0535

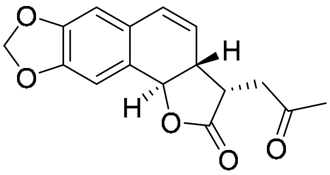

(*S*,*R*,*R*)-**4e**

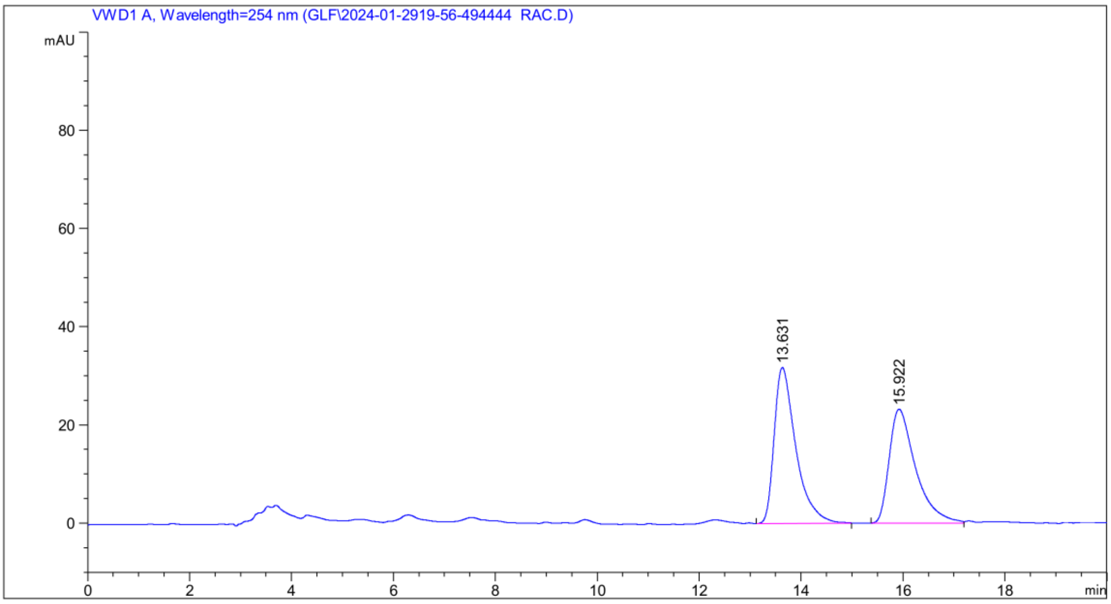

Peak #	RetTime [min]	Type	Width [min]	Area [mAU*s]	Height [mAU]	Area %
1	13.631	BBA	0.4408	938.40216	31.74929	50.9179
2	15.922	HH	0.5363	834.91632	23.17597	49.0821

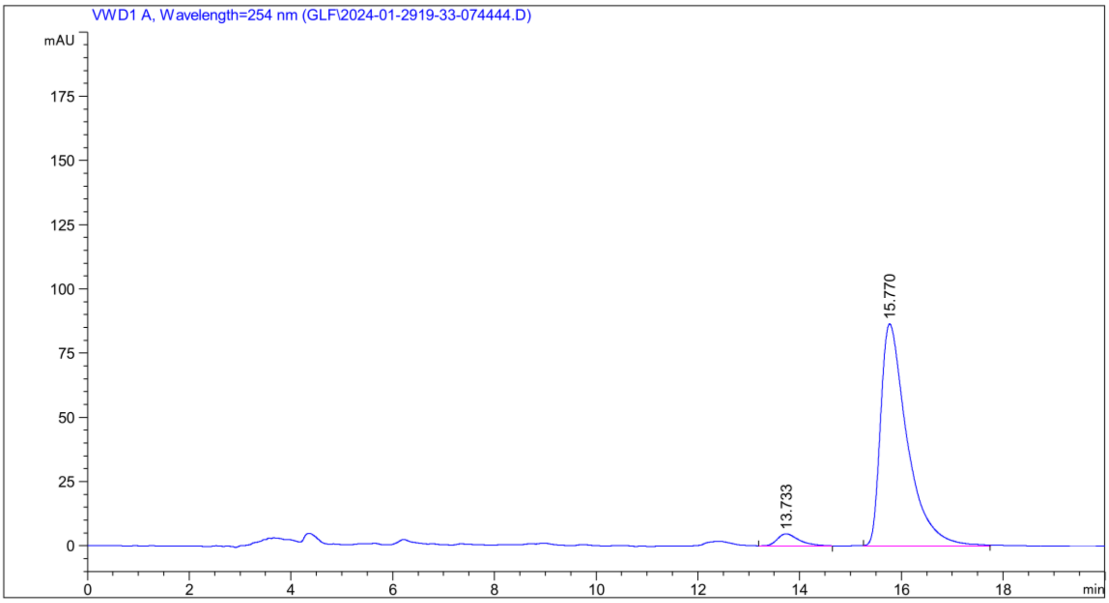

Peak #	RetTime [min]	Type	Width [min]	Area [mAU*s]	Height [mAU]	Area %
1	13.733	BB	0.4397	137.74564	4.67572	4.2044
2	15.770	HH	0.5396	3138.47998	86.43583	95.7956

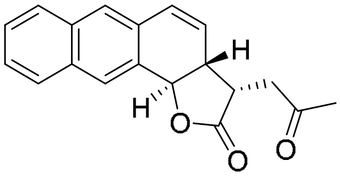

(*S*,*R*,*R*)-**4f**

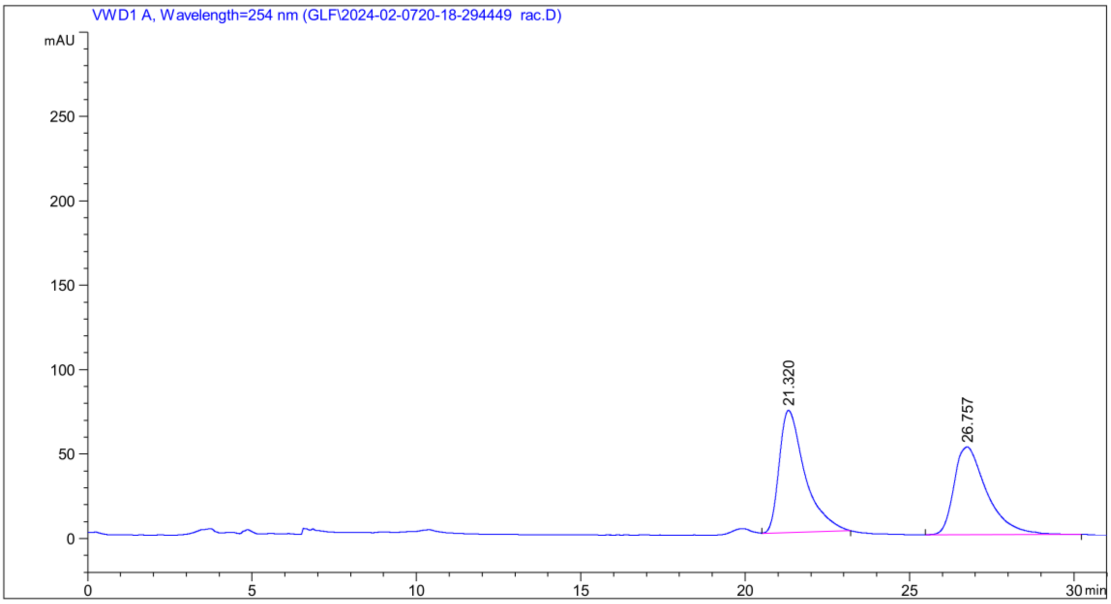

Peak #	RetTime [min]	Type	Width [min]	Area [mAU*s]	Height [mAU]	Area %
1	21.320	BBA	0.8109	3935.29443	72.31099	50.0895
2	26.757	BB	0.9946	3619.57886	51.96527	49.9105

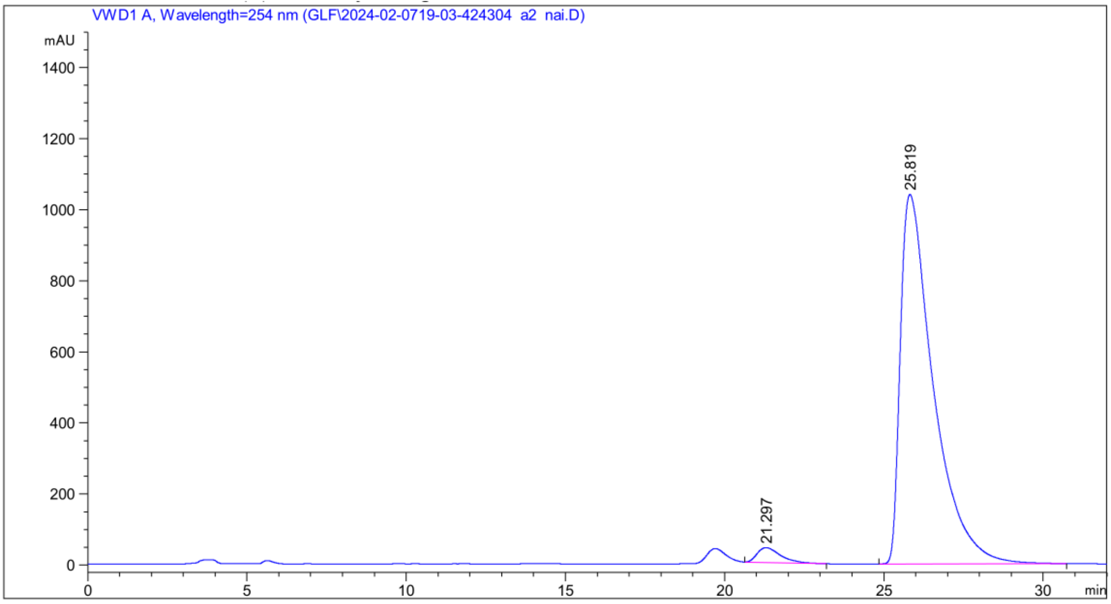

Peak #	RetTime [min]	Type	Width [min]	Area [mAU*s]	Height [mAU]	Area %
1	21.297	BBA	0.7888	2127.41431	41.72719	2.7676
2	25.819	BB	1.0520	7.47421e4	1039.36316	97.2324

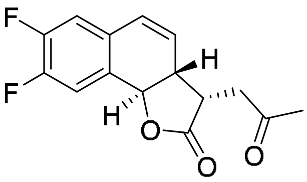

(*S*,*R*,*R*)-**4g**

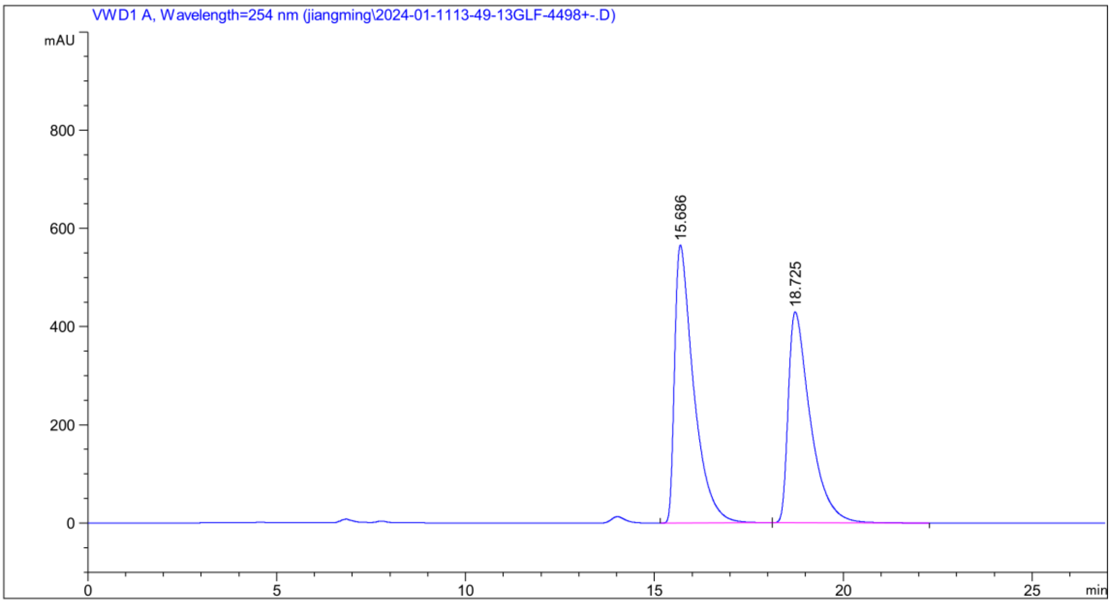

Peak #	RetTime [min]	Type	Width [min]	Area [mAU*s]	Height [mAU]	Area %
1	15.686	BB	0.5253	1.98731e4	566.48511	52.2398
2	18.725	BB	0.5990	1.74545e4	429.58163	47.7602

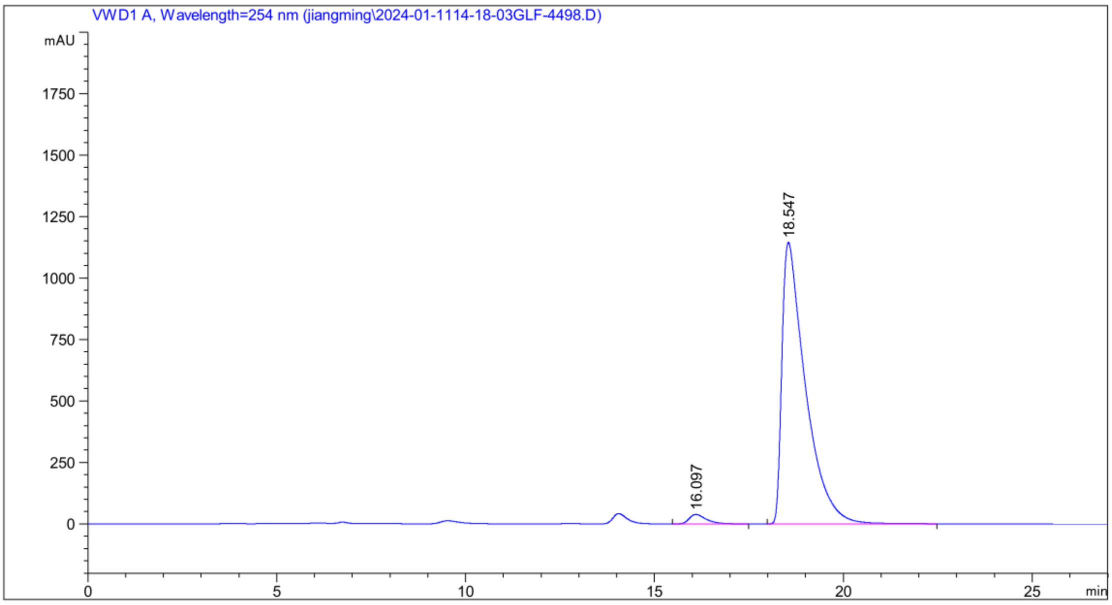

Peak #	RetTime [min]	Type	Width [min]	Area [mAU*s]	Height [mAU]	Area %
1	16.097	BB	0.4716	1212.55603	38.58886	2.3880
2	18.547	BB	0.6183	4.95636e4	1144.35962	97.6120

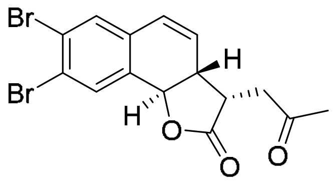

(*S*,*R*,*R*)-**4h**

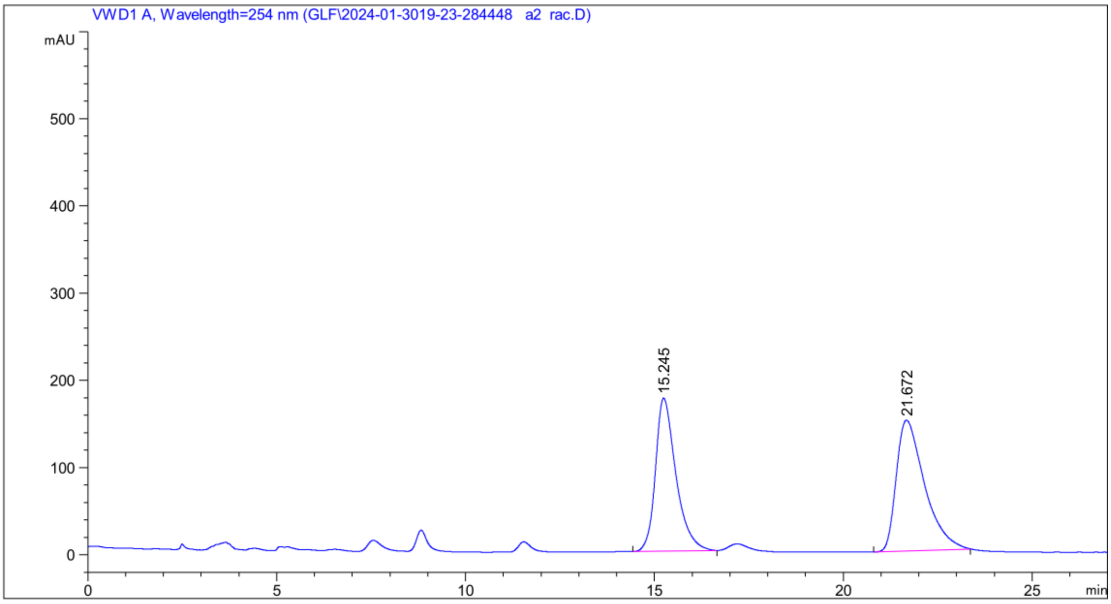

Peak #	RetTime [min]	Type	Width [min]	Area [mAU*s]	Height [mAU]	Area %
1	15.245	BB	0.5671	6619.77197	175.46933	47.9278
2	21.672	BBA	0.7694	7793.65234	149.95270	52.0722

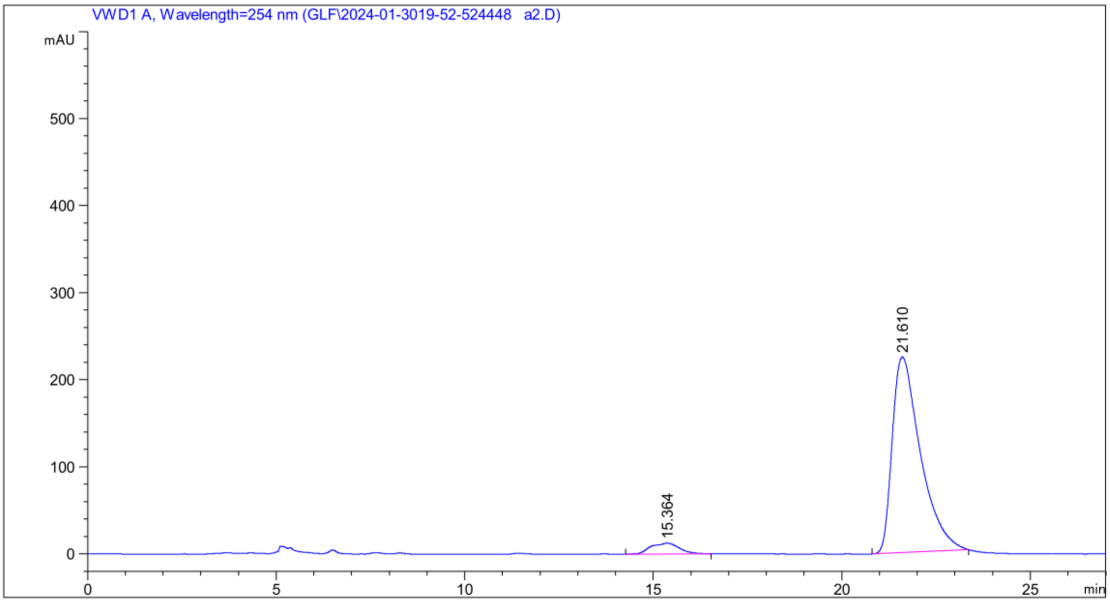

Peak #	RetTime [min]	Type	Width [min]	Area [mAU*s]	Height [mAU]	Area %
1	15.364	BB	0.7201	643.37189	12.32352	5.2100
2	21.610	BBA	0.7597	1.17055e4	224.72862	94.7900

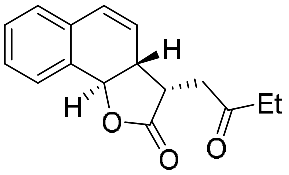

(*S*,*R*,*R*)-**4i**

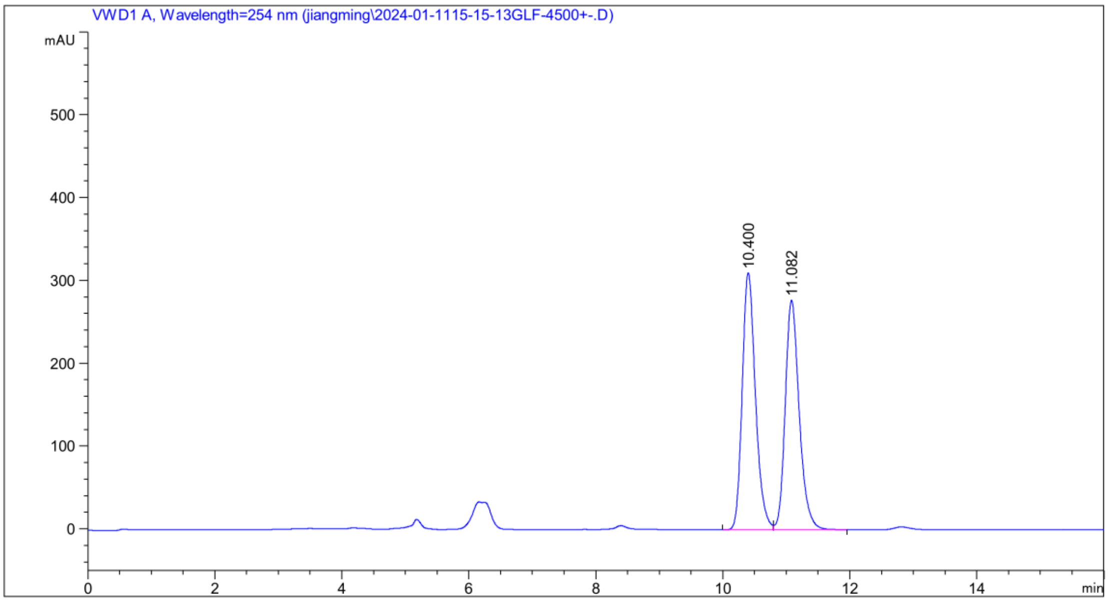

Peak #	RetTime [min]	Type	Width [min]	Area [mAU*s]	Height [mAU]	Area %
1	10.400	BV	0.2222	4498.04492	310.23553	50.4524
2	11.082	VB	0.2337	4244.10791	277.05295	49.5476

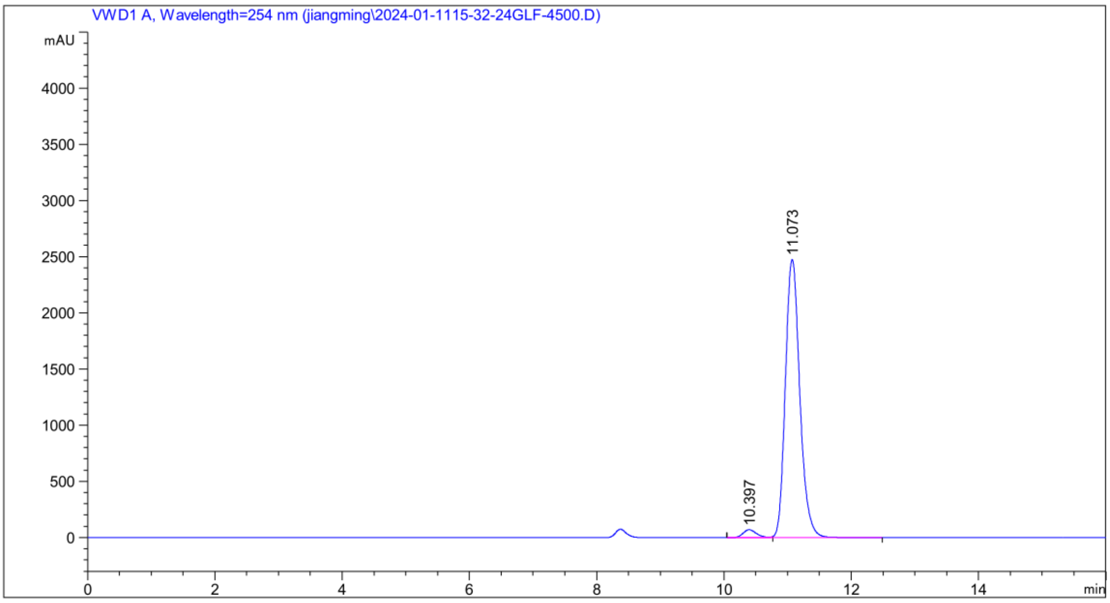

Peak #	RetTime [min]	Type	Width [min]	Area [mAU*s]	Height [mAU]	Area %
1	10.397	BV E	0.2245	1027.01794	70.26724	2.5601
2	11.073	VB R	0.2442	3.90886e4	2475.96973	97.4399

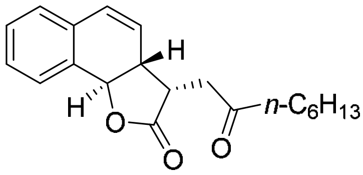

(*S*,*R*,*R*)-**4j**

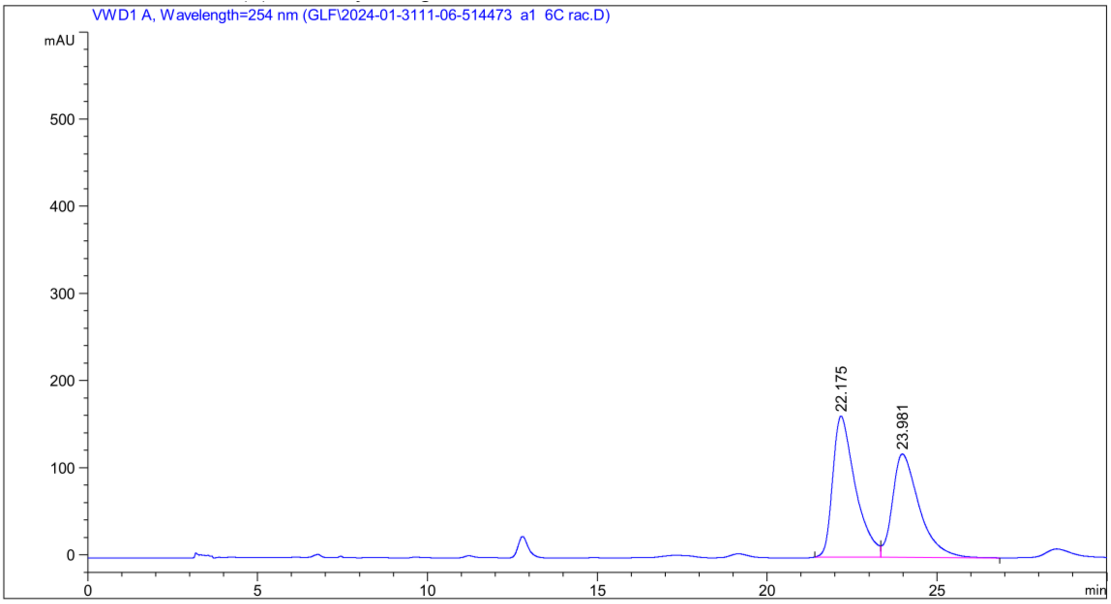

Peak #	RetTime [min]	Type	Width [min]	Area [mAU*s]	Height [mAU]	Area %
1	22.175	HV	0.7035	7517.57373	161.96008	51.6183
2	23.981	VB	0.8108	6502.96045	118.57484	48.3817

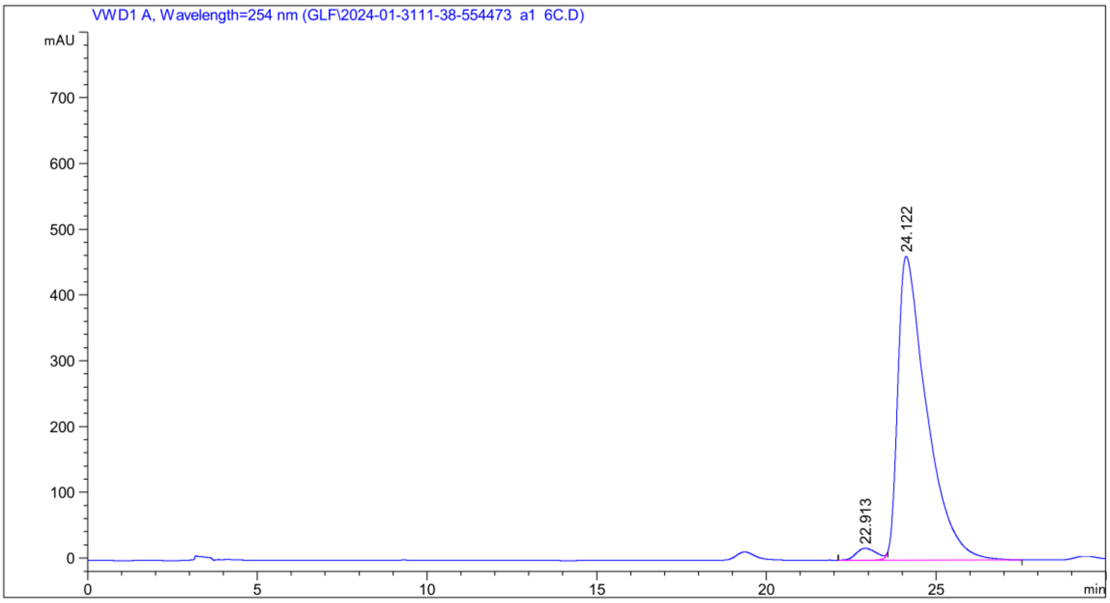

Peak #	RetTime [min]	Type	Width [min]	Area [mAU*s]	Height [mAU]	Area %
1	22.913	BV E	0.6469	740.89233	18.01061	2.6366
2	24.122	VB R	0.8639	2.73593e4	462.01764	97.3634

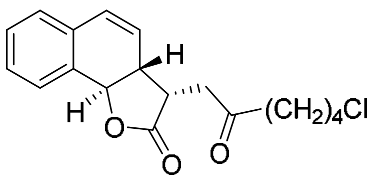

(*S*,*R*,*R*)-**4k**

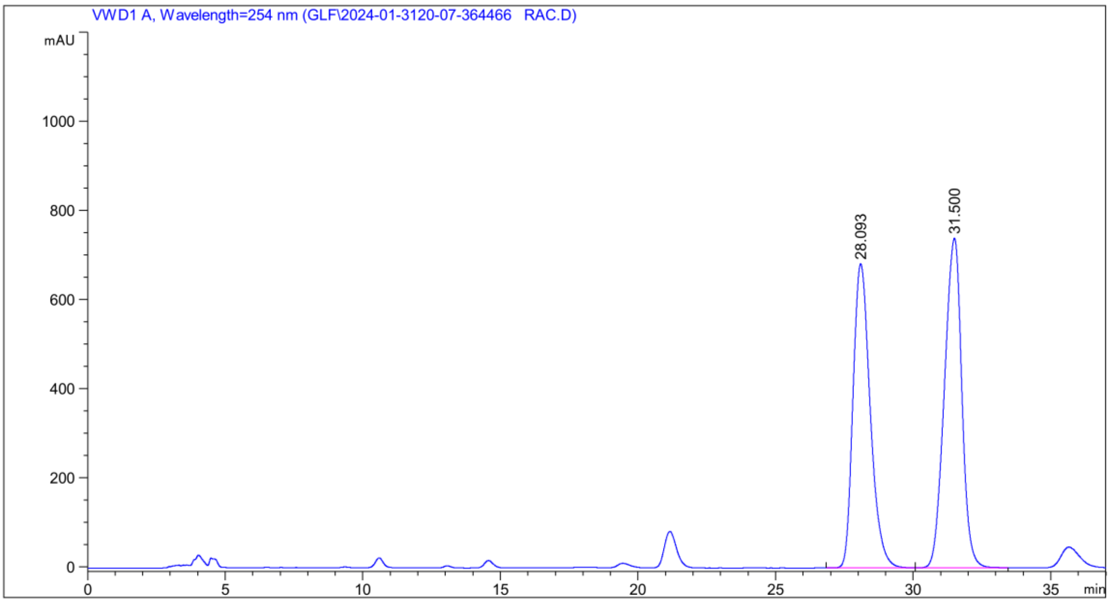

Peak #	RetTime [min]	Type	Width [min]	Area [mAU*s]	Height [mAU]	Area %
1	28.093	BB	0.6757	2.95557e4	681.89978	48.6748
2	31.500	BB	0.6661	3.24386e4	739.02893	51.3252

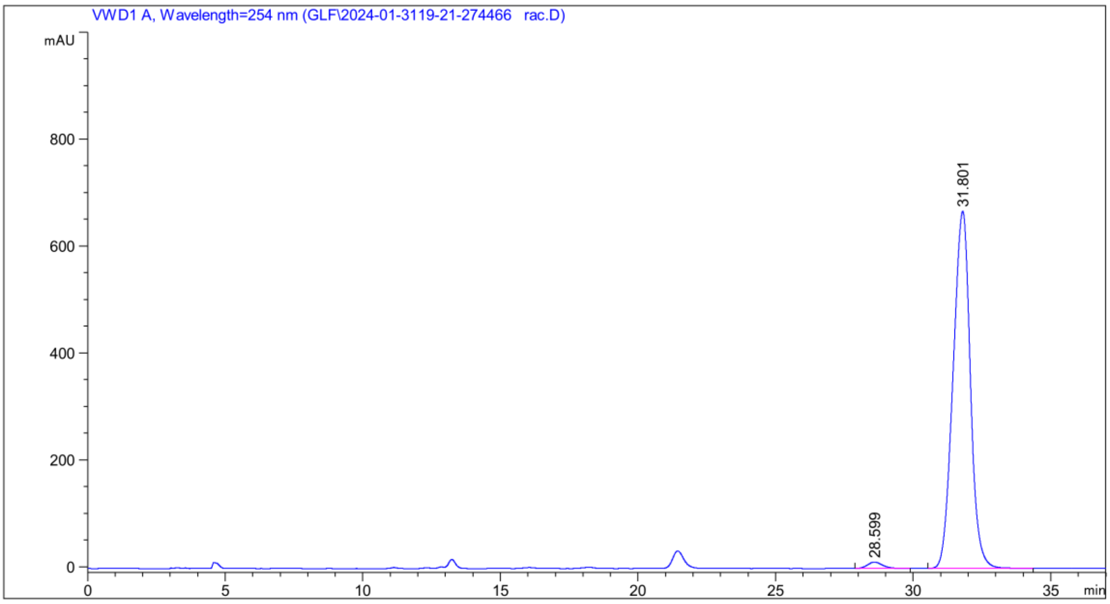

Peak #	RetTime [min]	Type	Width [min]	Area [mAU*s]	Height [mAU]	Area %
1	28.599	HH	0.5697	449.32431	11.92080	1.5201
2	31.801	HH	0.6767	2.91101e4	667.57953	98.4799

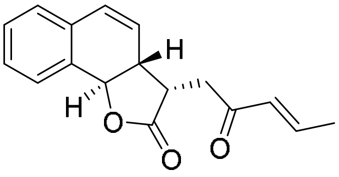

(*S*,*R*,*R*))-**4l**

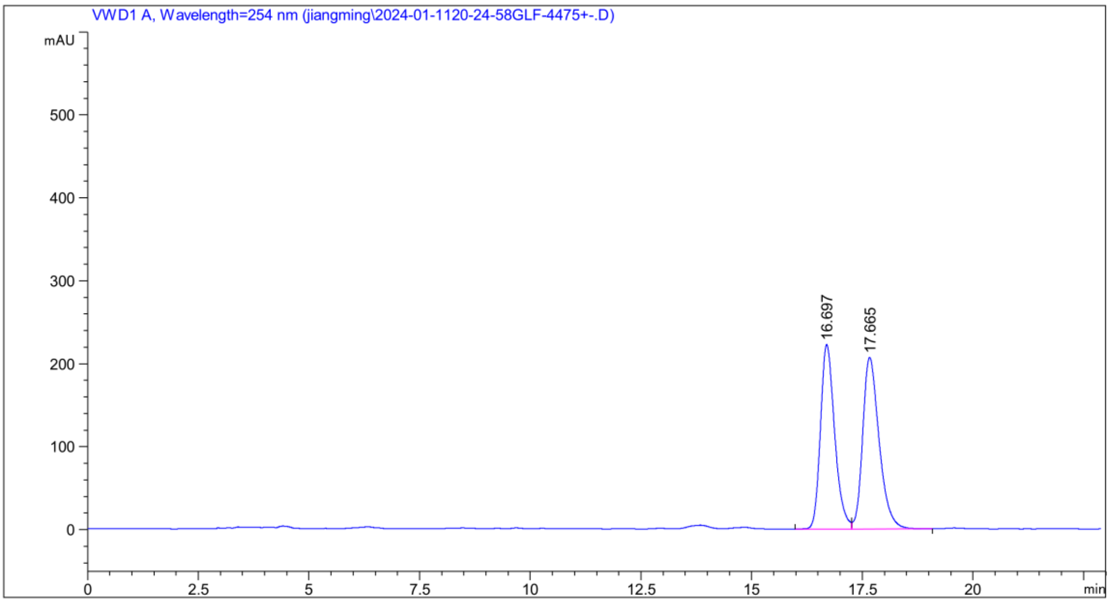

Peak #	RetTime [min]	Type	Width [min]	Area [mAU*s]	Height [mAU]	Area %
1	16.697	BV	0.3404	4964.72119	222.39754	48.1780
2	17.665	VB	0.3943	5340.22314	206.75359	51.8220

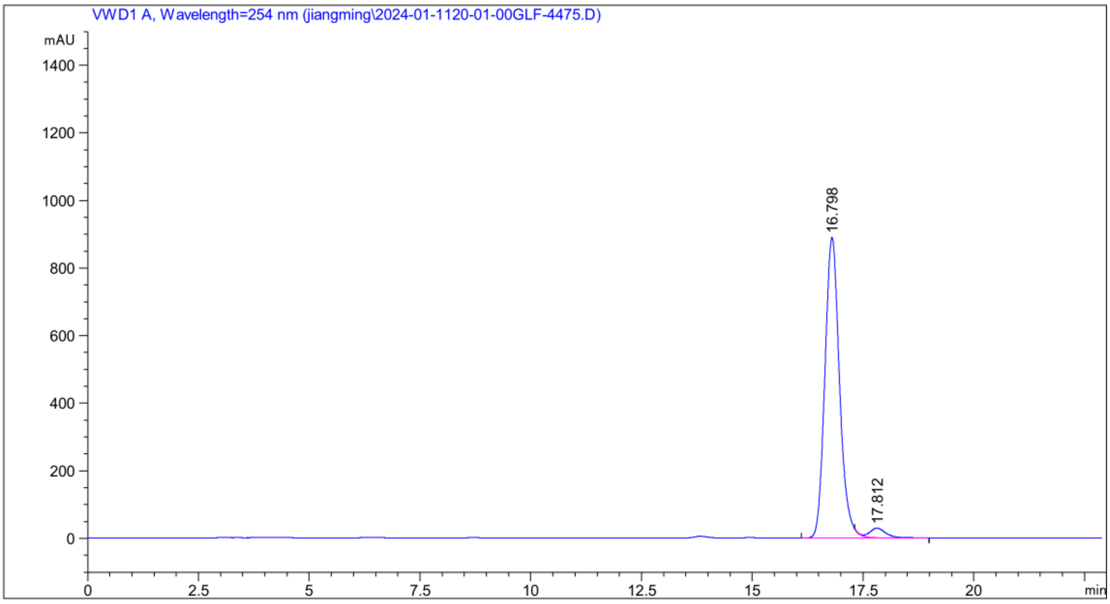

Peak #	RetTime [min]	Type	Width [min]	Area [mAU*s]	Height [mAU]	Area %
1	16.798	BV R	0.3535	2.04728e4	889.54742	96.6243
2	17.812	VB E	0.3821	715.24329	28.18115	3.3757

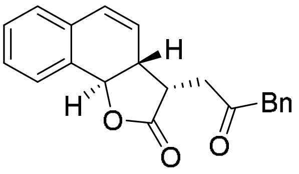

(*S*,*R*,*R*)-**4m**

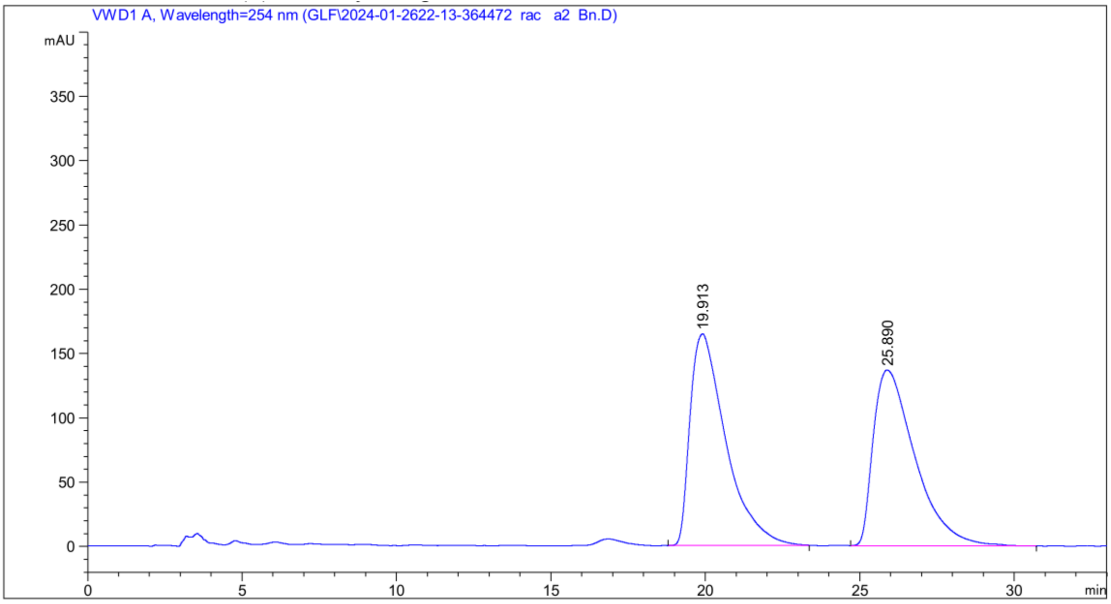

Peak #	RetTime [min]	Type	Width [min]	Area [mAU*s]	Height [mAU]	Area %
1	19.913	BBA	1.2200	1.32307e4	164.55432	50.7431
2	25.890	BB	1.4113	1.28432e4	136.67883	49.2569

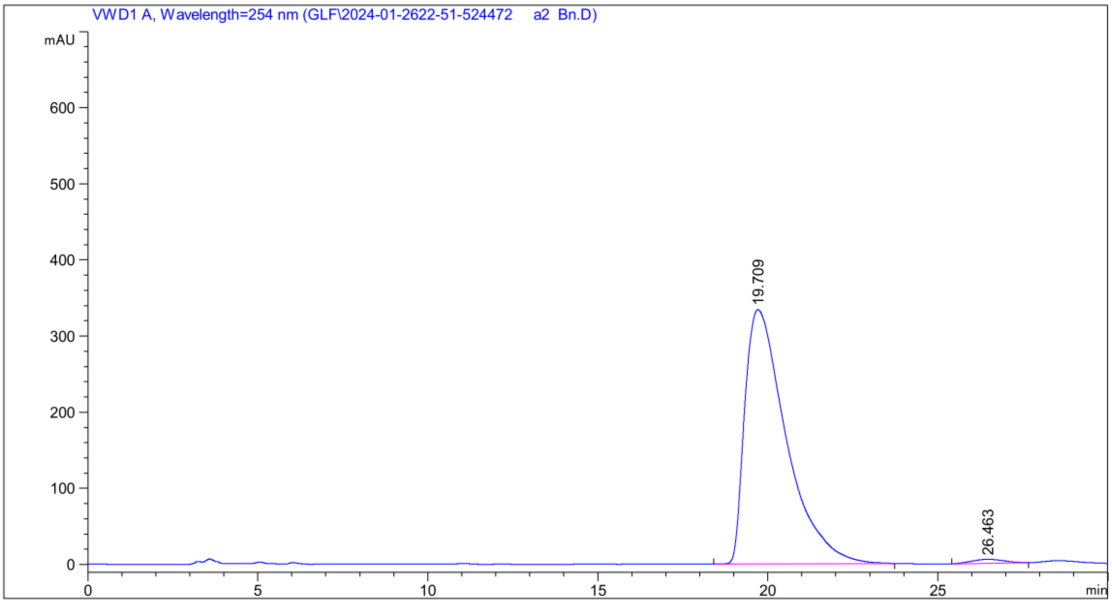

Peak #	RetTime [min]	Type	Width [min]	Area [mAU*s]	Height [mAU]	Area %
1	19.709	BBA	1.2704	2.81398e4	333.85138	98.7616
2	26.463	BB	0.9230	352.86670	5.33936	1.2384

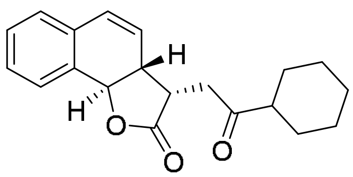

(*S*,*R*,*R*)-**4n**

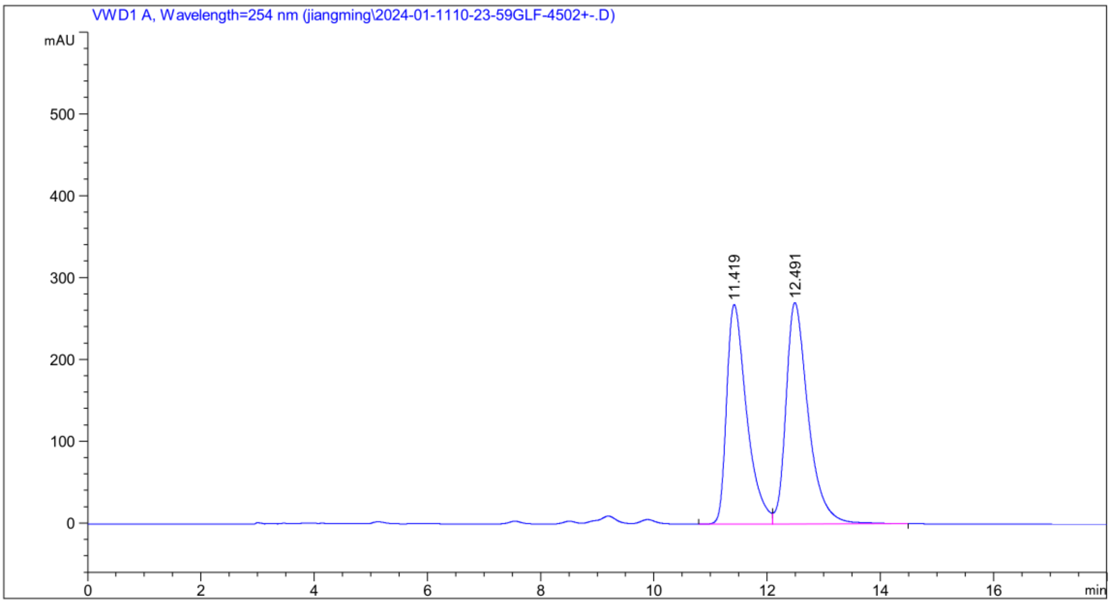

Peak #	RetTime [min]	Type	Width [min]	Area [mAU*s]	Height [mAU]	Area %
1	11.419	BV	0.3718	6607.56152	267.88489	49.1479
2	12.491	VB	0.4137	7406.97314	270.27524	50.8521

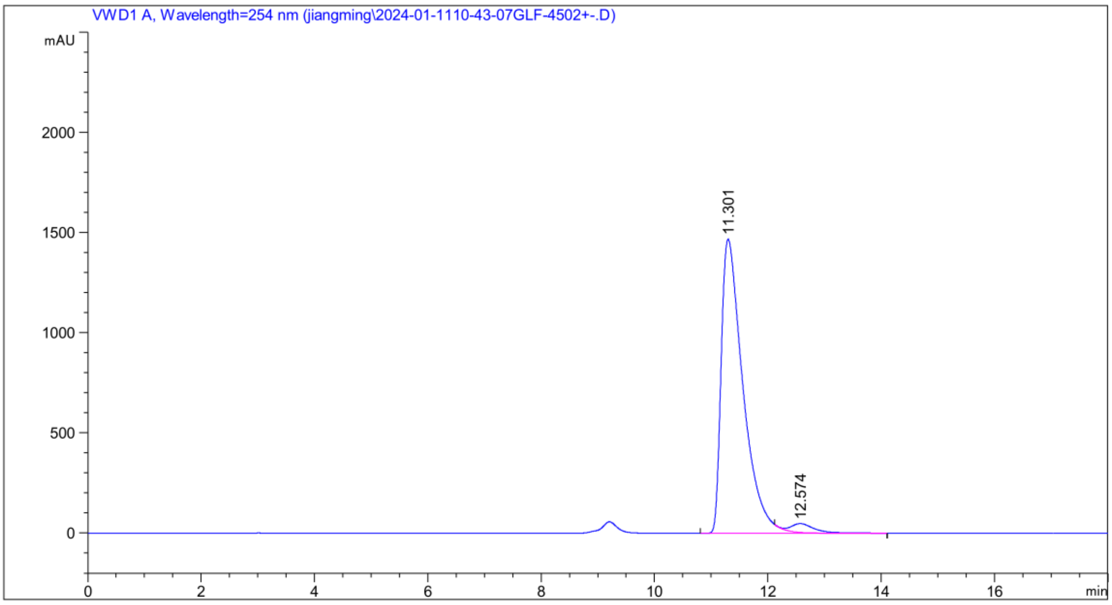

Peak #	RetTime [min]	Type	Width [min]	Area [mAU*s]	Height [mAU]	Area %
1	11.301	BV R	0.3985	3.91821e4	1467.52588	96.8422
2	12.574	VB E	0.4324	1277.62854	43.79144	3.1578

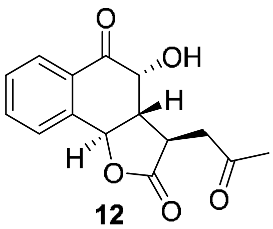

12

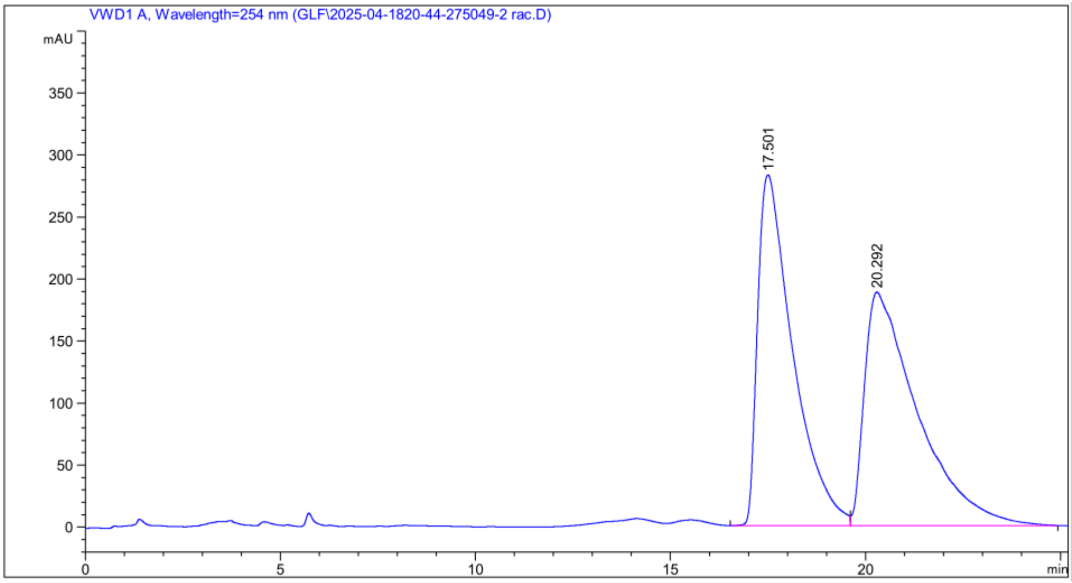

Peak #	RetTime [min]	Type	Width [min]	Area [mAU*s]	Height [mAU]	Area %
1	17.501	BV	0.8816	1.77135e4	282.84723	49.9258
2	20.292	VBA	1.2470	1.77661e4	188.26498	50.0742

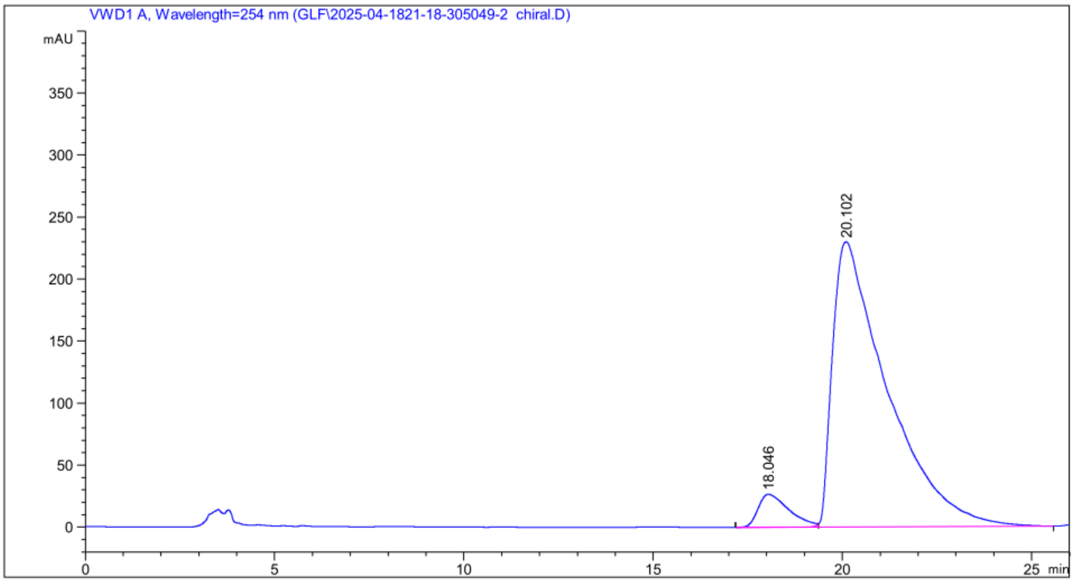

Peak #	RetTime [min]	Type	Width [min]	Area [mAU*s]	Height [mAU]	Area %
1	18.046	BV E	0.8402	1487.53125	26.52913	6.0555
2	20.102	VB R	1.3847	2.30774e4	229.77969	93.9445

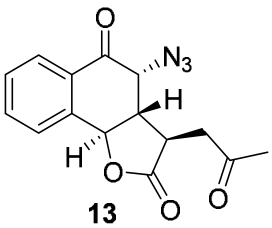

13

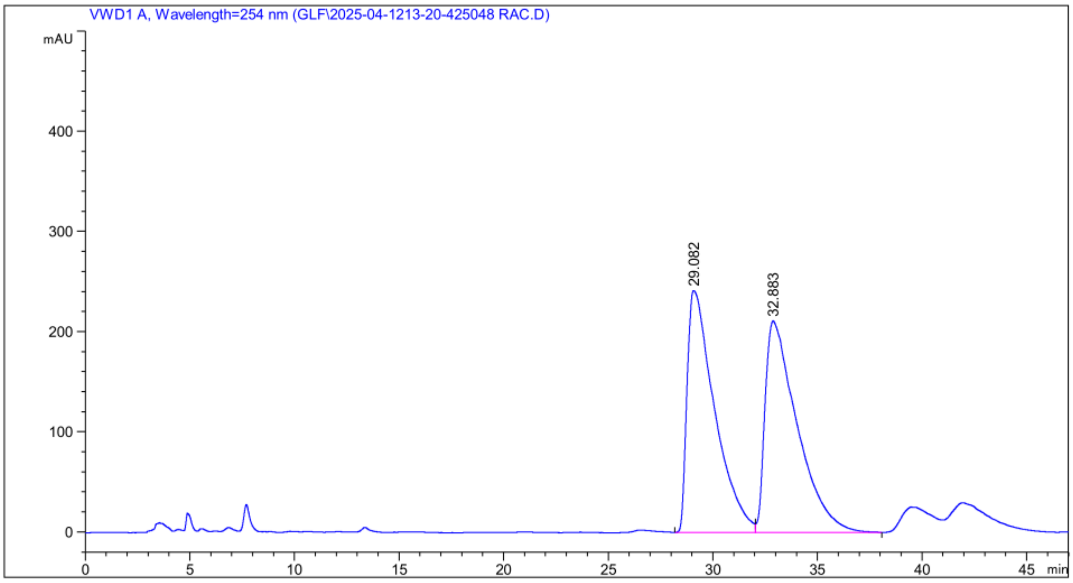

Peak #	RetTime [min]	Type	Width [min]	Area [mAU*s]	Height [mAU]	Area %
1	29.082	BV	1.2755	2.20437e4	241.37202	49.4967
2	32.883	VB	1.4466	2.24921e4	211.00339	50.5033

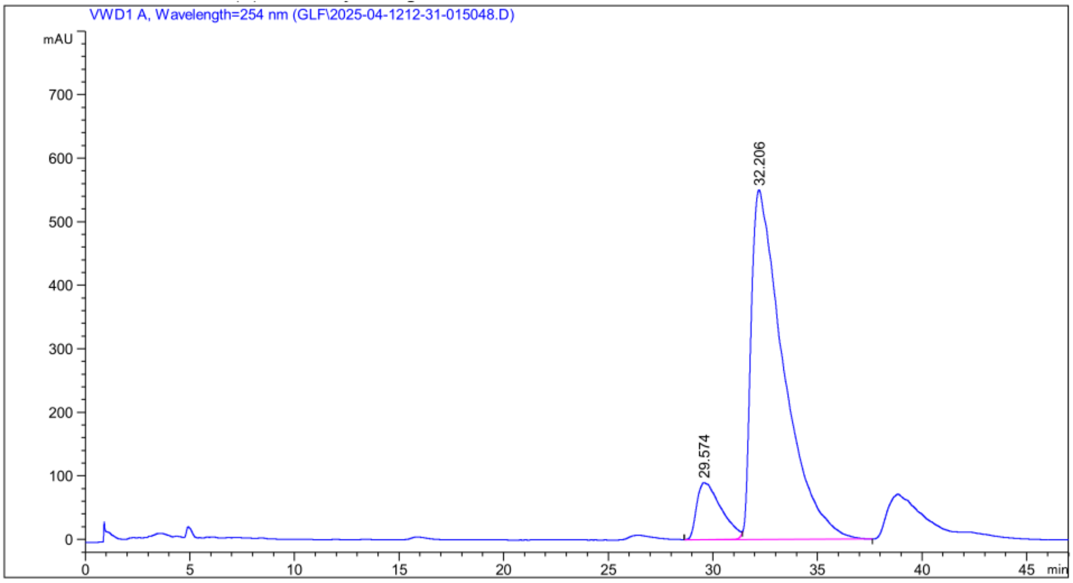

Peak #	RetTime [min]	Type	Width [min]	Area [mAU*s]	Height [mAU]	Area %
1	29.574	BV E	1.0838	7256.35742	89.70256	10.9142
2	32.206	VB R	1.4300	5.92291e4	550.15436	89.0858

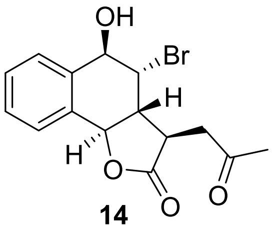

14

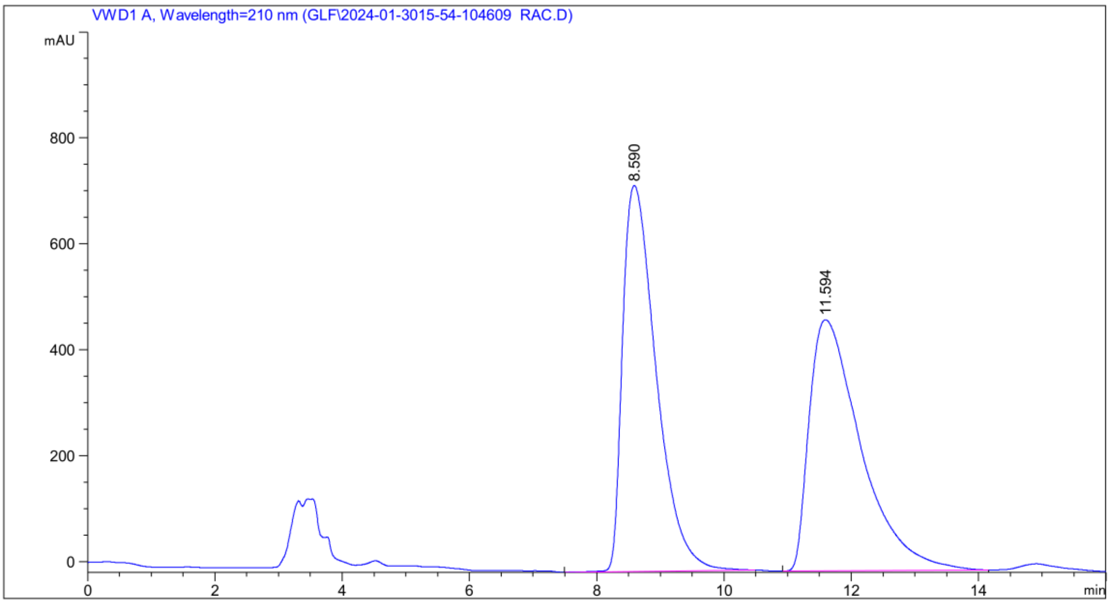

Peak #	RetTime [min]	Type	Width [min]	Area [mAU*s]	Height [mAU]	Area %
1	8.590	BB	0.5682	2.67442e4	728.67029	50.0465
2	11.594	BB	0.8367	2.66946e4	474.24036	49.9535

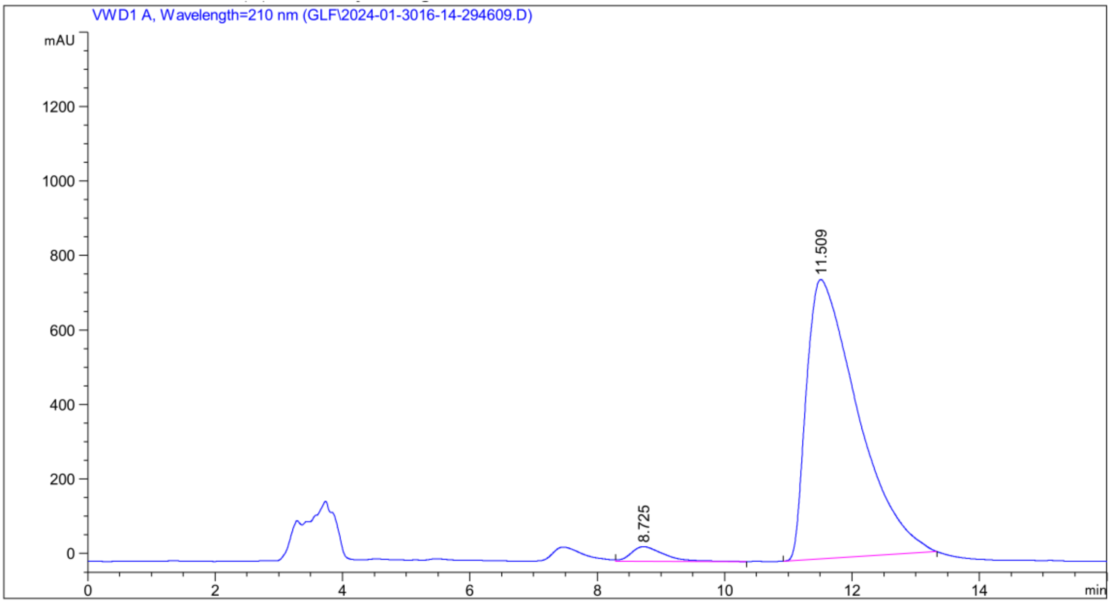

Peak #	RetTime [min]	Type	Width [min]	Area [mAU*s]	Height [mAU]	Area %
1	8.725	VB	0.5324	1422.11011	39.46996	3.2827
2	11.509	BBA	0.8399	4.18990e4	749.84259	96.7173

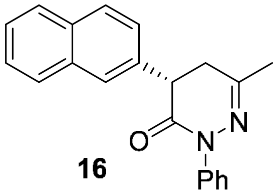

16

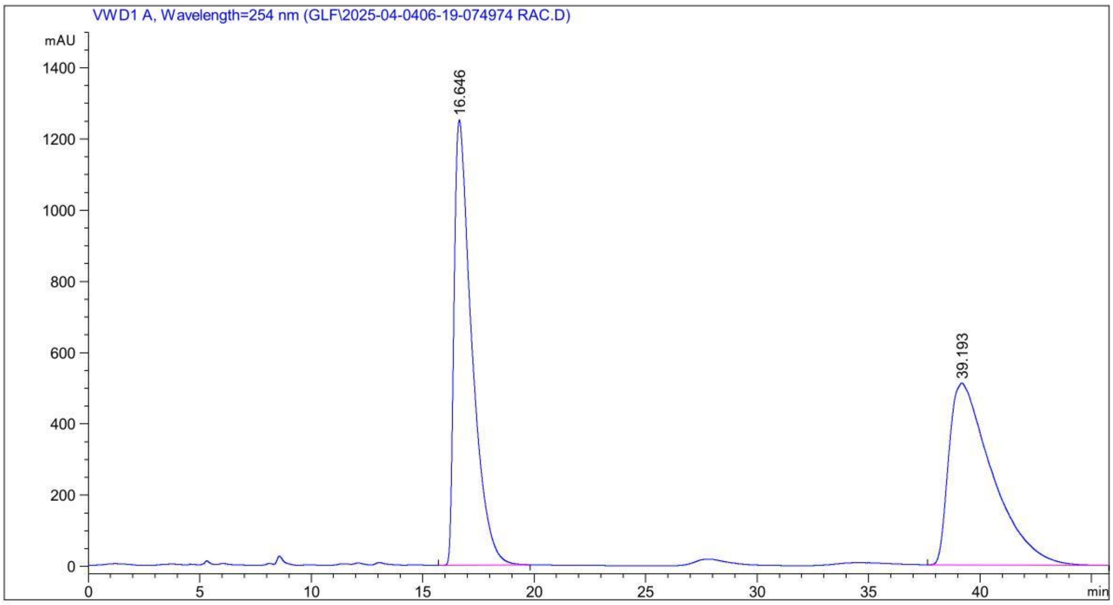

Peak #	RetTime [min]	Type	Width [min]	Area [mAU*s]	Height [mAU]	Area %
1	16.646	BB	0.8184	6.91554e4	1250.11572	50.0333
2	39.193	BBA	1.8290	6.90634e4	510.62399	49.9667

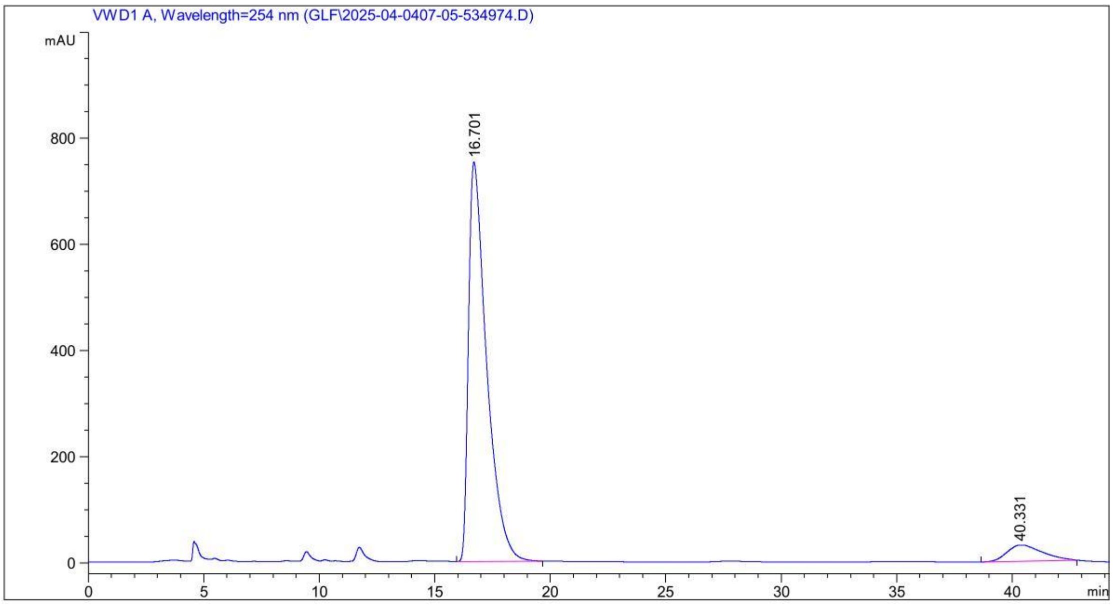

Peak #	RetTime [min]	Type	Width [min]	Area [mAU*s]	Height [mAU]	Area %
1	16.701	BB	0.8060	4.09719e4	752.73364	92.7546
2	40.331	BBA	1.3952	3200.48315	30.35250	7.2454

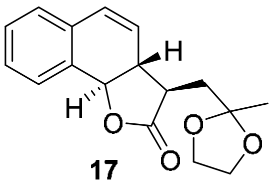

17

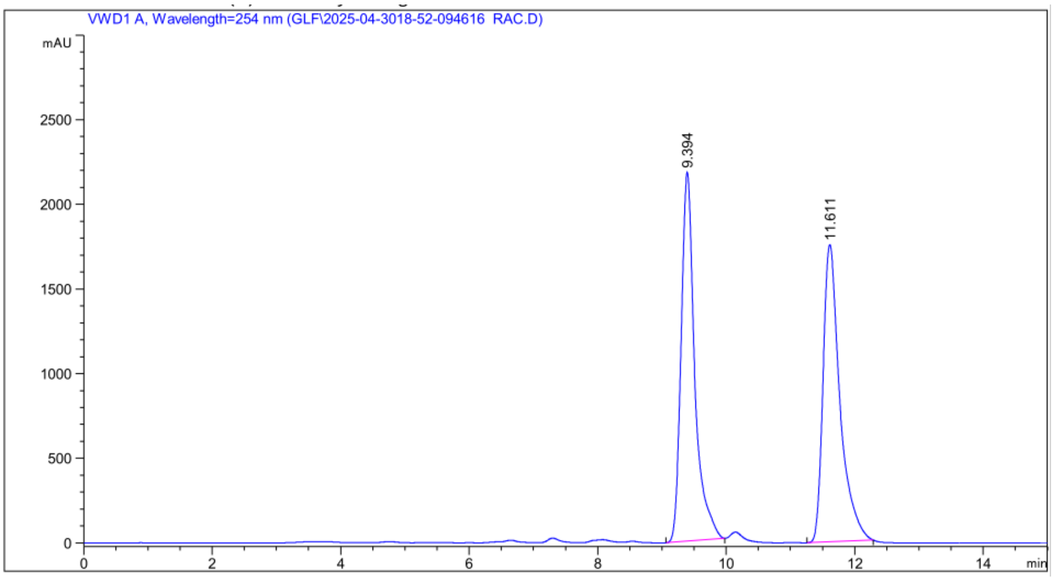

Peak #	RetTime [min]	Type	Width [min]	Area [mAU*s]	Height [mAU]	Area %
1	9.394	BB	0.2202	3.21605e4	2178.97290	50.1238
2	11.611	BBA	0.2726	3.20017e4	1755.99670	49.8762

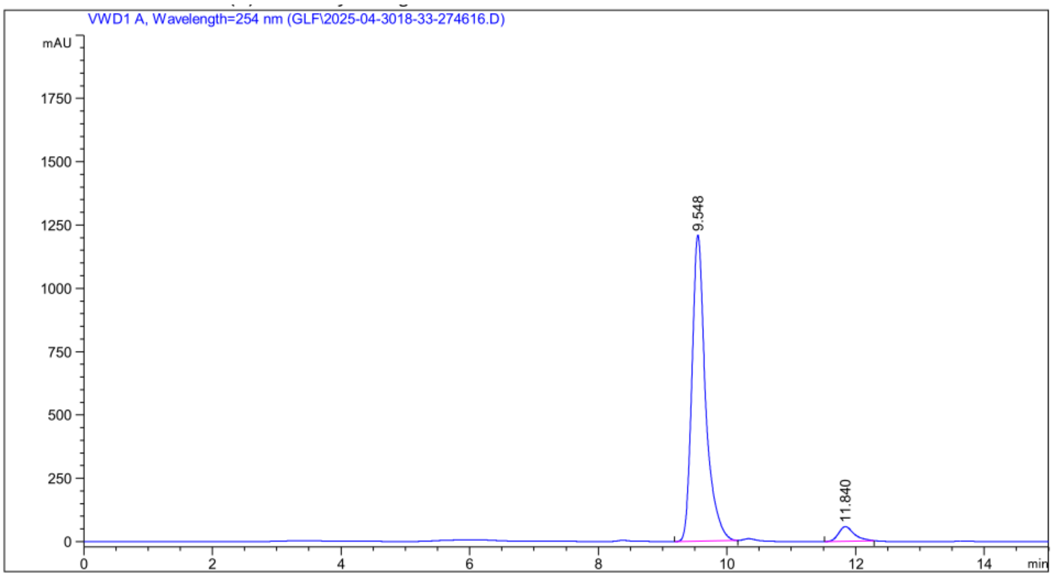

Peak #	RetTime [min]	Type	Width [min]	Area [mAU*s]	Height [mAU]	Area %
1	9.548	BB	0.2202	1.78459e4	1208.82666	94.8876
2	11.840	BBA	0.2522	961.49896	57.46272	5.1124

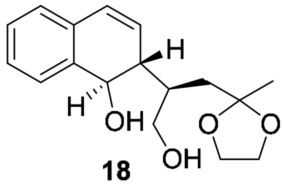

18

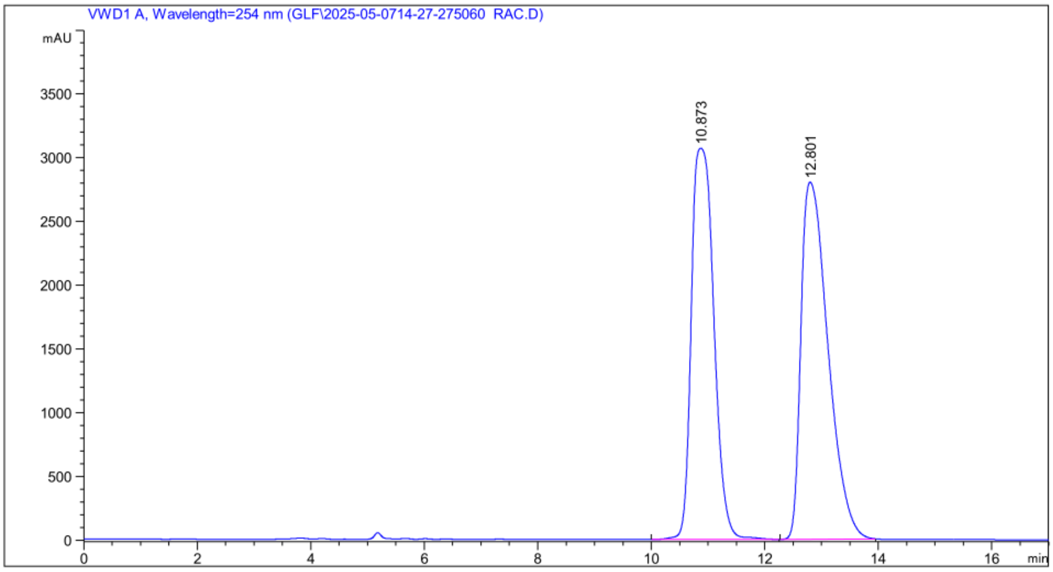

Peak #	RetTime [min]	Type	Width [min]	Area [mAU*s]	Height [mAU]	Area %
1	10.873	BB	0.4341	8.47123e4	3068.78149	47.7950
2	12.801	BBA	0.5055	9.25287e4	2800.05688	52.2050

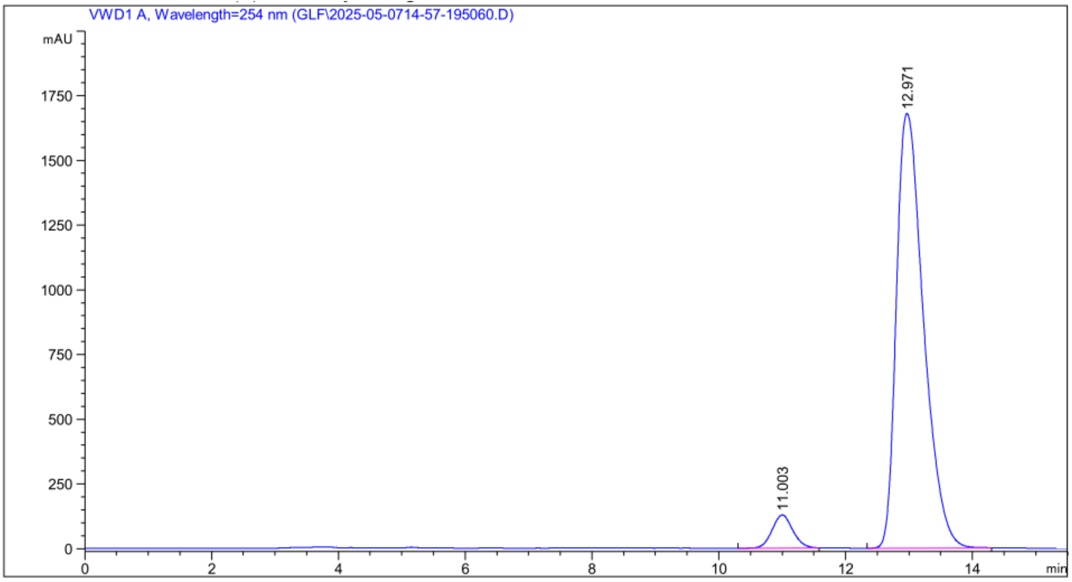

Peak #	RetTime [min]	Type	Width [min]	Area [mAU*s]	Height [mAU]	Area %
1	11.003	BBA	0.3481	2890.78613	127.71815	5.4748
2	12.971	BBA	0.4508	4.99113e4	1678.91138	94.5252

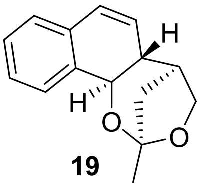

19

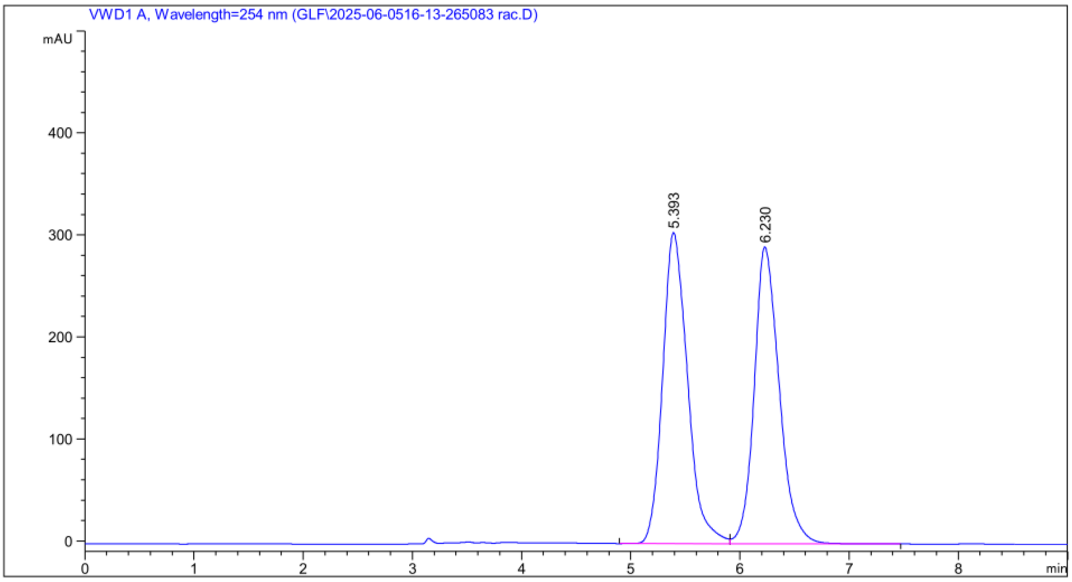

Peak #	RetTime [min]	Type	Width [min]	Area [mAU*s]	Height [mAU]	Area %
1	5.393	BV	0.2511	4838.28320	304.94125	51.0174
2	6.230	VB	0.2473	4645.30566	290.83701	48.9826

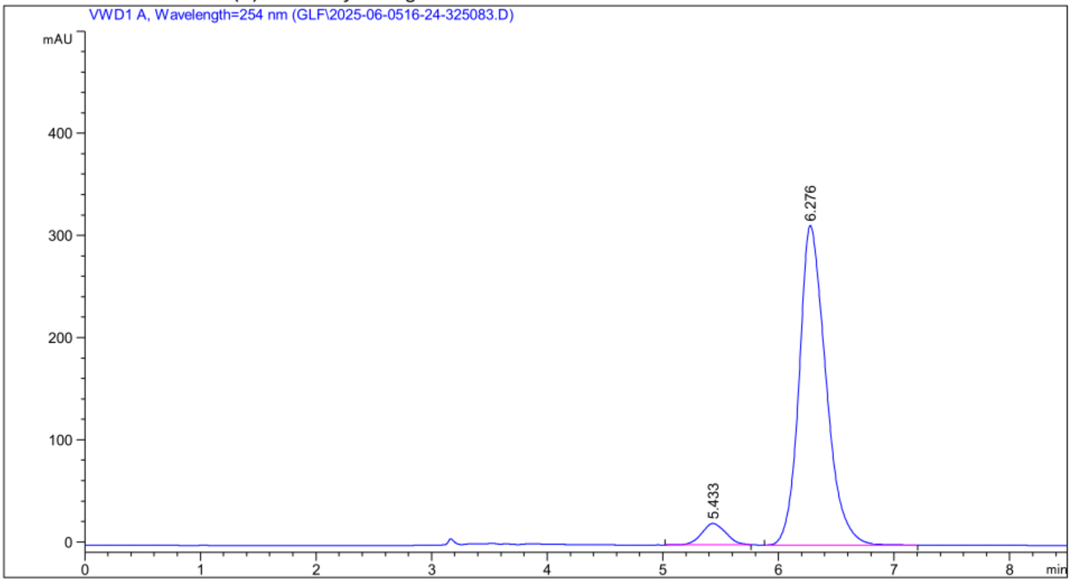

Peak #	RetTime [min]	Type	Width [min]	Area [mAU*s]	Height [mAU]	Area %
1	5.433	BBA	0.2300	301.59552	20.94279	5.6539
2	6.276	BBA	0.2477	5032.65479	312.84402	94.3461

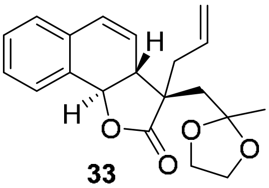

33

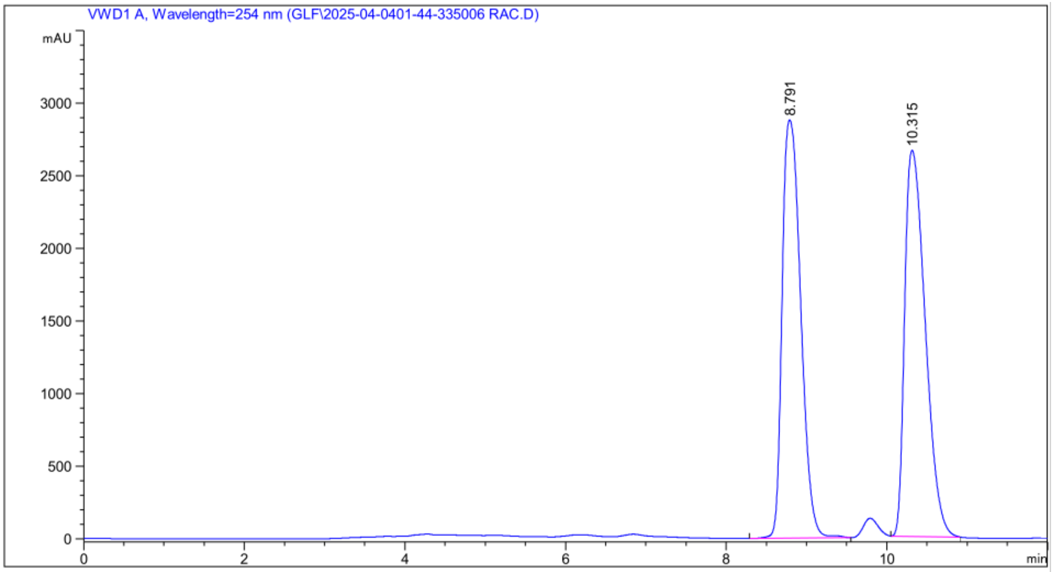

Peak #	RetTime [min]	Type	Width [min]	Area [mAU*s]	Height [mAU]	Area %
1	8.791	BV R	0.2537	4.62110e4	2879.44165	49.4953
2	10.315	BBA	0.2810	4.71535e4	2657.16211	50.5047

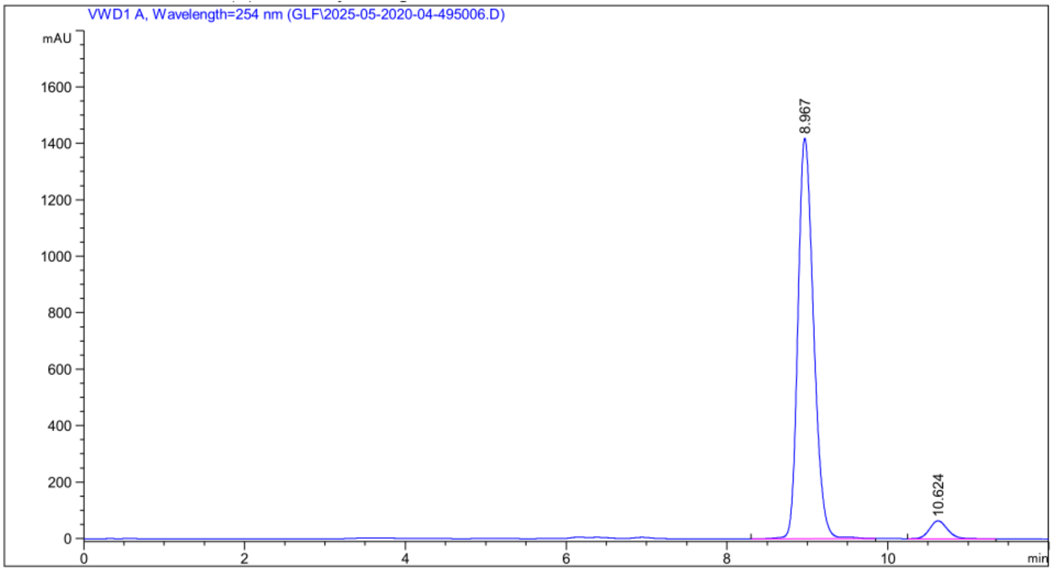

Peak #	RetTime [min]	Type	Width [min]	Area [mAU*s]	Height [mAU]	Area %
1	8.967	BV R	0.2090	1.91633e4	1419.29504	95.1464
2	10.624	BB	0.2388	977.55499	63.43624	4.8536

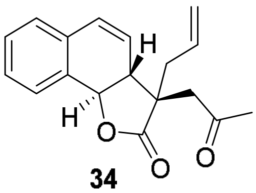

34

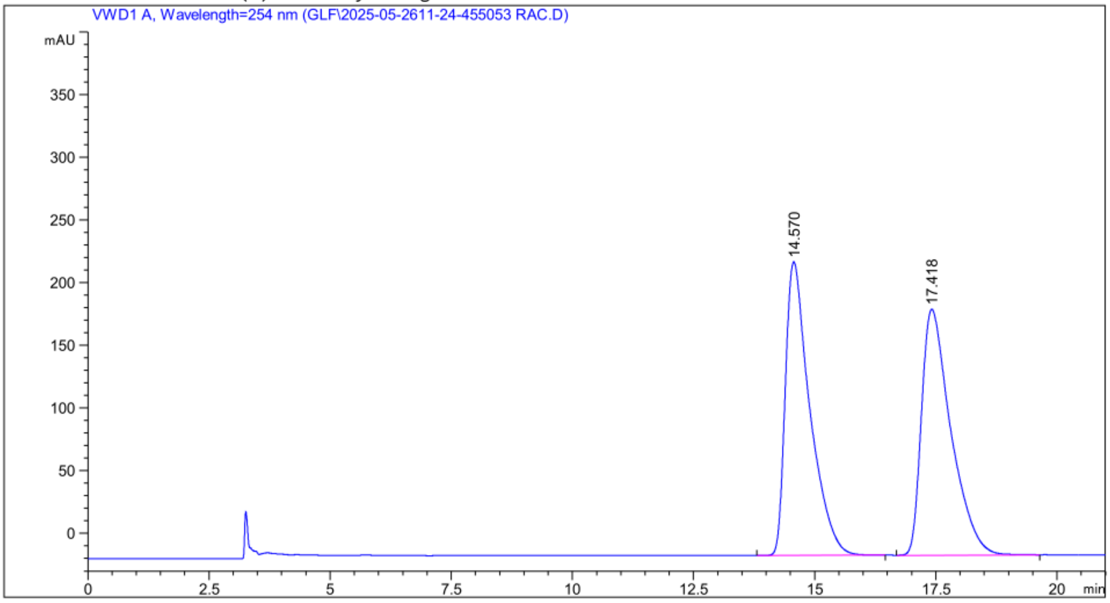

Peak #	RetTime [min]	Type	Width [min]	Area [mAU*s]	Height [mAU]	Area %
1	14.570	BB	0.5147	8242.87207	234.14523	49.9792
2	17.418	BB	0.6338	8249.74707	196.10515	50.0208

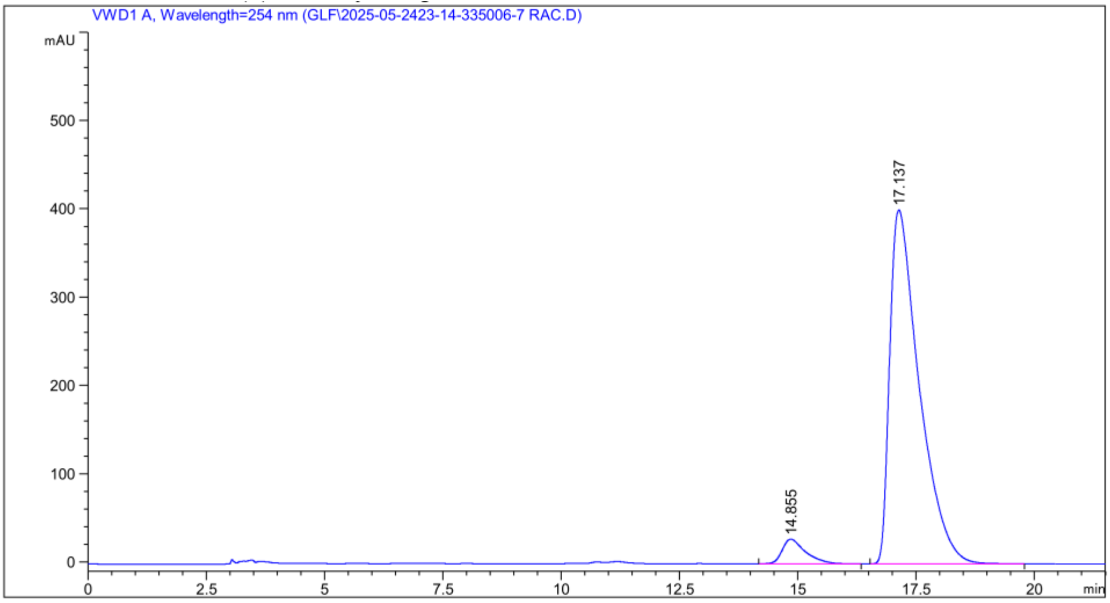

Peak #	RetTime [min]	Type	Width [min]	Area [mAU*s]	Height [mAU]	Area %
1	14.855	BB	0.5009	949.84540	27.85178	5.1881
2	17.137	BB	0.6465	1.73583e4	400.62842	94.8119

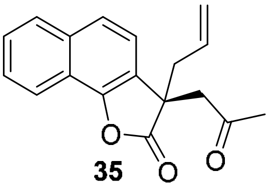

35

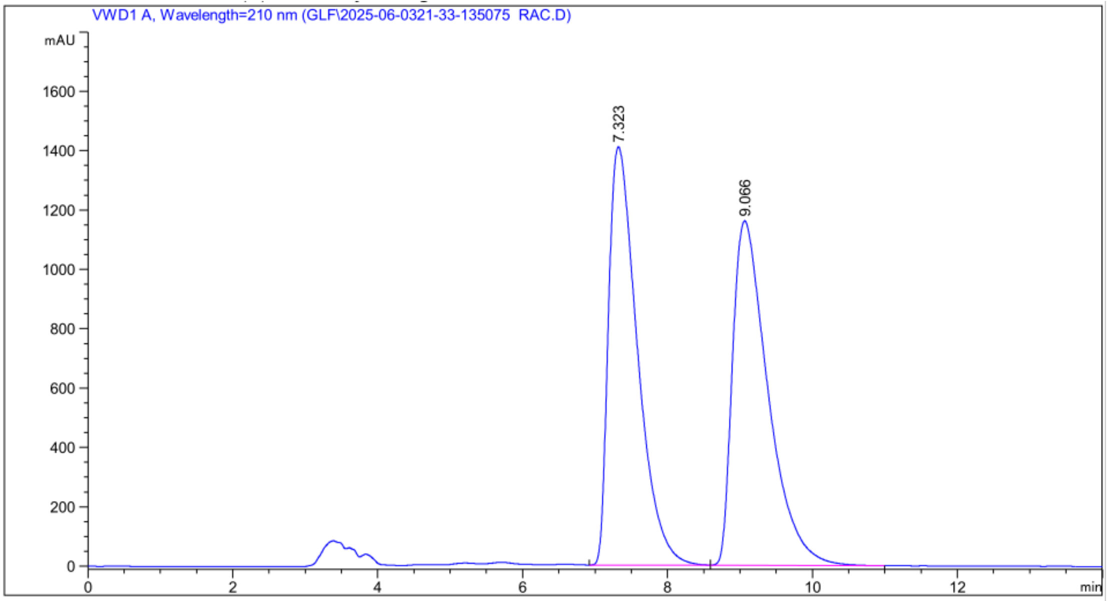

Peak #	RetTime [min]	Type	Width [min]	Area [mAU*s]	Height [mAU]	Area %
1	7.323	BB	0.4308	3.90107e4	1409.78345	49.7606
2	9.066	BB	0.5192	3.93860e4	1159.69934	50.2394

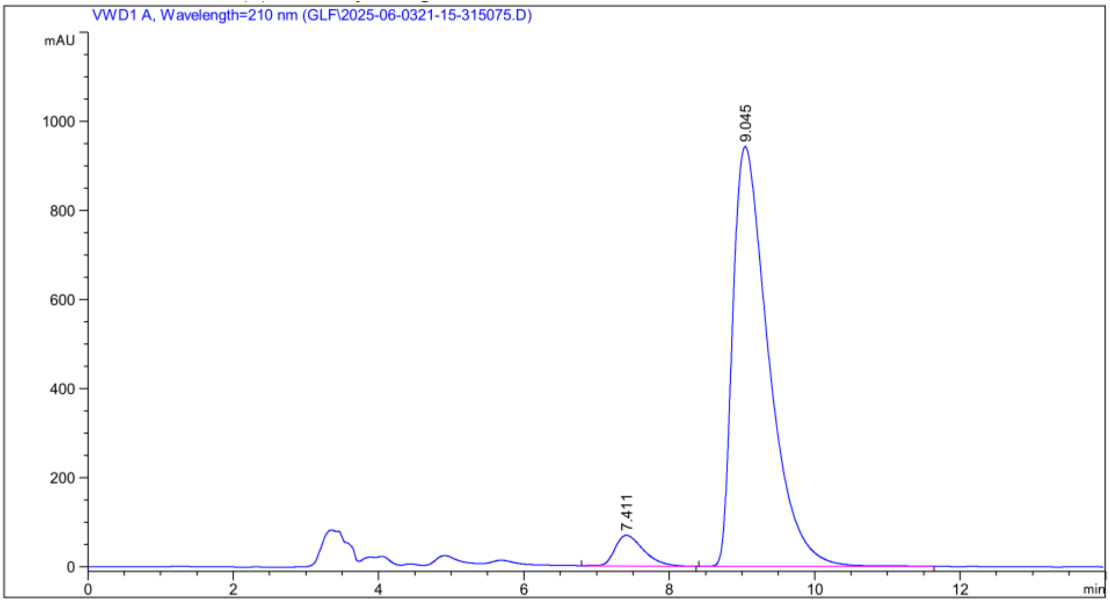

Peak #	RetTime [min]	Type	Width [min]	Area [mAU*s]	Height [mAU]	Area %
1	7.411	BB	0.4232	1879.21191	68.90212	5.5174
2	9.045	BV R	0.5225	3.21803e4	942.08911	94.4826

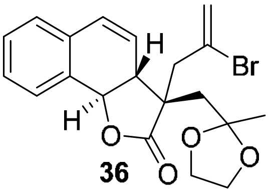

36

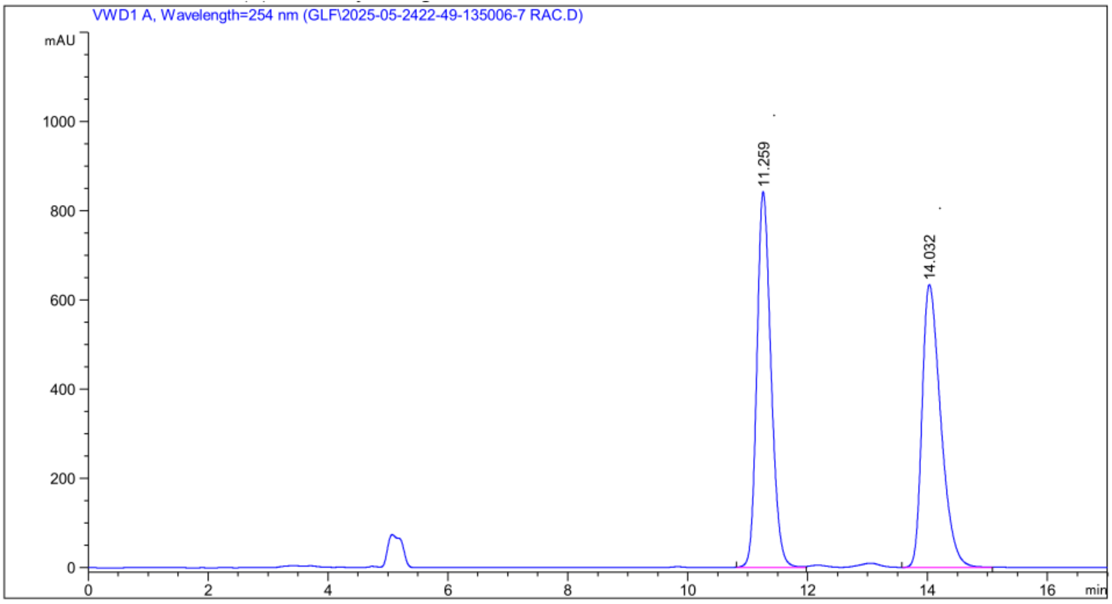

Peak #	RetTime [min]	Type	Width [min]	Area [mAU*s]	Height [mAU]	Area %
1	11.259	FM	0.2739	1.38461e4	842.59430	50.0651
2	14.032	MF	0.3626	1.38101e4	634.85333	49.9349

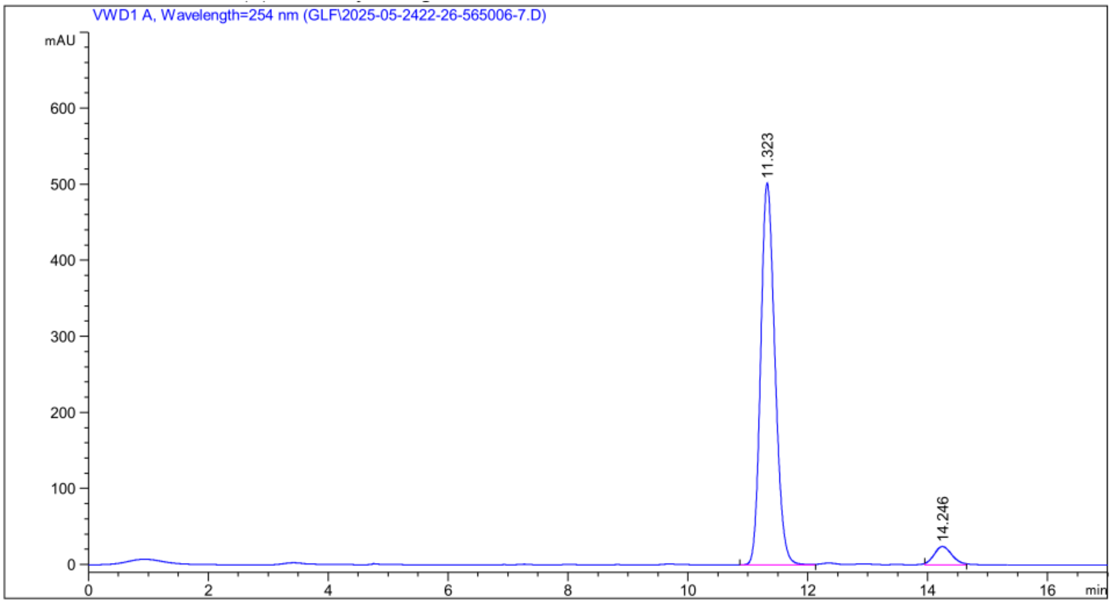

Peak #	RetTime [min]	Type	Width [min]	Area [mAU*s]	Height [mAU]	Area %
1	11.323	MF	0.2753	8292.87793	501.99460	94.3416
2	14.246	MF	0.3436	497.38974	24.12485	5.6584

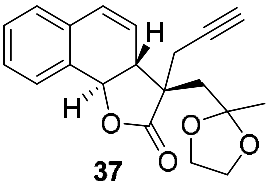

37

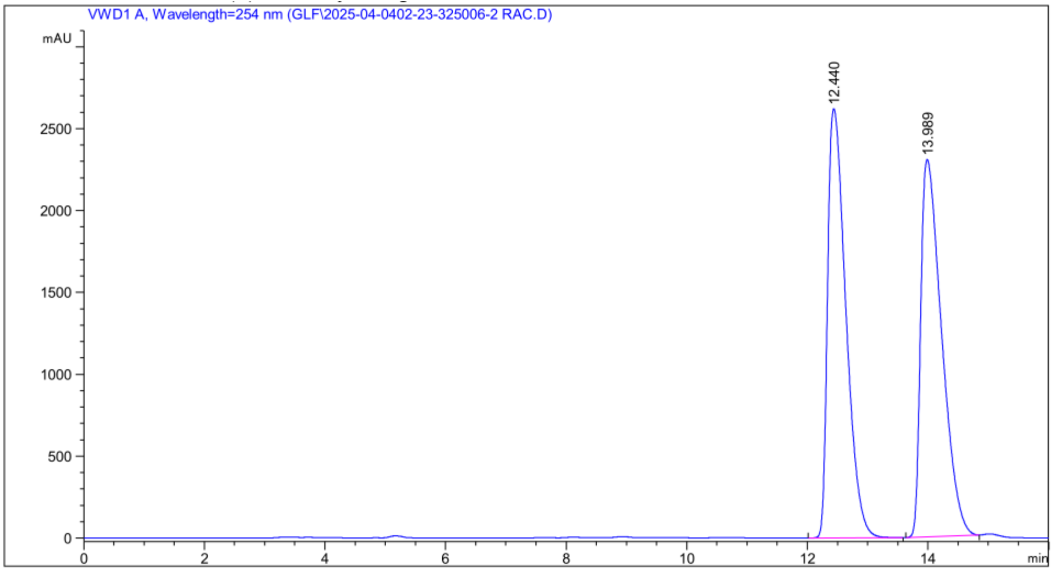

Peak #	RetTime [min]	Type	Width [min]	Area [mAU*s]	Height [mAU]	Area %
1	12.440	BB	0.3219	5.50124e4	2620.56445	49.7799
2	13.989	BB	0.3639	5.54990e4	2305.37158	50.2201

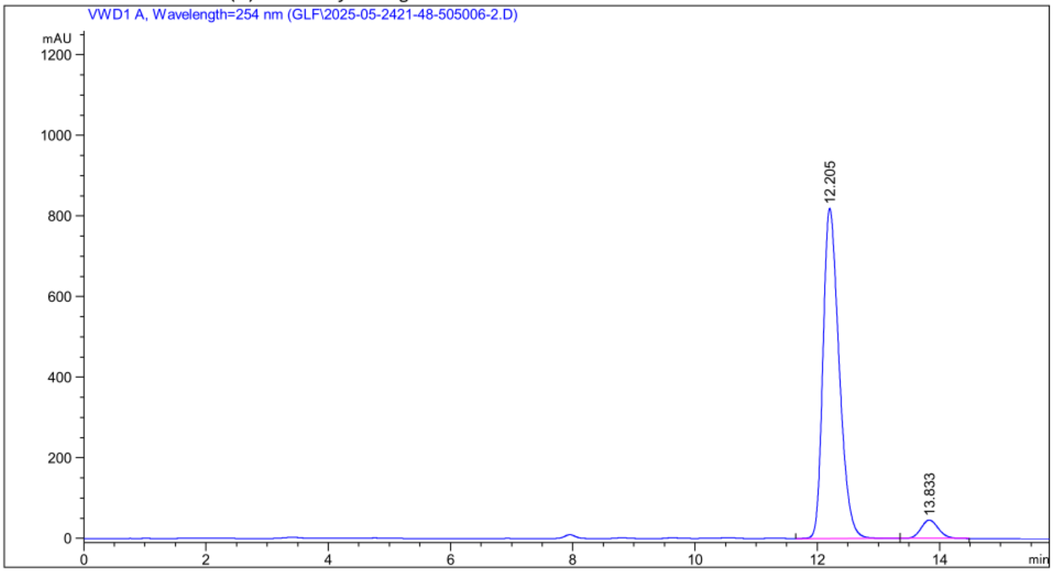

Peak #	RetTime [min]	Type	Width [min]	Area [mAU*s]	Height [mAU]	Area %
1	12.205	BB	0.2816	1.50059e4	819.75598	94.3417
2	13.833	BB	0.3077	900.00122	45.51839	5.6583

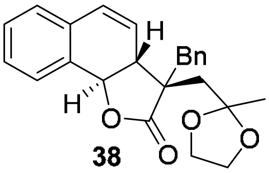

38

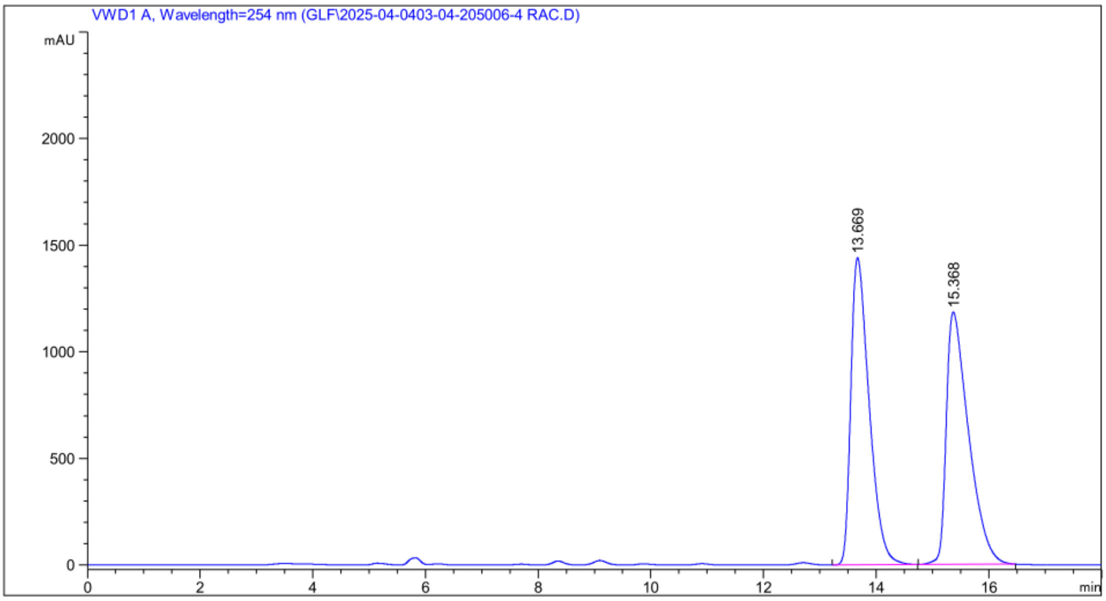

Peak #	RetTime [min]	Type	Width [min]	Area [mAU*s]	Height [mAU]	Area %
1	13.669	BB	0.3432	3.24857e4	1440.04614	49.9344
2	15.368	BBA	0.4081	3.25711e4	1183.53186	50.0656

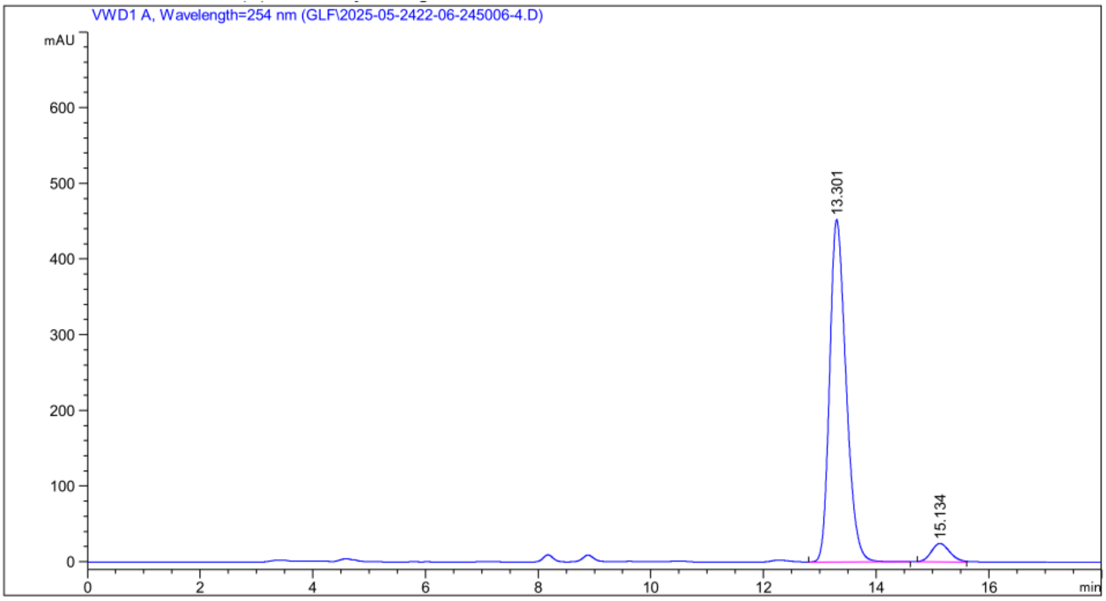

Peak #	RetTime [min]	Type	Width [min]	Area [mAU*s]	Height [mAU]	Area %
1	13.301	VB	0.3120	9148.14746	452.32254	94.2586
2	15.134	FM	0.3801	557.21991	24.43425	5.7414

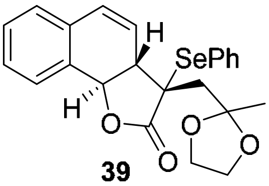

39

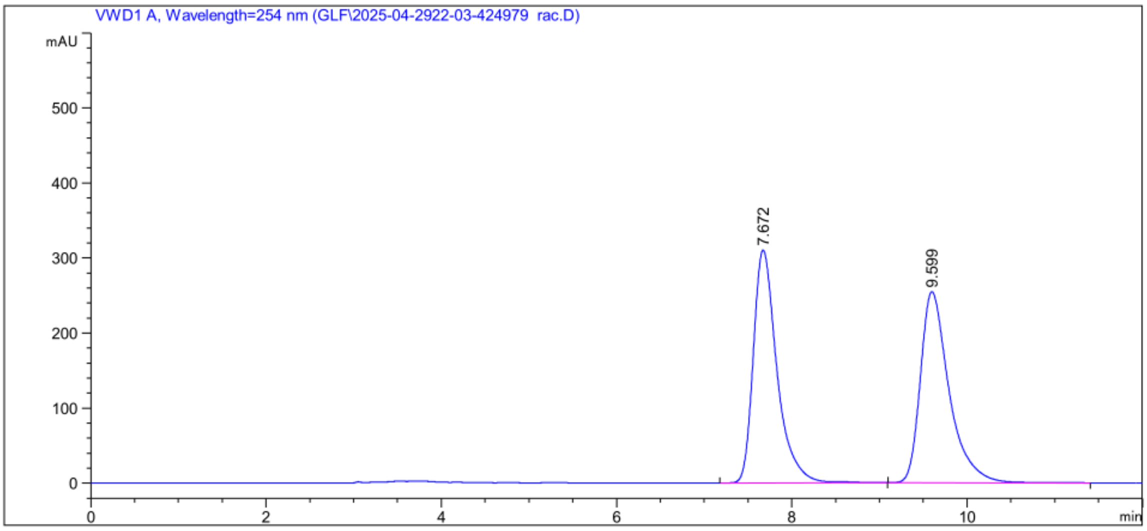

Peak #	RetTime [min]	Type	Width [min]	Area [mAU*s]	Height [mAU]	Area %
1	7.672	BV R	0.2850	5823.56396	310.17255	50.1421
2	9.599	BB	0.3423	5790.55029	254.61105	49.8579

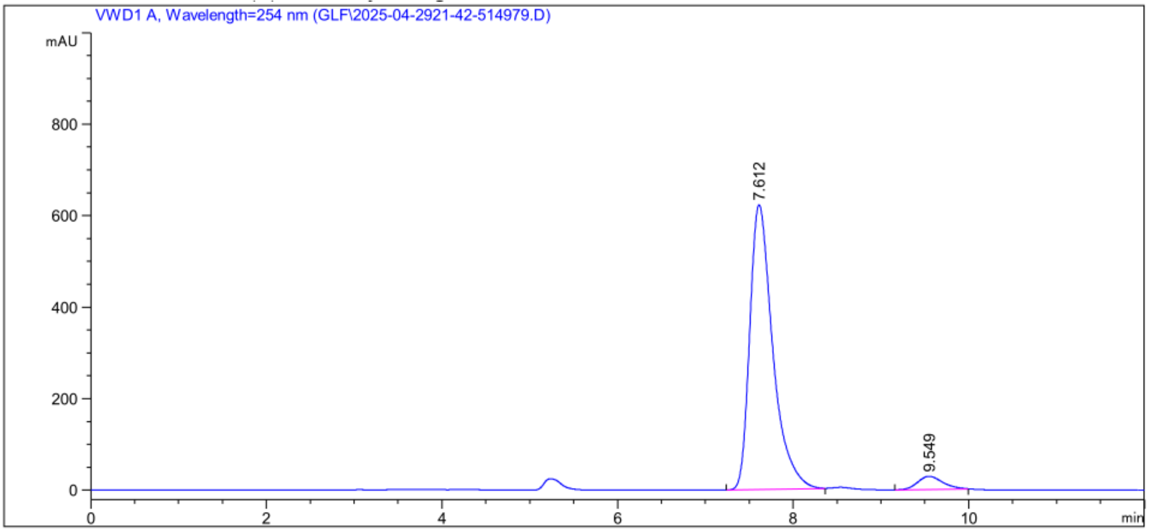

Peak #	RetTime [min]	Type	Width [min]	Area [mAU*s]	Height [mAU]	Area %
1	7.612	BB	0.2764	1.13279e4	622.18732	95.2438
2	9.549	BBA	0.3051	565.68060	28.56109	4.7562
